# Energy transfer-enabled enantioselective photocyclization using a privileged Al–salen catalyst

**DOI:** 10.1038/s41557-025-01857-1

**Published:** 2025-07-17

**Authors:** Julia Soika, Carina Onneken, Thorben Wiegmann, Timo Stünkel, Tobias Morack, Leander Lindfeld, Marian Hebenbrock, Christian Mück-Lichtenfeld, Johannes Neugebauer, Ryan Gilmour

**Affiliations:** 1 Institute for Organic Chemistry, University of Münster, Münster, Germany; 2 Center for Multiscale Theory and Computation, University of Münster, Münster, Germany; 3 Institute for Organic Chemistry, University of Heidelberg, Heidelberg, Germany; 4 Institute for Inorganic and Analytical Chemistry, University of Münster, Münster, Germany

**Keywords:** Synthetic chemistry methodology, Asymmetric catalysis

## Abstract

Chiral catalysts that can engage multiple substrates, via distinct ground-state activation modes, to deliver enantioenriched products with high levels of fidelity are often described as ‘privileged’. Achieving generality in excited-state processes remains challenging, and efforts to identify privileged chiral photocatalysts are being intensively pursued. Aluminium–salen complexes are emergent contenders on account of their well-defined photophysical properties. Here we report the development of an enantioselective energy transfer (EnT) catalysis-enabled photocyclization of acrylanilides to expand the activation repertoire of Al–salen photocatalysts. This approach allows reactivity and enantioselectivity to be simultaneously regulated by an inexpensive, commercial chiral Al–salen complex upon irradiation at *λ* = 400 nm. Diverse cyclic products can be forged with high levels of enantioselectivity (up to 96:4 e.r.). Establishing this dichotomy in excited-state activation modes serves to consolidate the privileged status of chiral Al–salen complexes in enantioselective photocatalysis and to complement their ubiquity in ground-state regimes.

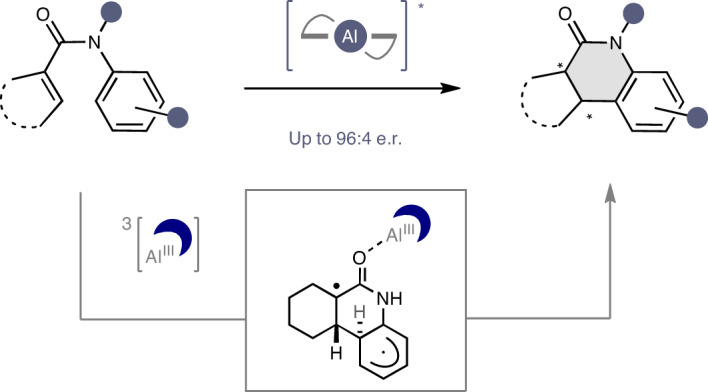

## Abstract (web summary)

Photocatalysts that can engage a range of substrates and regulate reactivity and enantioselectivity are highly desirable. Now, an enantioselective energy transfer catalysis-enabled photocyclization of acrylanilides has been developed. Reactivity and enantioselectivity are simultaneously regulated by a commercial chiral Al–salen complex upon irradiation, enabling the formation of diverse cyclic products with high levels of enantioselectivity.

## Main

**Fig. 1:**
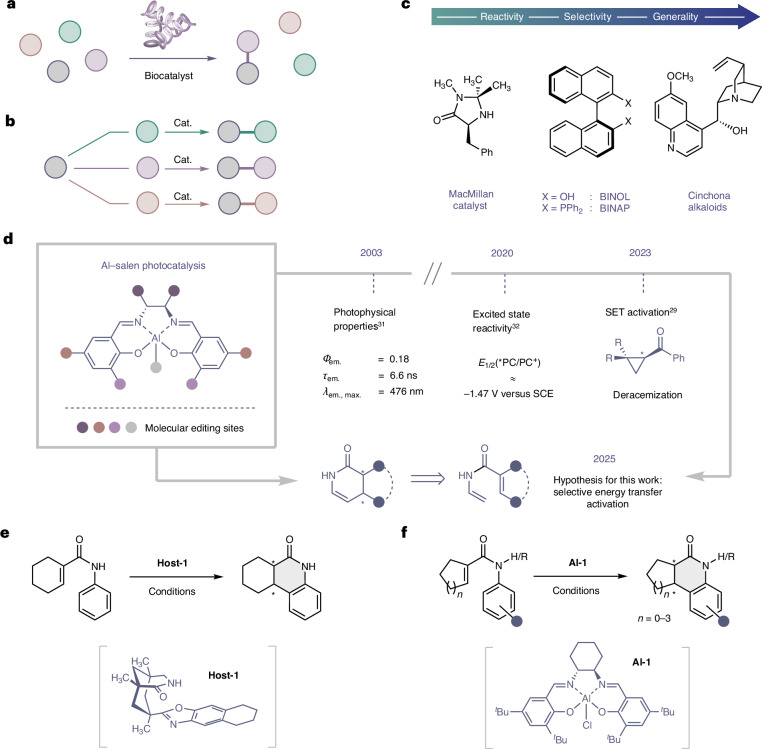
Generality of Al–salen photocatalysis. **a**, Selectivity of enzymatic catalysis. **b**, Generality of small-molecule catalysis. **c**, Selected examples of privileged chiral catalysts that operate in the ground state and unify reactivity and selectivity to enable generality. **d**, Emergence of Al–salen complexes as potential privileged chiral photocatalysts^29,31,32^. **e**, 6π-electrocyclization of an acrylanilide using a chiral host by direct excitation^44^. **f**, This work: enantioselective cyclization using an Al–salen photocatalyst. *λ*, wavelength; *Φ*, quantum yield; *τ*, lifetime; cat., catalyst; em., emission; max., maximum; PC, photocatalyst; *PC, excited state photocatalyst; SCE, saturated calomel electrode.

Reconciling the juxtaposition of selectivity and generality is a recurrent challenge in enantioselective small-molecule catalysis^1,2^. Driven by the socioeconomic importance of this technology^3–5^, this division has been bridged by a growing portfolio of versatile chiral catalysts that operate across a broad spectrum of transformations^6^. Successful catalyst blueprints are often bio-inspired^7^, and enabling functional promiscuity in a manner antipodal to enzymatic specificity^8^ has been a key driver of innovation (Fig. 1a). This creates the latitude necessary to rapidly penetrate new areas of chemical space (Fig. 1b)^9^. Orchestrated by well-defined architectures and substrate activation modes, enantioselectivity can often be rationalized a priori, thereby consolidating the status of these privileged actors in precision synthesis (Fig. 1c). Although the notion of privileged catalysts is common in ground-state reactivity^1,10^, extrapolating this conceptual paradigm to excited-state enantioselective scenarios represents a challenge in asymmetric synthesis^11–14^. Advancing this field requires new structure—activation guidelines to be delineated^15,16^ that reflect the energetic realities of asymmetric processes in which reactivity and chirality transfer occur in non-ground-state scenarios^17,18^. The renaissance of organic photochemistry provides an exciting opportunity to repurpose inexpensive, privileged chiral catalysts to simultaneously regulate reactivity and enable asymmetric induction in the presence of light as an external stimulus^19–21^.

Foundational examples from the groups of Bach^22–24^, Yoon^25,26^ and Meggers^27,28^ have propelled the development of enantioselective photocatalysis using substrate–catalyst ensembles that are appointed with specific recognition units. To complement these precision molecular design strategies, we recently validated quasi-*C*_2_-symmetric Al–salen complexes as efficient chiral photocatalysts for the deracemization of cyclopropanes via photo-induced single-electron transfer (SET; Fig. 1d)^29^. The success of these inexpensive chiral complexes is grounded in their favourable photophysical properties, which include photon absorption in the visible range of the electromagnetic spectrum, comparably high excited-state lifetimes, and the capacity to transfer a single electron upon excitation^30–32^. Given the transformative impact of quasi-*C*_2_-symmetric metal–salen complexes in asymmetric catalysis^33–37^, and in particular the Al–salen complexes by Jacobsen and colleagues^38–41^, extending their repertoire to excited-state reactions would be highly enabling. However, this requires generality to be established, and validation that chiral Al–salen complexes can effectively catalyse enantioselective excited-state processes via discrete activation modes. To complement the recent report of photo-induced SET^29^, evidence of enantioselective energy transfer catalysis^21,42^ using Al–salen complexes would be a persuasive argument towards this objective. To that end, the development of a highly enantioselective cyclization of simple acrylanilides was developed under the auspices of Al–salen photocatalysis (Fig. 1d). This transformation was inspired by the early work of Cleveland and Chapman^43^, and a seminal report by Bach et al. (Fig. 1e)^44^ describing the 6π-electrocyclization of a single acrylanilide by direct excitation in the ultraviolet (UV) range (*λ* = 300 nm) in the presence of a super-stoichiometric chiral host (57% enantiomeric excess). Key to our reaction design was the premise that, on irradiation with visible light, efficient energy transfer from the excited-state Al–salen to the substrate would enable a photocyclization event (Fig. 1f). Moreover, it was envisaged that Lewis coordination between the oxygen of the amide substrate and the central Al of the catalyst in the excited state would ensure that ring closure occurred within the confinement of the highly pre-organized chiral environment to deliver optically enriched products. In a broader sense, this approach would contribute to the current interest in enantioselective photocyclization reactions. Elegant studies by Smith and colleagues have demonstrated that combining an Ir(III) photosensitizer with a chiral Lewis acid complex can enable this challenging transformation^45^. Furthermore, Yoon and colleagues have leveraged a precision hydrogen-bonding interaction between an Ir(III) catalyst and the substrate to render 6π-photoelectrocyclizations highly enantioselective^46^.

## Results and discussion

To explore the feasibility of an enantioselective cyclization enabled by energy transfer photocatalysis, substrate **1a** bearing an unprotected amide was exposed to 10 mol% of commercially available Al–salen complex **Al-1** under irradiation at 400 nm (violet light) in dichloromethane (Table 1, entry 1). Gratifyingly, the products *trans*- and *cis*-**2a** were obtained in a combined yield of 44% with enantioenrichement observed for both diastereomers (*trans*-**2a**, 74:26 enantiomeric ratio (e.r.); *cis*-**2a**, 68:32 e.r.). The diastereomeric ratio (d.r.) of 71:29 favoured formation of the *trans*-product under these reaction conditions. Based on mechanistic investigations by Bach and colleagues^44^, it was evident that the enantiodetermining step (β to the carbonyl) was controlled by the photocatalyst. Competing protonation/H-shift pathways then account for the formation of two diastereomers. Encouraged by this initial result, the impact of structural and electronic changes at the editing site shown in Table 1 was investigated (a full catalyst screen is presented in Supplementary Table 1). Removal of either substituent of the salicylidene ring (catalysts **Al-2** to **Al-4**) led to substantial improvements in cyclization efficiency (up to 95%, entry 2), but at the expense of enantioselectivity (*trans*-**2a**, 63:37 e.r.; *cis*-**2a**, 55:45 e.r., entry 2). In contrast, augmenting the steric bias of one substituent from *tert*-butyl to adamantyl (catalyst **Al-5**, entry 5) led to enhanced enantiomeric ratios of 84:16 (*trans*-**2a**) and 80:20 (*cis*-**2a**), albeit with a reduced yield of 15%. The introduction of catalyst **Al-6** with a modified backbone (from cyclohexyl to phenyl groups, entry 6) inhibited both the yield (14%) and selectivity (*trans*-**2a**, 65:35 e.r.; *cis*-**2a**, 54:46 e.r.). Finally, changing the apical ligand from chloride to an oxygen-bridged dimeric structure (**Al-7**, entry 7) or a methyl group (**Al-8**, entry 8; Supplementary Table 1) had very marginal effects on enantioselectivity, but led to notable reductions in yields (42% and 36%, respectively). These data allowed further reaction optimization to be conducted with the commercial catalyst **Al-1**, beginning with a study of the impact of the reaction medium (a full solvent screen is presented in Supplementary Table 2). Reactions performed in acetone led to the formation of trace amounts of product (entry 9), whereas in acetonitrile (MeCN, entry 10) and toluene (entry 11), reduced yields (both 33%) and enantioselectivities were observed (*trans*-**2a**, 64:36 e.r.; *cis*-**2a**, 62:38 e.r.; *trans*-**2a**, 64:36 e.r.; *cis*-**2a**, 56:44 e.r., respectively).

**Table 1:** Optimization of the reaction conditions for the enantioselective photocyclization of **1a**


Entry	Catalyst	Solvent	Additive (1.5 equiv.)	*T* (°C)	Yield 2a (%)	d.r. (*trans*:*cis*)	e.r. *trans*	e.r. *cis*
1	**Al-1**	DCM	–	r.t.	44	71:29	74:26	68:32
2	**Al-2**	DCM	–	r.t.	95	55:45	63:37	55:45
3	**Al-3**	DCM	–	r.t.	64	64:36	67:33	53:47
4	**Al-4**	DCM	–	r.t.	83	63:37	67:33	57:43
5	**Al-5**	DCM	–	r.t.	15	66:34	84:16	80:20
6	**Al-6**	DCM	–	r.t.	14	71:29	65:35	54:46
7	**Al-7**	DCM	–	r.t.	42	67:33	23:77	27:73
8	**Al-8**	DCM	–	r.t.	36	71:29	73:27	75:25
9	**Al-1**	Acetone	–	r.t.	<5	ND	ND	ND
10	**Al-1**	MeCN	–	r.t.	33	56:44	64:36	62:38
11	**Al-1**	Toluene	–	r.t.	33	67:33	64:36	56:44
12	**Al-1**	DCM	^*n*^Bu_4_NCl	r.t.	67	67:33	78:22	80:20
13	**Al-1**	DCM	HFIP	r.t.	95	51:49	67:33	67:33
14^a^	**Al-1**	DCM	^*n*^Bu_4_NCl	−20	50	64:36	82:18	85:15
15^a,b^	**Al-1**	DCM	^*n*^Bu_4_NCl MS (3 Å)	−20	55	62:38	82:18	88:12
16^a,b,c^	**Al-1**	DCM	^*n*^Bu_4_NCl MS (3 Å)	−20	57	66:34	84:16	90:10
17^a,c,d^	**Al-1**	DCM	^*n*^Bu_4_NCl MS (3 Å)	−20	67	66:34	84:16	90:10

Unless stated otherwise, reactions were performed with **1a** (0.1 mmol), Al–salen catalyst (10 mol%) and additive (1.5 equiv.) in dichloromethane (DCM) under an argon atmosphere. Irradiation took place at 400 nm, and for irradiation at −20 °C a glass rod was used as an optical guiding rod. Isolated yields of combined *trans*- and *cis*-**2a** are reported. The d.r. was determined by ^1^H NMR spectroscopy; the e.r. was determined by high-performance liquid chromatography (HPLC) analysis on a chiral stationary phase. ^a^Reactions at −20 °C were run for 21 h; ^b^15 mg of molecular sieves were used; ^c^20 mol% of catalyst was used; ^d^6 mg of molecular sieves were used. r.t., room temperature; ND, not determined.

The addition of ^*n*^Bu_4_NCl had a beneficial effect on the reaction outcome (a detailed screen of additives is presented in Supplementary Table 3) (Table 1, entry 12), and its addition allowed **2a** to be generated in 67% yield with a d.r. of 67:33 (*trans*:*cis*), and with encouraging enantiomeric ratios of 78:22 (*trans*-**2a**) and 80:20 (*cis*-**2a**). It is tempting to speculate that ^*n*^Bu_4_NCl plays an important role in stabilizing the excited-state catalyst–substrate complex^47^. Yoon and colleagues observed a similar beneficial effect of this quaternary ammonium salt in the enantioselective conjugate addition of α-amino radicals by cooperative photoredox catalysis^48^. In the presence of hexafluoroisopropanol (HFIP, entry 13), product **2a** was formed almost quantitatively (95%) with an equimolar ratio of diastereomers (51:49 *trans*:*cis*). The enantioselectivity was lower (67:33 e.r.) for both diastereomers. The reaction with ^*n*^Bu_4_NCl was repeated at −20 °C (entry 14) and improved e.r. values of 82:18 (*trans*-**2a**) and 85:15 (*cis*-**2a**) could be achieved. The addition of a drying agent to reactions run at low temperatures proved to be pivotal, and the simple addition of molecular sieves (MS, entry 15) led to a notable improvement in yield without adversely affecting the enantioselectivity. Finally, increasing the catalyst loading and reducing the amounts of molecular sieves enabled product **2a** to be generated in 67% yield (84:16 (*trans*-**2a**) and 90:10 (*cis*-**2a**, entry 17)).

Having identified optimized reaction conditions, the scope of the transformation was investigated (Table 2). For an extended scope and limitations, including *meta*-substituted examples, see Supplementary Figs. 1 and 2. Gratifyingly, this enantioselective photocyclization catalysed by Al–salen **Al-1** proved to be compatible with secondary (**2a**) and tertiary amides, which complements existing results^45,46^. To explore the influence of changes to the aryl ring of the acrylanilide, *para*-substituted substrates (**2b**–**2h**) were investigated. These substrates proved to be compatible with the reaction conditions. In all cases, synthetically useful e.r. values were obtained for both diastereomers, with formation of the *trans*-products being favoured.

**Table 2:**
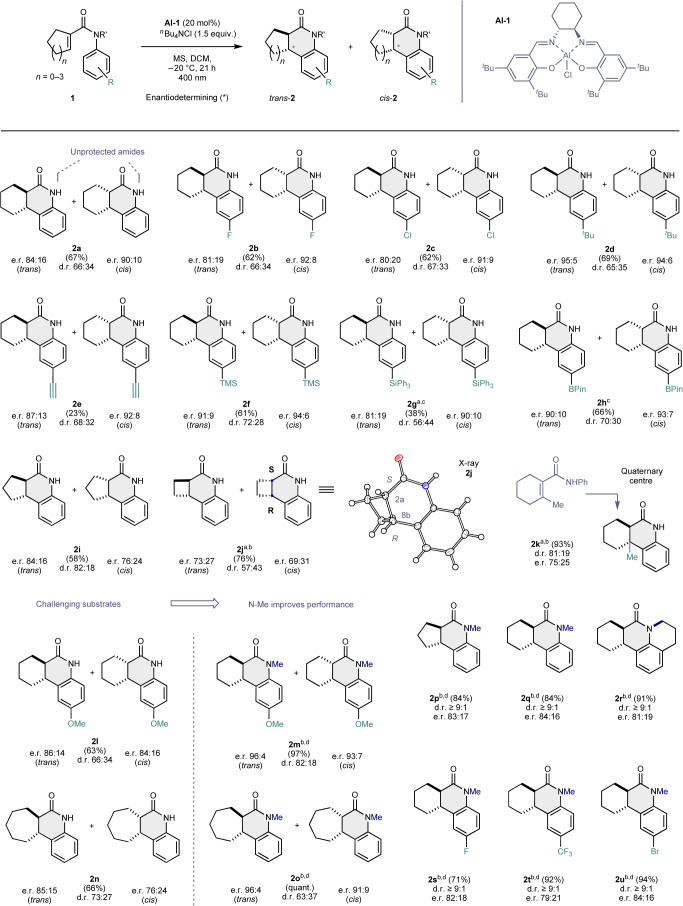
Scope of the enantioselective photocyclization

Unless stated otherwise, reactions were performed with substrate (0.1 mmol), **Al-1** (20 mol%), ^*n*^Bu_4_Cl (1.5 equiv.) and molecular sieves (3 Å, 6 mg) in DCM under an argon atmosphere. The reaction mixtures were irradiated at 400 nm and −20 °C for 21 h. Isolated yields of combined *trans*- and *cis*-products are reported. The d.r. was determined by ^1^H NMR spectroscopy; the e.r. was determined by HPLC analysis on a chiral stationary phase. ^a^65 h reaction time; ^b^HFIP (1.5 equiv.) was used instead of ^*n*^Bu_4_NCl; ^c^irradiated at r.t.; ^d^irradiated at −40 °C. quant., quantitative.

Halogen- (**2b**/**c**) and alkyl-substituents (**2d**/**e**) were tolerated, and enantioselectivities of up to 94:6 e.r. for *cis*-**2d** could be obtained. The broad functional-group tolerance of the transformation is reflected in the formation of silylated (**2f**/**g**, up to 94:6) and borylated adducts (up to e.r. 90:10 and 93:7 (*trans*- and *cis*-**2h**, respectively)). Decreasing or increasing the ring size of the cyclic olefin was also tolerated and enabled products **2i** and **2j** to be generated. In the case of *cis*-**2j**, the optical purity could be improved by facile recrystallization. This served a secondary purpose in enabling the absolute configuration of *cis*-**2j** to be assigned as (2a*S*, 8b*R*) (Table 2, insert; CCDC no. 2368374). Finally, product **2k**, possessing a quaternary stereocenter, could be formed from the tetra-substituted alkene precursor in 95% yield with e.r. 81:19 for the *trans*-diastereomer, which was the major product. In the case of more challenging substrates such as **1l** and **1n**, the performance could be improved by up to 15% by *N*-methylation through enhanced structural pre-organization before cyclization. This enabled tricycles **2m** and **2o** to be forged in 97% and quantitative yield, respectively. Consistently high levels of enantioselectivity were observed for both diastereomeric sets (*trans*-**2m** 96:4 and *cis*-**2m** 93:7; *trans*-**2o** 96:4 and *cis*-**2o** 91:9).

To further expand this *N*-Me amide scope, revised reaction conditions were employed in which HFIP was applied as an additive instead of ^*n*^Bu_4_NCl and the reactions were performed at a lower temperature (−40 °C). This enabled the 5- and 6-membered adducts **2p** and **2q** to be generated, as well as a challenging tetracyclic adduct **2r** (91% yield, e.r. 81:19). It is interesting to note that the *trans*:*cis* selectivity was ≥9:1 in all cases. Introducing electron-withdrawing substituents to complement the methoxy-derivative **2m** was also successful, as illustrated by compounds **2s**–**2u** (up to 94% yield, d.r. ≥9:1, up to 84:16 e.r.).

**Fig. 2:**
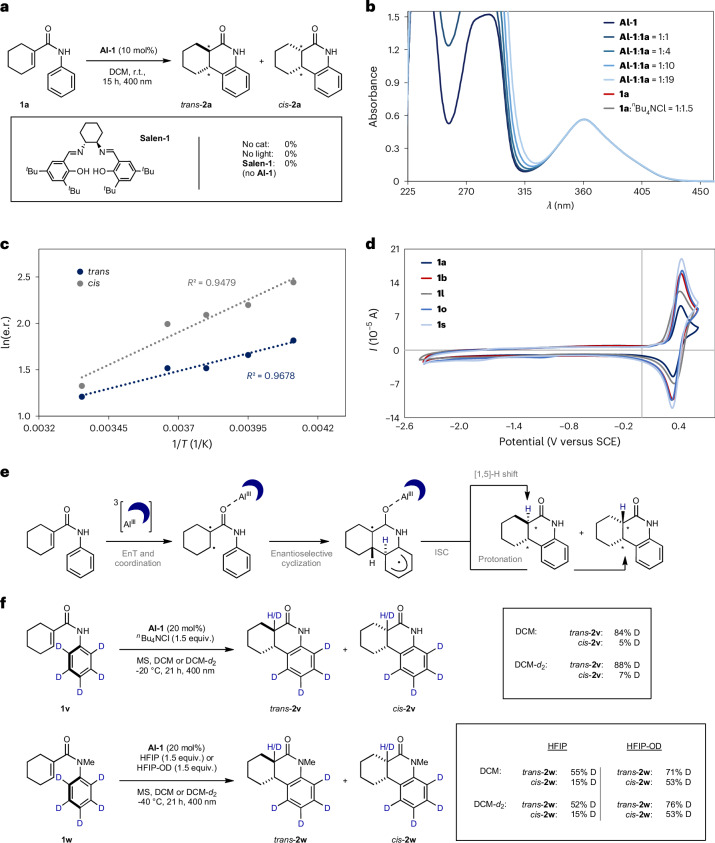
Mechanistic investigations. **a**, Control reactions. The reactions were performed on a 0.1 mmol scale. **b**, UV–vis absorption spectra of **1a** and **Al-1** (each *c* = 0.05 mM in DCM) and mixtures. **c**, Effect of varying the temperature on the e.r. The reactions were performed on a 0.1 mmol scale. **d**, Cyclic voltammograms of **1a**, **1b**, **1l**, **1o** and **1s** (in MeCN). **e**, A potential reaction mechanism for the photocyclization catalysed by **Al-1**. **f**, Deuteration experiments. The reactions were performed on a 0.1 mmol scale. Source data

Attention was then turned towards mechanistic investigations to gain further insights into this enantioselective energy-transfer process (Fig. 2). Control reactions in the absence of catalyst **Al-1**, or shielded from light, did not lead to product formation, but instead the starting material **1a** was recovered quantitatively (Fig. 2a). Furthermore, no product was observed when **Al-1** was replaced with the salen ligand **Salen-1**, indicating that substrate coordination to the Al centre is important in simultaneously regulating reactivity and selectivity. The lack of reactivity in the absence of the catalyst is further supported by UV–vis spectroscopy: only the catalyst absorbs electromagnetic radiation at 400 nm, while the absorption maximum of the substrate **1a** is located at ~260 nm (Fig. 2b). Titration of substrate **1a** to the catalyst did not cause a bathochromic shift of the absorption band (*λ*_max._ ≈ 360 nm), indicating that Lewis acid–base coordination between the catalyst and the substrate is not operational in the ground state. This was further verified by ^13^C NMR spectroscopy (Supplementary Fig. 15). Exploring the temperature dependence of the reaction revealed the expected linear correlation between ln(e.r.) and the reciprocal of the temperature (Fig. 2c). To gain additional insight into the nature of the interaction between the excited-state catalyst and the substrate, cyclic voltammetry of various substrates was performed. As is evident from Fig. 2d, no reduction was observed for any of the substrates investigated in MeCN. Consequently, SET to the substrates is highly improbable, and a mechanism based on energy transfer from the catalyst in its triplet excited state is likely to be operational (Fig. 2e). This is further supported by computational investigations (vide infra). Following energy transfer, the excited substrate undergoes cyclization, which is enantiodetermining. Subsequent intersystem crossing (ISC) leads to either a [1,5]-H shift or external protonation to furnish the final product diastereomers. To distinguish between these potential pathways, experiments with deuterated substrates **1v** and **1w** were conducted (Fig. 2f). Replacement of the phenyl ring of acrylanilide **1a** with a per-deuterated ring (substrate **1v**) led to high levels of deuterium (D) incorporation at the 6a-position of *trans*-**2v** (84% D), whereas in *cis*-**2v** only 5% D-incorporation was observed. This suggests that a [1,5]-H shift is operative in the formation of the *trans*-systems, whereas the corresponding *cis*-diastereomers are formed via external protonation. To investigate the potential role of the solvent, the experiment was repeated in deuterated dichloromethane (DCM). Similar degrees of deuterium incorporation were observed, thereby excluding the solvent as a proton source. In the case of secondary amides, a potential hydrogen source is the unprotected amide. Therefore, further experiments were performed with deuterated substrate **1w**, which contains an *N*-methyl group. In alignment with the previous observations, a higher level of deuterium incorporation was observed for *trans*-**2w** (55%) than for *cis*-**2w** (15%). Because the photocyclization of **1w** was facilitated by the addition of HFIP (vide supra), the possibility of this additive serving as a protonation agent was considered. Conducting the experiment with HFIP-OD led to a substantial increase in deuterium incorporation, and this was especially pronounced in the *cis*-diastereomer: from 15% D with HFIP-OH to 53% D with HFIP-OD. Repeating the experiment with DCM-*d*_2_ did not reveal a solvent influence, but rather verified the initial results. These findings further substantiate the hypothesis that the *cis*-products arise primarily via external protonation, but a [1,5]-H shift dominates in the formation of the *trans*-diastereomers.

Further experiments applying non-deuterated substrates in combination with deuterated solvent and/or HFIP as the proton source are in line with the presented results (Supplementary Tables 8 and 9 provide more details).

**Fig. 3:**
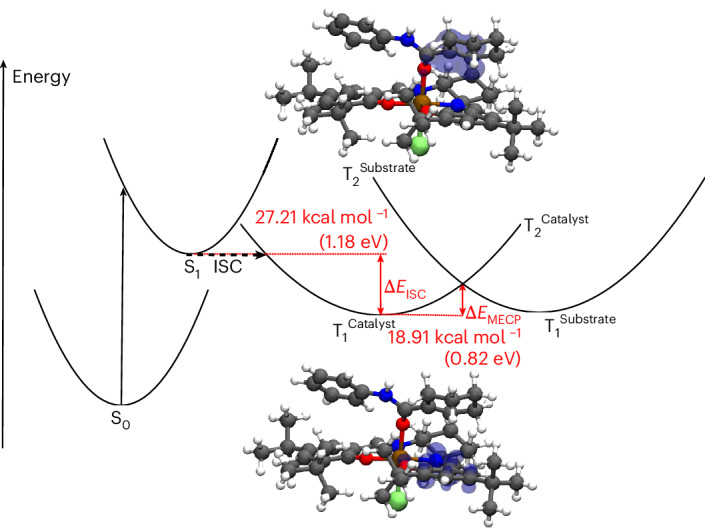
Reaction mechanism. Schematic illustration of the proposed path across multiple potential energy surfaces from the photoexcitation to the triplet energy transfer (EnT).

To further support the involvement of an energy-transfer mechanism, a detailed computational investigation was conducted. This enabled an electron-transfer mechanism to be ruled unlikely. Calculation of ground- and excited-state redox potentials in acetonitrile at 25 °C and in DCM at −20 °C revealed a mismatch of 0.61 eV and 0.50 eV for the highest investigated excited state (S_1_), respectively. The S_0_ and T_1_ redox potentials show a larger mismatch (Supplementary Table 14). Furthermore, the frontier molecular orbitals involved in the excitation show a significant localization solely on the catalyst, which contradicts an electron-transfer mechanism. In contrast, this investigation revealed that the postulated triplet energy transfer mechanism was plausible. Calculated triplet excitation-energy transfer couplings^49^ for the complex studied (**Al-1-1a**) are about one order of magnitude larger than the couplings of the related complex, which was shown to undergo electron transfer^29^. Density functional theory (DFT) calculations suggest a slight uphill energy-transfer mechanism as the triplet energy of the catalyst is ~0.17 eV higher than the triplet energy of the substrate. Upon coordination with the acrylanilide, however, the energy of the catalyst-localized triplet state increases, and model calculations suggest that the energy of the substrate-localized triplet state within the complex decreases (Supplementary Table 15). Consequently, the energy gap between the two triplet states is further reduced. A complete active space self-consistent field (CASSCF) single-point calculation on the DFT minimum-energy structure of the (catalyst-localized) T_1_ state revealed that the spin density in the T_2_ state is localized on the substrate. This further supports the hypothesis of an uphill triplet energy transfer mechanism. A minimum energy crossing point (MECP) between these two states was calculated to obtain the molecular structure for the crossing into a substrate-localized triplet state. The molecular structure obtained shows little change compared to the minimum-energy structure of the catalyst-localized triplet state, and the changes that are observed are in agreement with the expected motion along the reaction coordinate: this further supports the hypothesis of energy transfer. A Marcus-theory based approximation^50^ revealed a barrier of 0.82 eV for the energy transfer. The energy released by relaxation after the intersystem crossing is in the same energy range. Based on these CASSCF and DFT calculations, it is proposed that an uphill triplet energy-transfer mechanism following the path schematically shown in Fig. 3 is the most probable scenario. Following an initial excitation into the S_1_ state of the catalyst we assume that an intersystem crossing to the catalyst-localized T_1_ state occurs. This catalyst-localized state crosses with another substrate-localized triplet state, which becomes the lowest-energy triplet state beyond the crossing point. The energy barrier for this crossing can be overcome if the adiabatic energy difference of the S_1_ and T_1_ (Δ*E*_ISC_) states is larger than the energy barrier to the minimum energy crossing point (Δ*E*_MECP_).

To contribute to the privileged status of Al–salen complexes in asymmetric catalysis, a highly enantioselective photocyclization of simple acrylanilides mediated by triplet energy transfer catalysis is disclosed. Validating this second photoactivation mode in asymmetric photocatalysis substantially expands the operational capabilities of these commercial, earth-abundant complexes and further cements their privileged status.

## Methods

### General procedure for photocatalytic 6π-cyclization at –20 °C (standard conditions)

In a glovebox, a vial was charged with a stir bar, acrylanilide (0.10 mmol, 1.00 equiv.), Al catalyst **Al-1** (12.1 mg, 0.02 mmol, 20 mol%), tetrabutylammonium chloride (41.7 mg, 0.15 mmol, 1.50 equiv.) and 3-Å MS (6.0 mg). Dry DCM (1.5 ml) was added, the vial was capped with a cap with pre-slit septum, and the mixture was stirred until the catalyst and the acrylanilide were fully dissolved. Insertion of the glass rod into the solution was followed by sealing with Parafilm. The vial was removed from the glovebox, placed in the low-temperature photoreactor and cooled to −20 °C, before being irradiated at 400 nm for 21 h. Afterwards, the reaction mixture was warmed to room temperature and concentrated under reduced pressure. Following purification by column chromatography (SiO_2_, *n*-pentane/EtOAc), the product was obtained.

### General procedure for photocatalytic 6π-cyclization at room temperature

In a glovebox, a pressure tube was charged with a stir bar, acrylanilide (0.10 mmol, 1.00 equiv.), Al catalyst **Al-1** (12.1 mg, 0.02 mmol, 20 mol%), tetrabutylammonium chloride (41.7 mg, 0.15 mmol, 1.50 equiv.) and 3-Å MS (6.0 mg). Dry DCM (1.5 ml) was added, and the tube was sealed, removed from the glovebox, and irradiated at 400 nm and r.t. for 21 h. Afterwards, the reaction mixture was concentrated under reduced pressure. Following purification by column chromatography (SiO_2_, *n*-pentane/EtOAc), the product was obtained.

*Note*: in reactions with HFIP instead of tetrabutylammonium chloride, HFIP (15.8 µl, 0.15 mmol, 1.50 equiv.) was added following the addition of DCM.

### Computational details

Ground-state minimum energy geometries of the catalyst–substrate complex were found by a conformational search using CREST (v.2.12)^51,52^ employing GFN2-xTB (v.6.5.1)^53,54^, followed by further refinements with the workflow implemented in CENSO (v.1.2.0)^55^. For these stepwise refinements, the PBEh-3c^56^ and PBE(-D3)^57–59^ functionals and def2-TZVP^60^ basis set were used. The Gibbs free energy was calculated under reaction conditions using recalculated electronic energies (PW6B95(-D3)^61^/def2-TZVP). Solvation energies were calculated using COSMO-RS^62,63^. Only those conformers were considered that were in a 2 kcal mol^−1^ energy range to the conformer lowest in energy and showed no imaginary frequencies larger (in absolute value) than 10 cm^−1^. These calculations were conducted using TURBOMOLE (v.7.8)^64,65^.

The orbital transitions in the investigation of charge-transfer excitations were obtained from linear-response time-dependent DFT (TDDFT) using the range-separated hybrid functional CAM-B3LYP^66^ and the def2-TZVP basis set with Serenity (v.1.6.1)^67–69^.

Ground-state redox potentials were calculated using CAM-B3LYP(-D3)/def2-TZVP. Solvation effects were considered using COSMO-RS. Excited-state redox potentials were approximated via adiabatic electronic excitation energies. For the T_1_ state, this energy was obtained by a ΔSCF-like approach, whereas TDDFT was used for S_1_. These calculations were conducted using ORCA (v.5.0.3)^70^.

Electronic couplings were calculated from a projection-based subsystem TDDFT (sTDDFT) calculation performed using Serenity following the procedure in ref. ^49^ using CAM-B3LYP/def2-SVP^60^. The couplings were obtained from a coupled (FDEc-TDDFT) calculation within the Tamm–Dancoff approximation (TDA). For this purpose, the lowest-energy conformer of the conformational search was used.

Triplet-state minimum geometries were reoptimized using the B3LYP(-D3)^71,72^ functional and the def2-TZVP basis set. Electronic energies were recalculated using different functionals and the def2-TZVPP^60^ basis set with ORCA.

The energy barrier for the energy transfer was approximated using Marcus theory, similarly to the description in ref. ^50^, using ORCA.

MECP optimizations were performed using SA(2)-CAS(6,5)SCF and the valence double Zeta ANO-S basis set^73^ using OpenMolcas (v.24.02)^74^.

In view of the flexible molecular structure, a Boltzmann-weighted optical rotation was calculated using a conformational ensemble. For this purpose, the electronic energies were recalculated using DLPNO-CCSD(T_0_)^75,76^. Specific optical rotations were calculated at a wavelength of 589 nm via TDDFT calculations employing the CAM-B3LYP functional and def2-TZVP basis set. The coupled cluster and TDDFT calculations were performed using Serenity.

## Online content

Any methods, additional references, Nature Portfolio reporting summaries, source data, extended data, supplementary information, acknowledgements, peer review information; details of author contributions and competing interests; and statements of data and code availability are available at 10.1038/s41557-025-01857-1.

## Supplementary information

Supplementary Information Additional scope, synthesis of starting materials and products, Supplementary Figs. 1–29 and Tables 1–36, computational analysis, NMR spectra.

Supplementary Data 1 Crystallographic data for compound 2j-*cis*; CCDC reference 2368374.

Supplementary Data 2 Crystallographic data for compound *rac*-2j-*trans*; CCDC reference 2368376.

Supplementary Data 3 Crystallographic data for compound *rac*-2k-*trans*; CCDC reference 2368377.

Supplementary Data 4 Crystallographic data for compound *rac*-2n-*trans*; CCDC reference 2368375.

Supplementary Data 5 *xyz*-data for all calculated structures.

## Source data

Source Data Fig. 2 Source data for UV–vis and CVs.

## Data Availability

Crystallographic data for the structures reported in this Article have been deposited at the Cambridge Crystallographic Data Centre, under deposition nos. CCDC 2368376 (for ***rac*****-2j-*****trans***), 2368374 (for **2j-*****cis***), 2368375 (for ***rac-*****2n-*****trans***) and 2368377 (for ***rac*****-2k-*****trans***). These data can be obtained free of charge from The Cambridge Crystallographic Data Centre (http://www.ccdc.cam.ac.uk/data_request/cif). Supplementary Information is available for this paper. All data are available in the main text or the Supplementary Information. Source data are provided with this paper.
