# Supplementary material for: Energy transfer-enabled enantioselective photocyclization using a privileged Al–salen catalyst
Source: Nat Chem. 2025 Jul 17;17(9):1383–90. doi: 10.1038/s41557-025-01857-1 (PMC12411254; doi:10.1038/s41557-025-01857-1)
Supplement: Supplementary file 1 — Additional scope, synthesis of starting materials and products, Supplementary Figs. 1–29 and Tables 1–36, computational analysis, NMR spectra. [file 41557_2025_1857_MOESM1_ESM.pdf]

nature chemistry

Supplementary information https://doi.org/10.1038/s41557-025-01857-1

Energy transfer-enabled enantioselective photocyclization using a privileged Al–salen catalyst

In the format provided by the authors and unedited

Table of Content

- General Information 2
- Additional Scope and Limitations 5
- Preparation of Starting Materials 7
- Preparation of Catalysts 39
- 6π-Photocyclization: Procedure and Product Characterization 43
- Optimization of 6π-Photocyclization Reaction Conditions 115
- Mechanistic Investigations 120
- Computational Analysis 140
- Single Crystal X-ray Diffractometry 160
- NMR Spectra 168
- References 276

General Information

All chemicals were purchased as reagent grade and used without further purification unless otherwise stated. Solvents for purification (extraction and chromatography) were purchased as technical grade and distilled on the rotary evaporator prior to use. Solvents for photochemical reactions were degassed by purging with argon for 30 min. For column chromatography SiO_2 (40-63 µm for Flash-Chromatography, *VWR Chemicals*) was used as stationary phase. Analytical thin layer chromatography (TLC) was performed on aluminum foil pre-coated with *SiO_2-60 F_{254}* (*Merck*) and visualized with a UV-lamp (254 nm) and $KMnO_4$ or CAM solution. Concentration *in vacuo* was performed at ~10 mbar and 45 °C, drying at ~10^{-2} mbar and room temperature. NMR spectra were measured by the NMR service of the Organisch-Chemisches Institut, Universität Münster on a *Bruker AV300*, *Bruker Avance II 400, Bruker NEO 400*, *Agilent DD2 500* or an *Agilent DD2 600* spectrometers at room temperature. The chemical shifts are referenced to the residual solvent peak as internal standard.[1] The resonance multiplicity is abbreviated as: s (singlet), d (doublet), t (triplet), q (quadruplet), p (pentet), sext (sextet), sep (septet), m (multiplet) and br (broad). Assignments of unknown compounds are based on DEPT, COSY (HH), HMBC, HSQC, TOESY and NOESY spectra. Melting points were measured on a *Büchi B-545* melting-point apparatus in open capillaries. IR spectra were recorded on a *Perkin-Elmer 100 FT-IR* spectrometer, selected adsorption bands are reported in wavenumbers (cm^{-1}) and intensities are reported as: w (weak), m (medium), s (strong) and b (broad). High-resolution mass spectra (HR-ESI) were measured by the MS service of the Organisch-Chemisches Institut, Universität Münster. UV/vis absorption spectra were measured on an Agilent Cary 60 spectrophotometer in a 1 cm quartz cuvette. Optical rotations were measured on a *Perkin-Elmer 341 polarimeter*. Photochemical 6π-cyclization reactions at low temperatures were performed utilizing a set-up of 6 individual *Inolux 5 W High Power LED's* (IN-C39ATO, emission maximum 399 nm) on a star-platine with a radiation angle of 30°. The forward current per chip was set to 1200 mA (emission spectrum see Supporting Information Figure 1). A custom-made quartz-glass rod was used as an optical guiding rod. Temperature control was achieved by using a Julabo FT902 cryostat and acetone as liquid carrier (for reaction set-up see Supporting Information Figure 2). Photochemical 6π-cyclization reactions at room temperature were performed utilizing a set-up of 4 Avonec 3 W High Power LED on star-platine with a radiation angle of 120° (emission spectrum see Supporting Information Figure 3). The forward current per chip was set to 700 mA and the

resulting radiant flux was 1000 mW. The distance between the reaction vessels and the UV-lamp was set at approximately 0.5 cm for all reactions. The enantiomeric ratio of the products was determined by chiral HPLC on an *Agilent 1200* HPLC system or on an *Agilent 1260 Infinity II* HPLC system using a Chiralpak AS-H (5 µm, 250·4.6 mm), a Chiralpak AD-H (5 µm, 250·4.6 mm), a Chiralcel OJ-H (5 µm, 250·4.6 mm) or a ReproSil Chiral-NR (8 µm, 250·4.6 mm) column and *n*-hexane/*i*-propanol as eluent. Unless otherwise noted, the HPLC were recorded at 25 °C. HPLC traces in solvent mixtures of *n*-hexane/*i*-propanol ≤ 15:85 were performed at 35 °C. Cyclic voltammetry was measured on a *Metrohm compact potentiostat Autolab PGSTAT204* with a TSC1600 closed measuring cell with glassy carbon working electrode, Ag-pseudoreference and Pt-counter electrode. Ferrocene was used as an internal reference.

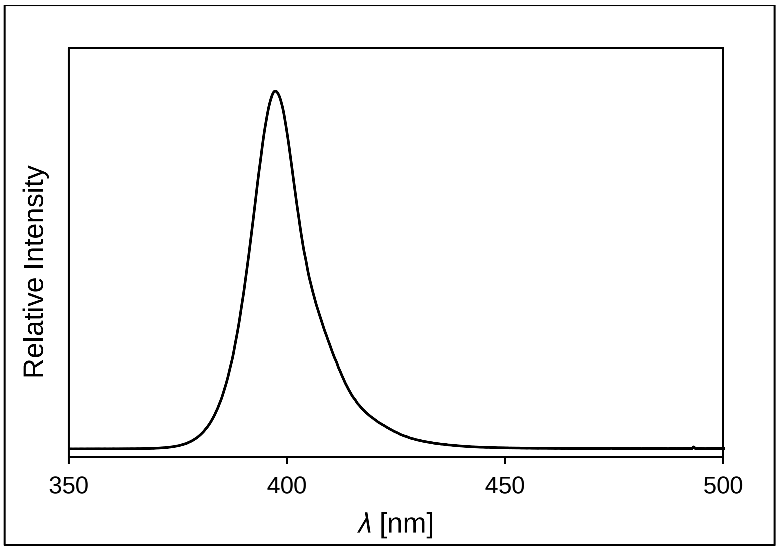

Figure 1: Emission spectrum of the LED’s utilized for 6π-cyclization reactions at low temperatures.

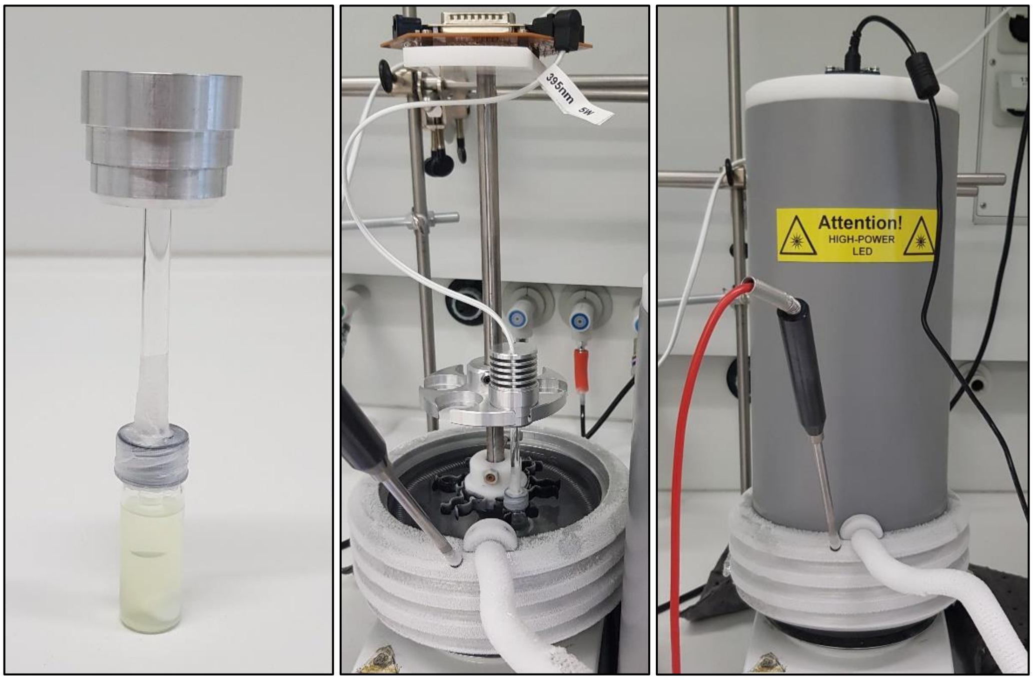

Figure 2: Set-up for low-temperature photoreactions: reaction vessel with inserted glass rod as an optical guiding rod (left), irradiation set-up with opened lid (middle), irradiation set-up eith closed lid (right).[2]

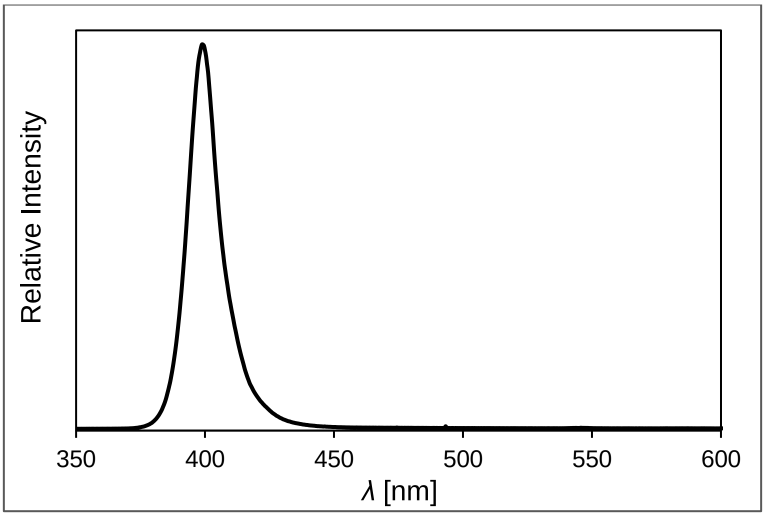

Figure 3: Emission spectrum of the LED's utilized for 6π-cyclization reactions at room temperature.

Additional Scope and Limitations

Additional scope examples:

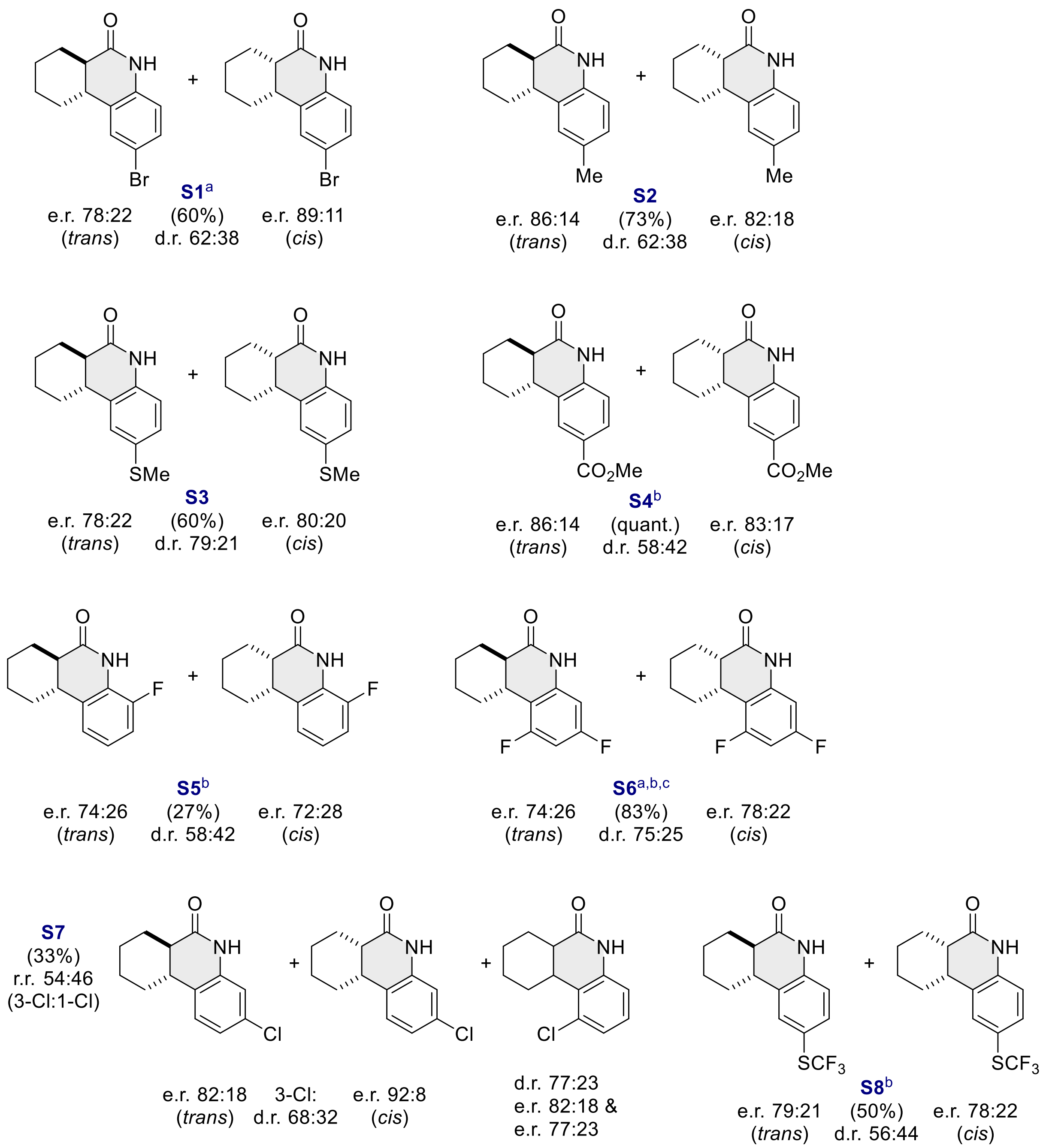

Figure 4: **Additional scope and limitation.** Unless stated otherwise, reactions were performed with substrate (0.1 mmol), **AI-1** (20 mol%), nBu_4Cl (1.5 eq.) and molecular sieves (3 Å, 6 mg) in DCM (1.5 mL) under argon atmosphere. Reaction mixtures were irradiated at 400 nm and –20 °C for 21 h. a) 65 h reaction time; b) HFIP (1.5 eq.) was used instead of nBu_4NCl; c) irradiated at room temperature; d) irradiated at –40 °C.

The additional scope examples demonstrate that thioethers and ester as well as meta-substitution of the aromatic ring are well tolerated by the developed protocol. Only ortho-F substituted **S5** was obtained in low yield (27%) with slightly diminished enantioselectivity.

Challenging substrates:

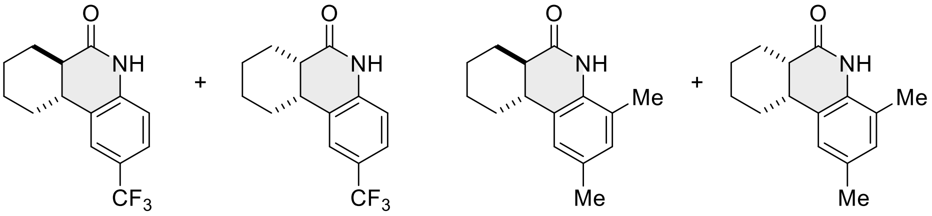

S9[a,c] (23%) d.r. 48:52; e.r. 67:33 (*trans*); e.r. 65:35 (*cis*)

S10 (63%) d.r. 75:25; e.r. 50:50 (*trans*); e.r. 56:44 (*cis*)

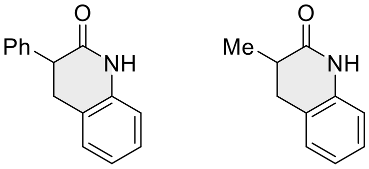

S11 (33%) e.r. 73:27

S12 (30%) e.r. 51:49

Failed substrates: (< 10% yield)

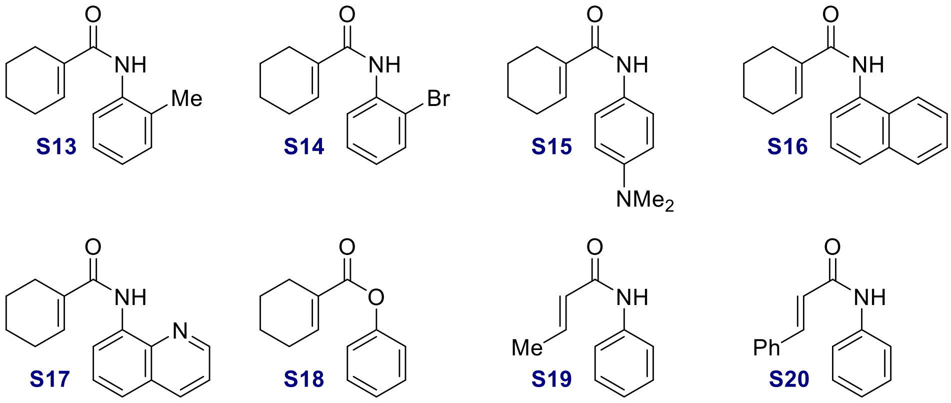

***Figure 5:* Challenging scope and limitations.** Unless stated otherwise, reactions were performed with substrate (0.1 mmol), **Al-1** (20 mol%), nBu_4Cl (1.5 eq.) and molecular sieves (3 Å, 6 mg) in DCM (1.5 mL) under argon atmosphere. Reaction mixtures were irradiated at 400 nm and –20 °C for 21 h. a) 65 h reaction time; b) HFIP (1.5 eq.) was used instead of nBu_4NCl; c) irradiated at room temperature; d) irradiated at –40 °C.

Photocyclization of an electron-poor substrate (*p*-CF_3) was low-yielding and under irradiation at room temperature only low enantioselectivity was obtained (*trans*-**S9** e.r. 67:33; *cis*-**S9** e.r. 65:35). Substrates with *ortho*-substitution (other than F) were unreactive (**S13**, **S14**) or cyclization was unselective (**S10**). Furthermore, amine groups or extended π-systems were not tolerated (**S15** – **S17**). Acyclic acrylanilides **S11** and **S12** were less reactive under standard conditions, however the obtained e.r. of 73:27 for **S11** is encouraging. For acyclic acrylanilides **S19** and **S20** only E/Z isomerization was observed.

Preparation of Starting Materials

General Procedure A: Synthesis of Acrylanilides via Acyl Chloride

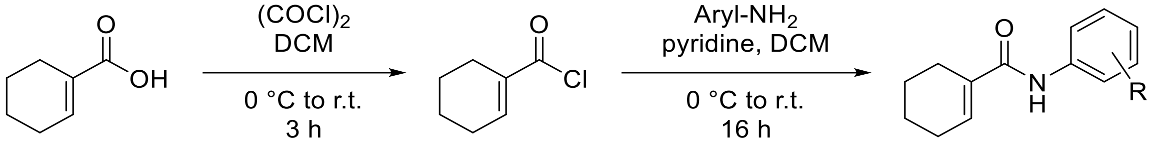

Cyclohex-1-ene-1-carboxylic acid (1.00 eq.) and DMF (1 drop/mmol) were dissolved in dry DCM (0.5 M). The solution was cooled to 0 °C and oxalyl chloride (1.20 eq.) was added under an argon atmosphere. The solution was stirred at room temperature for 3 h. The solvent was removed under reduced pressure and the acyl chloride was used in the next step without further purification.

The crude acyl chloride (1.00 eq.) was dissolved in dry DCM and the specified aniline (1.10 eq.) was added. The solution was cooled to 0 °C and pyridine (2.20 eq.) was added. The solution was allowed to slowly warm to room temperature and stirred for 16 h. DCM was added and the solution was washed successively with water, aqueous HCl (1 M) and brine. The crude acrylanilides were purified by column chromatography and/or recrystallization to yield the pure products.

General Procedure B: Synthesis of Acrylanilides with Coupling Reagents

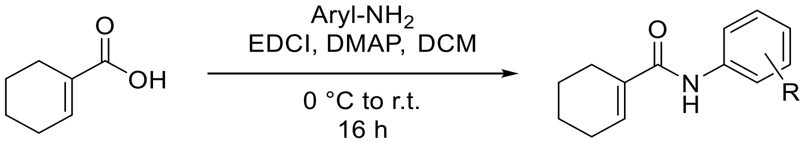

Cyclohex-1-ene-1-carboxylic acid (1.00 eq.) was dissolved in DCM (0.2 M) and aniline (1.10 eq.) was added. The mixture was cooled to 0 °C before EDCI (1.20 eq.) and DMAP (0.10 eq.) were added. The reaction was allowed to warm to room temperature and stirred for 16 h. The reaction was quenched by addition of NH_4Cl (aq., sat.), the layers were separated, and the aqueous layer was extracted with DCM (3x). The combined organic layers were washed with brine, dried over anhydrous $MgSO_4$ and concentrated *in vacuo*. The crude acrylanilides were purified by column chromatography and/or recrystallization to yield the pure products.

General Procedure C: Methylation of Acrylanilides

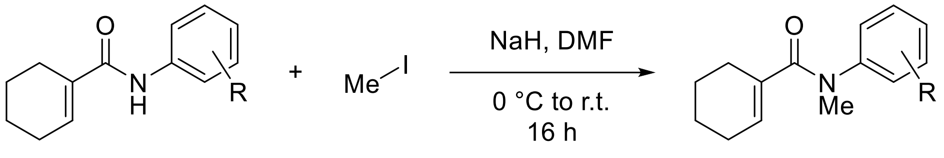

Under argon atmosphere at 0 °C, sodium hydride (60% in mineral oil, 1.50 eq.) was added to a solution of the respective acrylanilide (1.00 eq.) in dry DMF (0.3 M) and the mixture was stirred for 10 min. Methyl iodide (2.00 eq.) was added dropwise at 0 °C, the solution was allowed to warm to room temperature and stirred for 16 h. Water and EtOAc were added and the layers were separated. The aqueous layer was extracted with EtOAc twice and the combined organic layers were washed with saturated aqueous NaCl solution and dried over anhydrous $MgSO_4$. The solvent was removed under reduced pressure. Purification by column chromatography (SiO_2, *n*-pentane/EtOAc) yielded the pure products.

***N*-Phenylcyclohex-1-ene-1-carboxamide (1a)**

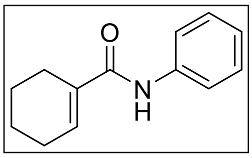

According to General Procedure **A**, cyclohex-1-ene-1-carboxylic acid (1.89 g, 15.00 mmol, 1.00 eq.) and aniline (1.51 mL, 16.50 mmol, 1.10 eq.) were converted to **1a**. Purification by column chromatography (SiO_2, 7% EtOAc in *n*-pentane) and subsequent recrystallization from DCM/*n*-hexane yielded the product as a white solid (2.55 g, 12.7 mmol, 85% over 2 steps).

R_f = 0.50 (*n*-pentane/EtOAc 8:2).

^{1}H NMR (400 MHz, $CDCl_3$): δ = 7.55 (dd, *J* = 8.6, 1.2 Hz, 2H), 7.38 (s, 1H), 7.33 (dd, *J* = 8.4, 7.3 Hz, 2H), 7.13 – 7.07 (m, 1H), 6.74 (tt, *J* = 3.9, 1.7 Hz, 1H), 2.35 (ddq, *J* = 6.1, 4.5, 2.2 Hz, 2H), 2.23 (dp, *J* = 8.8, 3.1 Hz, 2H), 1.78 – 1.69 (m, 2H), 1.69 – 1.59 (m, 2H) ppm.

HR-ESI-MS: m/z: 224.10419 ($[M-Cl]^+$, calcd. for $C_{13}H_{15}NONa^+$: 224.10458).

Mp = 119 – 122 °C.

Analytical data in agreement with literature.[3]

N-(4-Fluorophenyl)cyclohex-1-ene-1-carboxamide (1b)

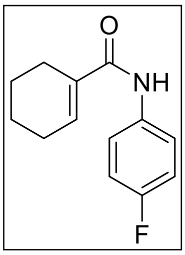

According to General Procedure **A**, cyclohex-1-ene-1-carboxylic acid (631 mg, 5.00 mmol, 1.00 eq.) and 4-fluoroaniline (0.52 mL, 5.5 mmol, 1.1 eq.) were converted to **1b**. Purification by column chromatography (SiO_2, 7-10% EtOAc in *n*-pentane) yielded the product as a white solid (836 mg, 3.81 mmol, 76% over 2 steps).

R_f = 0.44 (*n*-pentane/EtOAc 8:2).

^{1}H NMR (400 MHz, $CDCl_3$): δ = 7.56 – 7.47 (m, 2H), 7.43 (s, 1H), 7.05 – 6.94 (m, 2H), 6.72 (tt, *J* = 3.8, 1.7 Hz, 1H), 2.33 (ddt, *J* = 8.3, 4.3, 2.1 Hz, 2H), 2.21 (ddq, *J* = 8.0, 5.8, 2.8 Hz, 2H), 1.72 (tdd, *J* = 8.3, 5.1, 2.7 Hz, 2H), 1.63 (dtt, *J* = 8.9, 5.8, 2.5 Hz, 2H) ppm.

^{19}F NMR (377 MHz, $CDCl_3$): δ = –118.33 ppm.

HR-ESI-MS: *m/z*: 242.09510 ($[M+Na]^+$, calcd. for $C_{13}H_{14}NOFNa^+$: 242.09516).

Mp = 117 – 119 °C.

Analytical data in agreement with literature.[4]

N-(4-Chlorophenyl)cyclohex-1-ene-1-carboxamide (1c)

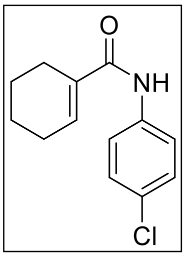

According to General Procedure **A**, cyclohex-1-ene-1-carboxylic acid (379 mg, 3.00 mmol, 1.00 eq.) and 4-chloroaniline (421 mg, 3.30 mmol, 1.10 eq.) were converted to **1c**. Purification by column chromatography (SiO_2, 12% EtOAc in *n*-pentane) yielded the product as a white solid (618 mg, 2.62 mmol, 87% over 2 steps).

R_f = 0.35 (15% EtOAc in *n*-pentane).

^{1}H NMR (400 MHz, $CDCl_3$): δ = 7.54 – 7.47 (m, 2H), 7.40 (s, 1H), 7.31 – 7.26 (m, 2H), 6.73 (tt, *J* = 3.8, 1.7 Hz, 1H), 2.33 (tdd, *J* = 6.1, 2.7, 1.8 Hz, 2H), 2.27 – 2.17 (m, 2H), 1.78 – 1.69 (m, 2H), 1.68 – 1.61 (m, 2H) ppm.

HR-ESI-MS: *m/z*: 258.0654 ($[M+Na]^+$, calcd. for $C_{13}H_{14}NOClNa^+$: 258.0661).

Mp = 146 – 148 °C.

Analytical data in agreement with literature.[5]

_N_-(4-(_tert_-Butyl)phenyl)cyclohex-1-ene-1-carboxamide (1d)

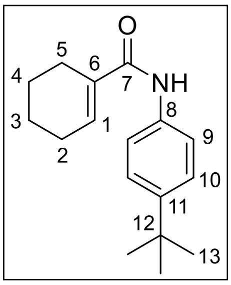

According to General Procedure **A**, cyclohex-1-ene-1-carboxylic acid (631 mg, 5.00 mmol, 1.00 eq.) and 4-(_tert_-butyl)aniline (0.88 mL, 5.5 mmol, 1.1 eq.) were converted to **1d**. Purification by column chromatography (SiO_2, 10% EtOAc in _n_-pentane) yielded the product as a white solid (1.07 g, 4.15 mmol, 83% over 2 steps).

R_f = 0.45 (15% EtOAc in _n_-pentane).

^{1}H NMR (500 MHz, $CDCl_3$): δ = 7.49 – 7.45 (m, 2H, H9), 7.40 (s, 1H, NH), 7.36 – 7.31 (m, 2H, H10), 6.71 (tt, _J_ = 3.9, 1.7 Hz, 1H, H1), 2.38 – 2.31 (m, 2H, H5), 2.24 – 2.18 (m, 2H, H2), 1.76 – 1.68 (m, 2H, H4), 1.67 – 1.60 (m, 2H, H3), 1.30 (s, 9H, H13) ppm.

^{13}C NMR (126 MHz, $CDCl_3$): δ = 166.9 (C7), 147.2 (C11), 135.6 (C8), 134.2 (C6), 134.1 (C1), 125.9 (C10), 119.9 (C9), 34.5 (C12), 31.5 (C13), 25.6 (C2), 24.5 (C5), 22.3 (C4), 21.6 (C3) ppm.

IR (ATR): $\tilde{\nu}$ = 649 (m), 672 (w), 729 (s), 832 (m), 907 (s), 1079 (w), 1113 (w), 1194 (w), 1251 (m), 1268 (m), 1297 (w), 1320 (m), 1366 (w), 1400 (m), 1435 (w), 1515 (s), 1596 (m), 1630 (m), 1659 (m), 2864 (w), 2939 (m), 3312 (w) cm^{-1}.

HR-ESI-MS: _m/z_: 280.16683 ($[M+Na]^+$, calcd. for $C_{17}H_{23}NONa^+$: 280.16719).

Mp = 83 – 84 °C.

_N_-(4-Ethynylphenyl)cyclohex-1-ene-1-carboxamide (1e)

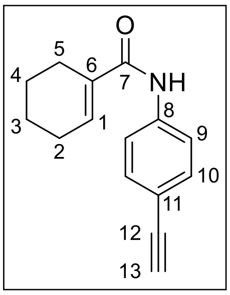

According to General Procedure **B**, cyclohex-1-ene-1-carboxylic acid (631 mg, 5.00 mmol, 1.00 eq.) and 4-ethynylaniline (644 mg, 5.50 mmol, 1.10 eq.) were converted to **1e**. Purification by column chromatography (SiO_2, 7-8% EtOAc in _n_-pentane) and subsequent recrystallization from DCM/_n_-pentane yielded the product as a white solid (561 mg, 2.49 mmol, 50%).

R_f = 0.53 (_n_-pentane/EtOAc 8:2).

^{1}H NMR (500 MHz, $CDCl_3$): δ = 7.57 – 7.50 (m, 2H, H9), 7.48 – 7.38 (m, 3H, H10, NH), 6.74 (tq, _J_ = 3.7, 1.5 Hz, 1H, H1), 3.04 (s, 1H, H13), 2.38 – 2.30 (m, 2H, H5), 2.26 – 2.18 (m, 2H, H2), 1.73 (pd, _J_ = 6.0, 1.6 Hz, 2H, H4), 1.64 (dtd, _J_ = 12.2, 6.3, 3.4 Hz, 2H, H3) ppm.

^{13}C NMR (126 MHz, $CDCl_3$): δ = 167.0 (C7), 139.0 (C8), 135.1 (C6), 134.3 (C1), 133.4 (C10), 119.9 (C9), 117.8 (C11), 83.8 (C12), 77.1 (C13), 25.9 (C2), 24.7 (C5), 22.5 (C4), 21.8 (C3) ppm.

IR (ATR): $\tilde{\nu}$ = 614 (s), 649 (s), 689 (m), 735 (w), 827 (s), 895 (w), 924 (w), 964 (w), 1079 (w), 1108 (w), 1136 (w), 1177 (w), 1234 (m), 1309 (s), 1366 (w), 1400 (m), 1429 (m), 1492 (s), 1510 (s), 1578 (m), 1636 (m), 1659 (m), 2858 (w), 2927 (m), 3289 (m) cm^{-1}.

HR-ESI-MS: *m/z*: 248.10455 ($[M+Na]^+$, calcd. for $C_{15}H_{15}NONa^+$: 248.10459).

Mp = 118 – 119 °C.

4-(Trimethylsilyl)aniline (S21)

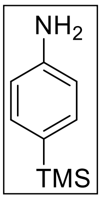

According to a procedure by Buchwald and co-workers,[6] a dried pressure tube was charged with NaO*t*Bu (404 mg, 4.20 mmol, 1.40 eq.) and evacuated and back-filled with argon three times. Then, 1,4-dioxane (20 mL, 0.15 M) was added, followed by the addition of (4-bromophenyl)trimethylsilane (0.57 mL, 3.0 mmol, 1.0 eq.) and ammonia (0.5 M in dioxane, 18 mL, 9.0 mmol, 3.0 eq.). Simultaneously, $Pd(dba)_2$ (69 mg, 0.12 mmol, 4.0 mol%) and $Me_4{}^tBu$-XPhos (87 mg, 0.18 mmol, 6.0 mol%) were added to another dried Schlenk flask and the flask was evacuated and back-filled with argon three times. 1,4-Dioxane (4 mL) was added to the mixture and the solution was heated to 100 °C and stirred at this temperature for 5 min (color change from purple to brown). After cooling to ambient temperature, the brown solution was added to the pressure tube. The reaction mixture was heated to 80 °C and stirred for 2 h at this temperature. The reaction mixture was cooled to r.t. and EtOAc was added to the solution. The solution was filtered over Celite© and the solvent was removed *in vacuo*. The crude residue was purified by column chromatography (10% EtOAc in *n*-pentane) yielding the desired product as a yellow oil (321 mg, 1.94 mmol, 65%).

R_f = 0.43 (15% EtOAc in *n*-pentane).

^{1}H NMR (400 MHz, $CDCl_3$): δ = 7.35 – 7.28 (m, 2H), 6.73 – 6.65 (m, 2H), 3.69 (s, 2H), 0.22 (s, 9H) ppm.

Analytical data in agreement with literature.[6]

N-(4-(Trimethylsilyl)phenyl)cyclohex-1-ene-1-carboxamide (1f)

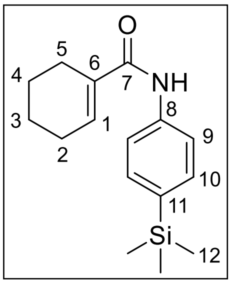

According to General Procedure **B**, cyclohex-1-ene-1-carboxylic acid (309 mg, 2.45 mmol, 1.00 eq.) and 4-(trimethylsilyl)aniline (**S21**) (446 mg, 2.70 mmol, 1.10 eq.) were converted to **1f**. Purification by column chromatography (SiO_2, 8% EtOAc in *n*-pentane) yielded the product as a white solid (463 mg, 1.69 mmol, 69%).

R_f = 0.74 (*n*-pentane/EtOAc 8:2).

^{1}H NMR (500 MHz, $CDCl_3$): δ = 7.56 – 7.51 (m, 2H, H9), 7.50 – 7.45 (m, 2H, H10), 7.37 (s, 1H, NH), 6.73 (tt, *J* = 3.9, 1.7 Hz, 1H, H1), 2.39 – 2.32 (m, 2H, H5), 2.22 (dddd, *J* = 8.8, 6.3, 3.9, 2.6 Hz, 2H, H2), 1.77 – 1.70 (m, 2H, H4), 1.68 – 1.59 (m, 2H, H3), 0.25 (s, 9H, H12) ppm.

^{13}C NMR (126 MHz, $CDCl_3$): δ = 166.8 (C7), 138.7 (C8), 136.0 (C11), 134.4 (C6), 134.3 (C1), 134.2 (C10), 119.3 (C9), 25.7 (C2), 24.5 (C5), 22.3 (C4), 21.6 (C3), –0.9 (C12) ppm.

^{29}Si NMR (99 MHz, $CDCl_3$): δ = –4.38 ppm.

IR (ATR): $\tilde{\nu}$ = 660 (m), 695 (w), 752 (m), 838 (s), 924 (w), 1113 (m), 1188 (w), 1246 (m), 1280 (m), 1320 (m), 1349 (w), 1389 (m), 1441 (w), 1510 (s), 1584 (s), 1636 (m), 1659 (m), 2864 (w), 2939 (m), 3025 (w), 3300 (w) cm^{-1}.

HR-ESI-MS: *m/z*: 272.14736 ([*M*-H]$^-$, calcd. for $C_{16}H_{22}NOSi^-$: 272.14761).

Mp = 92 –93 °C.

4-(Triphenylsilyl)aniline (S22)

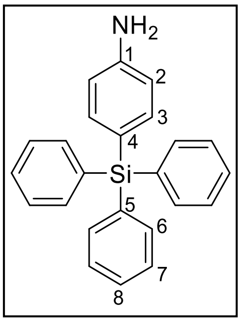

According to a procedure by Buchwald and co-workers,[6] a dried pressure tube was charged with (4-bromophenyl)triphenylsilane (1.66 g, 4.00 mmol, 1.00 eq.) and NaO*t*Bu (577 mg, 6.00 mmol, 1.50 eq.) and evacuated and back-filled with argon three times. Then, 1,4-dioxane (28 mL, 0.125 M) was added, followed by the addition of ammonia (0.5 M in dioxane, 24 mL, 10 mmol, 3.0 eq.). Simultaneously, $Pd(dba)_2$ (92 mg, 0.16 mmol, 4.0 mol%) and $Me_4{}^t$Bu-XPhos (115 mg, 0.24 mmol, 6.0 mol%) were added to another dried Schlenk flask and the flask was evacuated and back-filled with argon three times. 1,4-Dioxane (4 mL) was added to the mixture and the solution was heated to 100 °C and stirred at this temperature for 5 min (color change from purple to brown). After cooling to ambient temperature, the brown solution was added to the pressure tube. The reaction

mixture was heated to 80 °C and stirred for 6 h at this temperature. Then, Pd(dba)$_2$ (41 mg, 0.08 mmol, 2 mol%) and Me$_4{}^t$Bu-XPhos (68 mg, 0.12 mmol, 3 mol%) were added to the reaction mixture and the reaction was stirred for further 15 h at 80 °C. The reaction mixture was cooled to r.t. and EtOAc was added to the solution. The solution was filtered over Celite© and the solvent was removed *in vacuo*. The crude residue was purified by column chromatography (10% EtOAc in *n*-pentane) yielding the desired product as a yellow oil (1.05 g, 3.00 mmol, 75%).

R$_f$ = 0.29 (15% EtOAc in *n*-pentane).

^{1}H NMR (400 MHz, CDCl$_3$): δ = 7.60 – 7.53 (m, 6H, H6), 7.45 – 7.39 (m, 3H, H8), 7.38 – 7.33 (m, 8H, H7, H2), 6.75 – 6.68 (m, 2H, H3) ppm.

^{13}C NMR (126 MHz, CDCl$_3$): δ = 147.5 (C1), 137.9 (C2), 136.5 (C6), 135.1 (C5), 129.5 (C8), 127.9 (C7), 122.1 (C4), 114.9 (C3) ppm.

^{29}Si NMR (99 MHz, CDCl$_3$): δ = –14.68 ppm.

IR (ATR): $\tilde{\nu}$ = 637 (m), 700 (s), 740 (m), 821 (m), 907 (w), 999 (w), 1027 (w), 1108 (s), 1188 (m), 1268 (w), 1297 (m), 1423 (m), 1481 (w), 1504 (m), 1596 (m), 1619 (m), 2910 (w), 3013 (w), 3048 (w), 3065 (w), 3386 (w), 3478 (w) cm^{-1}.

HR-ESI-MS: *m/z*: 352.15148 ([*M*+H]$^+$, calcd. for $C_{24}H_{21}NSiH^+$: 352.15160).

Mp = 180 – 183 °C.

***N*-(4-(Triphenylsilyl)phenyl)cyclohex-1-ene-1-carboxamide (1g)**

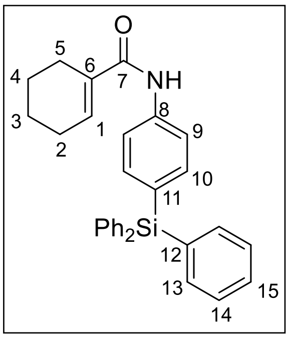

According to General Procedure **A**, cyclohex-1-ene-1-carboxylic acid (315 mg, 2.50 mmol, 1.00 eq.) and 4-(triphenylsilyl)aniline (**S22**) (966 mg, 2.75 mmol, 1.10 eq.) were converted to **1g**. Purification by column chromatography (SiO$_2$, 5-10% EtOAc in *n*-pentane) yielded the product as a white solid (725 mg, 1.58 mmol, 63% over 2 steps).

R$_f$ = 0.34 (15% EtOAc in *n*-pentane).

^{1}H NMR (500 MHz, DMSO-d$_6$): δ = 9.69 (s, 1H, NH), 7.78 – 7.71 (m, 2H, H9), 7.52 – 7.45 (m, 9H, H13, H15), 7.45 – 7.37 (m, 8H, H10, H14), 6.66 (tt, *J* = 3.9, 1.7 Hz, 1H, H1), 2.26 (ddt, *J* = 6.1, 4.5, 2.8 Hz, 2H, H5), 2.21 – 2.13 (m, 2H, H2), 1.66 – 1.60 (m, 2H, H4), 1.56 (pt, *J* = 5.4, 2.5 Hz, 2H, H3) ppm.

^{13}C NMR (126 MHz, DMSO-d$_6$): δ = 167.1 (C7), 140.9 (C8), 136.2 (C10), 135.8 (C13), 134.0 (C6), 133.8 (C12), 133.2 (C1), 129.7 (C15), 128.1 (C14), 127.1 (C11), 119.6 (C9), 24.8 (C2), 24.0 (C5), 21.7 (C4), 21.2 (C3) ppm.

IR (ATR): $\tilde{\nu}$ = 649 (s), 700 (s), 735 (s), 827 (w), 907 (m), 999 (w), 1039 (w), 1108 (s), 1188 (m), 1234 (m), 1280 (m), 1320 (m), 1389 (m), 1429 (m), 1510 (s), 1584 (s), 1636 (m), 1659 (m), 2365 (w), 2864 (w), 2933 (w), 3048 (w), 3065 (w), 3300 (w) cm^{-1}.

HR-ESI-MS: *m/z*: 482.19117 ([*M*+Na]$^+$, calcd. for $C_{31}H_{29}NOSiNa^+$: 482.19106).

Mp = 182 – 184 °C.

N-(4-(4,4,5,5-Tetramethyl-1,3,2-dioxaborolan-2-yl)phenyl)cyclohex-1-ene-1-carboxamide (1h)

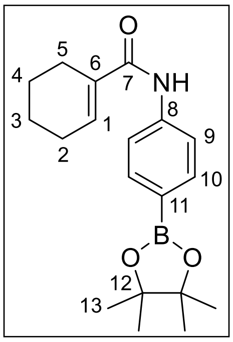

According to General Procedure **A**, cyclohex-1-ene-1-carboxylic acid (631 mg, 5.00 mmol, 1.00 eq.) and 4-(4,4,5,5-tetramethyl-1,3,2-dioxaborolan-2-yl)aniline (1.20 g, 5.50 mmol, 1.10 eq.) were converted to **1h**. Purification by column chromatography (SiO_2, 20% EtOAc in *n*-pentane) yielded the product as a white solid (648 mg, 1.98 mmol, 40% over 2 steps).

R$_f$ = 0.28 (*n*-pentane/EtOAc 8:2).

^{1}H NMR (500 MHz, $CDCl_3$): δ = 7.78 – 7.74 (m, 2H, H10), 7.59 – 7.55 (m, 2H, H9), 7.51 (s, 1H, NH), 6.72 (td, *J* = 3.9, 1.9 Hz, 1H, H1), 2.33 (tdp, *J* = 5.6, 2.6, 1.4 Hz, 2H, H5), 2.20 (ddtt, *J* = 5.2, 3.9, 2.7, 1.7 Hz, 2H, H2), 1.74 – 1.68 (m, 2H, H4), 1.65 – 1.59 (m, 2H, H3), 1.33 (s, 12H, H13) ppm.

^{13}C NMR (151 MHz, $CDCl_3$): δ = 166.8 (C7), 140.9 (C8), 135.9 (C10), 134.5 (C1), 134.2 (C6), 124.5 (C11), 118.8 (C9), 83.8 (C12), 25.6 (C2), 25.0 (C13), 24.4 (C5), 22.2 (C4), 21.6 (C3) ppm.

^{11}B NMR (160 MHz, $CDCl_3$): δ = 30.42 ppm.

IR (ATR): $\tilde{\nu}$ = 654 (s), 672 (m), 700 (m), 735 (w), 792 (w), 827 (m), 855 (m), 895 (w), 924 (w), 959 (w), 1022 (w), 1045 (w), 1085 (m), 1136 (s), 1165 (w), 1246 (m), 1280 (s), 1314 (s), 1355 (s), 1395 (s), 1446 (w), 1504 (m), 1584 (m), 1607 (m), 1630 (m), 1659 (m), 2336 (w), 2359 (w), 2858 (w), 2933 (w), 2979 (w), 3289 (w) cm^{-1}.

HR-ESI-MS: *m/z*: 350.1897 ([*M*+Na]$^+$, calcd. for $C_{19}H_{26}BNONa^+$: 350.1898).

Mp = 222 – 224 °C.

N-Phenylcyclopent-1-ene-1-carboxamide (1i)

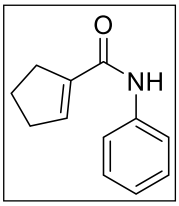

According to General Procedure **A**, cyclopent-1-ene-1-carboxylic acid (561 mg, 5.00 mmol, 1.00 eq.) and aniline (0.50 mL, 5.5 mmol, 1.1 eq.) were converted to **1i**. Purification by column chromatography (SiO_2, 20% EtOAc in *n*-pentane) yielded the product as a white solid (242 mg, 1.29 mmol, 26% over 2 steps).

R_f = 0.22 (10% EtOAc in *n*-pentane).

^{1}H NMR (400 MHz, $CDCl_3$): δ= 7.60 – 7.50 (m, 2H), 7.40 – 7.29 (m, 3H), 7.16 – 7.06 (m, 1H), 6.70 – 6.62 (m, 1H), 2.68 (dddd, *J* = 8.4, 7.7, 4.5, 2.4 Hz, 2H), 2.55 (ddq, *J* = 7.8, 6.8, 2.6 Hz, 2H), 2.05 (tt, *J* = 7.9, 6.9 Hz, 2H) ppm.

HR-ESI-MS: *m/z*: 210.08878 ($[M+Na]^+$, calcd. for $C_{12}H_{13}NONa^+$: 210.08894).

Mp = 127 – 128 °C.

Analytical data in agreement with literature.[7]

Cyclobut-1-ene-1-carboxylic acid (S23)

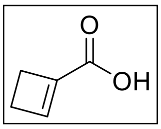

Adapted from a procedure by Deng and co-workers,[8] ethyl 1-bromocyclobutane-1-carboxylate (2.00 mL, 12.4 mmol, 1.00 eq.) was added to a solution of potassium hydroxide (3.03 g, 54.0 mmol, 4.25 eq.) in hot toluene (50 mL). The solution was heated at reflux and stirred for 1 h. After cooling to room temperature, water and diethyl ether were added and the layers were separated. The aqueous layer was washed with EtOAc, subsequently acidified to pH = 1 using aqueous HCl (1 M) and extracted with EtOAc three times. The combined organic extracts were washed with water and saturated aqueous NaCl solution successively, and dried over anhydrous $MgSO_4$. The solvent was removed under reduced pressure and the product was used without further purification.

N-Phenylcyclobut-1-ene-1-carboxamide (1j)

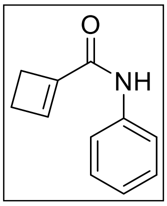

According to General Procedure **A**, crude cyclobut-1-ene-1-carboxylic acid (**S23**) (491 mg, 5.00 mmol, 1.00 eq.) and aniline (0.50 mL, 5.5 mmol, 1.1 eq.) were converted to **1j**. Purification by column chromatography (SiO_2, *n*-pentane/EtOAc: 87:13) yielded the product as white solid (291 mg, 1.68 mmol, 34% over 2 steps).

R_f = 0.29 (*n*-pentane/EtOAc 8:2).

^{1}**H NMR** (400 MHz, $CDCl_3$): δ 7.63 – 7.52 (m, 2H), 7.39 – 7.28 (m, 3H), 7.18 – 7.06 (m, 1H), 6.74 (d, *J* = 1.6 Hz, 1H), 2.85 – 2.75 (m, 2H), 2.51 (td, *J* = 3.3, 1.3 Hz, 2H) ppm.
HR-ESI-MS: *m/z*: 196.0731 ([*M*+Na]$^+$, calcd. for $C_{11}H_{11}NONa^+$: 196.0731).
Mp = 145 – 148 °C.
Analytical data in agreement with literature.[9]

Ethyl 2-(((trifluoromethyl)sulfonyl)oxy)cyclohex-1-ene-1-carboxylate (S24)

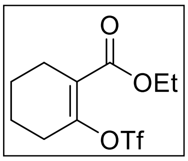

To a stirred suspension of NaH (60% in mineral oil, 0.54 g, 13.5 mmol, 1.80 eq.) in dry toluene (7.5 mL, in total 0.5 M) was added ethyl 2-oxocyclohexane-1-carboxylate (1.2 mL, 7.5 mmol, 1.00 eq.) in dry toluene (7.5 mL). The mixture was stirred at 85 °C for 1.5 h. The reaction mixture was cooled to 0 °C and Tf_2O (1.9 mL, 11.3 mmol, 1.5 eq.) was added dropwise. The mixture was stirred at 0 °C for 2 h. The reaction was quenched by addition of H_2O and the aqueous layer was extracted with EtOAc (3 x 15 mL). The combined organic layers were washed with water (15 mL) and brine (15 mL), dried over anhydrous $MgSO_4$ and the solvent was removed under reduced pressure. The crude residue was purified by column chromatography (2.5% Et_2O in *n*-pentane) yielding a colorless liquid (2.02 g, 6.69 mmol, 89%).

R_f **=** 0.25 (5% Et_2O in *n*-pentane).
^{1}**H NMR** (400 MHz, $CDCl_3$): δ = 4.27 (q, *J* = 7.1 Hz, 2H), 2.48 (ddt, *J* = 8.6, 5.8, 2.6 Hz, 2H), 2.40 (tt, *J* = 6.0, 2.6 Hz, 2H), 1.86 – 1.72 (m, 2H), 1.71 – 1.61 (m, 2H), 1.32 (t, *J* = 7.2 Hz, 3H) ppm.
^{19}**F NMR** (377 MHz, $CDCl_3$): δ = –74.74 ppm.
HR-ESI-MS: *m/z*: 325.03280 ([*M*+Na]$^+$, calcd. for $C_{10}H_{13}O_5SF_3Na^+$: 325.03280).
Analytical data in agreement with literature.[10]

Ethyl 2-methylcyclohex-1-ene-1-carboxylate (S25)

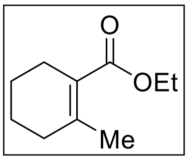

Prepared according to a procedure of Tius *et al.*[11] To a stirred solution of CuCN (840 mg, 9.40 mmol, 1.40 eq.) in dry Et_2O (33 mL, in total 0.17 M) under argon atmosphere, MeLi (1.6 M in Et_2O, 5.9 mL, 9.40 mmol, 1.40 eq.) was added dropwise at –50 °C. The reaction mixture was stirred for 30 min at –50 °C before a solution of **S24** (2.02 g, 6.70 mmol, 1.00 eq.) in dry Et_2O (7 mL) was added. The reaction was stirred at –50 °C for 30 min. The excess of cuprate reagent was quenched by the addition of NH_4Cl (sat.,

aq.). The mixture was filtered over Celite© and the aqueous layer was extracted with EtOAc (3 x 15 mL). The combined organic layers were washed with H_2O (20 mL) and brine (20 mL) and dried over anhydrous $MgSO_4$. The solvent was removed *in vacuo*. The crude residue was purified by column chromatography (1-3% Et_2O in *n*-pentane) yielding the desired product as a colorless oil (678 mg, 4.40 mmol, 66%).

R*f* = 0.35 (5% Et_2O in *n*-pentane).

^{1}H NMR (400 MHz, $CDCl_3$): δ = 4.18 (q, *J* = 7.1 Hz, 2H), 2.26 (dtq, *J* = 5.3, 3.5, 1.8 Hz, 2H), 2.15 – 2.06 (m, 2H), 1.97 (tt, *J* = 1.9, 0.8 Hz, 3H), 1.60 (p, *J* = 3.1 Hz, 4H), 1.29 (t, *J* = 7.1 Hz, 3H) ppm.

HR-ESI-MS: *m/z*: 177.08859 ([*M*+Na]$^+$, calcd. for $C_9H_{14}O_2Na^+$: 177.08860).

Analytical data in agreement with literature.[12]

2-Methyl-*N*-phenylcyclohex-1-ene-1-carboxamide (1k)

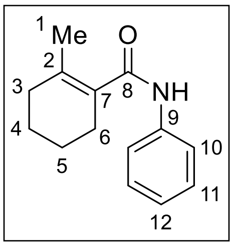

In a flame-dried Schlenk tube under argon atmosphere, methyl cyclohept-1-ene-1-carboxylate (**S25**) (678 mg, 4.40 mmol, 1.00 eq.) and aniline (0.80 mL, 8.80 mmol, 2.00 eq.) were dissolved in dry THF (18 mL, 0.25 M). At 0 °C *i*PrMgCl (2 M in THF, 5.50 mL, 11.0 mmol, 2.50 eq.) was added slowly and the solution was stirred at 0 °C for 2.5 h. Aqueous HCl solution (1 M) and EtOAc were added and the layers were separated. The aqueous layer was extracted with EtOAc (15 mL x 3). The combined organic layers were dried over anhydrous $MgSO_4$ and the solvent was removed under reduced pressure. Purification by column chromatography (SiO_2, 10% EtOAc in *n*-pentane) yielded the product **1k** as a white solid (694 mg, 3.22 mmol, 73%).

R*f* = 0.39 (15% EtOAc in *n*-pentane).

^{1}H NMR (500 MHz, $CDCl_3$): δ = 7.56 (d, *J* = 7.9 Hz, 2H, H10), 7.36 – 7.30 (m, 2H, H11), 7.18 (s, 1H, NH), 7.11 (tt, *J* = 7.4, 1.1 Hz, 1H, H12), 2.31 (s, 2H, H6), 2.05 (t, *J* = 5.5 Hz, 2H, H3), 1.85 (s, 3H, H1), 1.71 – 1.61 (m, 4H, H4, H5) ppm.

^{13}C NMR (126 MHz, $CDCl_3$): δ = 170.1 (C8), 138.1 (C9), 135.8 (C2), 130.0 (C7), 129.2 (C11), 124.3 (C12), 119.9 (C10), 31.6 (C3), 27.0 (C6), 22.5 (C4/C5), 22.4 (C4/C5), 21.3 (C1) ppm.

IR (ATR): $\tilde{\nu}$ = 689 (m), 723 (m), 752 (s), 895 (w), 918 (w), 1091 (w), 1142 (w), 1177 (w), 1257 (m), 1314 (s), 1360 (w), 1435 (s), 1498 (s), 1532 (s), 1596 (s), 1636 (s), 1665 (m), 2824 (w), 2933 (m), 3277 (m) cm^{-1}.

HR-ESI-MS: *m/z*: 238.12003 ($[M+Na]^+$, calcd. for $C_{14}H_{17}NOFNa^+$: 238.12024).

Mp = 127 – 128 °C.

N-(4-Methoxyphenyl)cyclohex-1-ene-1-carboxamide (1l)

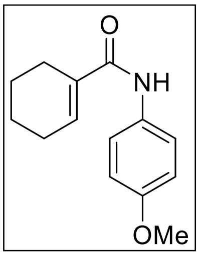

According to General Procedure **A**, cyclohex-1-ene-1-carboxylic acid (631 mg, 5.00 mmol, 1.00 eq.) and 4-methoxyaniline (677 mg, 5.5 mmol, 1.1 eq.) were converted to **1l**. Purification by column chromatography (SiO_2, *n*-pentane/EtOAc: 8:2) and subsequent recrystallization from DCM/*n*-hexane yielded the product as a white solid (813 mg, 3.51 mmol, 70% over 2 steps).

R*f* = 0.47 (*n*-pentane/EtOAc 7:3).

^{1}H NMR (400 MHz, $CDCl_3$): δ 7.47 – 7.41 (m, 2H), 7.38 (s, 1H), 6.89 – 6.81 (m, 2H), 6.71 (tt, *J* = 3.8, 1.7 Hz, 1H), 3.78 (s, 3H), 2.33 (tq, *J* = 6.0, 2.1 Hz, 2H), 2.23 – 2.16 (m, 2H), 1.75 – 1.68 (m, 2H), 1.67 – 1.59 (m, 2H) ppm.

^{13}C NMR (101 MHz, $CDCl_3$): δ = 166.8, 156.4, 134.0 (2C), 131.3, 122.0, 114.2, 55.6, 25.6, 24.5, 22.3, 21.6 ppm.

HR-ESI-MS: *m/z*: 254.11496 ($[M+Na]^+$, calcd. for $C_{14}H_{17}NO_2Na^+$: 254.11515).

Mp = 100 – 102 °C.

Analytical data in agreement with literature.[4]

N-(4-Methoxyphenyl)-*N*-methylcyclohex-1-ene-1-carboxamide (1m)

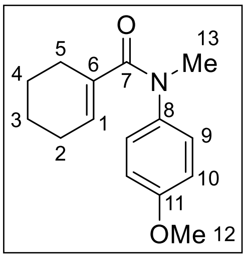

According to General Procedure **C**, *N*-(4-methoxyphenyl)cyclohex-1-ene-1-carboxamide (**1l**) (231 g, 1.00 mmol, 1.00 eq.) and methyl iodide (0.12 mL, 2.0 mmol, 2.0 eq.) were converted to **1m**. Purification by column chromatography (SiO_2, 30% EtOAc in *n*-pentane) yielded the product as a colorless oil (202 mg, 0.82 mmol, 82%).

R*f* = 0.15 (*n*-pentane/EtOAc 8:2).

^{1}H NMR (500 MHz, $CDCl_3$): δ = 7.06 – 7.00 (m, 2H, H10), 6.87 – 6.81 (m, 2H, H9), 5.83 (dp, *J* = 3.4, 1.5 Hz, 1H, H1), 3.81 (s, 3H, H12), 3.29 (s, 3H, H13), 1.90 (tt, *J* = 5.8, 3.5 Hz, 4H, H2, H5), 1.49 – 1.37 (m, 4H, H3, H4) ppm.
^{13}C NMR (126 MHz, $CDCl_3$): δ = 172.9 (C7), 158.1 (C11), 138.0 (C8), 135.0 (C6), 132.2 (C1), 127.8 (C10), 114.3 (C9), 55.6 (C12), 38.0 (C13), 26.2 (C5), 25.1 (C2), 22.2 (C4), 21.6 (C3) ppm.
IR (ATR): $\tilde{\nu}$ = 626 (w), 706 (w), 740 (w), 792 (w), 832 (s), 924 (w), 1033 (s), 1096 (w), 1171 (m), 1240 (s), 1286 (s), 1378 (m), 1435 (m), 1510 (s), 1624 (s), 2835 (w), 2858 (w), 2933 (w) cm^{-1}.

HR-ESI-MS: *m/z*: 268.12758 ($[M+Na]^+$, calcd. for $C_{15}H_{19}NO_2Na^+$: 268.13080).

Methyl 2-oxocycloheptane-1-carboxylate (S26)

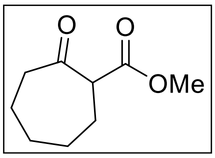

Adapted from a procedure by Booker-Milburn and co-workers,[13] in a flame-dried Schlenk tube under argon atmosphere, cycloheptanone (1.68 g, 15.0 mmol, 1.00 eq.) was added dropwise to a suspension of sodium hydride (60% in mineral oil, 1.20 g, 30.0 mmol, 2.00 eq.) and dimethyl carbonate (2.53 mL, 30.0 mmol, 2.00 eq.) in dry toluene (100 mL) at 90 °C. The reaction mixture was stirred at 90 °C for 16 h. After cooling to room temperature, an aqueous solution of acetic acid (0.4 M, 3 mL) was added slowly, and the mixture was diluted with water and EtOAc. The layers were separated, the aqueous layer was extracted with EtOAc twice and the combined organic layers were washed with saturated aqueous NaCl solution and dried over anhydrous $MgSO_4$. The solvent was removed under reduced pressure. Purification by column chromatography (SiO_2, *n*-pentane/EtOAc: 99:1) yielded the product as a colorless oil (2.22 g, 13.0 mmol, 87%).

R_f = 0.30 (*n*-pentane/EtOAc 19:1).
^{1}H NMR (400 MHz, C_6D_6): δ = 3.36 (s, 3H), 3.31 (dd, *J* = 10.2, 4.1 Hz, 1H), 2.30 – 2.19 (m, 2H), 1.78 – 1.71 (m, 1H), 1.70 – 1.59 (m, 1H), 1.54 – 1.22 (m, 3H), 1.13 – 0.84 (m, 3H) ppm.
HR-ESI-MS: *m/z*: 193.0833 ($[M+Na]^+$, calcd. for $C_9H_{14}O_3Na^+$: 193.0804).
Analytical data in agreement with literature.[14]

Methyl 7-(((trifluoromethyl)sulfonyl)oxy)cyclohept-1-ene-1-carboxylate (S27)

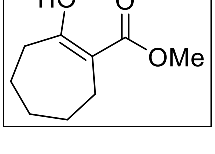

Adapted from a procedure by Booker-Milburn and co-workers,[13] in a flame-dried Schlenk tube under argon atmosphere, a solution of methyl 2-oxocycloheptane-1-carboxylate (**S26**) (2.20 g, 12.9 mmol, 1.00 eq.) in dry

Et_2O (20 mL) was added to a suspension of sodium hydride (60% in mineral oil, 2.58 g, 64.5 mmol, 5.00 eq.) in dry Et_2O (60 mL) at 0 °C. At the same temperature, the mixture was stirred for 30 min, Tf_2O (4.34 mL, 25.8 mmol, 2.00 eq.) was dropwise and the mixture was stirred for 1 h. Water and EtOAc were added and the layers were separated. The aqueous layer was extracted with EtOAc twice and the combined organic layers were washed with saturated aqueous NaCl solution and dried over anhydrous $MgSO_4$. The solvent was removed under reduced pressure. Purification by column chromatography (SiO_2, *n*-pentane/EtOAc: 8:2) yielded the product as a colorless oil (3.39 g, 11.2 mmol, 87%).

R_f = 0.78 (*n*-pentane/EtOAc 3:1).

^{1}H NMR (400 MHz, $CDCl_3$): δ = 3.79 (s, 3H), 2.63 – 2.57 (m, 2H), 2.55 – 2.49 (m, 2H), 1.82 – 1.74 (m, 2H), 1.74 – 1.60 (m, 2H) ppm.

^{19}F NMR (377 MHz, $CDCl_3$): δ = –74.82 ppm.

HR-ESI-MS: *m/z*: 325.0326 ([*M*+Na]$^+$, calcd. for $C10H13O_5F_3SNa^+$: 325.0333).

Analytical data in agreement with literature.[13]

Cyclohept-1-ene-1-carboxylate (S28)

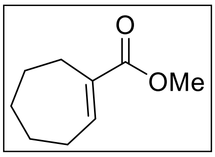

Adapted from a procedure by Zhou and co-workers,[15] under argon atmosphere, a flame-dried Schlenk tube was charged with $Pd(OAc)_2$ (198 mg, 0.88 mmol, 0.10 eq.), 1,1'-bis(diphenylphosphino)ferrocene (533 mg, 0.97 mmol, 0.11 eq.) and dry DMF (13 mL). The mixture was stirred at room temperature for 10 min, and a solution of methyl 7-(((trifluoromethyl)sulfonyl)oxy)cyclohept-1-ene-1-carboxylate (**S27**) (2.65, 8.77 mmol, 1.00 eq.) in dry THF (18 mL) and Et_3SiH (4.22 mL, 26.4 mmol, 3.01 eq.) were added successively under argon atmosphere. The reaction mixture was stirred at room temperature for 2.5 h. Water and EtOAc were added and the layers were separated. The aqueous layer was extracted with EtOAc twice and the combined organic layers were washed with saturated aqueous NaCl solution and dried over anhydrous $MgSO_4$. The solvent was removed under reduced pressure. Purification by column chromatography (SiO_2, *n*-pentane/Et_2O: 98:2) yielded the product as a colorless oil (1.14 g, 7.39 mmol, 84%).

R_f = 0.33 (*n*-pentane/Et_2O 98:2).

^{1}H NMR (400 MHz, $CDCl_3$): δ = 7.17 (tt, *J* = 6.8, 0.7 Hz, 1H), 3.72 (s, 3H), 2.58 – 2.48 (m, 2H), 2.33 – 2.24 (m, 2H), 1.83 – 1.72 (m, 2H), 1.53 (dddt, *J* = 8.5, 7.1, 6.0, 4.7 Hz, 4H) ppm.

ESI-MS: *m/z*: 154.11 ([*M*+Na]$^+$, calcd. for $C_9H_{14}O_2SNa^+$: 154.09).

Analytical data in agreement with literature.[16]

N-Phenylcyclohept-1-ene-1-carboxamide (1n)

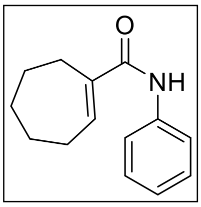

In a flame-dried Schlenk tube under argon atmosphere, methyl cyclohept-1-ene-1-carboxylate (**S28**) (463 mg, 3.00 mmol, 1.00 eq.) and aniline (0.32 mL, 3.45 mmol, 1.15 eq.) were dissolved in dry THF (15 mL). At 0 °C *i*PrMgCl (2 M in THF, 3.75 mL, 7.50 mmol, 2.50 eq.) was added slowly and the solution was stirred at 0 °C for 2.5 h. Aqueous HCl solution (1 M) and EtOAc were added and the layers were separated. The aqueous layer was extracted with EtOAc twice. The combined organic layers were dried over anhydrous $MgSO_4$ and the solvent was removed under reduced pressure. Purification by column chromatography (SiO_2, *n*-pentane/EtOAc: 85:15) yielded the product as a white solid (518 mg, 2.41 mmol, 80%).

R*f* = 0.55 (*n*-pentane/EtOAc 8:2).

^{1}H NMR (400 MHz, $CDCl_3$): δ = 7.54 (ddd, *J* = 8.6, 1.9, 1.0 Hz, 2H), 7.46 (s, 1H), 7.32 (tt, *J* = 7.6, 1.2 Hz, 2H), 7.09 (tq, *J* = 7.1, 1.1 Hz, 1H), 6.68 (t, *J* = 6.5 Hz, 1H), 2.63 – 2.51 (m, 2H), 2.31 (q, *J* = 6.2 Hz, 2H), 1.94 – 1.75 (m, 2H), 1.69 – 1.52 (m, 4H) ppm.

HR-ESI-MS: *m/z*: 238.1200 ([*M*+Na]$^+$, calcd. for $C_{14}H_{17}NONa^+$: 238.1207).

Mp = 123 – 125 °C.

Analytical data in agreement with literature.[4]

N-Methyl-*N*-phenylcyclohept-1-ene-1-carboxamide (1o)

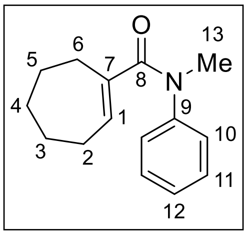

According to General Procedure **C**, *N*-phenylcyclohept-1-ene-1-carboxamide (**1p**) (215 mg, 1.00 mmol, 1.00 eq.) and methyl iodide (0.12 mL, 2.0 mmol, 2.0 eq.) were converted to **1o**. Purification by column chromatography (SiO_2, 20% EtOAc in *n*-pentane) yielded the product as a colorless oil (212 mg, 0.92 mmol, 92%).

R*f* = 0.42 (*n*-pentane/EtOAc 7:3).

^{1}H NMR (600 MHz, $CDCl_3$): δ = 7.35 – 7.31 (m, 2H, H11), 7.22 (ddt, *J* = 8.1, 7.1, 1.3 Hz, 1H, H12), 7.13 – 7.10 (m, 2H, H10), 6.04 (tt, *J* = 6.5, 0.8 Hz, 1H, H1), 3.32 (s, 3H, H13), 2.11 – 2.08 (m, 2H, H6), 2.03 – 1.99 (m, 2H, H2), 1.59 – 1.54 (m, 2H, H4), 1.32 – 1.27 (m, 2H, H3), 1.26 – 1.21 (m, 2H, H5) ppm.

^{13}C NMR (151 MHz, $CDCl_3$): δ = 174.1 (C8), 145.2 (C9), 140.8 (C7), 137.3 (C1), 129.2 (C11), 127.2 (C10), 126.7 (C12), 37.9 (C13), 32.0 (C4), 31.0 (C6), 28.8 (C2), 26.2 (C5), 26.0 (C3) ppm.

IR (ATR): $\tilde{\nu}$ = 672 (m), 700 (s), 735 (s), 769 (s), 821 (w), 861 (m), 895 (w), 976 (w), 1033 (w), 1079 (m), 1113 (m), 1131 (m), 1159 (w), 1217 (w), 1274 (m), 1297 (s), 1349 (s), 1418 (m), 1446 (m), 1492 (s), 1590 (s), 1630 (s), 2847 (m), 2921 (s) cm^{-1}.

HR-ESI-MS: *m/z*: 252.13513 ([*M*+Na]$^+$, calcd. for $C_{15}H_{19}NONa^+$: 252.13589).

N-Methylaniline (S29)

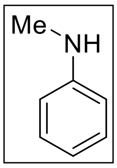

Aniline (1.86 mL, 20.0 mmol, 2.0 equiv.) and K_2CO_3 (2.76 g, 20.0 mmol, 2.0 equiv.) were dissolved in DMF (27 mL) under argon atmosphere and the mixture was stirred for 10 min before iodomethane (0.62 mL, 10.0 mmol, 1.0 equiv.) was added. The reaction mixture was stirred at room temperature for 16 h and a saturated solution of aqueous NH_4Cl was added. The aqueous layer was extracted EtOAc (3 x 40 mL) and the combined organic layers were washed with brine (30 mL), dried over $MgSO_4$, filtered and concentrated under reduced pressure. The crude residue was purified by column chromatography (SiO_2, 1% EtOAc in *n*-pentane) yielding the desired product as a yellow oil (490 mg, 4.60 mmol, 46%).

R_f (1% EtOAc in *n*-pentane) = 0.39.

^{1}H NMR (400 MHz, $CDCl_3$): δ = 7.23–7.17 (m, 2H), 6.70 (tt, *J* = 7.3, 1.1 Hz, 1H), 6.65–6.60 (m, 2H), 3.69 (s, 1H), 2.81 (s, 3H) ppm.

HR-ESI-MS: *m/z*: 108.0807 ([*M*+H]$^+$, calcd. for $C_7H_{10}N^+$: 108.0813).

Analytical data in agreement with literature.[17]

N-Methyl-*N*-phenylcyclopent-1-ene-1-carboxamide (1p)

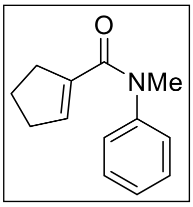

According to General Procedure **B**, cyclopent-1-ene-1-carboxylic acid (540 mg, 4.82 mmol, 1.00 eq.) and *N*-methylaniline (**S29**) (567 mg, 5.30 mmol, 1.10 eq.) were converted to **1p**. Purification by column chromatography (SiO_2, *n*-pentane/EtOAc: 97:3) yielded the product as a colorless oil (778 mg, 3.87 mmol, 80%).

R_f = 0.56 (*n*-pentane/EtOAc 9:1).

^{1}H NMR (400 MHz, $CDCl_3$): δ = 7.36 – 7.30 (m, 2H), 7.27 – 7.22 (m, 1H), 7.15 – 7.11 (m, 2H), 5.82 – 5.79 (m, 1H), 3.33 (s, 3H), 2.24 – 2.13 (m, 4H), 1.73 – 1.63 (m, 2H) ppm.
HR-ESI-MS: *m/z*: 224.10430 ([*M*+Na]$^+$, calcd. for $C_{13}H_{15}NONa^+$: 224.10459).
Analytical data in agreement with literature.[7]

N-Methyl-*N*-phenylcyclohex-1-ene-1-carboxamide (1q)

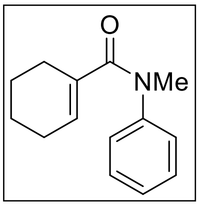

According to General Procedure **A**, cyclohex-1-ene-1-carboxylic acid (379 mg, 3.00 mmol, 1.00 eq.) and *N*-methylaniline (**S29**) (354 mg, 3.30 mmol, 1.10 eq.) were converted to **1q**. Purification by column chromatography (SiO_2, 20% EtOAc in *n*-pentane) yielded the product as a colorless oil (427 mg, 2.00 mmol, 65% after 2 steps).

R_f = 0.26 (20% EtOAc in *n*-pentane).
^{1}H NMR (400 MHz, $CDCl_3$): δ = 7.35 – 7.29 (m, 2H), 7.25 – 7.19 (m, 1H), 7.14 – 7.09 (m, 2H), 5.82 (tt, *J* = 3.8, 1.8 Hz, 1H), 3.34 (s, 3H), 1.95 (tt, *J* = 5.8, 2.2 Hz, 2H), 1.91 – 1.84 (m, 2H), 1.52 – 1.35 (m, 4H) ppm.
HR-ESI-MS: *m/z*: 238.1199 ([*M*+Na]$^+$, calcd. for $C_{14}H_{17}NONa^+$: 238.1207).
Analytical data in agreement with literature.[18]

Cyclohex-1-en-1-yl(3,4-dihydroquinolin-1(2*H*)-yl)methanone (1r)

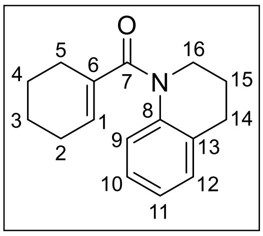

According to General Procedure **A**, cyclohex-1-ene-1-carboxylic acid (379 mg, 3.00 mmol, 1.00 eq.) and 1,2,3,4-tetrahydroquinoline (0.42 mL, 3.3 mmol, 1.1 eq.) were converted to **1r**. Purification by column chromatography (SiO_2, *n*-pentane/EtOAc: 8:2) yielded the product as a yellow oil (0.58 g, 2.4 mmol, 80% over 2 steps).

R_f = 0.29 (*n*-pentane/EtOAc 8:2).
^{1}H NMR (600 MHz, $CDCl_3$): δ = 7.16 (dd, *J* = 8.1, 1.3 Hz, 1H, H9), 7.13 – 7.06 (m, 2H, H12, H10), 7.03 (td, *J* = 7.4, 1.3 Hz, 1H, H11), 5.94 (tt, *J* = 3.8, 1.8 Hz, 1H, H1), 3.82 – 3.76 (m, 2H; H16), 2.74 (t, *J* = 6.6 Hz, 2H, H14), 2.13 – 2.04 (m, 2H, H2), 2.04 – 1.99 (m, 2H, H5), 1.99 – 1.95 (m, 2H, H15), 1.63 – 1.49 (m, 4H, H3, H4) ppm.

^{13}C NMR (151 MHz, $CDCl_3$): δ = 172.3 (C7), 139.7 (C8), 135.4 (C6), 132.6 (C1), 131.5 (C13), 128.4 (C12), 125.9 (C10), 124.6 (C11), 124.3 (C9), 43.9 (C16), 27.1 (C14), 25.7 (C2), 25.2 (C5), 24.3 (C15), 22.2 (C3/C4), 21.7 (C3/4) ppm.

IR (ATR): $\tilde{\nu}$ = 672 (w), 706 (w), 758 (m), 804 (w), 855 (w), 918 (w), 964 (w), 1033 (w), 1056 (w), 1079 (w), 1136 (w), 1177 (w), 1205 (m), 1257 (m), 1297 (m), 1332 (m), 1378 (m), 1452 (w), 1492 (m), 1578 (m), 1630 (s), 2858 (w), 2933 (m), 3031 (w) cm^{-1}.

HR-ESI-MS: *m/z*: 264.1361 ([*M*+Na]$^+$, calcd. for $C_{16}H_{19}NONa^+$: 264.1364).

***N*-(4-Fluorophenyl)-*N*-methylcyclohex-1-ene-1-carboxamide (1s)**

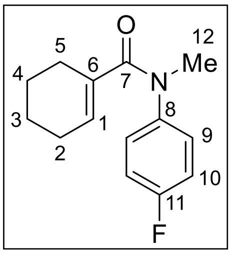

According to General Procedure **C**, *N*-(4-fluorophenyl)cyclohex-1-ene-1-carboxamide (**1b**) (0.33 g, 1.5 mmol, 1.0 eq.) and methyl iodide (0.19 mL, 3.1 mmol, 2.1 eq.) were converted to **1s**. Purification by column chromatography (SiO_2, *n*-pentane/EtOAc: 9:1) yielded the product as a colorless oil (0.31 g, 1.3 mmol, 87%).

R_f = 0.08 (*n*-pentane/EtOAc 9:1).

^{1}H NMR (500 MHz, $CDCl_3$): δ = 7.10 – 7.06 (m, 2H, H9), 7.04 – 6.98 (m, 2H, H10), 5.81 (tt, *J* = 3.8, 1.8 Hz, 1H, H1), 3.30 (s, 3H, H12), 1.97 – 1.87 (m, 4H, H2, H5), 1.49 – 1.38 (m, 4H, H3, H4) ppm.

^{13}C NMR (126 MHz, $CDCl_3$): δ = 172.7 (C7), 161.0 (d, *J* = 246.6 Hz, C11), 141.2 (d, *J* = 3.3 Hz, C8), 134.6 (C6), 132.7 (C1), 128.2 (d, *J* = 8.4 Hz, C9), 116.0 (d, *J* = 22.6 Hz, C10), 37.9 (C12), 26.1 (C5), 25.0 (C2), 22.1 (C4), 21.5 (C3) ppm.

^{19}F NMR (470 MHz, $CDCl_3$): δ = –115.19 (tt, *J* = 8.2, 4.9 Hz) ppm.

IR (ATR): $\tilde{\nu}$ = 626 (m), 706 (w), 723 (m), 740 (m), 804 (m), 838 (s), 924 (w), 1010 (m), 1050 (w), 1096 (m), 1154 (m), 1223 (s), 1303 (m), 1355 (m), 1372 (m), 1429 (m), 1504 (s), 1630 (s), 2858 (w), 2933 (m), 3054 (w) cm^{-1}.

HR-ESI-MS: *m/z*: 256.11048 ([*M*+Na]$^+$, calcd. for $C_{14}H_{16}NOFNa^+$: 256.11081).

N-(4-(Trifluoromethyl)phenyl)cyclohex-1-ene-1-carboxamide (S30)

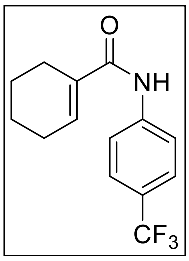

According to General Procedure **A**, cyclohex-1-ene-1-carboxylic acid (379 mg, 3.00 mmol, 1.00 eq.) and 4-(trifluoromethyl)aniline (0.36 mL, 3.3 mmol, 1.1 eq.) were converted to **S30**. Purification by column chromatography (SiO_2, 10% EtOAc in *n*-pentane) yielded the product as a white solid (582 mg, 2.16 mmol, 72% over 2 steps).

R_f = 0.50 (*n*-pentane/EtOAc 8:2).

^{1}H NMR (400 MHz, $CDCl_3$): δ =7.72 – 7.63 (m, 2H), 7.58 (d, *J* = 8.6 Hz, 2H), 7.52 (s, 1H), 6.77 (tt, *J* = 3.8, 1.7 Hz, 1H), 2.40 – 2.32 (m, 2H), 2.24 (tdd, *J* = 6.3, 3.8, 2.0 Hz, 2H), 1.74 (tdd, *J* = 8.4, 5.2, 2.7 Hz, 2H), 1.70 – 1.60 (m, 2H). ppm.

^{19}F NMR (377 MHz, $CDCl_3$): δ = –62.09 ppm.

HR-ESI-MS: *m/z*: 292.09204 ($[M+Na]^+$, calcd. for $C_{14}H_{14}NOF_3Na^+$: 292.09197).

Mp = 127 – 128 °C.

Analytical data in agreement with literature.[4]

N-Methyl-N-(4-(trifluoromethyl)phenyl)cyclohex-1-ene-1-carboxamide (1t)

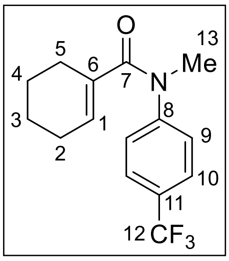

According to General Procedure **C**, *N*-(4-(trifluoromethyl)phenyl)cyclohex-1-ene-1-carboxamide (**S30**) (269 mg, 1.00 mmol, 1.00 eq.) and methyl iodide (0.12 mL, 2.0 mmol, 2.0 eq.) were converted to **1t**. Purification by column chromatography (SiO_2, 20% EtOAc in *n*-pentane) yielded the product as a colorless oil (211 g, 0.74 mmol, 74%).

R_f = 0.31 (*n*-pentane/EtOAc 8:2).

^{1}H NMR (500 MHz, $CDCl_3$): δ = 7.63 – 7.56 (m, 2H, H9), 7.26 – 7.21 (m, 2H, H10), 5.83 (tt, *J* = 3.8, 1.8 Hz, 1H, H1), 3.36 (s, 3H, H13), 2.05 – 2.00 (m, 2H, H5), 1.91 (dddd, *J* = 8.7, 6.2, 4.0, 2.6 Hz, 2H, H2), 1.54 – 1.48 (m, 2H, H4), 1.48 – 1.41 (m, 2H, H3) ppm.

^{13}C NMR (126 MHz, $CDCl_3$): δ = 172.6 (C7), 148.5 (C8), 134.3 (C6), 133.8 (C1), 128.4 (d, *J* = 32.5 Hz, C11), 126.4 (C9), 126.3 (q, *J* = 3.9 Hz, C10), 124.0 (q, *J* = 272.1 Hz, C12), 37.7 (C13), 26.0 (C5), 25.1 (C2), 22.1 (C4), 21.5 (C3) ppm.

^{19}F NMR {^{1}H} (470 MHz, $CDCl_3$): δ = –62.41 ppm.

IR (ATR): $\tilde{\nu}$ = 614 (m), 689 (w), 706 (w), 740 (m), 804 (w), 849 (m), 924 (w), 953 (w), 1010 (m), 1068 (s), 1113 (s), 1159 (s), 1286 (m), 1320 (s), 1378 (m), 1429 (w), 1515 (w), 1607 (s), 1636 (m), 2864 (w), 2933 (w) cm^{-1}.

HR-ESI-MS: *m/z*: 306.10732 ($[M+Na]^+$, calcd. for $C_{15}H_{16}NOF_3Na^+$: 306.10762).

N-(4-Bromophenyl)cyclohex-1-ene-1-carboxamide (S31)

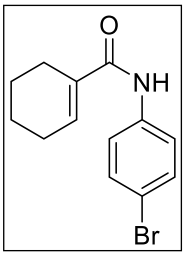

According to General Procedure **A**, cyclohex-1-ene-1-carboxylic acid (631 mg, 5.00 mmol, 1.00 eq.) and 4-bromoaniline (1.54 g, 8.94 mmol, 1.79 eq.) were converted to **S31**. Purification by column chromatography (SiO_2, 10% EtOAc in *n*-pentane) yielded the product as a white solid (799 mg, 3.41 mmol, 68% over 2 steps).

R_f = 0.30 (*n*-pentane/EtOAc 9:1).

^{1}H NMR (400 MHz, $CDCl_3$): δ = 7.49 – 7.40 (m, 4H), 7.39 (s, 1H), 6.73 (tt, *J* = 3.8, 1.7 Hz, 1H), 2.34 (tq, *J* = 6.2, 2.1 Hz, 2H), 2.22 (dtt, *J* = 8.9, 6.1, 2.7 Hz, 2H), 1.73 (tdd, *J* = 8.3, 5.1, 2.6 Hz, 2H), 1.64 (qd, *J* = 6.0, 2.4 Hz, 2H) ppm.

HR-ESI-MS: *m/z*: 302.01473 ($[M+Na]^+$, calcd. for $C_{13}H_{14}NOBrNa^+$: 302.01510).

Mp = 166 – 167 °C.

Analytical data in agreement with literature.[4]

N-(4-Bromophenyl)-*N*-methylcyclohex-1-ene-1-carboxamide (1u)

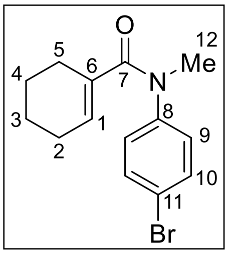

According to General Procedure **C**, *N*-(4-bromophenyl)cyclohex-1-ene-1-carboxamide (**S31**) (280 mg, 1.00 mmol, 1.00 eq.) and methyl iodide (0.12 mL, 2.0 mmol, 2.0 eq.) were converted to **1u**. Purification by column chromatography (SiO_2, 20% EtOAc in *n*-pentane) yielded the product as a colorless oil (260 mg, 0.88 mmol, 88%).

R_f = 0.23 (*n*-pentane/EtOAc 8:2).

^{1}H NMR (500 MHz, $CDCl_3$): δ = 7.47 – 7.43 (m, 2H, H9), 7.02 – 6.98 (m, 2H, H10), 5.83 (tt, *J* = 3.8, 1.8 Hz, 1H, H1), 3.31 (s, 3H, H12), 1.98 (ddt, *J* = 8.3, 5.9, 2.3 Hz, 2H, H5), 1.92 (tdd, *J* = 6.2, 3.8, 2.6 Hz, 2H, H2), 1.52 – 1.46 (m, 2H, H4), 1.46 – 1.40 (m, 2H, H3) ppm.

^{13}C NMR (126 MHz, $CDCl_3$): δ = 172.6 (C7), 144.3 (C8), 134.5 (C6), 133.2 (C1), 132.3 (C9), 128.1 (C10), 120.0 (C11), 37.8 (C12), 26.1 (C5), 25.1 (C2), 22.1 (C4), 21.6 (C3) ppm.

IR (ATR): $\tilde{\nu}$ =700 (w), 717 (m), 746 (w), 832 (s), 924 (w), 1010 (m), 1073 (m), 1096 (m), 1154 (w), 1263 (w), 1291 (m), 1332 (s), 1372 (m), 1429 (m), 1487 (s), 1584 (m), 1630 (s), 2858 (w), 2933 (w) cm^{-1}.

HR-ESI-MS: *m/z*: 316.03012 ($[M+Na]^+$, calcd. for $C_{14}H_{16}NOBrNa^+$: 316.03075).

N-(Phenyl-d_5)cyclohex-1-ene-1-carboxamide (1v)

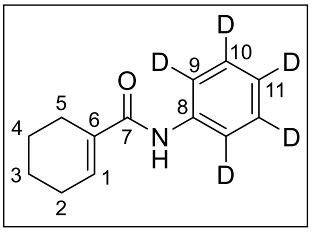

According to General Procedure **A**, cyclohex-1-ene-1-carboxylic acid (946 mg, 7.50 mmol, 1.00 eq.) and benzen-d_5-amine (0.75 mL, 8.3 mmol, 1.1 eq.) were converted to **1v**. Purification by column chromatography (SiO_2, *n*-pentane/EtOAc: 9:1) yielded the product as a white solid (1.35 g, 6.55 mmol, 87% over 2 steps).

R_f = 0.57 (*n*-pentane/EtOAc 7:3).

^{1}H NMR (600 MHz, $CDCl_3$): δ = 7.43 (s, 1H, NH), 6.73 (tt, *J* = 3.9, 1.7 Hz, 1H, H1), 2.35 (ttd, *J* = 6.1, 2.6, 1.7 Hz, 2H, H5), 2.22 (tdt, *J* = 6.4, 4.0, 2.6 Hz, 2H, H2), 1.75 – 1.71 (m, 2H, H4), 1.64 (qd, *J* = 6.1, 2.7 Hz, 2H, H3) ppm.

^{13}C NMR (151 MHz, $CDCl_3$): δ = 166.9 (C7), 138.1 (C8), 134.3 (C1), 134.2 (C6), 128.6 (dt, *J* = 24.5 Hz, C_{Ar}) 123.7 (td, *J* = 24.9 Hz, C_{Ar}), 119.7 (td, *J* = 23.5 Hz, C_{Ar}), 25.6 (C2), 24.5 (C5), 22.3 (C4), 21.6 (C3) ppm.

IR (ATR): $\tilde{\nu}$ = 631 (m), 695 (s), 729 (m), 758 (m), 827 (m), 884 (w), 924 (m), 959 (w), 1050 (w), 1079 (w), 1136 (w), 1182 (w), 1246 (s), 1268 (m), 1303 (s), 1326 (m), 1349 (m), 1383 (s), 1435 (m), 1504 (s), 1567 (m), 1578 (s), 1624 (m), 1653 (m), 2858 (w), 2933 (m), 3300 (w) cm^{-1}.

HR-ESI-MS: *m/z*: 229.13564 ($[M+Na]^+$, calcd. for $C_{13}H_{10}NOD_5Na^+$: 229.13597).

Mp = 119 – 120 °C.

***N*-Methyl-*N*-(phenyl-d_5)cyclohex-1-ene-1-carboxamide (1w)**

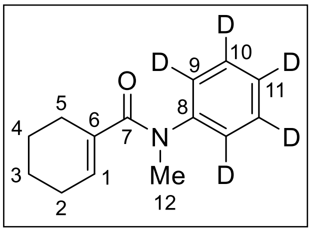

According to General Procedure **C**, **1v** (619 mg, 3.00 mmol, 1.00 eq.) and methyl iodide (0.37 mL, 6.0 mmol, 2.0 eq.) were converted to **1w**. Purification by column chromatography (SiO_2, 10% EtOAc in *n*-

pentane) yielded the product as a colorless oil (614 mg, 2.79 mmol, 93%).

R_f = 0.24 (*n*-pentane/EtOAc 85:15).

^{1}H NMR (600 MHz, $CDCl_3$): δ = 5.82 (tt, *J* = 3.8, 1.8 Hz, 1H, H1), 3.34 (s, 3H, H12), 1.95 (ddt, *J* = 6.0, 4.4, 2.2 Hz, 2H, H5), 1.88 (dtd, *J* = 7.3, 3.8, 2.0 Hz, 2H, H2), 1.47 – 1.43 (m, 2H, H4), 1.42 – 1.37 (m, 2H, H3) ppm.

^{13}C NMR (151 MHz, $CDCl_3$): δ = 173.0 (C7), 145.3 (C8), 135.0 (C6), 132.8 (C1), 128.9 (t, *J* = 24.5 Hz, C_{Ar}), 126.60 – 126.1 (m, C_{Ar}), 126.6 – 126.1 (m, C), 38.0 (C12), 26.3 (C5), 25.3 (C2), 22.4 (C4), 21.8 (C3) ppm.

IR (ATR): $\tilde{\nu}$ = 654 (w), 706 (w), 740 (m), 804 (w), 832 (w), 855 (w), 924 (m), 999 (w), 1079 (m), 1148 (m), 1257 (m), 1314 (s), 1355 (m), 1395 (s), 1561 (m), 1630 (s), 2273 (w), 2858 (w), 2927 (m) cm^{-1}.

HR-ESI-MS: *m/z*: 243.15130 ($[M+Na]^+$, calcd. for $C_{14}H_{12}NOD_5Na^+$: 243.15126).

N-(*p*-Tolyl)cyclohex-1-ene-1-carboxamide (S32)

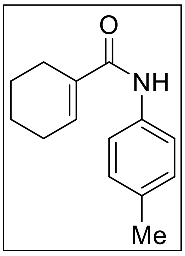

According to General Procedure **A**, cyclohex-1-ene-1-carboxylic acid (631 mg, 5.00 mmol, 1.00 eq.) and *p*-toluidine (589 mg, 5.50 mmol, 1.10 eq.) were converted to **S32**. Purification by column chromatography (SiO_2, 10% EtOAc in *n*-pentane) yielded the product as a white solid (681 mg, 3.16 mmol, 63% over 2 steps).

R_f = 0.49 (*n*-pentane/EtOAc 8:2).

^{1}H NMR (400 MHz, $CDCl_3$): δ = 7.47 – 7.39 (m, 2H), 7.33 (s, 1H), 7.16 – 7.09 (m, 2H), 6.72 (tt, *J* = 3.8, 1.7 Hz, 1H), 2.35 (dddd, *J* = 7.0, 4.4, 3.5, 1.6 Hz, 2H), 2.31 (s, 3H), 2.26 – 2.18 (m, 2H), 1.78 – 1.69 (m, 2H), 1.68 – 1.60 (m, 2H) ppm.

HR-ESI-MS: *m/z*: 238.11999 ($[M+Na]^+$, calcd. for $C_{14}H_{17}NONa^+$: 238.12024).

Mp = 127 – 128 °C.

Analytical data in agreement with literature.[4]

N-(4-(Methylthio)phenyl)cyclohex-1-ene-1-carboxamide (S33)

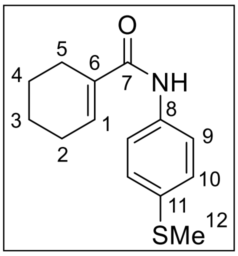

According to General Procedure **A**, cyclohex-1-ene-1-carboxylic acid (631 mg, 5.00 mmol, 1.00 eq.) and 4-(methylthio)aniline (0.68 mL, 5.5 mmol, 1.1 eq.) were converted to **S33**. Purification by column chromatography (SiO_2, 10-15% EtOAc in *n*-pentane) and subsequent recrystallization from DCM/*n*-pentane yielded the product as a white solid (716 mg, 2.89 mmol, 20% over 2 steps).

R_f = 0.30 (15% EtOAc in *n*-pentane).

^{1}H NMR (600 MHz, $CDCl_3$): δ = 7.51 – 7.47 (m, 2H, H9), 7.35 (s, 1H, NH), 7.26 – 7.23 (m, 2H, H10), 6.73 (dq, *J* = 3.8, 1.9 Hz, 1H, H1), 2.47 (s, 3H, H12), 2.37 – 2.31 (m, 2H, H5), 2.24 – 2.19 (m, 2H, H2), 1.77 – 1.70 (m, 2H, H4), 1.68 – 1.60 (m, 2H, H3) ppm.

^{13}C NMR (151 MHz, $CDCl_3$): δ = 166.8 (C7), 135.9 (C8), 134.5 (C1), 134.1 (C6), 133.5 (C11), 128.3 (C10), 120.7 (C9), 25.7 (C2), 24.5 (C5), 22.3 (C4), 21.6 (C3), 16.9 (C12) ppm.

IR (ATR): $\tilde{\nu}$ = 649 (m), 683 (m), 729 (m), 809 (s), 890 (w), 924 (m), 959 (w), 1016 (w), 1045 (w), 1079 (m), 1096 (w), 1182 (w), 1240 (s), 1286 (m), 1309 (s), 1395 (s), 1435 (m), 1498 (s), 1584 (m), 1630 (m), 1659 (m), 2858 (w), 2927 (m), 3318 (w) cm^{-1}.

HR-ESI-MS: *m/z*: 270.09202 ($[M+Na]^+$, calcd. for $C_{14}H_{17}NOSNa^+$: 270.09231).

Mp = 126 – 127 °C.

Methyl 4-(cyclohex-1-ene-1-carboxamido)benzoate (S34)

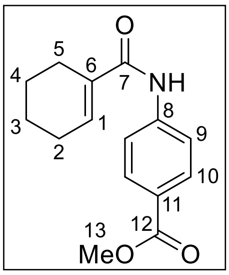

According to General Procedure **A**, cyclohex-1-ene-1-carboxylic acid (631 mg, 5.00 mmol, 1.00 eq.) and methyl 4-aminobenzoate (831 mg, 5.5 mmol, 1.1 eq.) were converted to **S34**. Purification by column chromatography (SiO_2, *n*-pentane/EtOAc: 3:1) yielded the product as a white solid (1.05 mg, 4.05 mmol, 81% over 2 steps).

R_f = 0.39 (*n*-pentane/EtOAc 7:3).

^{1}H NMR (600 MHz, $CDCl_3$): δ 8.00 – 7.97 (m, 2H, H10), 7.66 – 7.63 (m, 3H, H9, NH), 6.75 (tt, *J* = 3.9, 1.8 Hz, 1H, H1), 3.89 (s, 3H, H13), 2.34 (ddt, *J* = 8.4, 6.0, 2.4 Hz, 2H, H5), 2.21 (dddd, *J* = 8.8, 6.3, 4.0, 2.6 Hz, 2H, H2), 1.74 – 1.70 (m, 2H, H4), 1.65 – 1.60 (m, 2H, H3) ppm.

^{13}C NMR (151 MHz, $CDCl_3$): δ = 166.9 (C7), 166.8 (C12), 142.5 (C8), 135.2 (C1), 134.0 (C6), 130.9 (C10), 125.5 (C11), 119.1 (C9), 52.1 (C13), 25.7 (C2), 24.4 (C5), 22.2 (C4), 21.5 (C3) ppm.

IR (ATR): $\tilde{\nu}$ = 643 (w), 695 (m), 735 (w), 769 (m), 798 (w), 855 (m), 890 (w), 924 (w), 964 (w), 1016 (w), 1045 (w), 1079 (m), 1108 (s), 1177 (s), 1246 (s), 1274 (s), 1314 (m), 1372 (w), 1406 (m), 1435 (m), 1515 (s), 1590 (m), 1636 (m), 1665 (m), 1716 (m), 2933 (w), 3329 (w) cm^{-1}.

HR-ESI-MS: *m/z*: 282.10967 ($[M+Na]^+$, calcd. for $C_{15}H_{17}NO_3Na^+$: 282.11006).

Mp = 101 – 102 °C.

***N*-(2-fluorophenyl)cyclohex-1-ene-1-carboxamide (S35)**

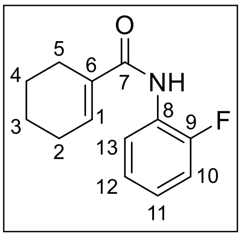

According to General Procedure **A**, cyclohex-1-ene-1-carboxylic acid (631 mg, 5.00 mmol, 1.00 eq.) and 2-fluoroaniline (0.53 mL, 5.5 mmol, 1.1 eq.) were converted to **S35**. Purification by column chromatography (SiO_2, *n*-pentane/EtOAc: 98:2) and subsequent recrystallization from DCM/*n*-pentane yielded the product as a white solid (790 mg, 3.61 mmol, 72% over 2 steps).

R_f = 0.48 (*n*-pentane/EtOAc 9:1).

^{1}H NMR {^{19}F} (500 MHz, $CDCl_3$): δ = 8.38 (dd, *J* = 8.2, 1.6 Hz, 1H, H13), 7.65 (s, 1H, NH), 7.13 (dddd, *J* = 8.1, 7.3, 1.6, 0.5 Hz, 1H, H12), 7.08 (dd, *J* = 8.2, 1.6 Hz, 1H, H10), 7.05 – 7.00 (m, 1H, H11), 6.78 (tt, *J* = 3.9, 1.7 Hz, 1H, H1), 2.36 (dddd, *J* = 6.1, 5.3, 2.6, 1.8 Hz, 2H, H5), 2.26 – 2.21 (m, 2H, H2), 1.77 – 1.71 (m, 2H, H4), 1.68 – 1.62 (m, 2H, H3) ppm.

^{13}C NMR (126 MHz, $CDCl_3$): δ = 166.6 (C7), 152.7 (d, *J* = 242.4 Hz, C9), 135.2 (C1), 133.9 (C6), 126.8 (d, *J* = 9.8 Hz, C8), 124.7 (d, *J* = 3.6 Hz, C12), 124.1 (d, *J* = 7.7 Hz, C11), 121.8 (d, *J* = 1.1 Hz, C13), 114.8 (d, *J* = 19.3 Hz, C10), 25.7 (C2), 24.4 (C5), 22.2 (C4), 21.6 (C3) ppm.

^{19}F NMR {^{1}H} (470 MHz, $CDCl_3$): δ = –131.89 ppm.

IR (ATR): $\tilde{\nu}$ = 660 (m), 700 (m), 746 (s), 804 (w), 832 (w), 867 (w), 890 (w), 930 (m), 964 (w), 1033 (w), 1085 (w), 1108 (m), 1136 (w), 1194 (m), 1234 (m), 1257 (s), 1280 (m), 1314 (s), 1446 (s), 1487 (m), 1521 (s), 1613 (m), 1636 (m), 1659 (m), 2864 (w), 2933 (m), 3312 (w) cm^{-1}.

HR-ESI-MS: *m/z*: 242.09472 ($[M+Na]^+$, calcd. for $C_{13}H_{14}NOFNa^+$: 242.09516).

Mp = 60 – 62 °C.

N-(3,5-Difluorophenyl)cyclohex-1-ene-1-carboxamide (S36)

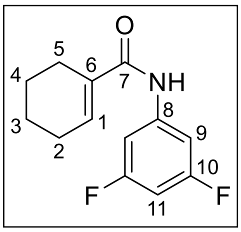

According to General Procedure **A**, cyclohex-1-ene-1-carboxylic acid (631 mg, 5.00 mmol, 1.00 eq.) and 3,5-difluoroaniline (0.50 mL, 5.5 mmol, 1.1 eq.) were converted to **S36**. Purification by column chromatography (SiO_2, *n*-pentane/EtOAc: 9:1) and subsequent recrystallization from DCM/*n*-pentane yielded the product as a white solid (460 mg, 1.94 mmol, 39% over 2 steps).

R_f = 0.66 (*n*-pentane/EtOAc 8:2).

^{1}H NMR (500 MHz, CD_2Cl_2): δ = 7.53 (s, 1H, NH), 7.24 – 7.18 (m, 2H, H9), 6.71 (tt, *J* = 3.9, 1.7 Hz, 1H, H1), 6.56 (tt, *J* = 9.0, 2.3 Hz, 1H, H11), 2.31 (tdd, *J* = 6.0, 2.7, 1.8 Hz, 2H, H5), 2.25 – 2.20 (m, 2H, H2), 1.76 – 1.68 (m, 2H, H4), 1.66 – 1.61 (m, 2H, H3) ppm.

^{13}C NMR {^{19}F} (126 MHz CD_2Cl_2): δ = 167.0 (C7), 163.6 (C10), 141.1 (C8), 135.5 (C1), 134.1 (C6), 103.0 (C9), 99.2 (C11), 25.9 (C2), 24.6 (C5), 22.5 (C4), 21.8 (C3) ppm.

^{19}F NMR {^{1}H} (470 MHz, CD_2Cl_2): δ = –108.02 ppm.

IR (ATR): $\tilde{\nu}$ = 631 (m), 666 (m), 706 (m), 735 (w), 849 (m), 884 (w), 924 (w), 953 (w), 993 (m), 1050 (w), 1108 (m), 1148 (m), 1211 (w), 1251 (m), 1280 (m), 1309 (w), 1349 (w), 1429 (m), 1475 (m), 1550 (m), 1607 (m), 1659 (m), 1969 (w), 2359 (w), 2864 (w), 2933 (w), 3140 (w), 3318 (w) cm^{-1}.

HR-ESI-MS: *m/z*: 260.08567 ([*M*+Na]$^+$, calcd. for $C_{13}H_{13}NOF_2Na^+$: 260.08574).

Mp = 125 – 126 °C.

N-(3,5-Difluorophenyl)cyclohex-1-ene-1-carboxamide (S37)

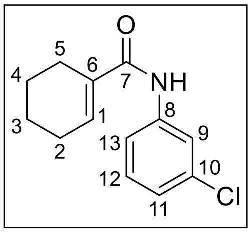

According to General Procedure **A**, cyclohex-1-ene-1-carboxylic acid (631 mg, 5.00 mmol, 1.00 eq.) and 3-chloroaniline (0.58 mL, 5.5 mmol, 1.1 eq.) were converted to **S37**. Purification by column chromatography (SiO_2, *n*-pentane/EtOAc: 9:1) and subsequent recrystallization from DCM/*n*-pentane yielded the product as a white solid (1.09 g, 4.62 mmol, 92% over 2 steps).

R_f = 0.75 (*n*-pentane/EtOAc 7:3).

^{1}H NMR (600 MHz, $CDCl_3$): δ = 7.68 (dd, *J* = 2.1, 2.1 Hz, 1H, H9), 7.45 (s, 1H, NH), 7.39 (ddd, *J* = 8.2, 2.1, 1.0 Hz, 1H, H13), 7.23 (dd, *J* = 8.1, 8.1 Hz, 1H, H12), 7.06 (ddd, *J* = 8.0, 2.0, 0.9 Hz, 1H, H11), 6.73 (tt, *J* = 3.9, 1.7 Hz, 1H, H1), 2.33 (ttd, *J* = 6.2, 2.6, 1.7 Hz, 2H, H5), 2.22 (tdt, *J* = 6.4, 3.9, 2.6 Hz, 2H, H2), 1.75 – 1.70 (m, 2H, H4), 1.66 – 1.61 (m, 2H, H3) ppm.

^{13}**C NMR** (151 MHz, $CDCl_3$): δ = 166.8 (C7), 139.4 (C8), 134.9 (C1), 134.8 (C10), 133.9 (C6), 130.1 (C12), 124.2 (C11), 120.2 (C9), 118.0 (C13), 25.7 (C2), 24.4 (C5), 22.2 (C4), 21.6 (C3) ppm.

IR (ATR): $\tilde{\nu}$ = 677 (s), 775 (s), 804 (m), 884 (m), 924 (m), 999 (w), 1045 (m), 1073 (m), 1136 (w), 1228 (m), 1280 (m), 1309 (m), 1412 (s), 1452 (m), 1475 (m), 1521 (s), 1590 (m), 1630 (m), 1653 (m), 2944 (m), 3318 (w), 3662 (w). cm^{-1}.

HR-ESI-MS: *m/z*: 258.06537 ($[M+Na]^+$, calcd. for $C_{13}H_{14}NOClNa^+$: 258.06561).

Mp = 140 – 142 °C.

***N*-(4-((trifluoromethyl)thio)phenyl)cyclohex-1-ene-1-carboxamide (S38)**

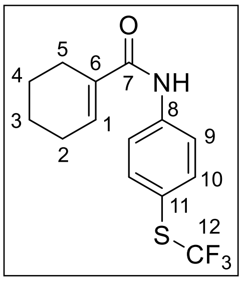

According to General Procedure **B**, cyclohex-1-ene-1-carboxylic acid (505 mg, 4.00 mmol, 1.00 eq.) and 4-((trifluoromethyl)thio)aniline (0.63 mL, 4.4 mmol, 1.1 eq.) were converted to **S38**. Purification by column chromatography (SiO_2, 14% EtOAc in *n*-pentane) yielded the product as a white solid (553 mg, 1.83 mmol, 46%).

$\mathbf{R_f}$ **=** 0.57 (*n*-pentane/EtOAc 8:2).

^{1}**H NMR** (600 MHz, $CDCl_3$): δ = 7.65 – 7.62 (m, 2H, H9), 7.61 – 7.59 (m, 2H, H10), 7.55 – 7.46 (m, 1H, NH), 6.75 (tt, *J* = 3.9, 1.7 Hz, 1H, H1), 2.35 (ttd, *J* = 6.1, 2.6, 1.7 Hz, 2H, H5), 2.23 (tdt, *J* = 6.5, 3.9, 2.6 Hz, 2H, H2), 1.76 – 1.71 (m, 2H, H4), 1.65 (qd, *J* = 6.1, 2.7 Hz, 2H, H3) ppm.

^{13}**C NMR** (151 MHz, $CDCl_3$): δ = 166.9 (C7), 140.9 (C8), 137.6 (C10), 135.3 (C1), 134.0 (C6), 129.7 (q, *J* = 308.3 Hz), C12), 120.5 (C9), 119.0 (C11), 25.7 (C2), 24.5 (C5), 22.2 (C4), 21.5 (C3) ppm.

^{19}**F NMR** (564 MHz, $CDCl_3$): δ = –43.37 ppm.

IR (ATR): $\tilde{\nu}$ = 649 (w), 729 (s), 832 (m), 907 (s), 1016 (w), 1085 (s), 1113 (s), 1154 (m), 1240 (w), 1309 (m), 1349 (w), 1395 (w), 1435 (w), 1510 (m), 1584 (m), 1636 (w), 1665 (m), 2939 (w), 3306 (w) cm^{-1}.

HR-ESI-MS: *m/z*: 324.06363 ($[M+Na]^+$, calcd. for $C_{14}H_{14}NOSF_3Na^+$: 324.06404).

Mp = 118 – 119 °C.

N-(2,4-Dimethylphenyl)cyclohex-1-ene-1-carboxamide (S39)

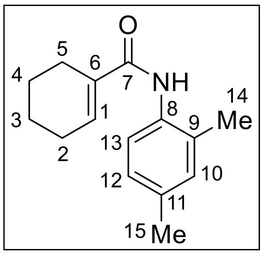

According to General Procedure **A**, cyclohex-1-ene-1-carboxylic acid (631 mg, 5.00 mmol, 1.00 eq.) and 2,4-dimethylaniline (0.68 mL, 5.5 mmol, 1.1 eq.) were converted to **S39**. Purification by column chromatography (SiO_2, 10% EtOAc in *n*-pentane) yielded the product as a white solid (680 mg, 2.97 mmol, 59% over 2 steps).

R_f = 0.56 (*n*-pentane/EtOAc 7:3).

^{1}H NMR (500 MHz, $CDCl_3$): δ = 7.71 (d, *J* = 8.0 Hz, 1H, H13), 7.20 (s, 1H, NH), 7.03 – 6.98 (m, 2H, H10, H12), 6.76 (tt, *J* = 3.9, 1.7 Hz, 1H, H1), 2.36 (tdd, *J* = 6.1, 2.7, 1.8 Hz, 2H, H5), 2.29 (s, 3H, H15), 2.25 – 2.19 (m, 5H, H2, H14), 1.77 – 1.71 (m, 2H, H4), 1.65 (qd, *J* = 6.1, 2.6 Hz, 2H, H3) ppm.

^{13}C NMR (126 MHz, $CDCl_3$): δ =166.7 (C7), 134.7 (C11), 134.2 (C1), 134.0 (C6), 133.4 (C8), 131.2 (C10), 129.2 (C9), 127.4 (C12), 123.2 (C13), 25.6 (C2), 24.6 (C5), 22.3 (C4), 21.6 (C3), 21.0 (C15), 17.8 (C14) ppm.

IR (ATR): $\tilde{\nu}$ = 695 (m), 740 (w), 815 (m), 895 (w), 924 (w), 1050 (m), 1131 (w), 1257 (m), 1274 (w), 1303 (m), 1349 (w), 1378 (w), 1406 (w), 1435 (w), 1504 (s), 1630 (m), 1659 (w), 2933 (m), 2973 (w), 3272 (w), 3656 (w) cm^{-1}.

HR-ESI-MS: *m/z*: 252.13538 ([*M*+Na]$^+$, calcd. for $C_{15}H_{19}NONa^+$: 252.13589).

Mp = 120 – 122 °C.

N,2-Diphenylacrylamide (S40)

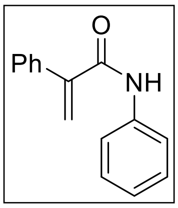

According to General Procedure **A**, 2-phenylacrylic acid (741 mg, 5.00 mmol, 1.00 eq.) and aniline (0.46 mL, 5.0 mmol, 1.0 eq.) were converted to **S40** (NEt_3 (1.5 eq.) was used instead of pyridine). Purification by column chromatography (SiO_2, 5-10% EtOAc in *n*-pentane) yielded the product as a white solid (707 mg, 3.17 mmol, 63% over 2 steps).

R_f = 0.39 (*n*-pentane/EtOAc 8:2).

^{1}H NMR (400 MHz, $CDCl_3$): δ = 7.56 – 7.49 (m, 2H), 7.48 – 7.40 (m, 5H), 7.40 – 7.28 (m, 3H), 7.17 – 7.08 (m, 1H), 6.31 (d, *J* = 1.2 Hz, 1H), 5.74 (d, *J* = 1.3 Hz, 1H) ppm.

HR-ESI-MS: *m/z*: 246.08887 ([*M*+Na]$^+$, calcd. for $C_{15}H_{13}NONa^+$: 246.08894).

Mp = 135 – 136 °C.

Analytical data in agreement with literature.[19]

_N_-Phenylmethacrylamide (S41)

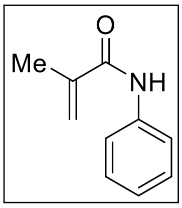

According to General Procedure **A**, methacryloyl chloride (1.07 mL, 11.0 mmol, 1.1 eq.) and aniline (0.91 mL, 10 mmol, 1.0 eq.) were converted to **S41** (NEt_3 (1.0 eq.) was used instead of pyridine). Purification by column chromatography (SiO_2, 5-10% EtOAc in *n*-pentane) yielded the product as a white solid (207 mg, 1.28 mmol, 13%).

R*f* = 0.36 (*n*-pentane/EtOAc 8:2).

^{1}H NMR (400 MHz, $CDCl_3$): δ = 7.60 – 7.50 (m, 2H), 7.47 (s, 1H), 7.39 – 7.29 (m, 2H), 7.19 – 7.08 (m, 1H), 5.79 (t, *J* = 0.9 Hz, 1H), 5.47 (qd, *J* = 1.6, 0.7 Hz, 1H), 2.07 (dd, *J* = 1.6, 0.9 Hz, 3H). ppm.

HR-ESI-MS: *m/z*: 184.07322 ($[M+Na]^+$, calcd. for $C_{10}H_{11}NONa^+$: 184.07329).

Mp = 72 – 74 °C.

Analytical data in agreement with literature.[19]

_N_-(_o_-Tolyl)cyclohex-1-ene-1-carboxamide (S13)

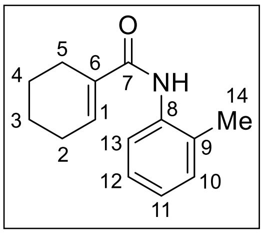

According to General Procedure **A**, cyclohex-1-ene-1-carboxylic acid (631 mg, 5.00 mmol, 1.00 eq.) and *o*-toluidine (0.59 mL, 5.5 mmol, 1.1 eq.) were converted to **S13**. Purification by column chromatography (SiO_2, 10% EtOAc in *n*-pentane) yielded the product as a white solid (362 mg, 1.68 mmol, 34% over 2 steps).

R*f* = 0.29 (*n*-pentane/EtOAc 85:15).

^{1}H NMR (600 MHz, $CDCl_3$): δ = 7.92 (dd, *J* = 8.1, 1.3 Hz, 1H, H13), 7.27 (s, 1H, NH), 7.21 (dddt, *J* = 8.0, 7.4, 1.6, 0.6 Hz, 1H, H12), 7.18 (ddq, *J* = 7.6, 1.3, 0.6 Hz, 1H, H10), 7.06 (td, *J* = 7.5, 1.3 Hz, 1H, H11), 6.77 (tt, *J* = 3.9, 1.7 Hz, 1H, H1), 2.37 (ttd, *J* = 6.1, 2.6, 1.7 Hz, 2H, H5), 2.27 (s, 3H, H14), 2.23 (tdt, *J* = 6.5, 3.9, 2.6 Hz, 2H, H2), 1.77 – 1.73 (m, 2H, H4), 1.68 – 1.63 (m, 2H, H3) ppm.

^{13}C NMR (151 MHz, $CDCl_3$): δ = 166.7 (C7), 136.1 (C8), 134.5 (C1), 134.1 (C6), 130.5 (C10), 128.7 (C9), 127.0 (C12), 124.9 (C11), 122.8 (C13), 25.7 (C2), 24.6 (C5), 22.3 (C4), 21.6 (C3), 17.9 (C14) ppm.

IR (ATR): $\tilde{\nu}$ = 637 (s), 677 (s), 746 (s), 804 (w), 827 (w), 861 (w), 895 (w), 924 (m), 964 (w), 1039 (m), 1073 (w), 1113 (w), 1136 (w), 1194 (w), 1257 (s), 1303 (s), 1343 (w), 1372 (w), 1452 (s), 1515 (s), 1584 (m), 1630 (s), 1659 (s), 2359 (w), 2853 (w), 2933 (m), 3025 (w), 3295 (w) cm^{-1}.
HR-ESI-MS: *m/z*: 214.12356 ($[M\text{-H}]^-$, calcd. for $C_{14}H_{16}NO^-$: 214.12374).
Mp = 91 – 92 °C.

N-(2-Bromophenyl)cyclohex-1-ene-1-carboxamide (S14)

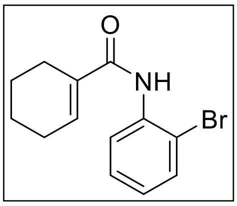

According to General Procedure **A**, cyclohex-1-ene-1-carboxylic acid (631 mg, 5.00 mmol, 1.00 eq.) and 2-bromoaniline (0.62 mL, 5.5 mmol, 1.1 eq.) were converted to **S14**. Purification by column chromatography (SiO_2, 5% EtOAc in *n*-pentane) yielded the product as a white solid (1.16 g, 4.15 mmol, 83% over 2 steps).

R_f = 0.56 (*n*-pentane/EtOAc 9:1).
^{1}H NMR (400 MHz, $CDCl_3$): δ = 8.46 (dd, *J* = 8.3, 1.6 Hz, 1H), 8.03 (s, 1H), 7.53 (dd, *J* = 8.1, 1.5 Hz, 1H), 7.32 (ddd, *J* = 8.6, 7.3, 1.5 Hz, 1H), 6.96 (ddd, *J* = 8.0, 7.4, 1.6 Hz, 1H), 6.87 (tt, *J* = 3.9, 1.7 Hz, 1H), 2.44 – 2.37 (m, 2H), 2.29 – 2.22 (m, 2H), 1.80 – 1.73 (m, 2H), 1.70 – 1.62 (m, 2H) ppm.
HR-ESI-MS: *m/z*: 302.01513, 304.01318 ($[M\text{+Na}]^+$, calcd. for $C_{13}H_{14}NOBrNa^+$: 302.01510, 304.01315).
Mp = 70 – 73 °C.
Analytical data in agreement with literature.[20]

N-(4-(Dimethylamino)phenyl)cyclohex-1-ene-1-carboxamide (S15)

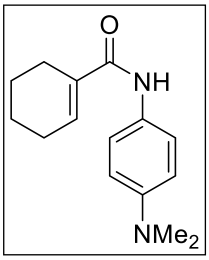

According to General Procedure **B**, cyclohex-1-ene-1-carboxylic acid (631 mg, 5.00 mmol, 1.00 eq.) and N^1,N^1-dimethylbenzene-1,4-diamine (749 mg, 5.50 mmol, 1.10 eq.) were converted to **S15**. Purification by column chromatography (SiO_2, 30-40% EtOAc in *n*-pentane) yielded the product as a white solid (209 mg, 0.86 mmol, 17%).

R_f = 0.56 (*n*-pentane/EtOAc 1:1).

¹H NMR (400 MHz, $CDCl_3$): δ = 7.45 – 7.37 (m, 2H), 7.24 (s, 1H), 6.83 – 6.66 (m, 3H), 2.93 (s, 6H), 2.39 – 2.30 (m, 2H), 2.21 (qt, *J* = 8.1, 4.2 Hz, 2H), 1.73 (tt, *J* = 6.1, 3.5 Hz, 2H), 1.64 (qd, *J* = 6.1, 2.5 Hz, 2H) ppm.

HR-ESI-MS: *m/z*: 267.14633 ($[M+Na]^+$, calcd. for $C_{15}H_{20}N_2ONa^+$: 267.14678).

Mp = 188 – 189 °C.

Analytical data in agreement with literature.[4]

N-(Naphthalen-1-yl)cyclohex-1-ene-1-carboxamide (S16)

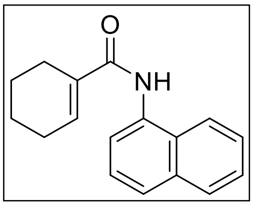

According to General Procedure **A**, cyclohex-1-ene-1-carboxylic acid (631 mg, 5.00 mmol, 1.00 eq.) and naphthalen-1-amine (788 mg, 5.50 mmol, 1.10 eq.) were converted to **S16**. Purification by column chromatography (SiO_2, 3-10% EtOAc in *n*-pentane) yielded the product as a light red solid (970 mg, 3.86 mmol, 77% over two steps).

R_f = 0.17 (*n*-pentane/EtOAc 9:1).

¹H NMR (400 MHz, $CDCl_3$): δ = 8.05 – 8.01 (m, 1H), 7.90 – 7.79 (m, 3H), 7.70 (dt, *J* = 8.3, 1.1 Hz, 1H), 7.56 – 7.47 (m, 3H), 6.90 (tt, *J* = 3.9, 1.8 Hz, 1H), 2.49 (tq, *J* = 5.3, 1.9 Hz, 2H), 2.32 – 2.26 (m, 2H), 1.84 – 1.77 (m, 2H), 1.74 – 1.67 (m, 2H) ppm.

HR-ESI-MS: *m/z*: 274.11981 ($[M+Na]^+$, calcd. for $C_{17}H_{17}NONa^+$: 274.12024).

Mp = 139 – 140 °C.

Analytical data in agreement with literature.[4]

N-(Quinolin-8-yl)cyclohex-1-ene-1-carboxamide (S17)

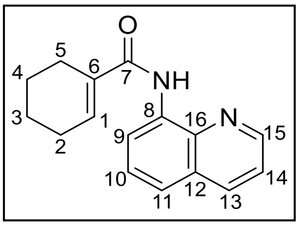

According to General Procedure **A**, cyclohex-1-ene-1-carboxylic acid (631 mg, 5.00 mmol, 1.00 eq.) and quinolin-8-amine (793 mg, 5.50 mmol, 1.10 eq.) were converted to **S17**. Purification by column chromatography (SiO_2, 10% EtOAc in *n*-pentane) yielded the product as a light-yellow solid (1.07 g, 4.24 mmol, 85% over 2 steps).

R_f = 0.40 (*n*-pentane/Et_2O 95:5).

¹H NMR (600 MHz, $CDCl_3$): δ = 10.24 (s, 1H, NH), 8.84 (dd, *J* = 7.6, 1.3 Hz, 1H, H9), 8.81 (dd, *J* = 4.2, 1.7 Hz, 1H, H15), 8.15 (dd, *J* = 8.2, 1.7 Hz, 1H, H13), 7.54 (t, *J* = 7.9 Hz, 1H, H11), 7.49

(dd, J = 8.2, 1.3 Hz, 1H, H10), 7.45 (dd, J = 8.2, 4.2 Hz, 1H, H14), 6.95 (tt, J = 3.9, 1.7 Hz, 1H, H1), 2.52 (ddt, J = 8.6, 6.1, 2.5 Hz, 2H, H5), 2.29 (dddd, J = 8.9, 6.3, 4.0, 2.6 Hz, 2H, H2), 1.84 – 1.76 (m, 2H, H4), 1.71 – 1.66 (m, 2H, H3) ppm.

^{13}C NMR (151 MHz, $CDCl_3$): δ = 166.9 (C7), 148.3 (C15), 138.9 (C16), 136.5 (C13), 134.9 (C1, C8), 134.3 (C6), 128.1 (C12), 127.7 (C11), 121.7 (C14), 121.3 (C10), 116.6 (C9), 25.8 (C2), 24.5 (C5), 22.4 (C4), 21.7 (C3) ppm.

IR (ATR): $\tilde{\nu}$ = 666 (s), 758 (m), 792 (s), 827 (s), 884 (m), 930 (m), 959 (w), 987 (w), 1079 (m), 1108 (w), 1142 (w), 1171 (w), 1223 (m), 1246 (m), 1326 (s), 1383 (s), 1423 (s), 1481 (s), 1521 (s), 1596 (w), 1630 (m), 1665 (s), 2864 (w), 2933 (m), 3352 (m) cm^{-1}.

HR-ESI-MS: *m/z*: 275.11524 ([*M*+Na]$^+$, calcd. for $C_{16}H_{16}N_2ONa^+$: 275.11548).

Mp = 59 – 60 °C.

Phenyl cyclohex-1-ene-1-carboxylate (S18)

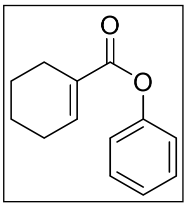

According to General Procedure **B**, cyclohex-1-ene-1-carboxylic acid (505 mg, 4.00 mmol, 1.00 eq.) and phenol (414 mg, 4.40 mmol, 1.10 eq.) were converted to **S18**. Purification by column chromatography (SiO_2, 13% Et_2O in *n*-pentane) yielded the product as a white solid (626 mg, 3.10 mmol, 78%).

R$_f$ = 0.75 (*n*-pentane/EtOAc 9:1).

^{1}H NMR (400 MHz, $CDCl_3$): δ = 7.41 – 7.33 (m, 2H), 7.26 – 7.17 (m, 2H), 7.14 – 7.05 (m, 2H), 2.39 (tq, J = 6.0, 2.2 Hz, 2H), 2.29 (tdd, J = 6.2, 4.0, 2.7 Hz, 2H), 1.78 – 1.61 (m, 4H) ppm.

HR-ESI-MS: *m/z*: 225.08843 ([*M*+Na]$^+$, calcd. for $C_{13}H_{14}O_2Na^+$: 225.08860).

Mp = 74 – 75 °C.

Analytical data in agreement with literature.[21]

(*E*)-*N*-Phenylbut-2-enamide (S19)

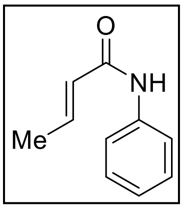

According to General Procedure **B**, (*E*)-but-2-enoic acid (861 mg, 10 mmol. 1.00 eq.) and aniline (1.0 mL, 11 mmol, 1.10 eq.) were converted to **S19**. Purification by column chromatography (SiO_2, DCM) and recrystallization (DCM/*n*-pentane) yielded the product as a white solid (371 mg, 2.3 mmol, 23%).

R$_f$ = 0.25 (*n*-pentane/EtOAc 4:1).

^{1}**H NMR** (400 MHz, $CDCl_3$): δ = 7.56 (d, *J* = 7.9 Hz, 2H), 7.33 (t, *J* = 8.0 Hz, 2H), 7.15 (s, 1H), 7.11 (t, *J* = 7.5 Hz, 1H), 7.00 (dq, *J* = 15.7, 6.9 Hz, 1H), 5.95 (dd, *J* = 15.0, 1.8 Hz, 1H), 1.92 (dd, *J* = 6.9, 1.9 Hz, 3H).

HR-ESI-MS: *m/z*: 184.07311 ($[M+Na]^+$, calcd. for $C_{10}H_{11}NONa^+$: 184.07329).

Mp = 117 – 118 °C.

Analytical data in agreement with literature.[22]

_N_-Phenylcinnamamide (S20)

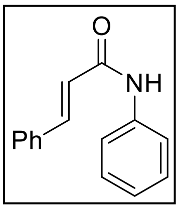

According to General Procedure **B**, cinnamic acid (1.48 g, 10 mmol, 1.00 eq.) and aniline (1.0 mL, 11 mmol, 1.10 eq.) were converted to **S20**. Purification by recrystallization (DCM/*n*-pentane) yielded the product as a white solid (1.77 g, 7.6 mmol, 76%).

$\mathbf{R_f}$ **=** 0.31 (*n*-pentane/EtOAc 4:1).

^{1}**H NMR** (400 MHz, $CDCl_3$): δ = 7.76 (d, *J* = 15.5 Hz, 1H), 7.64 (d, *J* = 7.8 Hz, 2H), 7.54 (dd, *J* = 6.6, 3.0 Hz, 2H), 7.45 (s, 1H), 7.44 – 7.30 (m, 5H), 7.14 (t, *J* = 7.5 Hz, 1H), 6.58 (d, *J* = 15.5 Hz, 1H).

HR-ESI-MS: *m/z*: 246.08885 ($[M+Na]^+$, calcd. for $C_{15}H_{13}NONa^+$: 246.08894).

Mp = 145 – 146 °C.

Analytical data in agreement with literature.[23]

Preparation of Catalysts

*Catalyst **Al-1** was purchased at BLD Pharm, dissolved in DCM, filtered and recrystallized from DCM/n-hexane. Catalysts **Al-5, Al-6** and **Al-7** were prepared according to previously reported procedures.*[2]

(*R,R*)-(−)-Bis(5-*tert*-butylsalicylidene)-1,2-cyclohexanediamine (S42)

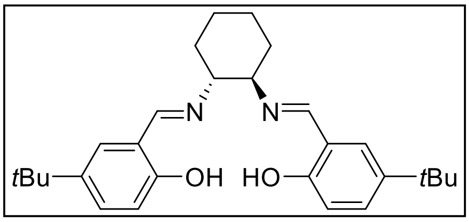

In a pressure tube, 2-(*tert*-butyl)-6-hydroxybenzaldehyde (356 mg, 2.00 mmol, 2.00 eq.), (1*R*,2*R*)-(+)-1,2-diaminocyclohexane *L*-tartrate (264 mg, 1.00 mmol, 1.00 eq.) and K_2CO_3 (276 mg, 2.00 mmol, 2.00 eq.) were suspended in EtOH/H_2O (6:1 v/v, 10 mL, 0.1 M). The reaction mixture was heated at 80 °C and stirred for 2 h. Then, it was cooled to room temperature and diluted with water, the layers were separated and the aqueous layer was extracted with DCM (3x). The combined organic layers were dried over $MgSO_4$ and concentrated *in vacuo*. The desired product was received as a yellow solid and used for the next step without further purification.

Chloro-(*R,R*)-(−)-bis(5-*tert*-butylsalicylidene)-1,2-cyclohexanediamino-aluminium (Al-2)

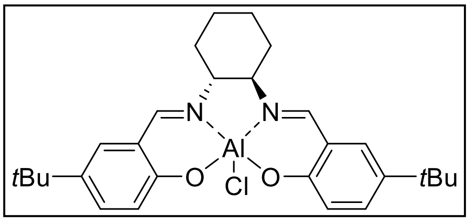

Under argon atmosphere, (*R*,*R*)-(−)-*N*,*N*′-bis(5-*tert*-butylsalicylidene)-1,2-cyclohexanediamine **S42** (217 g, 0.50 mmol, 1.00 eq.) was dissolved in dry DCM (5.0 mL, 0.1 M). Diethylaluminium chloride (1 M in *n*-hexane, 0.53 mL, 0.53 mmol, 1.05 eq.) was added dropwise and the resulting mixture was stirred for 2 h at room temperature, before being concentrated under reduced pressure. The obtained yellow solid was repetitively washed with *n*-hexane. The desired product was furnished as a yellow solid (254 mg, 0.51 mmol, quant.).

^{1}H NMR (300 MHz, CD_2Cl_2): δ = 8.35 (s, 2H), 7.55 (dd, *J* = 8.8, 2.6 Hz, 2H), 7.32 (d, *J* = 2.6 Hz, 2H), 6.97 (d, *J* = 8.8 Hz, 2H), 3.56 (br, 2H), 2.53 (s, 2H), 2.09 (d, *J* = 6.8 Hz, 2H), 1.50 (d, *J* = 7.7 Hz, 4H), 1.32 (s, 18H).

HR-ESI-MS: *m/z*: 459.25843 ([*M*-Cl]$^+$, calcd. for $C_{28}H_{36}N_2O_2Al^+$: 459.25867).

(*R,R*)-(−)-Bis(3-*tert*-butylsalicylidene)-1,2-cyclohexanediamine (S43)

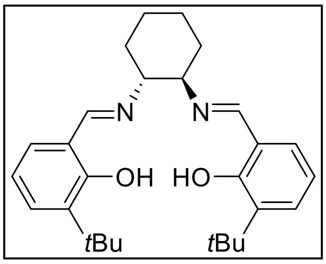

In a pressure tube, 4-(*tert*-butyl)-2-hydroxybenzaldehyde (356 mg, 2.00 mmol, 2.00 eq.), (1*R*,2*R*)-(+)-1,2-diaminocyclohexane *L*-tartarte (264 mg, 1.00 mmol, 1.00 eq.) and K_2CO_3 (276 mg, 2.00 mmol, 2.00 eq.) were suspended in EtOH/H_2O (6:1 v/v, 10 mL, 0.1 M). The reaction mixture was heated at 80 °C and stirred for 2 h. Then, it was cooled down to room temperature and diluted with water, the layers were separated and the aqueous layer was extracted with DCM (3x). The combined organic layers were dried over $MgSO_4$ and concentrated *in vacuo*. The desired product was received as a yellow solid and used for the next step without further purification.

Chloro-(*R,R*)-(−)-bis(3-*tert*-butylsalicylidene)-1,2-cyclohexanediamino-aluminium (Al-3)

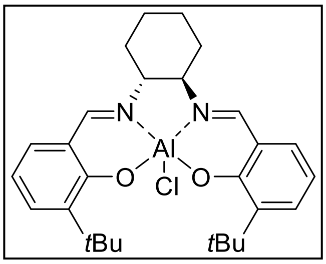

Under argon atmosphere (*R*,*R*)-(−)-*N*,*N′*-bis(5-*tert*-butylsalicylidene)-1,2-cyclohexanediamine **S43** (210 g, 0.50 mmol, 1.00 eq.) was dissolved in dry DCM (5.0 mL, 0.1 M). Diethylaluminium chloride (1 M in *n*-hexane, 0.53 mL, 0.53 mmol, 1.05 eq.) was added dropwise and the resulting mixture was stirred for 2 h at room temperature, before being concentrated under reduced pressure. The obtained yellow solid was repetitively washed with *n*-hexane. The desired product was furnished as a yellow solid (250 mg, 0.50 mmol, quant.).

^{1}H NMR (400 MHz, CD_2Cl_2): δ = 8.30 (s, 2H), 7.49 (dd, *J* = 7.6, 1.8 Hz, 2H), 7.23 (dd, *J* = 7.7, 1.8 Hz, 2H), 6.79 (t, *J* = 7.6 Hz, 2H), 4.00 – 2.98 (br, 2H), 2.48 (s, 2H), 2.05 (s, 2H), 1.51 (s, 22H) ppm.

HR-ESI-MS: *m/z*: 459.25826 ([*M*-Cl]$^+$, calcd. for $C_{28}H_{36}N_2O_2Al^+$: 459.25867).

(*R,R*)-(−)-Bis(3-phenylsalicylidene)-1,2-cyclohexanediamine (S44)

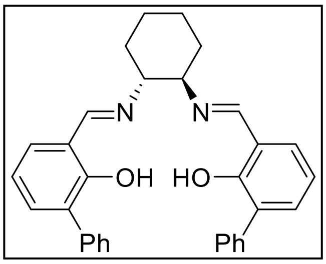

In a pressure tube, 4-phenyl-2-hydroxybenzaldehyde (300 mg, 2.00 mmol, 2.00 eq.), (1*R*,2*R*)-(+)-1,2-diaminocyclohexane *L*-tartarte (264 mg, 1.00 mmol, 1.00 eq.) and K_2CO_3 (276 mg, 2.00 mmol, 2.00 eq.) were suspended in EtOH/H_2O (6:1 v/v, 10 mL, 0.1 M). The reaction mixture was heated at 80 °C and stirred for 2 h.

Then, it was cooled down to room temperature and diluted with water and DCM, the layers were separated and the aqueous layer was extracted with DCM (3x). The combined organic layers were dried over $MgSO_4$ and concentrated *in vacuo*. The desired product was received as a yellow solid and used for the next step without further purification.

Chloro-(*R,R*)-(−)-bis(3-phenylsalicylidene)-1,2-cyclohexanediamino-aluminium (Al-4)

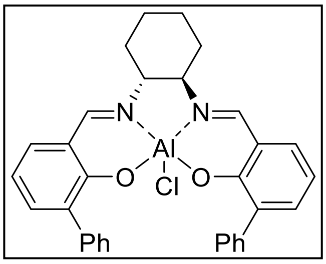

Under argon atmosphere (*R*,*R*)-(−)-*N*,*N*′-bis(3-phenylsalicylidene)-1,2-cyclohexanediamine **S42** (237 g, 0.50 mmol, 1.00 eq.) was dissolved in dry DCM (5.0 mL, 0.1 M). Diethylaluminium chloride (1 M in *n*-hexane, 0.50 mL, 0.50 mmol, 1.00 eq.) was added dropwise and the resulting mixture was stirred for 2 h at room temperature, before being concentrated under reduced pressure. The obtained yellow solid was repetitively washed with *n*-hexane. The desired product was furnished as a yellow solid (535 mg, 0.50 mmol, quant.).

^{1}H NMR (400 MHz, CD_2Cl_2): δ = 8.07 (s, 2H), 7.96 – 6.53 (m, 16H), 3.63 (br, 2H), 2.42 (s, 2H), 2.02 (s, 2H), 1.48 – 1.07 (m, 4H) ppm.

HR-ESI-MS: *m/z*: 499.19517 ([*M*-Cl]$^+$, calcd. for $C_{32}H_{28}N_2O_2^+$: 499.19607).

Methyl-(*R,R*)-(−)-*N,N′*-bis(3,5-di-*tert*-butylsalicylidene)-1,2-cyclohexanediamino-aluminium (Al-8)

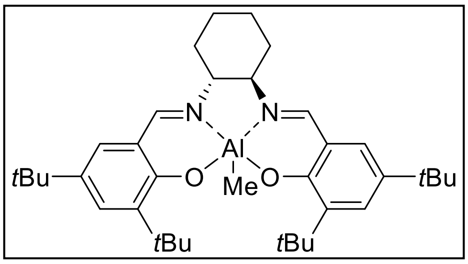

In a flame-dried pressure tube under argon (*R*,*R*)-(−)-*N*,*N*′-bis(3,5-di-*tert*-butylsalicylidene)-1,2-cyclohexane-diamine (1.09 g, 2.00 mmol, 1.00 eq.) was suspended in dry MeCN/toluene (3:1, 8 mL). Trimethylaluminium (2 M in *n*-hexane, 1.0 mL, 2.0 mmol, 1.0 eq.) was added dropwise and the resulting mixture was heated at 110 °C and stirred for 2 h. Afterwards the mixture was allowed to cool down for 5 min, water (18 µL, 1.00 mmol, 0.50 eq.) was added and the mixture was again heated at 110 °C for 10 min. Subsequently, the reaction was cooled to r.t. and stirred for 3 h. The resulting yellow precipitate was collected via vacuum filtration, washed with MeCN and dried *in vacuo* to yield the product as a yellow solid (831 mg, 1.42 mmol, 71%).

^{1}H NMR (400 MHz, CD_2Cl_2): δ = 8.29 (d, *J* = 1.9 Hz, 1H), 8.11 (d, *J* = 1.9 Hz, 1H), 7.49 (dd, *J* = 3.7, 2.7 Hz, 2H), 7.05 (d, *J* = 2.6 Hz, 1H), 6.98 (d, *J* = 2.6 Hz, 1H), 3.54 (t, *J* = 10.1 Hz, 1H), 3.07 (d,

J = 10.9 Hz, 1H), 2.58 (d, J = 9.8 Hz, 1H), 2.41 (d, J = 9.5 Hz, 1H), 2.08 (d, J = 9.4 Hz, 2H), 1.52 (d, J = 2.6 Hz, 20H), 1.32 – 1.30 (m, 20H), –1.14 (s, 3H) ppm.

HR-ESI-MS: *m/z*: 571.38334 ($[M{-}CH_3]^+$, calcd. for $C_{36}H_{52}AlN_2O_2^+$: 571.38387).

$[\boldsymbol{\alpha}]_{\mathbf{D}}^{\mathbf{25}\ ^\circ\mathbf{C}}$ = –624.4 (c = 1.0 in $CHCl_3$).

Analytical data in agreement with literature.[24]

6π-Photocyclization: Procedure and Product Characterization

Note: All reagents and substrates used in 6π-cyclization reactions were dried *in vacuo* and stored in a glovebox. Molecular sieves and vials were dried in an oven at 120 °C for at least 14 h before being stored in the glovebox. Aluminum catalysts were re-crystallized from *n*-hexanes/CH_2Cl_2 prior to use. Dry solvents were purchased in AcroSeal™ bottles and purged with Argon for 30 min before being stored in a glovebox.

General Procedure D: Photocatalytic 6π-Cyclization (standard conditions)

Reactions at low temperature: In a glovebox, a vial was charged with a stir bar, acrylanilide (0.10 mmol, 1.00 eq.), aluminum catalyst **Al-1** (12.1 mg, 0.02 mmol, 20 mol%), tetrabutylammonium chloride (41.7 mg, 0.15 mmol, 1.50 eq.) and 3 Å molecular sieves (6.0 mg). Dry DCM (1.5 mL) was added, the vial was capped with a cap with pre-slit septum and the mixture was stirred until the catalyst and the acrylanilide were fully dissolved. Insertion of the glass rod into the solution was followed by sealing with Parafilm®. The vial was removed from the glovebox, placed in the low temperature photoreactor and cooled to –20 °C, before being irradiated at 400 nm for 21 h. Afterwards the reaction mixture was warmed to room temperature and concentrated under reduced pressure. Following purification by column chromatography (SiO_2, *n*-pentane/EtOAc) the product was obtained.

General Procedure E: Photocatalytic 6π-Cyclization at room temperature

Reactions at room temperature: In a glovebox, a pressure tube was charged with a stir bar, acrylanilide (0.10 mmol, 1.00 eq.), aluminum catalyst **Al-1** (12.1 mg, 0.02 mmol, 20 mol%), tetrabutylammonium chloride (41.7 mg, 0.15 mmol, 1.50 eq.) and 3 Å molecular sieves (6.0 mg). Dry DCM (1.5 mL) was added, and the tube was sealed, removed from the glovebox, and irradiated at 400 nm and room temperature for 21 h. Afterwards the reaction mixture was concentrated under reduced pressure. Following purification by column chromatography (SiO_2, *n*-pentane/EtOAc) the product was obtained.

Note: In reactions with HFIP instead of tetrabutylammonium chloride, HFIP (15.8 µL, 0.15 mmol, 1.50 eq.) was added following the addition of DCM.

General Procedure F: Photocatalytic 6π-Cyclization with HFIP at –40°C

In a glovebox, a vial was charged with a stir bar, acrylanilide (0.10 mmol, 1.00 eq.), aluminum catalyst **Al-1** (12.1 mg, 0.02 mmol, 20 mol%) and 3 Å molecular sieves (6.0 mg). Dry DCM (1.5 mL) and HFIP (15.8 µL, 0.15 mmol, 1.50 eq.) was added, the vial was capped with a cap with pre-slit septum and the mixture was stirred until the catalyst and the acrylanilide were fully dissolved. Insertion of the glass rod into the solution was followed by sealing with Parafilm®. The vial was removed from the glovebox, placed in the low temperature photoreactor and cooled to –40 °C, before being irradiated at 400 nm for 21 h. Afterwards the reaction mixture was warmed to room temperature and concentrated under reduced pressure. Following purification by column chromatography (SiO_2, *n*-pentane/EtOAc) the product was obtained.

6a,7,8,9,10,10a-Hexahydrophenanthridin-6(*5H*)-one (2a)

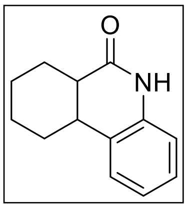

According to General Procedure **D**, **1a** (20.1 mg, 0.10 mmol, 1.00 eq.) was converted to **2a** yielding the product as a white solid (13.5 mg, 67%) after purification by column chromatography (SiO_2, 20 % EtOAC in *n*-pentane). The product was obtained as an inseparable mixture of diastereomers (d.r. 66:34 (*trans*:*cis*)).

R_f = 0.52 (*n*-pentane/EtOAc 7:3, both diastereomers).

HR-ESI-MS: *m/z*: 224.10451 ([*M*+Na]$^+$, calcd. for $C_{14}H_{15}NONa^+$: 224.10459).

trans-diastereomer:

^{1}H NMR (400 MHz, $CDCl_3$): δ = 8.01 (s, 1H), 7.21 – 7.16 (m, 2H), 7.04 (td, *J* = 7.6, 1.3 Hz, 1H), 6.76 (dd, *J* = 7.9, 1.3 Hz, 1H), 2.68 – 2.57 (m, 1H), 2.49 (dd, *J* = 9.1, 4.3 Hz, 1H), 2.45 – 2.38 (m, 1H), 2.13 – 2.02 (m, 1H), 1.99 – 1.90 (m, 2H), 1.44 – 1.21 (m, 4H) ppm.

e.r.: 84:16.

cis-diastereomer:

^{1}H NMR (400 MHz, $CDCl_3$): δ = 8.01 (s, 1H), 7.15 (m, 2H), 7.00 (td, *J* = 7.5, 1.2 Hz, 1H), 6.74 (dd, *J* = 8.2, 1.2 Hz, 1H), 2.95 (dt, *J* = 10.4, 5.1 Hz, 1H), 2.81 (q, *J* = 4.4 Hz, 1H), 1.77 – 1.45 (m, 8H) ppm.

e.r.: 90:10.

Analytical data in agreement with literature.[3]

HPLC: AS-H column; eluent: *n*-hexane/ *i*-propanol 15:85; flow rate: 1.0 mL/min.

Racemic sample: ***trans*-2a**

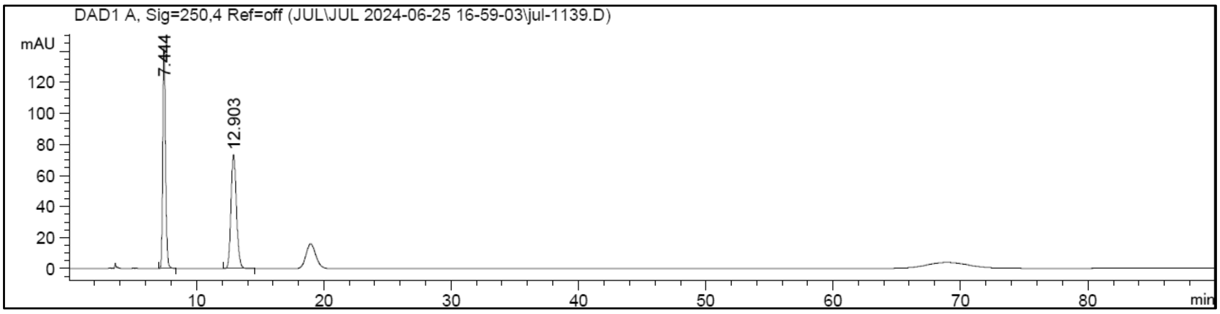

Signal 1: DAD1 A, Sig=250,4 Ref=off

Peak #	RetTime [min]	Type	Width [min]	Area [mAU*s]	Height [mAU]	Area %
1	7.444	BB	0.2340	2190.28271	143.59508	49.9709
2	12.903	BB	0.4590	2192.83545	73.73271	50.0291

Enantioselective sample: ***trans*-2a**

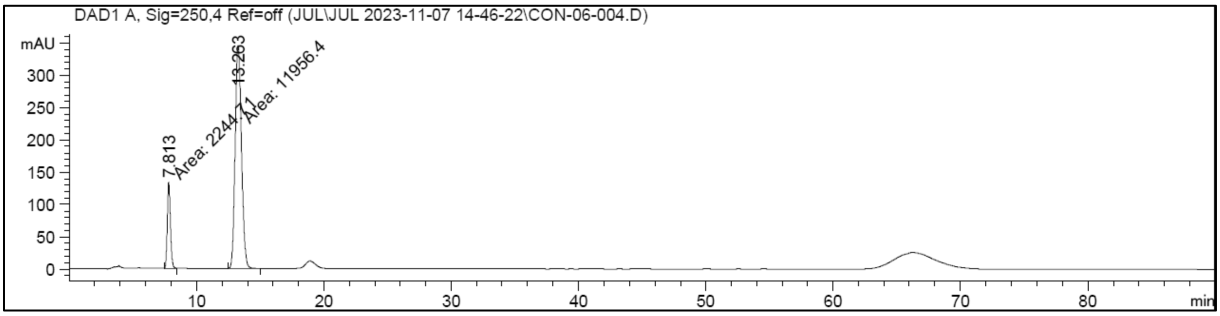

Signal 1: DAD1 A, Sig=250,4 Ref=off

Peak #	RetTime [min]	Type	Width [min]	Area [mAU*s]	Height [mAU]	Area %
1	7.813	MM	0.2808	2244.70679	133.25461	15.8065
2	13.263	MM	0.5770	1.19564e4	345.37857	84.1935

Racemic sample: ***cis*-2a**

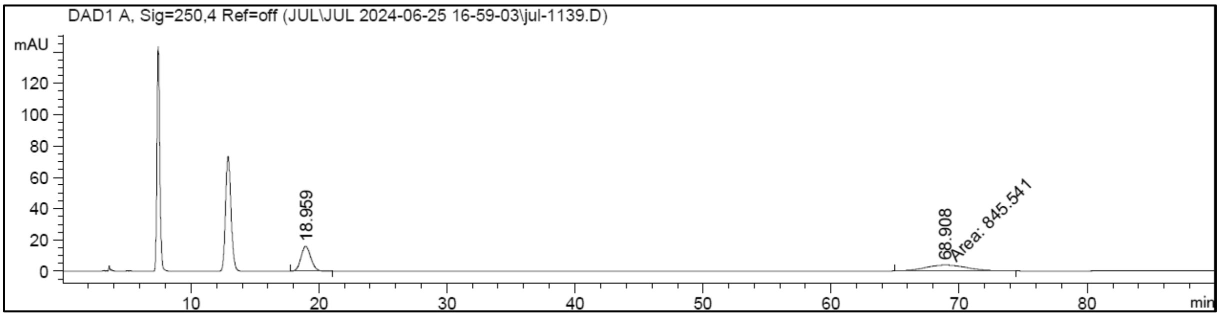

Signal 1: DAD1 A, Sig=250,4 Ref=off

Peak #	RetTime [min]	Type	Width [min]	Area [mAU*s]	Height [mAU]	Area %
1	18.959	BB	0.8097	853.30908	15.85943	50.2286
2	68.908	MM	3.7196	845.54071	3.78863	49.7714

Enantioselective sample: ***cis*-2a**

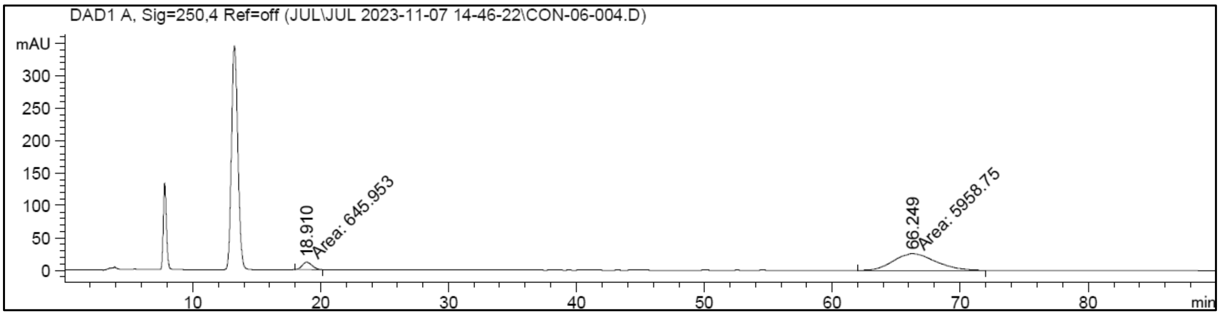

Signal 1: DAD1 A, Sig=250,4 Ref=off

Peak #	RetTime [min]	Type	Width [min]	Area [mAU*s]	Height [mAU]	Area %
1	18.910	MM	0.9167	645.95258	11.74473	9.7802
2	66.249	MM	3.8356	5958.74609	25.89202	90.2198

2-Fluoro-6a,7,8,9,10,10a-hexahydrophenanthridin-6(5H)-one (2b)

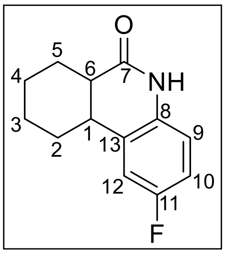

According to General Procedure **D**, **1b** (21.9 mg, 0.10 mmol, 1.00 eq.) was converted to **2b** yielding the product as white solid (13.5 mg, 62%) after purification by column chromatography (SiO_2, 25 % EtOAc in *n*-pentane). The product was obtained as an inseparable mixture of diastereomers (d.r. 66:34 (*trans*:*cis*)).

R*f* = 0.22 (20% EtOAc in *n*-pentane, both diastereomers).

HR-ESI-MS: *m/z*: 242.09509 ([*M*+Na]$^+$, calcd. for $C_{13}H_{14}NOFNa^+$: 242.09516).

trans-diastereomer:

^{1}H NMR (500 MHz, DMSO-d$_6$): δ = 10.05 (s, 1H NH), 7.02 – 6.92 (m, 2H, H10, H12), 6.82 (dd, *J* = 8.7, 5.2 Hz, 1H, H9), 2.52 – 2.42 (m, 1H, H1), 2.35 (d, *J* = 12.2 Hz, 1H; H2), 2.20 – 2.10 (m, 1H, H5), 1.90 (ddd, *J* = 14.5, 11.0, 3.6 Hz, 1H, H6), 1.77 (t, *J* = 14.4 Hz, 2H, H3, H4), 1.28 – 1.14 (m, 4H, H2, H3, H4, H5) ppm.

^{13}C NMR (126 MHz, DMSO-d$_6$): δ = 171.5 (C7), 157.9 (d, *J* = 237.5 Hz, C11), 134.0 (d, *J* = 2.1 Hz, C8), 130.1 (d, *J* = 7.0 Hz, C13), 116.0 (d, *J* = 8.2 Hz, C9), 113.4 (d, *J* = 22.6 Hz, C10/C12), 111.3 (d, *J* = 23.7 Hz, C10/C12), 42.0 (C6), 37.4 (C1), 28.2 (C2), 26.0 (C5), 24.7 (C3, C4) ppm.

^{19}F NMR (470 MHz, DMSO-d$_6$): δ = –121.21 (td, *J* = 9.2, 5.1 Hz) ppm.

e.r.: 81:19.

cis-diastereomer:

^{1}H NMR (500 MHz, DMSO-d$_6$): δ = 10.01 (s, 1H, NH), 6.94 – 6.88 (m, 2H, H10, H12), 6.79 – 6.77 (m, 1H, H9), 2.91 – 2.85 (m, 1H, H1), 2.63 (d, *J* = 5.1 Hz, 1H, H6), 2.11 – 2.04 (s, 1H, H5), 1.58

(s, 1H, H3), 1.56 – 1.49 (m, 1H, H2), 1.44 (d, *J* = 12.0 Hz, 1H, H4), 1.38 – 1.28 (m, 4H, H2, H3, H4, H5) ppm.

^{13}C NMR (126 MHz, DMSO-d$_6$): δ = 170.9 (C7), 157.6 (d, *J* = 237.9 Hz, C11), 133.7 (C8), 130.1 (C13), 116.1 (d, *J* = 8.1 Hz, C9), 113.9 (d, *J* = 22.8 Hz; C10/C12), 113.5 (d, *J* = 22.6 Hz, C10/C12), 39.6 (C6), 38.3 (C1), 29.7 (C2), 25.2 (C3), 24.0 (C5), 22.9 (C4) ppm. C1, C2, C3, C6 were assigned via 2D NMR.

^{19}F NMR (470 MHz, DMSO-d$_6$): δ = –121.46 (td, *J* = 9.0, 5.0 Hz) ppm.

e.r.: 92:8.

HPLC: AS-H column; eluent: *n*-hexane/ *i*-propanol 15:85; flow rate: 1.0 mL/min.

Racemic sample: ***trans*-2b**

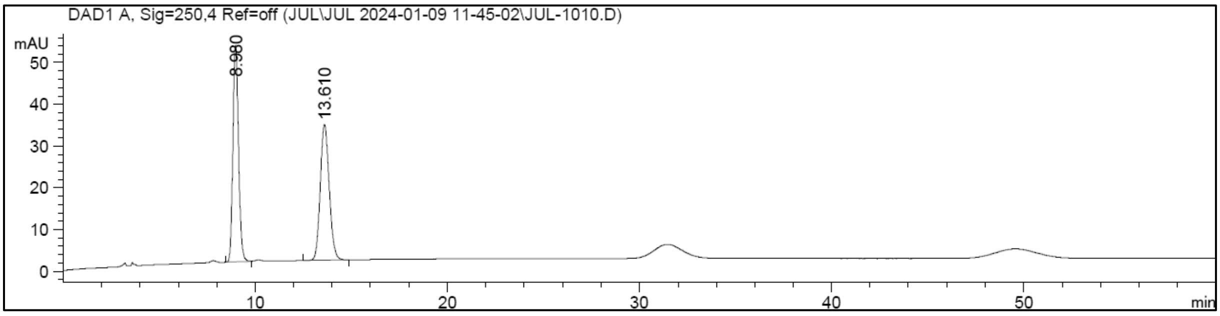

Signal 1: DAD1 A, Sig=250,4 Ref=off

Peak #	RetTime [min]	Type	Width [min]	Area [mAU*s]	Height [mAU]	Area %
1	8.980	BB	0.3112	1045.25208	51.85701	49.8532
2	13.610	BB	0.4975	1051.40771	32.49097	50.1468

Enantioselective sample: ***trans*-2b**

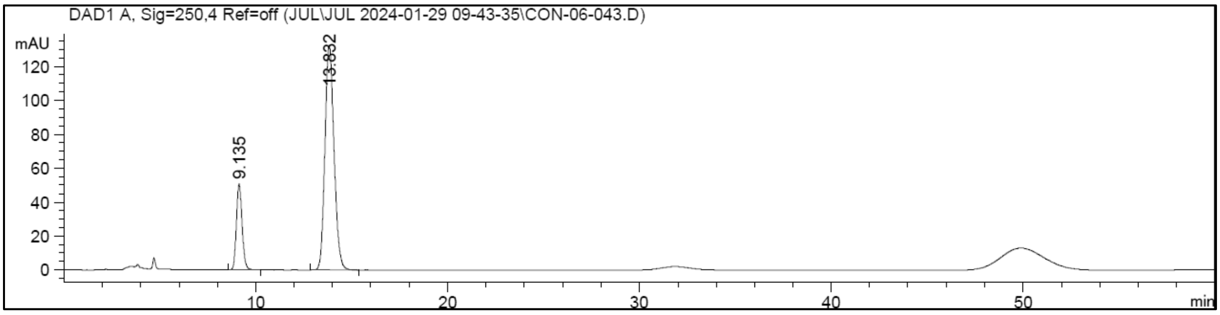

Signal 1: DAD1 A, Sig=250,4 Ref=off

Peak #	RetTime [min]	Type	Width [min]	Area [mAU*s]	Height [mAU]	Area %
1	9.135	BB	0.3185	1045.38245	50.71534	19.3194
2	13.832	BB	0.5104	4365.66113	132.48251	80.6806

Racemic sample: ***cis*-2b**

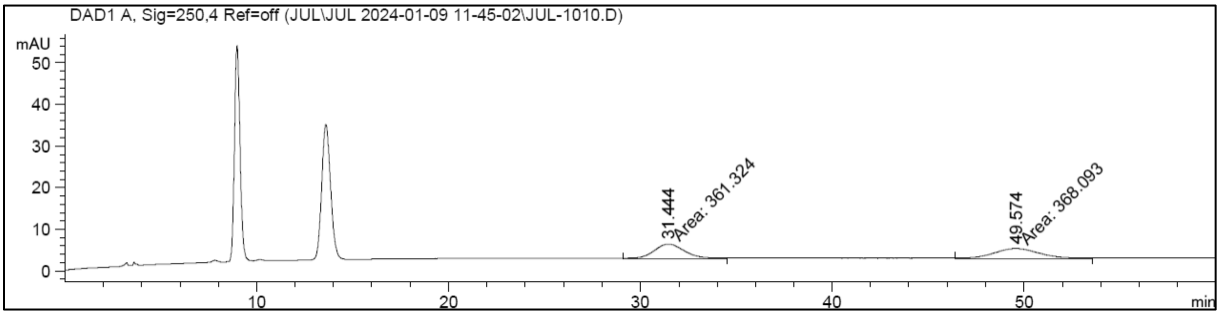

Signal 1: DAD1 A, Sig=250,4 Ref=off

Peak #	RetTime [min]	Type	Width [min]	Area [mAU*s]	Height [mAU]	Area %
1	31.444	MM	1.7674	361.32422	3.40731	49.5360
2	49.574	MM	2.6469	368.09299	2.31777	50.4640

Enantioselective sample: ***cis*-2b**

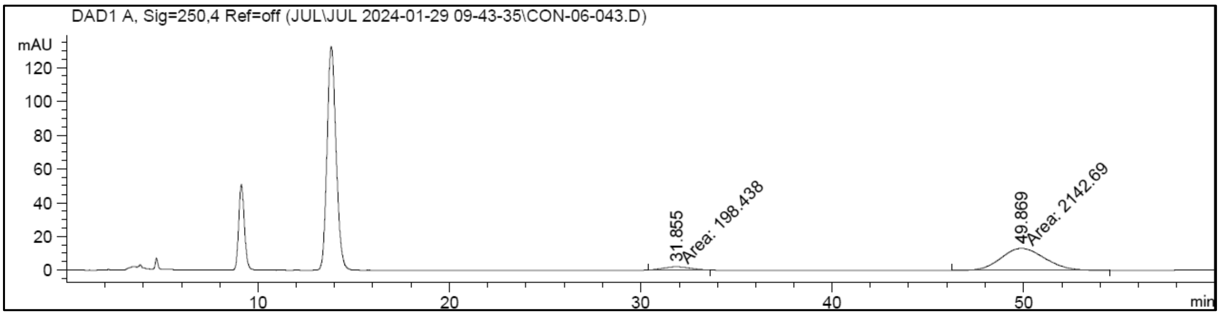

Signal 1: DAD1 A, Sig=250,4 Ref=off

Peak #	RetTime [min]	Type	Width [min]	Area [mAU*s]	Height [mAU]	Area %
1	31.855	MF	1.6742	198.43768	1.97549	8.4761
2	49.869	MM	2.7099	2142.69434	13.17796	91.5239

2-Chloro-6a,7,8,9,10,10a-hexahydrophenanthridin-6(5H)-one (2c)

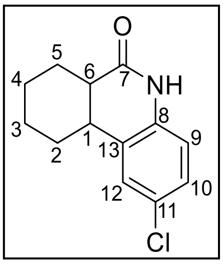

According to General Procedure **D**, **1c** (23.6 mg, 0.10 mmol, 1.00 eq.) was converted to **2c** yielding the product as white solid (14.7 mg, 62%) after purification by column chromatography (SiO_2, 25 % EtOAc in *n*-pentane). The product was obtained as an inseparable mixture of diastereomers (d.r. 67:33 (*trans*:*cis*)).

R*f* = 0.24 (20% EtOAc in *n*-pentane, both diastereomers).

HR-ESI-MS: *m/z*: 258.0655 ([*M*+Na]$^+$, calcd. for $C_{13}H_{14}NOClNa^+$: 258.0655).

trans-diastereomer:

^{1}H NMR (500 MHz, $CDCl_3$): δ = 8.67 (s, 1H, NH), 7.19 (t, *J* = 1.8 Hz, 1H, H12), 7.15 (ddd, *J* = 8.3, 2.3, 0.9 Hz, 1H, H10), 6.75 (d, *J* = 8.4 Hz, 1H, H9), 2.59 (ddd, *J* = 14.3, 10.4, 4.1 Hz, 1H, H1), 2.49 – 2.38 (m, 2H, H2, H5), 2.04 (ddd, *J* = 14.3, 11.5, 3.7 Hz, 1H, H6), 1.99 – 1.91 (m, 2H, H3, H4), 1.41 – 1.28 (m, 4H, H2, H3, H4, H5) ppm.

^{13}C NMR (126 MHz, $CDCl_3$): δ = 173.5 (C7), 135.5 (C8), 130.3 (C13), 128.4 (C11), 127.4 (C10), 124.9 (C12), 116.4 (C9), 43.1 (C6), 38.0 (C1), 28.8 (C2), 26.2 (C5), 25.6 (C3/C4), 25.23 (C3/C4) ppm.

e.r.: 80:20.

cis-diastereomer:

^{1}H NMR (500 MHz, $CDCl_3$): δ = 8.70 (s, 1H, NH), 7.14 – 7.11 (m, 2H, H10, H12), 6.74 – 6.71 (m, 1H, H9), 2.94 (dt, *J* = 10.2, 5.1 Hz, 1H, H1), 2.77 (q, *J* = 4.7 Hz, 1H, H6), 2.30 (s, 1H, H5), 1.77 – 1.69 (m, 1H, H4), 1.69 – 1.62 (m, 1H, H2), 1.62 – 1.56 (m, 1H, H2, H3), 1.55 – 1.48 (m, 2H, H3, H5), 1.48 – 1.41 (m, 1H, H4) ppm.

^{13}C NMR (126 MHz, $CDCl_3$): δ = 173.0 (C7), 135.2 (C8), 130.3 (C13), 128.1 (C11), 127.43 (C10/C12), 127.40 (C10/C12), 116.6 (C9), 40.6 (C6), 39.0 (C1), 29.5 (C2), 24.8 (C3), 24.5 (C5), 22.8 (C4) ppm. C3 was assigned via 2D NMR.

e.r.: 91:9.

HPLC: AS-H column; eluent: *n*-hexane/ *i*-propanol 15:85; flow rate: 1.0 mL/min.

Racemic sample: ***trans*-2c**

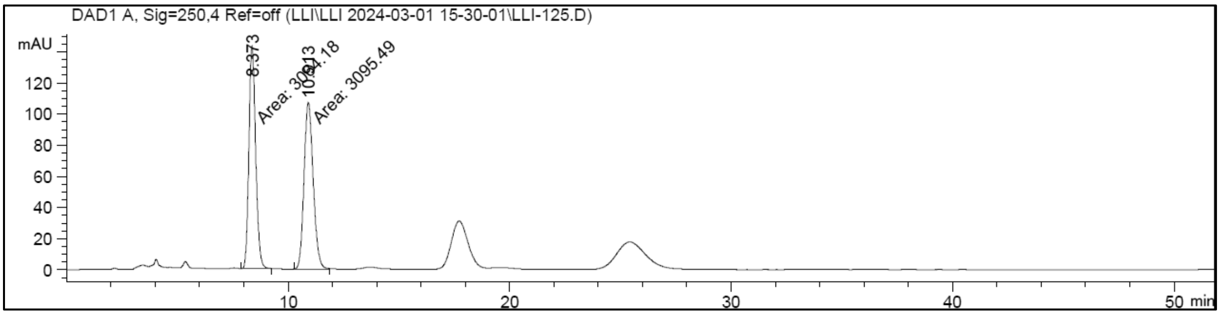

Signal 1: DAD1 A, Sig=250,4 Ref=off

Peak #	RetTime [min]	Type	Width [min]	Area [mAU*s]	Height [mAU]	Area %
1	8.373	MF	0.3562	3064.18188	143.35715	49.7459
2	10.913	FM	0.4820	3095.48706	107.04244	50.2541

Enantioselective sample: ***trans*-2c**

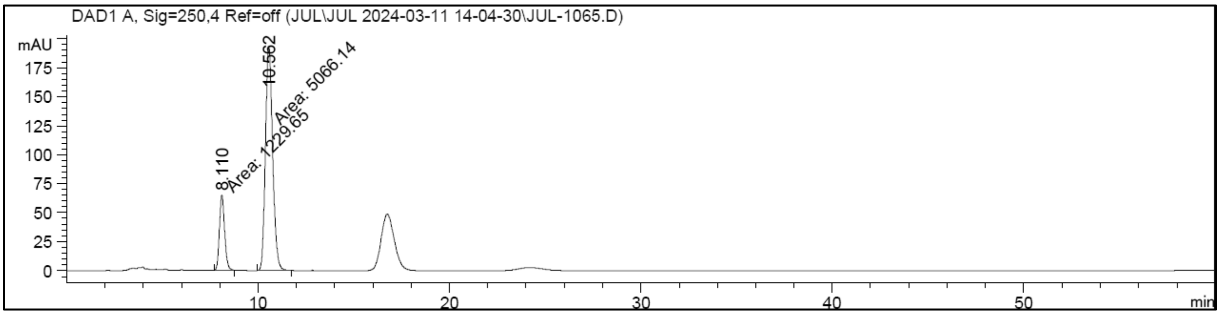

Signal 1: DAD1 A, Sig=250,4 Ref=off

Peak #	RetTime [min]	Type	Width [min]	Area [mAU*s]	Height [mAU]	Area %
1	8.110	MM	0.3153	1229.64563	65.00626	19.5312
2	10.562	MM	0.4368	5066.14307	193.30276	80.4688

Racemic sample: ***cis*-2c**

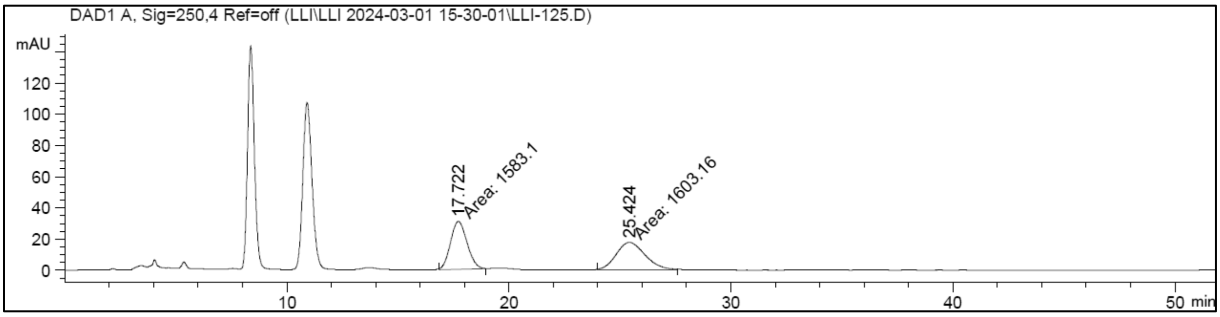

Signal 1: DAD1 A, Sig=250,4 Ref=off

Peak #	RetTime [min]	Type	Width [min]	Area [mAU*s]	Height [mAU]	Area %
1	17.722	FM	0.8604	1583.09583	30.66680	49.6851
2	25.424	FM	1.5174	1603.16016	17.60835	50.3149

Enantioselective sample: ***cis*-2c**

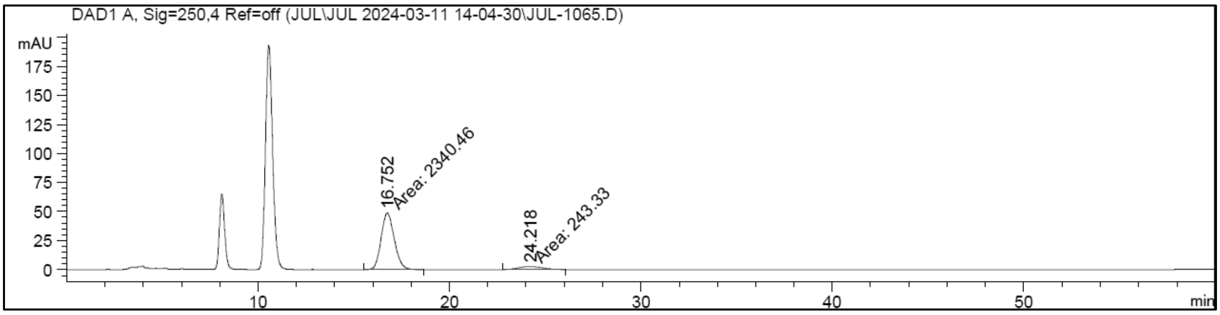

Signal 1: DAD1 A, Sig=250,4 Ref=off

Peak #	RetTime [min]	Type	Width [min]	Area [mAU*s]	Height [mAU]	Area %
1	16.752	MM	0.8016	2340.45703	48.66380	90.5824
2	24.218	MF	1.4589	243.32983	2.77985	9.4176

2-(*tert*-Butyl)-6a,7,8,9,10,10a-hexahydrophenanthridin-6(5H)-one (2d)

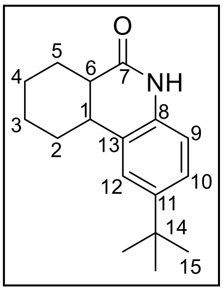

According to General Procedure **D**, **1d** (25.7 mg, 0.10 mmol, 1.00 eq.) was converted to **2d** yielding the product as white solid (17.7 mg, 69%) after purification by column chromatography (SiO_2, 20% EtOAc in *n*-pentane). The product was obtained as an inseparable mixture of diastereomers (d.r. 65:35 (*trans*:*cis*)).

R_f = 0.33 (20% EtOAc in *n*-pentane, both diastereomers).

HR-ESI-MS: *m/z*: 280.16715 ($[M+Na]^+$, calcd. for $C_{17}H_{23}NONa^+$: 280.16719).

trans-diastereomer:

^{1}H NMR (600 MHz, $CDCl_3$): δ = 8.22 (s, 1H, NH), 7.26 (m, 1H, H12), 7.21 (ddd, *J* = 8.2, 2.1, 0.9 Hz, 1H, H10), 6.73 (d, *J* = 8.2 Hz, 1H, H9), 2.66 – 2.59 (m, 1H, H1), 2.57 – 2.50 (m, 1H, H2), 2.42 (dq, *J* = 13.2, 2.8 Hz, 1H, H5), 2.08 (ddd, *J* = 13.9, 11.6, 3.7 Hz, 1H, H6), 2.00 – 1.91 (m, 2H, H3, H4), 1.46 – 1.35 (m, 4H, H2, H3, H4, H5), 1.31 (s, 9H, H15) ppm.

^{13}C NMR (151 MHz, $CDCl_3$): δ = 173.5 (C7), 146.0 (C8), 134.3 (C13), 127.9 (C11), 124.2 (C10), 121.4 (C12), 114.8 (C9), 43.5 (C6), 38.2 (C1), 34.7 (C14), 31.6 (C15), 28.9 (C2), 26.3 (C5), 25.43 (C3/C4), 25.41 (C3/C4) ppm.

e.r.: 95:5.

cis-diastereomer:

^{1}H NMR (600 MHz, $CDCl_3$): δ = 8.22 (s, 1H, NH), 7.19 – 7.14 (m, 2H, H10, H12), 6.70 (d, *J* = 8.1 Hz, 1H, H9), 2.93 (dt, *J* = 10.7, 4.9 Hz, 1H, H1), 2.84 – 2.77 (m, 1H, H6), 2.39 (d, *J* = 11.6 Hz, 1H, H5), 1.77 – 1.72 (m, 1H, H3), 1.66 (dd, *J* = 13.8, 4.0 Hz, 1H, H2), 1.64 – 1.55 (m, 2H, H2, H4), 1.54 – 1.45 (m, 2H, H4, H5), 1.30 (s, 9H, H15), 1.30 – 1.25 (m, 1H, H3) ppm.

^{13}C NMR (151 MHz, $CDCl_3$): δ = 172.9 (C7), 146.3 (C8), 133.9 (C13), 128.3 (C11), 124.4 (C12), 124.2 (C10), 114.9 (C9), 40.8 (C6), 39.7 (C1), 34.5 (C14), 31.6 (C15), 30.2 (C2), 25.4 (C3), 24.7 (C5), 22.8 (C4) ppm.

e.r.: 94:6.

HPLC: AD-H column; eluent: *n*-hexane/ *i*-propanol 90:10; flow rate: 1.0 mL/min.

Racemic sample: ***trans*-2d**

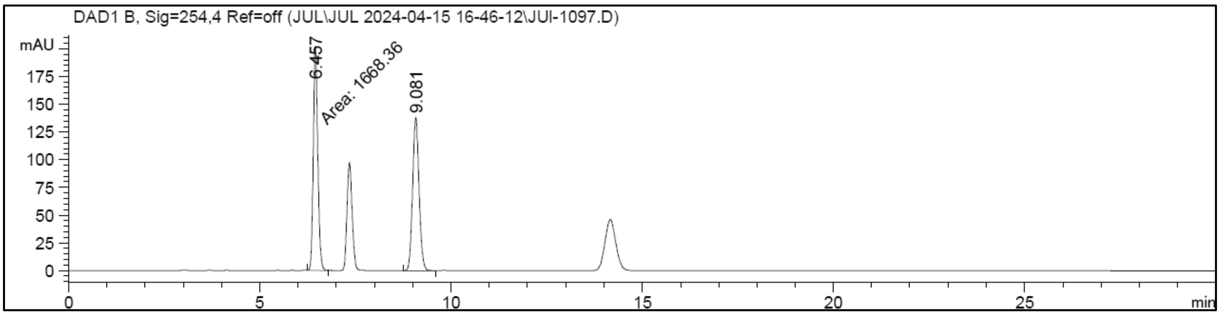

Signal 2: DAD1 B, Sig=254,4 Ref=off

Peak #	RetTime [min]	Type	Width [min]	Area [mAU*s]	Height [mAU]	Area %
1	6.457	MF	0.1378	1668.36316	201.80710	50.7066
2	9.081	BB	0.1834	1621.86584	137.75691	49.2934

Enantioselective sample: ***trans*-2d**

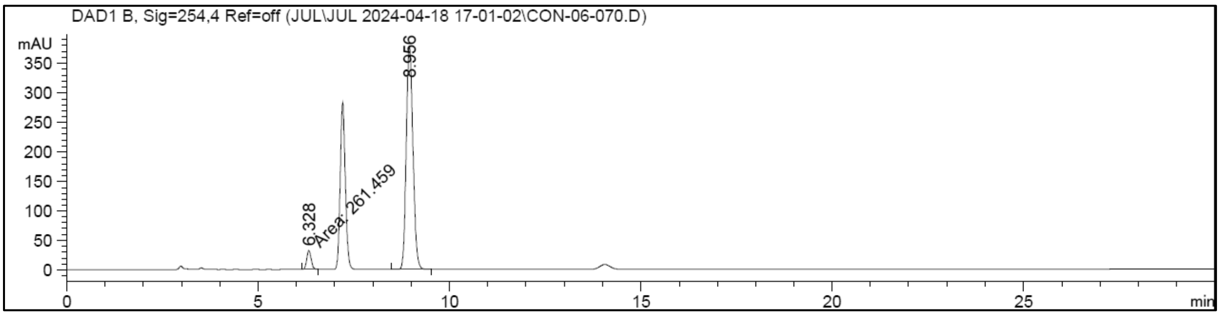

Signal 2: DAD1 B, Sig=254,4 Ref=off

Peak #	RetTime [min]	Type	Width [min]	Area [mAU*s]	Height [mAU]	Area %
1	6.328	FM	0.1356	261.45862	32.13293	5.3681
2	8.956	BB	0.1879	4609.15137	379.00790	94.6319

Racemic sample: ***cis*-2d**

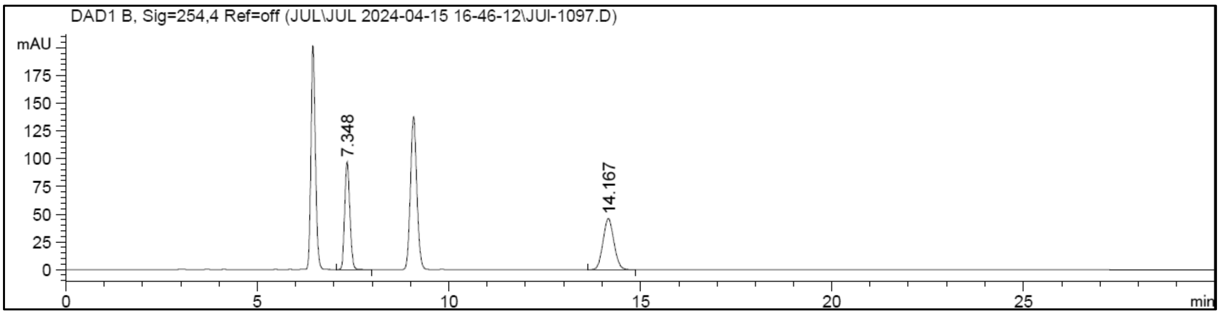

Signal 2: DAD1 B, Sig=254,4 Ref=off

Peak #	RetTime [min]	Type	Width [min]	Area [mAU*s]	Height [mAU]	Area %
1	7.348	BB	0.1421	898.68164	96.97955	49.6717
2	14.167	BB	0.3068	910.56024	46.02934	50.3283

Enantioselective sample: ***cis*****-2d**

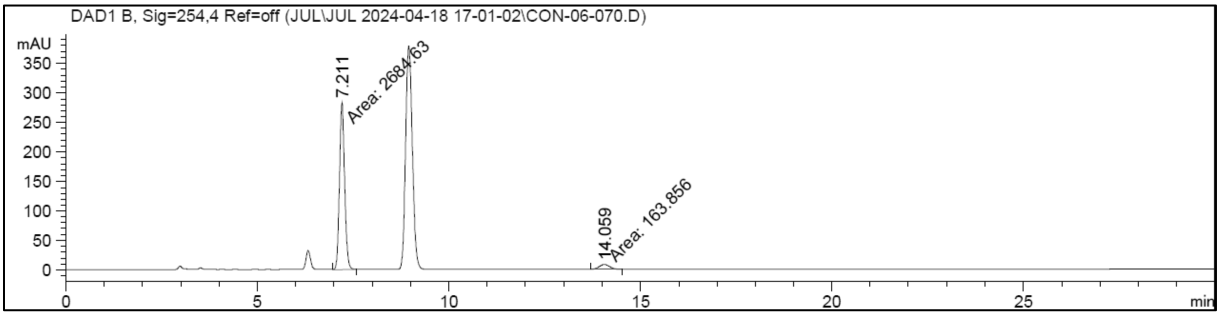

Signal 2: DAD1 B, Sig=254,4 Ref=off

Peak #	RetTime [min]	Type	Width [min]	Area [mAU*s]	Height [mAU]	Area %
1	7.211	MM	0.1580	2684.63257	283.19339	94.2476
2	14.059	MM	0.3273	163.85625	8.34269	5.7524

2-Ethynyl-6a,7,8,9,10,10a-hexahydrophenanthridin-6(5H)-one (2e)

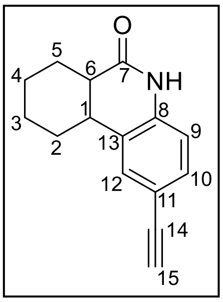

According to General Procedure **D**, **1e** (22.5 mg, 0.10 mmol, 1.00 eq.) was converted to **2e** yielding the product as white solid (5.2 mg, 23%) after purification by column chromatography (SiO_2, 20% EtOAc in *n*-pentane). The product was obtained as an inseparable mixture of diastereomers (d.r. 68:32 (*trans*:*cis*)).

When HFIP (1.5 eq.) was added as an additive instead of nBu$_4$NCl, the product was obtained in 56% yield with e.r. 78:22 for both diastereomers (d.r. 1:1).

R*f* = 0.31 (25% EtOAc in *n*-pentane, both diastereomers).

HR-ESI-MS: *m/z*: 248.10454 ([*M*+Na]$^+$, calcd. for $C_{15}H_{15}NONa^+$: 248.10459).

trans-diastereomer:

^{1}H NMR (500 MHz, $CDCl_3$): δ = 8.77 (s, 1H, NH), 7.36 (d, *J* = 1.5 Hz, 1H, H12), 7.33 (ddd, *J* = 8.1, 1.8, 0.9 Hz, 1H, H10), 6.76 (d, *J* = 8.1 Hz, 1H, H9), 3.03 (s, 1H, H15), 2.62 – 2.55 (m, 1H, H1), 2.47 (dd, *J* = 8.9, 4.6 Hz, 1H, H2), 2.42 (dt, J = 14.6, 2.4 Hz, 1H, H5), 2.06 (ddd, *J* = 14.4, 11.5, 3.7 Hz, 1H, H6), 2.00 – 1.93 (m, 2H, H3, H4), 1.44 – 1.32 (m, 4H, H2, H3, H4, H5) ppm.

^{13}C NMR (126 MHz, $CDCl_3$): δ = 173.8 (C7), 137.3 (C8), 131.6 (C10), 128.6 (C13), 128.5 (C12), 116.6 (C11), 115.3 (C9), 83.9 (C14), 76.4 (C15), 43.2 (C6), 37.8 (C1), 28.7 (C2), 26.2 (C5), 25.27 (C4), 25.25 (C3) ppm.

e.r.: 87:13.

cis-diastereomer:

^{1}H NMR (500 MHz, $CDCl_3$): δ = 8.80 (s, 1H, NH), 7.31 – 7.28 (m, 2H, H10, H12), 6.74 (d, *J* = 8.7 Hz, 1H, H9), 3.03 (s, 1H, H15), 2.94 (dt, *J* = 10.3, 5.2 Hz, 1H, H1), 2.81 – 2.77 (m, 1H, H6), 2.30 (s, 1H, H5), 1.94 – 1.91 (m, 1H, H3), 1.75 – 1.70 (m, 1H, H3), 1.68 – 1.57 (m, 3H, H2, H4), 1.53 – 1.46 (m, 2H, H4, H5) ppm.

^{13}C NMR (126 MHz, $CDCl_3$): δ = 173.3 (C7), 137.0 (C8), 131.6 (C10), 131.3 (C12), 128.6 (C13), 116.8 (C11), 115.4 (C9), 83.7 (C14), 76.5 (C15), 40.7 (C6), 38.8 (C1), 29.6 (C2), 24.8 (C3), 24.5 (C5), 22.8 (C4) ppm. C3 was assigned via 2D NMR.

e.r.: 92:8.

HPLC: AD-H column; eluent: *n*-hexane/ *i*-propanol 95:5; flow rate: 1.0 mL/min.

Racemic sample: ***trans*-2e**

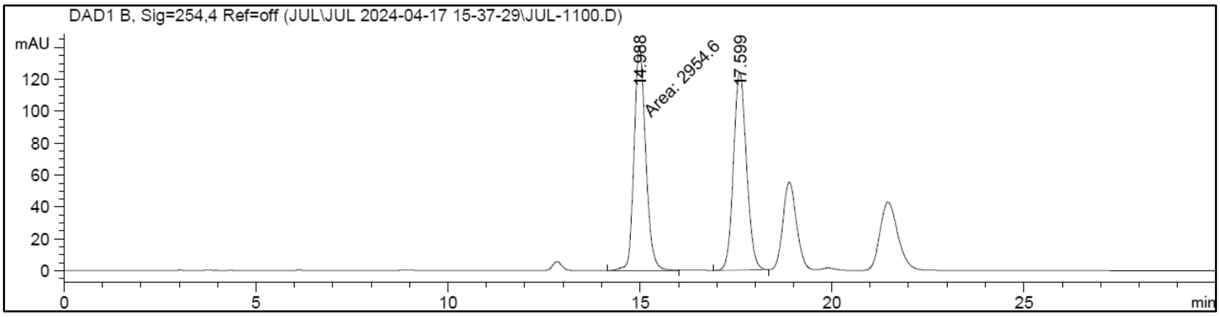

Signal 2: DAD1 B, Sig=254,4 Ref=off

Peak #	RetTime [min]	Type	Width [min]	Area [mAU*s]	Height [mAU]	Area %
1	14.988	MF	0.3491	2954.60229	141.04112	50.2553
2	17.599	BB	0.3654	2924.57910	123.89942	49.7447

Enantioselective sample: ***trans*-2e**

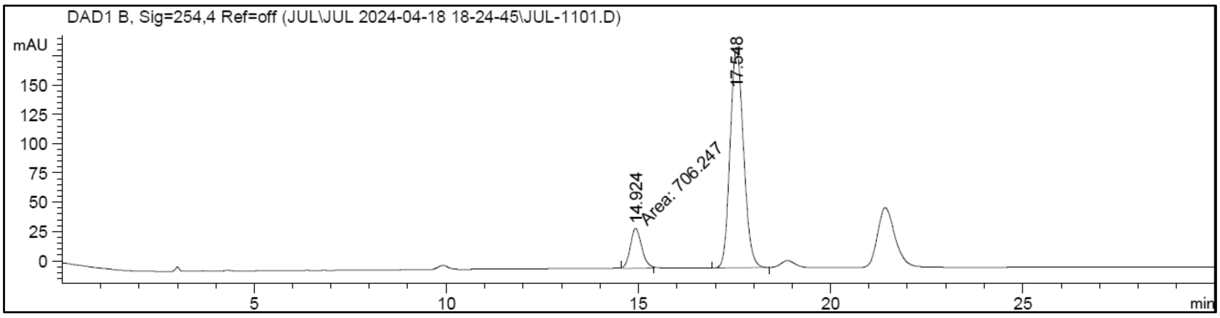

Signal 2: DAD1 B, Sig=254,4 Ref=off

Peak #	RetTime [min]	Type	Width [min]	Area [mAU*s]	Height [mAU]	Area %
1	14.924	FM	0.3461	706.24658	34.01162	13.4525
2	17.548	BB	0.3775	4543.66992	188.30681	86.5475

Racemic sample: ***cis*-2e**

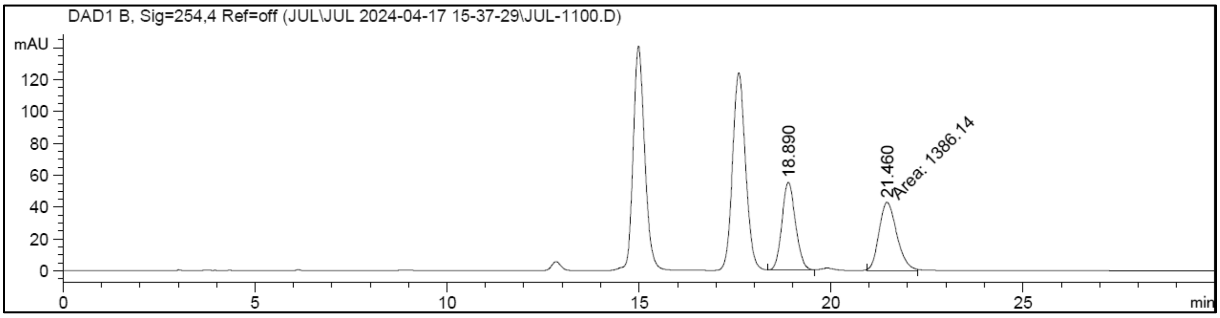

Signal 2: DAD1 B, Sig=254,4 Ref=off

Peak #	RetTime [min]	Type	Width [min]	Area [mAU*s]	Height [mAU]	Area %
1	18.890	BB	0.3774	1345.83289	55.01849	49.2624
2	21.460	FM	0.5380	1386.13513	42.94101	50.7376

Enantioselective sample: ***cis*-2e**

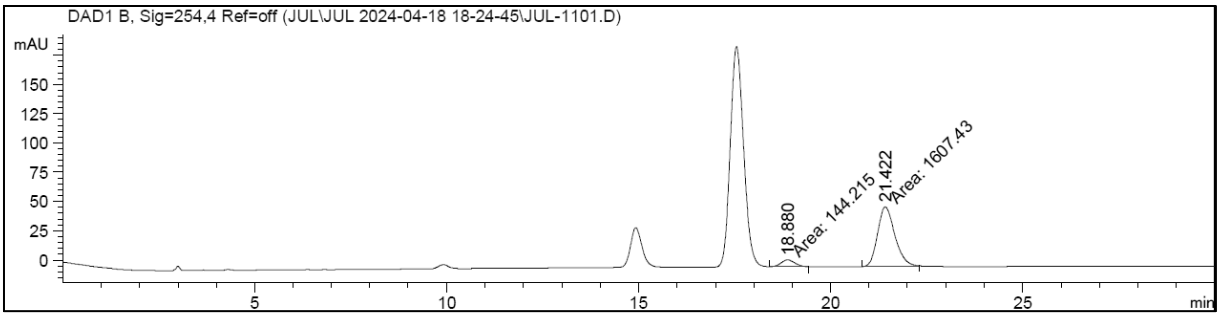

Signal 2: DAD1 B, Sig=254,4 Ref=off

Peak #	RetTime [min]	Type	Width [min]	Area [mAU*s]	Height [mAU]	Area %
1	18.880	MF	0.4162	144.21492	5.77540	8.2331
2	21.422	FM	0.5260	1607.42639	50.93195	91.7669

2-(Trimethylsilyl)-6a,7,8,9,10,10a-hexahydrophenanthridin-6(5H)-one (2f)

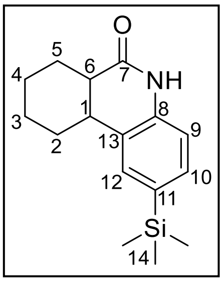

According to General Procedure **D**, **1f** (27.3 mg, 0.10 mmol, 1.00 eq.) was converted to **2f** yielding the product as white solid (16.7 mg, 61%) after purification by column chromatography (SiO_2, 15% EtOAc in *n*-pentane). The product was obtained as an inseparable mixture of diastereomers (d.r. 72:28 (*trans*:*cis*)).

R*f* = 0.57 (30% EtOAc in *n*-pentane, both diastereomers).

HR-ESI-MS: *m/z*: 296.14398 ([*M*+Na]$^+$, calcd. for $C_{16}H_{23}NOSiNa^+$: 296.14411).

trans-diastereomer:

^{1}H NMR (600 MHz, $CDCl_3$): δ = 8.47 (s, 1H, NH), 7.37 (d, *J* = 1.4 Hz, 1H, H12), 7.35 (dt, *J* = 7.7, 1.0 Hz, 1H, H10), 6.81 (d, *J* = 7.6 Hz, 1H, H9), 2.63 (ddd, *J* = 14.1, 10.2, 4.1 Hz, 1H, H1), 2.56 (d, *J* = 9.0 Hz, 1H, H2), 2.42 (dq, *J* = 13.3, 2.8 Hz, 1H, H5), 2.09 (ddd, *J* = 13.9, 11.6, 3.8 Hz, 1H, H6), 2.02 – 1.92 (m, 2H, H3, H4), 1.48 – 1.32 (m, 3H, H2, H3, H5), 1.35 – 1.27 (m, 1H, H4), 0.26 (s, 9H, H14) ppm.

^{13}C NMR (151 MHz, $CDCl_3$): δ = 173.8 (C7), 137.5 (C8), 134.5 (C13), 132.7 (C10), 129.1 (C12), 127.6 (C11), 114.8 (C9), 43.5 (C6), 38.1 (C1), 28.8 (C2), 26.3 (C5), 25.41 (C4), 25.38 (C3), –0.8 (C14) ppm.

^{29}Si NMR (119 MHz, $CDCl_3$): δ = –4.08 ppm.

e.r.: 91:9.

cis-diastereomer:

^{1}H NMR (600 MHz, $CDCl_3$): δ = 8.47 (s, 1H, NH), 7.31 (dd, *J* = 7.7, 1.4 Hz, 1H, H10), 7.28 (t, *J* = 0.9 Hz, 1H, H12), 6.77 (d, *J* = 7.7 Hz, 1H, H9), 2.95 (dt, *J* = 10.8, 5.0 Hz, 1H, H1), 2.82 (t, *J* = 4.8 Hz, 1H, H6), 2.41 – 2.34 (m, 1H, H5), 1.77 – 1.73 (m, 1H, H3), 1.67 (dd, *J* = 13.9, 4.0 Hz, 1H, H2), 1.63 – 1.55 (m, 2H, H2, H4), 1.54 – 1.45 (m, 3H, H3, H4, H5), 0.26 (s, 9H, H14) ppm.

^{13}C NMR (151 MHz, $CDCl_3$): δ = 173.2 (C7), 137.0 (C8), 134.8 (C13), 132.6 (C10), 132.4 (C12), 128.1 (C11), 114.9 (C9), 40.8 (C6), 39.4 (C1), 29.8 (C2), 25.4 (C3), 24.7 (C5), 22.7 (C4), –0.9 (C14) ppm.

^{29}Si NMR (119 MHz, $CDCl_3$): δ = –4.37 ppm.

e.r.: 94:6.

HPLC: AD-H column; eluent: *n*-hexane/ *i*-propanol 90:10; flow rate: 1.0 mL/min.

Racemic sample: ***trans*-2f**

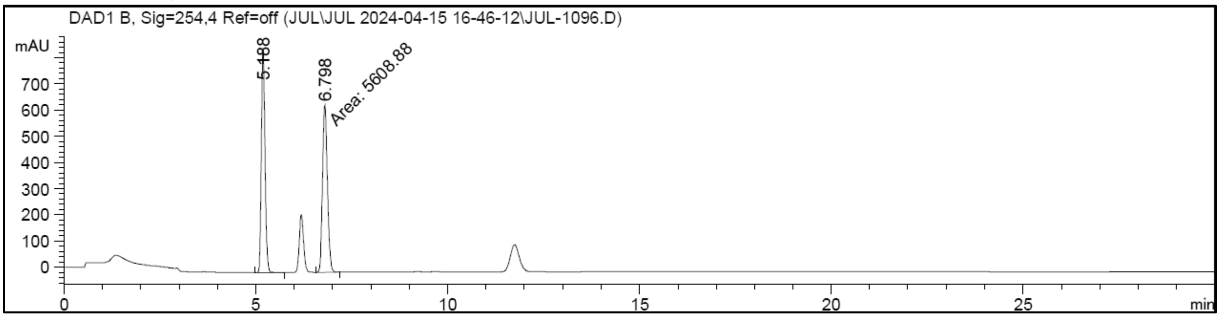

Signal 2: DAD1 B, Sig=254,4 Ref=off

Peak #	RetTime [min]	Type	Width [min]	Area [mAU*s]	Height [mAU]	Area %
1	5.188	BB	0.0998	5573.87988	858.14032	49.8435
2	6.798	MF	0.1467	5608.87891	637.42163	50.1565

Enantioselective sample: ***trans*-2f**

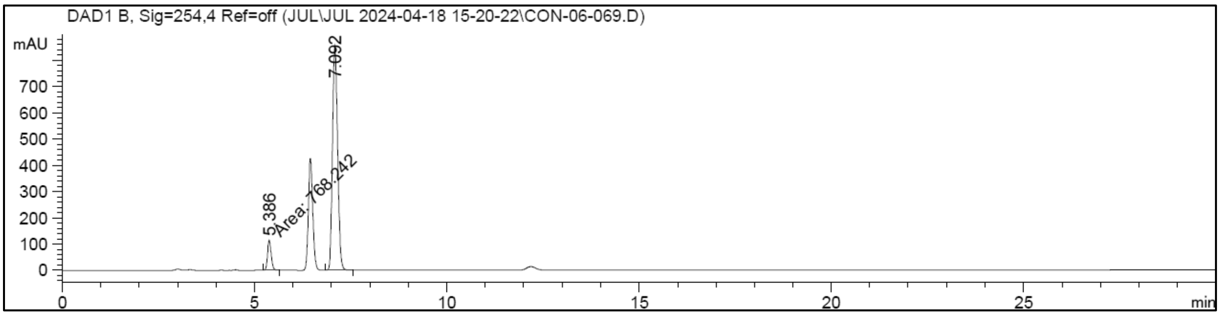

Signal 2: DAD1 B, Sig=254,4 Ref=off

Peak #	RetTime [min]	Type	Width [min]	Area [mAU*s]	Height [mAU]	Area %
1	5.386	FM	0.1119	768.24225	114.41156	8.9706
2	7.092	BB	0.1404	7795.76123	854.96191	91.0294

Racemic sample: ***cis*-2f**

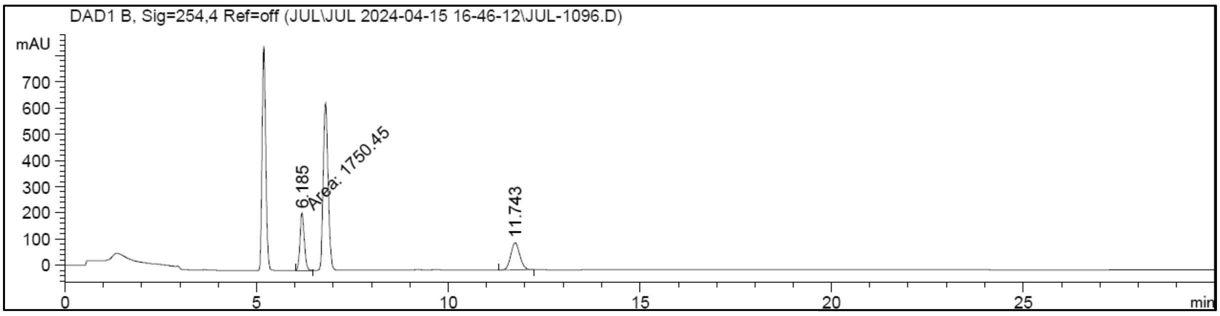

Signal 2: DAD1 B, Sig=254,4 Ref=off

Peak #	RetTime [min]	Type	Width [min]	Area [mAU*s]	Height [mAU]	Area %
1	6.185	FM	0.1323	1750.44678	220.54312	50.8301
2	11.743	BB	0.2524	1693.27295	103.77907	49.1699

Enantioselective sample: ***cis*-2f**

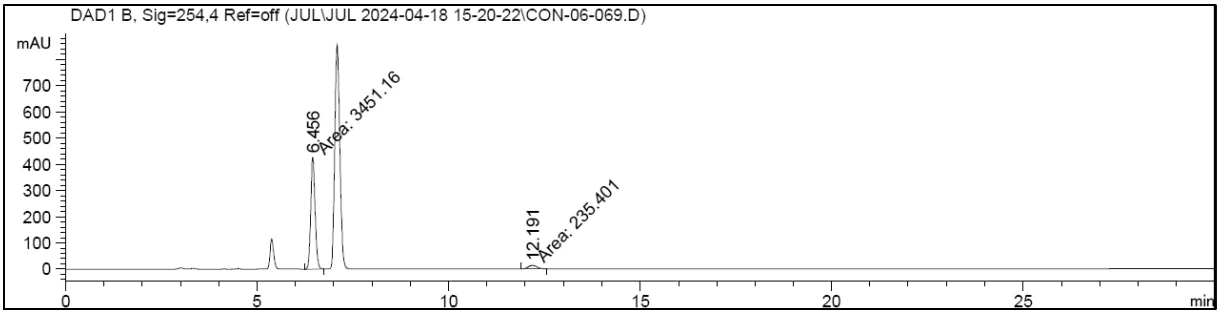

Signal 2: DAD1 B, Sig=254,4 Ref=off

Peak #	RetTime [min]	Type	Width [min]	Area [mAU*s]	Height [mAU]	Area %
1	6.456	MM	0.1347	3451.16235	426.98947	93.6146
2	12.191	MF	0.2831	235.40057	13.85808	6.3854

2-(Triphenylsilyl)-6a,7,8,9,10,10a-hexahydrophenanthridin-6(5H)-one (2g)

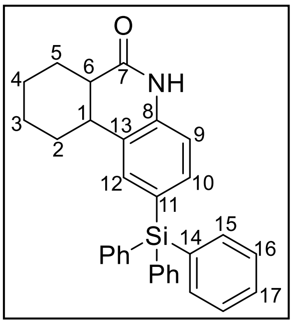

According to General Procedure **E**, **1g** (46.0 mg, 0.10 mmol, 1.00 eq.) was converted to **2g** after being stirred for 68 h yielding the product as white solid (17.5 mg, 38%) after purification by column chromatography (SiO_2, 20% EtOAc in *n*-pentane). The product was obtained as an inseparable mixture of diastereomers (d.r. 56:44 (*trans*:*cis*)).

R*f* = 0.23 (20% EtOAc in *n*-pentane, both diastereomers).

HR-ESI-MS: *m/z*: 482.19106 ($[M+Na]^+$, calcd. for $C_{31}H_{29}NOSiNa^+$: 482.19106).

trans-diastereomer:

^{1}H NMR (500 MHz, $CDCl_3$): δ = 7.77 (s, 1H, NH), 7.58 – 7.54 (m, 6H, H16), 7.46 – 7.42 (m, 4H H12, H17), 7.40 – 7.36 (m, 7H, H10, H15), 6.76 (d, *J* = 7.7 Hz, 1H, H9), 2.65 – 2.56 (m, 1H, H1), 2.41 (s, 1H, H5), 2.26 (dd, *J* = 14.6, 7.7 Hz, 1H, H2), 2.10 (ddd, *J* = 14.3, 11.5, 3.8 Hz, 1H, H6), 1.92 – 1.84 (m, 2H, H3/H4, H3/H4), 1.35 – 1.29 (m, 4H, H2, H3/H4, H3/H4, H5) ppm.

^{13}C NMR (126 MHz, $CDCl_3$): δ = 173.4 (C7), 138.0 (C8), 136.5 (C16), 136.0 (C10), 134.4 (C14), 132.4 (C12), 129.8 (C17), 128.5 (C11), 128.0 (C15), 114.7 (C9), 43.5 (C6), 38.1 (C1), 28.6 (C2), 26.3 (C5), 25.33 (C3/C4), 25.29 (C3/C4) ppm. C13 was not observed.

^{29}Si NMR (99 MHz, $CDCl_3$): δ = –14.35 ppm.

e.r.: 81:19.

cis-diastereomer:

^{1}H NMR (500 MHz, $CDCl_3$): δ = 7.75 (s, 1H, NH), 7.58 – 7.54 (m, 6H, H16), 7.46 –7.42 (m, 3H, H17), 7.41 – 7.35 (m, 6H, H15), 7.35 – 7.33 (m, 2H, H10, H12), 6.72 (d, *J* = 8.1 Hz, 1H, H9), 2.89 (dt, *J* = 10.6, 4.9 Hz, 1H, H1), 2.85 – 2.82 (m, 1H, H6), 2.38 (s, 1H, H5), 1.73 – 1.68 (m, 1H, H3), 1.61 – 1.52 (m, 3H, H2, H4), 1.49 – 1.44 (m, 2H, H4, H5), 1.40 – 1.33 (m, 1H, H3) ppm.

^{13}C NMR (126 MHz, $CDCl_3$): δ = 172.7 (C7), 137.6 (C8), 136.5 (C16), 136.0 (C13), 135.9 (C10/C12), 135.5 (C10/C12), 134.4 (C14), 129.8 (C17), 128.5 (C11), 128.0 (C15), 114.8 (C9), 40.7 (C6), 39.3 (C1), 30.2 (C2), 25.3 (C3), 24.7 (C5), 22.7 (C4) ppm.

^{29}Si NMR (99 MHz, $CDCl_3$): δ = –14.59 ppm.

e.r.: 90:10.

HPLC: NR column; eluent: *n*-hexane/ *i*-propanol 90:10; flow rate: 1.0 mL/min.

Racemic sample: ***trans*-2g**

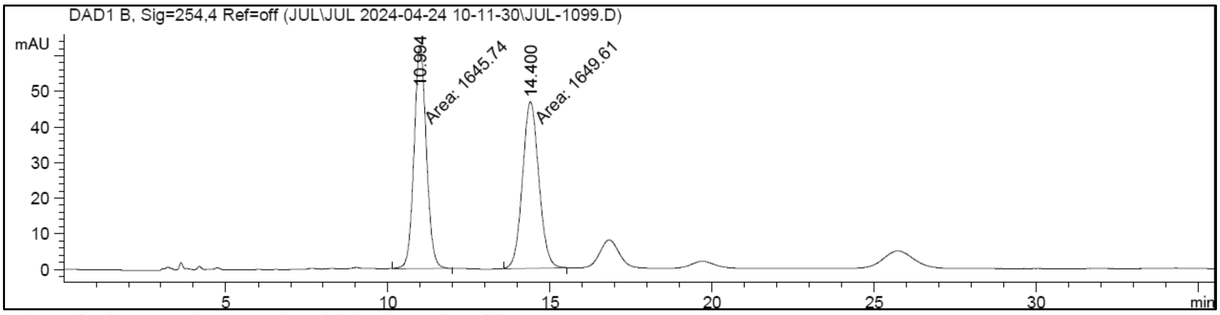

Signal 2: DAD1 B, Sig=254,4 Ref=off

Peak #	RetTime [min]	Type	Width [min]	Area [mAU*s]	Height [mAU]	Area %
1	10.994	FM	0.4391	1645.73938	62.46290	49.9413
2	14.400	MF	0.5873	1649.60547	46.81359	50.0587

Enantioselective sample: ***trans*-2g**

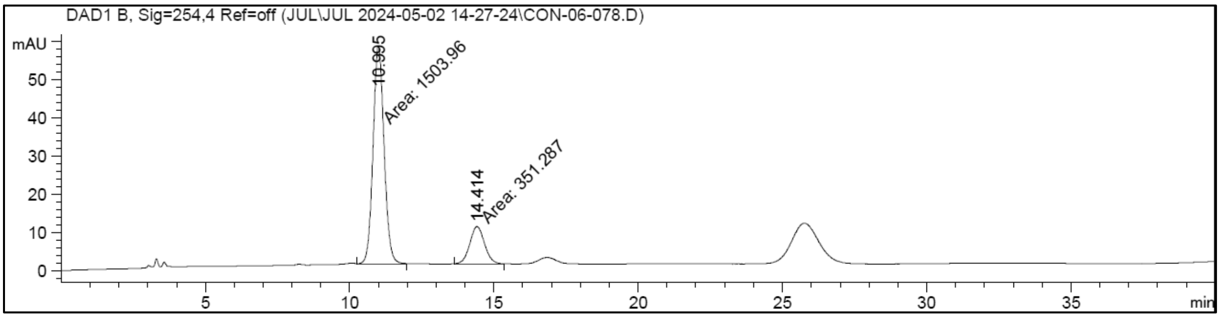

Signal 2: DAD1 B, Sig=254,4 Ref=off

Peak #	RetTime [min]	Type	Width [min]	Area [mAU*s]	Height [mAU]	Area %
1	10.995	MF	0.4405	1503.95850	56.90205	81.0652
2	14.414	FM	0.5971	351.28741	9.80600	18.9348

Racemic sample: ***cis*-2g**

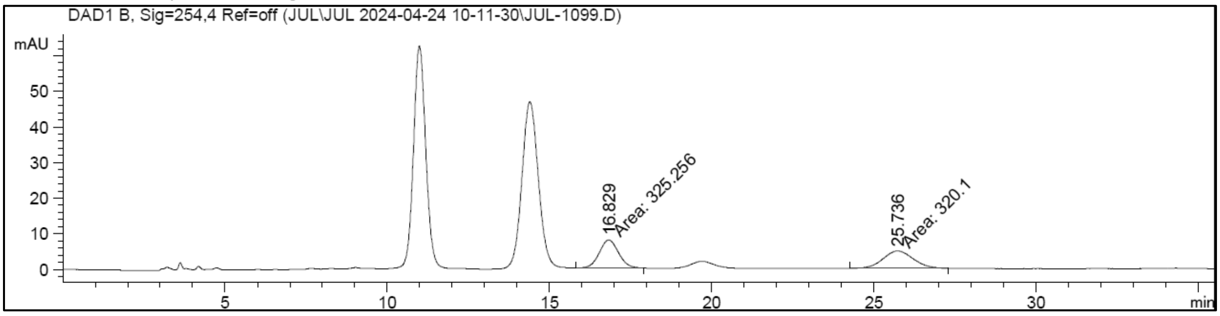

Signal 2: DAD1 B, Sig=254,4 Ref=off

Peak #	RetTime [min]	Type	Width [min]	Area [mAU*s]	Height [mAU]	Area %
1	16.829	MM	0.6896	325.25589	7.86132	50.3994
2	25.736	MM	1.0935	320.10016	4.87870	49.6006

Enantioselective sample: ***cis*-2g**

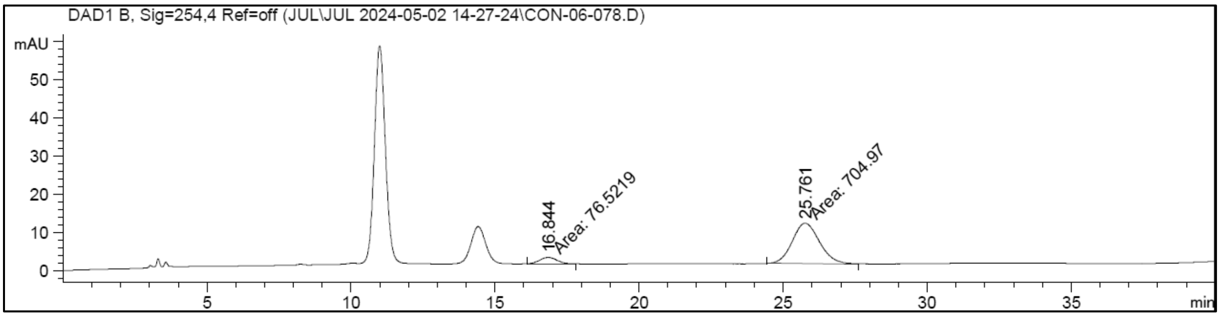

Signal 2: DAD1 B, Sig=254,4 Ref=off

Peak #	RetTime [min]	Type	Width [min]	Area [mAU*s]	Height [mAU]	Area %
1	16.844	MM	0.7285	76.52187	1.75070	9.7918
2	25.761	MM	1.1084	704.96997	10.60029	90.2082

2-(4,4,5,5-Tetramethyl-1,3,2-dioxaborolan-2-yl)-6a,7,8,9,10,10a-hexahydrophenanthridin-6(5H)-one (2h)

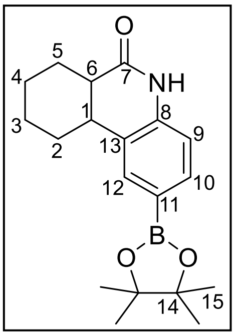

According to General Procedure **E**, **1h** (32.7 mg, 0.10 mmol, 1.00 eq.) was converted to **2h** yielding the product as white solid (21.6 mg, 66%) after purification by column chromatography (SiO_2, 20% EtOAc in *n*-pentane). The product was obtained as an inseparable mixture of diastereomers (d.r. 70:30 (*trans*:*cis*)).

R*f* = 0.23 (20% EtOAc in *n*-pentane, both diastereomers).

HR-ESI-MS: *m/z*: 350.18974 ([*M*+Na]$^+$, calcd. for $C_{19}H_{26}NO_3BNa^+$: 350.19014).

trans-diastereomer:

^{1}H NMR (500 MHz, $CDCl_3$): δ = 8.48 (s, 1H, NH), 7.67 (s, 1H, H12), 7.64 (dt, *J* = 7.7, 0.9 Hz, 1H, H10), 6.79 (d, *J* = 7.7 Hz, 1H, H9), 2.64 – 2.57 (m, 2H, H1, H2), 2.44 – 2.39 (m, 1H, H5), 2.06 (ddd, *J* = 13.5, 11.6, 3.8 Hz, 1H, H6), 1.98 – 1.89 (m, 2H, H3, H4), 1.43 – 1.36 (m, 3H, H2, H3, H5), 1.33 (s, 12H, H15), 1.31 – 1.27 (m, 1H, H4) ppm.

^{13}C NMR (126 MHz, $CDCl_3$): δ = 173.8 (C7), 139.4 (C8), 134.5 (C10), 131.0 (C12), 127.7 (C13), 123.2 (C11), 114.7 (C9), 83.8 (C14), 43.5 (C6), 38.0 (C1), 29.0 (C2), 26.3 (C5), 25.4 (C3), 25.3 (C4), 25.00 (C15), 24.96 (C15') ppm. Boron bearing carbon assigned via 2D NMR.

^{11}B NMR (160 MHz, $CDCl_3$): δ = 30.25 ppm.

e.r.: 90:10.

cis-diastereomer:

^{1}H NMR (500 MHz, $CDCl_3$): δ = 8.48 (s, 1H, NH), 7.63 – 7.58 (m, 2H, H10, H12), 6.76 (d, *J* = 7.9 Hz, 1H, H9), 2.95 (dt, *J* = 10.7, 5.0 Hz, 1H, H1), 2.80 (dd, *J* = 5.0, 4.4 Hz, 1H, H6), 2.36 (s, 1H, H5), 1.73 (dd, *J* = 12.1, 4.0 Hz, 1H, H3), 1.68 (dd, *J* = 13.9, 3.9 Hz, 1H, H2), 1.62 – 1.51 (m, 2H, H2, H4), 1.51 –1.43 (m, 3H, H3, H4, H5), 1.34 (s, 12H, H15) ppm.

^{13}C NMR (126 MHz, $CDCl_3$): δ = 173.3 (C7), 139.0 (C8), 134.4 (C10), 134.1 (C12), 127.9 (C13), 123.8 (C11), 114.8 (C9), 83.8 (C14), 40.8 (C6), 39.1 (C1), 29.9 (C2), 25.3 (C3), 25.0 (C15), 24.9 (C15'), 24.7 (C5), 22.8 (C4) ppm. Boron bearing carbon assigned via 2D NMR.

^{11}B NMR (160 MHz, $CDCl_3$): δ = 30.25 ppm.

e.r.: 93:7.

HPLC: AD-H column; eluent: *n*-hexane/ *i*-propanol 90:10; flow rate: 1.0 mL/min.

Racemic sample: ***trans*-2h**

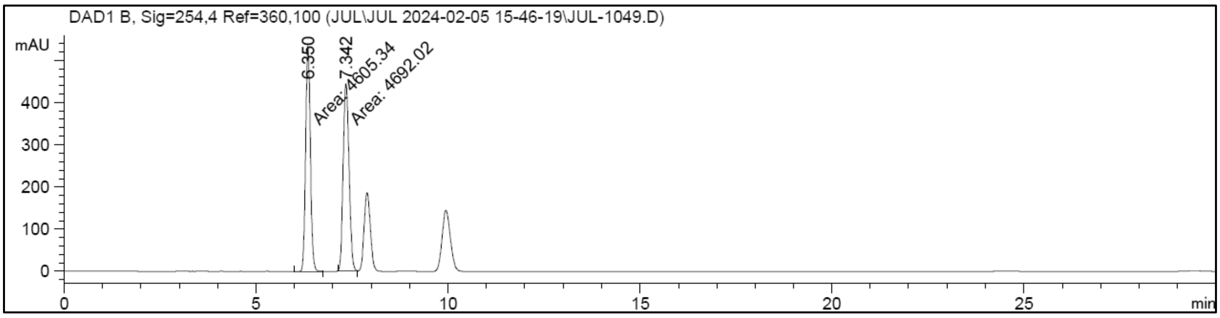

Signal 2: DAD1 B, Sig=254,4 Ref=360,100

Peak #	RetTime [min]	Type	Width [min]	Area [mAU*s]	Height [mAU]	Area %
1	6.350	MF	0.1443	4605.34229	532.00043	49.5339
2	7.342	MM	0.1759	4692.02100	444.46744	50.4661

Enantioselective sample: ***trans*-2h**

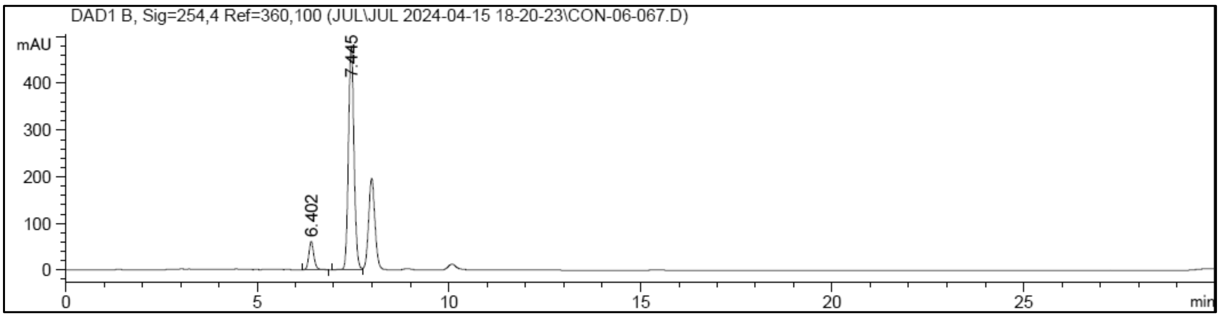

Signal 2: DAD1 B, Sig=254,4 Ref=360,100

Peak #	RetTime [min]	Type	Width [min]	Area [mAU*s]	Height [mAU]	Area %
1	6.402	BB	0.1406	553.25879	60.54416	10.1657
2	7.445	BV	0.1569	4889.13818	479.93210	89.8343

Racemic sample: ***cis*-2h**

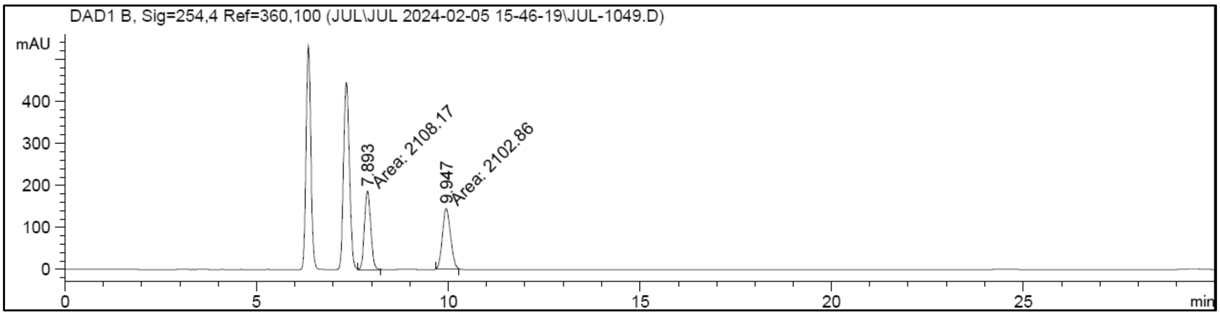

Signal 2: DAD1 B, Sig=254,4 Ref=360,100

Peak #	RetTime [min]	Type	Width [min]	Area [mAU*s]	Height [mAU]	Area %
1	7.893	MF	0.1879	2108.16748	187.01639	50.0630
2	9.947	MM	0.2454	2102.86377	142.83052	49.9370

Enantioselective sample: ***cis*-2h**

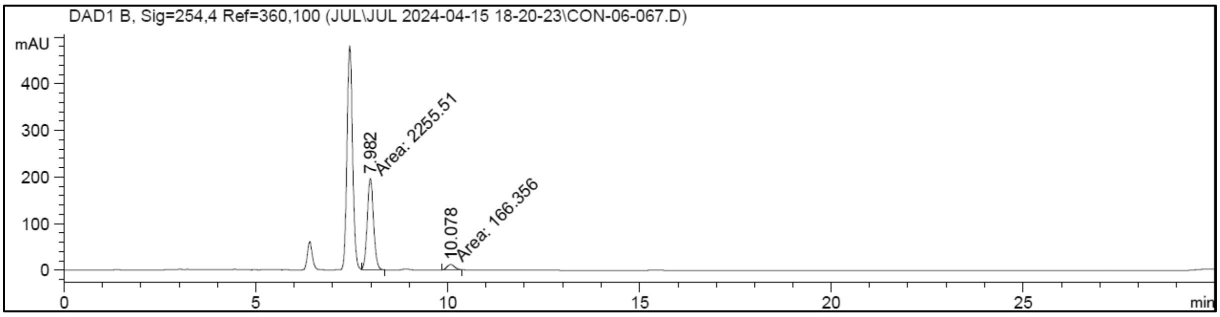

Signal 2: DAD1 B, Sig=254,4 Ref=360,100

Peak #	RetTime [min]	Type	Width [min]	Area [mAU*s]	Height [mAU]	Area %
1	7.982	FM	0.1918	2255.51270	195.99715	93.1311
2	10.078	MM	0.2346	166.35587	11.81686	6.8689

1,2,3,3a,5,9b-Hexahydro-4H-cyclopenta[c]quinolin-4-one (2i)

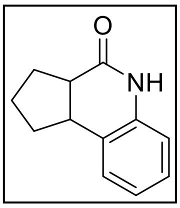

According to General Procedure **D**, **1i** (18.7 mg, 0.10 mmol, 1.00 eq.) was converted to **2i** yielding the product as white solid (10.8 mg, 58%) after purification by column chromatography (SiO_2, 20% EtOAc in *n*-pentane). The product was obtained as an inseparable mixture of diastereomers (d.r. 82:18 (*trans*:*cis*)).

R*f* = 0.17 (20% EtOAc in *n*-pentane, both diastereomers).

^{1}H NMR (400 MHz, $CDCl_3$): δ = 8.73 (s, 1H, cis), 8.40 (s, 1H, trans), 7.24 – 7.09 (m, 4H), 7.01 (dtd, *J* = 12.0, 7.5, 1.2 Hz, 2H), 6.83 (ddd, *J* = 23.4, 7.8, 1.2 Hz, 2H), 3.26 (q, *J* = 8.2 Hz, 1H),

3.05 – 2.81 (m, 2H), 2.42 – 2.24 (m, 3H), 2.19 – 2.03 (m, 3H), 2.03 – 1.81 (m, 3H), 1.81 – 1.60 (m, 4H) ppm.

HR-ESI-MS: *m/z*: 210.08885 ([*M*+Na]$^+$, calcd. for $C_{12}H_{13}NONa^+$: 210.08894).

Analytical data in agreement with literature.[7]

e.r. *(trans):* 84:16.

e.r. *(cis):* 76:24.

HPLC: AS-H column; eluent: *n*-hexane/ *i*-propanol 20:80; flow rate: 1.0 mL/min.

Racemic sample: ***trans*-2i**

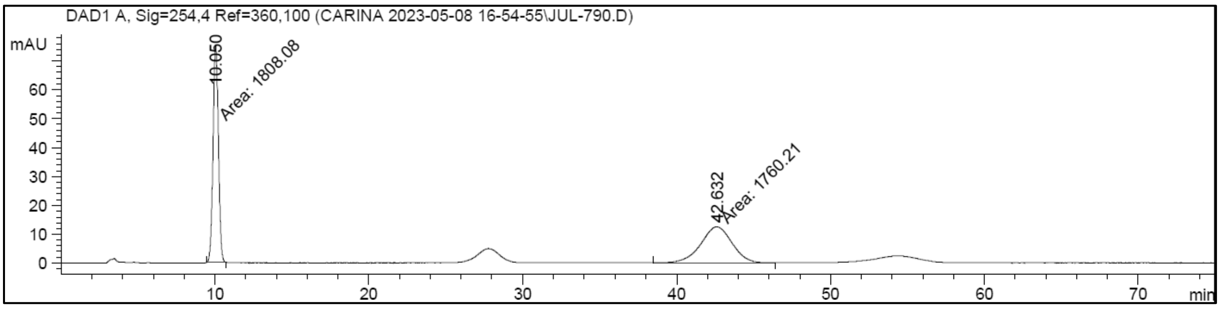

Signal 1: DAD1 A, Sig=254,4 Ref=360,100

Peak #	RetTime [min]	Type	Width [min]	Area [mAU*s]	Height [mAU]	Area %
1	10.050	MM	0.4011	1808.08337	75.12605	50.6709
2	42.632	MM	2.3589	1760.20667	12.43685	49.3291

Enantioselective sample: ***trans*-2i**

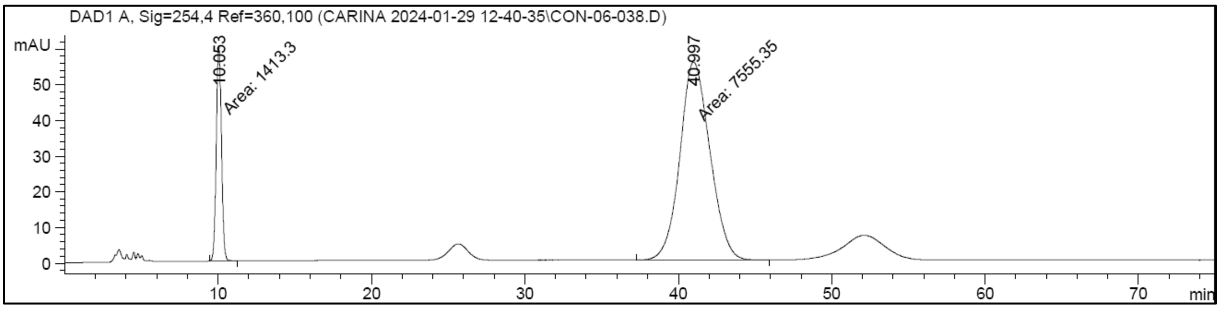

Signal 1: DAD1 A, Sig=254,4 Ref=360,100

Peak #	RetTime [min]	Type	Width [min]	Area [mAU*s]	Height [mAU]	Area %
1	10.053	MM	0.3908	1413.30493	60.27538	15.7583
2	40.997	MM	2.2707	7555.34717	55.45654	84.2417

Racemic sample: ***cis*-2i**

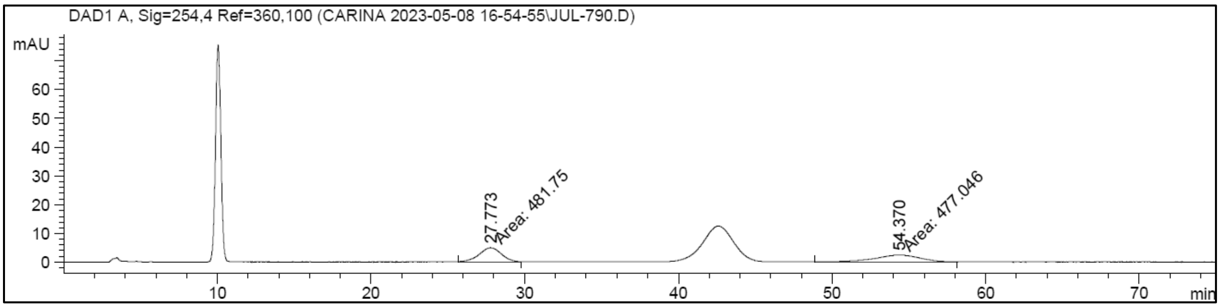

Signal 1: DAD1 A, Sig=254,4 Ref=360,100

Peak #	RetTime [min]	Type	Width [min]	Area [mAU*s]	Height [mAU]	Area %
1	27.773	MM	1.6472	481.75043	4.87434	50.2453
2	54.370	MM	3.3590	477.04639	2.36700	49.7547

Enantioselective sample: ***cis*-2i**

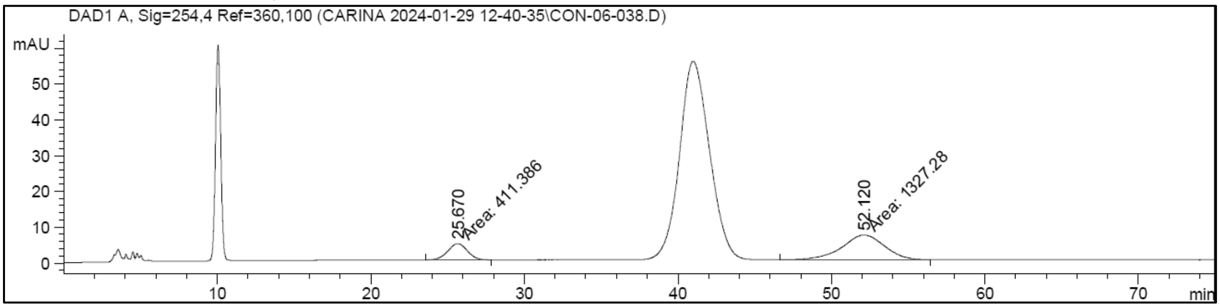

Signal 1: DAD1 A, Sig=254,4 Ref=360,100

Peak #	RetTime [min]	Type	Width [min]	Area [mAU*s]	Height [mAU]	Area %
1	25.670	MM	1.4958	411.38602	4.58391	23.6610
2	52.120	MM	3.2065	1327.28296	6.89888	76.3390

2,2a,4,8b-Tetrahydrocyclobuta[c]quinolin-3(1H)-one (2j)

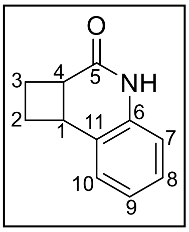

According to General Procedure **D**, **1j** (18.7 mg, 0.10 mmol, 1.00 eq.) was converted to **2j** after 65 h reaction time using HFIP instead of *n*Bu_4NCl yielding the product as white solid (13.1 mg, 76%) after purification by column chromatography (SiO_2, 20% EtOAc in *n*-pentane). The product was obtained as a mixture of diastereomers (d.r. 57:43 (*trans*:*cis*)).

trans-diastereomer:

R*f* = 0.17 (20% EtOAc in *n*-pentane).

^{1}H NMR (500 MHz, $CDCl_3$): δ = 7.65 (s, 1H, NH), 7.16 – 7.10 (m, 2H, H9, H10), 6.99 (td, *J* = 7.5, 1.1 Hz, 1H, H8), 6.81 (dd, *J* = 7.8, 1.1 Hz, 1H, H7), 3.21 (ddd, *J* = 15.4, 10.3, 6.6 Hz, 1H, H1), 2.51

(dtd, *J* = 15.1, 6.6, 3.7 Hz, 1H, H4), 2.33 – 2.24 (m, 2H, H2, H3), 2.20 (dt, *J* = 8.9, 6.8 Hz, 1H, H3), 2.15 – 2.07 (m, 1H, H2) ppm.

^{13}C NMR (126 MHz, $CDCl_3$): δ = 173.1 (C5), 138.7 (C6), 132.0 (C11), 126.9 (C9), 122.9 (C8), 122.8 (C10), 116.1 (C7), 41.9 (C4), 40.4 (C1), 24.7 (C2), 23.9 (C3) ppm.

IR (ATR): $\tilde{\nu}$ = 3214 (m), 3156 (w), 3088 (w), 3025 (m), 2973 (w), 2939 (m), 2870 (w), 1682 (s), 1613 (m), 1469 (m), 1446 (w), 1383 (m), 1369 (w), 1286 (w), 1269 (m), 1234 (m), 1177 (w), 1102 (w), 1067 (w), 1033 (w), 907 (w), 867 (w), 804 (w), 752 (m), 666 (w), 631 (w) cm^{-1}.

HR-ESI-MS: *m/z*: 196.07329 ([*M*+Na]$^+$, calcd. for $C_{11}H_{11}NONa^+$: 196.07329).

Mp = 185 – 187 °C.

e.r.: 73:27.

HPLC: AD-H column; eluent: *n*-hexane/ *i*-propanol 95:5; flow rate: 1.0 mL/min.

Racemic sample: ***trans*-2j**

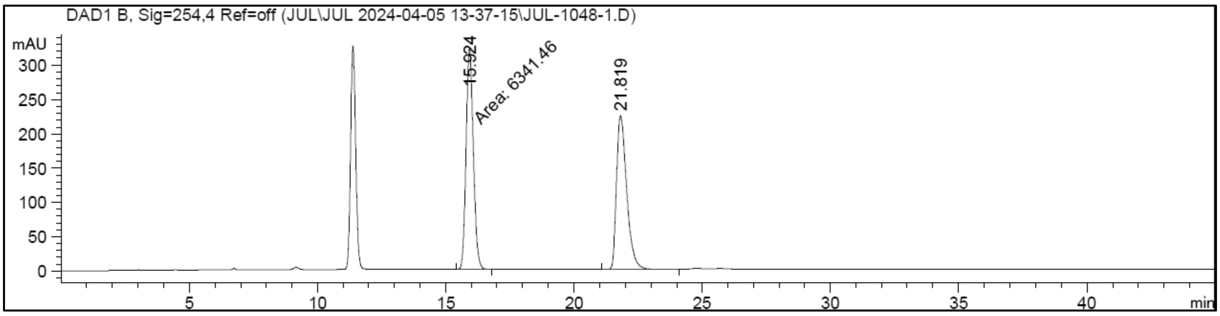

Signal 2: DAD1 B, Sig=254,4 Ref=off

Peak #	RetTime [min]	Type	Width [min]	Area [mAU*s]	Height [mAU]	Area %
1	15.924	MF	0.3280	6341.46484	322.25348	50.6462
2	21.819	BB	0.4233	6179.64697	224.36108	49.3538

Enantioselective sample: ***trans*-2j**

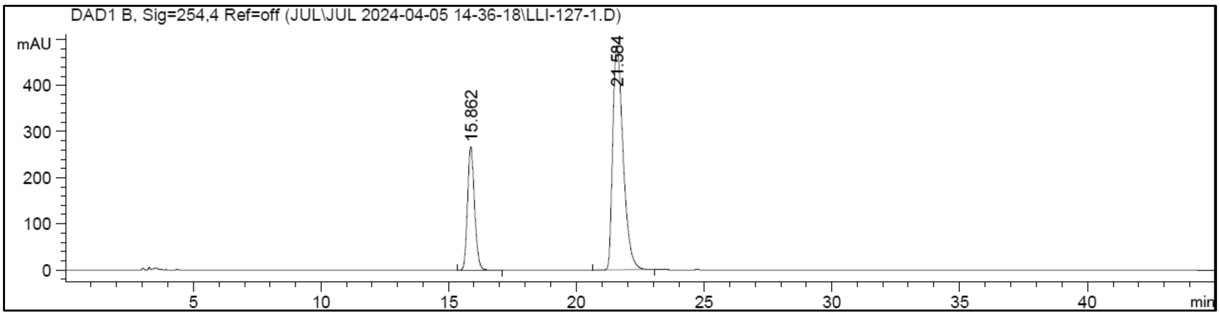

Signal 2: DAD1 B, Sig=254,4 Ref=off

Peak #	RetTime [min]	Type	Width [min]	Area [mAU*s]	Height [mAU]	Area %
1	15.862	BB	0.2930	5071.40234	267.74329	26.9229
2	21.584	BB	0.4345	1.37654e4	485.87631	73.0771

cis-diastereomer:

R$_f$ = 0.10 (20% EtOAc in *n*-pentane).

^{1}H NMR (500 MHz, $CDCl_3$): δ = 8.41 (s, 1H, NH), 7.14 (tdd, *J* = 7.9, 1.6, 0.6 Hz, 1H, H9), 7.05 (ddd, *J* = 7.5, 1.6, 0.8 Hz, 1H, H10), 6.98 (td, *J* = 7.4, 1.2 Hz, 1H, H8), 6.74 (ddt, *J* = 7.9, 1.0, 0.5 Hz, 1H, H7), 3.82 – 3.76 (m, 1H, H1), 3.41 – 3.35 (m, 1H, H4), 2.63 – 2.50 (m, 2H, H2, H3), 2.48 – 2.41 (m, 1H, H3), 2.26 – 2.18 (m, 1H, H2) ppm.

^{13}C NMR (126 MHz, $CDCl_3$): δ = 172.7 (C5), 136.7 (C6), 128.0 (C10), 127.8 (C9), 125.2 (C11), 123.6 (C8), 115.7 (C7), 38.2 (C4), 36.5 (C1), 30.3 (C2), 26.6 (C3) ppm.

IR (ATR): $\tilde{\nu}$ = 752 (w), 789 (w), 887 (w), 925 (w), 1051 (w), 1103 (w), 1131 (w),1194 (w), 1246 (w), 1297 (w), 1332 (w), 1383 (w), 1435 (w), 1492 (w), 1596 (w), 1670 (s), 2864 (w), 2939 (w), 2985 (w), 3065 (w), 3203 (w) ppm.

HR-ESI-MS: *m/z*: 196.07325 ([*M*+Na]$^+$, calcd. for $C_{11}H_{11}NONa^+$: 196.07329).

Mp = 155 – 154 °C.

e.r.: 69:31.

HPLC: AD-H column; eluent: *n*-hexane/ *i*-propanol 95:5; flow rate: 1.0 mL/min.

Racemic sample: ***cis*-2j**

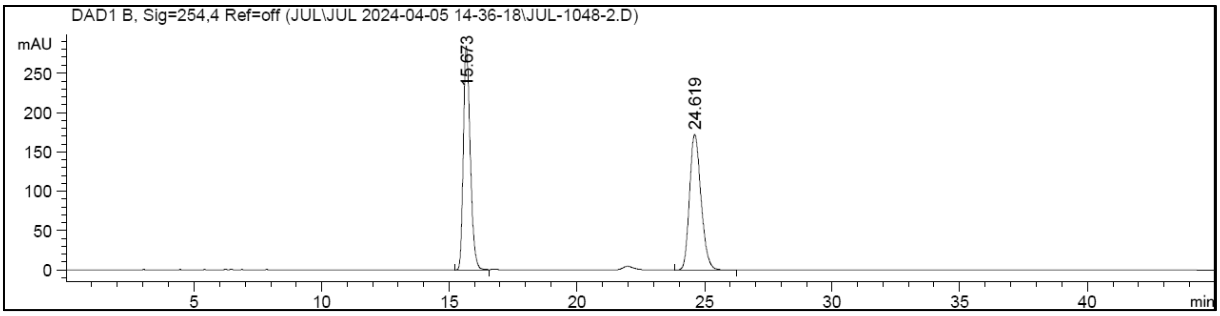

Signal 2: DAD1 B, Sig=254,4 Ref=off

Peak #	RetTime [min]	Type	Width [min]	Area [mAU*s]	Height [mAU]	Area %
1	15.673	BB	0.2894	5343.18994	284.18954	49.8026
2	24.619	BB	0.4790	5385.55518	172.12489	50.1974

Enantioselective sample: ***cis*-2j**

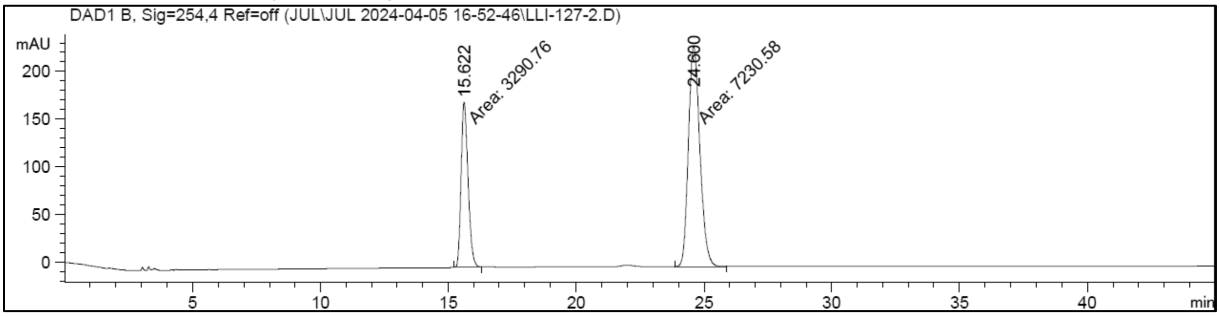

Signal 2: DAD1 B, Sig=254,4 Ref=off

Peak #	RetTime [min]	Type	Width [min]	Area [mAU*s]	Height [mAU]	Area %
1	15.622	MM	0.3160	3290.75610	173.58063	31.2770
2	24.600	MM	0.5210	7230.58350	231.32188	68.7230

Enantioselective sample: ***cis*-2j** after recrystallization

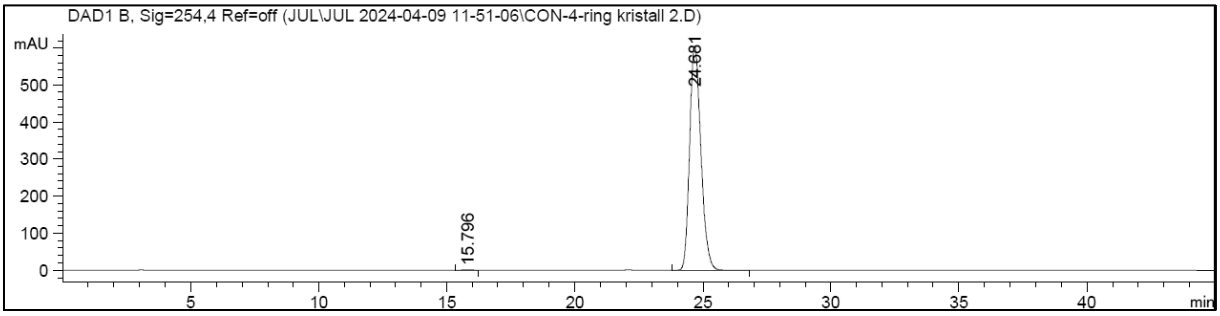

Signal 2: DAD1 B, Sig=254,4 Ref=off

Peak #	RetTime [min]	Type	Width [min]	Area [mAU*s]	Height [mAU]	Area %
1	15.796	BB	0.3311	31.11515	1.40080	0.1672
2	24.681	BB	0.4817	1.85786e4	605.90741	99.8328

10a-Methyl-6a,7,8,9,10,10a-hexahydrophenanthridin-6(5H)-one (2k)

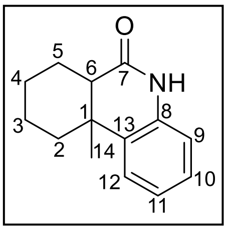

According to General Procedure **D**, **1k** (21.5 mg, 0.10 mmol, 1.00 eq.) was converted to **2k** using HFIP instead of *n*Bu_4NCl yielding the product after 65 h reaction time as a white solid (20.1 mg, 93%) after purification by column chromatography (SiO_2, 10-20% EtOAc in *n*-pentane). The product was obtained as an inseparable mixture of diastereomers (d.r. 81:19 (*trans*:*cis*)). The relative configuration was assigned via x-ray crystallography (vide infra). The e.r. of the *cis*-diastereomer could not be determined due to the lack of a racemic sample.

trans-diastereomer:

R*f* = 0.32 (20% EtOAc in *n*-pentane).

^{1}H NMR (500 MHz, $CDCl_3$): δ = 7.86 (s, 1H, NH), 7.23 (dd, *J* = 7.6, 1.4 Hz, 1H, H12), 7.17 (td, *J* = 7.6, 1.5 Hz, 1H, H10), 7.05 (td, *J* = 7.6, 1.2 Hz, 1H, H11), 6.76 (dd, *J* = 7.8, 1.2 Hz, 1H, H9), 2.32 (dd, *J* = 12.4, 3.7 Hz, 1H, H6), 2.26 – 2.20 (m, 1H, H2), 2.19 – 2.12 (m, 1H, H5), 1.96 – 1.90 (m, 1H, H4), 1.81 – 1.72 (m, 1H, H3), 1.64 – 1.50 (m, 3H, H2, H3, H5), 1.31 – 1.24 (m, 1H, H4), 1.09 (s, 3H, H14) ppm.

^{13}C NMR (126 MHz, $CDCl_3$): δ = 172.7 (C7), 135.9 (C13), 135.6 (C8), 127.3 (C10), 123.7 (C12), 123.5 (C11), 115.8 (C9), 47.0 (C6), 36.1 (C1), 35.6 (C2), 25.5 (C4), 21.4 (C3), 21.1 (C5), 19.2 (C14) ppm.

IR (ATR): $\tilde{\nu}$ = 620 (m), 654 (s), 746 (s), 809 (s), 861 (m), 901 (w), 930 (w), 987 (w), 1039 (m), 1085 (m), 1154 (w), 1217 (m), 1251 (m), 1280 (m), 1332 (s), 1383 (s), 1441 (m), 1487 (s), 1590 (m), 1607 (m), 1670 (s), 2858 (m), 2921 (s), 3065 (w), 3191 (w) cm^{-1}.

HR-ESI-MS: *m/z*: 238.12011 ($[M+Na]^+$, calcd. for $C_{14}H_{17}NONa^+$: 238.12024).

Mp = 142 – 143 °C.

e.r.: 75:25.

$[\alpha]_D^{22\,°C}$ = +69.7° (*c* = 0.2 in $CHCl_3$, for *e.r.* 84:16).

HPLC: AS-H column; eluent: *n*-hexane/ *i*-propanol 15:85; flow rate: 1.0 mL/min.

Racemic sample: ***trans*-2k**

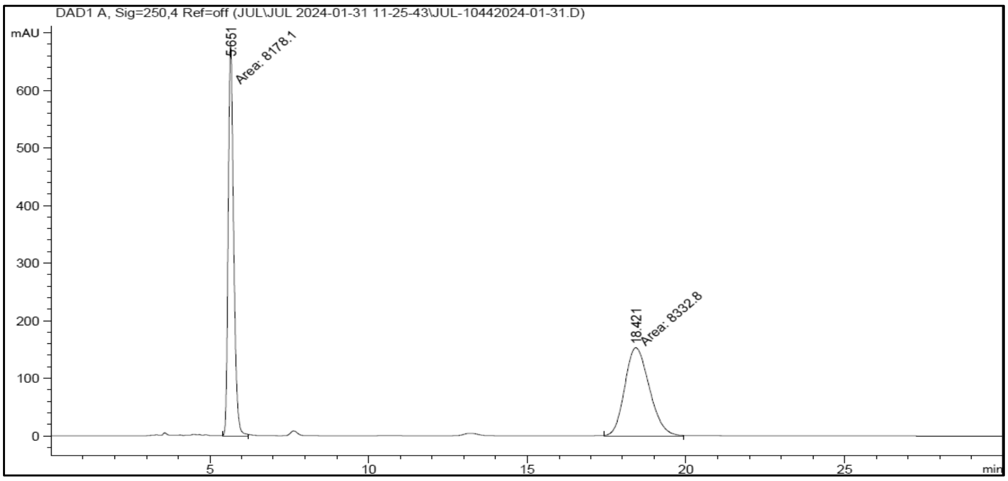

Signal 1: DAD1 A, Sig=250,4 Ref=off

Peak #	RetTime [min]	Type	Width [min]	Area [mAU*s]	Height [mAU]	Area %
1	5.651	MM	0.2010	8178.10303	678.28528	49.5315
2	18.421	MM	0.9042	8332.80273	153.59338	50.4685

Enantioselective sample: ***trans*-2k**

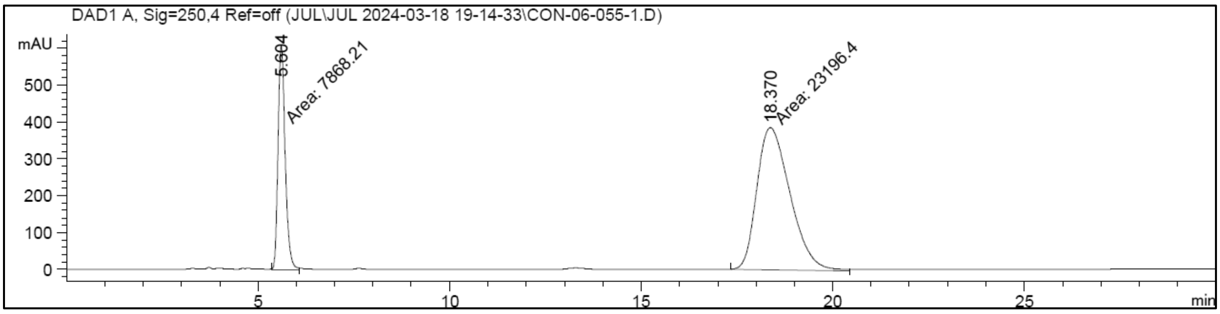

Signal 1: DAD1 A, Sig=250,4 Ref=off

Peak #	RetTime [min]	Type	Width [min]	Area [mAU*s]	Height [mAU]	Area %
1	5.604	MM	0.2154	7868.21191	608.84595	25.3285
2	18.370	MM	1.0029	2.31964e4	385.50754	74.6715

2-Methoxy-6a,7,8,9,10,10a-hexahydrophenanthridin-6(5H)-one (2l)

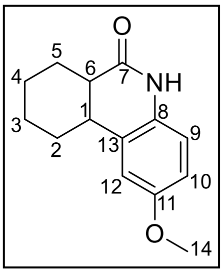

According to General Procedure **D**, **1l** (23.1 mg, 0.10 mmol, 1.00 eq.) was converted to **2l** yielding the product as white solid (14.5 mg, 63%) after purification by column chromatography (SiO_2, 40% EtOAc in *n*-pentane). The product was obtained as an inseparable mixture of diastereomers (d.r. 66:34 (*trans*:*cis*)).

R*f* = 0.21 (30% EtOAc in *n*-pentane, both diastereomers).

HR-ESI-MS: *m/z*: 254.11508 ($[M+Na]^+$, calcd. for $C_{14}H_{17}NO_2Na^+$: 254.11515).

trans-diastereomer:

^{1}H NMR (500 MHz, $CDCl_3$): δ = 8.49 (s, 1H, NH), 6.81 (t, *J* = 1.8 Hz, 1H, H12), 6.75 – 6.68 (m, 2H, H9, H10), 3.79 (s, 3H, H14), 2.58 (ddd, *J* = 14.3, 10.6, 4.0 Hz, 1H, H1), 2.48 – 2.37 (m, 2H, H2, H5), 2.03 (ddd, *J* = 14.1, 11.6, 3.7 Hz, 1H, H6), 1.98 – 1.89 (m, 2H, H3, H4), 1.41 – 1.33 (m, 3H, H2, H3, H5), 1.33 – 1.28 (m, 1H, H4) ppm.

^{13}C NMR (126 MHz, $CDCl_3$): δ = 173.4 (C7), 155.9 (C11), 130.4 (C13), 130.1 (C8), 116.0 (C9), 111.7 (C10), 111.3 (C12), 55.7 (C14), 43.2 (C6), 38.2 (C1), 28.9 (C2), 26.3 (C5), 25.4 (2C, C3, C4) ppm.

e.r.: 86:14.

cis-diastereomer:

^{1}H NMR (500 MHz, $CDCl_3$): δ = 8.52 (s, 1H, NH), 6.75 – 6.68 (m, 3H, H9, H10, H12), 3.78 (s, 3H, H14), 2.91 (dt, *J* = 10.2, 5.1 Hz, 1H, H1), 2.76 (dd, *J* = 4.8, 4.5 Hz, 1H, H6), 2.35 – 2.26 (m, 1H, H5), 1.72 (dd, *J* = 12.3, 4.5 Hz, 1H, H3), 1.68 – 1.62 (m, 1H, H2), 1.62 – 1.55 (m, 2H, H2, H4), 1.52 – 1.47 (m, 2H, H4, H5), 1.42 (t, *J* = 3.3 Hz, 1H, H3) ppm.

^{13}C NMR (126 MHz, $CDCl_3$): δ = 172.8 (C7), 155.9 (C11), 130.0 (2C, C8, C13), 116.2 (C9), 113.3 (C12), 112.3 (C10), 55.7 (C14), 40.7 (C6), 39.3 (C1), 29.6 (C2), 25.1 (C3), 24.6 (C5), 22.9 (C4) ppm.

e.r.: 84:16.

HPLC: AS-H column; eluent: *n*-hexane/ *i*-propanol 20:80; flow rate: 1.0 mL/min.

Racemic sample: ***trans*-2l**

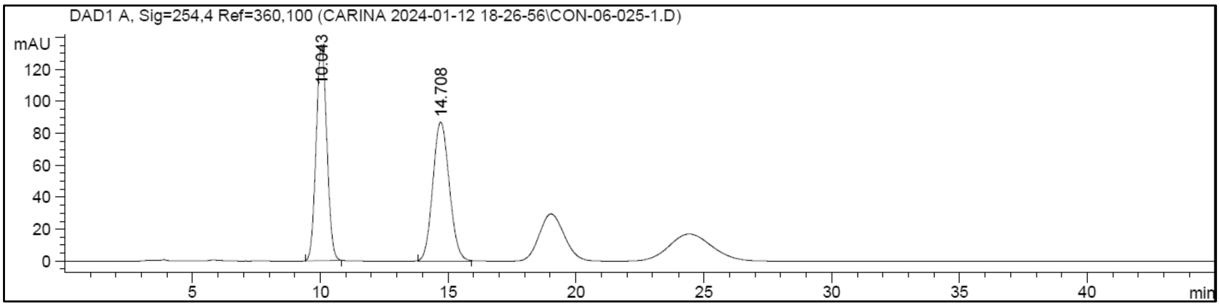

Signal 1: DAD1 A, Sig=254,4 Ref=360,100

Peak #	RetTime [min]	Type	Width [min]	Area [mAU*s]	Height [mAU]	Area %
1	10.043	BB	0.4508	3897.92920	135.03870	50.0258
2	14.708	BB	0.6995	3893.91016	86.78307	49.9742

Enantioselective sample: ***trans*-2l**

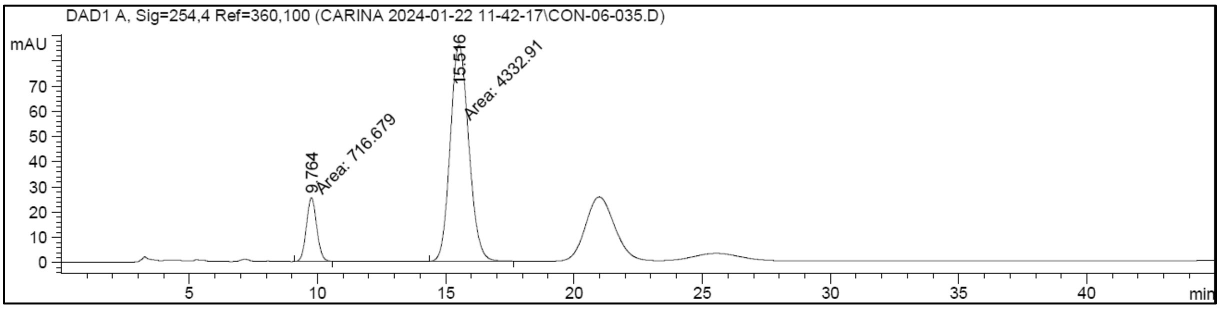

Signal 1: DAD1 A, Sig=254,4 Ref=360,100

Peak #	RetTime [min]	Type	Width [min]	Area [mAU*s]	Height [mAU]	Area %
1	9.764	MM	0.4716	716.67902	25.32714	14.1928
2	15.516	MM	0.8400	4332.90723	85.96744	85.8072

Racemic sample: ***cis*-2l**

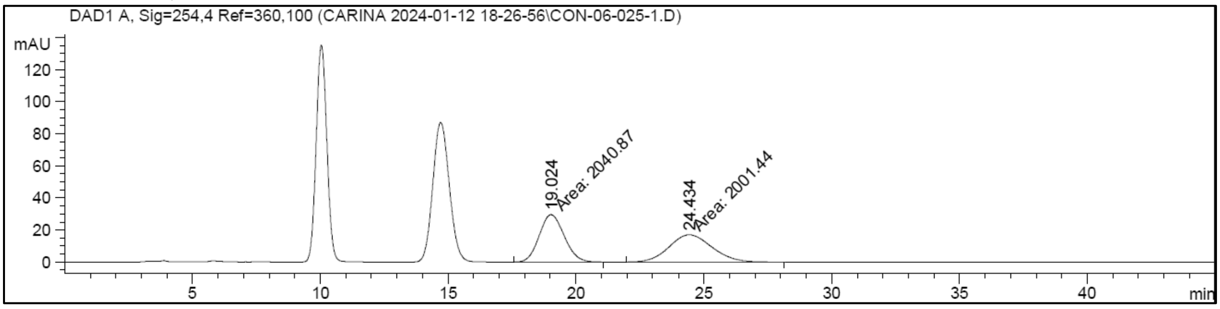

Signal 1: DAD1 A, Sig=254,4 Ref=360,100

Peak #	RetTime [min]	Type	Width [min]	Area [mAU*s]	Height [mAU]	Area %
1	19.024	MM	1.1450	2040.87048	29.70636	50.4877
2	24.434	MM	1.9587	2001.44336	17.03055	49.5123

Enantioselective sample: ***cis*-2l**

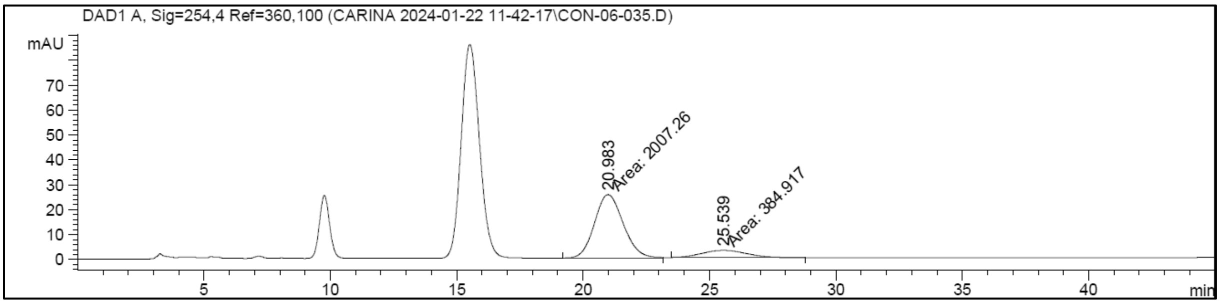

Signal 1: DAD1 A, Sig=254,4 Ref=360,100

Peak #	RetTime [min]	Type	Width [min]	Area [mAU*s]	Height [mAU]	Area %
1	20.983	MM	1.3134	2007.25708	25.47122	83.9093
2	25.539	MM	2.1182	384.91748	3.02872	16.0907

2-Methoxy-5-methyl-6a,7,8,9,10,10a-hexahydrophenanthridin-6(5H)-one (2m)

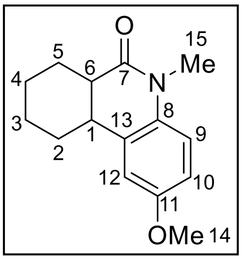

According to General Procedure **F**, **1m** (24.5 mg, 0.10 mmol, 1.00 eq.) was converted to **2m** yielding the product as stinky white solid (23.7 mg, 97%) after purification by column chromatography (SiO_2, 20% EtOAc in *n*-pentane). The product was obtained as a mixture of diastereomers (d.r. 82:18 (*trans*:*cis*)).

trans-diastereomer:

R*f* = 0.32 (20% EtOAc in *n*-pentane).

^{1}H NMR (600 MHz, $CDCl_3$): δ = 6.90 (d, *J* = 8.7 Hz, 1H, H9), 6.82 (dd, *J* = 2.9, 1.2 Hz, 1H, H12), 6.78 (ddd, *J* = 8.7, 2.9, 0.9 Hz, 1H, H10), 3.80 (s, 3H, H14), 3.34 (s, 3H, H15), 2.52 (ddd, *J* = 14.4, 10.4, 4.2 Hz, 1H, H1), 2.43 – 2.37 (m, 2H, H2, H5), 1.97 (ddd, *J* = 13.6, 11.6, 3.8 Hz, 1H, H6), 1.96 – 1.89 (m, 2H, H3, H4), 1.41 – 1.32 (m, 3H, H2, H3, H5), 1.30 – 1.21 (m, 1H, H4) ppm.

^{13}C NMR (151 MHz, $CDCl_3$): δ = 172.1 (C7), 155.7 (C11), 133.9 (C8), 132.4 (C13), 115.3 (C9), 111.2 (C12), 111.0 (C10), 55.7 (C14), 43.6 (C6), 37.3 (C1), 30.1 (C15), 29.0 (C2), 27.1 (C5), 25.34 (C4), 25.29 (C3) ppm.

IR (ATR): $\tilde{\nu}$ = 660(w), 809 (w), 849 (w), 947 (w), 1039 (m), 1062 (m), 1136 (m), 1165 (m), 1240 (s), 1297 (s), 1378 (m), 1429 (m), 1452 (m), 1504 (s), 1590 (w), 1665 (s), 2858 (m), 2927 (s) cm^{-1}.

HR-ESI-MS: *m/z*: 268.13063 ($[M+Na]^+$, calcd. for $C_{15}H_{19}NO_2Na^+$: 268.13080).

e.r.: 96:4.

$[\alpha]_D^{21\,°C}$ = +110° (*c* = 1.0 in $CHCl_3$, for *e.r.* 96:4).

HPLC: OD-H column; eluent: *n*-hexane/ *i*-propanol 97:3; flow rate: 1.0 mL/min.

Racemic sample: ***trans*-2m**

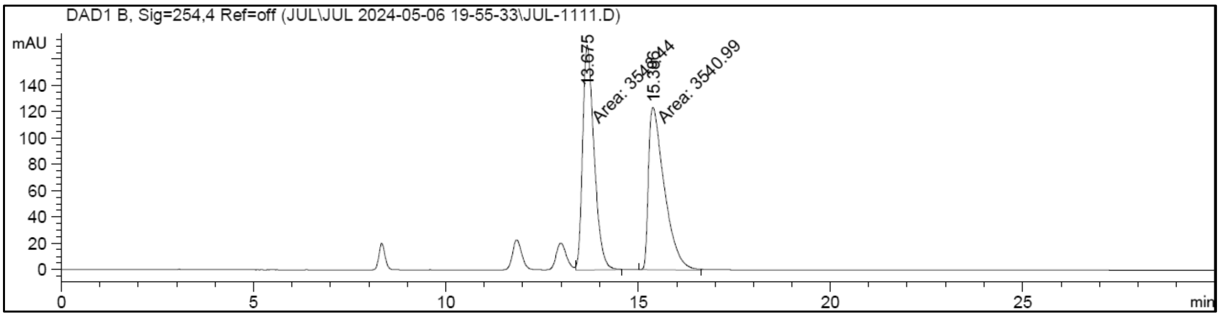

Signal 2: DAD1 B, Sig=254,4 Ref=off

Peak #	RetTime [min]	Type	Width [min]	Area [mAU*s]	Height [mAU]	Area %
1	13.675	MF	0.3456	3543.44092	170.87595	50.0173
2	15.386	MF	0.4775	3540.98804	123.59720	49.9827

Enantioselective sample: ***trans*-2m**

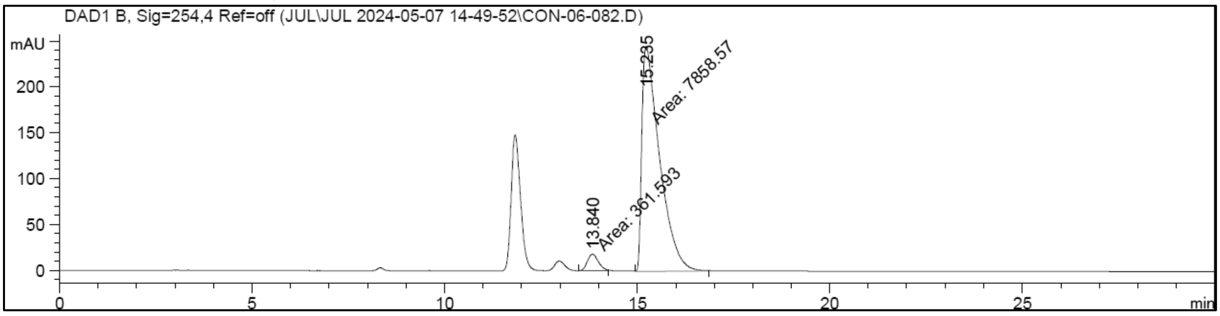

Signal 2: DAD1 B, Sig=254,4 Ref=off

Peak #	RetTime [min]	Type	Width [min]	Area [mAU*s]	Height [mAU]	Area %
1	13.840	MF	0.3326	361.59253	18.11883	4.3988
2	15.235	MM	0.5337	7858.57227	245.41508	95.6012

cis-diastereomer:

R*f* = 0.38 (20% EtOAc in *n*-pentane).

^{1}H NMR (600 MHz, $CDCl_3$), selected signals: δ = 6.87 (d, *J* = 8.7 Hz, 1H, $H_{Arom.}$), 6.76 – 6.74 (m, 1H, $H_{Arom.}$), 6.74 – 6.72 (m, 1H, $H_{Arom.}$), 3.79 (s, 3H, H14), 3.34 (s, 3H, H15), 2.87 – 2.82 (m, 1H, $H_{Aliph.}$), 2.74 (t, *J* = 4.5 Hz, 1H, $H_{Aliph.}$), 2.30 (s, 1H, H_{Aliph}), 1.73 – 1.70 (m, 2H, $H_{Aliph.}$), 1.67 – 1.63 (m, 1H, $H_{Aliph.}$), 1.58 – 1.51 (m, 2H, $H_{Aliph.}$), 1.50 – 1.43 (m, 2H, $H_{Aliph.}$) ppm.

^{13}C NMR (151 MHz, $CDCl_3$), selected signals: δ = 171.1 (C7), 155.5 (C11), 133.4 ($C_{Arom.}$), 115.6 ($C_{Arom.}$), 113.6 ($C_{Arom.}$), 111.7 ($C_{Arom.}$), 55.7 (C14), 41.0 ($C_{Aliph.}$), 33.1 ($C_{Aliph.}$), 29.8 (C15), 22.7 ($C_{Aliph.}$), 21.3 ($C_{Aliph.}$) ppm.

HR-ESI-MS: *m/z*: 268.13063 ([*M*+Na]$^+$, calcd. for $C_{15}H_{19}NO_2Na^+$: 268.13080).

e.r.: 93:7.

HPLC: OD-H column; eluent: *n*-hexane/ *i*-propanol 97:3; flow rate: 1.0 mL/min.

Racemic sample: ***cis*-2m**

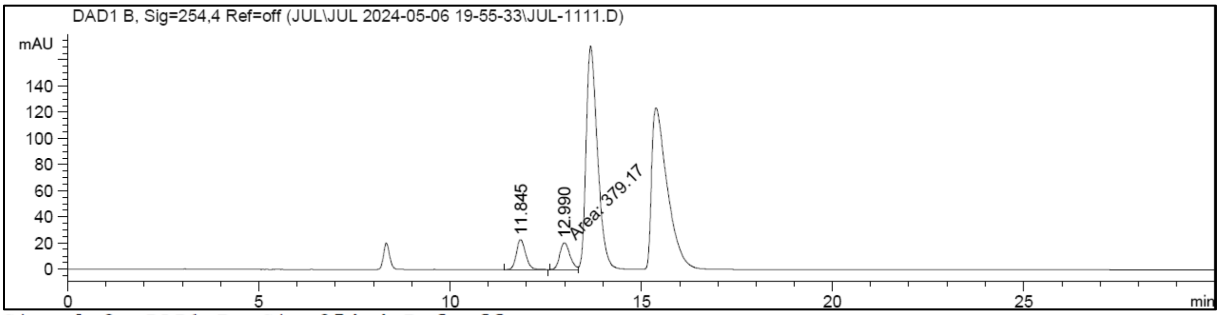

Signal 2: DAD1 B, Sig=254,4 Ref=off

Peak #	RetTime [min]	Type	Width [min]	Area [mAU*s]	Height [mAU]	Area %
1	11.845	BB	0.2581	384.32111	22.86565	50.3374
2	12.990	MF	0.3091	379.16959	20.44642	49.6626

Enantioselective sample: ***cis*-2m**

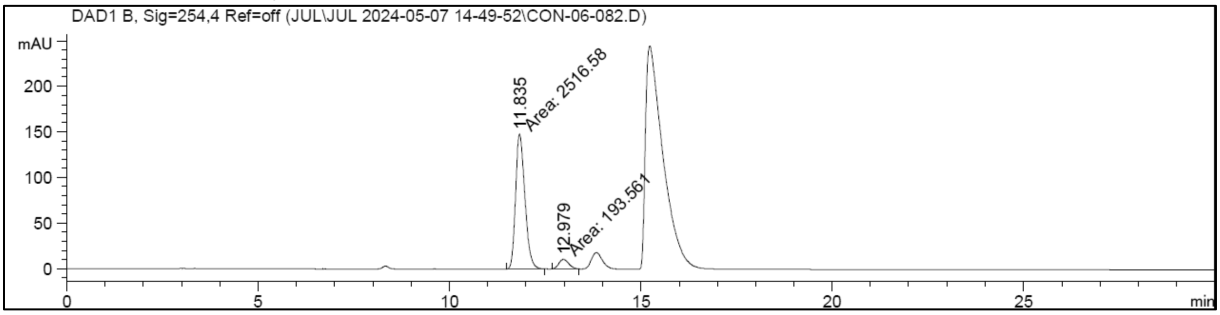

Signal 2: DAD1 B, Sig=254,4 Ref=off

Peak #	RetTime [min]	Type	Width [min]	Area [mAU*s]	Height [mAU]	Area %
1	11.835	FM	0.2834	2516.58301	147.98846	92.8579
2	12.979	MF	0.3054	193.56056	10.56353	7.1421

5,6a,7,8,9,10,11,11a-Octahydro-6H-cyclohepta[c]quinolin-6-one (2n)

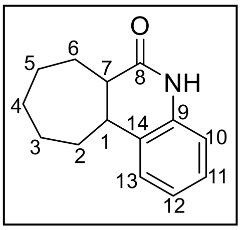

According to General Procedure **D**, **1n** (21.5 mg, 0.10 mmol, 1.00 eq.) was converted to **2n** yielding the product as white solid (14.2 mg, 66%) after purification by column chromatography (SiO_2, 15% EtOAc in *n*-pentane). The product was obtained as an inseparable mixture of diastereomers (d.r. 73:27 (*trans*:*cis*)).

trans-diastereomer:

R_f = 0.31 (20% EtOAc in *n*-pentane).

^{1}H NMR (500 MHz, $CDCl_3$): δ = 7.83 (s, 1H, NH), 7.29 (d, *J* = 7.7 Hz, 1H, H13), 7.19 (tt, *J* = 7.6, 1.1 Hz, 1H, H11), 7.05 (td, *J* = 7.6, 1.3 Hz, 1H, H12), 6.77 (dd, *J* = 7.7, 1.2 Hz, 1H, H10), 2.84 (ddd, *J* = 13.1, 7.8, 4.5 Hz, 1H, H1), 2.46 (ddt, *J* = 14.7, 7.3, 2.6 Hz, 1H, H5), 2.35 – 2.28 (m, 1H,

H2), 2.22 (ddd, J = 14.1, 8.7, 3.0 Hz, 1H, H7), 1.93 – 1.84 (m, 2H, H2, H6), 1.82 – 1.70 (m, 2H, H3, H5), 1.70 – 1.63 (m, 3H, H3, H4), 1.50 – 1.41 (m, 1H, H6) ppm.

^{13}C NMR (126 MHz, $CDCl_3$): δ = 174.4 (C8), 137.2 (C9), 130.1 (C14), 127.4 (C11), 124.3 (C13), 123.1 (C12), 115.2 (C10), 47.0 (C7), 40.8 (C1), 28.9 (C4), 28.0 (C2), 26.3 (C6), 24.71 (C3/C5), 24.69 (C3/C5) ppm.

IR (ATR): $\tilde{\nu}$ = 649 (w), 672 (w), 746 (m), 815 (w), 867 (w), 930 (w), 1045 (w), 1131 (w), 1257 (w), 1274 (w), 1297 (w), 1332 (w), 1383 (w), 1441 (w), 1464 (w), 1487 (w), 1590 (w), 1613 (w), 1676 (m), 2009 (w), 2175 (w), 2244 (w), 2359 (w), 2858 (w), 2927 (m), 2985 (w), 3071 (w), 3128 (w), 3203 (w), 3323 (w) cm^{-1}.

HR-ESI-MS: m/z: 238.12008 ($[M+Na]^+$, calcd. for $C_{14}H_{17}NONa^+$: 238.12024).

Mp = 172 – 174 °C.

e.r.: 85:15.

$[\alpha]_D^{22\,°C}$ = +126° (c = 0.5 in $CHCl_3$, for *e.r.* 85:15).

HPLC: AS-H column; eluent: *n*-hexane/ *i*-propanol 15:85; flow rate: 1.0 mL/min.

Racemic sample: ***trans*-2n**

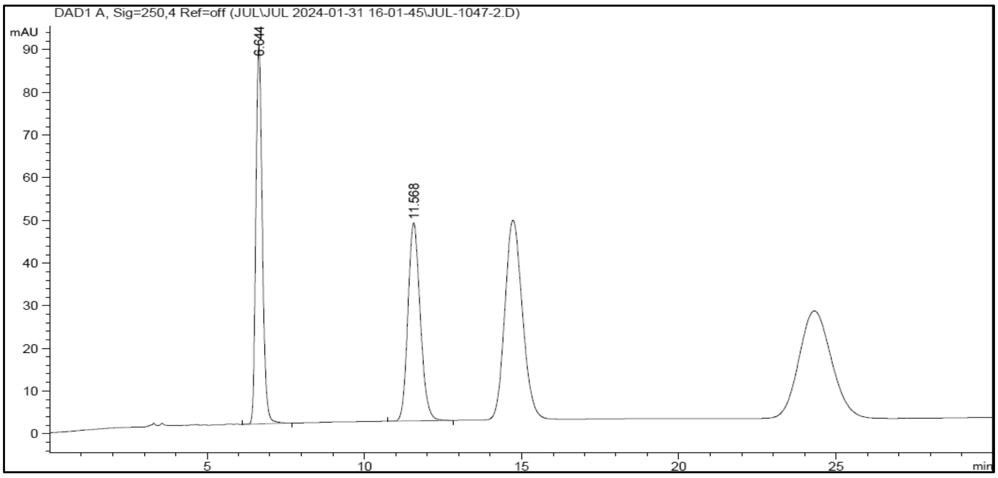

Signal 1: DAD1 A, Sig=250,4 Ref=off

Peak #	RetTime [min]	Type	Width [min]	Area [mAU*s]	Height [mAU]	Area %
1	6.644	BB	0.2174	1258.07190	88.72859	49.9478
2	11.568	BB	0.4193	1260.70154	46.35764	50.0522

Enantioselective sample: ***trans*-2n**

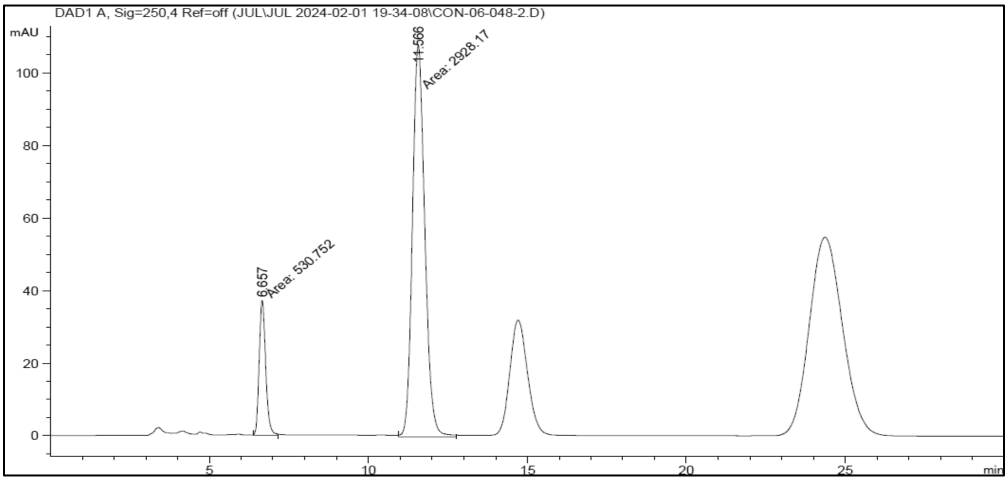

Signal 1: DAD1 A, Sig=250,4 Ref=off

Peak #	RetTime [min]	Type	Width [min]	Area [mAU*s]	Height [mAU]	Area %
1	6.657	MM	0.2375	530.75226	37.25038	15.3444
2	11.566	MM	0.4526	2928.17139	107.82748	84.6556

cis-diastereomer:

R*f* = 0.23 (20% EtOAc in *n*-pentane).

^{1}H NMR (500 MHz, $CDCl_3$): δ = 7.77 (s, 1H, NH), 7.19 – 7.13 (m, 2H, H11, H13), 7.00 (td, *J* = 7.5, 1.2 Hz, 1H, H12), 6.71 (dd, *J* = 7.9, 1.2 Hz, 1H, H10), 3.10 (ddd, *J* = 9.5, 5.9, 3.1 Hz, 1H, H1), 2.94 – 2.86 (m, 1H, H7), 2.17 – 2.07 (m, 1H, H6), 1.89 – 1.70 (m, 6H,, H2, H3, H4, H5, H6), 1.66 – 1.53 (m, 2H, H3, H5), 1.53 – 1.44 (m, 1H, H4) ppm.

^{13}C NMR (126 MHz, $CDCl_3$): δ = 173.7 (C8), 136.1 (C9), 130.0 (C14), 127.44 (C11), 127.38 (C13), 123.4 (C12), 115.1 (C10), 43.9 (C7), 41.8 (C1), 32.2 (C2), 28.9 (C4), 26.9 (C3), 26.7 (C6), 24.7 (C5) ppm.

IR (ATR): $\tilde{\nu}$ = 666 (w), 700 (w), 752 (w), 832 (w), 872 (w), 1194 (w), 1263 (w), 1291 (w), 1395 (w), 1435 (w), 1458 (w), 1492 (w), 1596 (w), 1596 (w), 1676 (m), 2032 (w), 2095 (w), 2336 (w), 2359 (w), 2858 (w), 2921 (m), 3059 (w), 3208 (w), 3260 (w) cm^{-1}.

HR-ESI-MS: *m/z*: 238.12004 ($[M+Na]^+$, calcd. for $C_{14}H_{17}NONa^+$: 238.12024).

Mp = 178 – 180 °C.

e.r.: 76:24.

$[\alpha]_{\mathrm{D}}^{22\,°\mathrm{C}}$ = –12.6° (*c* = 0.1 in $CHCl_3$, for *e.r.* 76:24).

HPLC: AS-H column; eluent: *n*-hexane/ *i*-propanol 15:85; flow rate: 1.0 mL/min.

Racemic sample: ***cis*-2n**

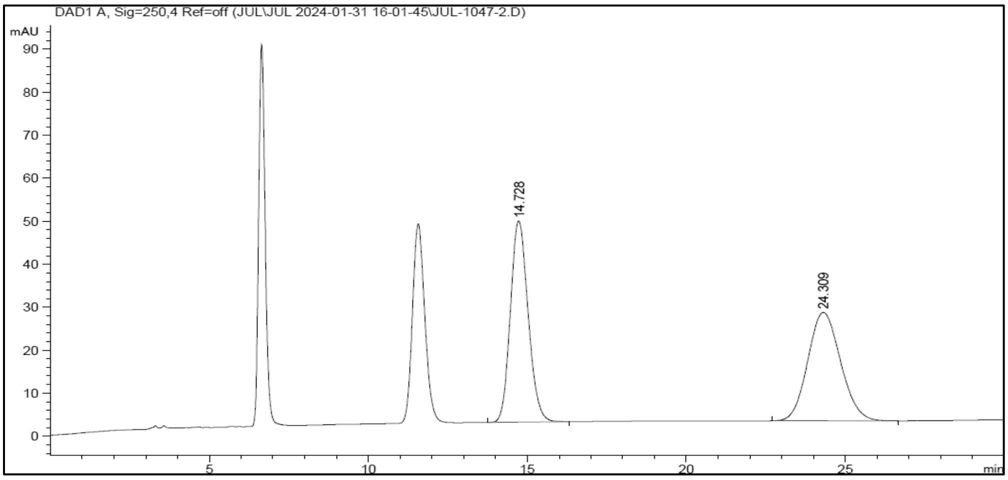

Signal 1: DAD1 A, Sig=250,4 Ref=off

Peak #	RetTime [min]	Type	Width [min]	Area [mAU*s]	Height [mAU]	Area %
1	14.728	BB	0.6026	1814.62878	46.77843	50.1675
2	24.309	BB	1.0746	1802.50964	25.19569	49.8325

Enantioselective sample: ***cis*-2n**

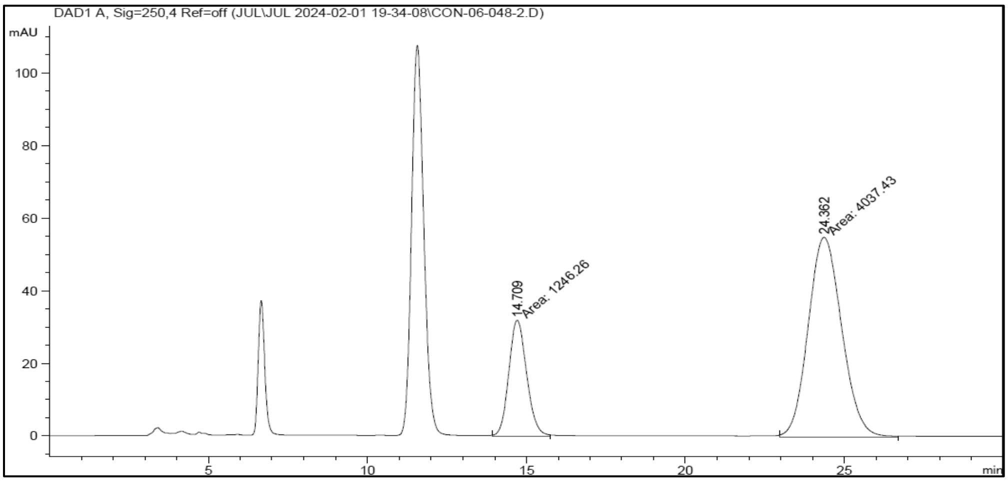

Signal 1: DAD1 A, Sig=250,4 Ref=off

Peak #	RetTime [min]	Type	Width [min]	Area [mAU*s]	Height [mAU]	Area %
1	14.709	MM	0.6503	1246.25977	31.94012	23.5869
2	24.362	MM	1.2233	4037.42822	55.00533	76.4131

5-Methyl-5,6a,7,8,9,10,11,11a-octahydro-6H-cyclohepta[c]quinolin-6-one (2o)

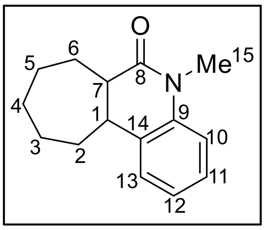

According to General Procedure **F**, **1o** (22.9 mg, 0.10 mmol, 1.00 eq.) was converted to **2o** yielding the product as white solid (22.6 mg, quant.) after purification by column chromatography (SiO_2, 10% EtOAc

in *n*-pentane). The product was obtained as an inseparable mixture of diastereomers (d.r. 63:37 (*trans*:*cis*)).

R*f* = 0.59 (15% EtOAc in *n*-pentane, both diastereomers).

HR-ESI-MS: *m/z*: 252.13561 ([*M*+Na]$^+$, calcd. for $C_{15}H_{19}NONa^+$: 252.13589).

trans-diastereomer:

^{1}H NMR (500 MHz, $CDCl_3$): δ = 7.29 – 7.24 (m, 2H, H11, H13), 7.07 (td, *J* = 7.6, 1.2 Hz, 1H, H12), 6.99 (dd, *J* = 8.0, 1.2 Hz, 1H, H10), 3.38 (s, 3H, H15), 2.78 (ddd, *J* = 14.2, 7.1, 4.5 Hz, 1H, H1), 2.39 (dddd, *J* = 14.7, 7.7, 3.1, 1.9 Hz, 1H, H6), 2.24 – 2.11 (m, 2H, H2, H7), 1.94 (ddd, *J* = 14.5, 9.7, 7.1 Hz, 1H, H2), 1.90 – 1.83 (m, 1H, H5), 1.80 – 1.70 (m, 2H, H3, H6), 1.70 – 1.58 (m, 2H, H3, H4), 1.45 – 1.36 (m, 1H, H5) ppm.

^{13}C NMR (126 MHz, $CDCl_3$): δ = 173.8 (C8), 140.7 (C9), 132.5 (C14), 127.2 (C11), 123.3 (C13), 122.9 (C12), 114.8 (C10), 47.6 (C7), 40.1 (C1), 30.4 (C15), 29.4 (C4), 27.3 (C2), 26.6 (C5), 25.1 (C6), 24.6 (C3) ppm.

e.r.: 96:4.

cis-diastereomer:

^{1}H NMR (500 MHz, $CDCl_3$), selected signals: δ = 7.22 (dd, *J* = 7.7, 1.6 Hz, 1H, H11), 7.17 (dd, *J* = 7.5, 1.6 Hz, 1H, H13), 7.03 (dd, *J* = 7.5, 1.1 Hz, 1H, H12), 6.95 (dd, *J* = 8.1, 1.1 Hz, 1H, H10), 3.36 (s, 3H, H15), 3.03 (ddd, *J* = 9.3, 5.6, 3.0 Hz, 1H, H1), 2.87 (d, *J* = 6.0 Hz, 1H, H7) ppm.

^{13}C NMR (126 MHz, $CDCl_3$): δ = 172.7 (C8), 139.4 (C9), 132.0 (C14), 127.3 (C11), 127.0 (C13), 123.0 (C12), 114.6 (C10), 44.2 (C7), 41.3 (C1), 31.8 ($C_{aliph.}$), 29.8 (C15), 28.7 ($C_{aliph.}$), 27.4 ($C_{aliph.}$), 26.8 (C6), 24.9 ($C_{aliph.}$) ppm.

e.r.: 91:9.

HPLC: AS-H column; eluent: *n*-hexane/ *i*-propanol 99:1; flow rate: 1.0 mL/min.

Racemic sample: ***trans*-2o**

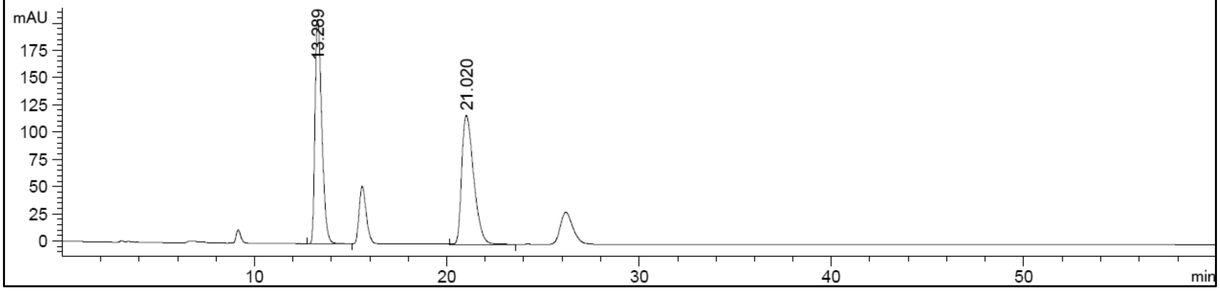

Signal 2: DAD1 B, Sig=254,4 Ref=360,100

Peak #	RetTime [min]	Type	Width [min]	Area [mAU*s]	Height [mAU]	Area %
1	13.289	BB	0.3851	5098.31543	205.76176	50.0849
2	21.020	BB	0.6631	5081.02881	118.28623	49.9151

Enantioselective sample: ***trans-2o***

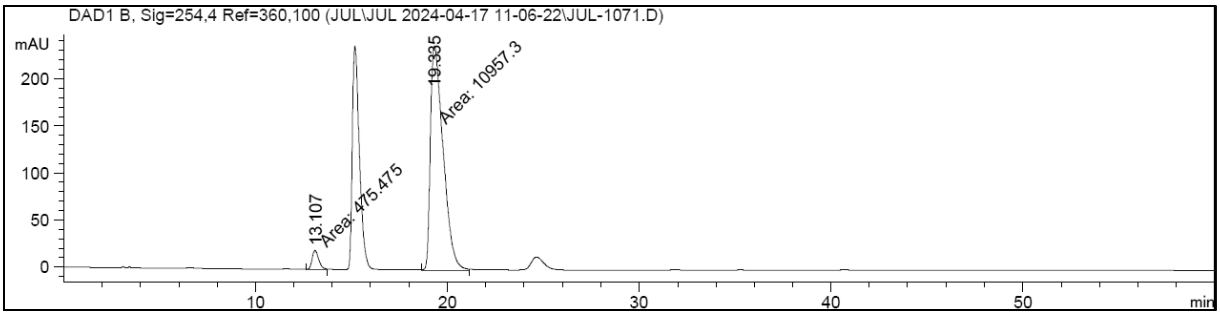

Signal 2: DAD1 B, Sig=254,4 Ref=360,100

Peak #	RetTime [min]	Type	Width [min]	Area [mAU*s]	Height [mAU]	Area %
1	13.107	MM	0.3950	475.47488	20.06220	4.1589
2	19.335	MM	0.7681	1.09573e4	237.76747	95.8411

Racemic sample: ***cis-2o***

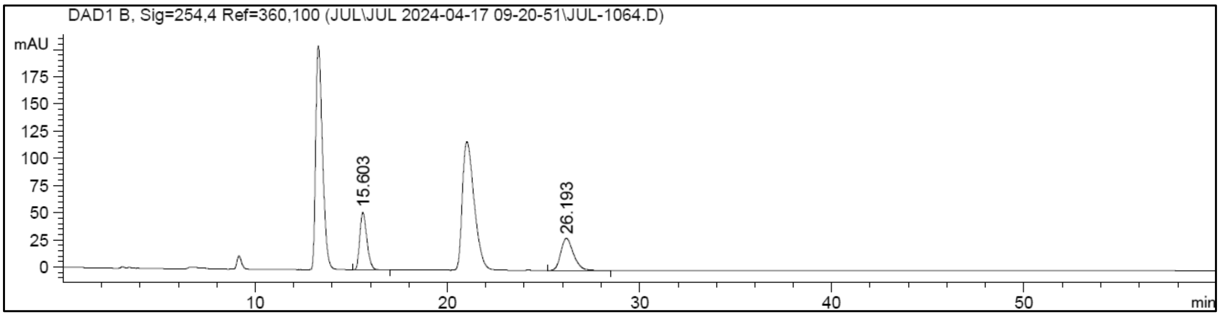

Signal 2: DAD1 B, Sig=254,4 Ref=360,100

Peak #	RetTime [min]	Type	Width [min]	Area [mAU*s]	Height [mAU]	Area %
1	15.603	BB	0.3946	1360.37341	53.14430	50.2505
2	26.193	BB	0.6945	1346.80981	29.50570	49.7495

Enantioselective sample: ***cis*-2o**

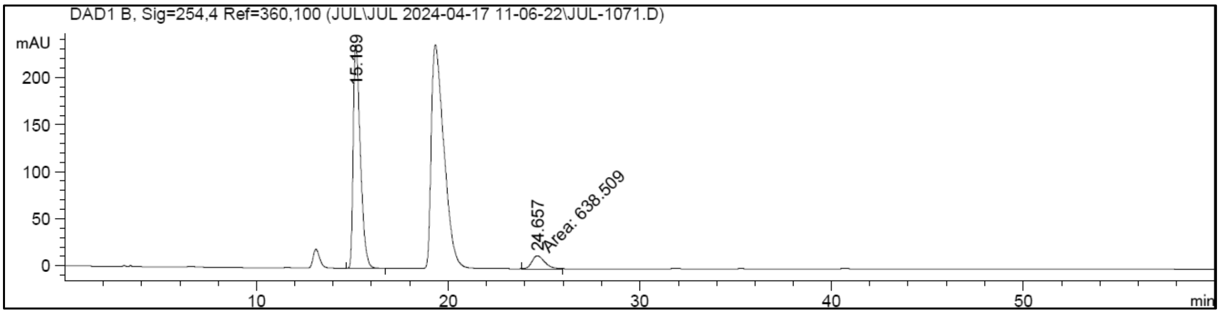

Signal 2: DAD1 B, Sig=254,4 Ref=360,100

Peak #	RetTime [min]	Type	Width [min]	Area [mAU*s]	Height [mAU]	Area %
1	15.189	BB	0.4000	6139.50537	237.14577	90.5797
2	24.657	FM	0.7690	638.50861	13.83818	9.4203

5-Methyl-1,2,3,3a,5,9b-hexahydro-4H-cyclopenta[c]quinolin-4-one (2p)

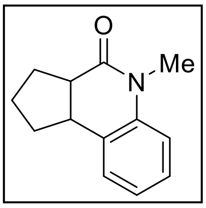

According to General Procedure **F**, **1p** (20.1 mg, 0.10 mmol, 1.00 eq.) was converted to **2p** yielding the product as white solid (16.8 mg, 84%) after purification by column chromatography (SiO_2, 20% EtOAc in *n*-pentane). The product was obtained as an inseparable mixture of diastereomers (d.r. 90:10 (*trans*:*cis*)).

trans-diastereomer:

R*f* = 0.41 (20% EtOAc in *n*-pentane, both isomers).

^{1}H NMR (400 MHz, $CDCl_3$): δ = 7.27 (tdd, *J* = 6.7, 1.7, 0.9 Hz, 1H), 7.14 (dt, *J* = 7.4, 1.5 Hz, 1H), 7.08 – 6.98 (m, 2H), 3.37 (s, 3H), 2.83 (ddd, *J* = 14.9, 11.4, 6.7 Hz, 1H), 2.28 (dddd, *J* = 13.1, 9.4, 7.6, 2.0 Hz, 2H), 2.14 – 2.04 (m, 1H), 2.00 – 1.79 (m, 3H), 1.72 – 1.58 (m, 1H) ppm.

HR-ESI-MS: *m/z*: 224.10444 ([*M*+Na]$^+$, calcd. for $C_{13}H_{15}NONa^+$: 224.10459).

Analytical data in agreement with literature.[7]

e.r.: 83:17.

HPLC: AD-H column; eluent: *n*-hexane/ *i*-propanol 99:1; flow rate: 1.0 mL/min.

Racemic sample: ***trans*-2p**

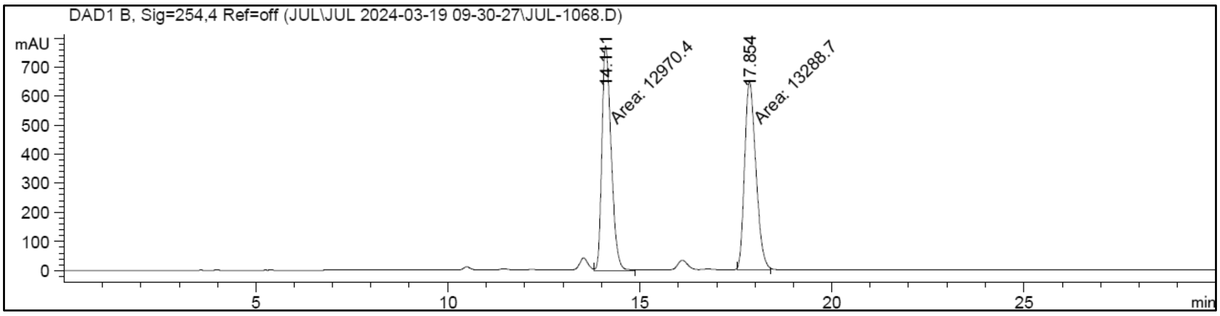

Signal 2: DAD1 B, Sig=254,4 Ref=off

Peak #	RetTime [min]	Type	Width [min]	Area [mAU*s]	Height [mAU]	Area %
1	14.111	FM	0.2802	1.29704e4	771.37653	49.3940
2	17.854	MF	0.3407	1.32887e4	650.08209	50.6060

Enantioselective sample: ***trans*-2p**

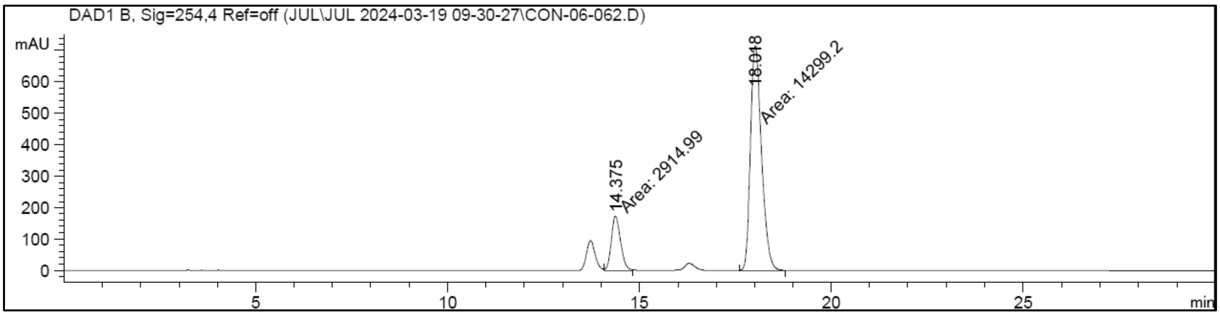

Signal 2: DAD1 B, Sig=254,4 Ref=off

Peak #	RetTime [min]	Type	Width [min]	Area [mAU*s]	Height [mAU]	Area %
1	14.375	MF	0.2814	2914.98535	172.63699	16.9337
2	18.018	MF	0.3338	1.42992e4	714.02631	83.0663

5-Methyl-6a,7,8,9,10,10a-hexahydrophenanthridin-6(5H)-one (2q)

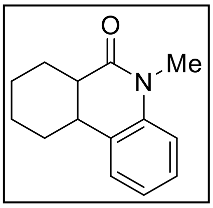

According to General Procedure **F**, **1q** (20.1 mg, 0.10 mmol, 1.00 eq.) was converted to **2q** yielding the product as white solid (16.8 mg, 84%) after purification by column chromatography (SiO_2, 20% EtOAc in *n*-pentane). The product was obtained as an inseparable mixture of diastereomers (d.r. 90:10 (*trans*:*cis*)).

trans-diastereomer:

R_f = 0.41 (20% EtOAc in *n*-pentane).

^{1}H NMR (400 MHz, $CDCl_3$): δ = 7.31 – 7.20 (m, 2H), 7.07 (td, *J* = 7.6, 1.2 Hz, 1H), 6.99 (dd, *J* = 8.1, 1.2 Hz, 1H), 3.37 (d, *J* = 0.7 Hz, 3H), 2.56 (ddd, *J* = 14.1, 10.4, 3.9 Hz, 1H), 2.50 – 2.35 (m, 2H), 2.07 – 1.97 (m, 1H), 1.93 (dddd, *J* = 12.3, 5.9, 3.0, 1.2 Hz, 2H), 1.48 – 1.29 (m, 4H) ppm.

HR-ESI-MS: *m/z*: 224.10444 ($[M+Na]^+$, calcd. for $C_{13}H_{15}NONa^+$: 224.10459).

Analytical data in agreement with literature.[25]

e.r.: 84:16.

HPLC: AD-H column; eluent: *n*-hexane/ *i*-propanol 97.5:2.5; flow rate: 1.0 mL/min.

Racemic sample: ***trans*-2q**

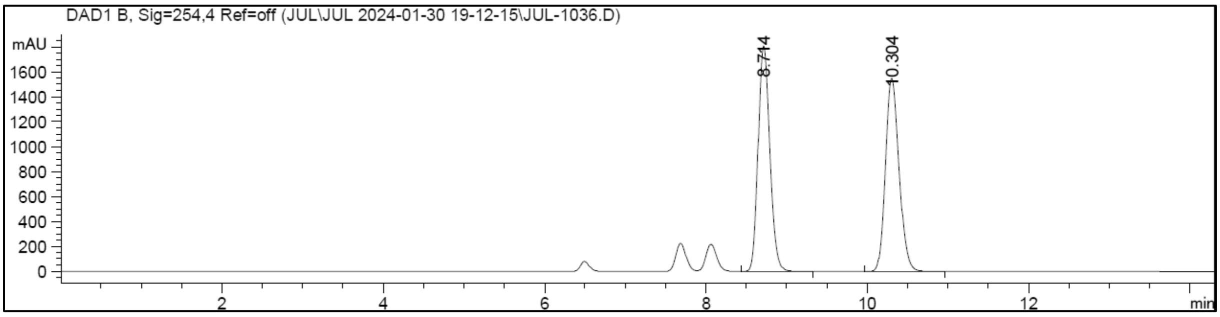

Signal 2: DAD1 B, Sig=254,4 Ref=off

Peak #	RetTime [min]	Type	Width [min]	Area [mAU*s]	Height [mAU]	Area %
1	8.714	BB	0.1576	1.82211e4	1807.99805	49.9719
2	10.304	BB	0.1834	1.82416e4	1549.26428	50.0281

Enantioselective sample: ***trans*-2q**

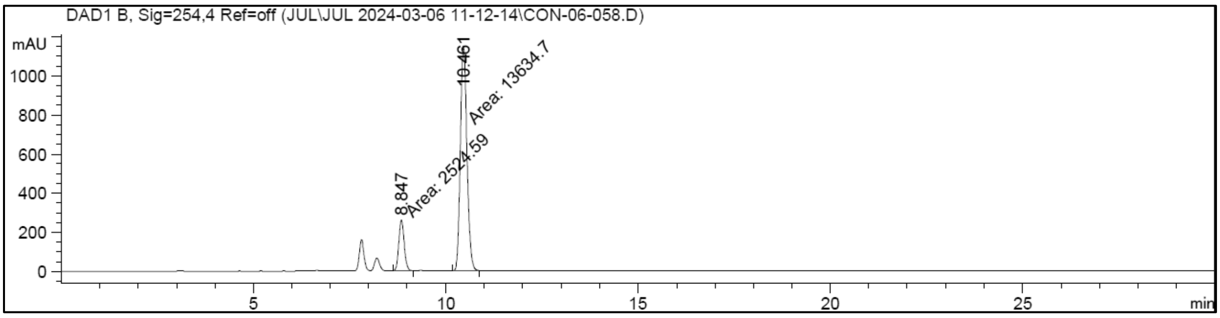

Signal 2: DAD1 B, Sig=254,4 Ref=off

Peak #	RetTime [min]	Type	Width [min]	Area [mAU*s]	Height [mAU]	Area %
1	8.847	MM	0.1622	2524.58545	259.43124	15.6232
2	10.461	MM	0.1974	1.36347e4	1151.08289	84.3768

5,6,8a,9,10,11,12,12a-Octahydro-4H,8H-pyrido[3,2,1-de]phenanthridin-8-one (2r)

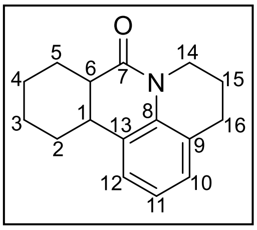

According to General Procedure **F**, **1r** (24.1 mg, 0.10 mmol, 1.00 eq.) was converted to **2r** yielding the product as white solid (22.0 mg, 91%) after purification by column chromatography (SiO_2, 20% EtOAc in *n*-pentane). The product was obtained as a mixture of diastereomers (d.r. >95:5 (*trans*:*cis*)).

trans-diastereomer:

R_f = 0.48 (15% EtOAc in *n*-pentane).

^{1}H NMR (600 MHz, $CDCl_3$): δ = 7.08 (dt, *J* = 7.4, 1.4 Hz, 1H, H12), 7.02 (ddq, *J* = 7.5, 1.7, 0.9 Hz, 1H, H11), 6.97 (t, *J* = 7.5 Hz, 1H, H10), 4.29 (ddd, *J* = 12.5, 7.8, 4.2 Hz, 1H, H14), 3.48 (dddt, *J* = 13.3, 7.7, 3.9, 0.9 Hz, 1H, H14), 2.86 – 2.81 (m, 1H, H16), 2.76 (dt, *J* = 16.1, 5.4 Hz, 1H, H16), 2.51 (ddd, *J* = 14.4, 10.4, 4.1 Hz, 1H, H1), 2.46 – 2.38 (m, 2H, H2, H5), 2.02 (ddd, *J* = 14.2, 11.7, 3.9 Hz, 1H, H6), 1.98 – 1.88 (m, 4H, H3, H4, 2 x H15), 1.42 – 1.32 (m, 3H, H2, H3, H5), 1.31 – 1.25 (m, 1H, H4) ppm.

^{13}C NMR (151 MHz, $CDCl_3$): δ = 171.8 (C7), 135.7 (C8), 130.0 (C13), 127.8 (C11), 125.1 (C9), 122.4 (10), 122.0 (C12), 43.4 (C6), 41.3 (C14), 37.2 (C1), 29.0 (C2), 27.6 (C16), 27.0 (C5), 25.4 (C3, C4), 21.7 (C15) ppm.

HR-ESI-MS: *m/z*: 269.13573 ([*M*+Na]$^+$, calcd. for $C_{16}H_{19}NO_2Na^+$: 269.13589).

e.r.: 81:19.

HPLC: AS-H column; eluent: *n*-hexane/ *i*-propanol 99:1; flow rate: 1.0 mL/min.

Racemic sample: ***trans*-2r**

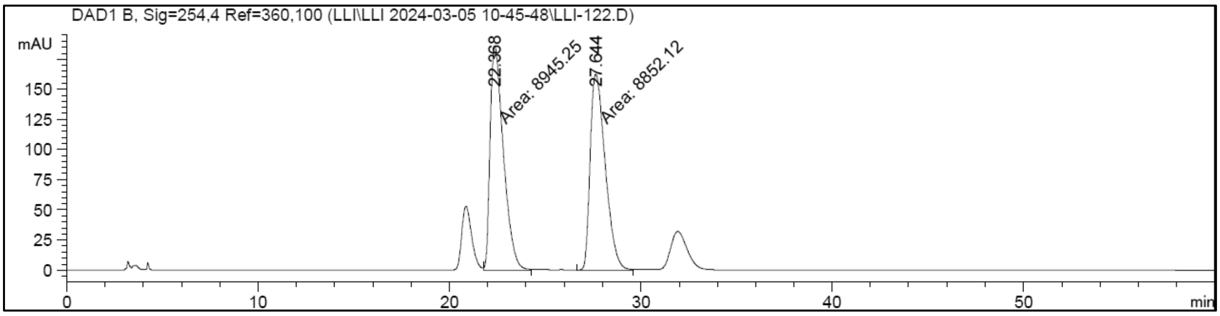

Signal 2: DAD1 B, Sig=254,4 Ref=360,100

Peak #	RetTime [min]	Type	Width [min]	Area [mAU*s]	Height [mAU]	Area %
1	22.368	FM	0.8023	8945.24512	185.81577	50.2616
2	27.644	MF	0.9084	8852.11914	162.41460	49.7384

Enantioselective sample: ***trans*-2r**

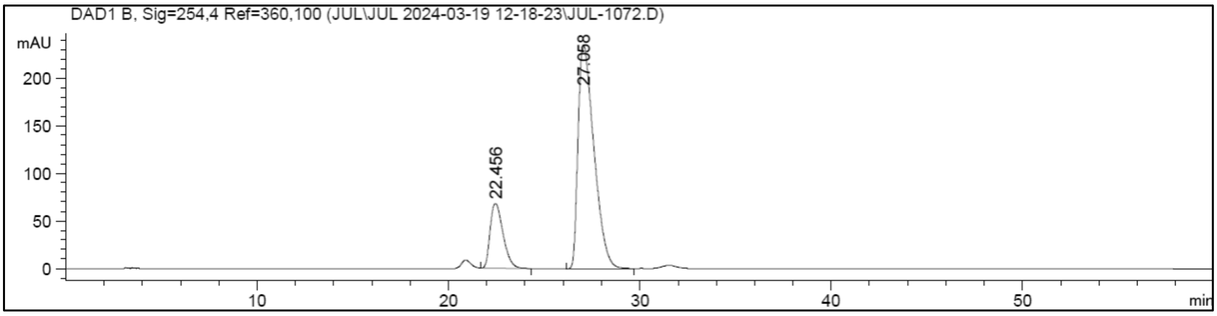

Signal 2: DAD1 B, Sig=254,4 Ref=360,100

Peak #	RetTime [min]	Type	Width [min]	Area [mAU*s]	Height [mAU]	Area %
1	22.456	BB	0.7425	3192.12646	68.14601	19.0908
2	27.058	BB	0.8730	1.35287e4	235.23956	80.9092

2-Fluoro-5-methyl-6a,7,8,9,10,10a-hexahydrophenanthridin-6(5H)-one (2s)

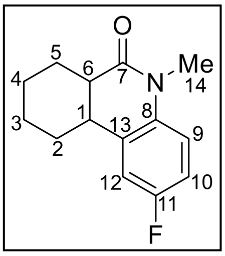

According to General Procedure **F**, **1s** (23.3 mg, 0.10 mmol, 1.00 eq.) was converted to **2s** yielding the product as white solid (16.5 mg, 71%) after purification by column chromatography (SiO_2, 10% EtOAc in *n*-pentane). The product was obtained as an inseparable mixture of diastereomers (d.r. 90:10 (*trans*:*cis*)).

R_f = 0.50 (20% EtOAc in *n*-pentane, both diastereomers).

HR-ESI-MS: *m/z*: 256.1108 ([*M*+Na]$^+$, calcd. for $C_{14}H_{16}NONa^+$: 256.1108).

trans-diastereomer:

^{1}H NMR (500 MHz, $CDCl_3$): δ = 6.96 (q, *J* = 0.9 Hz, 1H, H12), 6.94 (dd, *J* = 2.8, 0.8 Hz, 1H, H10), 6.92 (d, *J* = 1.2 Hz, 1H, H9), 3.35 (s, 3H, H14), 2.53 (ddd, *J* = 14.4, 10.5, 4.0 Hz, 1H, H1), 2.39 (dtd, *J* = 10.2, 6.3, 3.1 Hz, 2H, H2, H5), 2.01 – 1.90 (m, 3H, H3, H4, H6), 1.40 – 1.32 (m, 3H, H2, H3/H4, H5), 1.28 – 1.24 (m, 1H, H3/H4) ppm.

^{13}C NMR {^{19}F} (126 MHz, $CDCl_3$): δ = 172.1 (C7), 159.1 (d, *J* = 241.7 Hz, C11), 136.4 (C8), 132.9 (d, *J* = 7.1 Hz, C13), 115.6 (d, *J* = 8.1 Hz, C9), 113.4 (d, *J* = 22.4 Hz, C10), 111.6 (d, *J* = 23.8 Hz, C12), 43.3 (C6), 37.1 (C1), 30.2 (C14), 28.9 (C2), 27.0 (C5), 25.24 (C3/C4), 25.17 (C3/C4) ppm.

^{19}F NMR {^{1}H} (470 MHz, $CDCl_3$): δ = –120.51 ppm.

e.r.: 82:18.

HPLC: AS-H column; eluent: *n*-hexane/ *i*-propanol 99:1; flow rate: 1.0 mL/min.

Racemic sample: ***trans*-2s**

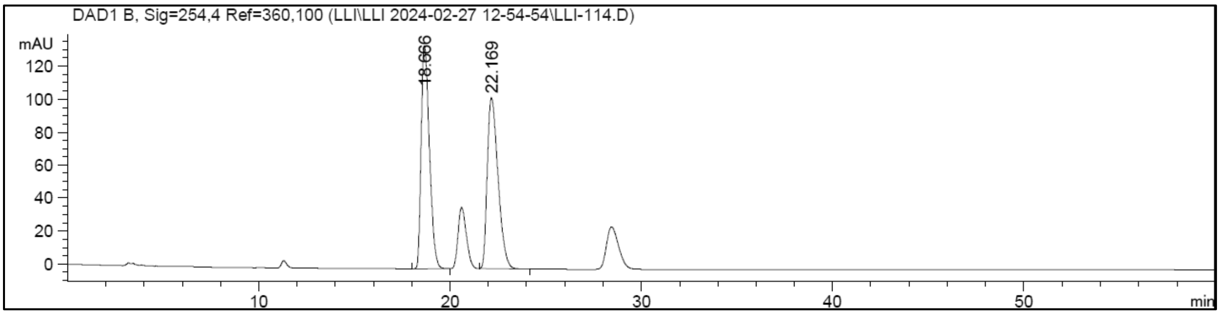

Signal 2: DAD1 B, Sig=254,4 Ref=360,100

Peak #	RetTime [min]	Type	Width [min]	Area [mAU*s]	Height [mAU]	Area %
1	18.666	BB	0.4687	4068.55640	135.36670	50.0029
2	22.169	BB	0.6053	4068.07983	103.78036	49.9971

Enantioselective sample: ***trans*-2s**

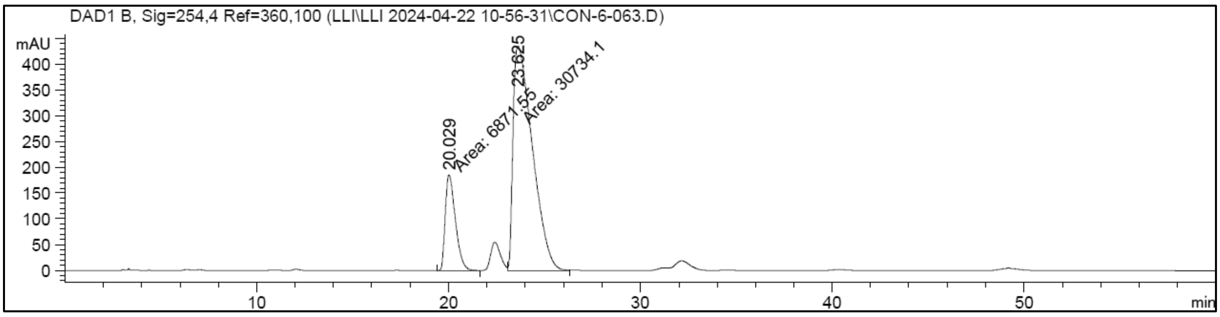

Signal 2: DAD1 B, Sig=254,4 Ref=360,100

Peak #	RetTime [min]	Type	Width [min]	Area [mAU*s]	Height [mAU]	Area %
1	20.029	MM	0.6172	6871.54541	185.56987	18.2727
2	23.625	FM	1.1740	3.07341e4	436.30698	81.7273

5-Methyl-2-(trifluoromethyl)-6a,7,8,9,10,10a-hexahydrophenanthridin-6(5H)-one (2t)

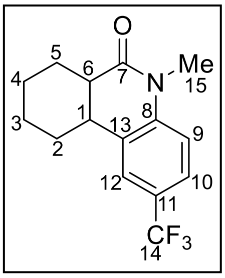

According to General Procedure **F**, **1t** (28.3 mg, 0.10 mmol, 1.00 eq.) was converted to **2t** yielding the product as white solid (26.0 mg, 92%) after purification by column chromatography (SiO_2, 10% EtOAc in *n*-pentane). The product was obtained as a mixture of diastereomers (d.r. 90:10 (*trans*:*cis*)).

trans-diastereomer:

R*f* = 0.37 (20% EtOAc in *n*-pentane).

^{1}H NMR (600 MHz, $CDCl_3$): δ = 7.56 – 7.52 (m, 1H, H10), 7.49 – 7.46 (m, 1H, H12), 7.06 (t, *J* = 8.7 Hz, 1H, H9), 3.39 (s, 3H, H15), 2.59 (ddd, *J* = 14.4, 10.5, 4.1 Hz, 1H, H1), 2.51 – 2.46 (m,

1H, H2), 2.45 – 2.39 (m, 1H, H5), 2.06 – 1.92 (m, 3H, H3, H4, H6), 1.45 – 1.35 (m, 3H, H2, H3, H5), 1.32 – 1.24 (m, 1H, H4) ppm.

^{13}C NMR (151 MHz, $CDCl_3$): δ = 172.4 (C7), 143.0 (C8), 131.2 (C13), 124.9 (d, *J* = 32.7 Hz, C11), 124.7 (q, *J* = 4.0 Hz, C10), 124.5 (C14), 121.3 (q, *J* = 3.8 Hz, C12), 114.5 (C9), 43.3 (C6), 37.0 (C1), 30.1 (C15), 28.3 (C2), 27.0 (C5), 25.2 (C4), 25.1 (C3) ppm.

^{19}F NMR {^{1}H} (564 MHz, $CDCl_3$): δ = –61.90 ppm.

IR (ATR): $\tilde{\nu}$ = 637 (w), 660 (w), 695 (w), 740 (w), 758 (w), 827 (w), 855 (w), 890 (w), 953 (w), 993 (w), 1113 (s), 1234 (w), 1286 (s), 1332 (s), 1366 (m), 1418 (w), 1452 (w), 1475 (w), 1510 (w), 1619 (m), 1682 (s), 2858 (w), 2927 (w) cm^{-1}.

HR-ESI-MS: *m/z*: 306.10762 ($[M+Na]^+$, calcd. for $C_{15}H_{16}NOF_3Na^+$: 306.10762).

Mp = 85 – 86 °C.

e.r.: 79:21.

$[\alpha]_D^{21\,°C}$ = +94.9° (*c* = 1.0 in $CHCl_3$, for *e.r.* 79:21).

HPLC: OD-H column; eluent: *n*-hexane/ *i*-propanol 99:1; flow rate: 1.0 mL/min.

Racemic sample: ***trans*-2t**

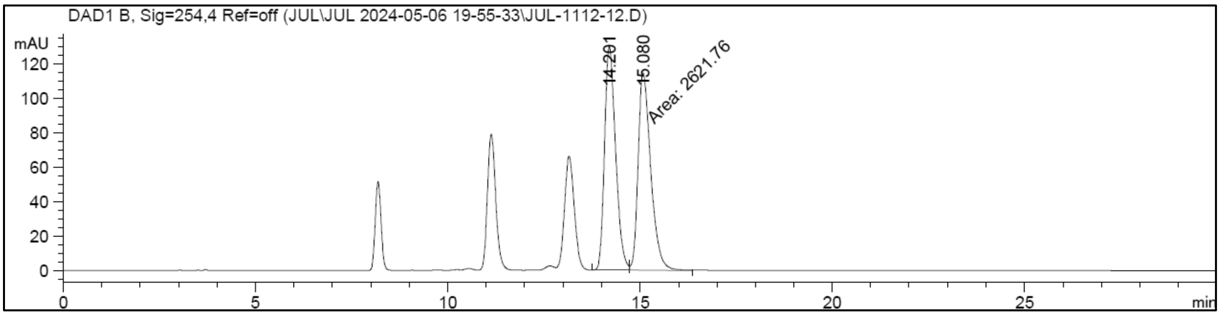

Signal 2: DAD1 B, Sig=254,4 Ref=off

Peak #	RetTime [min]	Type	Width [min]	Area [mAU*s]	Height [mAU]	Area %
1	14.201	BV	0.3065	2597.06616	130.29582	49.7634
2	15.080	MF	0.3821	2621.75879	114.36512	50.2366

Enantioselective sample: ***trans*-2t**

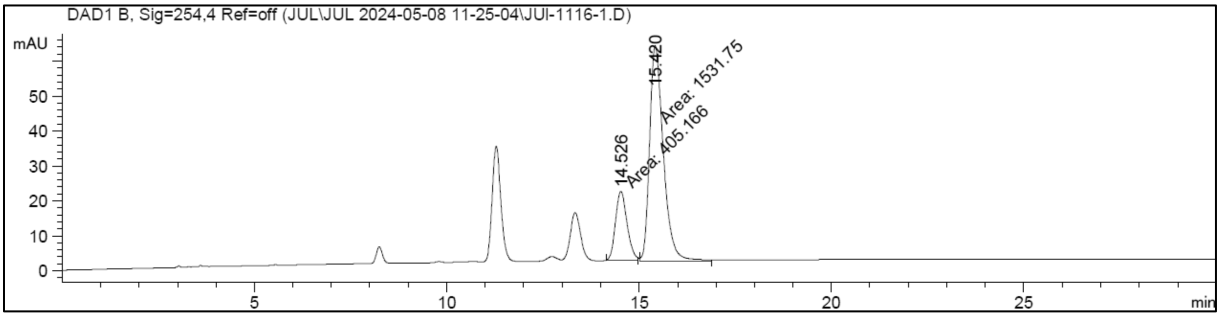

```
Signal 2: DAD1 B, Sig=254,4 Ref=off

Peak RetTime Type  Width     Area      Height     Area
  #   [min]        [min]   [mAU*s]     [mAU]       %
----|-------|----|-------|----------|----------|--------|
   1  14.526 MM    0.3453  405.16580   19.55865  20.9181
   2  15.420 FM    0.4143 1531.75122   61.61400  79.0819
```

2-Bromo-5-methyl-6a,7,8,9,10,10a-hexahydrophenanthridin-6(5H)-one (2u)

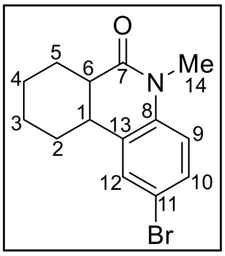

According to General Procedure **F**, **1u** (29.4 mg, 0.10 mmol, 1.00 eq.) was converted to **2u** yielding the product as white solid (27.5 mg, 94%) after purification by column chromatography (SiO_2, 10% EtOAc in *n*-pentane). The product was obtained as a mixture of diastereomers (d.r. 91:9 (*trans*:*cis*)).

trans-diastereomer:

R*f* = 0.55 (20% EtOAc in *n*-pentane).

^{1}H NMR (600 MHz, $CDCl_3$): δ = 7.37 (ddd, *J* = 8.6, 2.3, 1.0 Hz, 1H, H10), 7.33 (dd, *J* = 2.3, 1.3 Hz, 1H, H12), 6.84 (d, *J* = 8.6 Hz, 1H, H9), 3.33 (s, 3H, H14), 2.58 – 2.50 (m, 1H, H1), 2.42 – 2.36 (s, 2H, H2, H5), 2.00 – 1.89 (m, 3H, H3, H4, H6), 1.40 – 1.31 (m, 3H, H2, H3, H5), 1.39 – 1.24 (m, 1H, H4) ppm.

^{13}C NMR (151 MHz, $CDCl_3$): δ = 172.2 (C7), 139.3 (C8), 132.8 (C13), 130.1 (C10), 127.4 (C12), 116.2 (C9), 116.0 (C11), 43.3 (C6), 37.1 (C1), 30.1 (C14), 28.8 (C2), 27.0 (C5), 25.2 (C4), 25.1 (C3) ppm.

IR (ATR): $\tilde{\nu}$ = 637 (w), 781 (w), 809 (m), 855 (w), 872 (w), 895 (w), 947 (w), 993 (w), 1068 (w), 1108 (m), 1136 (m), 1234 (w), 1263 (w), 1337 (m), 1366 (m), 1412 (s), 1452 (m), 1487 (m), 1590 (w), 1670 (s), 2853 (w), 2927 (m) cm^{-1}.

HR-ESI-MS: *m/z*: 316.03075 ([*M*+Na]$^+$, calcd. for $C_{14}H_{16}NOBrNa^+$: 316.03075).

Mp = 109 – 111 °C.

e.r.: 84:16.

$[\alpha]_D^{21\,°C}$ = +96.5° (*c* = 1.0 in $CHCl_3$, for *e.r.* 84:16).

HPLC: AD-H column; eluent: *n*-hexane/ *i*-propanol 98:2; flow rate: 1.0 mL/min.

Racemic sample: ***trans*-2u**

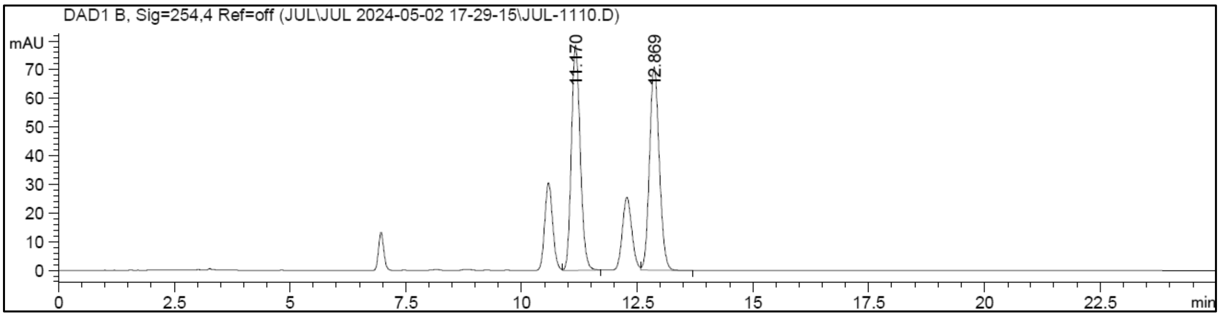

Signal 2: DAD1 B, Sig=254,4 Ref=off

Peak #	RetTime [min]	Type	Width [min]	Area [mAU*s]	Height [mAU]	Area %
1	11.170	VB	0.2050	1042.49475	78.45105	49.9051
2	12.869	VB	0.2281	1046.45996	70.92318	50.0949

Enantioselective sample: ***trans*-2u**

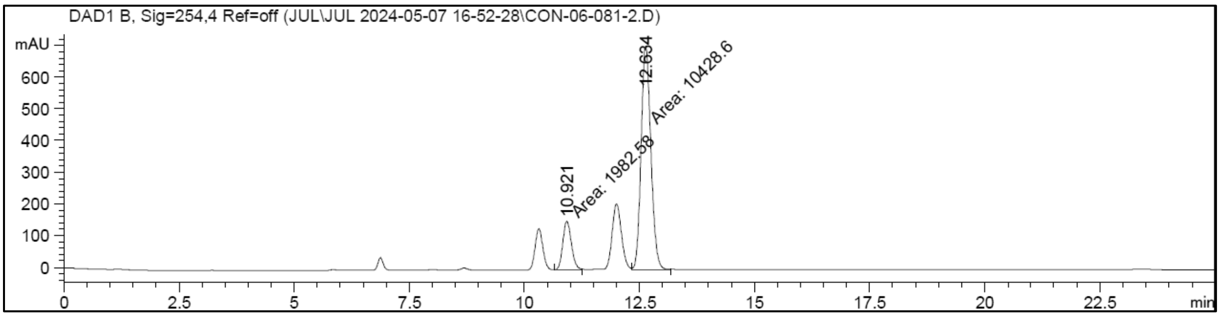

Signal 2: DAD1 B, Sig=254,4 Ref=off

Peak #	RetTime [min]	Type	Width [min]	Area [mAU*s]	Height [mAU]	Area %
1	10.921	MF	0.2157	1982.57703	153.21458	15.9741
2	12.634	MF	0.2461	1.04286e4	706.21631	84.0259

2-Bromo-6a,7,8,9,10,10a-hexahydrophenanthridin-6(5H)-one (S1)

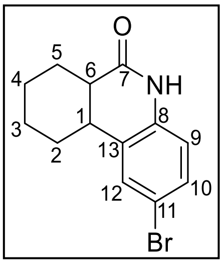

According to General Procedure **D**, **S31** (23.6 mg, 0.10 mmol, 1.00 eq.) was converted to **S1** after 65 h reaction time yielding the product as white solid (16.9 mg, 60%) after purification by column chromatography (SiO_2, 15-20% EtOAc in *n*-pentane). The product was obtained as an inseparable mixture of diastereomers (d.r. 62:38 (*trans*:*cis*)).

R*f* = 0.33 (30% EtOAc in *n*-pentane, both diastereomers).

HR-ESI-MS: *m/z*: 302.01503 ([*M*+Na]$^+$, calcd. for $C_{13}H_{14}NOBrNa^+$: 302.0151).

trans-diastereomer:

^{1}H NMR (500 MHz, $CDCl_3$): δ = 8.88 (s, 1H, NH), 7.33 (t, *J* = 1.8 Hz, 1H, H12), 7.29 (ddd, *J* = 8.3, 2.2, 0.9 Hz, 1H, H10), 6.71 (d, *J* = 8.3 Hz, 1H, H9), 2.64 – 2.55 (m, 1H, H1), 2.49 – 2.37 (m, 2H,

H2, H5), 2.04 (ddd, J = 14.1, 11.5, 3.7 Hz, 1H, H6), 1.99 – 1.89 (m, 2H, H3, H4), 1.42 – 1.33 (m, 3H, H2, H3/H4, H5), 1.33 – 1.27 (m, 1H, H3/H4) ppm.

^{13}C NMR (126 MHz, $CDCl_3$): δ = 173.7 (C7), 136.0 (C8), 130.6 (C10), 130.3 (C13), 127.8 (C12), 116.9 (C9), 115.9 (C11), 43.1 (C6), 38.0 (C1), 28.8 (C2), 26.2 (C5), 25.24 (C3/C4), 25.22 (C3/C4) ppm.

e.r.: 78:22.

cis-diastereomer:

^{1}H NMR (500 MHz, $CDCl_3$): δ = 8.91 (s, 1H, NH), 7.28 – 7.25 (m, 2H, H10, H12), 6.70 – 6.67 (m, 1H, H9), 2.93 (dt, J = 10.3, 5.2 Hz, 1H, H1), 2.77 (q, J = 4.6 Hz, 1H, H6), 2.30 (s, 1H, H5), 1.94 – 1.90 (m, 1H, H3), 1.75 – 1.69 (m, 1H, H3), 1.69 – 1.63 (m, 1H, H2), 1.62 – 1.55 (m, 2H, H2, H4), 1.53 – 1.43 (m, 2H, H4, H5) ppm.

^{13}C NMR (126 MHz, $CDCl_3$): δ = 173.1 (C7), 135.7 (C8), 130.34 (C10/12, C13), 130.26 (C10/12), 117.1 (C9), 115.6 (C11), 40.6 (C6), 38.9 (C1), 29.6 (C2), 25.0 (C3), 24.4 (C5), 22.8 (C4) ppm.

e.r.: 89:11.

HPLC: AS-H column; eluent: *n*-hexane/ *i*-propanol 15:85; flow rate: 1.0 mL/min.

Racemic sample: ***trans*-S1**

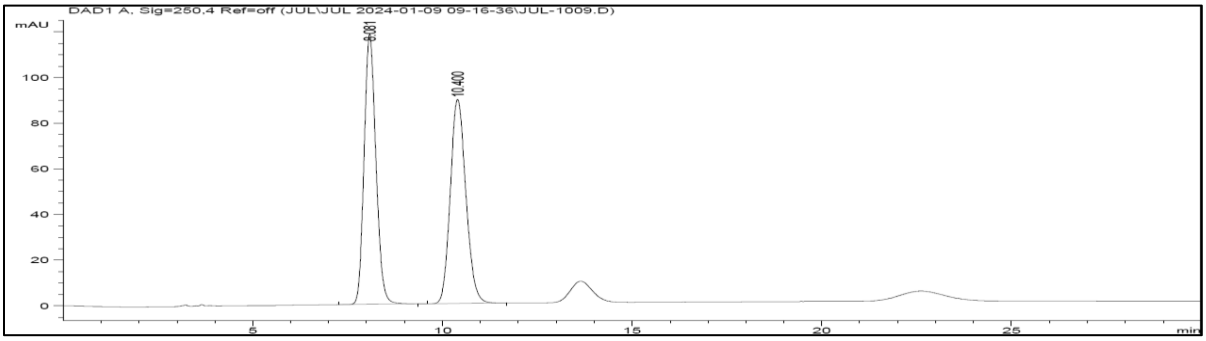

Signal 1: DAD1 A, Sig=250,4 Ref=off

Peak #	RetTime [min]	Type	Width [min]	Area [mAU*s]	Height [mAU]	Area %
1	8.081	BB	0.3343	2560.73120	118.39822	50.0244
2	10.400	BB	0.4437	2558.23267	89.45093	49.9756

Enantioselective sample: ***trans*-S1**

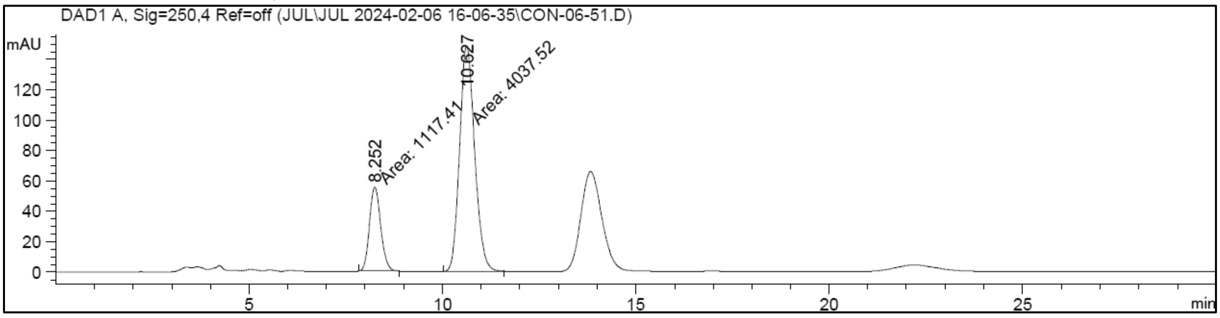

```
Signal 1: DAD1 A, Sig=250,4 Ref=off

Peak RetTime Type  Width     Area       Height     Area
  #    [min]        [min]   [mAU*s]     [mAU]        %
----|-------|----|-------|----------|----------|--------|
   1    8.252 MM    0.3369 1117.40796   55.28631  21.6765
   2   10.627 MM    0.4527 4037.52466  148.64900  78.3235
```

Racemic sample: ***cis*-S1**

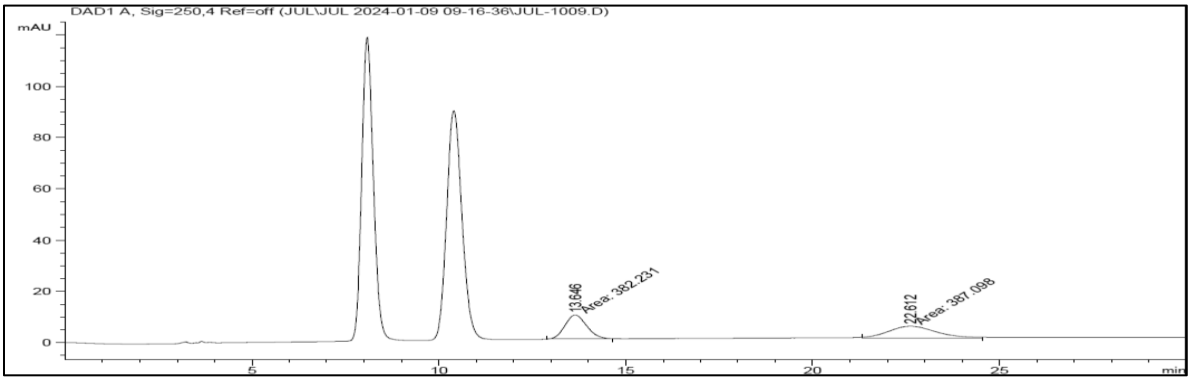


```
Signal 1: DAD1 A, Sig=250,4 Ref=off

Peak RetTime Type  Width     Area       Height     Area
  #    [min]        [min]   [mAU*s]     [mAU]        %
----|-------|----|-------|----------|----------|--------|
   1   13.646 MM    0.6809  382.23065    9.35593  49.6837
   2   22.612 MF    1.4346  387.09796    4.49731  50.3163
```

Enantioselective sample: ***cis*-S1**

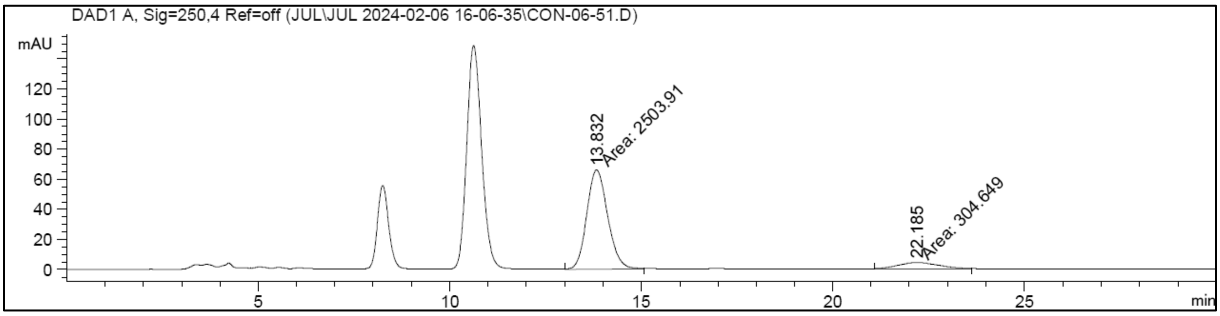


```
Signal 1: DAD1 A, Sig=250,4 Ref=off

Peak RetTime Type  Width     Area       Height     Area
  #    [min]        [min]   [mAU*s]     [mAU]        %
----|-------|----|-------|----------|----------|--------|
   1   13.832 MM    0.6325 2503.91016   65.97643  89.1528
   2   22.185 MM    1.2290  304.64944    4.13146  10.8472
```

2-Methyl-6a,7,8,9,10,10a-hexahydrophenanthridin-6(5H)-one (S2)

According to General Procedure **D**, **S32** (21.5 mg, 0.10 mmol, 1.00 eq.) was converted to **S2** yielding the product as white solid (15.7 mg, 73%) after purification by column

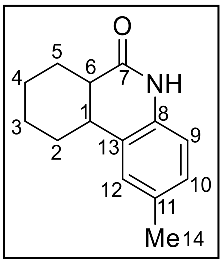

chromatography (SiO_2, 20% EtOAc in *n*-pentane). The product was obtained as an inseparable mixture of diastereomers (d.r. 62:38 (*trans*:*cis*)).

R*f* = 0.42 (30% EtOAc in *n*-pentane, both diastereomers).

HR-ESI-MS: *m/z*: 238.12010 ($[M+Na]^+$, calcd. for $C_{14}H_{17}NONa^+$: 238.12024).

trans-diastereomer:

^{1}H NMR (500 MHz, $CDCl_3$): δ = 8.49 (s, 1H, NH), 7.04 (s, 1H, H12), 6.99 (ddt, *J* = 7.9, 1.8, 0.9 Hz, 1H, H10), 6.70 (d, *J* = 7.9 Hz, 1H, H9), 2.58 (ddd, *J* = 14.3, 10.4, 4.0 Hz, 1H, H1), 2.51 – 2.45 (m, 1H, H2), 2.45 – 2.39 (m, 1H, H5), 2.32 (s, 3H, H14), 2.04 (ddd, *J* = 14.0, 11.6, 3.7 Hz, 1H, H6), 1.98 – 1.90 (m, 2H, H4), 1.41 – 1.34 (m, 3H, H2, H3/H4, H5), 1.31 – 1.25 (m, 1H, H3/H4) ppm.

^{13}C NMR (126 MHz, $CDCl_3$): δ = 173.7 (C7), 134.4 (C8), 132.5 (C11), 128.4 (C13), 127.8 (C10), 125.1 (C12), 115.2 (C9), 43.5 (C6), 38.0 (C1), 28.9 (C2), 26.3 (C5), 25.41 (C3/C4), 25.39 (C3/C4), 21.2 (C14) ppm.

e.r.: 86:14.

cis-diastereomer:

^{1}H NMR (500 MHz, $CDCl_3$): δ = 8.49 (s, 1H, NH), 6.97 – 6.95 (m, 2H, H10, H12), 6.68 (d, *J* = 8.4 Hz, 1H, H9), 2.90 (dt, *J* = 10.2, 5.1 Hz, 1H, H1), 2.78 (q, *J* = 4.5 Hz, 1H, H6), 2.37 – 2.33 (m, 1H, H5), 2.30 (s, 3H, H14), 1.93 – 1.89 (m, 1H, H3), 1.76 – 1.70 (m, 1H, H3), 1.68 – 1.55 (m, 3H, H2, H4), 1.54 – 1.47 (m, 2H, H4, H5) ppm.

^{13}C NMR (126 MHz, $CDCl_3$): δ = 173.1 (C7), 134.0 (C8), 132.7 (C11), 128.4 (C13), 128.0 (C10), 127.9 (C12), 115.3 (C9), 40.9 (C6), 39.1 (C1), 29.8 (C2), 25.5 (C3), 24.7 (C5), 22.9 (C4), 21.0 (C14) ppm. C3 detected via 2D NMR.

e.r.: 83:17.

HPLC: AS-H column; eluent: *n*-hexane/ *i*-propanol 15:85; flow rate: 1.0 mL/min.

Racemic sample: ***trans*-S2**

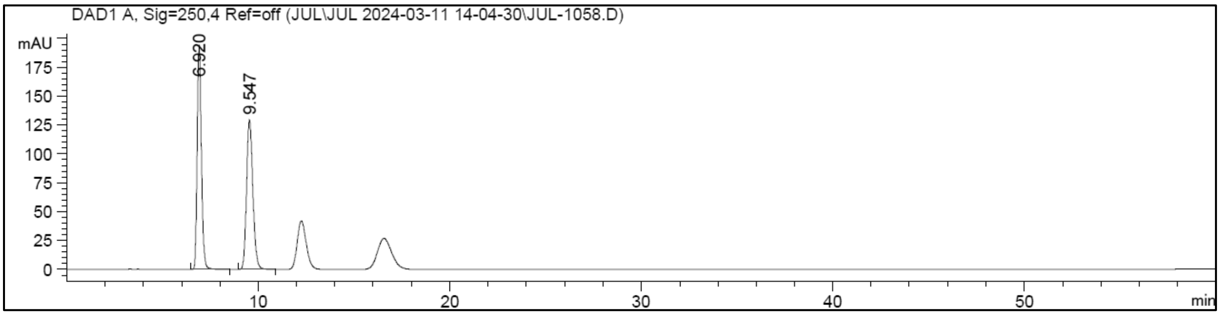

Signal 1: DAD1 A, Sig=250,4 Ref=off

Peak #	RetTime [min]	Type	Width [min]	Area [mAU*s]	Height [mAU]	Area %
1	6.920	BB	0.2342	2963.85107	194.04907	50.0855
2	9.547	BB	0.3526	2953.72998	129.25456	49.9145

Enantioselective sample: ***trans*-S2**

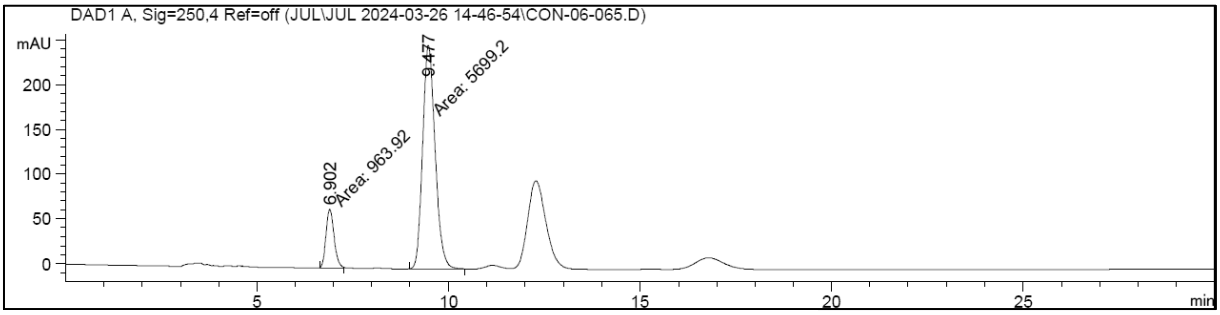

Signal 1: DAD1 A, Sig=250,4 Ref=off

Peak #	RetTime [min]	Type	Width [min]	Area [mAU*s]	Height [mAU]	Area %
1	6.902	MF	0.2452	963.92041	65.52028	14.4665
2	9.477	MF	0.3796	5699.19824	250.22485	85.5335

Racemic sample: ***cis*-S2**

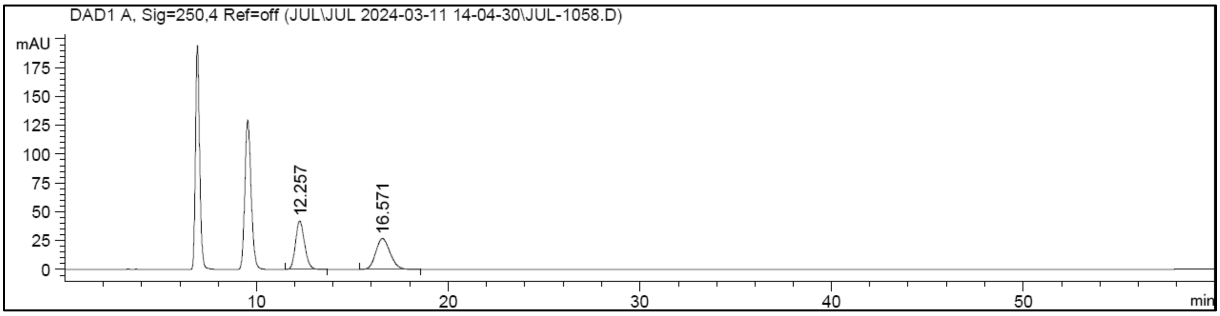

Signal 1: DAD1 A, Sig=250,4 Ref=off

Peak #	RetTime [min]	Type	Width [min]	Area [mAU*s]	Height [mAU]	Area %
1	12.257	BB	0.5066	1372.93433	41.86980	49.6986
2	16.571	BB	0.7977	1389.58838	26.76671	50.3014

Enantioselective sample: ***cis*-S2**

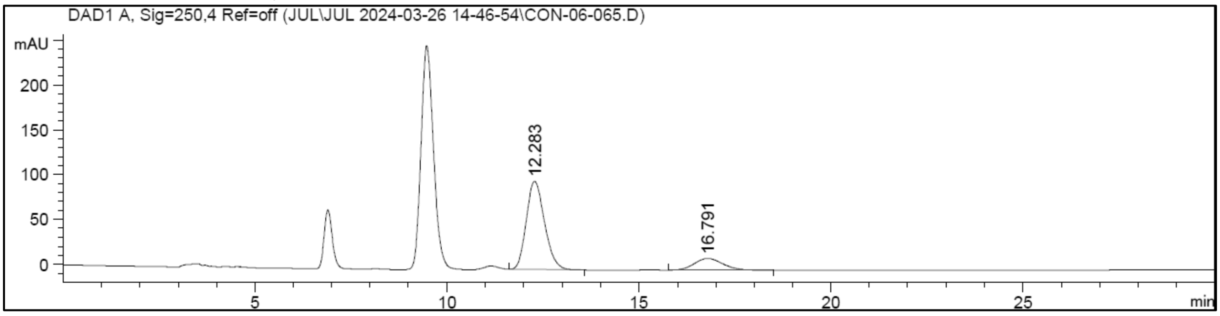

Signal 1: DAD1 A, Sig=250,4 Ref=off

Peak #	RetTime [min]	Type	Width [min]	Area [mAU*s]	Height [mAU]	Area %
1	12.283	BB	0.5121	3251.94312	98.26451	83.0631
2	16.791	BB	0.7842	663.08594	12.93167	16.9369

2-(Methylthio)-6a,7,8,9,10,10a-hexahydrophenanthridin-6(5H)-one (S3)

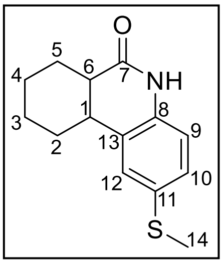

According to General Procedure **D**, **S33** (24.7 mg, 0.10 mmol, 1.00 eq.) was converted to **S3** yielding the product as white solid (14.7 mg, 60%) after purification by column chromatography (SiO_2, 18 – 40% EtOAc in *n*-pentane). The product was obtained as an inseparable mixture of diastereomers (d.r. 79:21 (*trans*:*cis*)).

R*f* = 0.18 (20% EtOAc in *n*-pentane, both diastereomers).

HR-ESI-MS: *m/z*: 270.09185 ([*M*+Na]$^+$, calcd. for $C_{14}H_{17}NOSNa^+$: 270.09231).

trans-diastereomer:

^{1}H NMR (600 MHz, $CDCl_3$): δ = 8.23 (s, 1H, NH), 7.20 (t, *J* = 1.7 Hz, 1H, H12), 7.14 (ddd, *J* = 8.1, 2.1, 0.9 Hz, 1H, H10), 6.73 (d, *J* = 8.1 Hz, 1H, H9), 2.63 – 2.56 (m, 1H, H1), 2.50 – 2.48 (s, 1H, H2), 2.47 (s, 3H, H14), 2.45 – 2.37 (m, 1H, H5), 2.05 (ddd, *J* = 14.0, 11.6, 3.8 Hz, 1H, H6), 1.99 – 1.93 (m, 2H, H3, H4), 1.37 (dddd, J = 16.2, 14.6, 8.4, 3.4 Hz, 3H, H2, H3, H5), 1.32 – 1.26 (m, 1H, H4) ppm.

^{13}C NMR (151 MHz, $CDCl_3$): δ = 173.4 (C7), 134.9 (C8), 132.1 (C13), 129.3 (C11), 127.2 (C10), 124.8 (C12), 115.8 (C9), 43.3 (C6), 38.1 (C1), 28.8 (C2), 26.3 (C5), 25.32 (C4), 25.30 (C3), 17.6 (C14) ppm.

e.r.: 78:22.

cis-diastereomer:

^{1}H NMR (600 MHz, $CDCl_3$): δ = 8.23 (s, 1H, NH), 7.12 – 7.08 (m, 2H, H10, H12), 6.71 – 6.68 (m, 1H, H9), 2.97 – 2.89 (m, 1H, H1), 2.82 – 2.75 (m, 1H, H6), 2.46 (s, 3H, H14), 2.37 – 2.28 (m, 1H, H5), 1.94 – 1.91 (m, 1H, H3), 1.73 (d, *J* = 12.5 Hz, 1H, H3), 1.69 – 1.63 (m, 1H, H2), 1.63 – 1.56 (m, 2H, H2, H4), 1.52 – 1.47 (m, 2H, H4, H5) ppm.

^{13}C NMR (151 MHz, $CDCl_3$): δ = 172.8 (C7), 134.4 (C8), 132.2 (C13), 129.3 (C11), 127.2 (C12), 127.1 (C10), 115.9 (C9), 40.7 (C6), 39.2 (C1), 29.8 (C2), 25.2 (C3) 24.6 (C5), 22.8 (C4), 17.4 (C14) ppm.

e.r.: 80:20.

HPLC: AS-H column; eluent: *n*-hexane/ *i*-propanol 15:85; flow rate: 1.0 mL/min.

Racemic sample: ***trans*-S3**

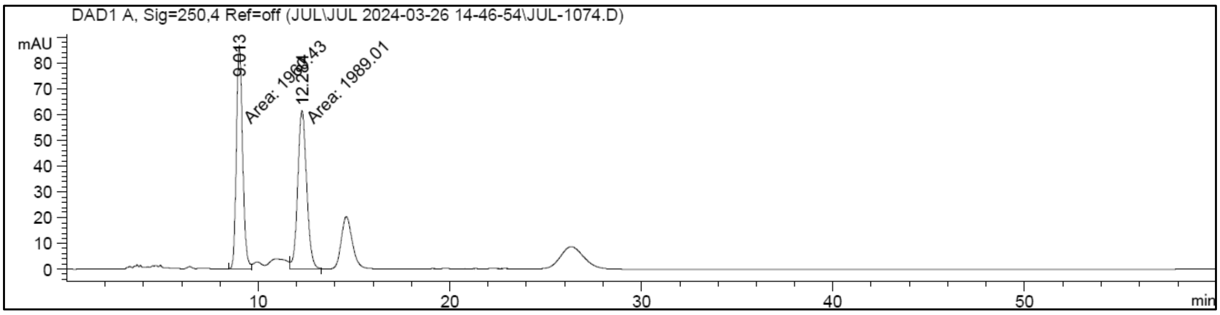

Signal 1: DAD1 A, Sig=250,4 Ref=off

Peak #	RetTime [min]	Type	Width [min]	Area [mAU*s]	Height [mAU]	Area %
1	9.013	MF	0.3771	1960.43176	86.63654	49.6383
2	12.284	FM	0.5397	1989.00513	61.42371	50.3617

Enantioselective sample: ***trans*-S3**

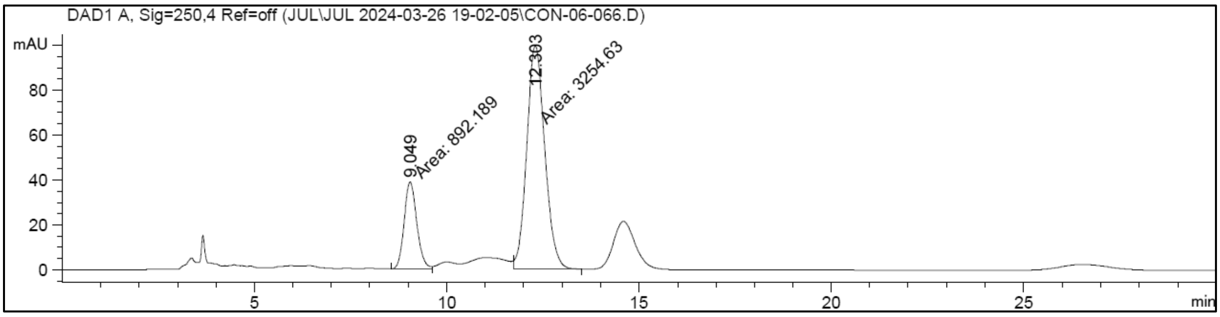

Signal 1: DAD1 A, Sig=250,4 Ref=off

Peak #	RetTime [min]	Type	Width [min]	Area [mAU*s]	Height [mAU]	Area %
1	9.049	MF	0.3837	892.18903	38.75608	21.5150
2	12.303	FM	0.5450	3254.62842	99.52350	78.4850

Racemic sample: ***cis*-S3**

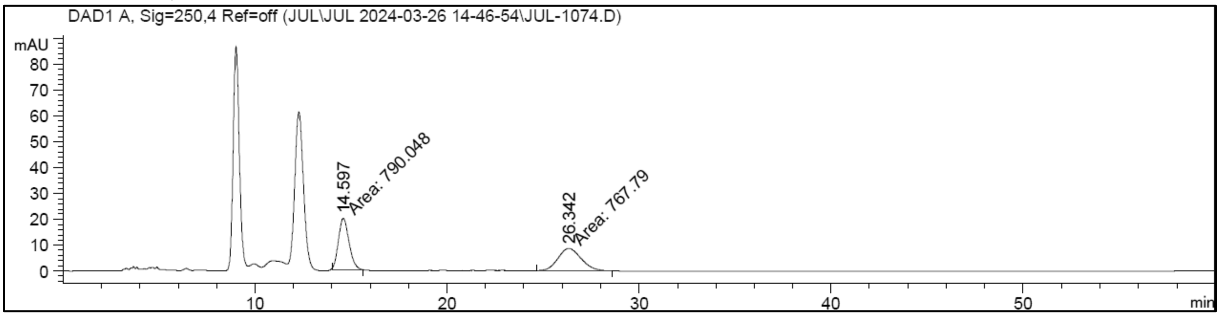

Signal 1: DAD1 A, Sig=250,4 Ref=off

Peak #	RetTime [min]	Type	Width [min]	Area [mAU*s]	Height [mAU]	Area %
1	14.597	FM	0.6541	790.04779	20.13069	50.7144
2	26.342	MM	1.4548	767.79004	8.79599	49.2856

Enantioselective sample: ***cis*-S3**

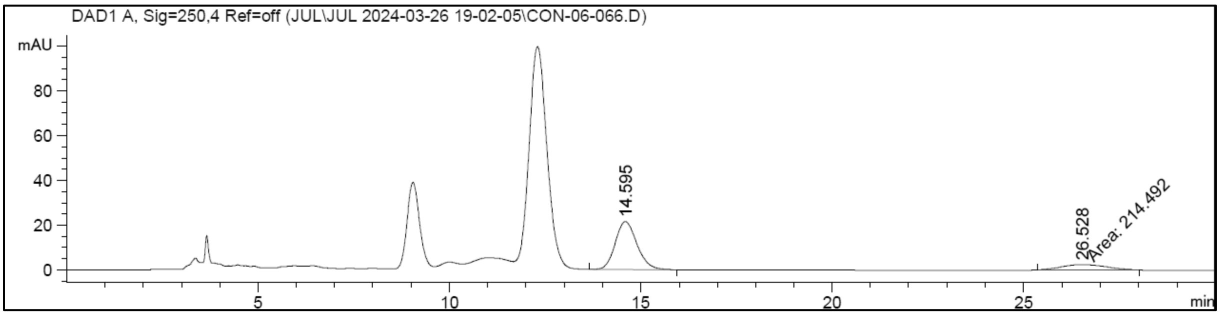

Signal 1: DAD1 A, Sig=250,4 Ref=off

Peak #	RetTime [min]	Type	Width [min]	Area [mAU*s]	Height [mAU]	Area %
1	14.595	BB	0.6058	844.85999	21.43478	79.7525
2	26.528	MF	1.3820	214.49220	2.58665	20.2475

Methyl 6-oxo-5,6,6a,7,8,9,10,10a-octahydrophenanthridine-2-carboxylate (S4)

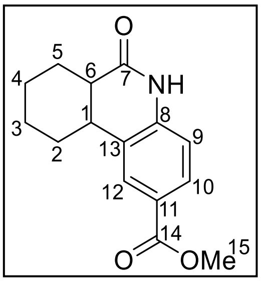

According to General Procedure **D**, **S34** (25.9 mg, 0.10 mmol, 1.00 eq.) was converted to **S4** using HFIP instead of *n*Bu_4NCl yielding the product as white solid (25.8 mg, quant.) after purification by column chromatography (SiO_2, 30% EtOAc in *n*-pentane). The product was obtained as an inseparable mixture of diastereomers (d.r. 58:42 (*trans*:*cis*)).

R*f* = 0.32 (30% EtOAc in *n*-pentane, both diastereomers).

HR-ESI-MS: *m/z*: 282.10993 ([*M*+Na]$^+$, calcd. for $C_{15}H_{17}NO_3Na^+$: 282.11006).

trans-diastereomer:

^{1}H NMR (600 MHz, $CDCl_3$): δ = 8.95 (s, 1H, NH), 7.92 (t, *J* = 1.6 Hz, 1H, H12), 7.89 (ddd, *J* = 8.2, 1.9, 0.8 Hz, 1H, H10), 6.86 (d, *J* = 8.2 Hz, 1H, H9), 3.90 (s, 3H, H15), 2.62 (dd, *J* = 14.1, 10.7 Hz, 1H, H1), 2.59 – 2.54 (m, 1H, H5), 2.43 (dt, *J* = 13.3, 2.5 Hz, 1H, H2), 2.08 (ddd, *J* = 14.0, 11.6, 3.8 Hz, 1H, H6), 2.00 – 1.93 (m, 2H, H3, H4), 1.43 – 1.37 (m, 3H, H2, H3/H4, H5), 1.34 – 1.28 (m, 1H, H3/H4) ppm.

^{13}C NMR (151 MHz, $CDCl_3$): δ = 174.0 (C7), 167.0 (C14), 140.9 (C8), 129.6 (C10), 128.2 (C13), 126.3 (C12), 124.9 (11), 115.1 (C9), 52.2 (C15), 43.3 (C6), 37.9 (C1), 28.8 (C5), 26.2 (C2), 25.3 (C3/C4), 25.2 (C3/C4) ppm.

e.r.: 86:14.

cis-diastereomer:

^{1}H NMR (600 MHz, $CDCl_3$): δ = 8.97 (s, 1H, NH), 7.87 – 7.85 (m, 2H, H10, H12), 6.84 (d, *J* = 8.7 Hz, 1H, H9), 3.89 (s, 3H, H15), 3.02 (dt, *J* = 10.2, 5.1 Hz, 1H, H1), 2.82 (dd, *J* = 4.8, 4.4 Hz, 1H, H6), 2.32 (s, 1H, H5), 1.75 – 1.71 (m, 1H, H3), 1.70 – 1.66 (m, 1H, H2), 1.63 – 1.55 (d, *J* = 12.3 Hz, 2H, H2, H4), 1.54 – 1.50 (m, 1H, H5), 1.49 – 1.44 (m, 2H, H3, H4) ppm.

^{13}C NMR (151 MHz, $CDCl_3$): δ = 173.5 (C7), 166.8 (C14), 140.6 (C8), 129.5 (C10), 129.2 (C12), 128.2 (C13), 125.0 (C11), 115.2 (C9), 52.1 (C15), 40.7 (C6), 38.8 (C1), 29.8 (C2), 25.1 (C3), 24.5 (C5), 22.8 (C4) ppm.

e.r.: 83:17.

HPLC: AS-H column; eluent: *n*-hexane/ *i*-propanol 15:85; flow rate: 1.0 mL/min.

Racemic sample: ***trans*-S4**

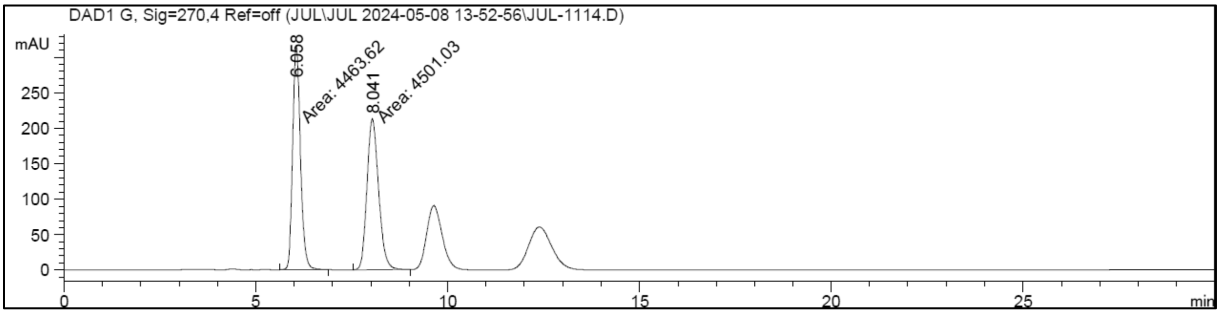

Signal 4: DAD1 G, Sig=270,4 Ref=off

Peak #	RetTime [min]	Type	Width [min]	Area [mAU*s]	Height [mAU]	Area %
1	6.058	FM	0.2352	4463.62109	316.30106	49.7913
2	8.041	FM	0.3523	4501.03467	212.91843	50.2087

Enantioselective sample: ***trans*-S4**

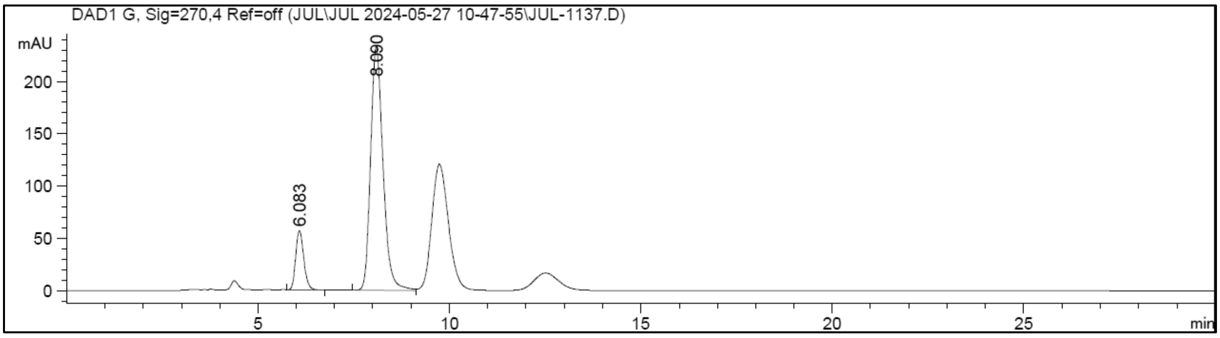

Signal 5: DAD1 G, Sig=270,4 Ref=off

Peak #	RetTime [min]	Type	Width [min]	Area [mAU*s]	Height [mAU]	Area %
1	6.083	BB	0.2243	829.35333	56.80783	13.6451
2	8.090	BV	0.3444	5248.66748	233.38246	86.3549

Racemic sample: ***cis*-S4**

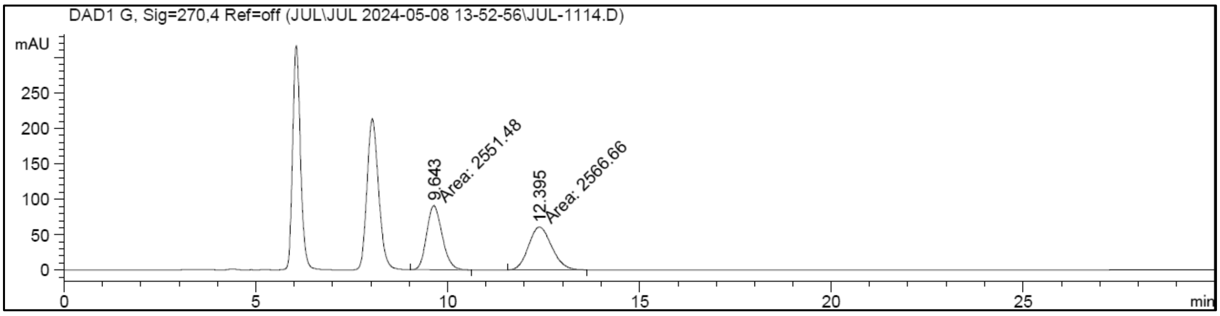

Signal 4: DAD1 G, Sig=270,4 Ref=off

Peak #	RetTime [min]	Type	Width [min]	Area [mAU*s]	Height [mAU]	Area %
1	9.643	MF	0.4702	2551.47754	90.44674	49.8517
2	12.395	FM	0.7057	2566.65601	60.61943	50.1483

Enantioselective sample: ***cis*-S4**

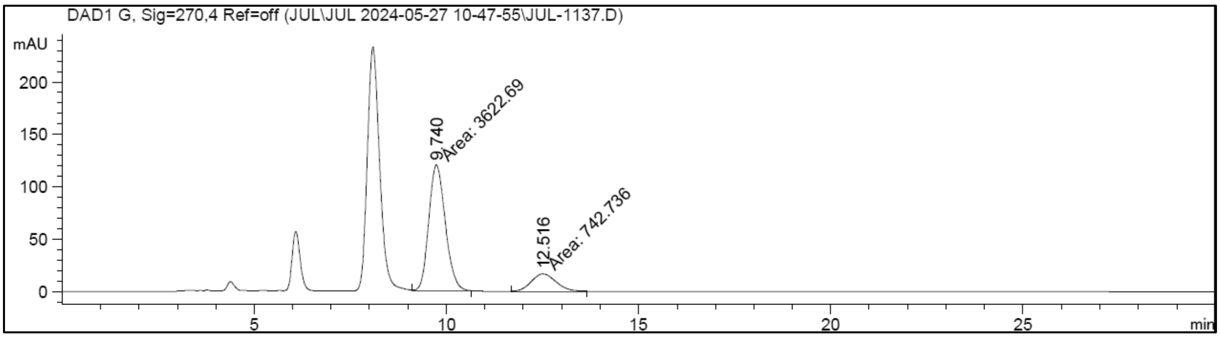

Signal 5: DAD1 G, Sig=270,4 Ref=off

Peak #	RetTime [min]	Type	Width [min]	Area [mAU*s]	Height [mAU]	Area %
1	9.740	MM	0.5009	3622.68652	120.53815	82.9859
2	12.516	MM	0.7430	742.73578	16.65977	17.0141

4-Fluoro-6a,7,8,9,10,10a-hexahydrophenanthridin-6(5*H*)-one (S5)

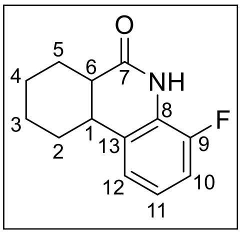

According to General Procedure **D**, **S35** (21.9 mg, 0.10 mmol, 1.00 eq.) was converted to **S5** using HFIP instead of *n*Bu_4NCl yielding the product as white solid (5.9 mg, 27%) after purification by column chromatography (SiO_2, 10 % EtOAc in *n*-pentane). The product was obtained as an inseparable mixture of diastereomers (d.r. 58:42 (*trans*:*cis*)).

R*f* = 0.29 (20% EtOAc in *n*-pentane, both diastereomers).

trans-diastereomer:

^{1}H NMR (500 MHz, $CDCl_3$): δ = 7.72 (s, 1H, NH), 7.03 – 6.96 (m, 3H, H10, H11, H12), 2.65 (ddd, *J* = 14.4, 10.8, 3.9 Hz, 1H, H6), 2.51 – 2.46 (m, 1H, H5), 2.45 – 2.40 (m, 1H, H2), 2.09 (ddd, *J* = 14.0, 11.6, 3.7 Hz, 1H, H1), 1.98 – 1.91 (m, 2H, H3, H4), 1.41 – 1.35 (m, 3H, H2, H4, H5), 1.33 – 1.26 (m, 1H, H3) ppm.

^{13}C NMR (126 MHz, $CDCl_3$): δ = 172.2 (C7), 149.8 (d, *J* = 243.2 Hz, C9), 130.7 (d, *J* = 1.5 Hz, C13), 125.1 (d, *J* = 11.6 Hz, C8), 122.9 (d, *J* = 7.3 Hz, C11), 119.8 (d, *J* = 3.3 Hz, C12), 113.8 (d, *J* = 18.1 Hz, C10), 43.2 (C1), 38.2 (C6), 29.0 (C5), 26.2 (C2), 25.3 (2C, C3, C4) ppm.

^{19}F NMR {^{1}H} (376 MHz, $CDCl_3$): δ = –135.58 ppm.

e.r.: 74:26.

cis-diastereomer:

^{1}H NMR (500 MHz, $CDCl_3$): δ = 7.69 (s, 1H, NH), 6.96 – 6.92 (m, 3H, H10, H11, H12), 3.00 (dt, *J* = 10.5, 5.0 Hz, 1H, H1), 2.82 (q, *J* = 4.5 Hz, 1H, H6), 2.35 (s, 1H, H5), 1.76 – 1.71 (m, 1H, H3), 1.69 – 1.63 (m, 1H, H2), 1.63 – 1.56 (m, 2H, H2, H4), 1.52 – 1.42 (m, 3H, H3, H4, H5) ppm.

^{13}C NMR (126 MHz, $CDCl_3$): δ = 171.6 (C7), 149.7 (d, *J* = 243.4 Hz, C9), 130.7 (C13), 124.8 (d, *J* = 11.6 Hz, C8), 123.1 (d, *J* = 7.4 Hz, C11), 122.6 (d, *J* = 3.1 Hz, C12), 113.7 (d, *J* = 18.1 Hz, C10), 40.7 (C6), 39.2 (C1), 29.8 (C2), 25.3 (C3), 24.6 (C5), 22.7 (C4) ppm. C3 and C13 were assigned via 2D NMR.

^{19}F NMR {^{1}H} (376 MHz, $CDCl_3$): δ = –135.26 ppm.

e.r.: 72:28.

HPLC: AS-H column; eluent: *n*-hexane/ *i*-propanol 95:5; flow rate: 0.9 mL/min.

Racemic sample: ***trans*-S5**

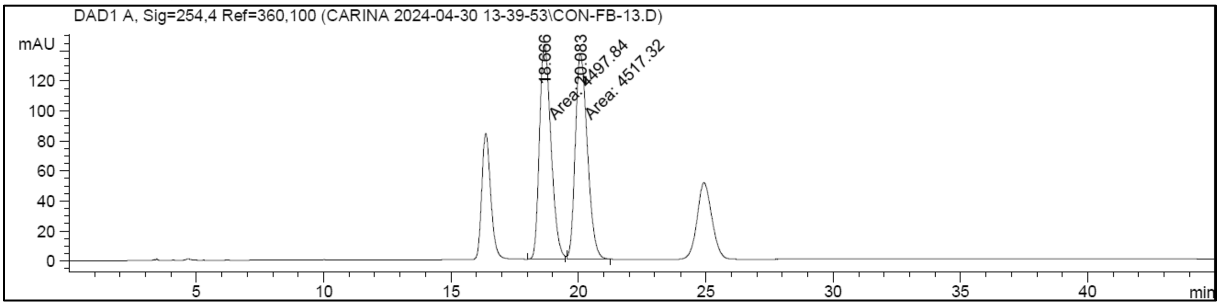

Signal 1: DAD1 A, Sig=254,4 Ref=360,100

Peak #	RetTime [min]	Type	Width [min]	Area [mAU*s]	Height [mAU]	Area %
1	18.666	MF	0.5250	4497.84424	142.78864	49.8920
2	20.083	FM	0.5502	4517.31592	136.83813	50.1080

Enantioselective sample: ***trans*-S5**

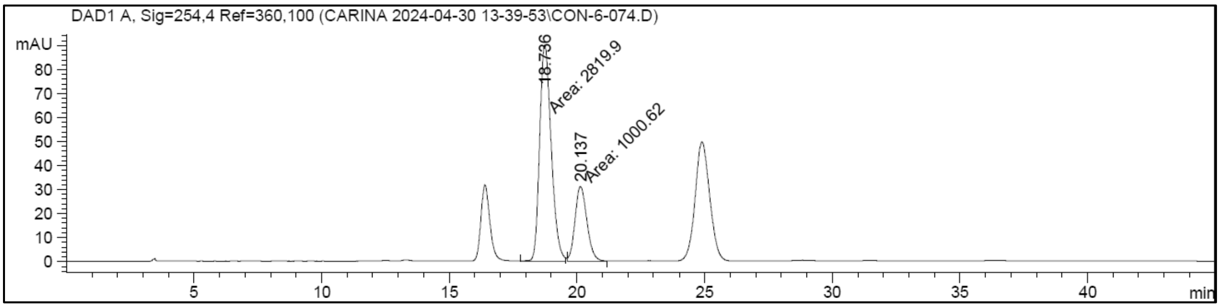

Signal 1: DAD1 A, Sig=254,4 Ref=360,100

Peak #	RetTime [min]	Type	Width [min]	Area [mAU*s]	Height [mAU]	Area %
1	18.736	MF	0.5218	2819.90283	90.07449	73.8093
2	20.137	MF	0.5368	1000.62384	31.06736	26.1907

Racemic sample: ***cis*-S5**

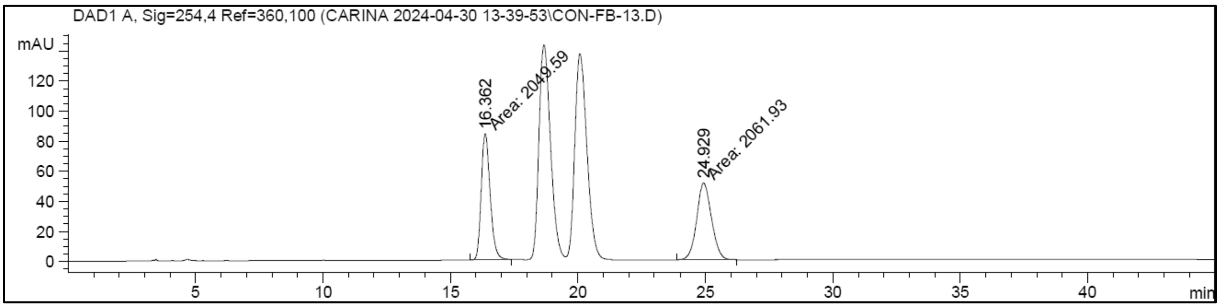

Signal 1: DAD1 A, Sig=254,4 Ref=360,100

Peak #	RetTime [min]	Type	Width [min]	Area [mAU*s]	Height [mAU]	Area %
1	16.362	MM	0.4063	2049.59326	84.07039	49.8499
2	24.929	MM	0.6721	2061.93262	51.13045	50.1501

Enantioselective sample: ***cis*-S5**

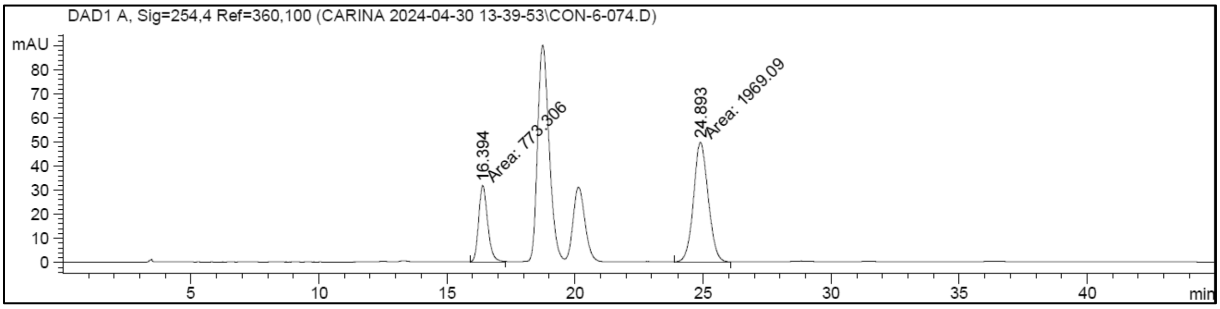

Signal 1: DAD1 A, Sig=254,4 Ref=360,100

Peak #	RetTime [min]	Type	Width [min]	Area [mAU*s]	Height [mAU]	Area %
1	16.394	MM	0.4070	773.30591	31.66457	28.1982
2	24.893	MM	0.6607	1969.08728	49.66834	71.8018

1,3-Difluoro-6a,7,8,9,10,10a-hexahydrophenanthridin-6(5*H*)-one (S6)

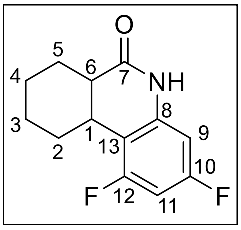

According to General Procedure **E**, **S36** (23.7 mg, 0.10 mmol, 1.00 eq.) was converted to **S6** using HFIP instead of *n*Bu_4NCl yielding the product after 65 h as a white solid (19.7 mg, 83%) after purification by column chromatography (SiO_2, 12 % EtOAc in *n*-pentane). The product was obtained as s mixture of diastereomers (d.r. 75:25 (*trans*:*cis*)).

HR-ESI-MS: *m/z*: 260.08557 ($[M+Na]^+$, calcd. for $C_{13}H_{13}NOF_2Na^+$: 260.08574).

trans-diastereomer:

R*f* = 0.68 (30% EtOAc in *n*-pentane).

^{1}H NMR (500 MHz, $CDCl_3$): δ = δ 8.56 (s, 1H, NH), 6.48 (td, *J* = 9.2, 2.4 Hz, 1H, H9), 6.35 (dt, *J* = 9.1, 1.9 Hz, 1H, H11), 3.21 (ddd, *J* = 12.2, 5.9, 4.0 Hz, 1H, H1), 2.79 – 2.75 (m, 1H, H6), 2.59 – 2.52 (m, 1H, H5), 1.82 – 1.76 (m, 1H, H3), 1.75 – 1.69 (m, 1H, H2), 1.68 – 1.63 (m, 1H, H4), 1.53 – 1.38 (m, 3H, H3, H4, H5), 1.34 – 1.27 (m, 1H, H2) ppm.

^{13}C NMR {^{19}F} (126 MHz, $CDCl_3$): δ = 172.7 (C7), 162.0 (C10), 159.9 (C12), 138.7 (C8), 112.3 (C13), 98.7 (C11), 98.3 (C9), 39.7 (C6), 32.7 (C1), 29.1 (C2), 25.9 (C3), 24.6 (C5), 22.2 (C4) ppm.

^{19}F NMR {^{1}H} (470 MHz, $CDCl_3$): δ = –111.61 (d, *J* = 7.2 Hz), –117.04 (d, *J* = 7.2 Hz) ppm.

e.r.: 74:26.

cis-diastereomer:

R*f* = 0.59 (30% EtOAc in *n*-pentane);

^{1}H NMR (500 MHz, $CDCl_3$): δ = 8.47 (s, 1H, NH), 6.45 (ddd, *J* = 12.3, 9.0, 2.6 Hz, 1H, H9), 6.35 (ddd, *J* = 8.8, 2.5, 1.4 Hz, 1H, H11), 2.90 – 2.83 (m, 1H, H2), 2.81 – 2.73 (m, 1H, H1), 2.43 (d, *J* = 12.5 Hz, 1H, H5), 2.16 (ddd, *J* = 13.7, 11.1, 3.6 Hz, 1H, H6), 1.93 – 1.87 (m, 2H, H3, H4), 1.62 – 1.53 (m, 1H, H2), 1.39 – 1.27 (m, 3H, H3, H4, H5) ppm.

^{13}C NMR {^{19}F} (126 MHz, $CDCl_3$): δ = 173.3 (C7), 161.8 (C10/C12), 161.7 (C10/C12), 139.7 (C8), 111.0 (C13), 99.2 (2C, C9, C11), 43.4 (C6), 38.1 (C1), 30.0 (C2), 26.3 (C5), 25.8 (C3/C4), 25.2 (C3/C4) ppm.

^{19}F NMR {^{1}H} (470 MHz, $CDCl_3$): δ = –108.93 (d, *J* = 8.1 Hz), –111.99 (d, *J* = 8.1 Hz) ppm.

e.r.: 78:22.

HPLC: AS-H column; eluent: *n*-hexane/ *i*-propanol 93:7; flow rate: 0.7 mL/min.

Racemic sample: ***trans*-S6**

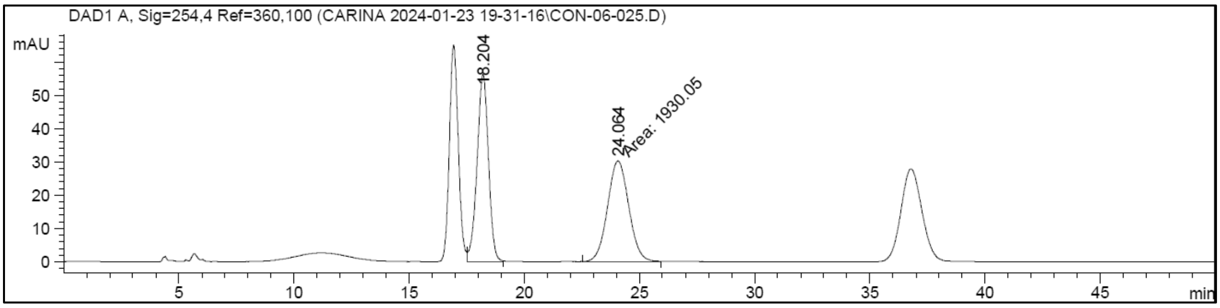

Signal 1: DAD1 A, Sig=254,4 Ref=360,100

Peak #	RetTime [min]	Type	Width [min]	Area [mAU*s]	Height [mAU]	Area %
1	18.204	VB	0.5247	1928.98340	56.16932	49.9862
2	24.064	MM	1.0650	1930.04932	30.20432	50.0138

Enantioselective sample: ***trans*-S6**

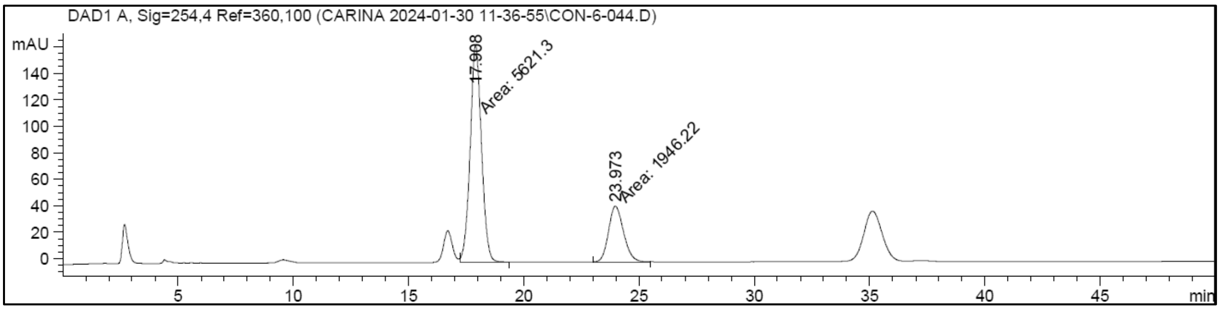

Signal 1: DAD1 A, Sig=254,4 Ref=360,100

Peak #	RetTime [min]	Type	Width [min]	Area [mAU*s]	Height [mAU]	Area %
1	17.908	FM	0.5701	5621.29883	164.32956	74.2819
2	23.973	FM	0.7655	1946.21875	42.37196	25.7181

Racemic sample: ***cis*-S6**

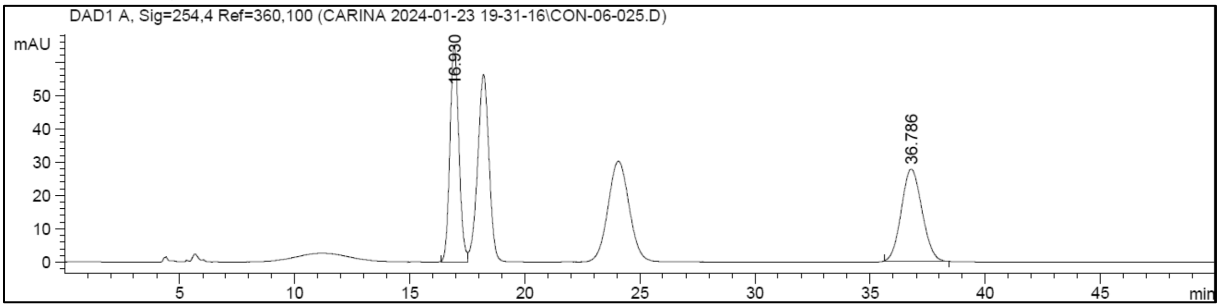

Signal 1: DAD1 A, Sig=254,4 Ref=360,100

Peak #	RetTime [min]	Type	Width [min]	Area [mAU*s]	Height [mAU]	Area %
1	16.930	BV	0.4187	1754.94653	65.06829	50.4756
2	36.786	BB	0.9548	1721.87195	27.63849	49.5244

Enantioselective sample: ***cis*-S6**

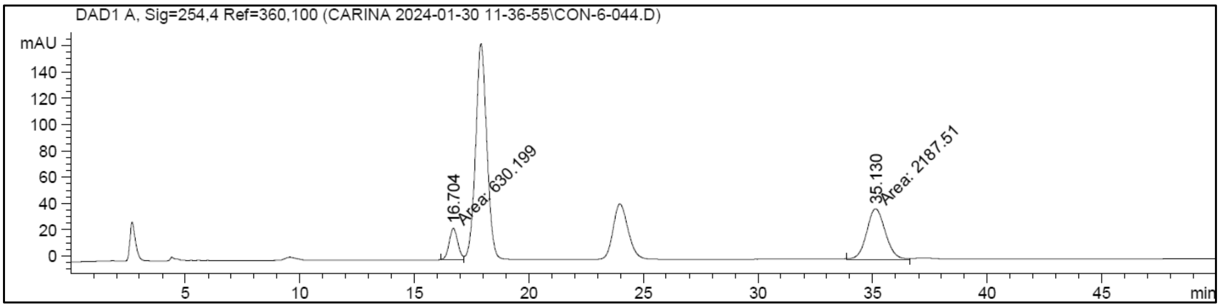

Signal 1: DAD1 A, Sig=254,4 Ref=360,100

Peak #	RetTime [min]	Type	Width [min]	Area [mAU*s]	Height [mAU]	Area %
1	16.704	FM	0.4398	630.19916	23.88440	22.3657
2	35.130	MM	0.9462	2187.51025	38.53017	77.6343

3-Chloro-6a,7,8,9,10,10a-hexahydrophenanthridin-6(5*H*)-one (S7)

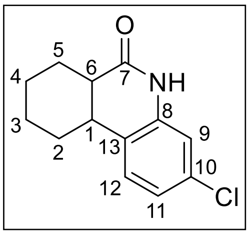

According to General Procedure **D**, **S37** (23.6 mg, 0.10 mmol, 1.00 eq.) was converted to **S7** yielding the product as a white solid (7.8 mg, 33%) after purification by column chromatography (SiO_2, 15 % EtOAc in *n*-pentane). The regioisomers were separated by column chromatography and the 3-chloro isomer was observed as the main product (r.r. 54:46 (3-Cl:1-Cl).

HR-ESI-MS: *m/z*: 258.06561 ([*M*+Na]$^+$, calcd. for $C_{13}H_{14}NOClNa^+$: 258.06554).

3-Cl regioisomer:

The products were obtained as an inseparable mixture of diastereomers (d.r. 68:32 (*trans*:*cis*)).

R*f* = 0.30 (20% EtOAc in *n*-pentane, 3-Cl, both diastereomers).

trans-diastereomer:

^{1}H NMR (500 MHz, $CDCl_3$): δ = 8.23 (s, 1H, NH), 7.14 (dd, *J* = 8.2, 1.2 Hz, 1H, H12), 7.00 (dd, *J* = 8.2, 2.1 Hz, 1H, H11), 6.79 (d, *J* = 2.1 Hz, 1H, H9), 2.60 – 2.55 (m, 1H, H1), 2.47 – 2.39 (m, 2H, H2, H5), 2.05 (ddd, *J* = 14.1, 11.6, 3.8 Hz, 1H, H6), 1.98 – 1.92 (m, 2H, H3, H4), 1.42 – 1.34 (m, 3H, H2, H3/H4, H5), 1.30 (tt, *J* = 12.5, 3.4 Hz, 1H, H3/H4) ppm.

^{13}C NMR (151 MHz, $CDCl_3$): δ = 173.5 (C7), 137.9 (C8), 133.0 (C13), 127.0 (C10), 125.8 (C12), 123.0 (C11), 115.3 (C9), 43.3 (C6), 37.7 (C1), 28.9 (C2), 26.2 (C5), 25.27 (C3/C4), 25.25 (C3/C4) ppm.

e.r.: 82:18.

cis-diastereomer:

^{1}H NMR (600 MHz, $CDCl_3$): δ = 8.23 (s, 1H, NH), 7.07 (d, *J* = 8.1 Hz, 1H, H12), 6.96 (dd, *J* = 8.0, 2.0 Hz, 1H, H11), 6.77 (d, *J* = 2.1 Hz, 1H, H9), 2.94 (dt, *J* = 10.4, 5.1 Hz, 1H, H1), 2.79 (t, *J* = 5.0 Hz, 1H, H6), 2.33 (s, 1H, H5), 1.75 – 1.70 (m, 1H, H3), 1.67 – 1.57 (m, 3H, H2, H4), 1.56 – 1.44 (m, 3H, H3, H4, H5) ppm.

^{13}C NMR (151 MHz, $CDCl_3$): δ = 172.9 (C7), 137.6 (C8), 132.9 (C13), 128.6 (C12), 127.0 (C10), 123.2 (C11), 115.4 (C9), 40.7 (C6), 38.7 (C1), 29.9 (C2), 25.2 (C3), 24.6 (C5), 22.8 (C4) ppm.

e.r.: 92:8.

HPLC: AS-H column; eluent: *n*-hexane/ *i*-propanol 80:20; flow rate: 1.0 mL/min.

Racemic sample: ***trans*-S7**

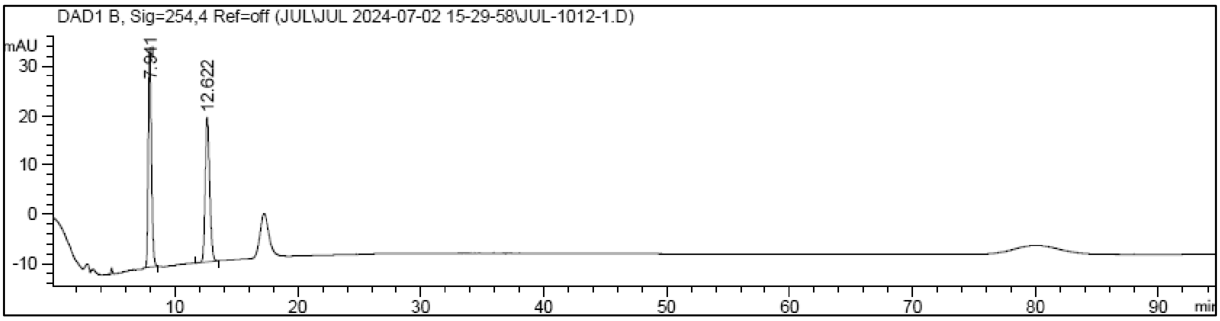

Signal 2: DAD1 B, Sig=254,4 Ref=off

Peak #	RetTime [min]	Type	Width [min]	Area [mAU*s]	Height [mAU]	Area %
1	7.941	BB	0.2644	772.47821	44.52210	50.2489
2	12.622	BB	0.4049	764.82489	29.45592	49.7511

Enantioselective sample: ***trans*-S7**

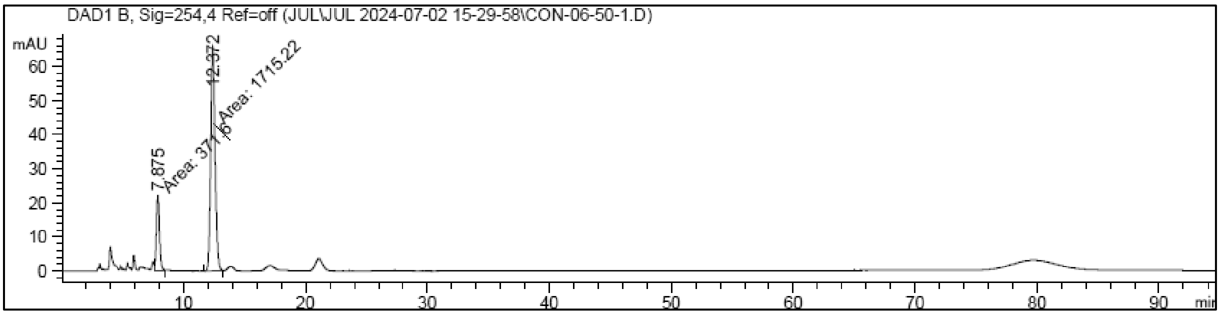

Signal 2: DAD1 B, Sig=254,4 Ref=off

Peak #	RetTime [min]	Type	Width [min]	Area [mAU*s]	Height [mAU]	Area %
1	7.875	FM	0.2829	371.59958	21.89338	17.8070
2	12.372	MM	0.4329	1715.21997	66.03319	82.1930

Racemic sample: ***cis*-S7**

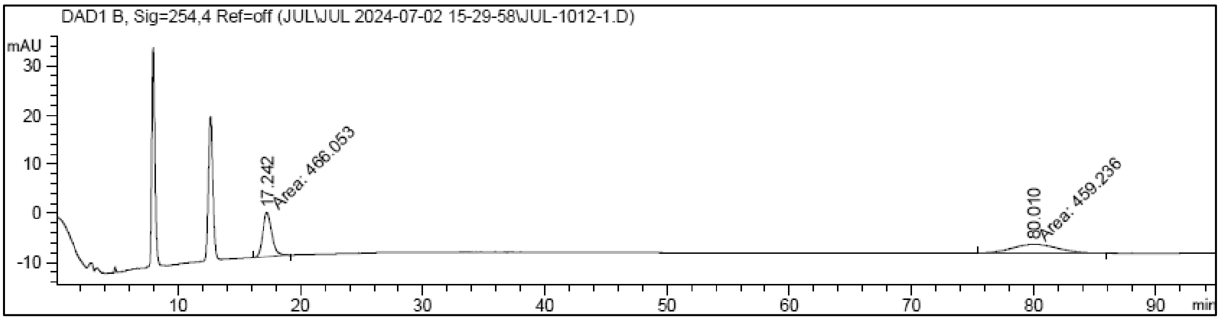

Signal 2: DAD1 B, Sig=254,4 Ref=off

Peak #	RetTime [min]	Type	Width [min]	Area [mAU*s]	Height [mAU]	Area %
1	17.242	MM	0.8621	466.05283	9.01049	50.3683
2	80.010	MM	4.2832	459.23630	1.78698	49.6317

Enantioselective sample: ***cis*-S7**

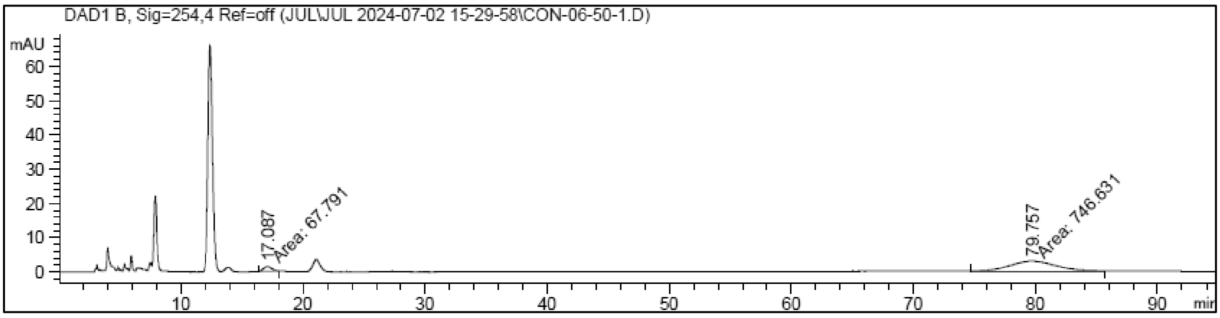

Signal 2: DAD1 B, Sig=254,4 Ref=off

Peak #	RetTime [min]	Type	Width [min]	Area [mAU*s]	Height [mAU]	Area %
1	17.087	MM	0.7784	67.79105	1.45144	8.3238
2	79.757	MM	4.3073	746.63135	2.88900	91.6762

1-Cl regioisomer:

The products were obtained as an inseparable mixture of diastereomers (d.r. 77:23).

R*f* = 0.23 (20% EtOAc in *n*-pentane, 1-Cl, both diastereomers).

major diastereomer:

^{1}H NMR (600 MHz, $CDCl_3$): δ = 8.28 (s, 1H, NH), 7.06 (td, *J* = 7.9, 0.9 Hz, 1H, H10), 7.01 (dd, *J* = 8.1, 1.4 Hz, 1H, H11), 6.68 – 6.66 (m, 1H, H9), 3.50 (dd, *J* = 13.4, 3.7 Hz, 1H, H5), 2.90 (td, *J* = 12.5, 3.7 Hz, 1H, H6), 2.48 (dt, *J* = 13.5, 2.6 Hz, 1H, H2), 2.24 (ddd, *J* = 11.0, 3.5, 1.9 Hz, 1H, H1), 1.96 – 1.92 (m, 1H, H4), 1.90 – 1.86 (m, 1H, H3), 1.57 – 1.53 (m, 1H, H5), 1.39 (dd, *J* = 10.8, 2.6 Hz, 1H, H2), 1.34 – 1.30 (m, 2H, H3, H4) ppm.

^{13}C NMR (151 MHz, $CDCl_3$): δ = 172.4 (C7), 137.7 (C8), 133.0 (C13), 128.2 (C10), 126.9 (C12), 124.1 (C11), 113.9 (C9), 39.7 (C1), 37.2 (C6), 27.5 (C5), 26.1 (C4), 24.6 (C2), 22.3 (C3) ppm.

e.r.: 82:18

minor diastereomer:

^{1}H NMR (600 MHz, $CDCl_3$): δ = 8.28 (s, 1H), 7.08 – 7.05 (m, 1H), 7.02 – 6.99 (m, 1H), 6.68 – 6.66 (m, 1H), 3.50 (dd, *J* = 13.4, 3.7 Hz, 1H), 2.90 (td, *J* = 12.5, 3.7 Hz, 1H), 2.48 (dt, *J* = 13.5, 2.6 Hz, 1H), 2.28 – 2.21 (m, 1H), 1.98 – 1.86 (m, 2H), 1.57 (dd, *J* = 12.5, 3.4 Hz, 1H), 1.42 – 1.35 (m, 1H), 1.34 – 1.26 (m, 2H) ppm.

^{13}C NMR (151 MHz, $CDCl_3$): δ = 172.8 (C7), 139.3 (C8), 133.5 (C13), 128.2 (C10), 126.8 (C11), 125.2 (C12), 114.8 (C9), 43.6 (C1), 41.2 (C6), 30.9 (C5), 26.9 (C2), 26.2 (C4), 25.2 (C3) ppm.

e.r.: 77:23.

HPLC: AS-H column; eluent: *n*-hexane/ *i*-propanol 15:85; flow rate: 1.0 mL/min, 35 °C.

Racemic sample: ***major*-S7**

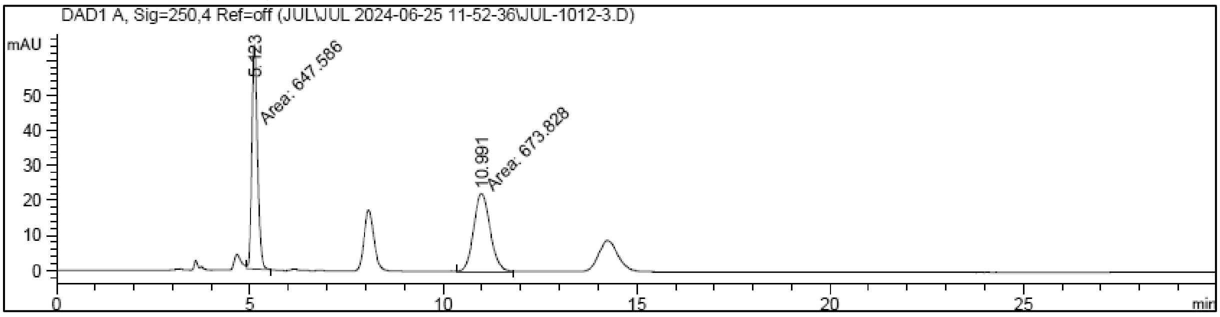

Signal 1: DAD1 A, Sig=250,4 Ref=off

Peak #	RetTime [min]	Type	Width [min]	Area [mAU*s]	Height [mAU]	Area %
1	5.123	MM	0.1687	647.58624	63.99442	49.0071
2	10.991	MM	0.4980	673.82819	22.55287	50.9929

Enantioselective sample: ***major*-S7**

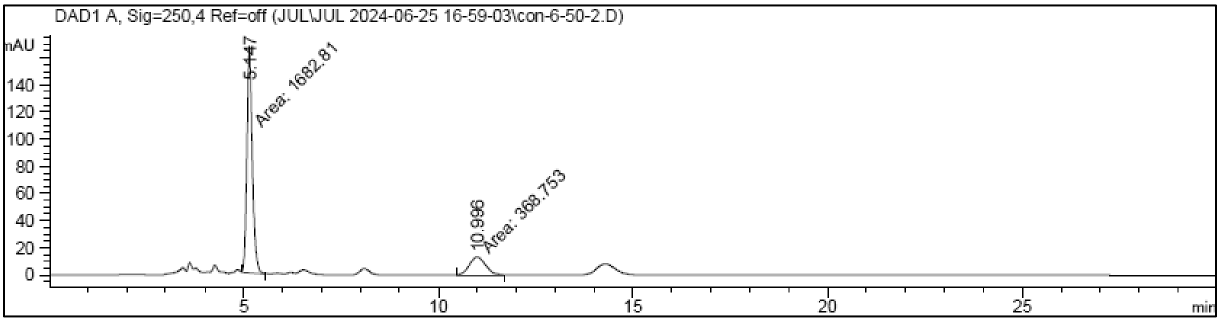

```
Signal 1: DAD1 A, Sig=250,4 Ref=off

Peak RetTime Type  Width     Area      Height     Area
  #   [min]        [min]   [mAU*s]     [mAU]        %
----|-------|----|-------|----------|----------|--------|
   1   5.147 MM    0.1688 1682.81238  166.11847  82.0258
   2  10.996 MM    0.4681  368.75275   13.12842  17.9742
```

Racemic sample: ***minor*-S7**

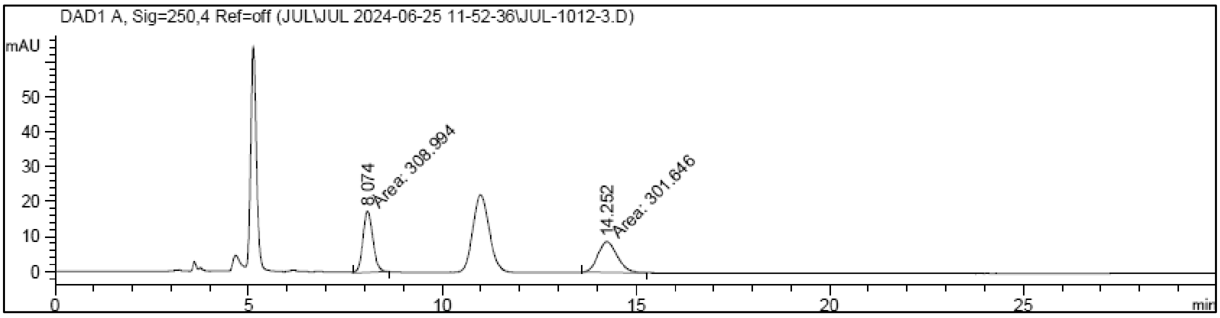


```
Signal 1: DAD1 A, Sig=250,4 Ref=off

Peak RetTime Type  Width     Area      Height     Area
  #   [min]        [min]   [mAU*s]     [mAU]        %
----|-------|----|-------|----------|----------|--------|
   1   8.074 MM    0.2941  308.99414   17.51028  50.6016
   2  14.252 MM    0.5754  301.64648    8.73668  49.3984
```

Enantioselective sample: ***minor*-S7**

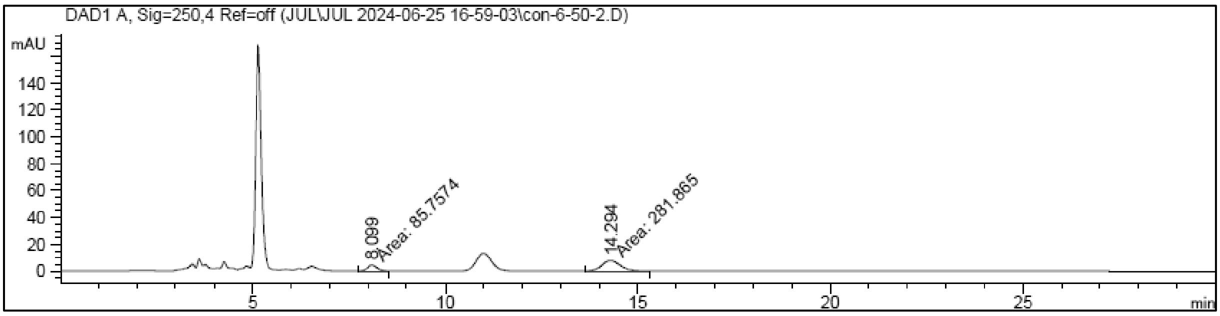


```
Signal 1: DAD1 A, Sig=250,4 Ref=off

Peak RetTime Type  Width     Area      Height     Area
  #   [min]        [min]   [mAU*s]     [mAU]        %
----|-------|----|-------|----------|----------|--------|
   1   8.099 MM    0.2986   85.75738    4.78672  23.3276
   2  14.294 MM    0.5791  281.86493    8.11238  76.6724
```

2-((Trifluoromethyl)thio)-6a,7,8,9,10,10a-hexahydrophenanthridin-6(5*H*)-one (S8)

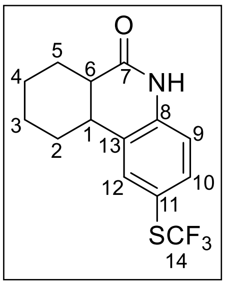

According to General Procedure **D**, **S38** (30.1 mg, 0.10 mmol, 1.00 eq.) was converted to **S8** using HFIP instead of *n*Bu_4NCl yielding the product as a white solid (18.2 mg, 50%) after purification by column chromatography (SiO_2, 20% EtOAc in *n*-pentane). The product was obtained as an inseparable mixture of diastereomers (d.r. 56:44 (*trans*:*cis*)).

R$_f$ = 0.37 (25% EtOAc in *n*-pentane, both diastereomers).

HR-ESI-MS: *m/z*: 324.06389 ([*M*+Na]$^+$, calcd. for $C_{14}H_{14}NOSF_3Na^+$: 324.06404).

trans-diastereomer:

^{1}H NMR (600 MHz, CDCl$_3$): δ = 8.79 (s, 1H, NH), 7.51 – 7.47 (m, 2H, H10, H12), 6.86 (d, *J* = 8.7 Hz, 1H, H9), 2.67 – 2.61 (m, 1H, H1), 2.53 – 2.48 (m, 1H, H2), 2.43 (dt, *J* = 13.2, 2.7 Hz, 1H, H5), 2.10 (ddd, *J* = 14.6, 11.6, 3.8 Hz, 1H, H6), 2.01 – 1.93 (m, 2H, H3, H4), 1.43 – 1.35 (m, 3H, H2, H3, H5), 1.34 – 1.29 (m, 1H, H4) ppm.

^{13}C NMR (151 MHz, CDCl$_3$): δ = 173.8 (C7), 139.4 (C8), 136.2 (C10), 133.1 (C12), 129.72 (q, *J* = 308.3 Hz, C14), 129.69 (C13), 117.8 (q, *J* = 2.2 Hz, C11), 116.2 (C9), 43.0 (C6), 37.9 (C1), 28.7 (C2), 26.2 (C5), 25.22 (C4), 25.19 (C3) ppm.

^{19}F NMR {^{1}H} (564 MHz, CDCl$_3$): δ = –43.54 ppm.

e.r.: 79:21.

cis-diastereomer:

^{1}H NMR (600 MHz, CDCl$_3$): δ = 8.82 (s, 1H, NH), 7.47 – 7.43 (m, 2H, H10, H12), 6.83 (d, *J* = 7.9 Hz, 1H; H9), 3.01 (dt, *J* = 10.5, 5.1 Hz, 1H, H1), 2.83 (q, *J* = 4.7 Hz, 1H, H6), 2.33 (s, 1H, H5), 1.76 – 1.71 (m, 1H, H3), 1.70 – 1.65 (m, 1H, H2), 1.64 – 1.58 (m, 2H, H2, H4), 1.56 – 1.44 (m, 3H, H3, H4, H5) ppm.

^{13}C NMR (151 MHz, CDCl$_3$): δ = 173.3 (C7), 139.1 (C8), 136.1 (C10), 135.8 (C12), 129.72 (q, *J* = 308.3 Hz, C14), 129.70 (C13), 117.7 (q, *J* = 2.2 Hz, C11), 116.4 (C9), 40.6 (C6), 38.8 (C1), 29.5 (C2), 25.2 (C3), 24.5 (C5), 22.8 (C4) ppm.

^{19}F NMR {^{1}H} (564 MHz, CDCl$_3$): δ = –43.55 ppm.

e.r.: 78:22.

HPLC: AS-H column; eluent: *n*-hexane/ *i*-propanol 90:10; flow rate: 1.0 mL/min.

Racemic sample: ***trans*-S8**

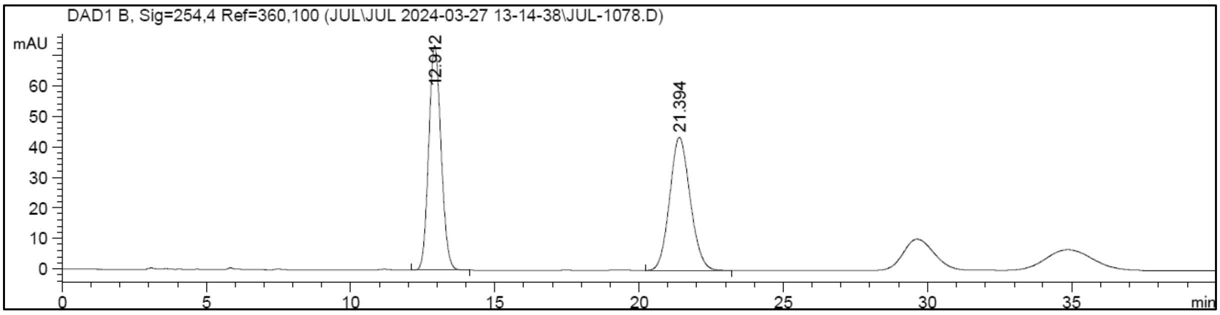

Signal 2: DAD1 B, Sig=254,4 Ref=360,100

Peak #	RetTime [min]	Type	Width [min]	Area [mAU*s]	Height [mAU]	Area %
1	12.912	BB	0.4627	2183.77979	73.49091	50.2621
2	21.394	BB	0.7526	2161.00098	43.57493	49.7379

Enantioselective sample: ***trans*-S8**

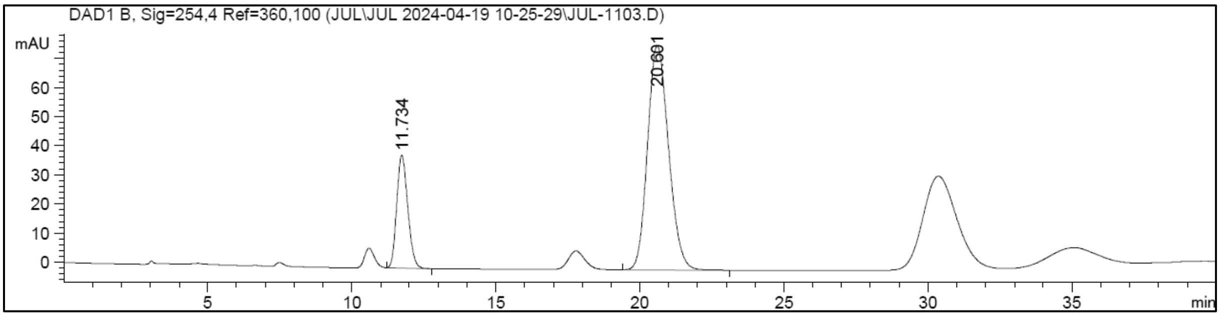

Signal 2: DAD1 B, Sig=254,4 Ref=360,100

Peak #	RetTime [min]	Type	Width [min]	Area [mAU*s]	Height [mAU]	Area %
1	11.734	BB	0.4167	1033.31262	38.79900	20.9037
2	20.601	BB	0.7875	3909.90308	77.12193	79.0963

Racemic sample: ***cis*-S8**

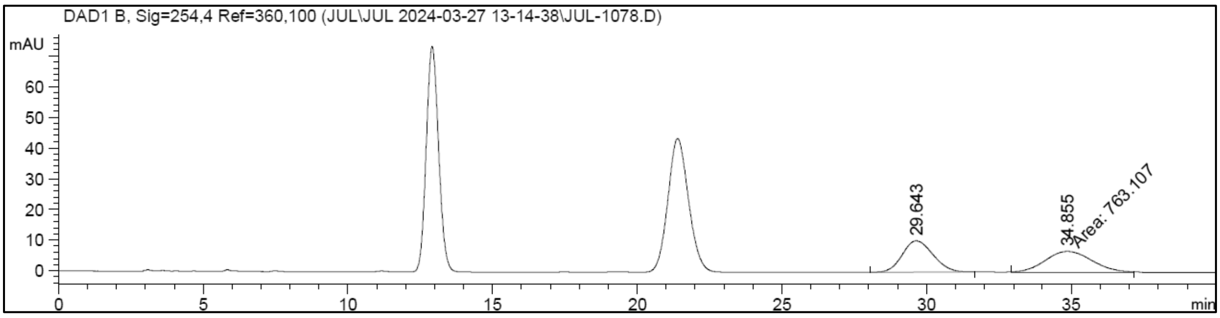

Signal 2: DAD1 B, Sig=254,4 Ref=360,100

Peak #	RetTime [min]	Type	Width [min]	Area [mAU*s]	Height [mAU]	Area %
1	29.643	BB	1.0298	750.76483	10.18665	49.5923
2	34.855	FM	1.8722	763.10748	6.79336	50.4077

Enantioselective sample: ***cis*-S8**

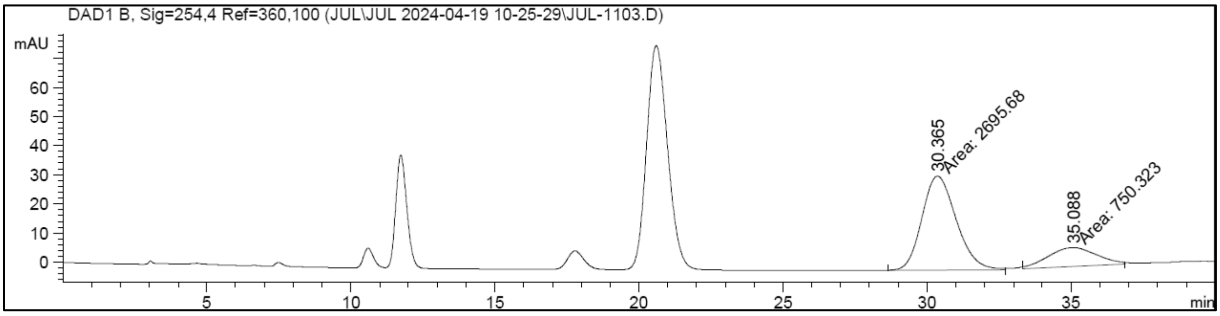

Signal 2: DAD1 B, Sig=254,4 Ref=360,100

Peak #	RetTime [min]	Type	Width [min]	Area [mAU*s]	Height [mAU]	Area %
1	30.365	MM	1.3878	2695.68115	32.37258	78.2263
2	35.088	MM	1.9476	750.32251	6.42090	21.7737

2-(Trifluoromethyl)-6a,7,8,9,10,10a-hexahydrophenanthridin-6(5*H*)-one (S9)

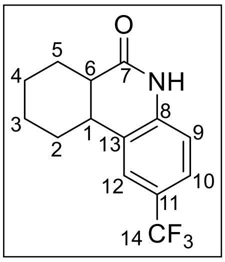

According to General Procedure **E**, **S30** (26.1 mg, 0.10 mmol, 1.00 eq.) was converted to **S9** yielding the product after 65 h as a white solid (6.3 mg, 23%) after purification by column chromatography (SiO_2, 20% EtOAc in *n*-pentane). The product was obtained as an inseparable mixture of diastereomers (d.r. 48:52 (*trans*:*cis*)).

R*f* = 0.26 (20% EtOAc in *n*-pentane, both diastereomers).

HR-ESI-MS: *m/z*: 268.09536 ([*M*-H]$^-$, calcd. for $C_{14}H_{13}NOF_3^-$: 268.09547).

trans-diastereomer:

^{1}H NMR (500 MHz, $CDCl_3$): δ = 8.48 (s, 1H, NH), 7.48 – 7.46 (m, 1H, H12), 7.44 – 7.41 (m, 1H, H10), 6.87 (d, *J* = 8.1 Hz, 1H, H9), 2.68 – 2.61 (m, 1H, H1), 2.56 – 2.50 (m, 1H, H2), 2.44 (dt, *J* = 13.1, 2.7 Hz, 1H, H5), 2.09 (ddd, *J* = 14.5, 11.5, 3.7 Hz, 1H, H6), 2.02 – 1.93 (m, 2H, H3, H4), 1.46 – 1.38 (m, 3H, H2, H3/H4, H5), 1.35 – 1.28 (m, 1H, H3/H4) ppm.

^{13}C NMR (126 MHz, $CDCl_3$): δ = 173.5 (C7), 139.8 (C8), 128.9 (C13), 125.4 (q, *J* = 32.3 Hz, C11), 124.7 (q, *J* = 3.9 Hz, C10), 124.5 (d, *J* = 271.6 Hz, C14), 121.9 (q, *J* = 3.9 Hz, C12), 115.3 (C9), 43.1 (C6), 37.9 (C1), 28.7 (C2), 26.2 (C5), 25.21 (C3/C4), 25.18 (C3/C4) ppm.

^{19}F NMR {^{1}H} (470 MHz, $CDCl_3$): δ = –61.80 ppm.

e.r.: 67:33.

cis-diastereomer:

^{1}H NMR (500 MHz, $CDCl_3$): δ = 8.48 (s, 1H, NH), 7.47 – 7.44 (m, 2H, H10, H12), 6.85 (d, *J* = 8.0 Hz, 1H, H9), 3.02 (dt, *J* = 10.5, 5.0 Hz, 1H, H1), 2.83 (dd, *J* = 4.9, 4.6 Hz, 1H, H6), 2.35 (s, 1H, H5), 1.77 – 1.73 (m, 1H, H3), 1.70 – 1.66 (m, 1H, H2), 1.64 – 1.60 (d, *J* = 6.7 Hz, 2H, H2, H4), 1.54 – 1.45 (m, 3H, H3, H4, H5) ppm.

^{13}C NMR {^{19}F} (126 MHz, $CDCl_3$): δ = 173.5 (C7) 139.4 (C8), 128.9 (C13), 125.4 (C14), 124.91 (C10/C12), 124.87 (C10/C12), 124.3 (C11), 115.3 (C9), 40.6 (C6), 39.1 (C1), 29.7 (C2), 25.2 (C3), 24.5 (C5), 22.7 (C4) ppm.

^{19}F NMR {^{1}H} (470 MHz, $CDCl_3$): δ = –61.86 ppm.

e.r.: 65:35.

HPLC: AS-H column; eluent: *n*-hexane/ *i*-propanol 60:40; flow rate: 0.5 mL/min.

Racemic sample: ***trans*-S9**

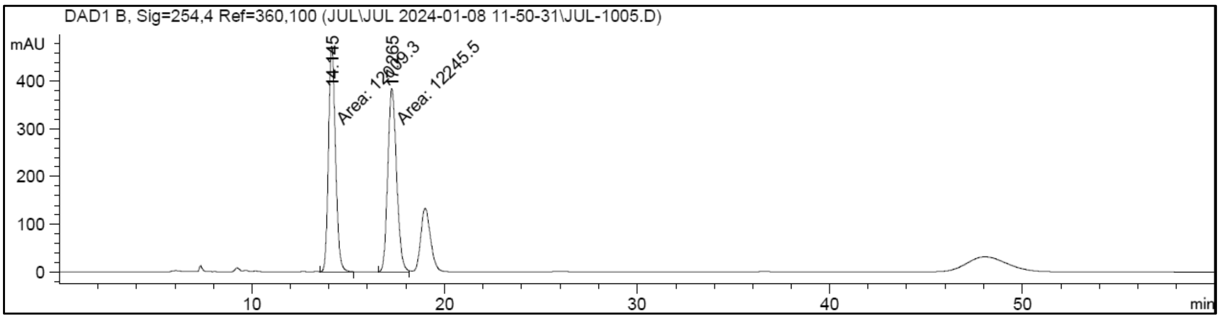

Signal 2: DAD1 B, Sig=254,4 Ref=360,100

Peak #	RetTime [min]	Type	Width [min]	Area [mAU*s]	Height [mAU]	Area %
1	14.145	MF	0.4233	1.20093e4	472.79102	49.5133
2	17.265	MF	0.5309	1.22455e4	384.41022	50.4867

Enantioselective sample: ***trans*-S9**

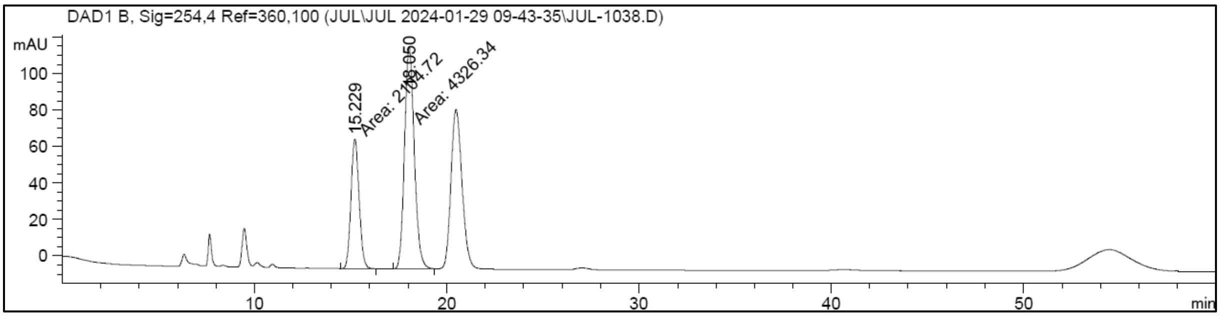

Signal 2: DAD1 B, Sig=254,4 Ref=360,100

Peak #	RetTime [min]	Type	Width [min]	Area [mAU*s]	Height [mAU]	Area %
1	15.229	MF	0.4933	2104.72388	71.11446	32.7274
2	18.050	FM	0.5905	4326.34326	122.11077	67.2726

Racemic sample: ***cis*-S9**

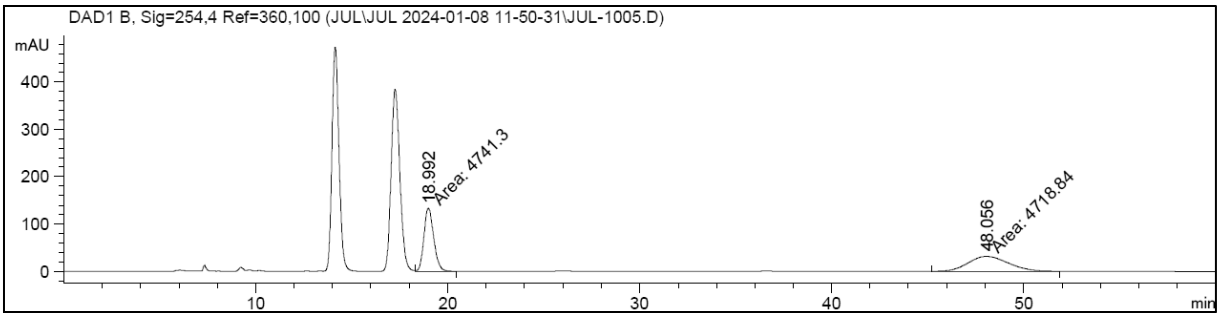

Signal 2: DAD1 B, Sig=254,4 Ref=360,100

Peak #	RetTime [min]	Type	Width [min]	Area [mAU*s]	Height [mAU]	Area %
1	18.992	MF	0.5916	4741.30371	133.57158	50.1187
2	48.056	MM	2.4614	4718.83740	31.95232	49.8813

Enantioselective sample: ***cis*-S9**

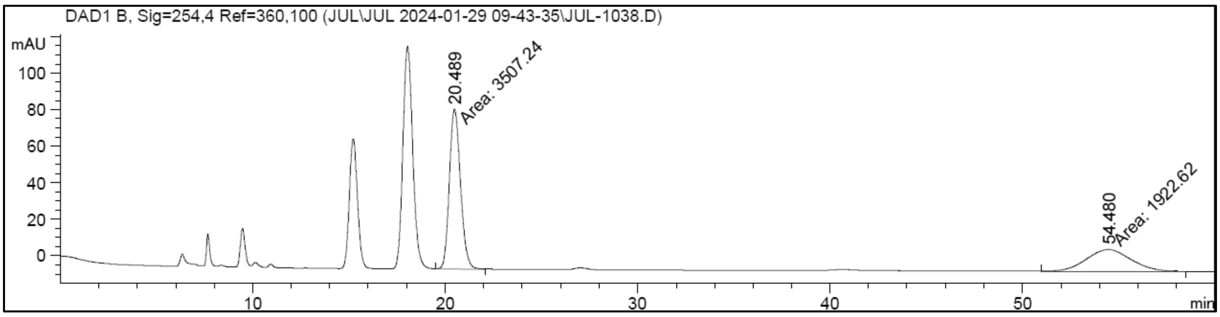

Signal 2: DAD1 B, Sig=254,4 Ref=360,100

Peak #	RetTime [min]	Type	Width [min]	Area [mAU*s]	Height [mAU]	Area %
1	20.489	FM	0.6674	3507.24414	87.58627	64.5917
2	54.480	MM	2.6838	1922.62256	11.93958	35.4083

2,4-Dimethyl-6a,7,8,9,10,10a-hexahydrophenanthridin-6(5*H*)-one (S10)

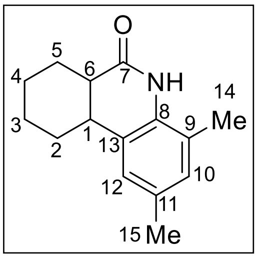

According to General Procedure **D**, **S39** (22.9 mg, 0.10 mmol, 1.00 eq.) was converted to **S10** yielding the product as a white solid (14.4 mg, 63%) after purification by column chromatography (SiO_2, 5-20% EtOAc in *n*-pentane). The product was obtained as a mixture of diastereomers (d.r. 75:25 (*trans*:*cis*)).

HR-ESI-MS: *m/z*: 252.13554 ([*M*+Na]$^+$, calcd. for $C_{15}H_{19}NONa^+$: 252.13589).

trans-diastereomer:

R*f* = 0.53 (30% EtOAc in *n*-pentane).

^{1}**H NMR** (500 MHz CDCl$_3$): δ = 7.54 (s, 1H, NH), 6.91 (s, 1H, H12), 6.87 (s, 1H, H10), 2.56 (ddd, *J* = 14.2, 10.4, 4.2 Hz, 1H, H1), 2.48 (dt, *J* = 9.6, 4.2 Hz, 1H, H2), 2.43 – 2.35 (m, 1H, H5), 2.29 (s, 3H, H15), 2.20 (s, 3H, H14), 2.03 (ddd, *J* = 14.4, 11.7, 3.8 Hz, 1H, H6), 1.98 – 1.89 (m, 2H, H3, H4), 1.44 – 1.33 (m, 3H, H2, H3, H5), 1.34 – 1.24 (m, 1H, H4) ppm.

^{13}**C NMR** (126 MHz, CDCl$_3$): δ = 173.3 (C7), 132.5 (C11), 132.2 (C9), 129.6 (C10), 128.5 (C13), 122.9 (C12), 122.4 (C8), 43.2 (C6), 38.1 (C1), 29.1 (C2), 26.3 (C5), 25.4 (C3), 25.3 (C4), 21.1 (C15), 16.9 (C14) ppm.

e.r.: 50:50.

cis-diastereomer:

R*f* = 0.61 (30% EtOAc in *n*-pentane).

^{1}H NMR (600 MHz, $CDCl_3$), selected signals: δ = 7.45 (s, 1H, NH), 6.84 (s, 1H, H10), 6.82 (s, 1H, H12), 2.86 (dt, *J* = 11.0, 5.1 Hz, 1H, H1), 2.80 – 2.77 (m, 1H, H6), 2.41 – 2.36 (m, 1H, $H_{Aliph.}$), 2.26 (s, 3H, H15), 2.19 (s, 3H, H14), 1.75 (d, *J* = 12.2 Hz, 1H, $H_{Aliph.}$), 1.66 – 1.58 (m, 2H, $H_{Aliph.}$), 1.54 – 1.46 (m, 3H, $H_{Aliph.}$) ppm.

^{13}C NMR (151 MHz, $CDCl_3$), selected signals: δ = 172.5 (C7), 132.4, 132.1, 129.6 (C10), 125.9 (C12), 122.4, 40.5 (C6), 39.6 (C1), 29.8, 29.4, 25.6, 24.7, 22.7, 20.8 (C15), 16.8 (C14) ppm. C1 and C7 were assigned via 2D NMR.

e.r.: 56:44.

HPLC: AS-H column; eluent: *n*-hexane/ *i*-propanol 90:10; flow rate: 0.5 mL/min.

Racemic sample: ***trans*-S10**

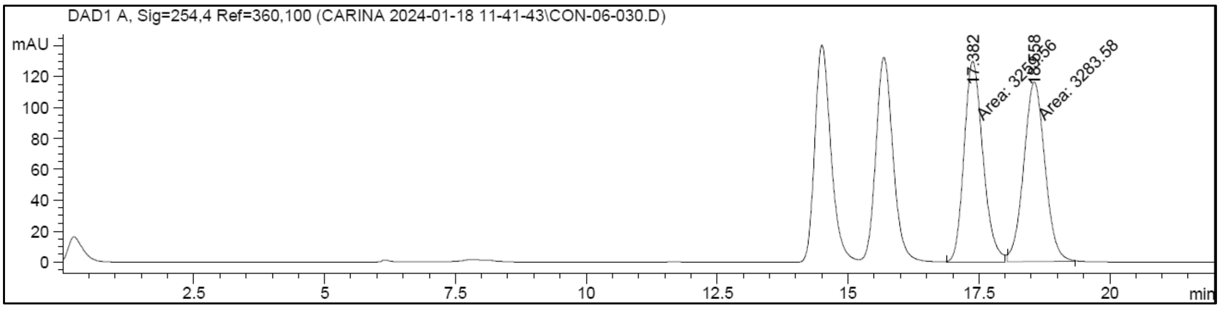

Signal 1: DAD1 A, Sig=254,4 Ref=360,100

Peak #	RetTime [min]	Type	Width [min]	Area [mAU*s]	Height [mAU]	Area %
1	17.382	MF	0.4188	3259.56201	129.72260	49.8164
2	18.558	MF	0.4708	3283.58374	116.23634	50.1836

Enantioselective sample: ***trans*-S10**

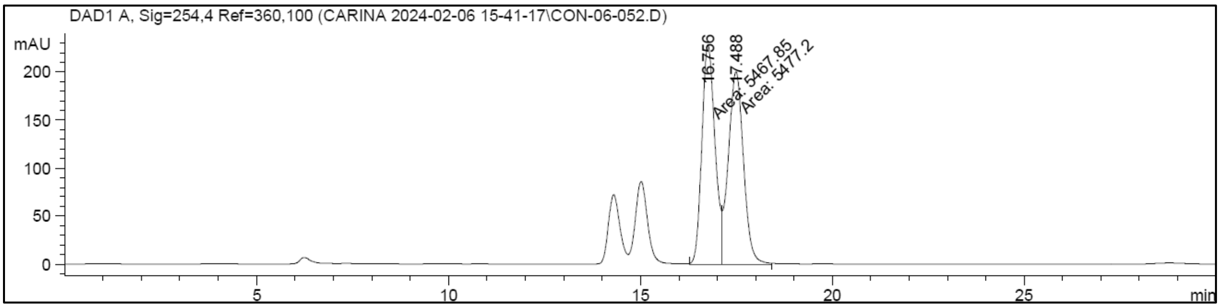

Signal 1: DAD1 A, Sig=254,4 Ref=360,100

Peak #	RetTime [min]	Type	Width [min]	Area [mAU*s]	Height [mAU]	Area %
1	16.756	MF	0.3991	5467.85352	228.35304	49.9573
2	17.488	FM	0.4556	5477.20361	200.37279	50.0427

*Racemic sample: **cis*-S10**

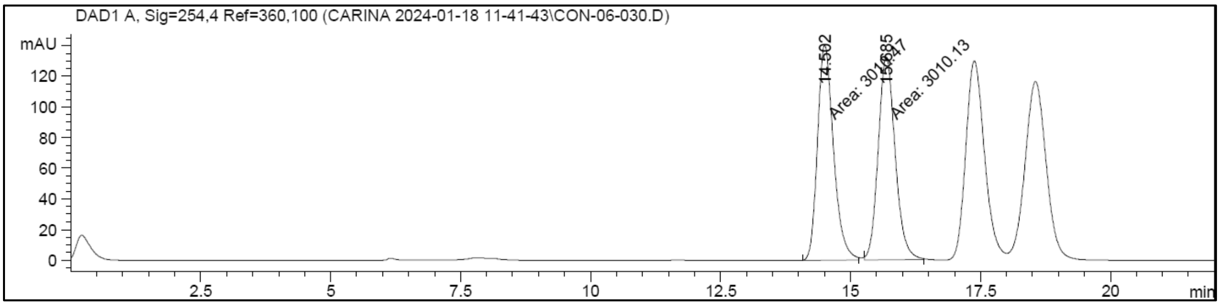

Signal 1: DAD1 A, Sig=254,4 Ref=360,100

Peak #	RetTime [min]	Type	Width [min]	Area [mAU*s]	Height [mAU]	Area %
1	14.502	MF	0.3568	3010.47339	140.62845	50.0028
2	15.685	FM	0.3785	3010.13354	132.53648	49.9972

*Enantioselective sample: **cis*-S10**

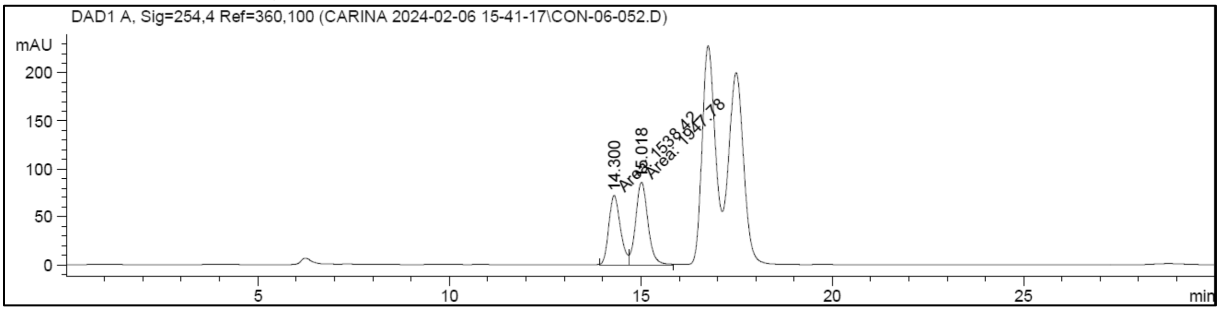

Signal 1: DAD1 A, Sig=254,4 Ref=360,100

Peak #	RetTime [min]	Type	Width [min]	Area [mAU*s]	Height [mAU]	Area %
1	14.300	MF	0.3515	1538.41553	72.94157	44.1288
2	15.018	FM	0.3751	1947.77881	86.54618	55.8712

3-Phenyl-3,4-dihydroquinolin-2(1*H*)-one (S11)

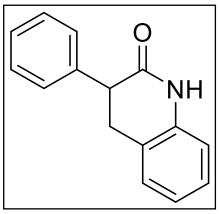

According to General Procedure **D**, **S40** (22.3 mg, 0.10 mmol, 1.00 eq.) was converted to **S11** yielding the product as a white solid (7.4 mg, 33%) after purification by column chromatography (SiO_2, 30% EtOAc in *n*-pentane).

R*f* = 0.32 (30% EtOAc in *n*-pentane, both diastereomers).

^{1}H NMR (400 MHz, $CDCl_3$): δ = 8.30 (s, 1H), 7.35 – 7.24 (m, 5H), 7.22 – 7.15 (m, 2H), 7.00 (t, *J* = 7.4 Hz, 1H), 6.79 (d, *J* = 7.8 Hz, 1H), 3.88 (dd, *J* = 8.8, 7.2 Hz, 1H), 3.32 – 3.20 (m, 2H) ppm.

HR-ESI-MS: *m/z*: 246.08882 ([*M*+Na]$^+$, calcd. for $C_{15}H_{13}NONa^+$: 246.08894).

Analytical data are in agreement with literature.[26]

e.r.: 73:27.

HPLC: AS-H column; eluent: *n*-hexane/ *i*-propanol 40:60; flow rate: 1.0 mL/min.

Racemic sample: **S11**

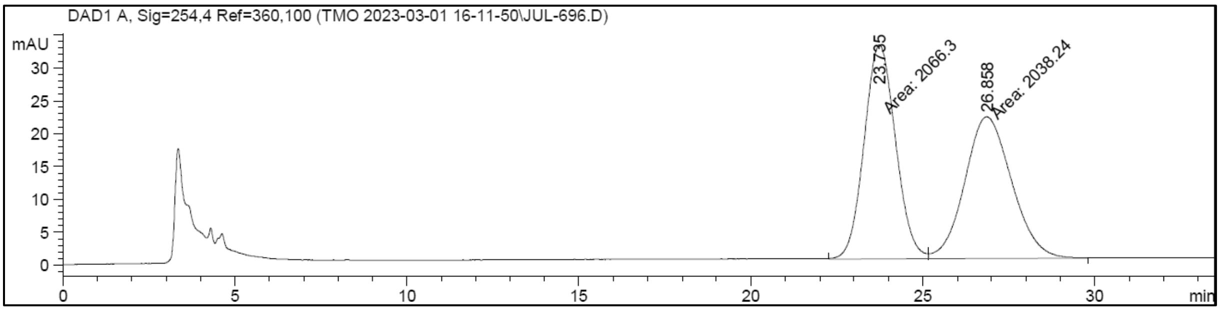

Signal 1: DAD1 A, Sig=254,4 Ref=360,100

Peak #	RetTime [min]	Type	Width [min]	Area [mAU*s]	Height [mAU]	Area %
1	23.735	MF	1.0593	2066.29663	32.50910	50.3418
2	26.858	FM	1.5791	2038.23779	21.51287	49.6582

Enantioselective sample: **S11**

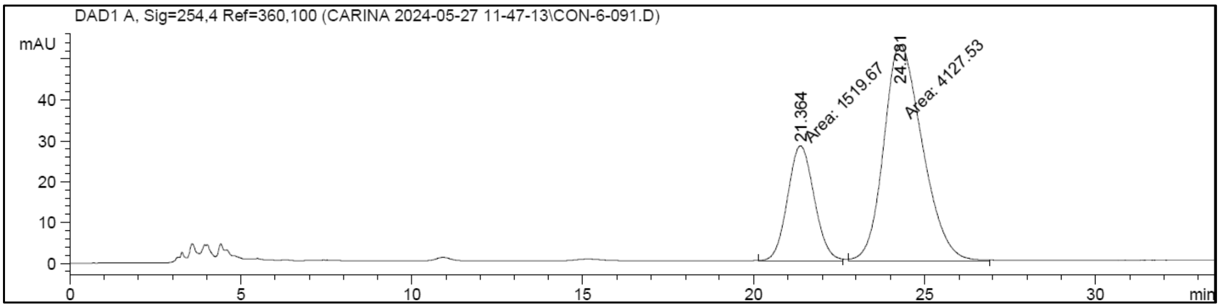

Signal 1: DAD1 A, Sig=254,4 Ref=360,100

Peak #	RetTime [min]	Type	Width [min]	Area [mAU*s]	Height [mAU]	Area %
1	21.364	MF	0.9021	1519.66663	28.07565	26.9101
2	24.281	MF	1.3040	4127.53223	52.75286	73.0899

3-Methyl-3,4-dihydroquinolin-2(1*H*)-one (S12)

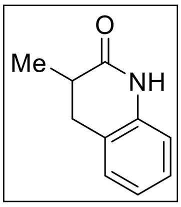

According to General Procedure **D**, **S43** (16.1 mg, 0.10 mmol, 1.00 eq.) was converted to **S8** yielding the product as a white solid (4.8 mg, 30%) after purification by column chromatography (SiO_2, 25% EtOAc in *n*-pentane).

R_f = 0.23 (20% EtOAc in *n*-pentane).

^{1}H NMR (400 MHz, $CDCl_3$): δ = 7.80 (s, 1H), 7.22 – 7.13 (m, 2H), 6.98 (td, *J* = 7.5, 1.2 Hz, 1H), 6.74 (d, *J* = 8.2 Hz, 1H), 3.00 (dd, *J* = 15.1, 5.5 Hz, 1H), 2.82 – 2.60 (m, 2H), 1.29 (d, *J* = 6.8 Hz, 3H). ppm.

HR-ESI-MS: *m/z*: 184.07329 ([*M*+Na]$^+$, calcd. for $C_{10}H_{11}NONa^+$: 184.07329).

Analytical data are in agreement with literature.[27]

e.r.: 51:49.

HPLC: AS-H column; eluent: *n*-hexane/ *i*-propanol 40:60; flow rate: 1.0 mL/min.

Racemic sample: **S8**

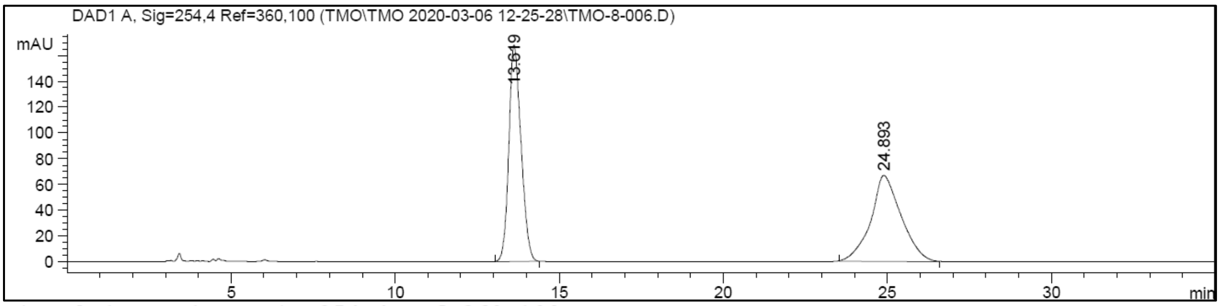

Signal 1: DAD1 A, Sig=254,4 Ref=360,100

Peak #	RetTime [min]	Type	Width [min]	Area [mAU*s]	Height [mAU]	Area %
1	13.619	BB	0.3930	4342.22949	168.29176	50.1730
2	24.893	BB	0.9116	4312.28516	66.75698	49.8270

Enantioselective sample: **S8**

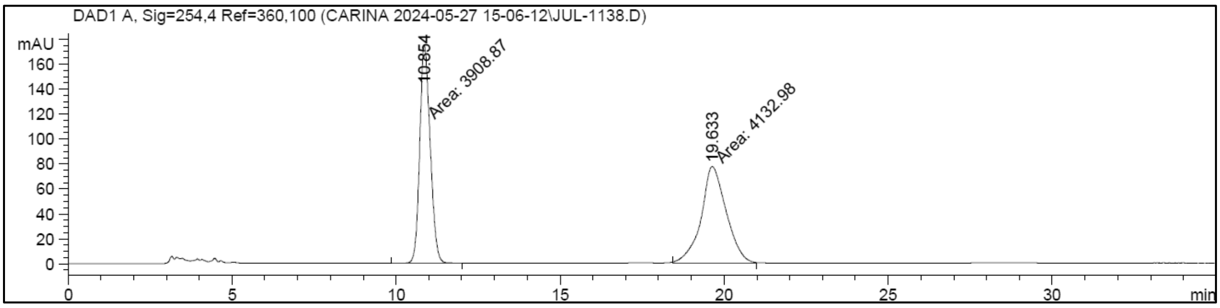

Signal 1: DAD1 A, Sig=254,4 Ref=360,100

Peak #	RetTime [min]	Type	Width [min]	Area [mAU*s]	Height [mAU]	Area %
1	10.854	MM	0.3717	3908.86646	175.26147	48.6066
2	19.633	FM	0.8904	4132.97900	77.36610	51.3934

Optimization of 6π-Photocyclization Reaction Conditions

Optimization reactions were performed according to general procedures **D** or **E** (according to the reaction temperature). The products were purified by column chromatography (SiO_2, *n*-pentane/EtOAc: 80/20). Diastereomeric ratios were determined by ^{1}H NMR spectroscopy and enantiomeric ratios were determined by HPLC (column: AS-H; eluent: *n*-hexane/*i*-propanol: 15/85; flow rate: 1.0 mL/min, 35 °C).

Table 1: Optimization of the 6π-cyclization: Catalyst screening.

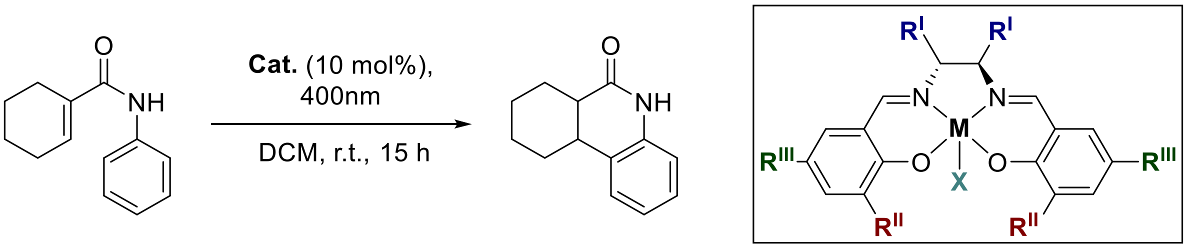

entry	M	X	R^{I}	R^{II}	R^{III}	yield [%]	*d.r.* (*trans*:*cis*)	*e.r.* *trans*	*e.r.* *cis*
**1**	Al	Cl	$(CH_2)_2$	^{t}Bu	^{t}Bu	44	71:29	74:26	68:32
**2**	Al	Cl	Ph	^{t}Bu	^{t}Bu	14	71:29	65:35	54:46
**3**	Al	O-Al(salen)	$(CH_2)_2$	^{t}Bu	^{t}Bu	42	67:33	23:77	27:73
**4**	Al	Me	$(CH_2)_2$	^{t}Bu	^{t}Bu	36	71:29	73:27	75:25
**5**	Al	F	$(CH_2)_2$	^{t}Bu	^{t}Bu	20	66:34	79:21	76:24
**6**	Al	Cl	$(CH_2)_2$	H	^{t}Bu	95	55:45	63:37	55:45
**7**	Al	Cl	$(CH_2)_2$	^{t}Bu	H	64	64:36	67:33	53:47
**8**	Al	Cl	$(CH_2)_2$	Ph	H	83	63:37	67:33	57:43
**9**	Al	Cl	$(CH_2)_2$	Ad	Me	15	66:34	84:16	80:20
**10**	Co	-	$(CH_2)_2$	^{t}Bu	^{t}Bu	0	-	-	-
**11**	-	-	$(CH_2)_2$	^{t}Bu	^{t}Bu	0	-	-	-

As the best compromise between high yield and enantioselectivities, the optimization was continued using the commercially available (*R,R*)-*N,N'*-Bis(3,5-di-*tert*-butylsalicylidene)-1,2-cyclohexanediaminoaluminum chloride, referred to as **Al-1**, as catalyst.

Table 2: Optimization of the 6π-cyclization: Solvent and drying agent screening.

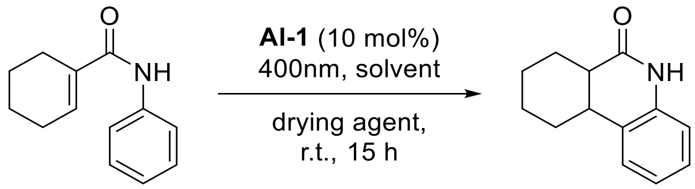

entry	solvent	drying agent[a]	yield [%]	*d.r.* (*trans*:*cis*)	*e.r.* *trans*	*e.r.* *cis*
**1**	DCM	-	44	71:29	74:26	68:32
**2**	DCE	-	43	71:29	72:28	71:29
**3**	MeCN	-	33	56:44	64:36	62:38
**4**	PhMe	-	33	67:33	64:36	56:44
**5**	$PhCF_3$	-	46	77:23	71:29	63:27
**6**	Acetone	-	< 5	n.d.	n.d.	n.d.
**7**	1,4-Dioxane	-	5	54:46	57:43	54:46
**8**	EtOAc	-	8	59:41	n.d.	n.d.
**9**	DCM	MS (3 Å)	45	70:30	76:24	69:31
**10**	DCM	$MgSO_4$	35	66:34	73:27	69:31
**11**	DCM	Na_2SO_4	28	72:28	77:23	74:26
**12**	DCM	Drierite®	41	69:31	73:27	70:30

a) 15 mg of drying agent were added.

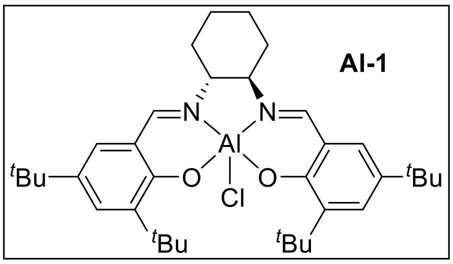

The best enantioselectivities were obtained performing the cyclization reactions in dichloromethane, which was used as solvent for the remainder of the study. Additionally, slightly improved enantioselectivities were observed with the addition of molecular sieves or sodium sulfate as drying agent. Because the improvement was only marginal, and in the case of sodium sulfate a reduced yield was obtained, an additive screen was performed without the addition of a drying agent.

Table 3: Optimization of the 6π-cyclization: Additive screening.

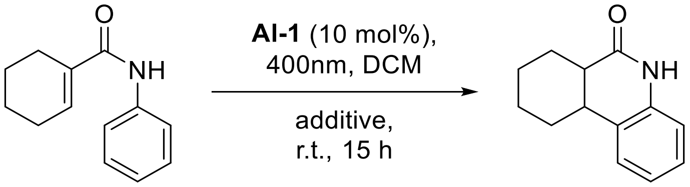

entry	additive[a]	yield [%]	*d.r.* (*trans*:*cis*)	*e.r.* *trans*	*e.r.* *cis*
**1**	-	44	71:29	74:26	68:32
**2**	nBu$_4$NCl	67	67:33	78:22	80:20
**3**	nBu$_4$NBr	62	58:42	75:25	76:24
**4**	nBu$_4$NI	13	62:38	80:20	80:20
**5**	nBu$_4$NBF$_4$	58	61:39	70:30	67:33
**6**	nBu$_4$NPF$_6$	62	71:29	69:31	68:32
**7**	nBu$_4$OAc	< 5	n.d.	n.d.	n.d.
**8**	Et$_4$NCl	77	60:40	77:23	82:18
**9**	Me$_4$NCl	40	68:32	74:26	74:26
**10**	Me$_3$BnNCl	66	70:30	79:21	77:23
**11**	Selectfluor®	57	64:36	69:31	69:31
**12**	DIPEA	< 5	n.d.	n.d.	n.d.
**13**	Et$_3$N*HCl	48	53:47	75:25	77:23
**14**	Biphenyl	50	67:33	68:32	64:34
**15**	1-Cyanonaphthalene	25	62:38	53:47	n.d.
**16**	K$_2$CO$_3$	30	67:33	80:20	75:25
**17**	KI	60	49:51	70:30	62:38
**18**	HFIP	95	51:49	67:33	67:33

a) 1.5 eq. of additive were added.

The optimization was continued using Et$_4$NCl (1.5 eq.) as additive as the best compromise between high yield and enantioselectivities.

Table 4: Optimization of the 6π-cyclization at low temperature.

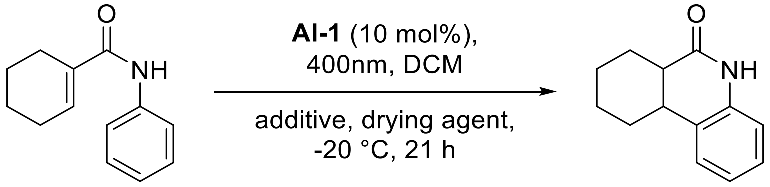

entry	additive[a]	drying agent[b]	yield [%]	*d.r.* (*trans*:*cis*)	*e.r.* *trans*	*e.r.* *cis*
**1**	Et_4NCl	-	41	59:41	79:21	88:12
**2**	Et_4NCl	MS (3 Å)	32	57:43	80:20	90:10
**3**	$n\text{Bu}_4NCl$	-	50	64:36	82:18	85:15
**4**	$n\text{Bu}_4NCl$	MS (3 Å)	55	62:38	82:18	88:12
**5**	$n\text{Bu}_4NCl$	Na_2SO_4	54	65:35	81:19	87:13
**6** [c]	$n\text{Bu}_4NCl$	-	62	69:31	82:18	90:10
**7** [c]	$n\text{Bu}_4NCl$	MS (3 Å)	57	66:34	84:16	90:10
**8**[c]	$n\text{Bu}_4NCl$	Na_2SO_4	65	67:33	82:18	89:11

a) 1.5 eq. of additive were added; b) 15 mg of drying agent were added; c) 20 mol% of catalyst were used.

Due to the practical reaction set-up (irradiation via a glass rod that is inserted through a pre-slit septum) the two best performing drying agents were reconsidered for reactions at low temperatures.

In reactions with Et_4NCl as additive at –20 °C, precipitation of the additive and decomposition of the catalyst were observed to hamper the reproducibility of the results presented in SI Table 4 entries 1 and 2. Therefore, the additive was changed to $n\text{Bu}_4NCl$, which is less likely to precipitate at low reaction temperatures. Similar to the results presented in SI Table 2, the addition of molecular sieves led to a slightly improved enantioselectivity. Therefore, the optimization was continued with the conditions presented in in SI Table 4 entry 7. Under these conditions perfect reproducibility was achieved. As the addition of molecular sieves causes cloudiness of the reaction mixture, the effect of varying the amount of molecular sieves was studied at a later stage of the optimization (SI Table 6). This might also explain the decreased yield observed upon addition of molecular sieves when 20 mol% of catalyst were used (SI Table 4 entries 6 and 7).

Table 5: Optimization of the 6π-cyclization: Influence of concentration.

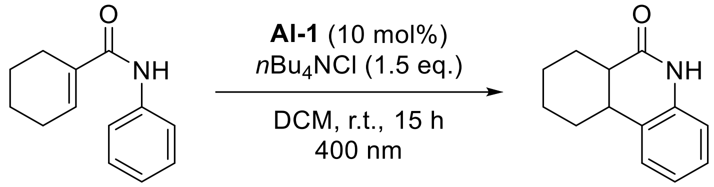

entry	concentration [M]	yield [%]	*d.r.* (*trans*:*cis*)	*e.r.* *trans*	*e.r.* *cis*
**1**	0.10	68	65:35	77:23	80:20
**2**	0.066	67	67:33	78:22	80:20
**3**	0.05	53	66:34	76:24	79:21

The influence of the concentration on the enantioselectivities was negligible and a slight decrease of yield was observed at higher concentrations. Thus, a concentration of 0.066 M was retained.

Table 6: Optimization of the 6π-cyclization: Molecular sieves loading.

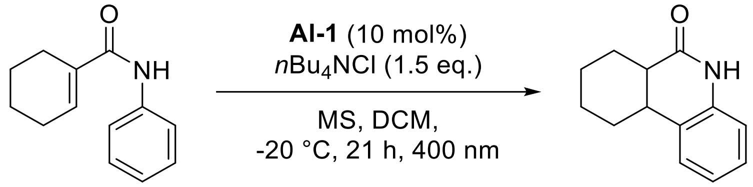

Entry	amount MS (3 Å) [mg]	yield [%]	*d.r.* (*trans*:*cis*)	*e.r.* *trans*	*e.r.* *cis*
**1**	6	67	66:34	84:16	90:10
**2**	9	58	66:34	84:16	91:9
**3**	15	57	66:34	84:16	90:10
**4**	20	54	63:37	84:16	86:14

Decreasing the amount of molecular sieves had a beneficial effect on the cyclization yield and the conditions presented in SI Table 6 entry 1 were chosen as the final conditions for the photocatalytic 6π-cyclization of acrylanilides.

Mechanistic Investigations

Temperature effect:

Reactions were performed according to general procedures **D** or **E** (according to the reaction temperature). The products were purified by column chromatography (SiO_2, *n*-pentane/EtOAc: 80/20). Diastereomeric ratios were determined by ^1H NMR spectroscopy and enantiomeric ratios were determined by HPLC (column: AS-H; eluent: *n*-hexane/*i*-propanol: 15/85; flow rate: 1.0 mL/min, 35 °C).

Table 7: Effect of temperature on the 6π-cyclization reaction under optimized conditions.

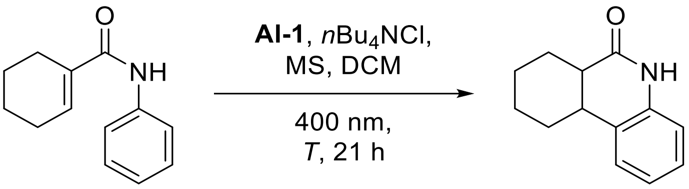

entry	*T* [°C]	yield [%]	*d.r.* (*trans*:*cis*)	*trans*-diastereomer		*cis*-diastereomer	
				e.r.	ln(*e.r.*)	*e.r.*	ln(*e.r.*)
**1**	-30	41	64:36	86:14	1.815	92:8	2.442
**2**	-20	67	66:34	84:16	1.658	90:10	2.197
**3**	-10	67	65:35	82:18	1.516	89:11	2.091
**4**	0	79	67:33	82:18	1.516	88:12	1.992
**5**[a]	25	69	66:34	77:23	1.208	79:21	1.325

a) reaction time: 16 h.

In agreement with an expected temperature dependence of the enantiomeric ratios, correlation of ln(*e.r.*) with the reciprocal of *T* [K] yields the following linear correlations:

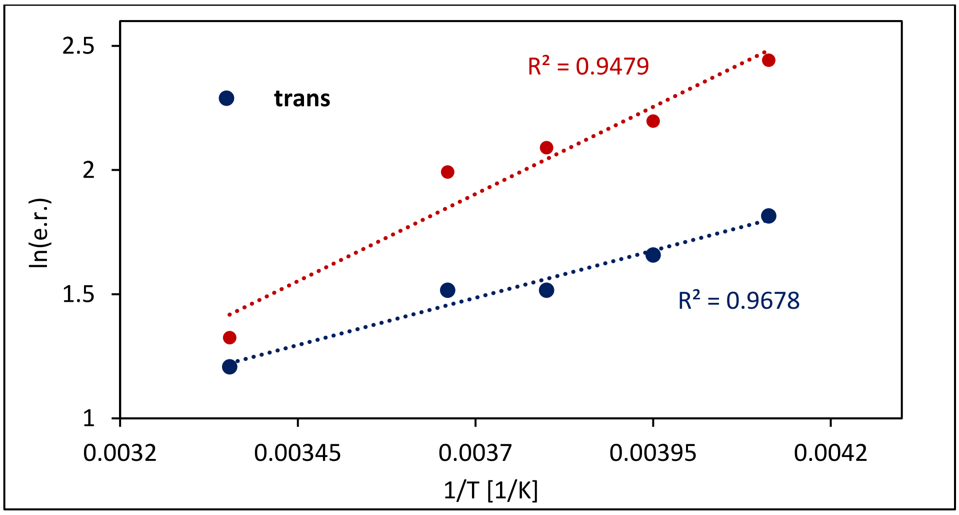

Figure 6: Temperature correlation for the 6π-cyclization reaction under optimized conditions.

UV/vis spectroscopy:

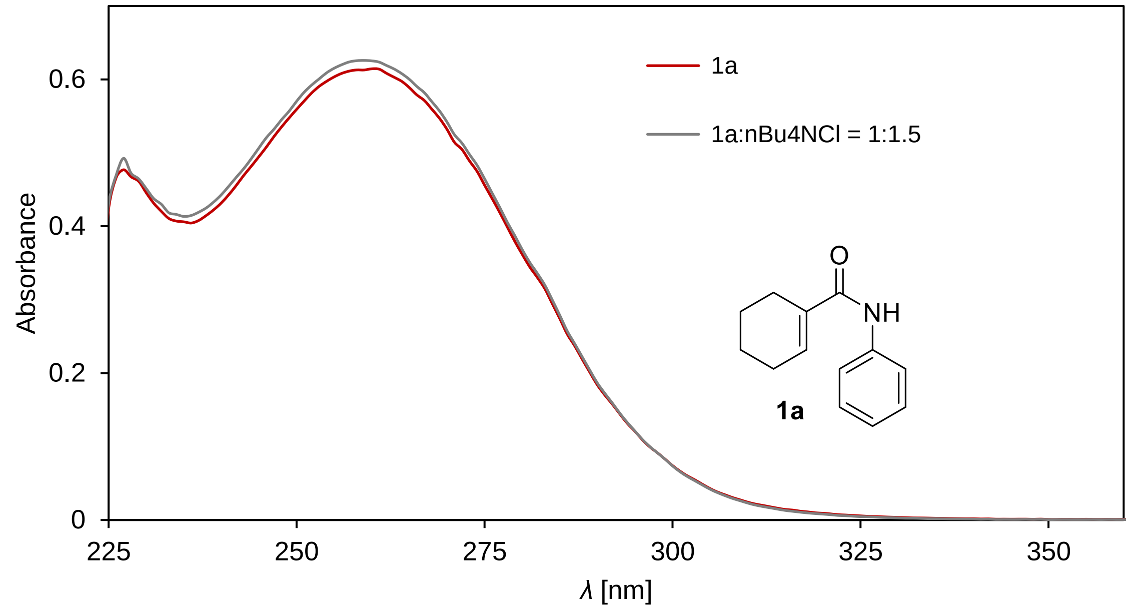

Figure 7: Absorption spectra of **1a** in the presence and absence of $n\text{Bu}_4\text{NCl}$ (1.5 eq.) (c = 0.05 mM in DCM).

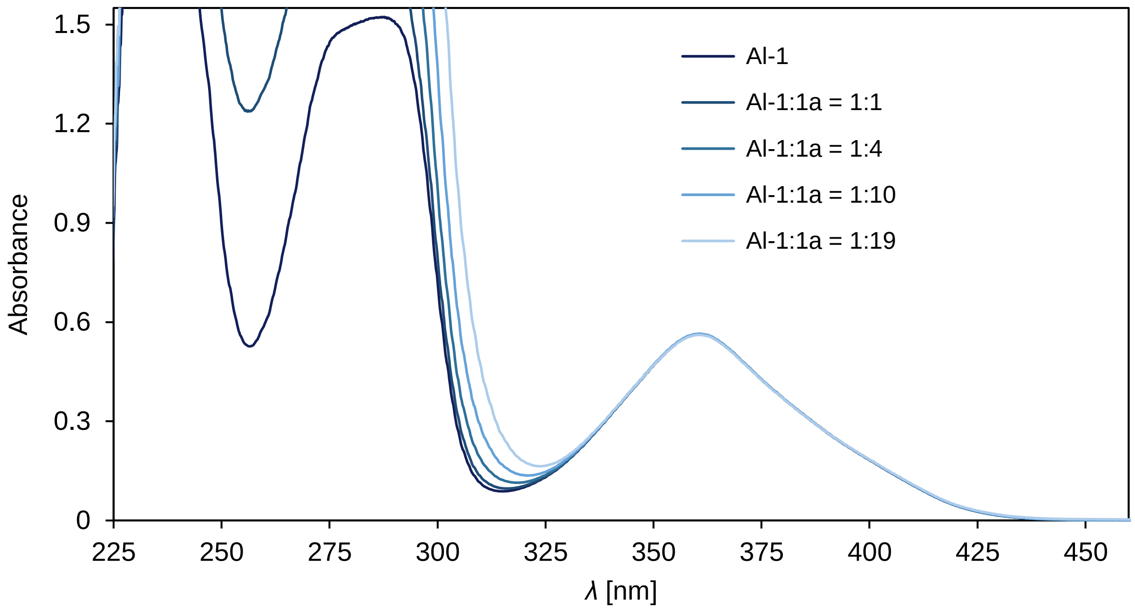

***Figure 8*:** Absorption spectra of **Al-1** in the presence of varying amounts of **1a** ($c_{\text{Al-1}}$ = 0.05 mM in DCM).

The absence of a shift (or increase/decrease of intensity) of the absorption band with $\lambda_{\text{max.}} \approx 360$ nm indicated that no ground state coordination between the catalyst **Al-1** and the substrate **1a** takes place. The increasing intensity below 325 nm is due to the absorption by the substrate **1a**.

Cyclic voltammetry:

Cyclic voltammograms of **1a**, **1b**, **1l**, **1o**, and **1s** were recorded at a 1 V/s scan rate with ferrocene as internal reference (for instrumental details see General Information). Measurements were performed in dry and degassed acetonitrile under argon atmosphere at c = 5.0 mM for the substrates and c = 0.1 M of Bu_4NPF_6 as conducting salt. Voltammograms referenced against SCE were obtained by correction against literature values (+0.382 V vs. SCE).[28]

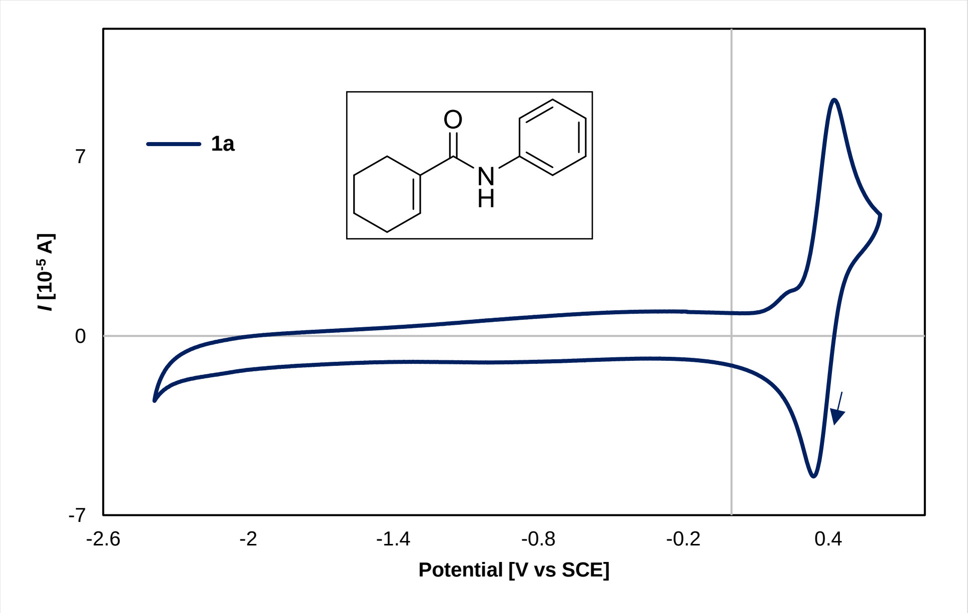

Figure 9: Cyclic voltammogram of **1a**.

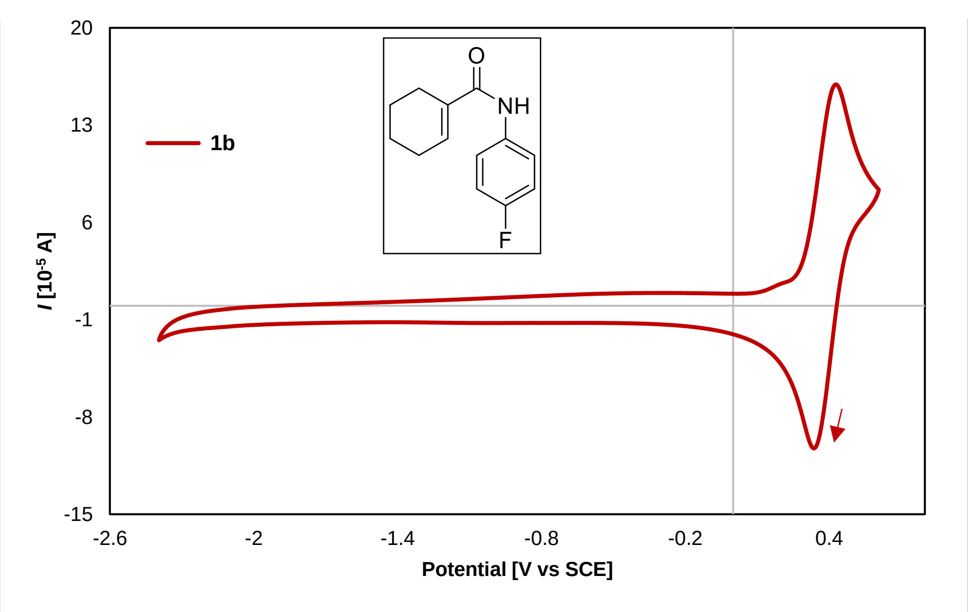

Figure 10: Cyclic voltammogram of **1b**.

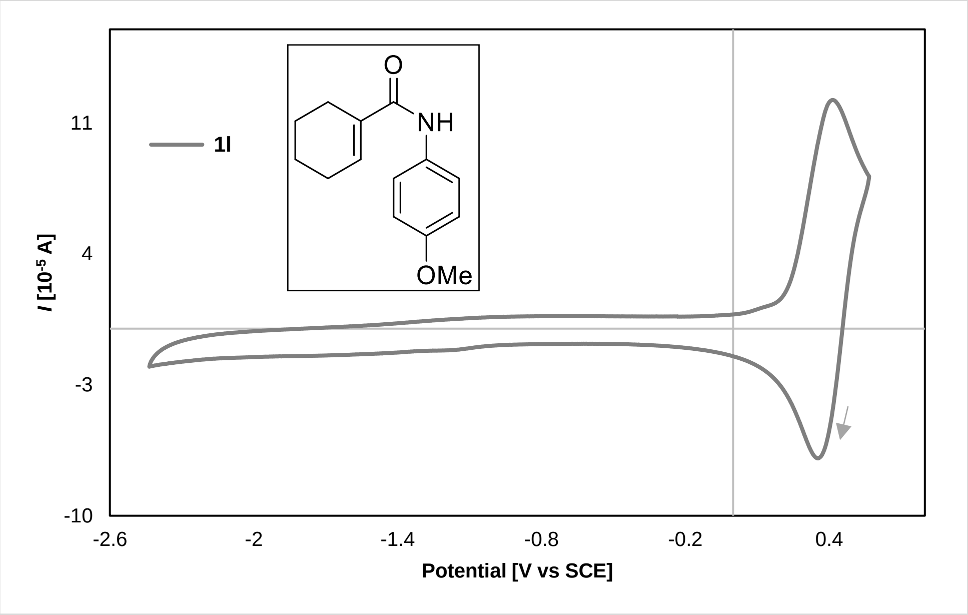

Figure 11: Cyclic voltammogram of **1l**.

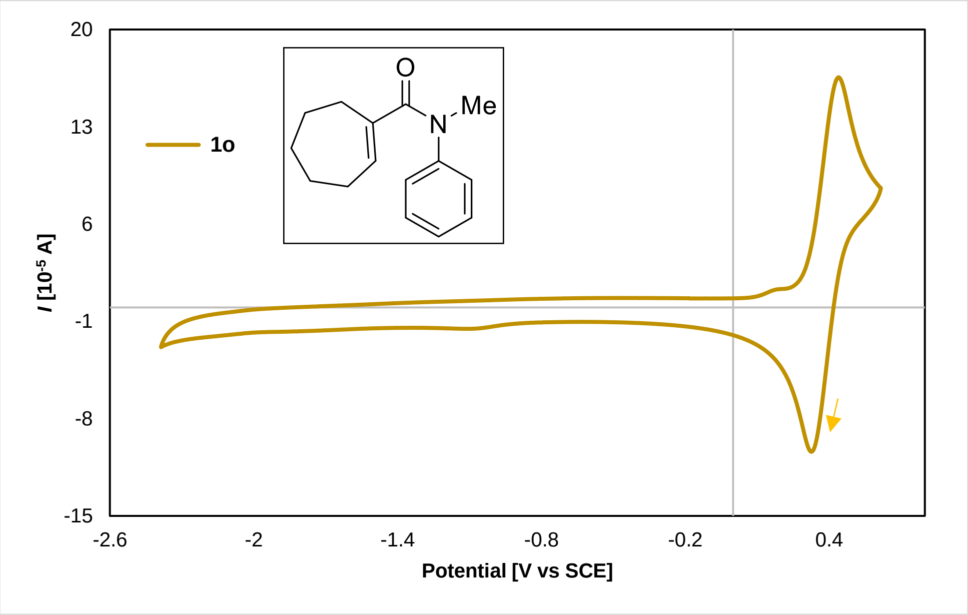

Figure 12: Cyclic voltammogram of **1o**.

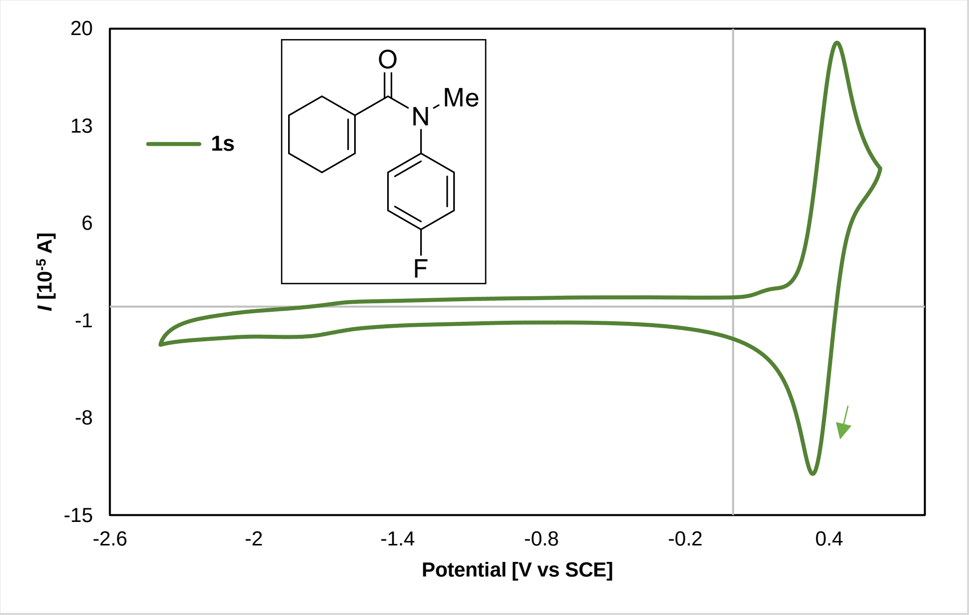

Figure 13: Cyclic voltammogram of **1s**.

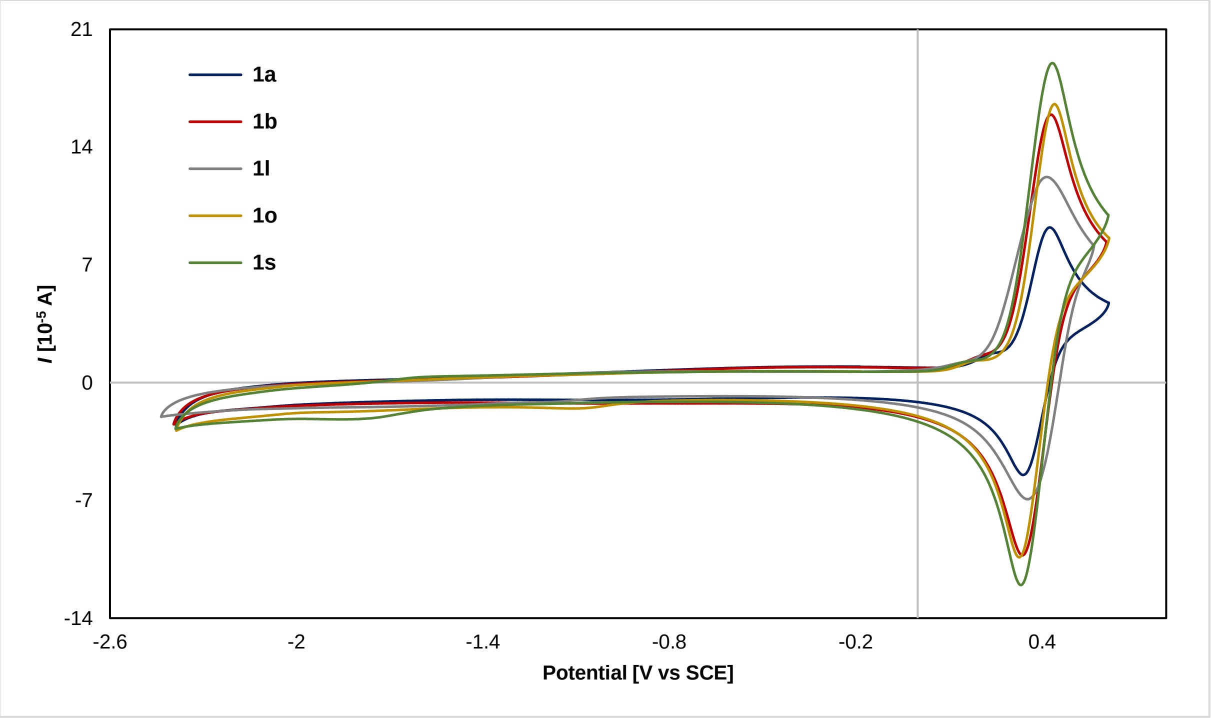

Figure 14: Cyclic voltammogram of **1a**, **1b**, **1l**, **1o** and **1s** in one graph.

No reduction potential could be determined by the cyclic voltammetry measurement leading to the assumption that all compounds have a reduction potential below –2.3 V vs. SCE.

Radical trapping experiment with TEMPO and styrene:

The effect of the addition of TEMPO or styrene as radical scavengers on the reaction outcome was investigated. Substrate **1a** was probed using 2 and 5 equivalents of radical scavenger under the standard conditions (SI Scheme 1).

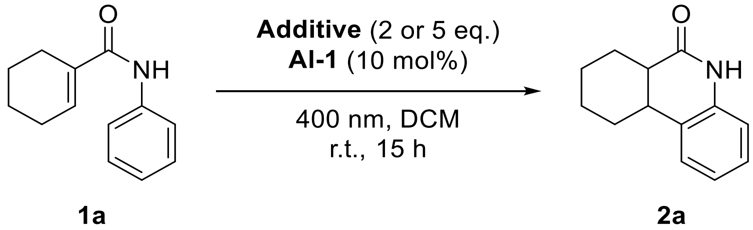

Scheme 1: Trapping Experiment with TEMPO or styrene as radical scavenger.

Following General Procedure **E**, a pressure tube was charged with a stir bar, **1a** (20.1 mg,0.10 mmol, 1.00 eq.) and aluminum catalyst **Al-1** (12.1 mg, 0.02 mmol, 20 mol%). TEMPO (78 mg, 0.5 mmol, 5.0 equiv.) [or 31 mg, 0.2 mmol, 2.0 equiv.]) and DCM (1.5 mL), or DCM (1.5 mL) and styrene (22.9 µL, 0.2 mmol, 2.00 eq.) [or 57.2 µL, 0.5 mmol, 5.00 eq.] were added to reaction mixture. The pressure tube was sealed, removed from the glove box and stirred under light irradiation at 400 nm for 21 h at room temperature. Afterwards, the solvent was removed under reduced pressure, 1,3,5-trimethoxy-benzene (5.6 mg, 0.033 mmol, 0.33 equiv.) was added as internal standard and the crude mixture was analyzed by ^{1}H NMR and HRMS.

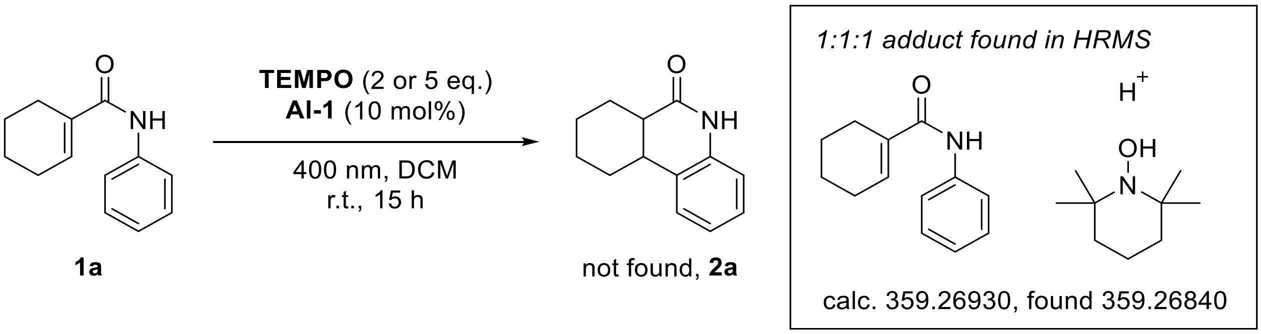

Scheme 2**:** Results of the trapping experiments with TEMPO.

HR-ESI-MS: *m/z*: 359.26840 ([*M*+H]$^+$, calcd. for $C_{22}H_{34}N_2O_2H^+$: 359.26930).

Adding TEMPO (in either 2.00 eq. or 5.00 eq.) to the reaction mixture did not lead to any product formation (investigated via the NMR) and the adduct 1:1:1 of the substrate, TEMPO and H$^+$ was observed in the high-resolution mass spectrometry (HRMS). This suggests the participation of radicals in the reaction.

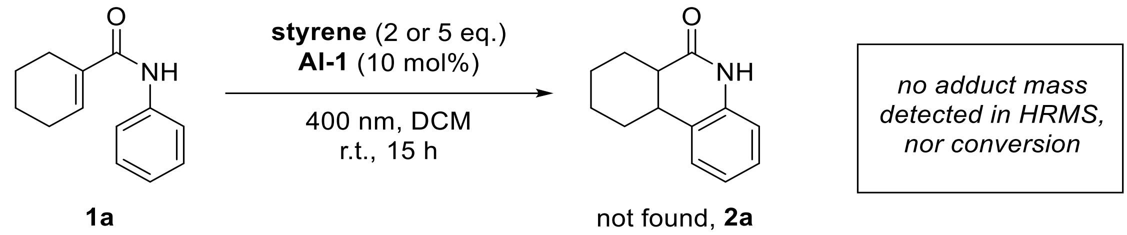

***Scheme 3*:** Results of the trapping experiments with stryene.

The addition of styrene supresses the reaction completely and the starting material was recovered almost quantitively (>95%). However, no adduct formation was observed in the NMR spectra or HRMS.

Deuterium experiments:

Experiments with deuterated substrates and/or reagents were performed on a 0.1 mmol scale according to general procedures **D** (cyclization of **1a** and **1v**) or **F** (cyclization of **1q** and **1w**). Products were purified by column chromatography (SiO_2, *n*-pentane/EtOAc 8:2 (**2a**, **2v**) or *n*-pentane/EtOAc 9:1 (**2q**, **2w**)). The degree of deuterium incorporation was determined by ^1H NMR spectroscopy.

Table 8: Deuterium incorporation at the 6a-position in **2a** and **2v**.

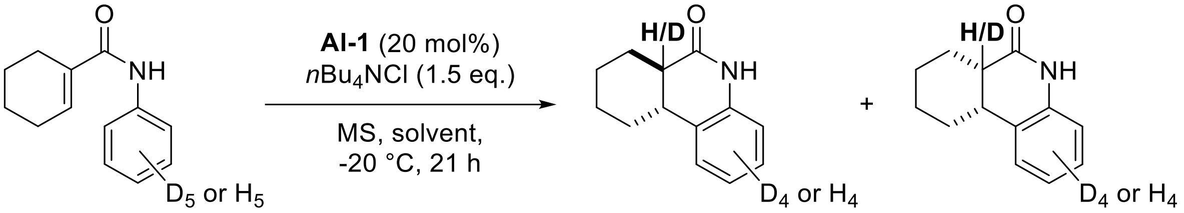

H_5: **1a** H_4: *trans*-**2a** H_4: *cis*-**2a**
D_5: **1v** D_4: *trans*-**2v** D_4: *cis*-**2v**

entry	substrate	solvent	yield [%]	d.r. (*trans*:*cis*)	D-incorporation *trans* [%]	D-incorporation *cis* [%]
**1**	**1v**	CH_2Cl_2	69	61:39	84	5
**2**	**1v**	CD_2Cl_2	61	61:39	88	7
**3**	**1a**	CD_2Cl_2	61	65:35	0	0

Table 9: Deuterium incorporation at the 6a-position in **2q** and **2w**.

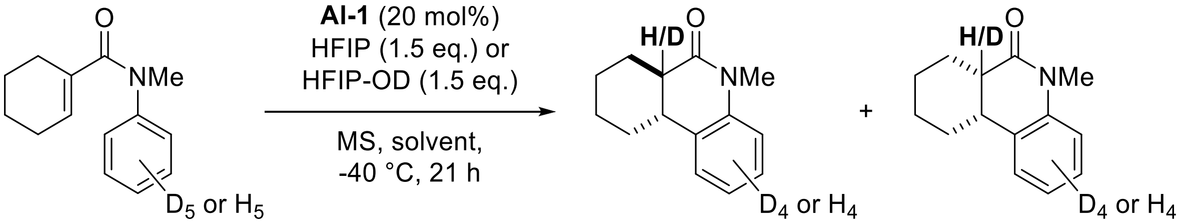

H_5: **1q** H_4: *trans*-**2q** H_4: *cis*-**2q**
D_5: **1w** D_4: *trans*-**2w** D_4: *cis*-**2w**

entry	substrate	solvent	additive	yield [%]	d.r. (*trans*:*cis*)	D-incorporation *trans* [%]	D-incorporation *cis* [%]
**1**	**1w**	CH_2Cl_2	HFIP	86	85:15	55	15
**2**	**1w**	CD_2Cl_2	HFIP	93	82:18	52	15
**3**	**1q**	CD_2Cl_2	HFIP	54	88:12	0	0
**4**	**1w**	CH_2Cl_2	HFIP-OD	94	86:14	71	53
**5**	**1w**	CD_2Cl_2	HFIP-OD	89	86:14	76	53
**6**	**1q**	CH_2Cl_2	HFIP-OD	75	90:10	11	29
**7**	**1q**	CD_2Cl_2	HFIP-OD	77	88:12	11	29

^1H NMR spectra of the products of the deuterium experiment:

Table 8, entry 1: D-incorporation of *trans*-**2v**

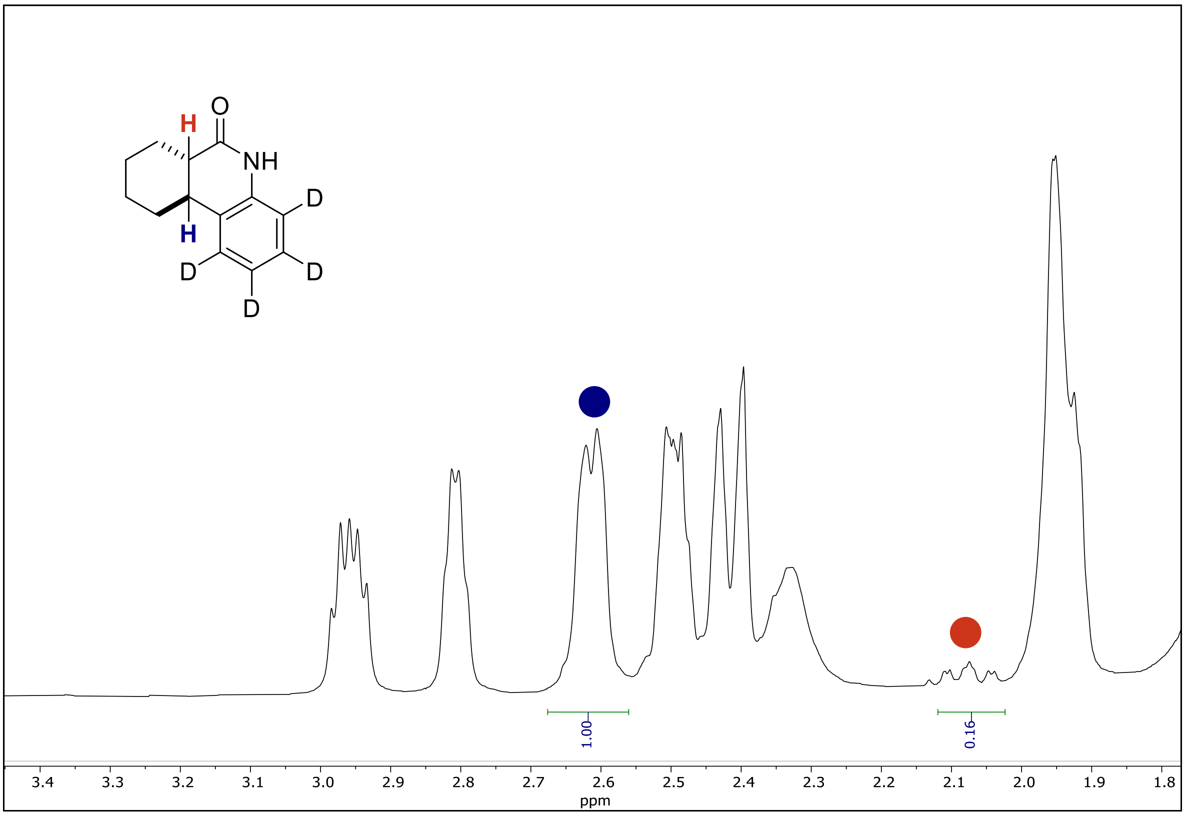

Table 8, entry 1: D-incorporation of *cis*-**2v**

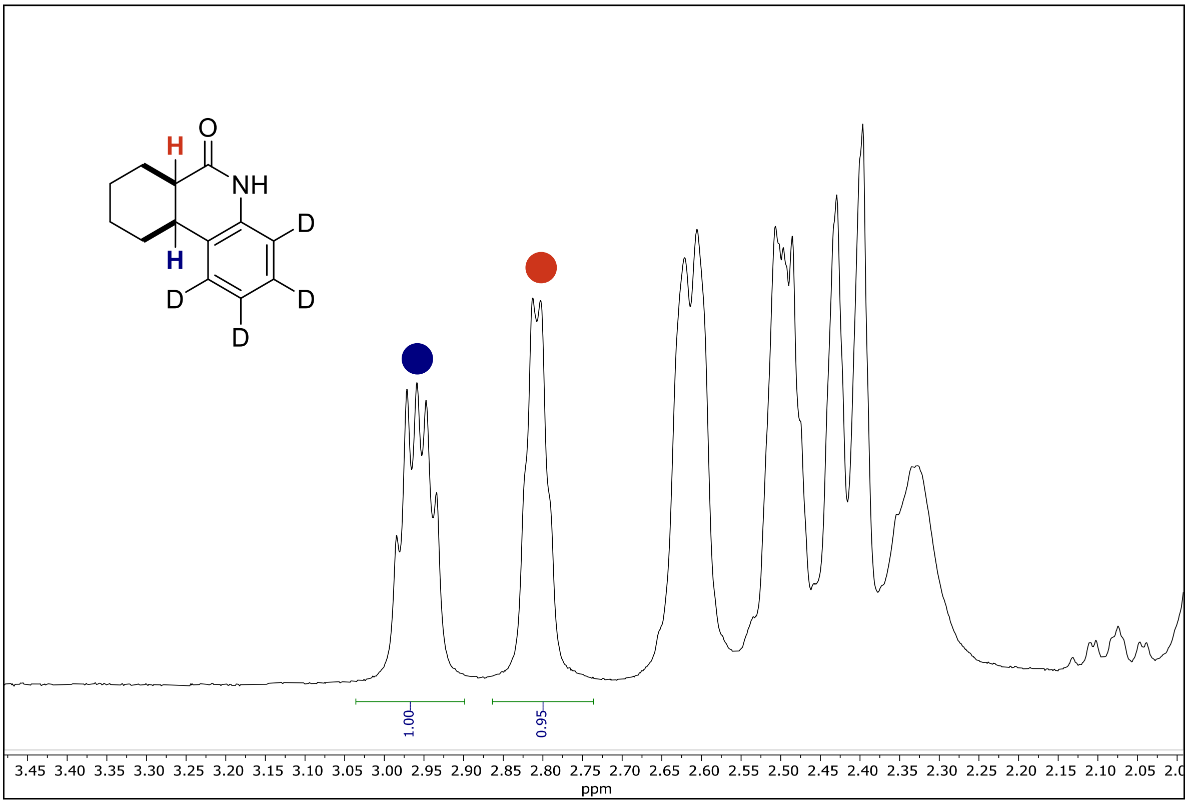

Table 8, entry 2: D-incorporation of *trans*-**2v**

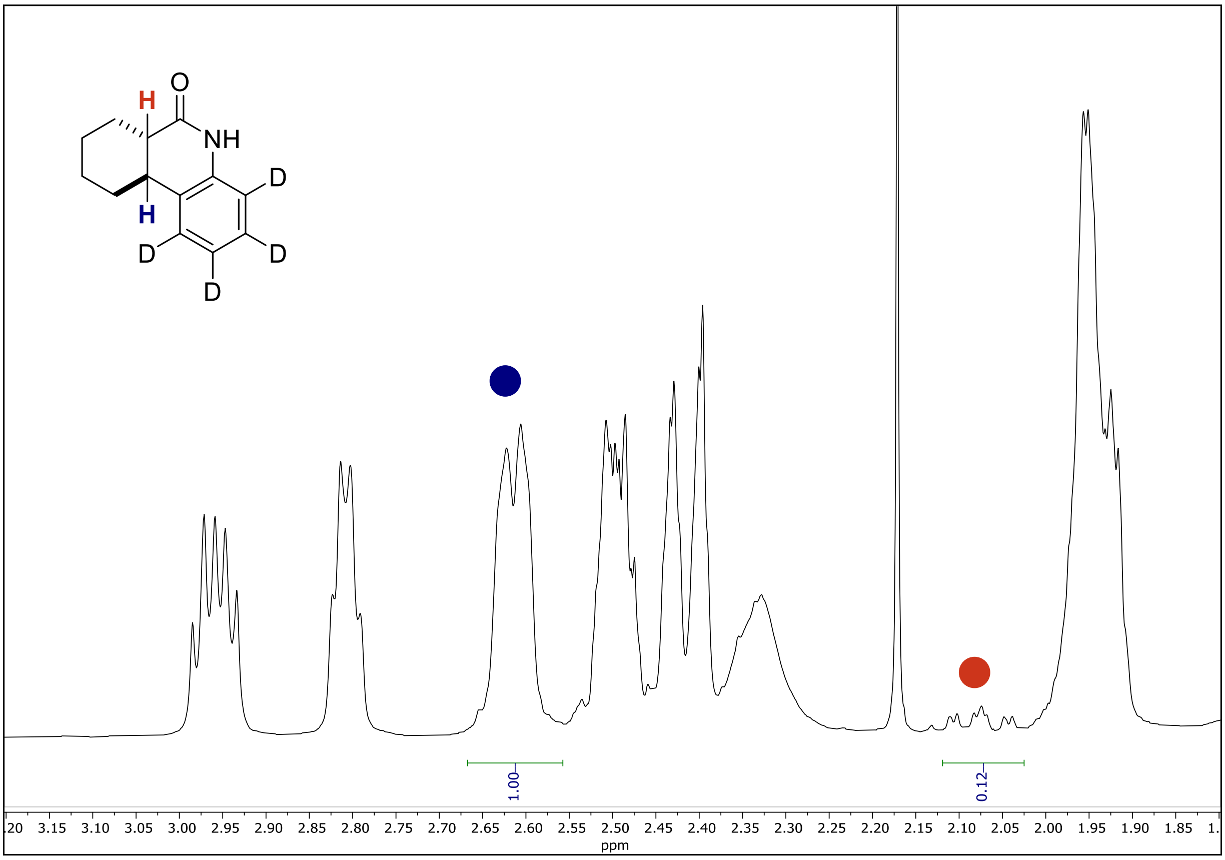

Table 8, entry 2: D-incorporation of *cis*-**2v**

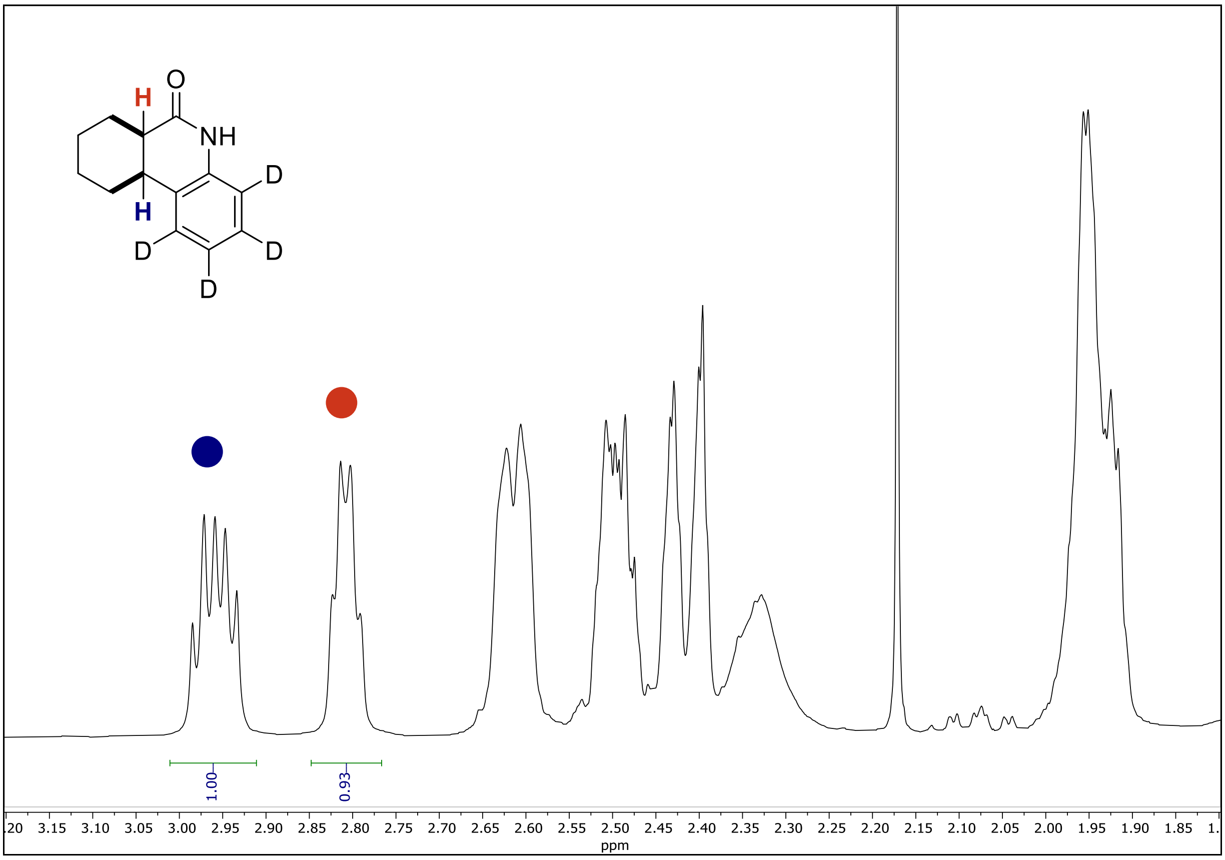

Table 9, entry 1: D-incorporation of *trans*-**2w**

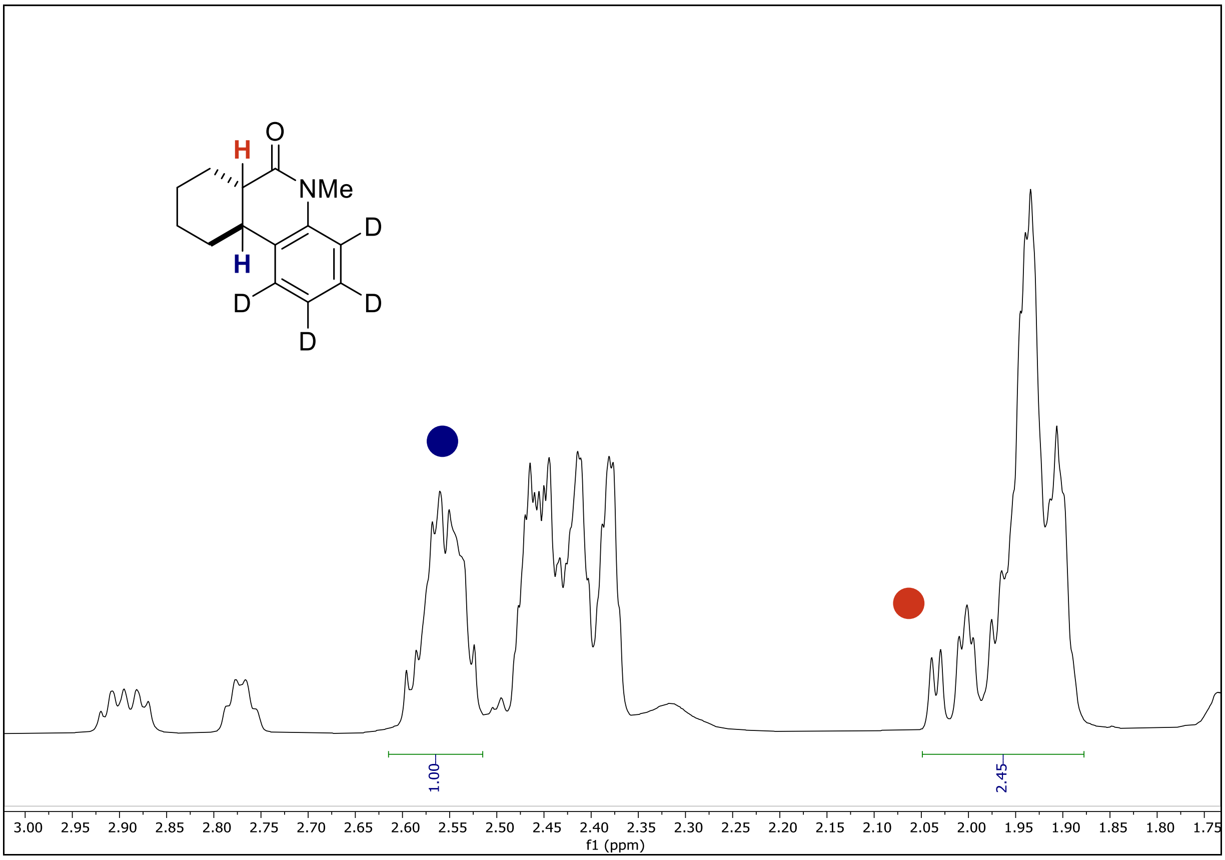

Table 9, entry 1: D-incorporation of *cis*-**2w**

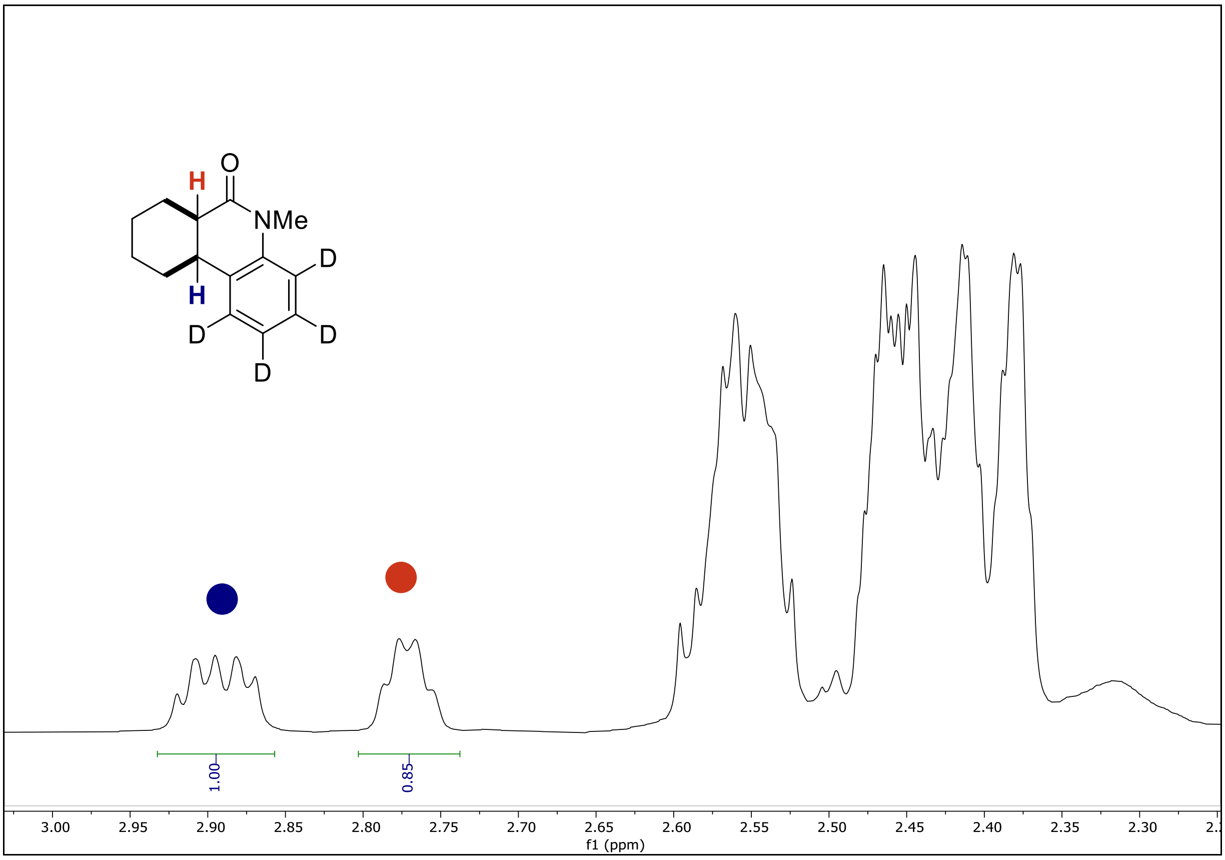

Table 9, entry 2: D-incorporation of *trans*-**2w**

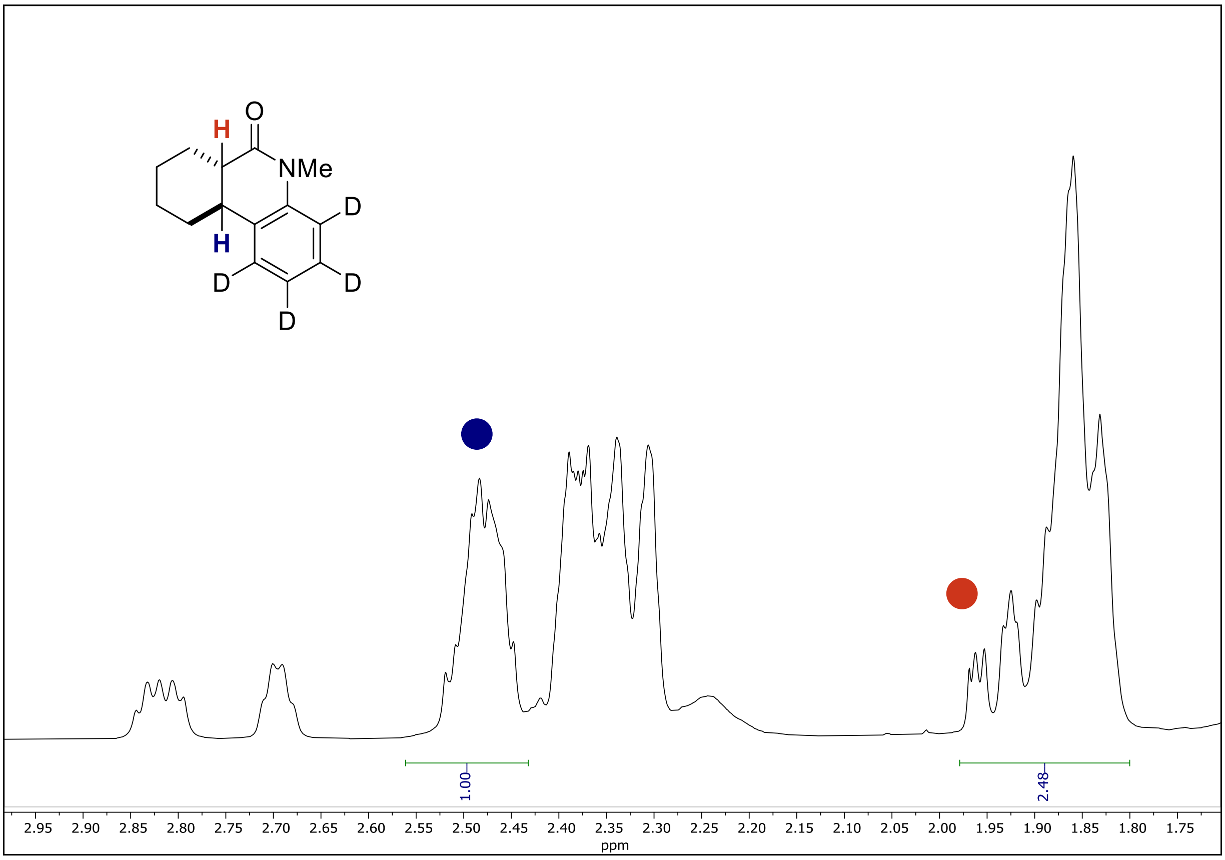

Table 9, entry 2: D-incorporation of *cis*-**2w**

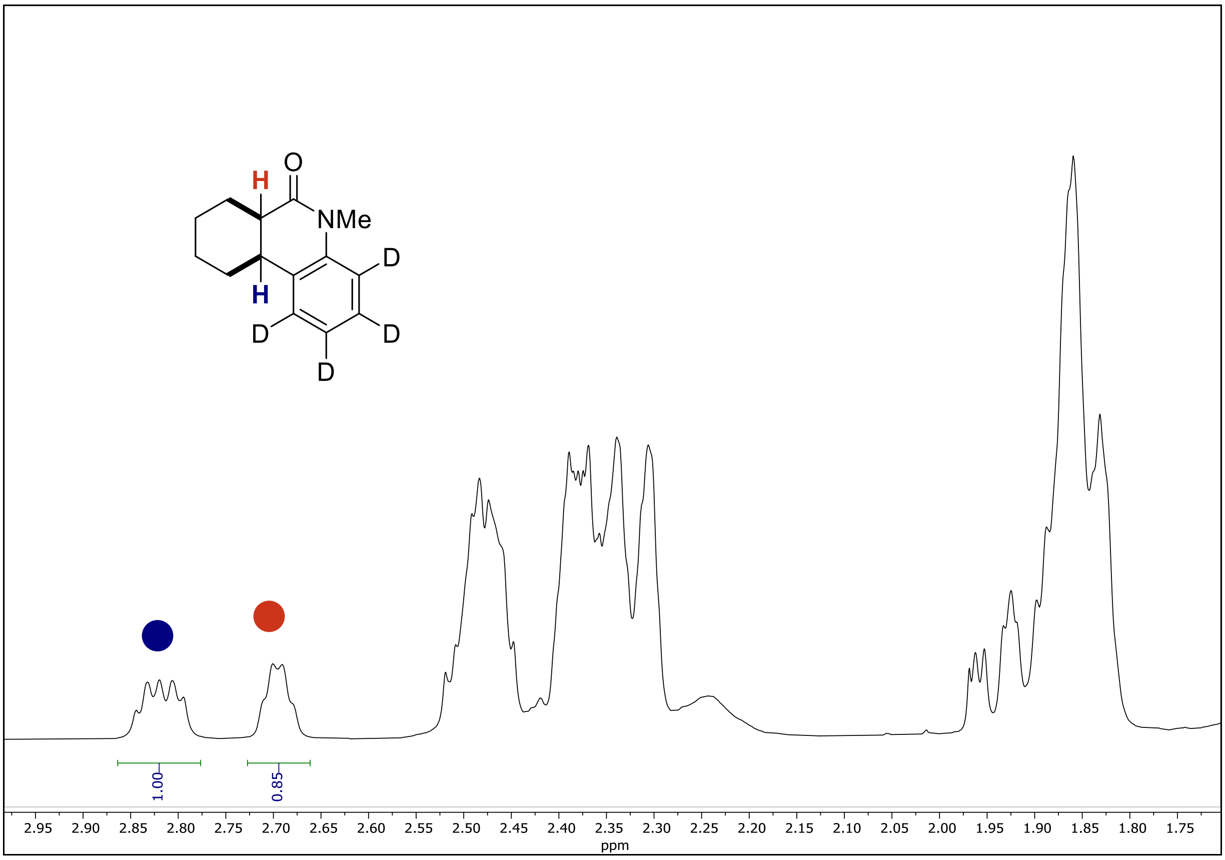

Table 9, entry 4: D-incorporation of *trans*-**2w**

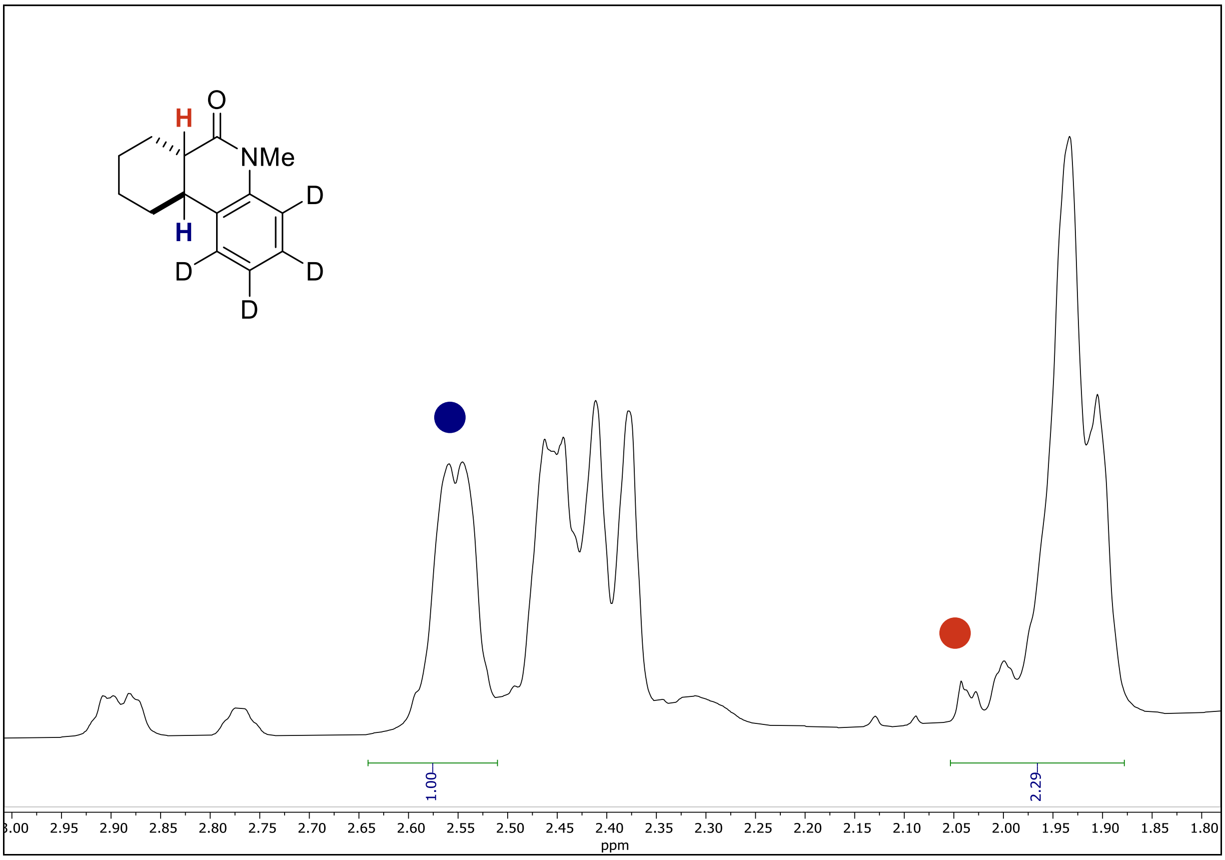

Table 9, entry 4: D-incorporation of *cis*-**2w**

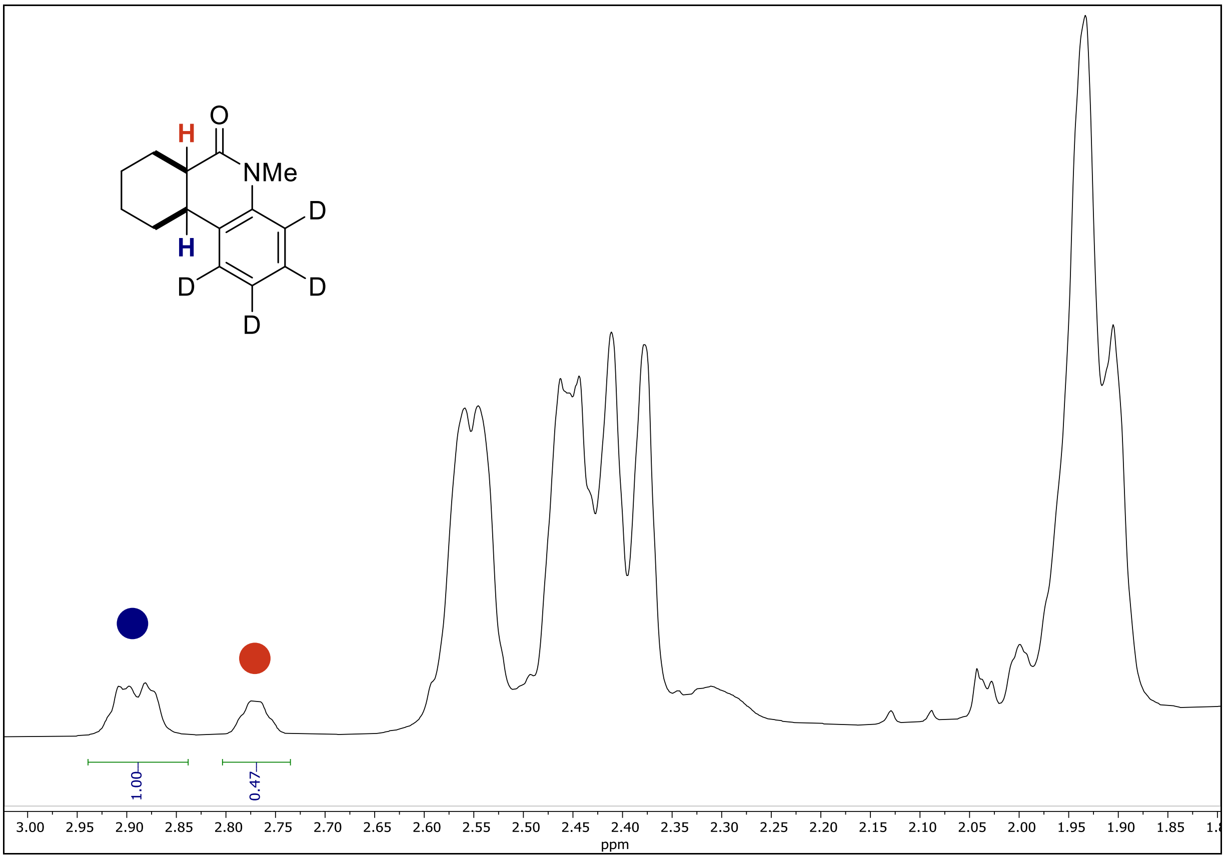

Table 9, entry 5: D-incorporation of *trans*-**2w**

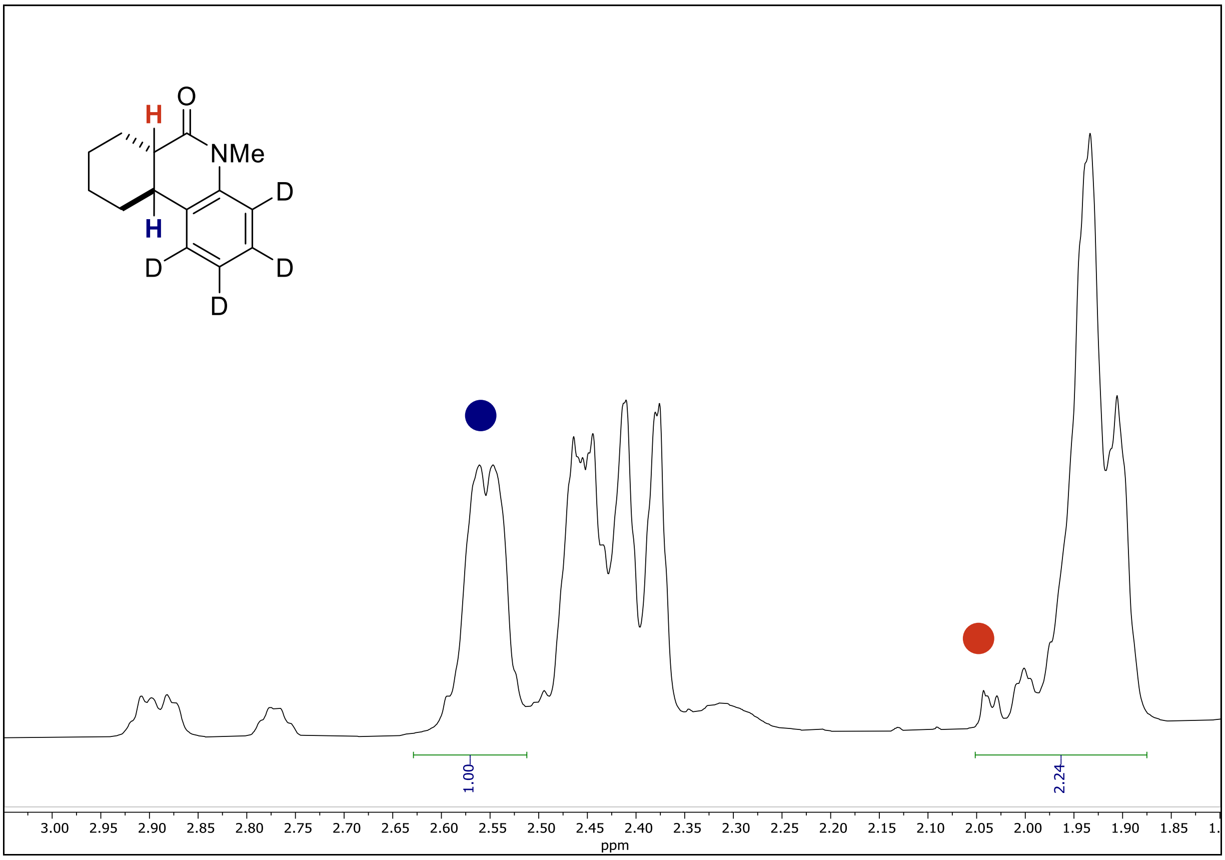

Table 9, entry 5: D-incorporation of *cis*-**2w**

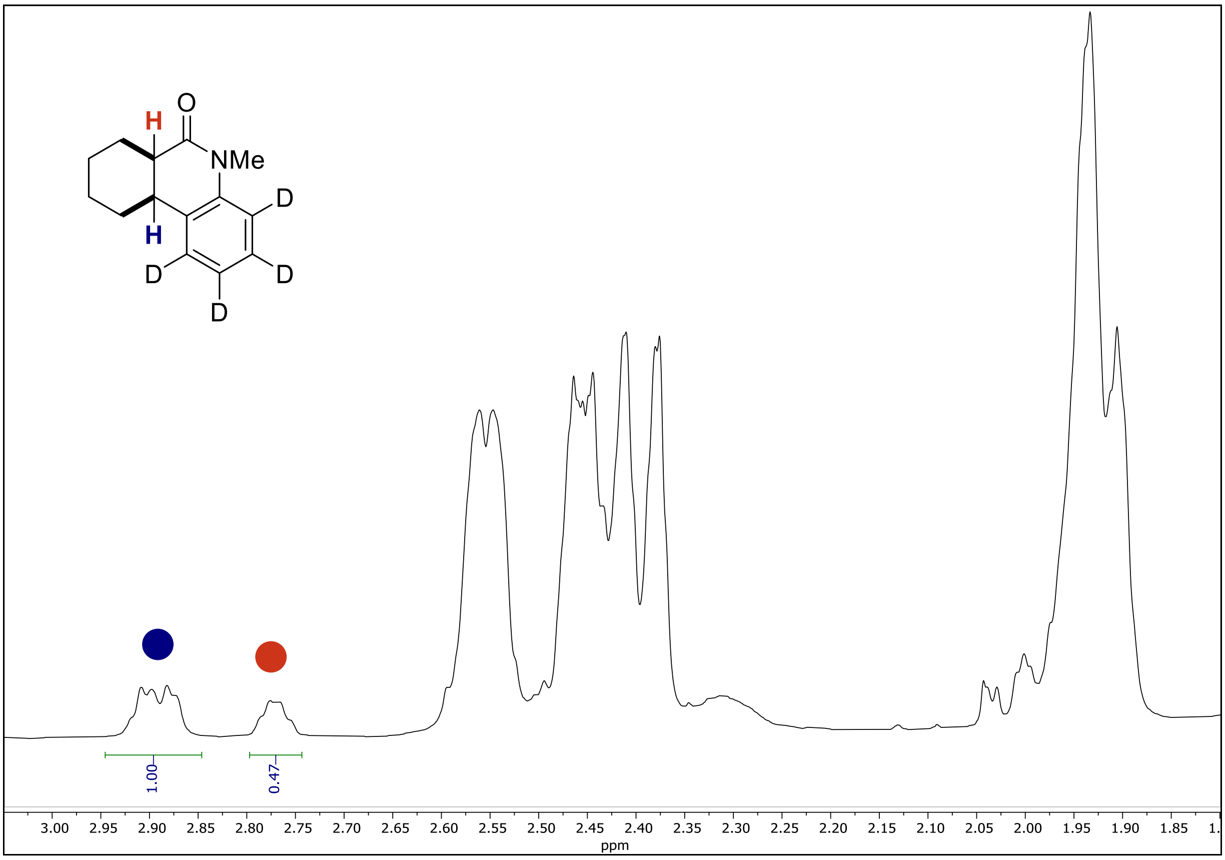

Table 9, entry 6: D-incorporation of *trans*-**2q**

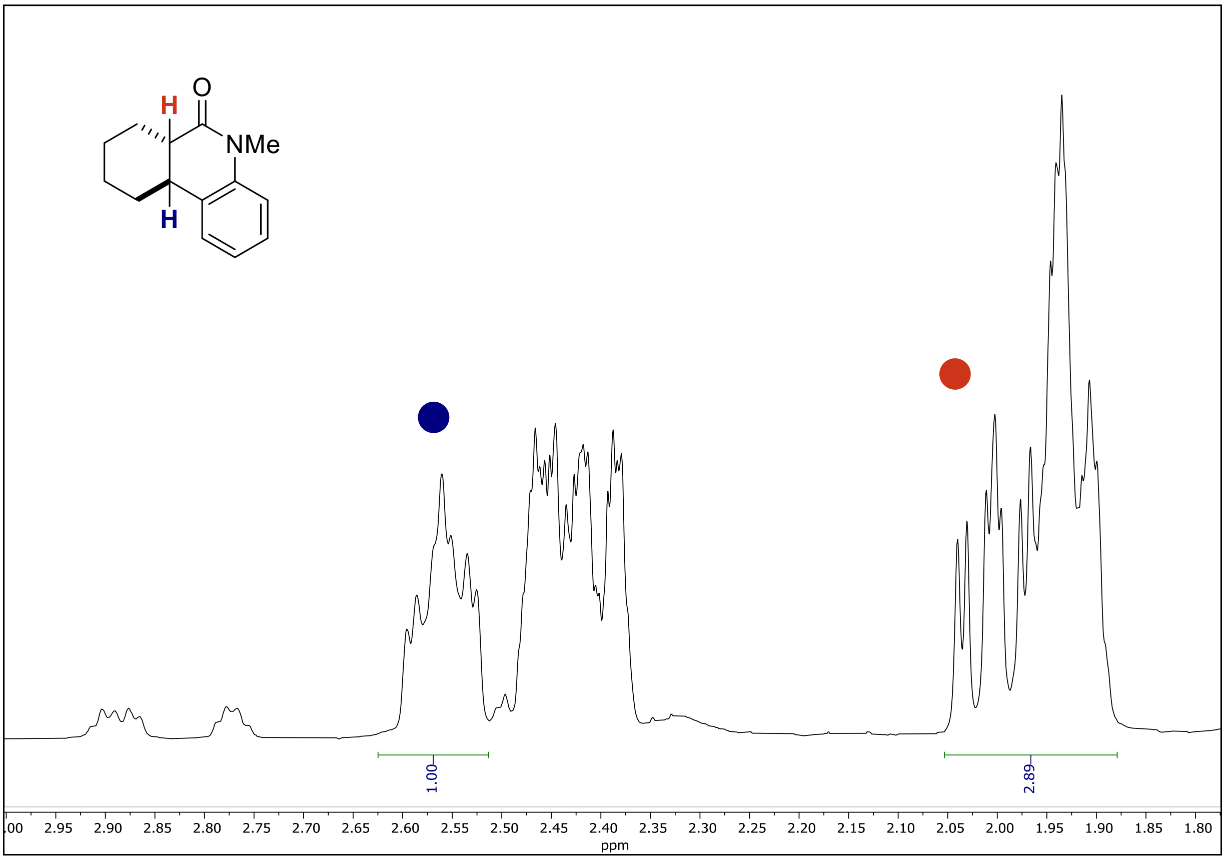

Table 9, entry 6: D-incorporation of *cis*-**2q**

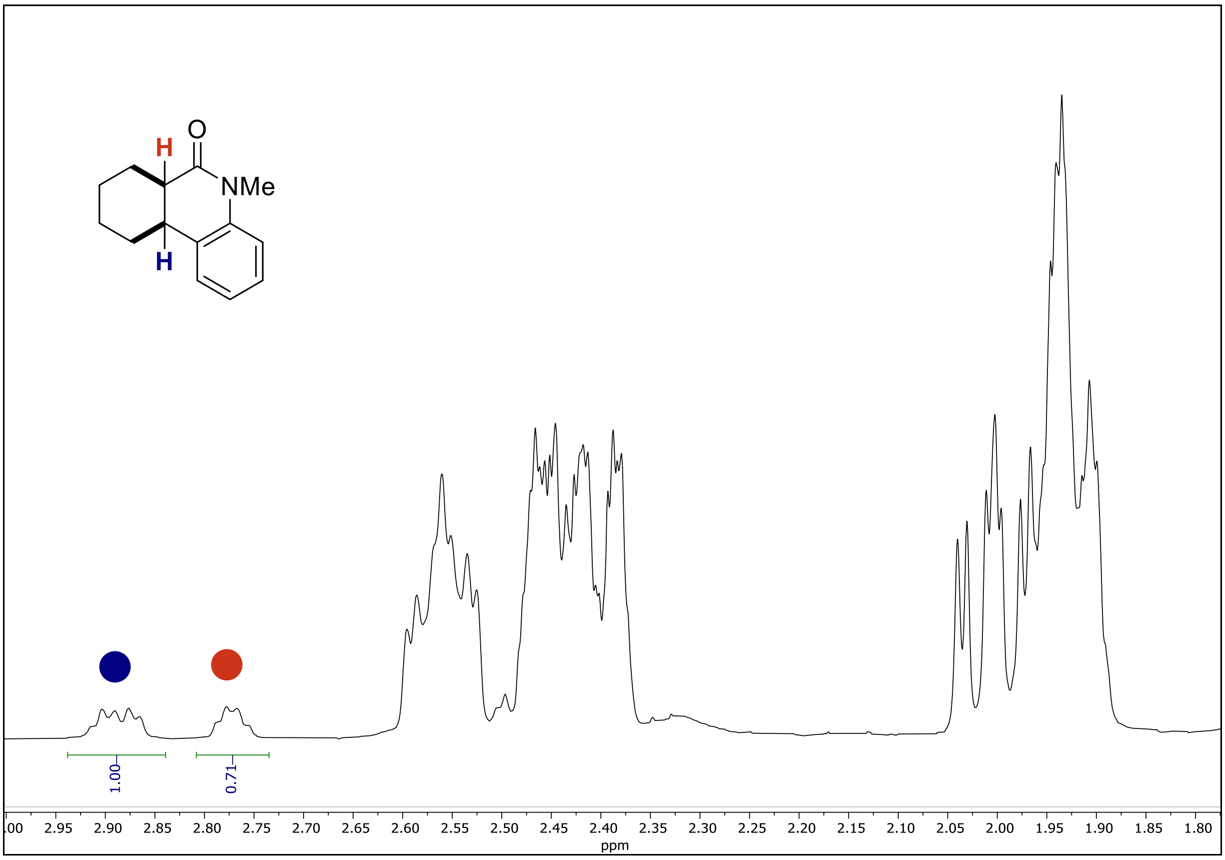

Table 9, entry 7: D-incorporation of *trans*-**2q**

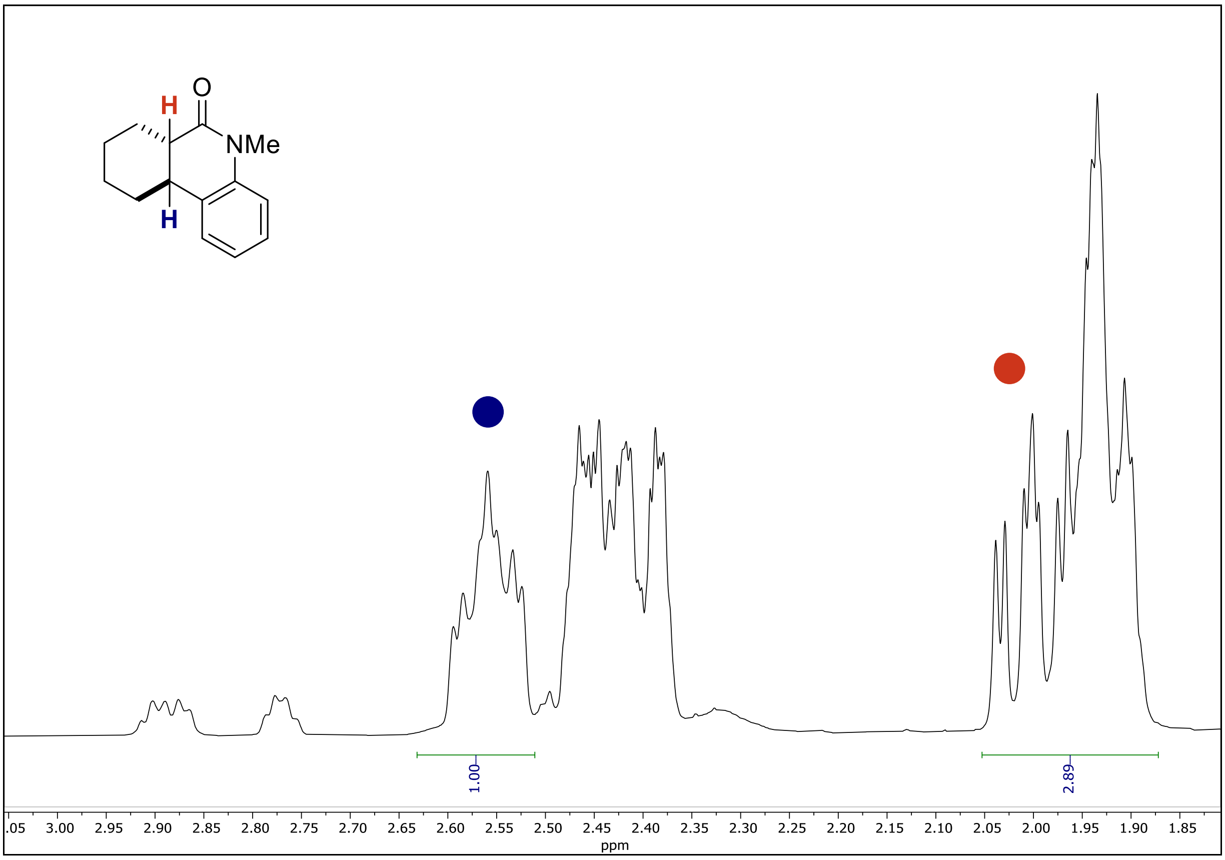

Table 9, entry 7: D-incorporation of *cis*-**2q**

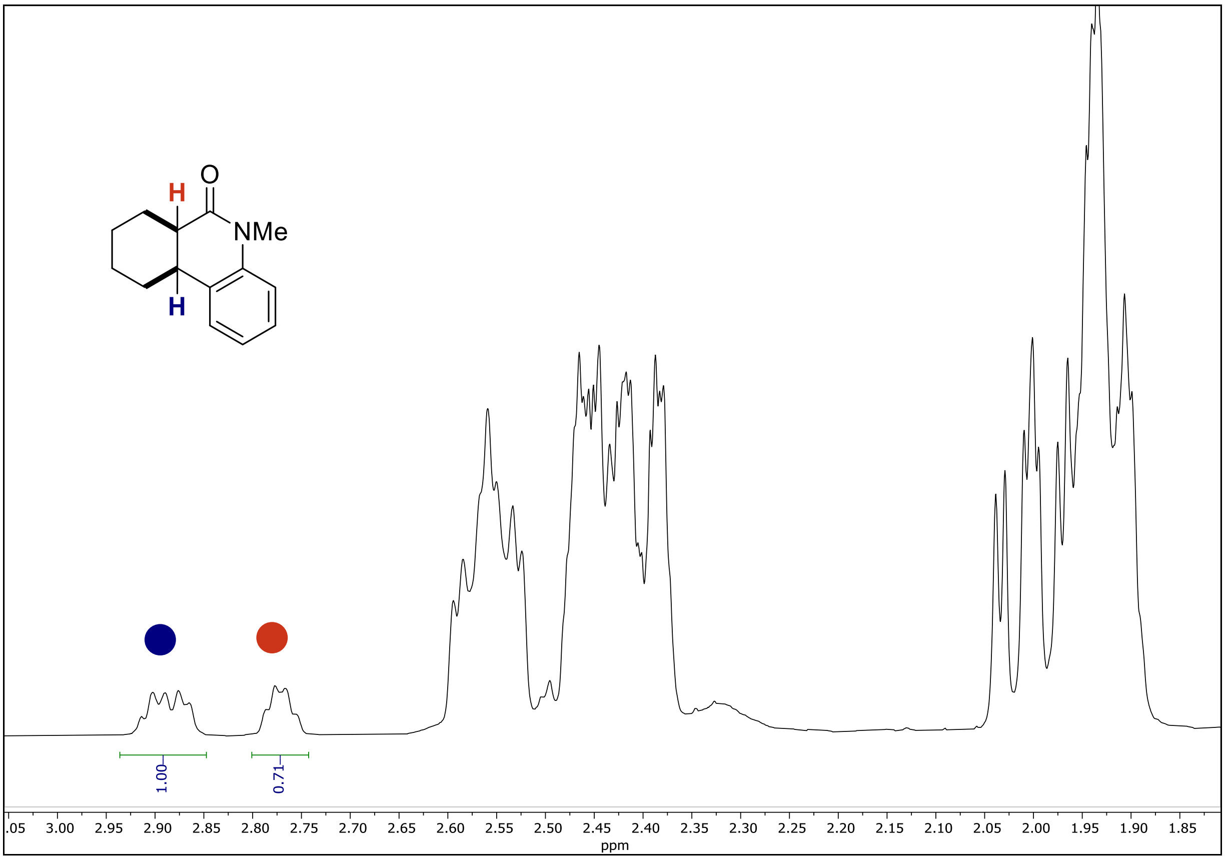

To evaluate the final steps of product formation after the cyclization took place, experiments with deuterated substrates were performed. First, a substrate with deuterated phenyl ring (**1v**) was subjected to the standard reaction conditions (SI Table 8, entry 1). A high level of deuterium incorporation was observed in the *trans*-diastereomer of the product, while D-incorporation in the *cis*-diastereomer was negligible. This indicates that the *trans*-product is formed mainly via a [1,5]-shift, while protonation from an external source seems to be operative to produce the *cis*-product. The solvent was considered as external protonation source, and the experiment was repeated in deuterated solvent (SI Table 8, entry 2). However, the observed levels of deuterium incorporation were similar to the previous experiment. The negligible role of the solvent in the protonation step was further evidenced by performing the reaction with non-deuterated substrate **1a** in deuterated solvent (SI Table 8, entry 3). No D-incorporation was observed. As alternative proton source the free NH of another substrate or product molecule was considered next, and the set of experiments was repeated with *N*-methylated substrates **1q** and **1w** (SI Table 9, entries 1 – 3, adjusted reaction conditions were applied due to previous findings, *vide supra*). Consistent with the previous results, D-incorporation in the *trans*-product was greater than in the *cis*-product and deuteration of the solvent did not have a significant influence on the reaction outcome. However, D-incorporation in *trans*-**2w** was lower than in *trans*-**2v** (55% vs 84%). This might be due to enhanced external protonation from the HFIP which is necessary to achieve satisfactory conversion when methylated amides are applied as substrates. To evaluate the potential of the hydroxy group of HFIP to function as proton source, the set of reactions was repeated using HFIP-OD instead of HFIP. As expected, the degree of deuterium incorporation was increased and significant D-incorporation was also observed in the *cis*-diastereomers of the products (SI Table 9, entries 4 and 5). This substantiates the hypothesis that the *cis*-products are formed via external protonation. Furthermore, the non-deuterated substrate **1q** was subjected to the reaction conditions applying HFIP-OD (SI Table 9, entries 6 and 7). The low level of D-incorporation found in the *trans*-diastereomer (11% D) further verifies that formation of the *trans*-product predominantly occurs via a [1,5]-shift. Contrary to our expectations, the level of D-incorporation in the *cis*-product was also decreased when the non-deuterated substrate was used. This suggests that both product formation pathways may be operative for the final step of the formation of the *cis*-diastereomer with external protonation dominating (but not eliminating) a [1,5]-H shift.

NMR Experiments

The NMR experiments were conducted with mixtures of substrate and catalyst in different ratios in the corresponding deuterated reaction solvent (0.5 mL in DCM-d_2). The ratios of substrate to catalyst (1:1, 1:2, 2:1, 1:0.1) were measured, with the catalyst in 0.025 mmol and a varied amount of substrate, except for 1:0.1 (0.025 mmol substrate, 0.0025 mmol catalyst). The ^{13}C NMR spectra were recorded and compared to those of the pure substrates.

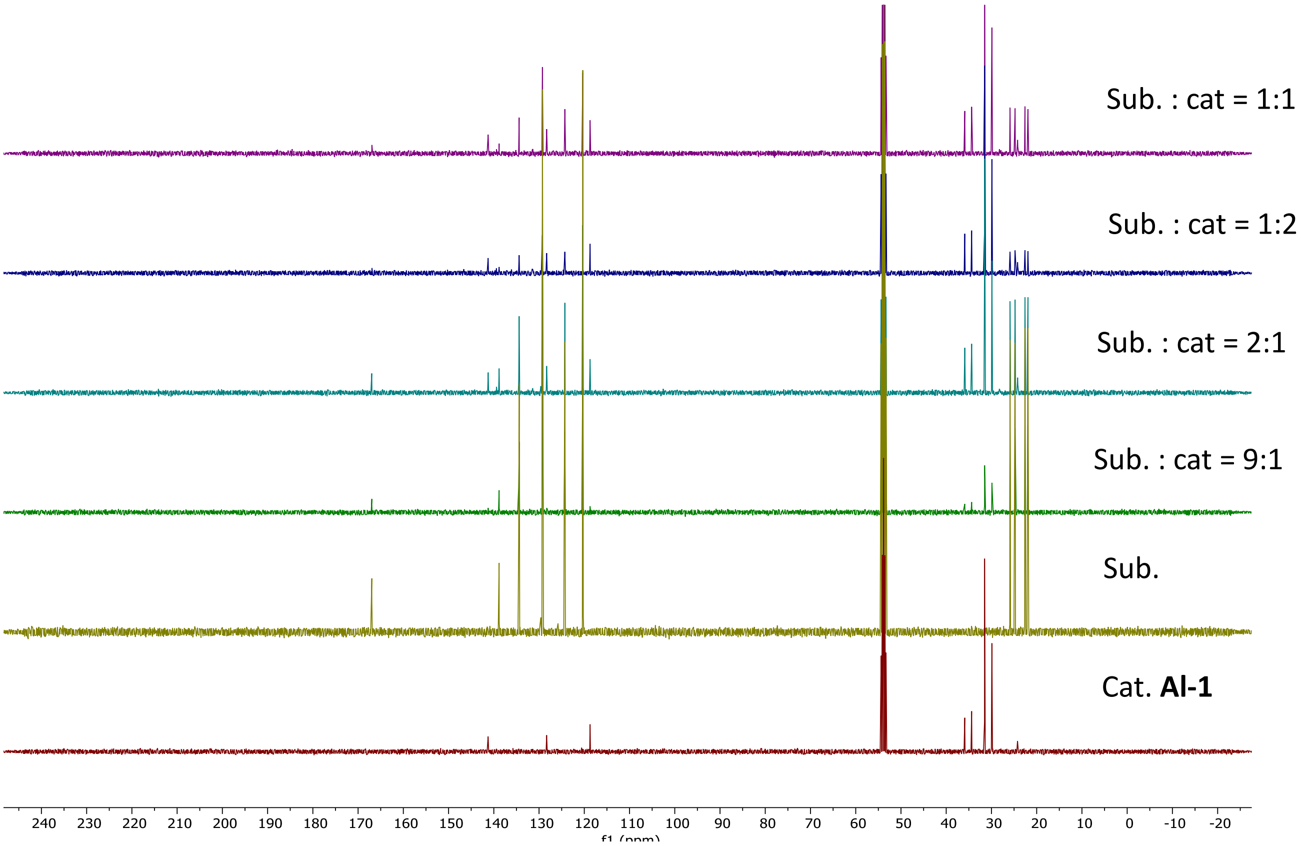

Figure 15: ^{13}C NMR spectra of substrate **1a** and **Al-1** in different ratios, measured in DCM-d_2.

No shift in the ^{13}C NMR spectra was observed, particularly the carbonyl peak, which would be coordinated by the Lewis acid, showed no shift. Therefore, Lewis acid coordination was excluded.

Non-linear effect study:

Reactions were performed according to general procedures **D** with varying *e.e.* of **Al-1**. **Al-1** with varying optical purity was obtained by mixing the optical antipodes in the corresponding ratio. The products were purified by column chromatography (SiO_2, *n*-pentane/EtOAc: 80/20). Enantiomeric ratios were determined by HPLC (column: AS-H; eluent: *n*-hexane/*i*-propanol:

15/85; flow rate: 1.0 mL/min, 35 °C). A linear correlation between *e.e.* of **Al-1** and *e.e.* of **2a** was observed. Therefore, no non-linear effect can be observed.

Table 10: Effect of *e.e.* of **Al-1** on the *e.e.* of **2a**.

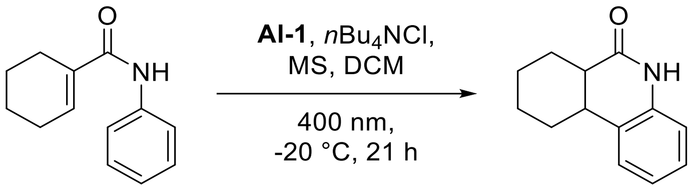

entry	*e.e.* Al-1 [%]	*trans*-diastereomer		*cis*-diastereomer	
		e.r.	*e.e.* [%]	*e.r.*	*e.e.* [%]
**1**	0	50:50	0	50:50	0
**2**	11	53:47	6	46:54	8
**3**	21	57:43	14	44:56	12
**4**	29	59:41	18	41:59	18
**5**	40	66:34	32	33:67	34
**6**	49	69:31	38	33:67	34
**7**	61	70:30	40	32:68	36
**8**	70	80:20	60	17:83	66
**9**	85	79:21	58	16:84	68
**10**	100	84:16	68	10:90	80

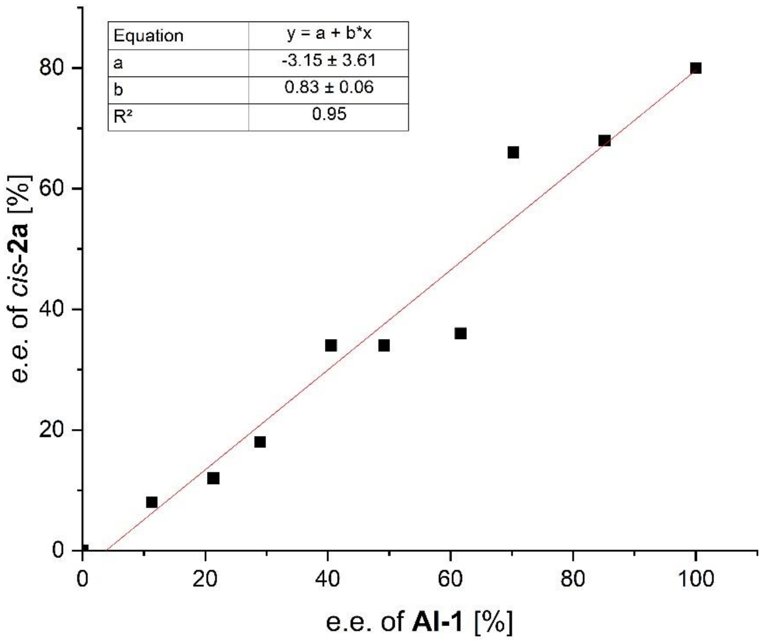

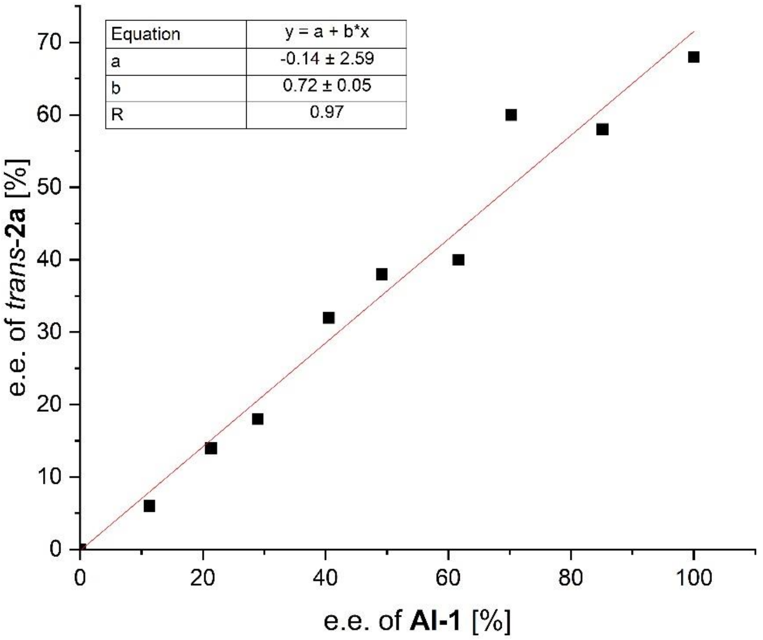

Figure 16: Plot of *e.e.* of **Al-1** against the *e.e.* of **2a**.

Computational Analysis

Computational Details

The minimum energy conformations of the catalyst-substrate complex were found by a conformational search using CREST (V. 2.12)[29,30] employing GFN2-xTB (V. 6.5.1)[31,32] calculations. Solvation effects were considered using the analytical linearized Poisson–Boltzmann (ALPB)[33] model with dichloromethane as a solvent. The results of the conformational search were refined by empirically corrected density-functional theory (DFT; PBEh- 3c)[34] and further optimized using the generalized gradient approximation (GGA) functional PBE[35] with an atom-pairwise dispersion correction with Becke–Johnson damping[36,37] and the polarized triple Zeta basis set def2-TZVP,[38] following the workflow implemented in CENSO (V. 1.2.0).[39] Following that workflow, the DFT calculations were performed with TURBOMOLE (V. 7.8)[40,41] and the direct COSMO-RS (DCOSMO-RS)[42] implicit solvation model. DCOSMO-RS does not feature dichloromethane, hence we approximated the solvent effects of dichloromethane by using chloroform. This approximation was solidified by recalculating the energies of the final conformers using the hybrid functional PW6B95(-D3)[43] for the electronic energies, COSMO-RS[44,45] using dichloromethane as a solvent at 253.15 K for the solvation energies, and PBE(-D3) for the vibrational frequencies at 253.15 K. The nature of the minimum of the conformers was proven by the absence of imaginary frequencies larger (in absolute value) than 10 cm^{-1}. The conformers lowest in energy, which were used for further calculations in this work, did not show any imaginary frequencies. Only those conformers were considered that were in an energy range of 2 kcal/mol relative to the conformer lowest in energy.

The orbital transitions in the investigation of charge transfer excitations were obtained from linear-response time-dependent density-functional theory (TDDFT) using the range-separated hybrid functional CAM-B3LYP[46] and the def2-TZVP basis set. This calculation was performed using SERENITY (V. 1.6.1).[47–49]

The ground state redox potentials were calculated according to reference [50] using the range-separated hybrid functional CAM-B3LYP(-D3) and the def2-TZVP basis set. For this purpose, all structures used were optimized using this functional and confirmed to have no imaginary frequencies. These calculations were performed using ORCA (V. 5.0.3).[51] Solvation effects were considered using COSMO-RS. Excited state redox potentials were approximated using

the adiabatic electronic excitation energies.[52,53] For the T_1 state this energy was obtained by a ΔSCF like approach, for the S_1 state this energy was obtained by TDDFT calculations.

Electronic couplings were calculated from a projection based subsystem TDDFT (sTDDFT) calculation performed using SERENITY following the procedure in reference [54]. For this calculation the CAM-B3LYP functional and the def2-SVP[38] basis set were employed. The non-additive exchange-correlation functional was approximated using CAM-B3LYP. Additionally, a level-shift operator was used to ensure external orthogonality. The couplings were obtained from a coupled (FDEc-TDDFT) calculation. These sTDDFT calculations employed the Tamm–Dancoff approximation (TDA). For this purpose the lowest energy conformer of the conformational search was used.

For the calculations of the energies of the triplet-state minimum structures a reoptimization using the B3LYP(-D3)[55,56] functional and the def2-TZVP basis set was performed. The lowest energy conformer of a conformational search was used, in case the system was previously optimized by a conformational search. The minima were confirmed by the absence of any imaginary frequencies. Electronic energies at the optimized structures were recalculated using the range-separated hybrid functionals ωB97X-D3[57] and CAM-B3LYP(-D3), the hybrid functional PW6B95(-D3) and the def2-TZVPP[38] basis set. Solvation effects were considered during the optimization and recalculation of the electronic energy via SMD[58] using dichloromethane as solvent. These calculations were performed using ORCA.

The energy barrier for the energy transfer was calculated using Marcus theory similar to the description in reference [59]. The energies were calculated at B3LYP(-D3) optimized structures employing the def2-TZVP basis set. Frequencies were calculated using the same functional used for the optimization and the minima were confirmed to not have any imaginary frequencies. Solvation effects were considered using COSMO-RS and dichloromethane as a solvent. The temperature was set to 253.15 K. Contrary to the workflow shown in reference [59] the counterpart energies – the energies of the triplet state equilibrium geometry in the singlet state and the singlet equilibrium geometry in the triplet state – are pure electronic energies.

Minimum energy crossing points (MECP) were optimized using CAS(6,5)SCF and the valence double Zeta ANO-S basis set.[60] The active space was chosen based on orbitals obtained from a minimal basis ANO-S calculation and subsequent basis set expansion. These calculations

were performed using OpenMolcas (V. 24.02).[61] The spin densities shown in SI Figs. 21 and 23 were obtained from these calculations. Furthermore, the optimization of MECPs was started from preoptimized structures. These preoptimizations were conducted using the workflow described in reference [62].

Specific optical rotations were calculated at a wavelength of 589 nm using the TDDFT implementation of SERENITY employing the CAM-B3LYP functional and def2-TZVP basis set. To compensate for the flexible ring, molecular structures of the investigated compounds were obtained from a conformational search and a Boltzmann weighted optical rotation was calculated. The conformational search was conducted following the above outlined workflow. However, chloroform was used as a solvent in all steps following the experimental setup. Additionally, the recalculation of the electronic energy was performed using DLPNO-CCSD(T0) instead of a DFT method to ensure more reliable energies. The coupled cluster calculations employed the def2-TZVP basis set and were performed with SERENITY.

Conformational Search

Table 11: Energies of the conformational ensemble of the catalyst-substrate system in the ground state.

Conformer	E_{el} (PBE-D3 / def2-TZVP) [E_h]	$G^{rrho}_{253.15}$ (PBE-D3 / def2-TZVP) [kcal/mol]	E_{el} (PW6B95-D3 / def2-TZVP) [E_h]	$G^{s}_{253.15}$, DCM (COSMO-RS) [kcal/mol]	$\Delta G^{solv}_{253.15}$ [kcal/mol]
**GS1**	-2998.160234	601.007	-3004.934896	-35.383	0.0
**GS2**	-2998.160583	601.767	-3004.935869	-35.519	0.0
**GS3**	-2998.159545	601.239	-3004.934363	-35.629	0.3
**GS4**	-2998.160608	602.123	-3004.935598	-35.523	0.5
**GS5**	-2998.159271	600.823	-3004.933615	-35.536	0.5
**GS6**	-2998.159221	600.868	-3004.933583	-35.448	0.6
**GS7**	-2998.159209	601.274	-3004.933661	-35.637	0.8
**GS8**	-2998.159848	602.005	-3004.934874	-35.334	1.1
**GS9**	-2998.158642	601.540	-3004.933517	-35.698	1.1
**GS10**	-2998.157772	600.668	-3004.932366	-35.501	1.1
**GS11**	-2998.158745	601.954	-3004.933885	-35.717	1.2
**GS12**	-2998.157801	601.116	-3004.932765	-35.605	1.2
**GS13**	-2998.160574	602.638	-3004.935228	-35.544	1.3
**GS14**	-2998.158985	601.215	-3004.932957	-35.512	1.3
**GS15**	-2998.15973	602.400	-3004.934936	-35.358	1.4
**GS16**	-2998.158426	601.732	-3004.933207	-35.812	1.4
**GS17**	-2998.159095	602.384	-3004.933952	-35.733	1.6
**GS18**	-2998.159439	602.242	-3004.933654	-35.758	1.6
**GS19**	-2998.158884	601.871	-3004.933742	-35.347	1.6
**GS20**	-2998.159170	601.968	-3004.933677	-35.382	1.7
**GS21**	-2998.160187	602.252	-3004.934242	-35.226	1.8
**GS22**	-2998.156542	600.773	-3004.930270	-36.286	1.8
**GS23**	-2998.158500	602.254	-3004.933257	-35.740	1.9
**GS24**	-2998.158404	601.918	-3004.933146	-35.481	1.9

Table 12: Energies of the conformer of the catalyst-substrate system in the T_1 state.

Conformer	E_{el} (PBE-D3/ def2-TZVP) [E_h]	$G_{253.15}^{\mathrm{rrho}}$ (PBE-D3/ def2-TZVP) [kcal/mol]	E_{el} (PW6B95-D3 / def2-TZVP) [E_h]	$G_{253.15}^{\mathrm{s}}$, DCM (COSMO-RS) [kcal/mol]	$\Delta G_{253.15}^{\mathrm{solv}}$ [kcal/mol]
**IM1**	-2998.073009	600.991	-3004.832981	-37.825	0.0

The conformational search in the T_1 state resulted in one viable conformer as the others were significantly higher in energy and sorted out during the conformational sampling.

Electron Transfer Investigation

Charge-Transfer Excitation

Table 13: Significant contributions to the S_1 and the S_2 states of the catalyst substrate system obtained by TDDFT using the polarized triple Zeta basis set def2-TZVP and the CAM-B3LYP range–separated hybrid functional.

Excited State	Excitation Energy / nm	Orbital Transition	Contribution/%
1	347.38	HOMO → LUMO	51.78
		HOMO → LUMO + 1	32.99
		HOMO – 1 → LUMO + 1	7.93
2	326.38	HOMO – 1 → LUMO	63.74
		HOMO → LUMO + 1	17.10
		HOMO –1 → LUMO + 1	12.94

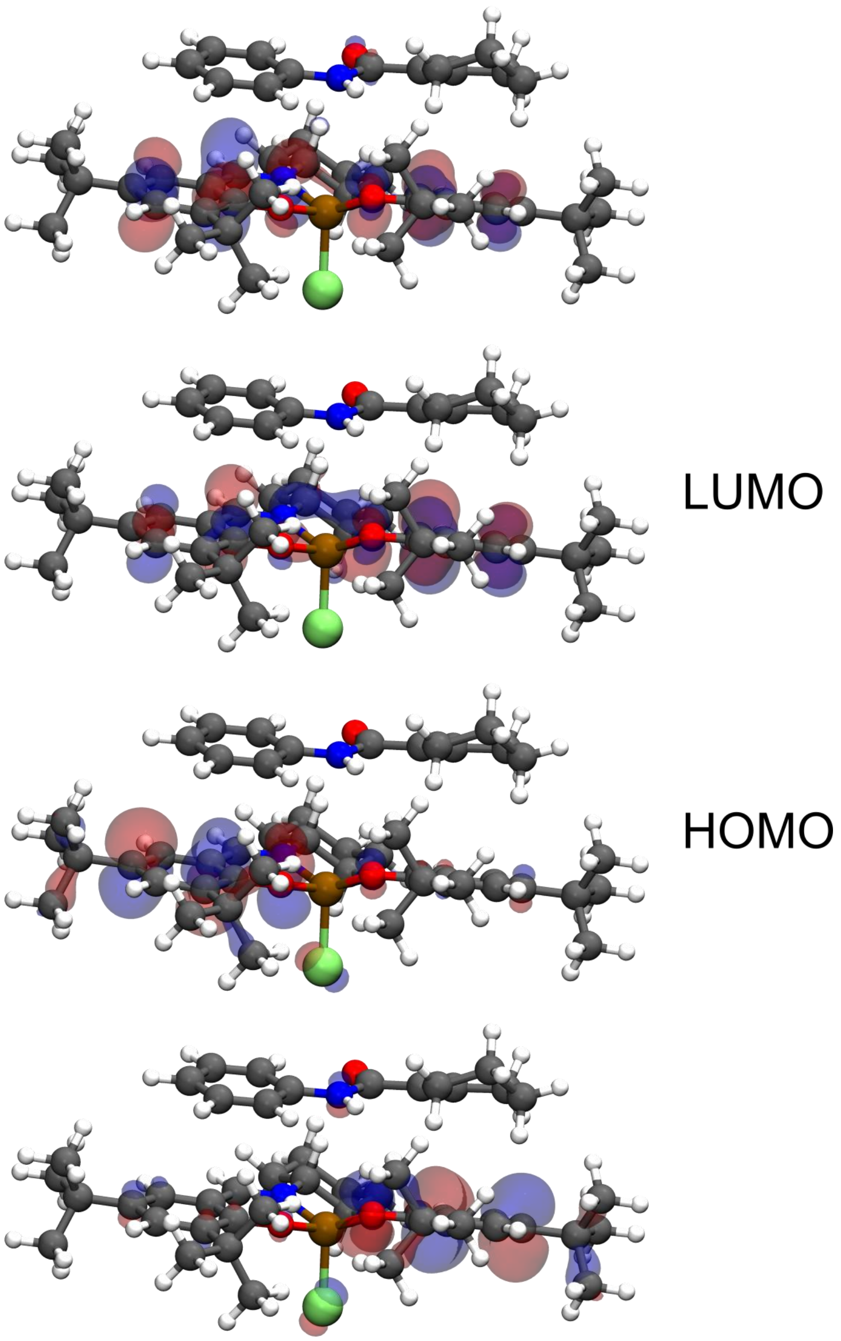

Figure 17: Frontier orbitals of the catalyst-substrate system at the **GS1** structure using the CAM-B3LYP functional and def2-TZVP basis set.

Redox Potentials

The redox potentials are calculated relative to the saturated calomel electrode (SCE). The absolute potential of SCE was calculated as a sum of the absolute potential of the standard hydrogen electrode (4.281 V) in acetonitrile and a conversion factor (0.241 V) according to reference [63] at 25 °C. This reference potential was approximated to also be the reference potential in dichloromethane at –20 °C. The excited state redox potentials were approximated

by subtracting the adiabatic electronic excitation energy from the ground state potential. The molecular structures were optimized using the CAM-B3LYP(-D3) functional and def2-TZVP basis set (**CatRshS0**, **CatRshS1**, **CatRshT1**, **CatRshCation**, **SubRshS0**, **SubRshAnion**).

Table 14: Calculated redox potentials relative to SCE, adiabatic excitation energies, and approximated excited state redox potentials relative to SCE. Oxidation potentials are given for the catalyst and reduction potentials for the substrate. The reference measurements are taken from reference [64].

$E_{\mathrm{Ox,MeCN}}^{\mathrm{S_0,298}}$ / V	1.53
$E_{\mathrm{Red,MeCN}}^{\mathrm{S_0,298}}$ / V	−2.55
$E_{\mathrm{0-0,catalyst}}^{\mathrm{S_0 \to S_1}}$ / eV	3.47
$E_{\mathrm{0-0,catalyst}}^{\mathrm{S_0 \to T_1}}$ / eV	2.30
$E_{\mathrm{Ox,MeCN}}^{\mathrm{S_1,298}}$ / V	−1.94
$E_{\mathrm{Ox,MeCN}}^{\mathrm{T_1,298}}$ / V	−0.77
$E_{\mathrm{Ox,ref}}^{\mathrm{S_0}}$ / V	1.44
$E_{\mathrm{Ox,ref}}^{\mathrm{S_1}}$ / V	−1.47
$E_{\mathrm{0-0,catalyst,ref}}^{\mathrm{S_0 \to S_1}}$/ eV	2.91

Table 15: Calculated redox potentials relative to SCE. Oxidation potentials are given for the catalyst and reduction potentials are given for the substrate. The redox potentials were calculated at reaction conditions (−20 °C and in DCM).

$E_{\mathrm{Ox,DCM}}^{\mathrm{S_0,253}}$/ V	1.35
$E_{\mathrm{Red,DCM}}^{\mathrm{S_0,253}}$/ V	−2.62
$E_{\mathrm{Ox,DCM}}^{\mathrm{S_1,253}}$/ V	−2.12
$E_{\mathrm{Ox,DCM}}^{\mathrm{T_1,253}}$/ V	−0.95

Energy Transfer Investigation

Electronic Couplings

The electronic coupling for the catalyst-substrate complex investigated in this work will be compared with a similar complex from a previous study[2] that has been shown to undergo electron transfer rather than energy transfer. The reference complex consists of the same

catalyst but with a different substrate. Additionally, the geometry optimization conducted in the previous work is similar to the one conducted in this work.

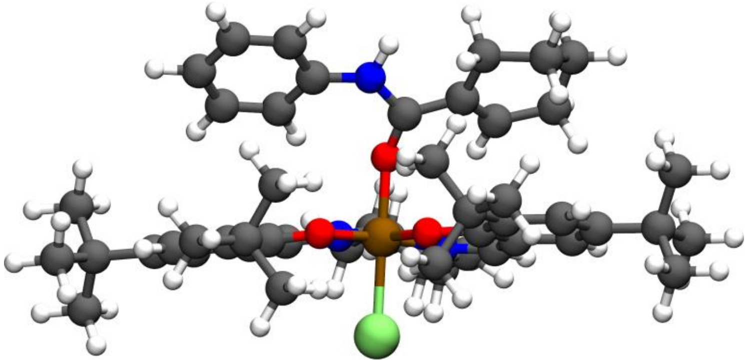

Figure 18: Molecular structure for the T_1 minimum of the substrate-catalyst complex investigated in this work (**IM1**).

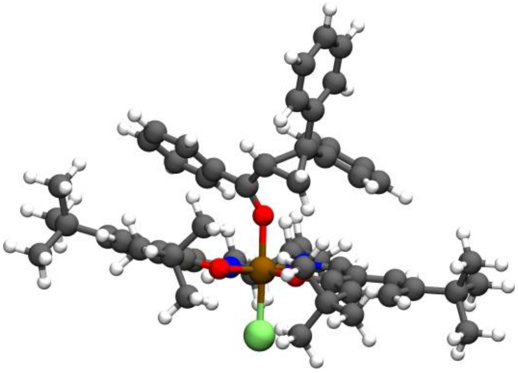

Figure 19: Molecular structure for the T_1 minimum of the reference complex. This structure has been taken from reference [2].

Table 16: Triplet transition electronic coupling and excitations energies from a PbE-sTDDFT calculation using the CAM-B3LYP functional and def2-SVP basis set. Each complex was divided into two subsystems which included the catalyst and substrate (divided between aluminium and the substrate oxygen). The given excitation energies and couplings were obtained by an FDEc calculation. The reference structure was taken from reference [2].

Catalyst substrate complex	Excitation energy substrate system / nm	Excitation energy catalyst system / nm	Coupling / eV
this work (**IM1**)	395.8	356.5	0.145
reference	345.6	349.9	0.002

Triplet Energy Differences

The triplet electronic energies and Gibbs free energies were determined for multiple systems at different structures, namely the catalyst (**CatT**), the catalyst-substrate complex (**CompT**), the substrate (**SubT**), and a model complex composed of $AlMe_3$ and the substrate (**ModelT**). All these structures are T_1 minimum energy structures. The calculated energies are adiabatic excitation energies and the ground state energies were calculated at the S_0 minimum energy structures (**CatS**, **CompS**, **SubS**, **ModelS**). The model system was used to approximate the effect of coordination on a substrate-localized triplet state. For this the model system was necessary since the substrate-localized triplet state could not be calculated using the catalyst-substrate system and DFT methods.

Table 17: ΔG of the S_0 minimum and T_1 minimum structures. The investigated systems are the catalyst, catalyst-substrate complex, substrate, and $AlMe_3$-substrate complex. The thermochemical contributions in all results were obtained from B3LYP-D3 calculations employing the def2-TZVP basis set. The solvation effects were considered using SMD. The calculations were performed at 253.15 K. The electronic energies were calculated using the given functional and the def2-TZVPP basis set.

State	ΔG / eV ωB97XD-3	ΔG / eV PW6B95-D3	ΔG / eV CAM-B3LYP-D3	Character
T_1^{catalyst}	2.34	2.27	2.29	catalyst-localized
$T_1^{\text{catalyst}-\text{substrate}}$	2.56	2.38	2.47	catalyst-localized
$T_1^{\text{substrate}}$	2.54	2.63	2.52	substrate-localized
$T_1^{\text{AlMe}_3-\text{substrate}}$	2.50	2.56	2.48	substrate-localized

Table 18: Electronic energy differences between the S_0 minimum and T_1 minimum. The investigated systems are the catalyst, catalyst-substrate complex, substrate, and $AlMe_3$-substrate complex. The energies were obtained using the given functional and the def2-TZVPP basis set. During the calculations solvation effects were considered using SMD.

State	E^{0-0} / eV ωB97X-D3	E^{0-0} / eV PW6B95-D3	E^{0-0} / eV CAM-B3LYP-D3	Character
T_1^{catalyst}	2.49	2.42	2.44	catalyst-localized
$T_1^{\text{catalyst}-\text{substrate}}$	2.71	2.53	2.62	catalyst-localized
$T_1^{\text{substrate}}$	2.66	2.74	2.64	substrate-localized
$T_1^{\text{AlMe}_3-\text{substrate}}$	2.59	2.65	2.57	substrate-localized

Energy Barrier from Marcus Theory

The energy barrier was calculated at B3LYP(-D3) optimized structures. Note that the structures used here are not the same as in the calculation of the triplet energies. The change in the optimization consists in the consideration of the solvation effect in the calculation which was not done here. Solvation effects for the calculation of $\Delta_R G$ were considered using COSMO-RS with the optimized structure (**CatMarcusS0**, **CatMarcusT1**, **SubMarcusS0**, **SubMarcusT1**). The obtained energy difference is 0.82 eV. This is an approximation to ΔE_{MECP}. ΔE_{ISC} was calculated to be 1.18 eV. This energy difference was obtained from an S_1 energy and a T_1 energy (**CompS1** and **CompT**, respectively). Both these energies were obtained using the CAM-B3LYP(-D3) functional and the def2-TZVPP basis set. The geometries were obtained by a TDDFT optimization of the S_1 state employing the CAM-B3LYP(-D3) functional and def2-TZVP basis set. No frequencies were calculated for the S_1 state. This shows that ΔE_{ISC} is larger than ΔE_{MECP}. Thus, the energy barrier can be overcome by the energy released in the intersystem crossing.

Spin Density Distribution

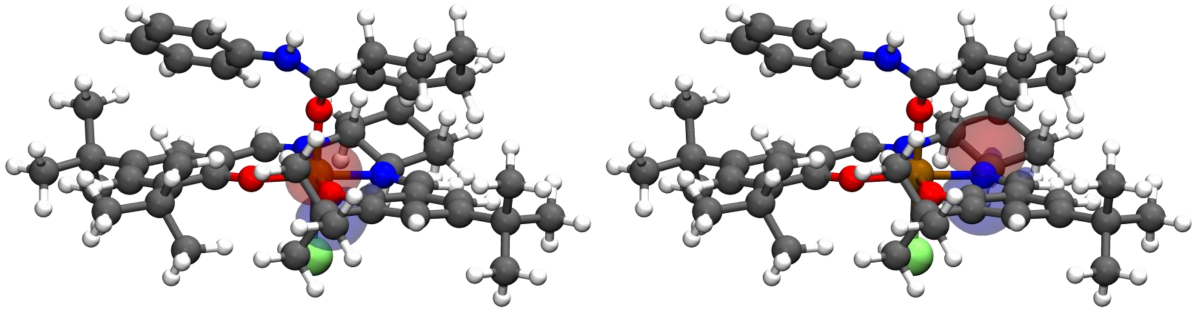

Figure 20: Orbitals involved in the active space that are localized on the catalyst. These orbitals were obtained by a minimal basis ANO-S Hartree–Fock SCF and subsequent localization on the **IM1** structure.

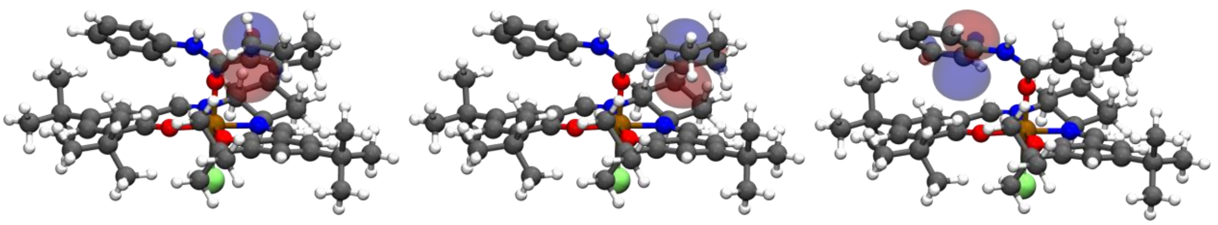

Figure 21: Orbitals involved in the active space that are localized on the substrate. These orbitals were obtained by a minimal basis ANO-S Hartree–Fock SCF and subsequent localization on the **IM1** structure.

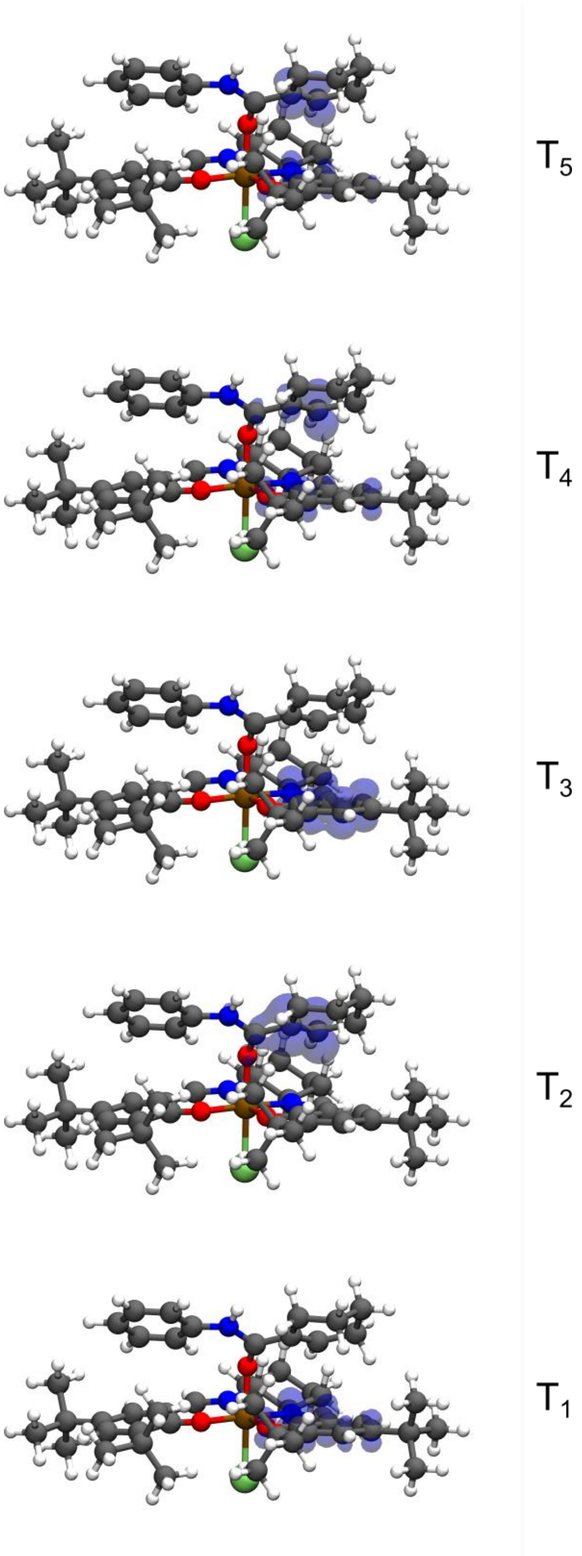

Figure 22: Spin densities obtained for the first five triplet states of the catalyst-substrate complex (**IM1**). The densities were obtained from a CAS(6,5)SCF calculation using the ANO-S valence double Zeta basis set.

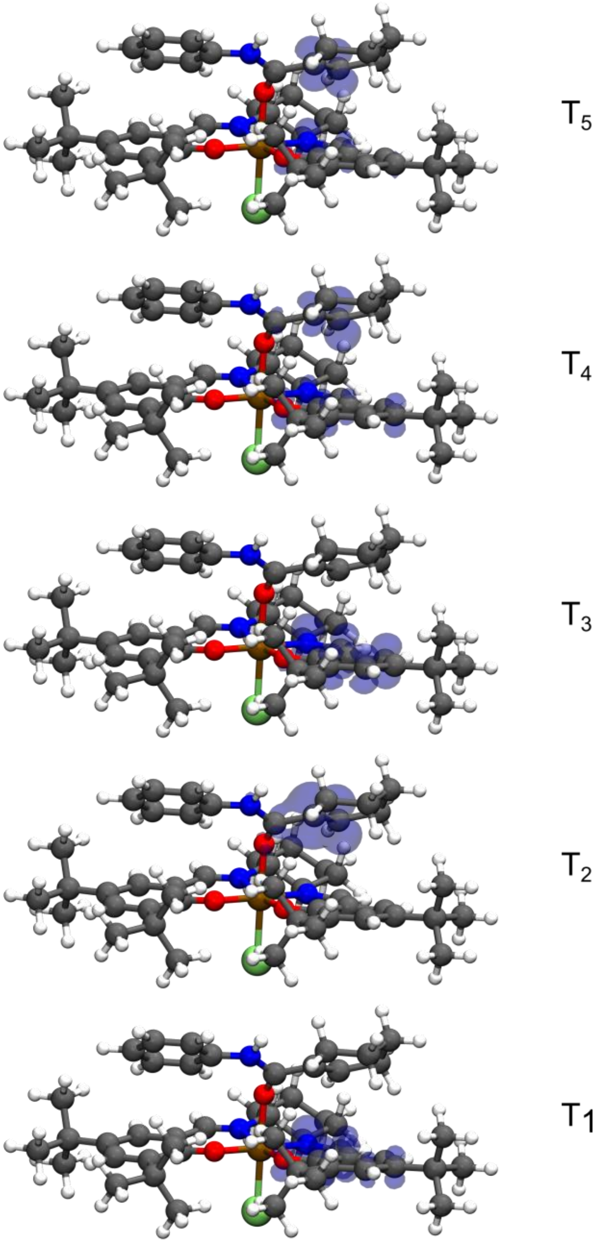

Figure 23: Spin densities obtained of the first five triplet states of the catalyst-substrate complex (**CompT**). The densities were obtained from a CAS(6,5)SCF calculation using the ANO-S valence double Zeta basis set.

Minimum Energy Crossing Point between First and Second Triplet State

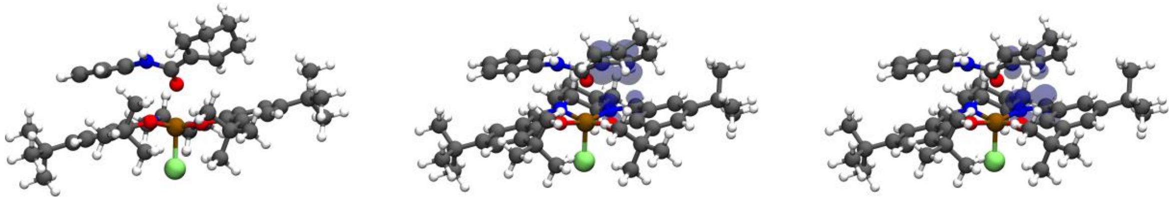

Figure 24: Molecular structure at the minimum energy crossing point (**CompMECP**; left) and the spin densities in the T_1 (middle) and T_2 (right) states at the point. Note that T_1 and T_2 are almost degenerate at this structure with an energy difference of about 0.01 eV.

Table 19: Al–O distances at different triplet state structures. The oxygen considered is the ketone oxygen of the substrate.

	IM1	CompT	CompMECP
Al–O distance / Å	1.96	2.02	2.22

The MECP structure (**CompMECP**) was optimized to an energy difference of 0.01 eV between the involved states (T_1 and T_2). Evidently, very little structural change is necessary to change the electronic structure so that the catalyst- and substrate-localized states cross and the substrate-localized state becomes lower in energy. The structural change can mainly be seen in the aluminium oxgen distance which increases (see SI Table 18). Additionally, a twisting in the substrate can be identified which is needed for the reaction. This twisting motion is accompanied by a deformation of the catalyst (see SI Fig. 23).

Optical Rotations

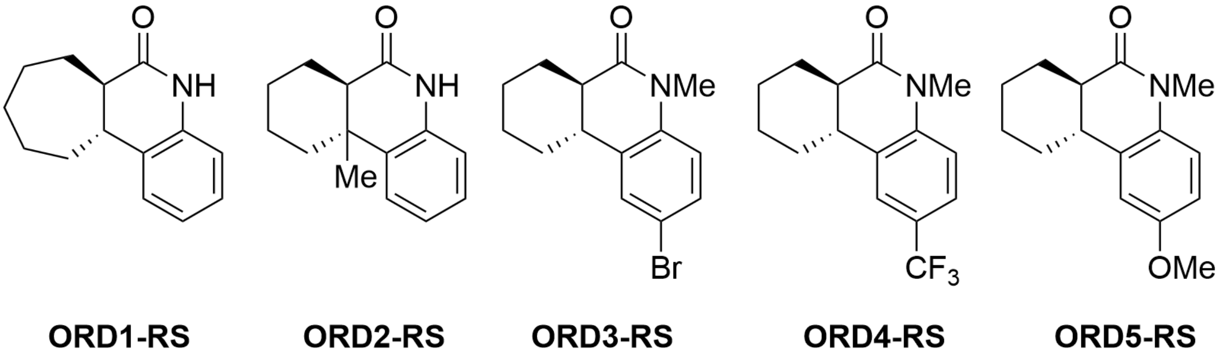

Figure 25: Molecules for which the specific optical rotations have been calculated. Shown is the *R,S* conformation.

Table 20: Optical rotation of different conformers of **ORD1** as found by conformational search. The results were obtained from TDDFT calculations using the CAM-B3LYP functional and def2-TZVP basis set.

Conformer	[α_{TDDFT}] / degrees
**ORD1-RR-1**	71.32
**ORD1-RR-2**	82.51
**ORD1-RR-3**	47.92
**ORD1-RR-4**	57.85
**ORD1-RR-5**	−114.98
**ORD1-RR-6**	−46.06
**ORD1-RR-7**	−77.81
**ORD1-RR-8**	−77.14
**ORD1-RR-9**	114.48
**ORD1-SS-1**	−77.28
**ORD1-SS-2**	−81.96
**ORD1-SS-3**	−48.67
**ORD1-SS-4**	−58.60
**ORD1-SS-5**	121.11
**ORD1-SS-6**	44.86
**ORD1-SS-7**	83.10
**ORD1-SS-8**	77.65
**ORD1-SS-9**	−115.02
**ORD1-SS-10**	153.33
**ORD1-RS-1**	234.39
**ORD1-RS-2**	72.79
**ORD1-RS-3**	24.47
**ORD1-RS-4**	−148.35
**ORD1-RS-5**	−157.69
**ORD1-SR-1**	−233.33
**ORD1-SR-2**	−300.40
**ORD1-SR-3**	−285.73
**ORD1-SR-4**	−72.73
**ORD1-SR-5**	150.26
**ORD1-SR-6**	−14.49
**ORD1-SR-7**	157.46

Table 21: Free energies and Boltzmann weights of the conformers of **ORD1**.

Conformer	$\Delta G_{\mathrm{solv}}^{294}$ / kcal/mol	Boltzmann weight / %
**ORD1-RR-1**	0.00	21.73
**ORD1-RR-2**	0.01	21.51
**ORD1-RR-3**	0.05	20.27
**ORD1-RR-4**	0.32	12.59
**ORD1-RR-5**	0.52	8.91
**ORD1-RR-6**	0.82	5.30
**ORD1-RR-7**	0.99	4.02
**ORD1-RR-8**	1.12	3.18
**ORD1-RR-9**	1.27	2.49
**ORD1-SS-1**	0.00	21.05
**ORD1-SS-2**	0.02	20.18
**ORD1-SS-3**	0.04	19.50
**ORD1-SS-4**	0.25	13.79
**ORD1-SS-5**	0.48	9.32
**ORD1-SS-6**	0.81	5.30
**ORD1-SS-7**	0.92	4.36
**ORD1-SS-8**	1.14	3.00
**ORD1-SS-9**	1.26	2.42
**ORD1-SS-10**	1.73	1.08
**ORD1-RS-1**	0.00	95.75
**ORD1-RS-2**	2.04	2.92
**ORD1-RS-3**	3.09	0.49
**ORD1-RS-4**	3.14	0.44
**ORD1-RS-5**	3.20	0.40
**ORD1-SR-1**	0.00	61.42
**ORD1-SR-2**	0.67	19.37
**ORD1-SR-3**	0.76	16.84
**ORD1-SR-4**	2.15	1.56
**ORD1-SR-5**	3.14	0.29
**ORD1-SR-6**	3.16	0.28
**ORD1-SR-7**	3.21	0.25

Table 22: Optical rotation of different conformers of **ORD2** as found by conformational search. The results were obtained from TDDFT calculations using the CAM-B3LYP functional and def2-TZVP basis set.

Conformer	$[\alpha_{\text{TDDFT}}]$ / degrees
**ORD2-RR-1**	14.61
**ORD2-RR-2**	6.12
**ORD2-SS-1**	−13.83
**ORD2-SS-2**	−7.55
**ORD2-RS-1**	77.48
**ORD2-RS-2**	73.58
**ORD2-SR-1**	−77.27

Table 23: Free energies and Boltzmann weights of the conformers of **ORD2**.

Conformer	$\Delta G_{\text{solv}}^{294}$ / kcal/mol	Boltzmann weight / %
**ORD2-RR-1**	0.0	99.42
**ORD2-RR-2**	3.00	0.58
**ORD2-SS-1**	0.00	99.50
**ORD2-SS-2**	3.09	0.50
**ORD2-RS-1**	0.0	51.40
**ORD2-RS-2**	0.03	48.60
**ORD2-SR-1**	0.00	100.00

Table 24: Optical rotation of different conformers of **ORD3** as found by conformational search. The results were obtained from TDDFT calculations using the CAM-B3LYP functional and def2-TZVP basis set.

Conformer	$[\alpha_{\text{TDDFT}}]$ / degrees
**ORD3-RR-1**	164.14
**ORD3-RR-2**	−57.18
**ORD3-SS-1**	−164.53
**ORD3-SS-2**	57.11
**ORD3-RS-1**	178.43
**ORD3-SR-1**	−178.06

Table 25: Free energies and Boltzmann weights of the conformers of **ORD3**.

Conformer	$\Delta G_{\mathrm{solv}}^{294}$ / kcal/mol	Boltzmann weight / %
**ORD3-RR-1**	0.00	79.77
**ORD3-RR-2**	0.80	20.23
**ORD3-SS-1**	0.00	80.90
**ORD3-SS-2**	0.84	19.10
**ORD3-RS-1**	0.00	100.00
**ORD3-SR-1**	0.00	100.00

Table 26: Optical rotation of different conformers for **ORD4** as found by conformational search. The results were obtained from TDDFT calculations using the CAM-B3LYP functional and def2-TZVP basis set.

Conformer	[α_{TDDFT}] / degrees
**ORD4-RR-1**	149.80
**ORD4-RR2**	−115.74
**ORD4-SS-1**	−148.58
**ORD4-SS-2**	111.99
**ORD4-RS-1**	209.66
**ORD4-RS-2**	222.05
**ORD4-SR-1**	−210.78
**ORD4-SR-2**	−217.33

Table 27: Free energies and Boltzmann weights of the conformers of **ORD4**.

Conformer	$\Delta G_{\mathrm{solv}}^{294}$/ kcal/mol	Boltzmann weight / %
**ORD4-RR-1**	0.00	88.04
**ORD4-RR-2**	1.17	11.96
**ORD4-SS-1**	0.00	85.20
**ORD4-SS-2**	1.02	14.80
**ORD4-RS-1**	0.00	56.69
**ORD4-RS-2**	0.16	43.31
**ORD4-SR-1**	0.00	57.39
**ORD4-SR-2**	0.17	42.61

Table 28: Optical rotation of different conformers of **ORD5** as found by conformational search. The results were obtained from TDDFT calculations using the CAM-B3LYP functional and def2-TZVP basis set.

Conformer	[α_{TDDFT}] / degrees
**ORD5-RR-1**	160.07
**ORD5-RR-2**	171.49
**ORD5-RR-3**	−119.62
**ORD5-RR-4**	−28.10
**ORD5-SS-1**	−161.04
**ORD5-SS-2**	−171.67
**ORD5-SS-3**	120.70
**ORD5-SS-4**	25.40
**ORD5-RS-1**	221.82
**ORD5-RS-2**	158.72
**ORD5-SR-1**	−219.35
**ORD5-SR-2**	−163.57

Table 29: Free energies and Boltzmann weights of the conformers of **ORD5**.

Conformer	$\Delta G_{\text{solv}}^{294}$/ kcal/mol	Boltzmann weight
**ORD5-RR-1**	0.00	44.18
**ORD5-RR-2**	0.05	40.58
**ORD5-RR-3**	0.85	10.26
**ORD5-RR-4**	1.28	4.98
**ORD5-SS-1**	0.00	43.45
**ORD5-SS-2**	0.03	41.61
**ORD5-SS-3**	0.86	10.03
**ORD5-SS-4**	1.27	4.91
**ORD5-RS-1**	0.00	64.90
**ORD5-RS-2**	0.36	35.10
**ORD5-SR-1**	0.00	69.67
**ORD5-SR-2**	0.49	30.33

Table 30: Boltzmann weighted overall optical rotations of the *R*,*R*, *S*,*S*, *R*,*S*, and *S*,*R* conformations.

Compound	[α_{TDDFT}] / degrees
**ORD1-RR**	34.84
**ORD1-SS**	−30.55
**ORD1-RS**	225.39
**ORD1-SR**	−245.40
**ORD2-RR**	14.56
**ORD2-SS**	−13.80
**ORD2-RS**	75.58
**ORD2-SR**	−77.27
**ORD3-RR**	119.37
**ORD3-SS**	−122.19
**ORD3-RS**	178.43
**ORD3-SR**	−178.06
**ORD4-RR**	118.03
**ORD4-SS**	−110.01
**ORD4-RS**	215.02
**ORD4-SR**	−213.57
**ORD5-RR**	126.64
**ORD5-SS**	−128.06
**ORD5-RS**	199.67
**ORD5-SR**	−202.43

The optical rotation of the pure compound [α_{pure}] has been calculated as

$$[\alpha_{\mathrm{pure}}] = \frac{[\alpha_{\mathrm{observed}}]\cdot 100}{ee} \qquad \text{(Eq. 1)}$$

with the enantiomeric excess *e.e.* given in percent.

Table 31: Measured optical rotations and calculated theoretical pure value.

Compound	[$\alpha_{observed}$] degrees	*e.r.*	[α_{pure}] degrees
**ORD1** *cis*	−12.6	76:24	−24.2
**ORD1** *trans*	126.0	85:15	180.0
**ORD2** *trans*	69.7	84:16	102.5
**ORD3** *trans*	96.5	84:16	141.9
**ORD4** *trans*	94.9	79:21	163.6
**ORD5** *trans*	110.0	96:4	119.6

Table 32: Differences of the calculated and measured optical rotations. The measured cis values are compared to the *R,R* and *S,S* values. The measured trans values are compared to the *R,S* and *S,R* values.

Compound	$\Delta\alpha^{TDDFT}$ / degrees
**ORD1-RR**	59.0
**ORD1-SS**	−6.4
**ORD1-RS**	45.39
**ORD1-SR**	−425.4
**ORD2-RS**	−26.9
**ORD2-SR**	−179.8
**ORD3-RS**	36.5
**ORD3-SR**	−320.0
**ORD4-RS**	51.4
**ORD4-SR**	−377.2
**ORD5-RS**	80.1
**ORD5-SR**	−322.0

These findings suggest that the measured *cis*-conformation corresponds to the *S,S* conformation and the measured *trans*-conformations correspond to the *R,S* conformation.

Single Crystal X-ray Diffractometry

X-ray diffraction analysis and data collection were performed using Cu-Kα or Mo-Kα radiation on a Bruker Venture D8 instrument equipped with a microsource and a Photon III CMOS detector. Initial structure solutions were obtained using the SHELXT[65] package via direct methods and refined with SHELXL[66,67] against all $|F^2|$ using initially isotropic and subsequently anisotropic thermal parameters. Anisotropic refinement was applied to all non-hydrogen atoms, while hydrogen atoms were positioned at ideal coordinates based on calculated positions. The graphical representations of the molecular structures were generated with Diamond 4.6.1 (Crystal Impact GbR, Bonn, Germany).

Table 33: X-ray crystal structure analysis of ***rac*-2j-*trans***.

CCDC deposition number	2368376	
Empirical formula	C11 H11 N O	
Formula weight (g mol^{-1})	173.21	
Temperature	120(2) K	
Radiation Type	Mo-Kα	
Wavelength	0.71073 Å	
Crystal system	Triclinic	
Space group	P –1	
Unit cell dimensions	a = 5.7297(5) Å	α = 82.813(2)°.
	b = 8.3873(7) Å	β = 86.393(2)°.
	c = 9.1498(7) Å	γ = 80.960(2)°.
Volume	430.42(6) Å^3	
Z	2	
D_{calc} (g cm^{-3})	1.336	
μ (mm^{-1})	0.086	
F(000)	184	
Crystal dimensions (mm)	0.350 x 0.310 x 0.070	
Θ limit (°)	2.246 – 25.697°	
Index ranges (h,k,l)	-6:6, –10:10, –11:11	
Reflections collected	7913	
Independent reflections	1622 [R_{int} = 0.0225]	
Completeness to theta = 25.242°	99.0 %	
Absorption correction	Semi-empirical from equivalents	

Max. and min. transmission	0.7454 and 0.6540
Refinement method	Full-matrix least-squares on F^2
Data / restraints / parameters	1622 / 64 / 155
Goodness-of-fit on F^2	1.277
Final R indices [I>2σ(I)]	$R1$ = 0.0899, w$R2$ = 0.1929
R indices (all data)	$R1$ = 0.0911, w$R2$ = 0.1933
Min./max. res. dens e (Å^{-3})	0.306 / –0.568

The four-membered ring was found disordered over two positions with a ratio of 0.65:0.35.

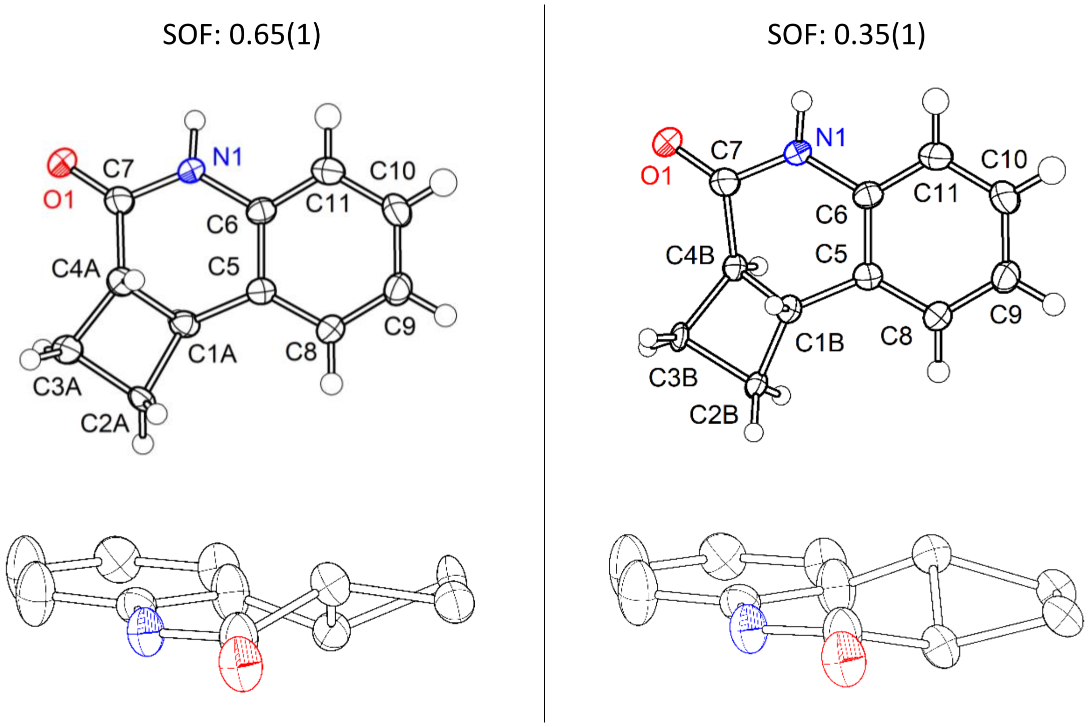

Figure 26: Crystal Structure of compound ***rac*-2j-*trans***. For each disorder two different orientations are depicted. Thermal ellipsoids (except H) are shown at 50% probability.

Table 34**: X-ray crystal structure analysis of **2j-*cis.

CCDC deposition number	2368374	
Empirical formula	C11 H11 N O	
Formula weight (g mol^{-1})	173.21	
Temperature	120(2) K	
Wavelength	1.54178 Å	
Radiation Type	Cu-Kα	
Crystal system	Monoclinic	
Space group	P 21	
Unit cell dimensions	a = 6.4392(2) Å	α = 90°.
	b = 7.5168(2) Å	β = 109.6380(10)°.
	c = 9.3141(3) Å	γ = 90°.
Volume	424.60(2) Å^3	
Z	2	
D_{calc} (g cm^{-3})	1.355	
μ (mm^{-1})	0.694	
F(000)	184	
Crystal size	0.300 x 0.100 x 0.050	
Θ limit (°)	5.042 – 79.326°.	
Index ranges (h,k,l)	-7:7, –8:9, –11:11	
Reflections collected	7818	
Independent reflections	1737 [R_{int} = 0.0263]	
Completeness to theta = 67.679°	100.0 %	
Absorption correction	Semi-empirical from equivalents	
Max. and min. transmission	0.7542 and 0.6478	
Refinement method	Full-matrix least-squares on F2	
Data / restraints / parameters	1737 / 1 / 118	
Goodness-of-fit on F2	1.049	
Final R indices [I>2σ(I)]	R1 = 0.0277, wR2 = 0.0724	
R indices (all data)	R1 = 0.0281, wR2 = 0.0730	
Absolute structure parameter	-0.01(9)	
Min./max. res. dens e (Å^{-3})	0.142 / –0.161 e.Å-3	

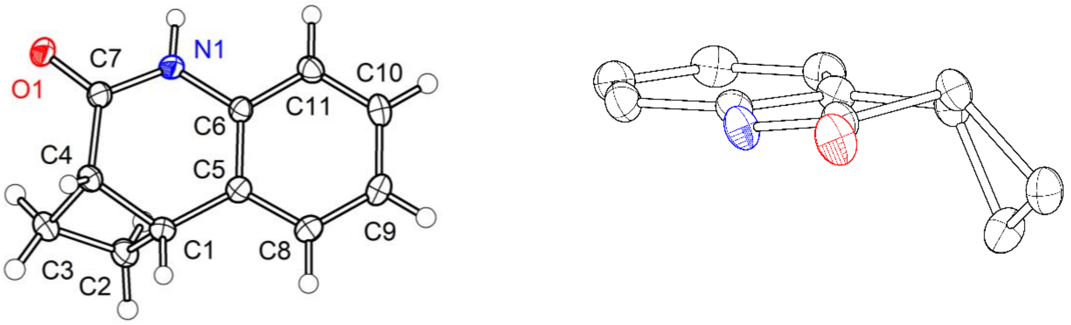

Figure 27: Crystal Structure of compound **2j**-***cis***. Two different orientations are depicted. Thermal ellipsoids (except H) are shown at 50% probability.

Table 35: X-ray crystal structure analysis of ***rac*-2n-*trans.***

CCDC deposition number	2368375	
Empirical formula	C14 H17 N O	
Formula weight	215.28	
Temperature	133(2) K	
Radiation Type	Cu-Kα	
Wavelength	1.54178 Å	
Crystal system	Monoclinic	
Space group	P 21	
Unit cell dimensions	a = 5.7220(2) Å	α = 90°.
	b = 18.9208(6) Å	β = 102.751(2)°.
	c = 10.5557(3) Å	γ = 90°.
Volume	1114.63(6) $Å^3$	
Z	4	
D_{calc} (g cm^{-3})	1.283	
μ (mm^{-1})	0.627	
F(000)	464	
Crystal dimensions (mm)	0.330 x 0.085 x 0.050	
Θ limit (°)	4.294 – 68.685°.	
Index ranges (h,k,l)	-6:6, –22:22, –12:12	
Reflections collected	36600	
Independent reflections	4018 [R_{int} = 0.0399]	
Completeness to theta = 67.679°	98.7 %	
Absorption correction	Semi-empirical from equivalents	
Max. and min. transmission	0.7531 and 0.6456	
Refinement method	Full-matrix least-squares on F2	
Data / restraints / parameters	4018 / 1 / 290	
Goodness-of-fit on F2	1.052	
Final R indices [I>2σ(I)]	R1 = 0.0537, wR2 = 0.1516	
R indices (all data)	R1 = 0.0551, wR2 = 0.1537	
Absolute structure parameter	0.5(4)	
Min./max. res. dens e ($Å^{-3}$)	0.750 / –0.243	

The structure was refined as inversion twin.

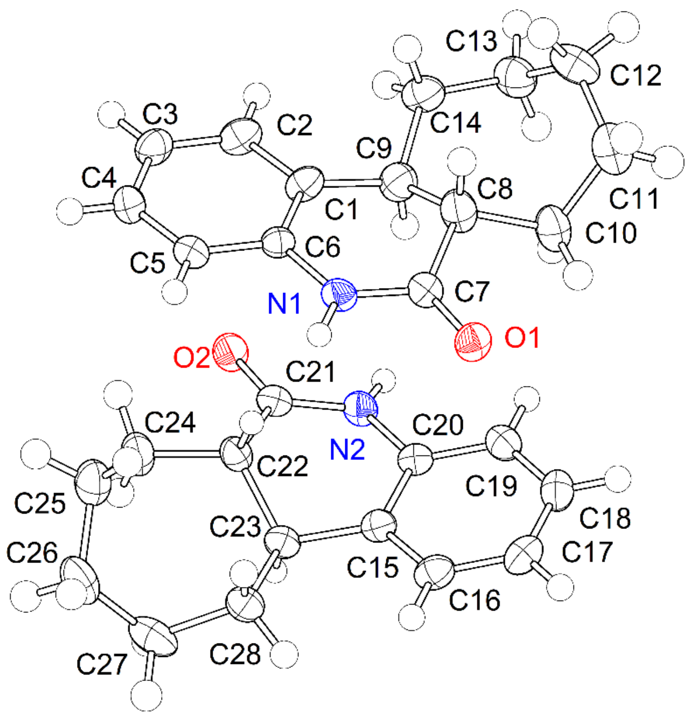

Figure 28: Crystal Structure of compound ***rac*-2n-*trans***. Thermal ellipsoids (except H) are shown at 50% probability.

Table 36: X-ray crystal structure analysis of-***rac*-2k-*trans*.**

CCDC deposition number	2368377	
Empirical formula	C14 H17 N O	
Formula weight	215.28	
Temperature	150(2) K	
Radiation Type	Cu-Kα	
Wavelength	1.54178 Å	
Crystal system	Monoclinic	
Space group	P 21/c	
Unit cell dimensions	a = 12.8668(3) Å	α = 90°.
	b = 13.7860(3) Å	β = 112.5220(10)°.
	c = 13.9230(3) Å	γ = 90°.
Volume	2281.33(9) $Å^3$	
Z	8	
D_{calc} (g cm^{-3})	1.254	
μ (mm^{-1})	0.613	
F(000)	928	
Crystal dimensions (mm)	0.170 x 0.110 x 0.070	
Θ limit (°)	3.719 – 78.687°.	
Index ranges (h,k,l)	-15:16, –17:17, –17:17	
Reflections collected	40747	
Independent reflections	4852 [R_{int} = 0.0313]	
Completeness to theta = 67.679°	99.8 %	
Absorption correction	Semi-empirical from equivalents	
Max. and min. transmission	0.7542 and 0.7016	
Refinement method	Full-matrix least-squares on F^2	
Data / restraints / parameters	4852 / 0 / 291	
Goodness-of-fit on F^2	1.020	
Final R indices [I>2σ(I)]	R1 = 0.0372, wR2 = 0.1015	
R indices (all data)	R1 = 0.0429, wR2 = 0.1091	
Min./max. res. dens e ($Å^{-3}$)	0.283 / –0.208	

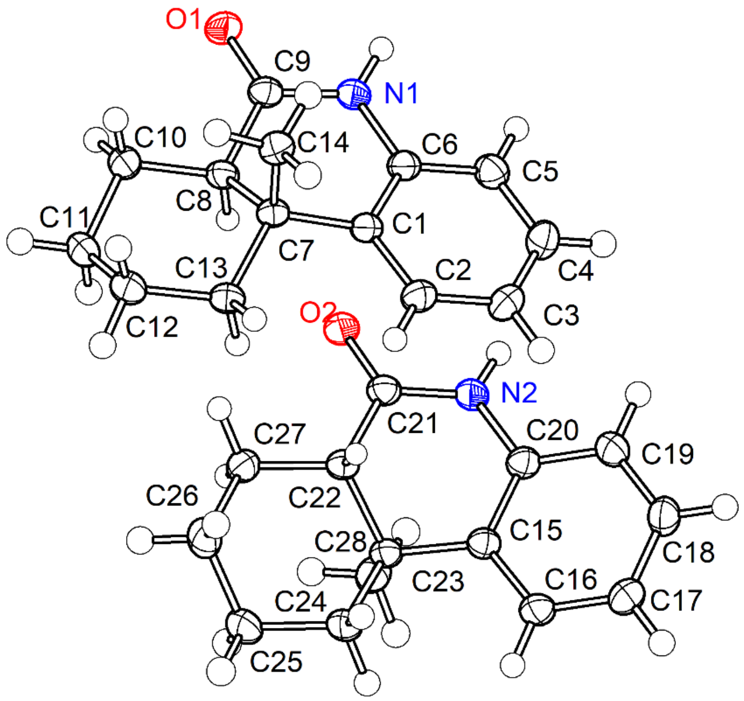

Figure 29: Crystal Structure of compound ***rac*-2k-*trans***. Thermal ellipsoids (except H) are shown at 50% probability.

NMR Spectra

^{1}H NMR (400 MHz, $CDCl_3$): **1a**

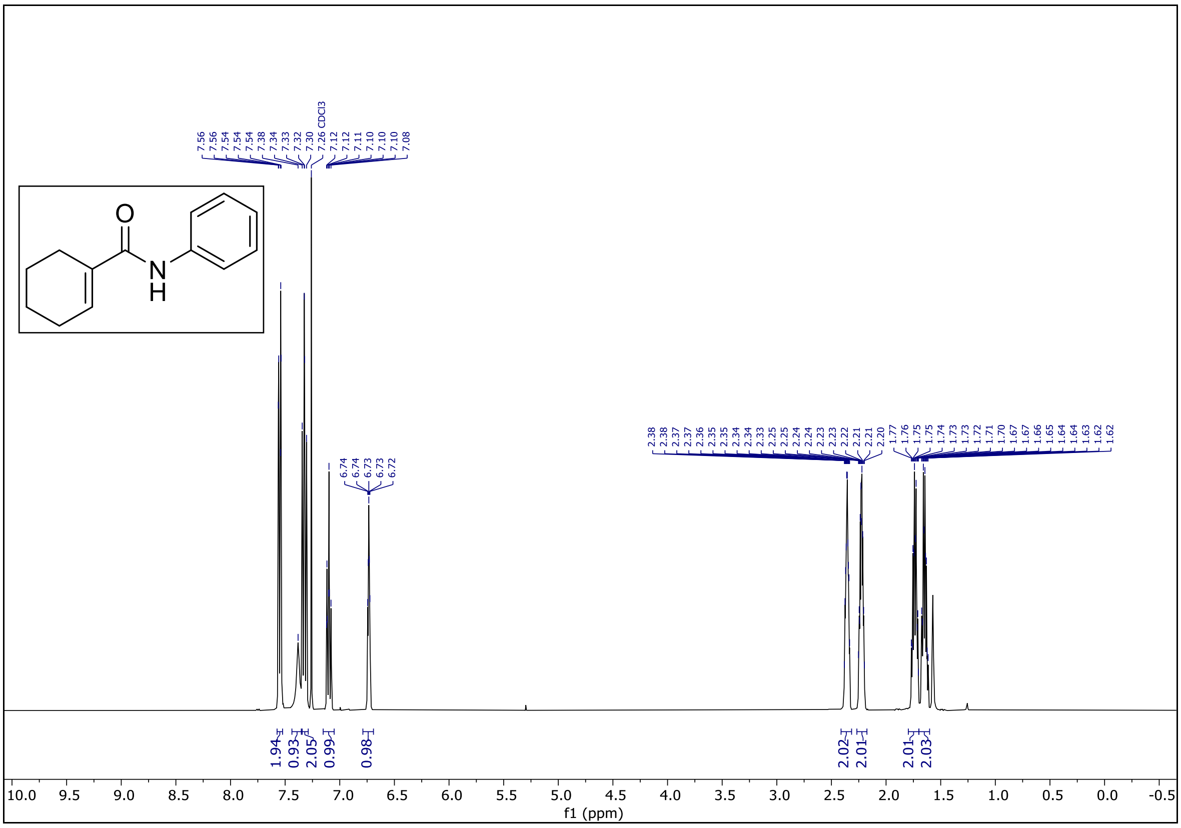

^{1}H NMR (400 MHz, $CDCl_3$): **1b**

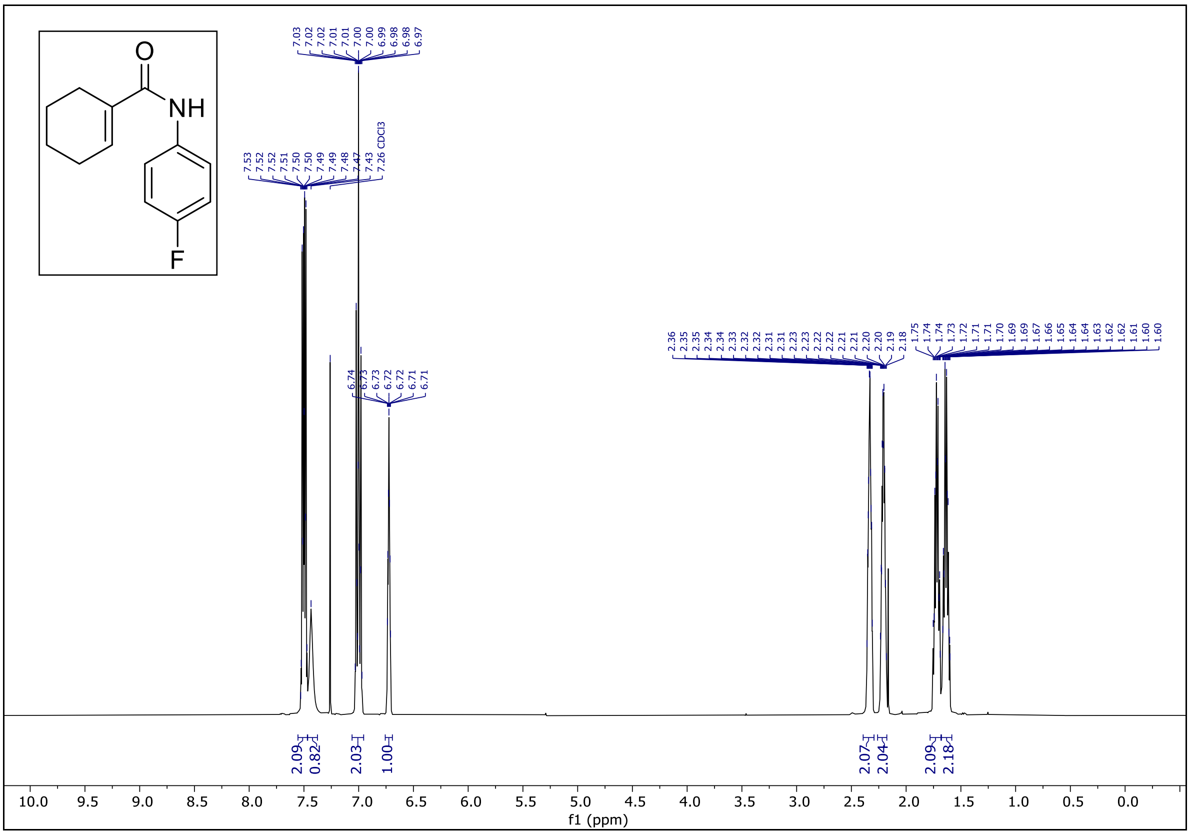

^{19}F NMR (377 MHz, $CDCl_3$): **1b**

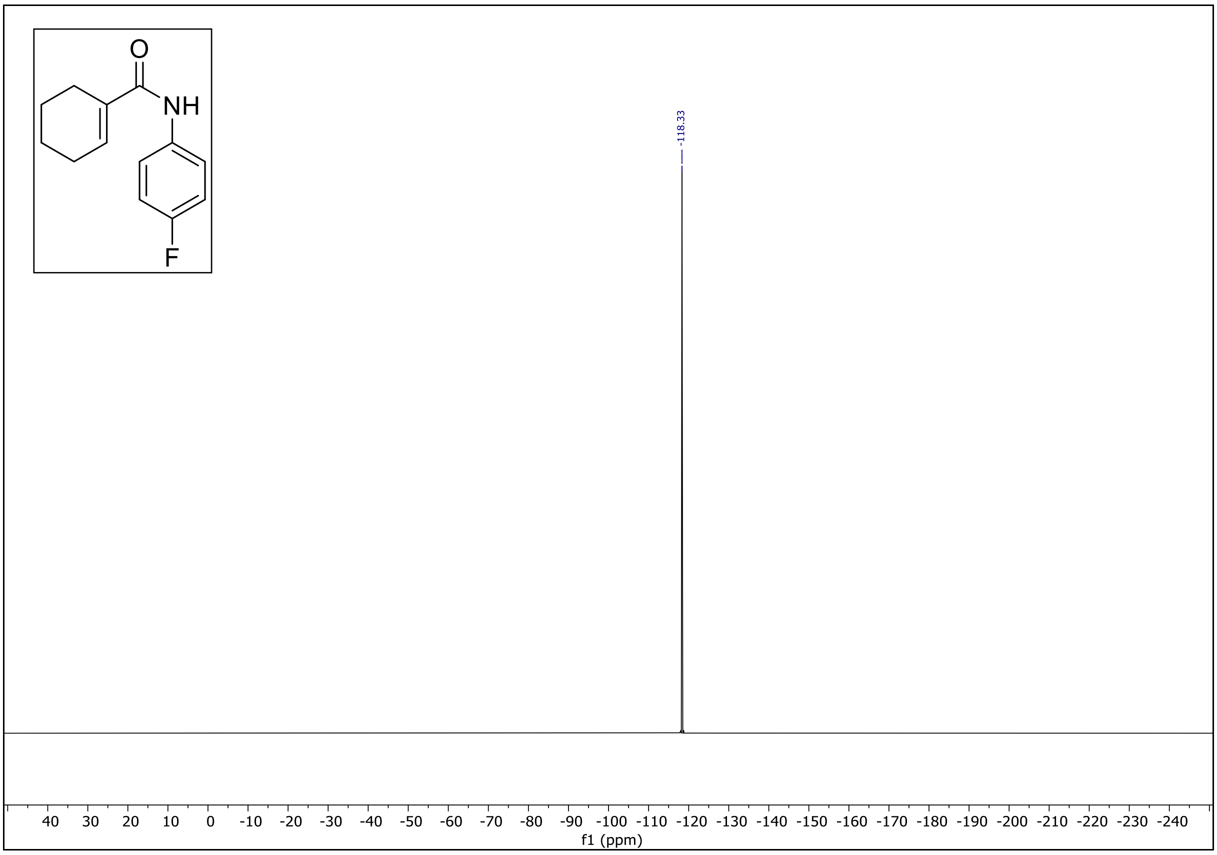

^{1}H NMR (400 MHz, $CDCl_3$): **1c**

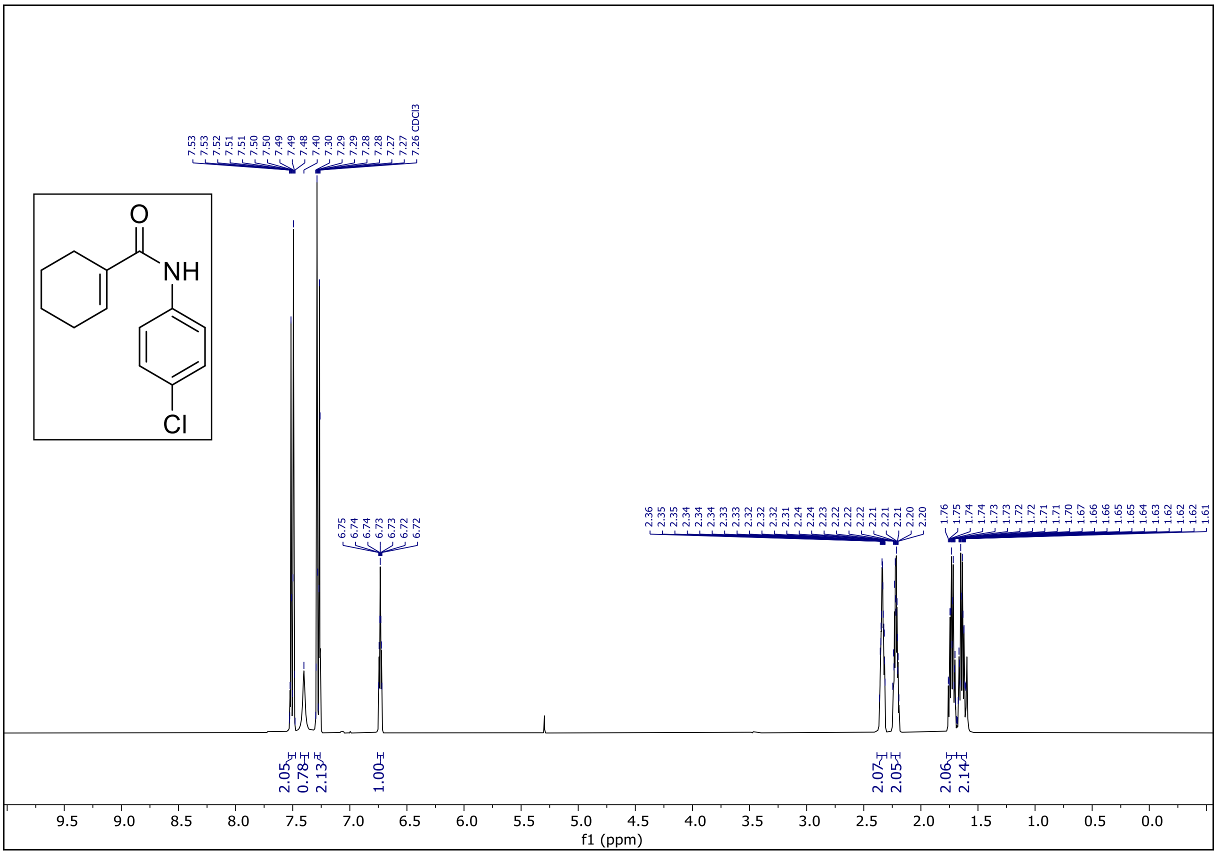

^{1}H NMR (500 MHz, $CDCl_3$): **1d**

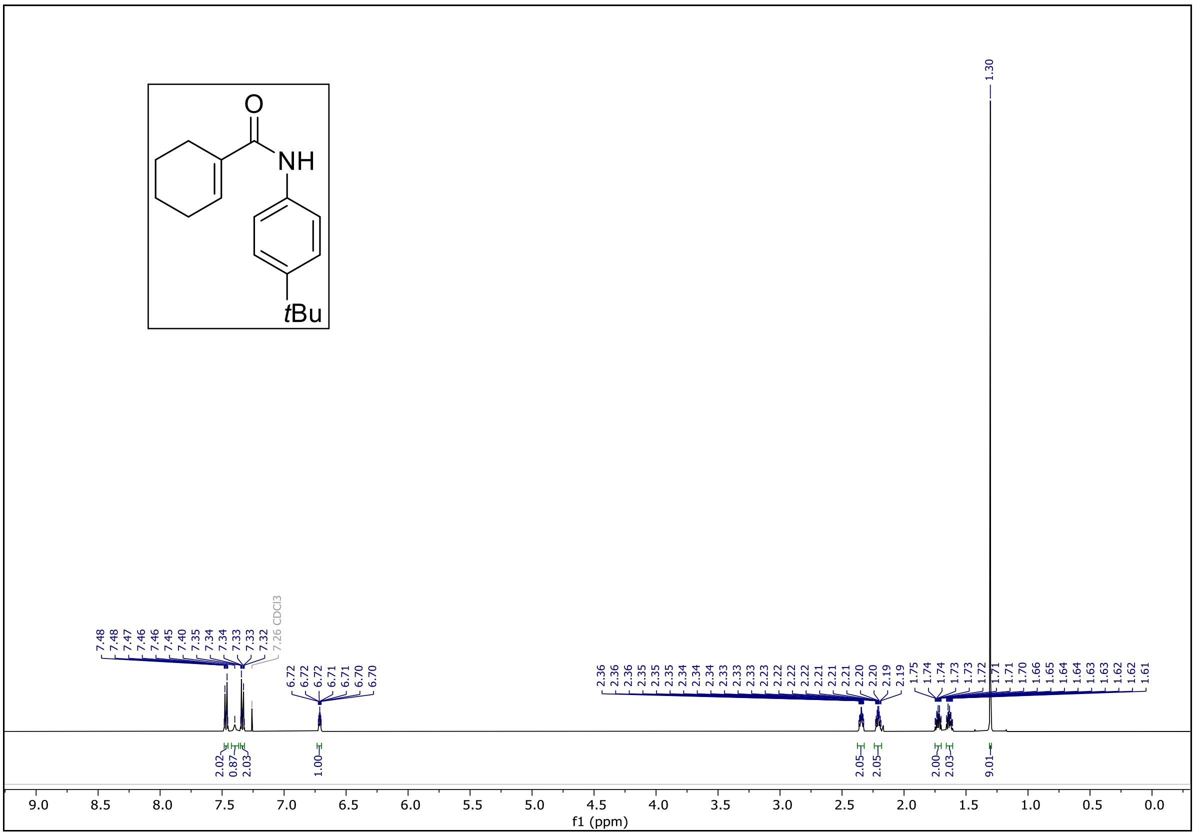

^{13}C NMR (126 MHz, $CDCl_3$): **1d**

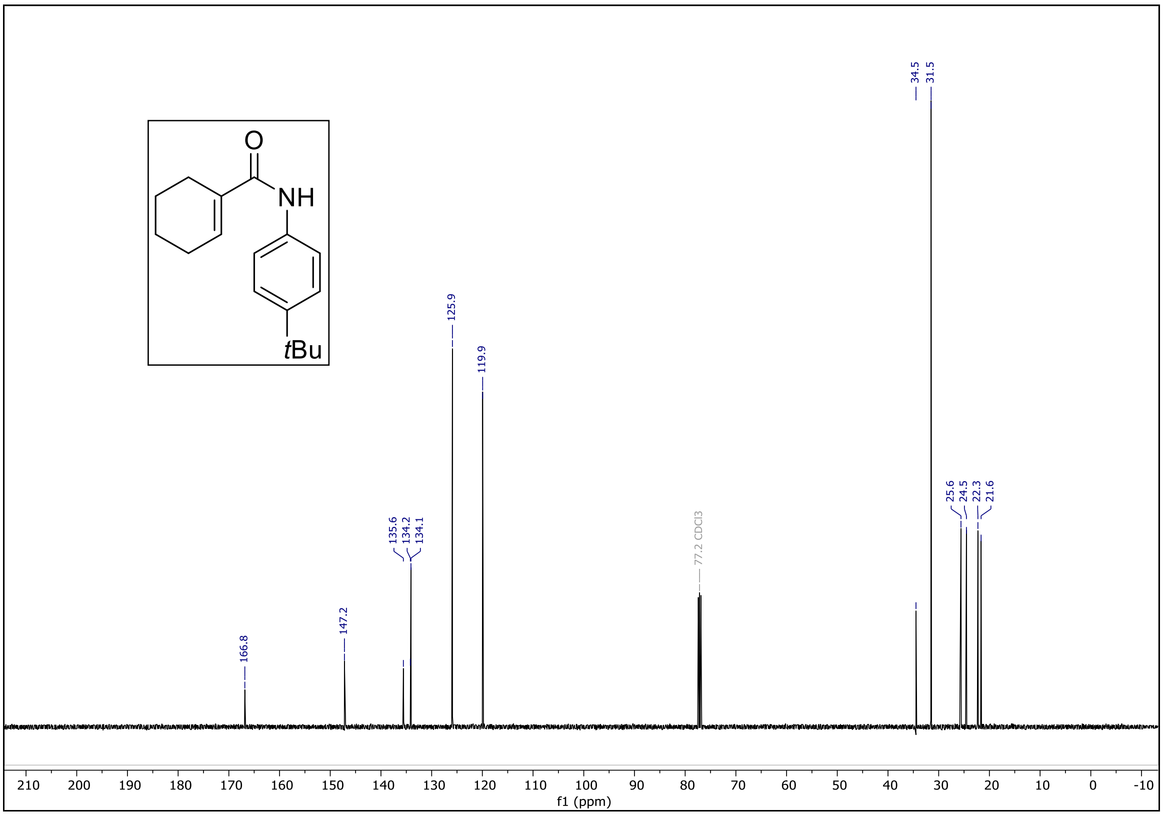

^{1}H NMR (500 MHz, $CDCl_3$): **1e**

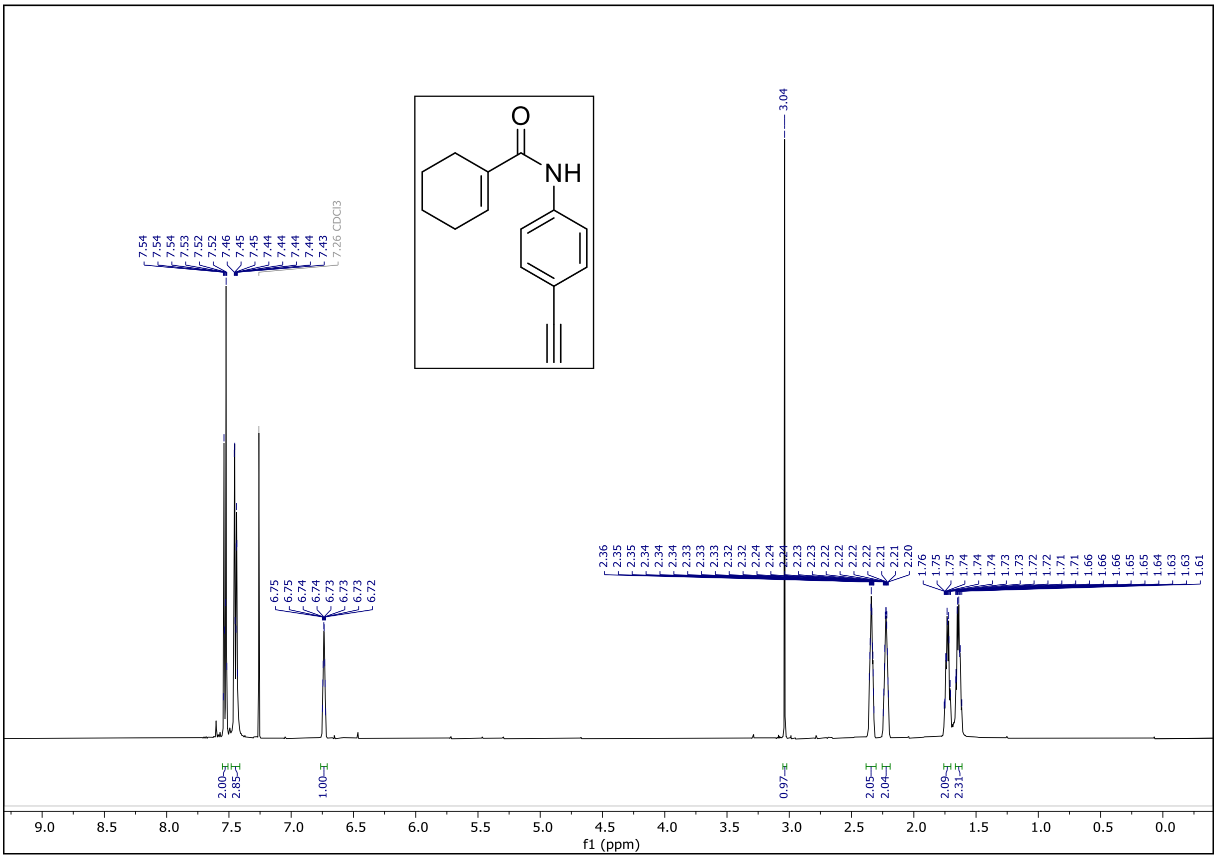

^{13}C NMR (126 MHz, CDCl$_3$): **1e**

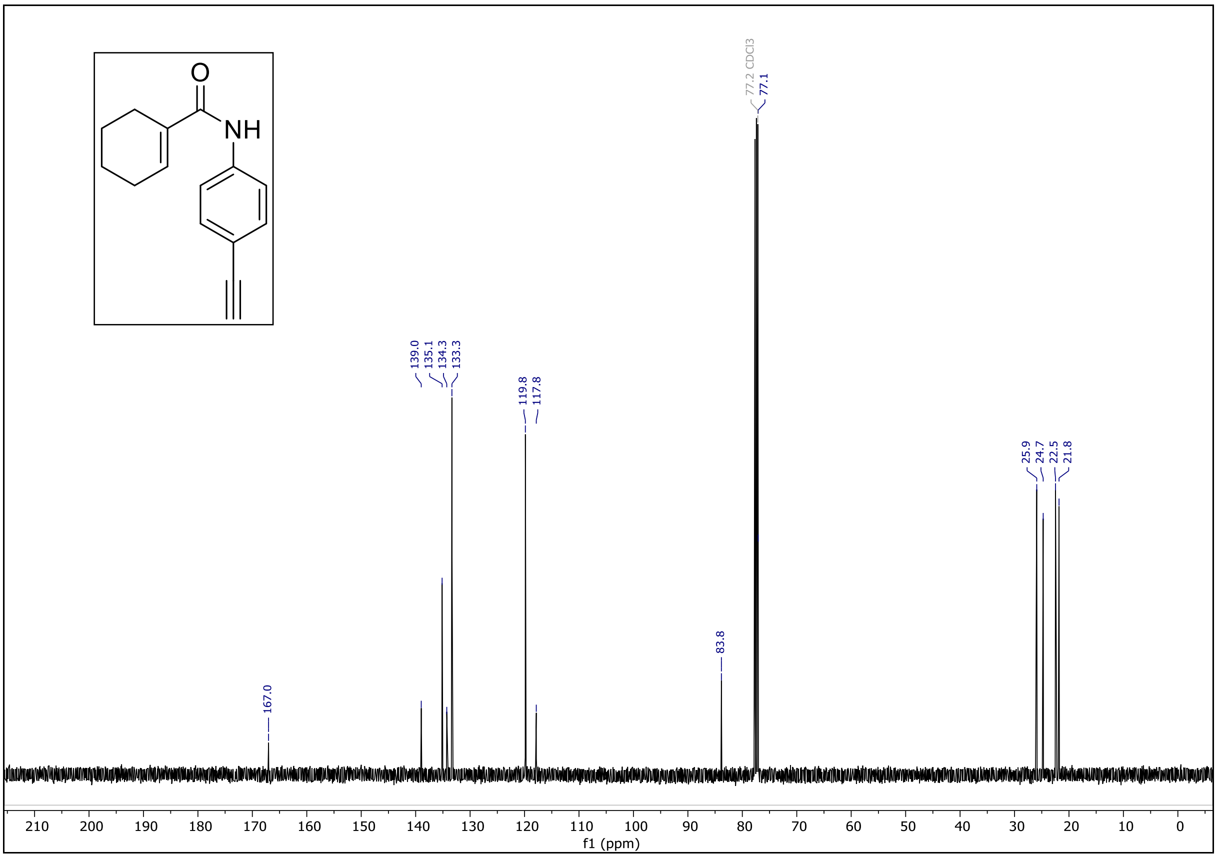

^{1}H NMR (400 MHz, $CDCl_3$): **S21**

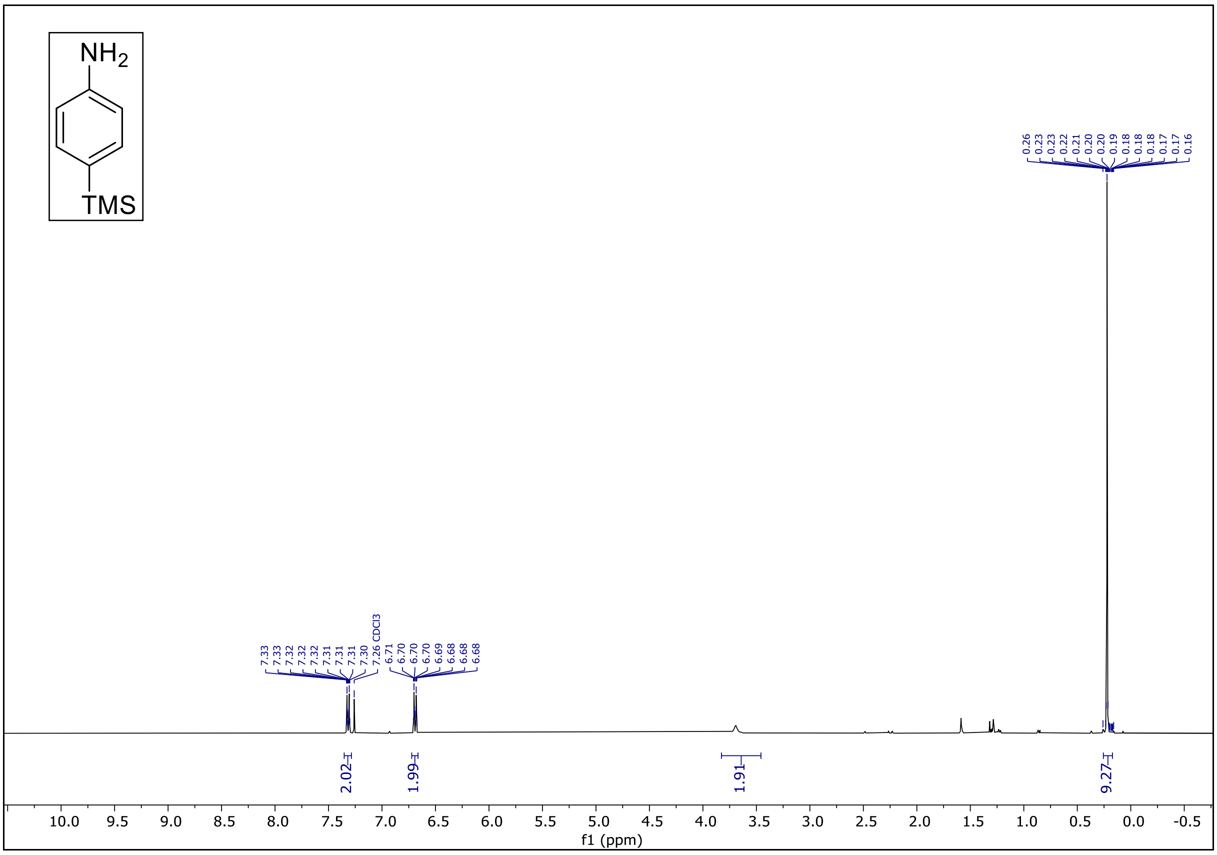

^{1}H NMR (500 MHz, $CDCl_3$): **1f**

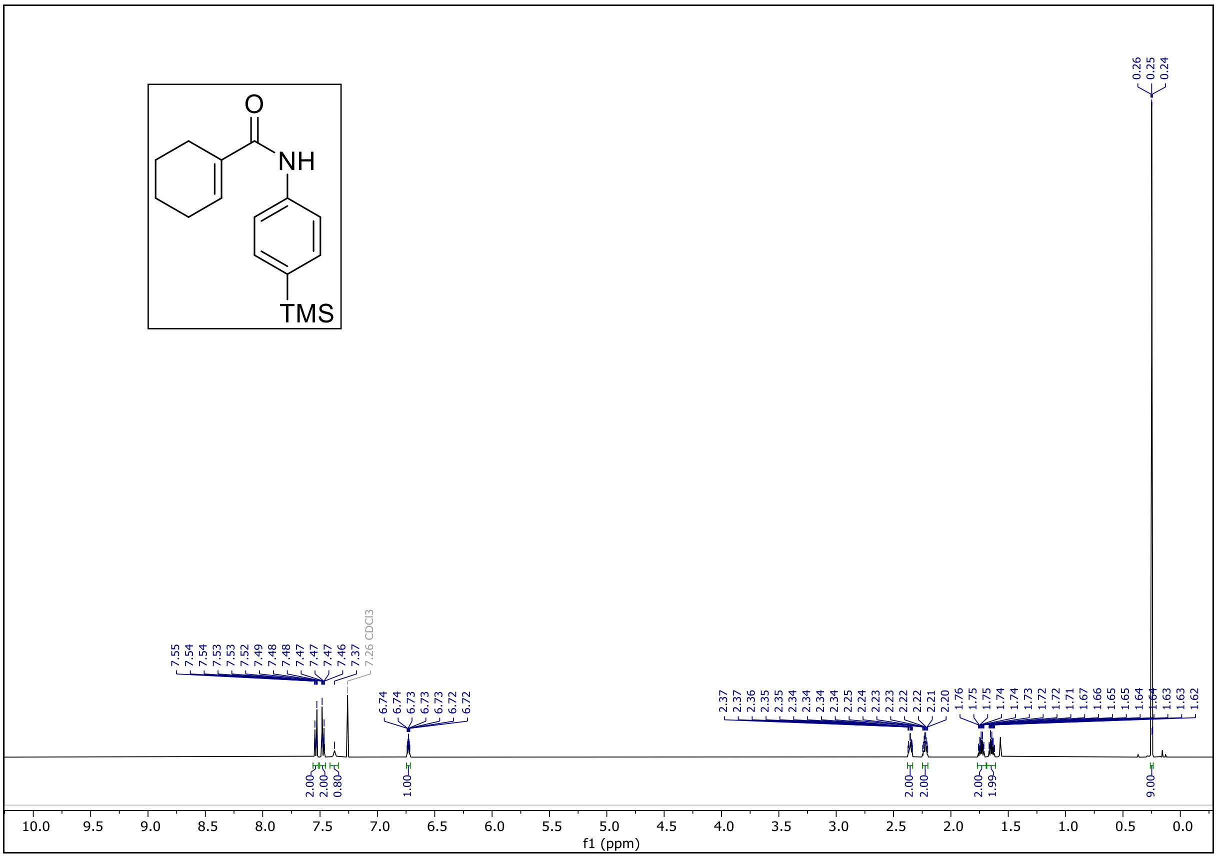

^{13}C NMR (126 MHz, $CDCl_3$): **1f**

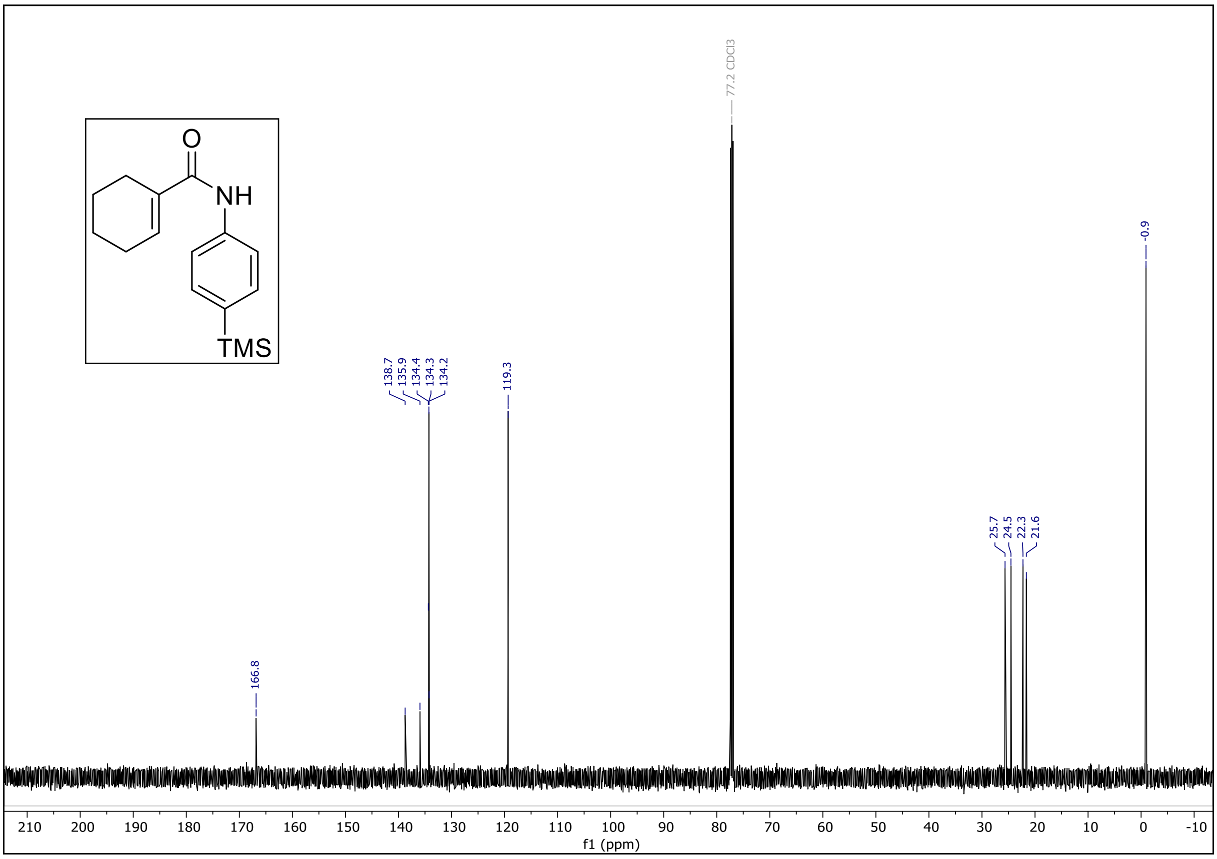

^{29}Si NMR (99 MHz, $CDCl_3$): **1f**

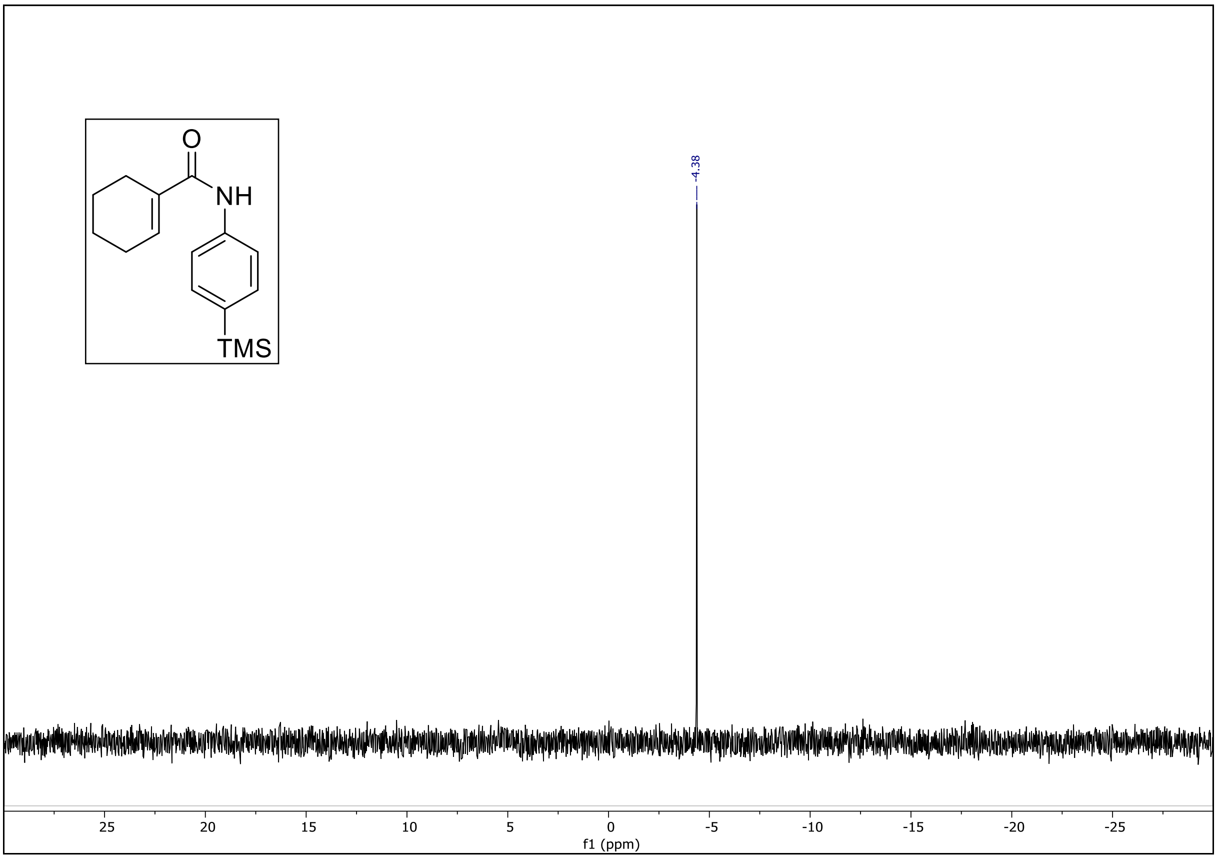

^{1}H NMR (500 MHz, $CDCl_3$): **S20**

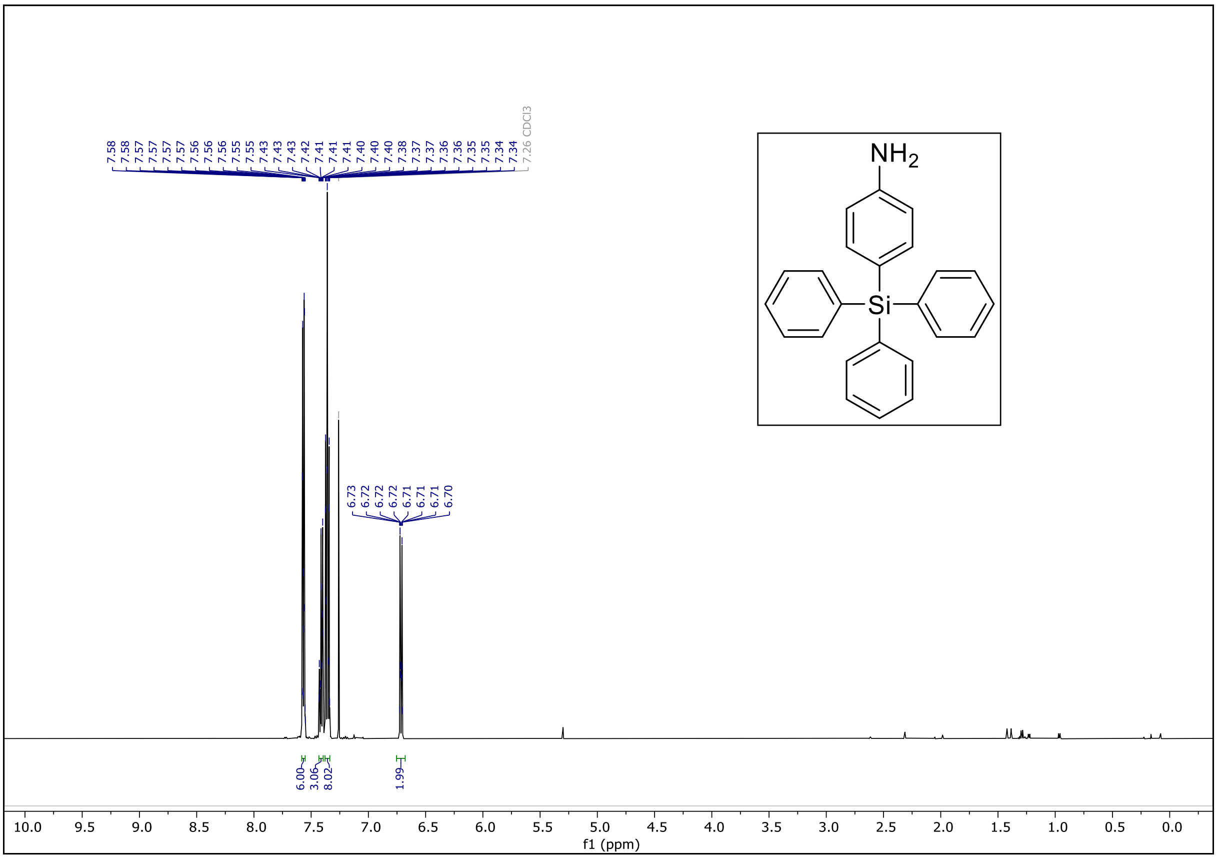

^{13}C NMR (126 MHz, $CDCl_3$): **S22**

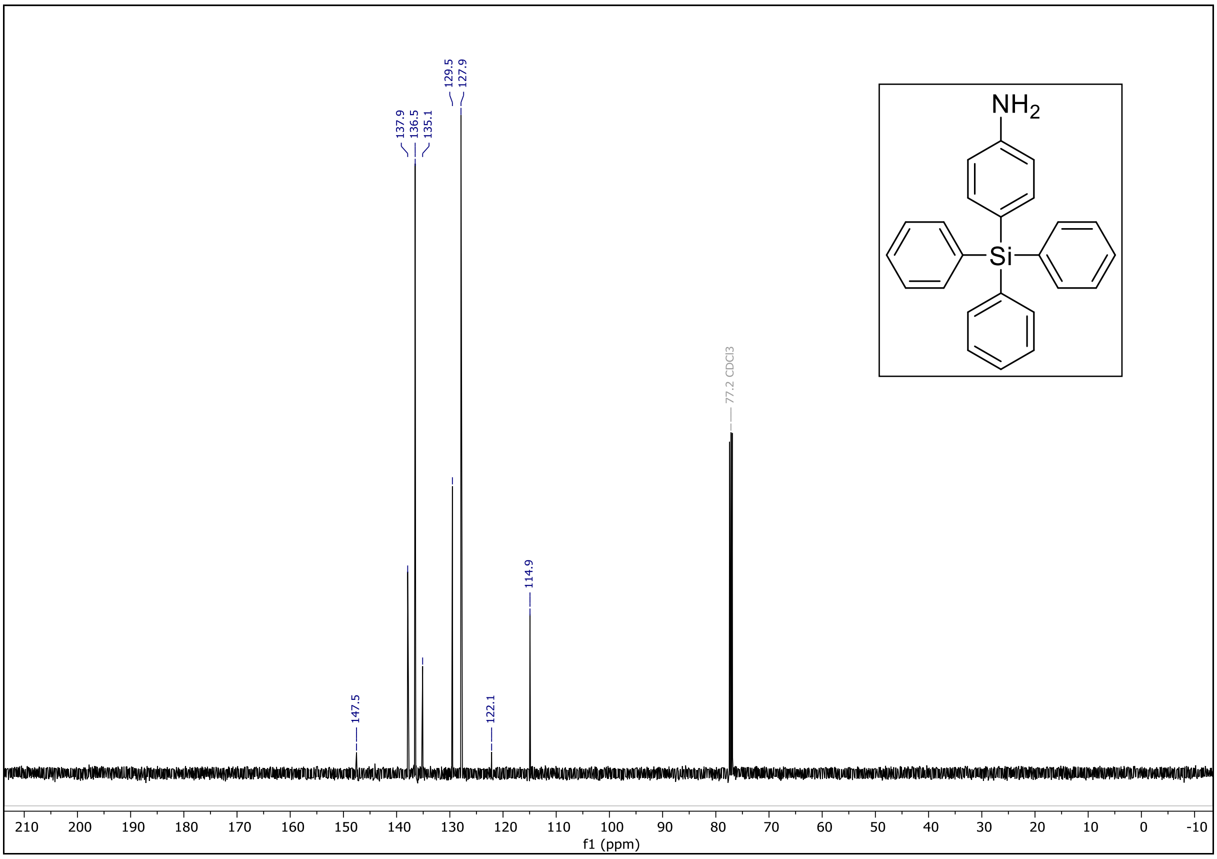

^{29}Si NMR (99 MHz, $CDCl_3$): **S22**

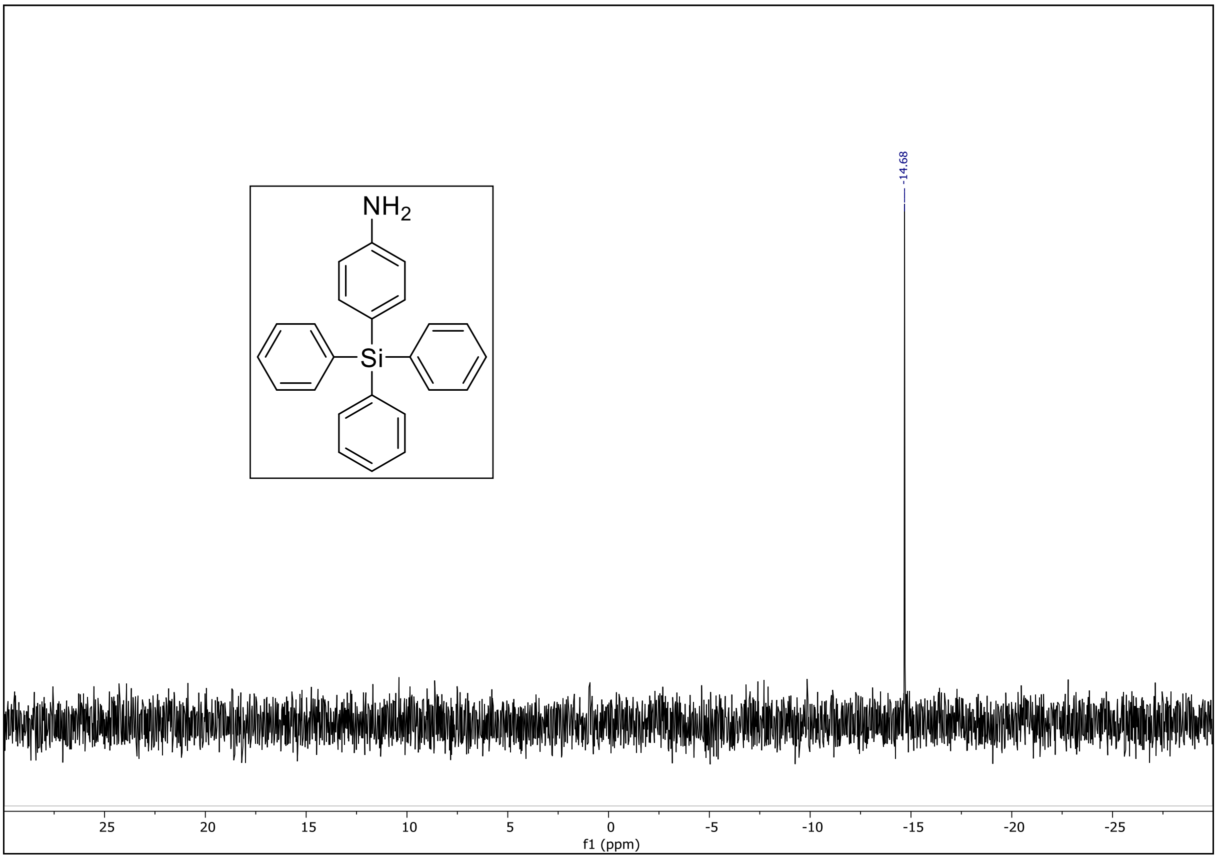

^{1}H NMR (500 MHz, DMSO-d_6): **1g**

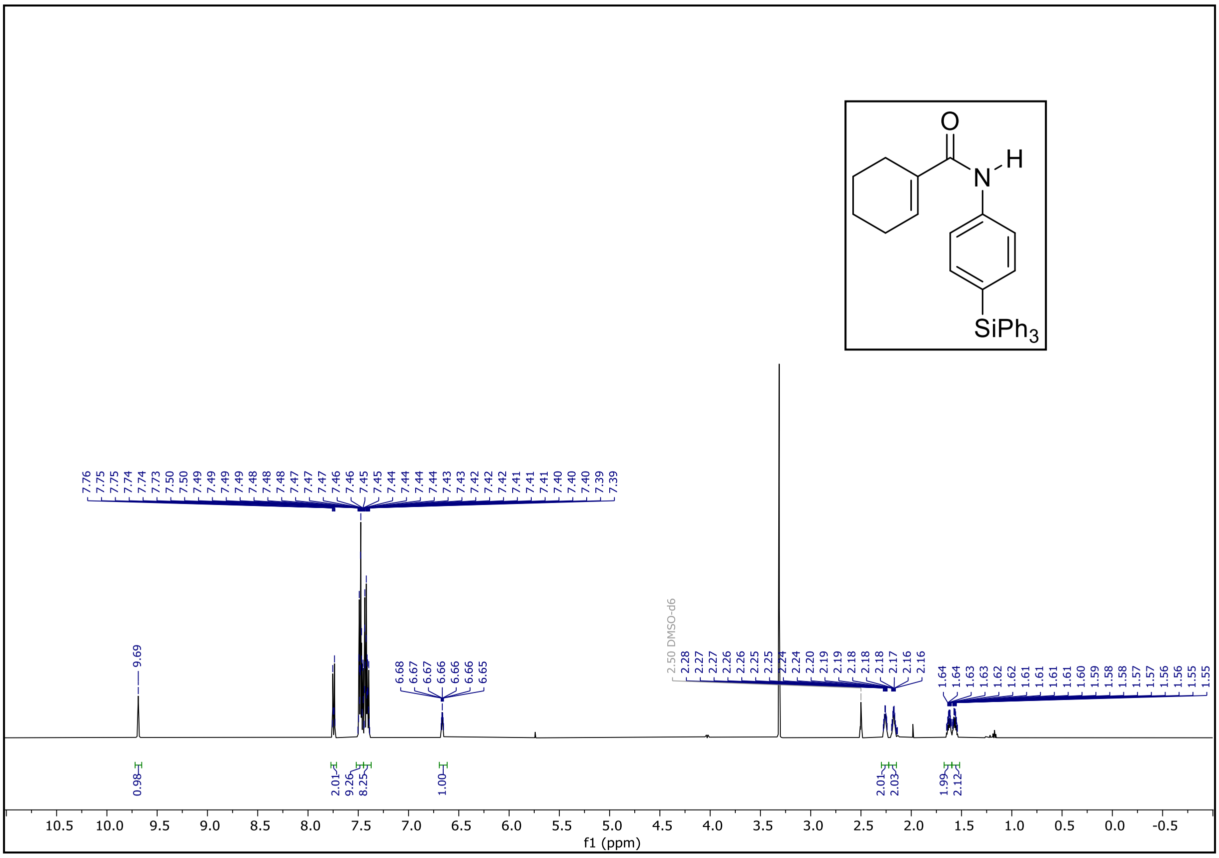

^{13}C NMR (126 MHz, DMSO-d_6): **1g**

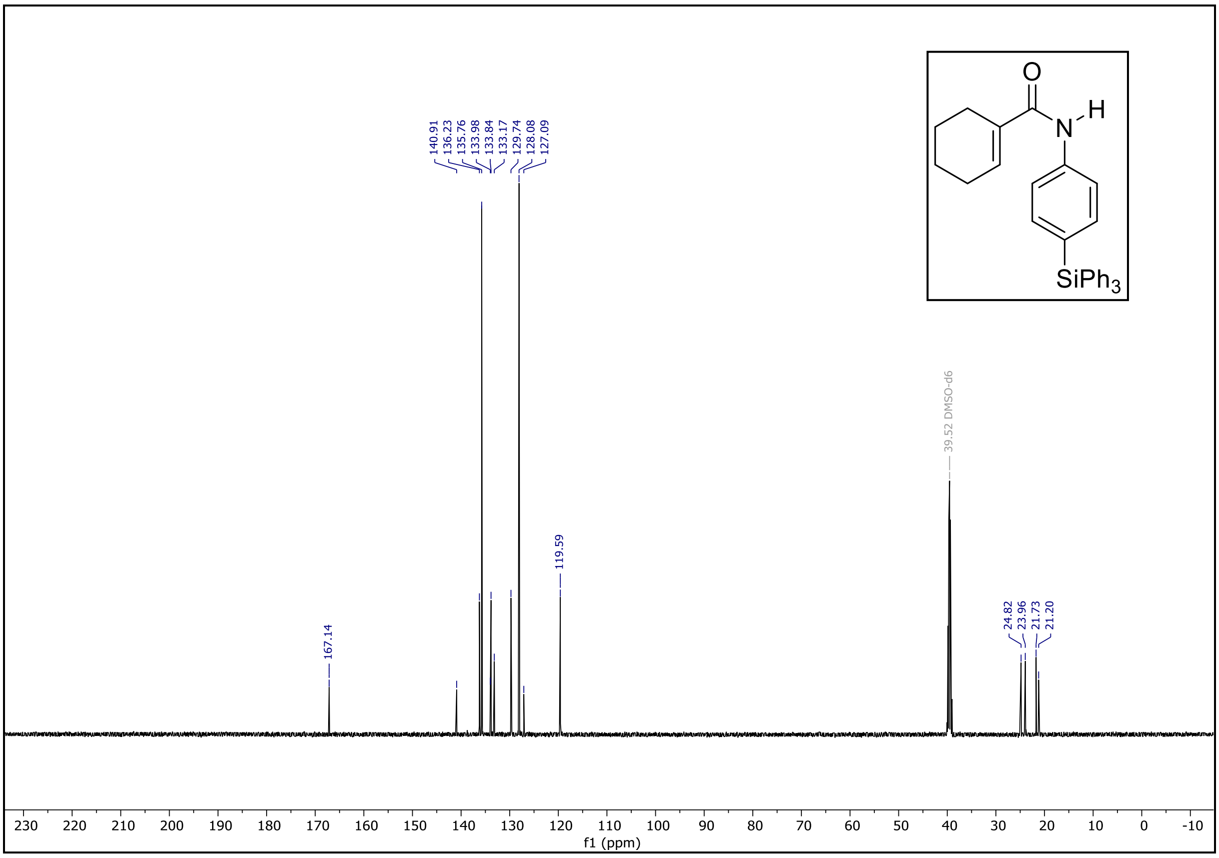

^{1}H NMR (500 MHz, $CDCl_3$): **1h**

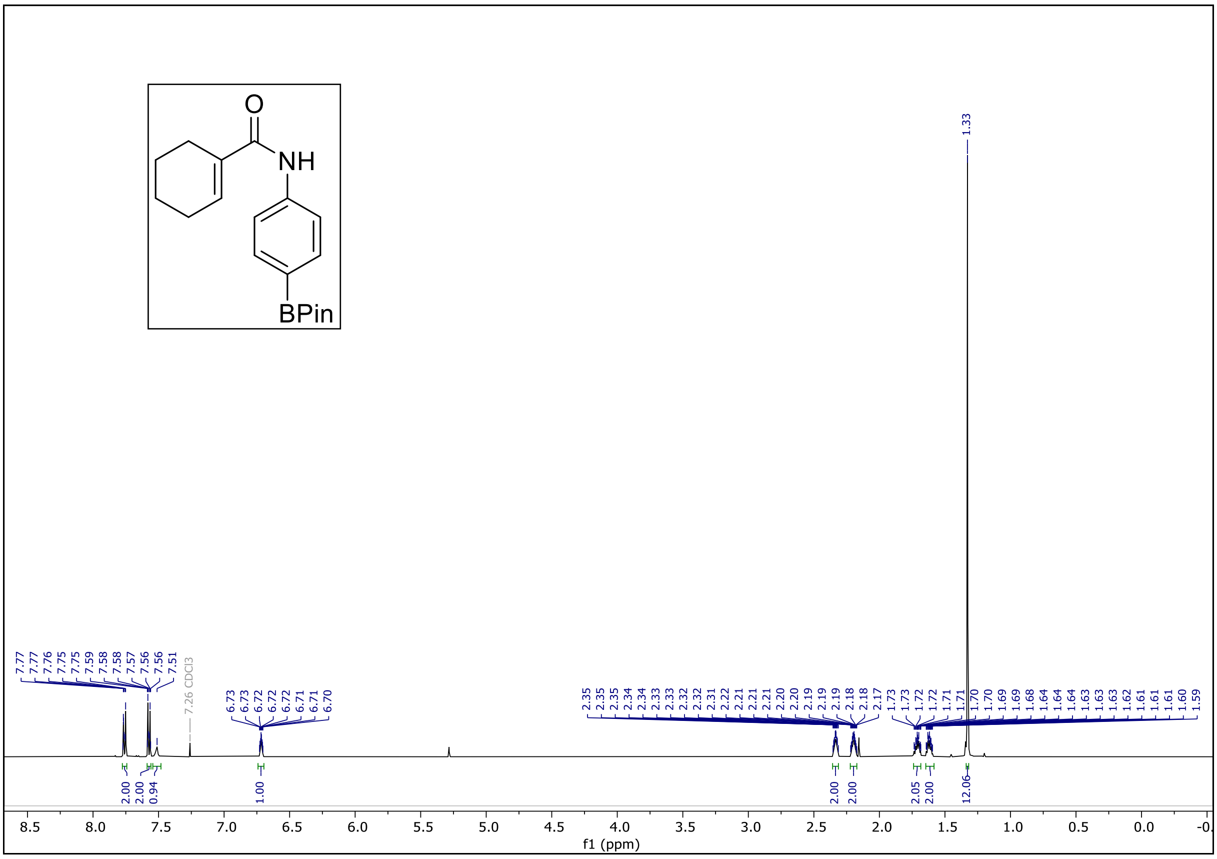

^{13}C NMR (126 MHz, $CDCl_3$): **1h**

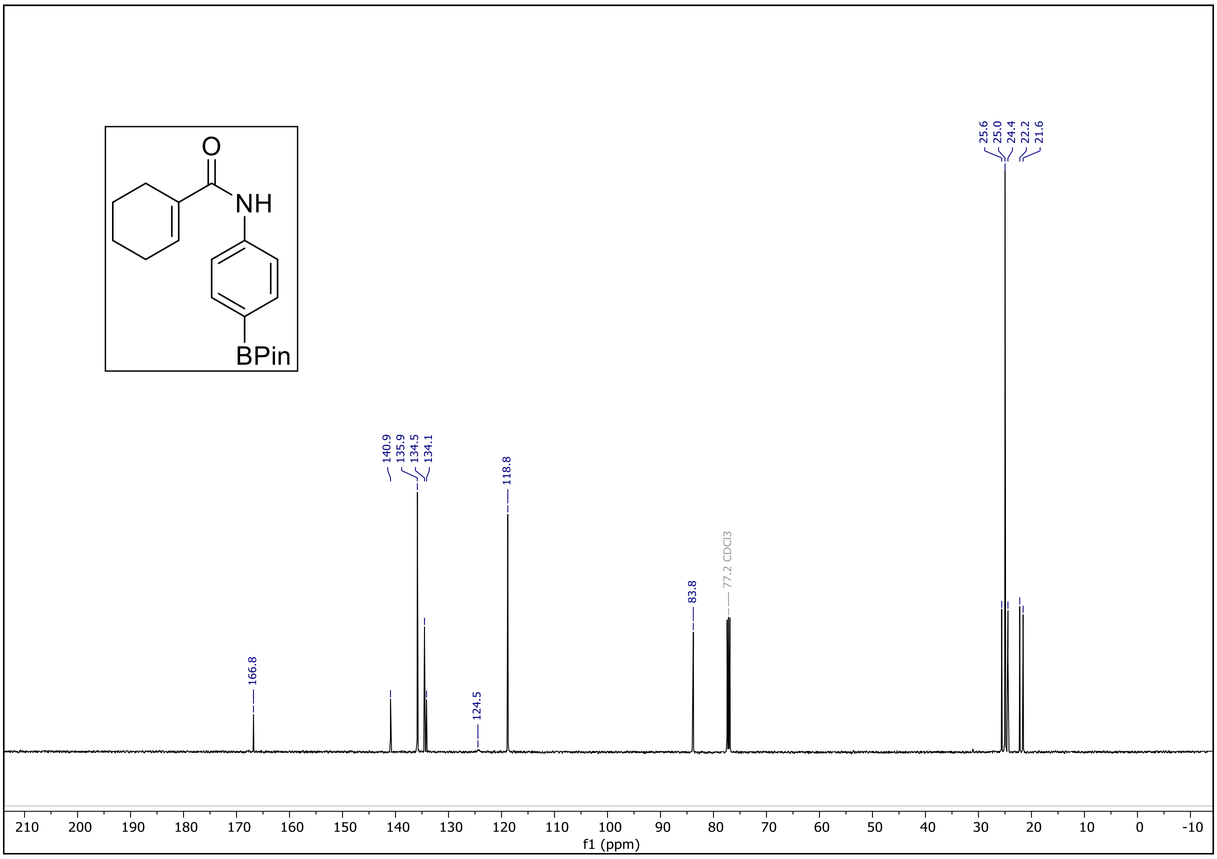

^{11}B NMR (160 MHz, $CDCl_3$): **1h**

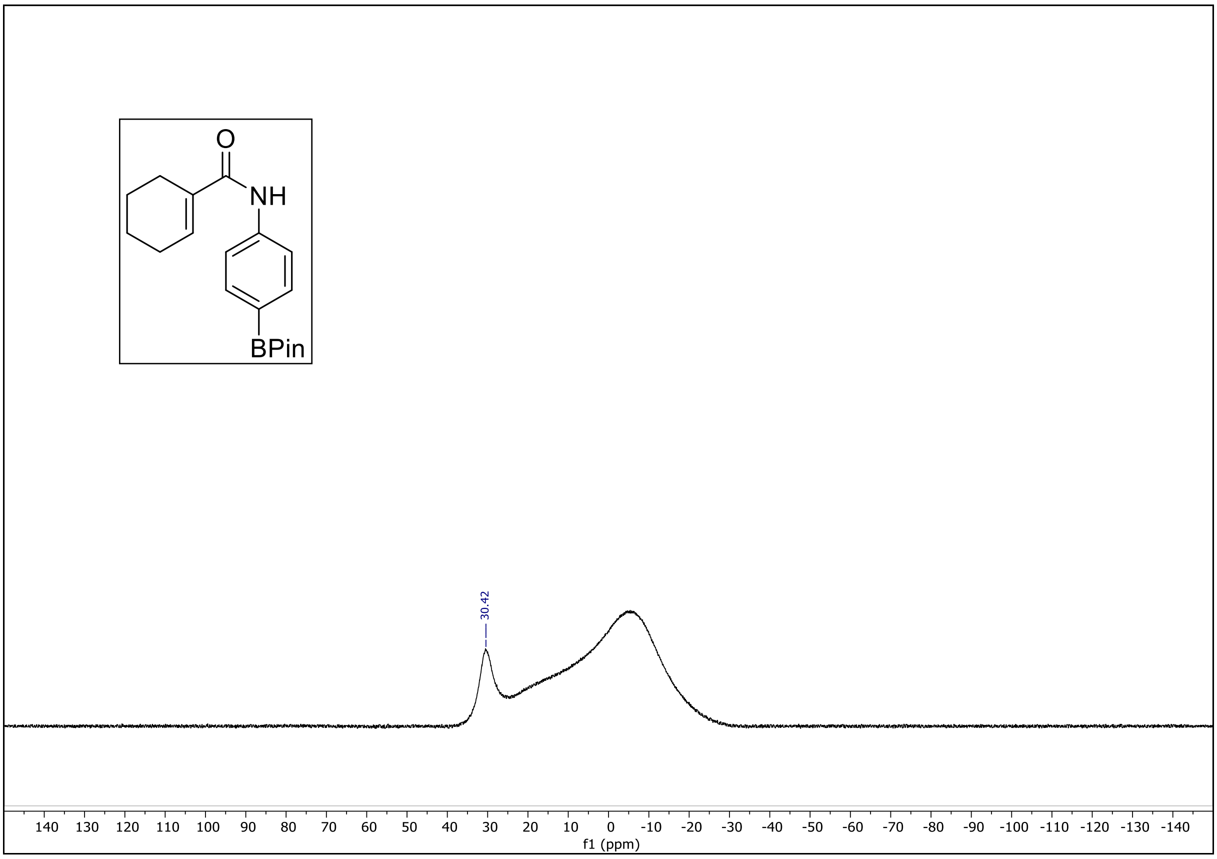

^1H NMR (400 MHz, $CDCl_3$): **1i**

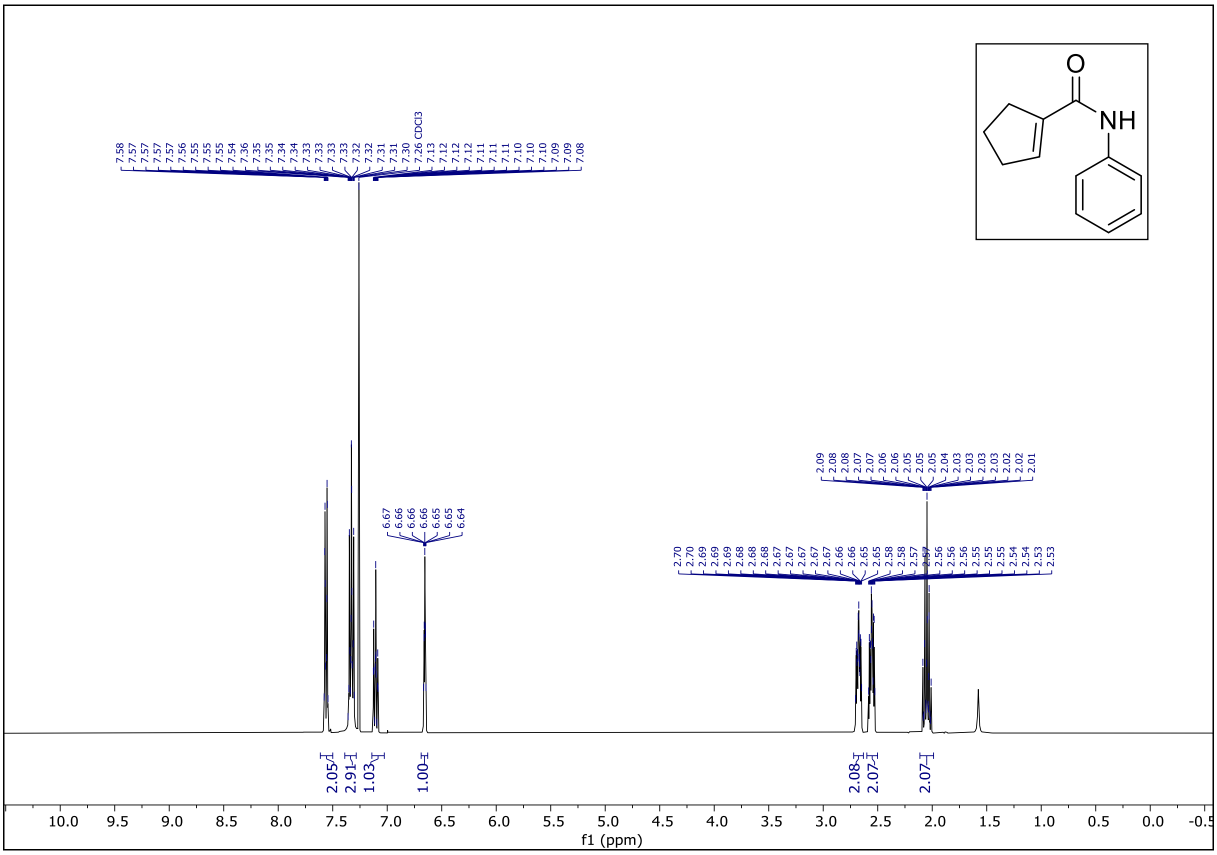

^1H NMR (400 MHz, $CDCl_3$): **1j**

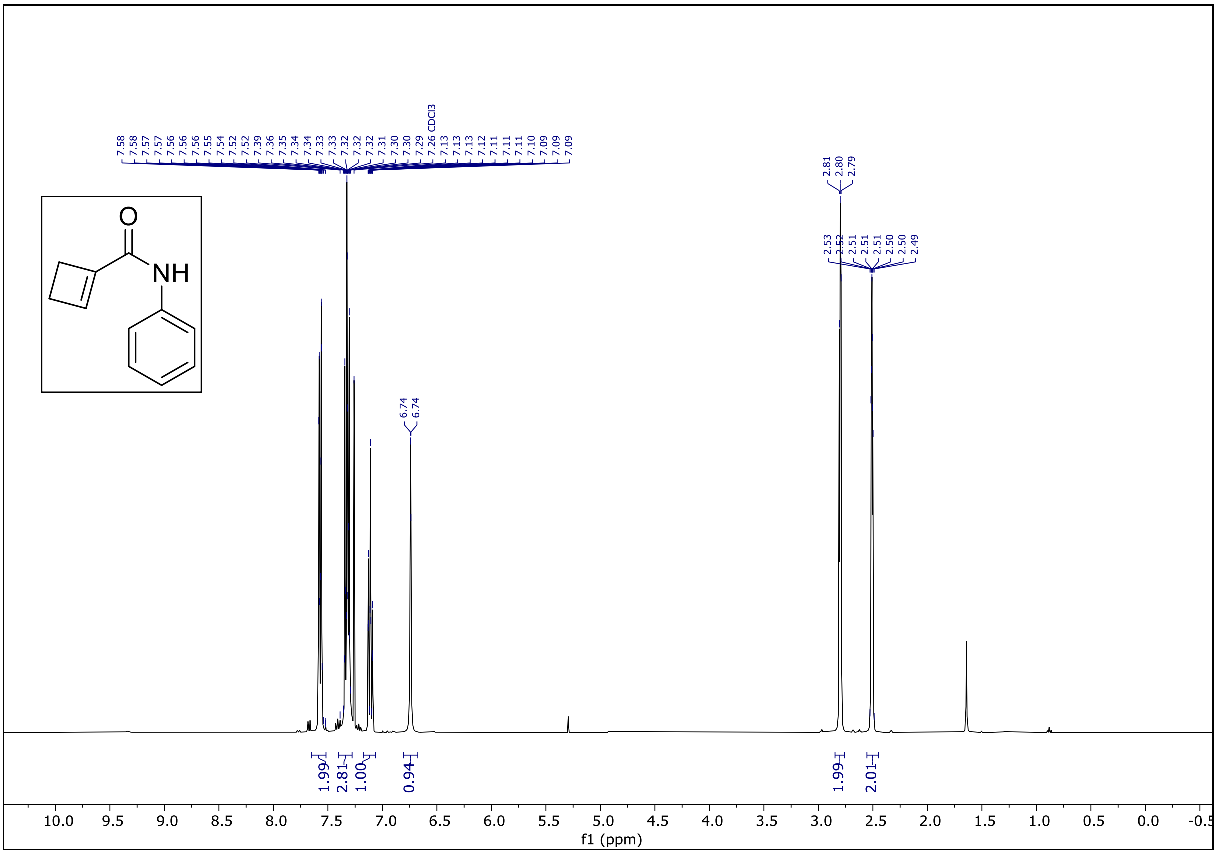

^1H NMR (400 MHz, $CDCl_3$): **S24**

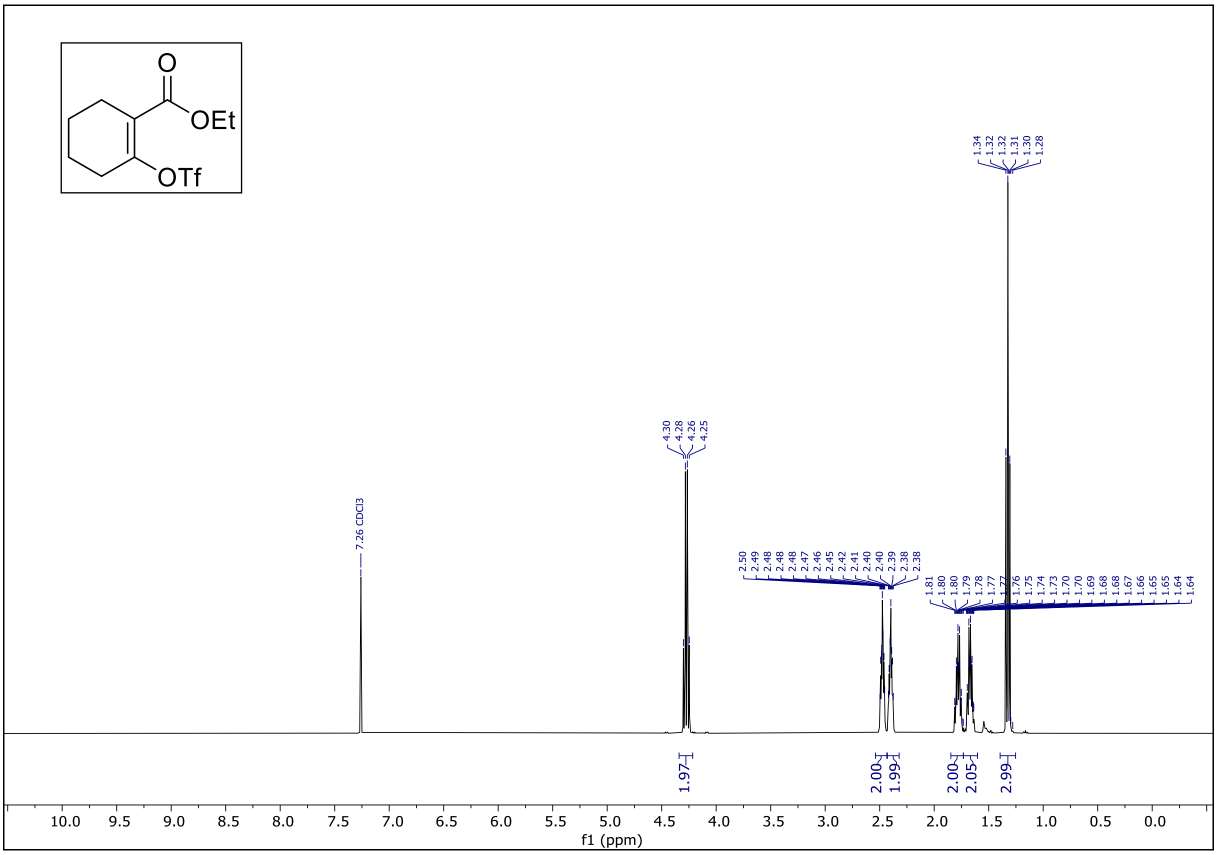

^{19}F NMR (377 MHz, $CDCl_3$): **S24**

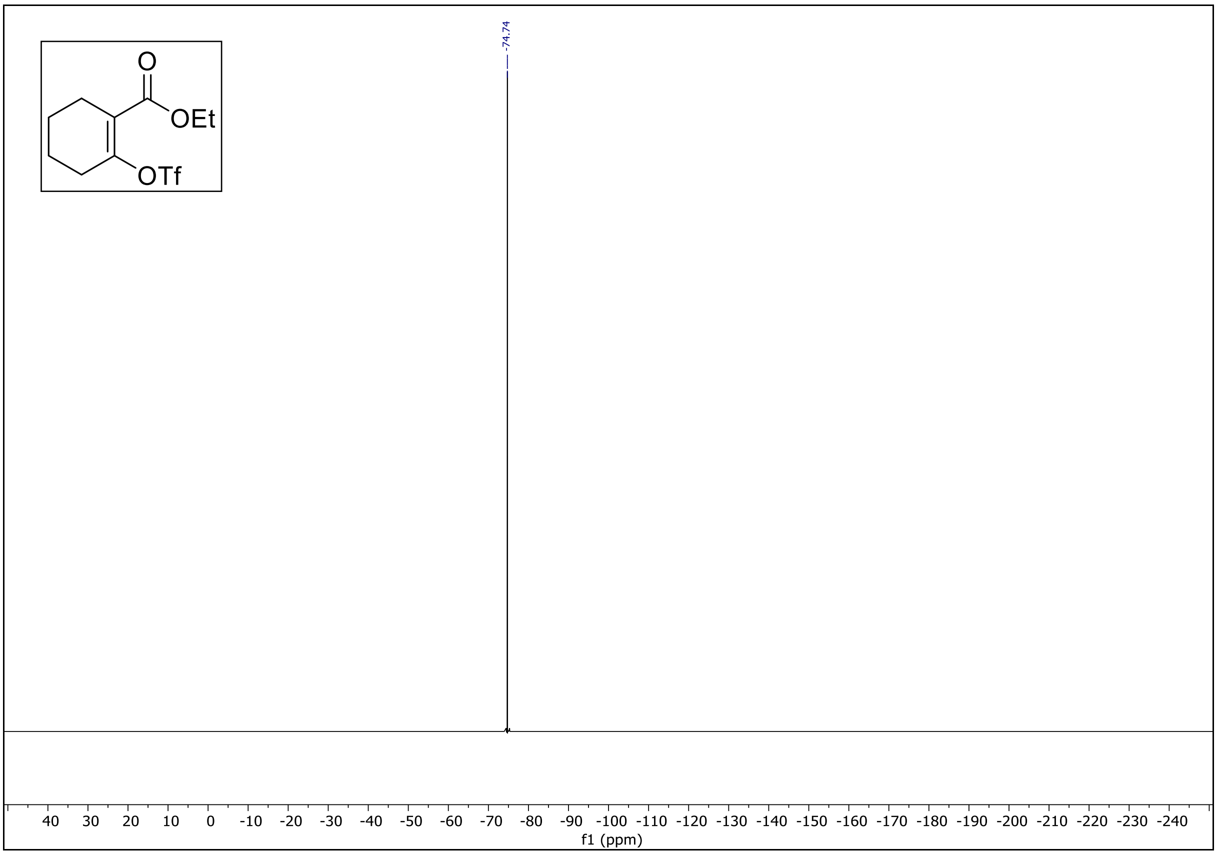

^{1}H NMR (400 MHz, $CDCl_3$): **S25**

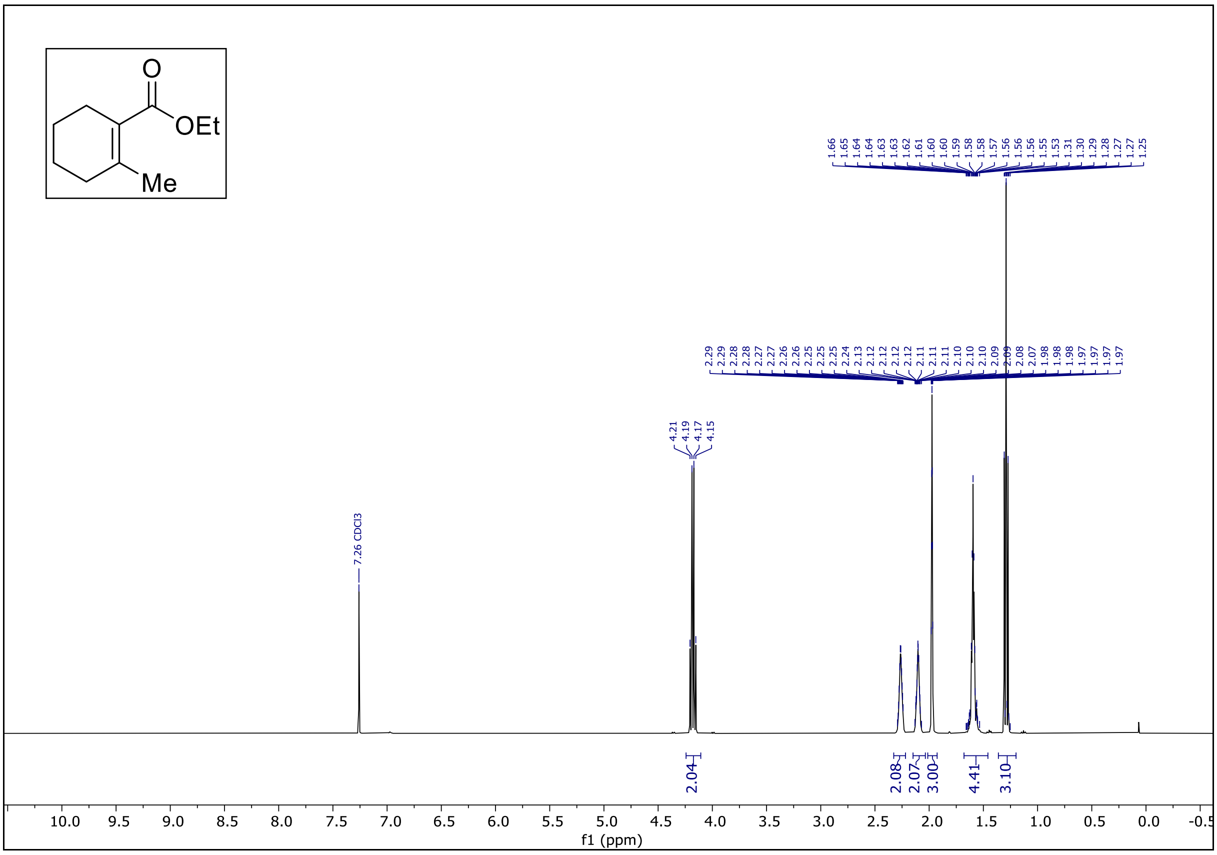

^{1}H NMR (500 MHz, $CDCl_3$): **1k**

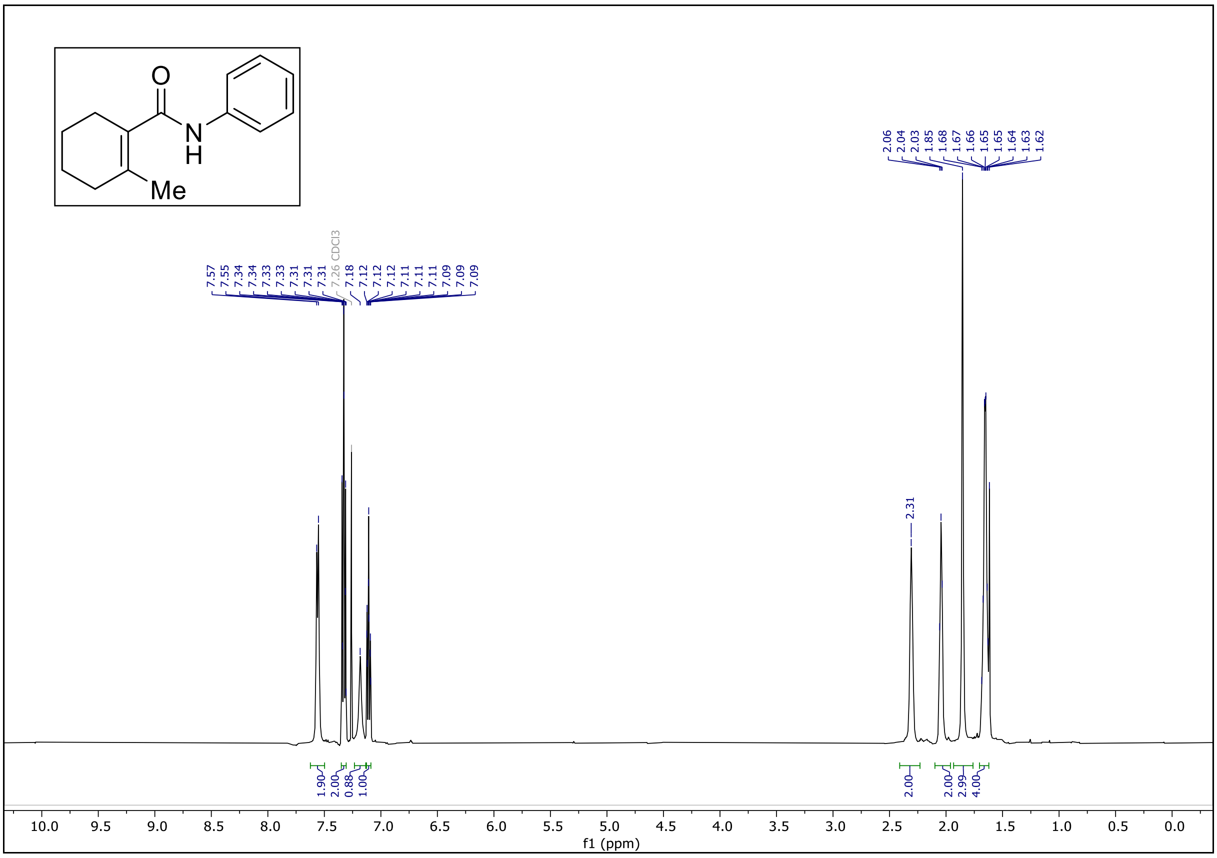

^{13}C NMR (126 MHz, $CDCl_3$): **1k**

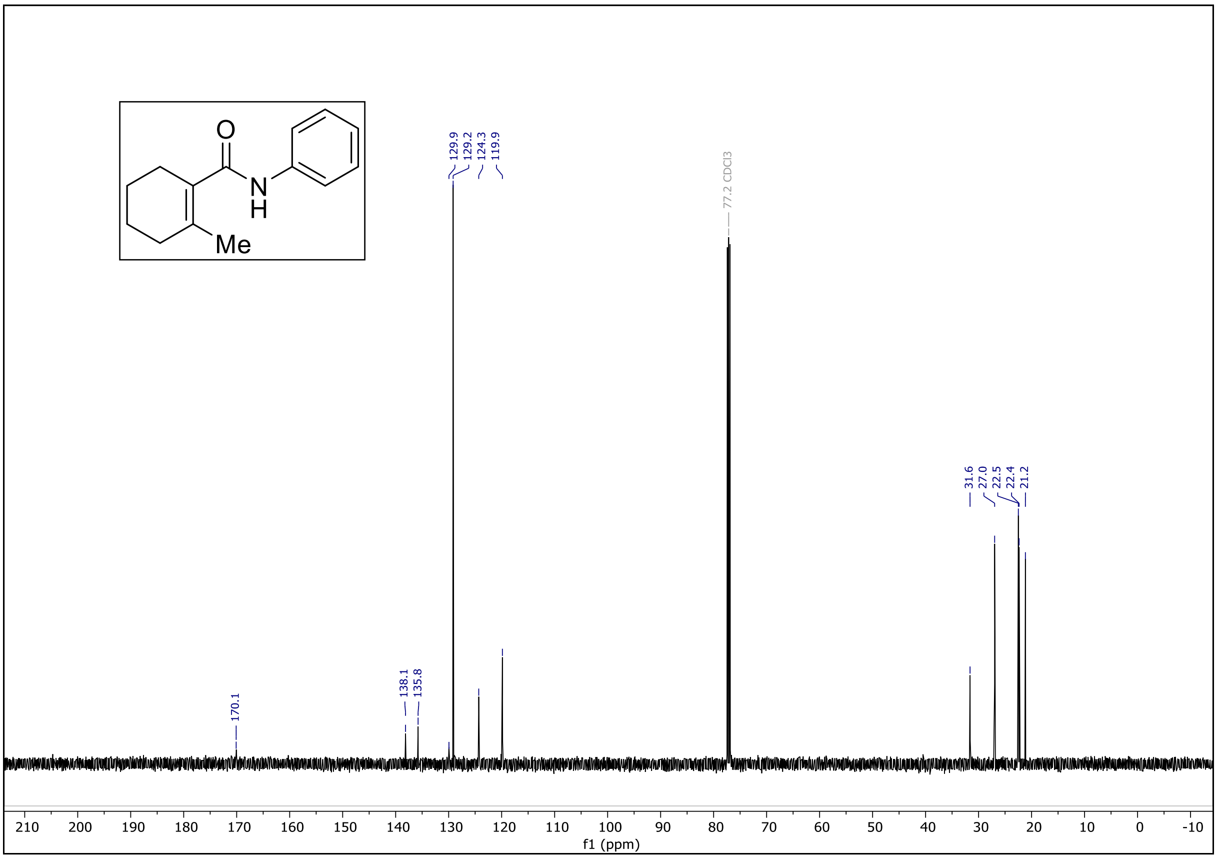

^{1}H NMR (400 MHz, $CDCl_3$): **1l**

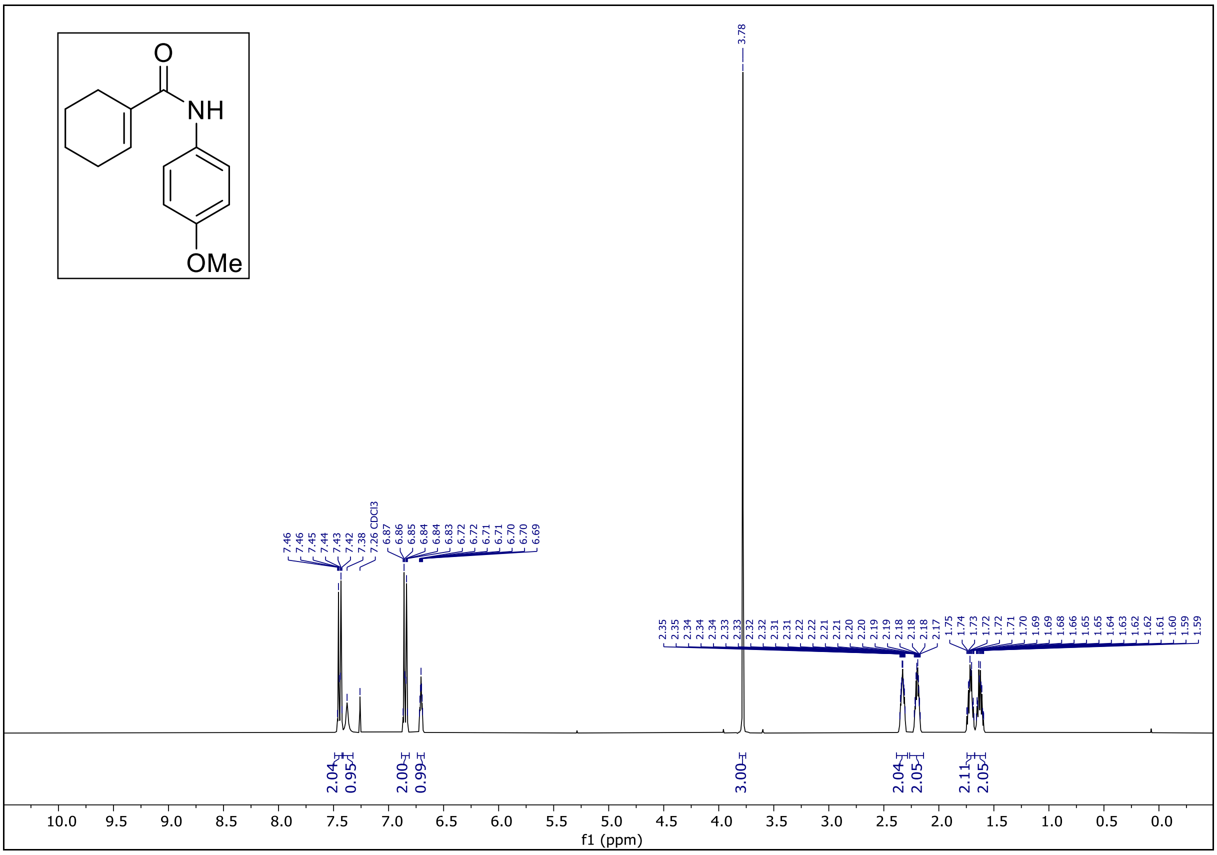

^{1}H NMR (500 MHz, $CDCl_3$): **1m**

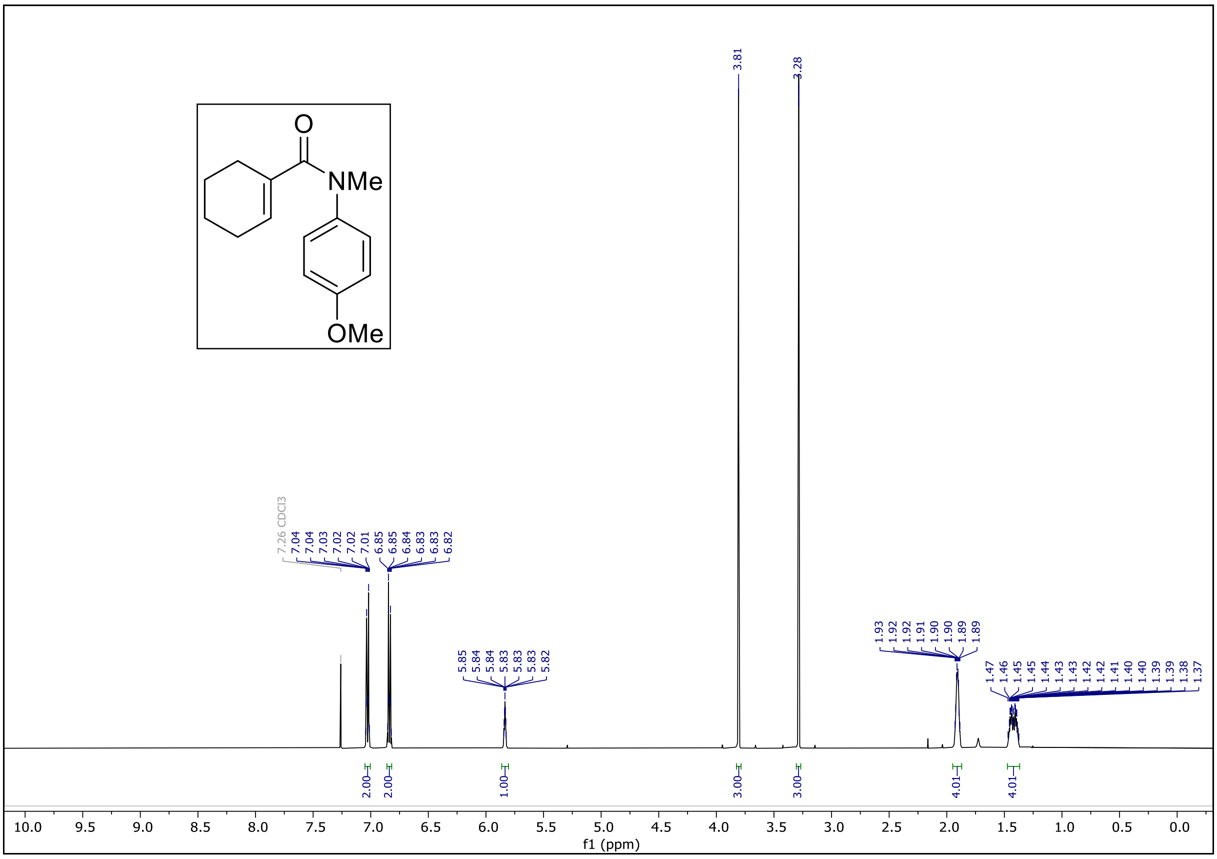

^{13}C NMR (126 MHz, $CDCl_3$): **1m**

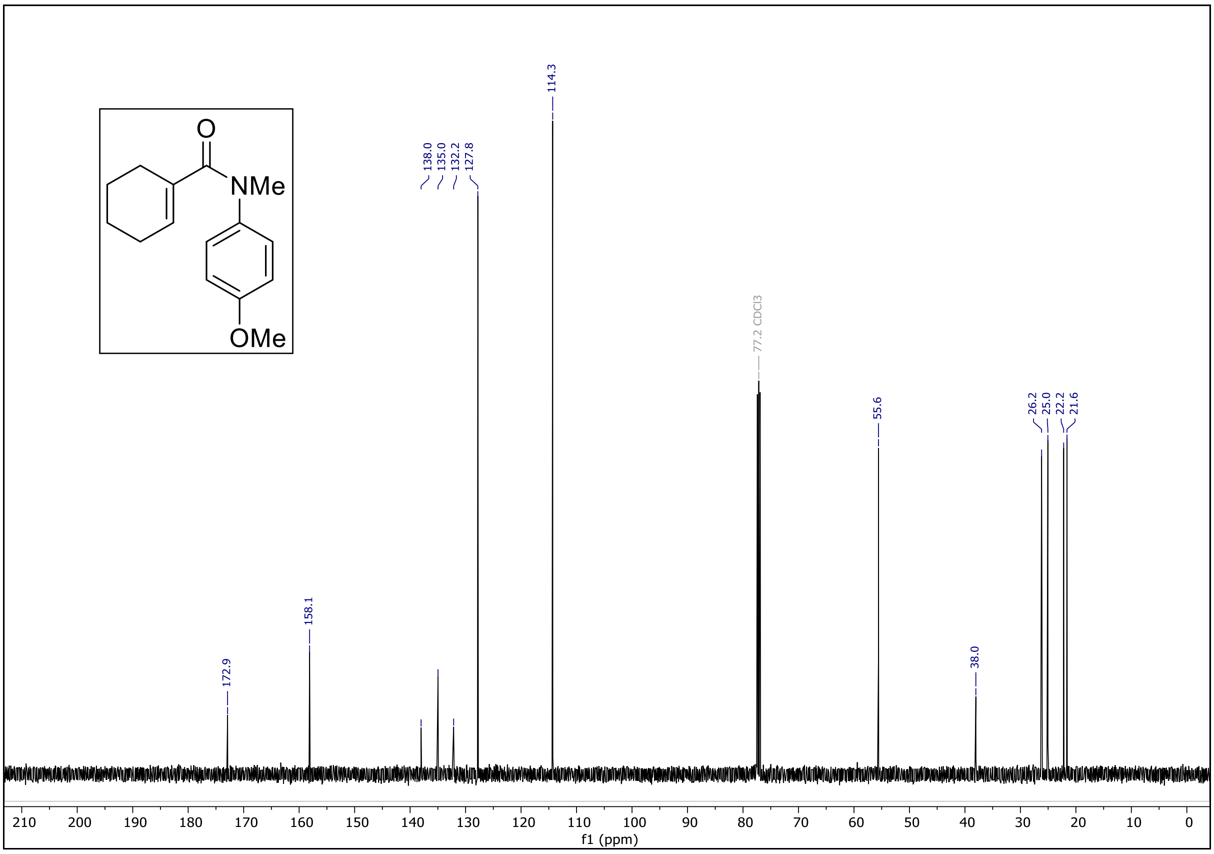

^{1}H NMR (400 MHz, C_6D_6): **S26**

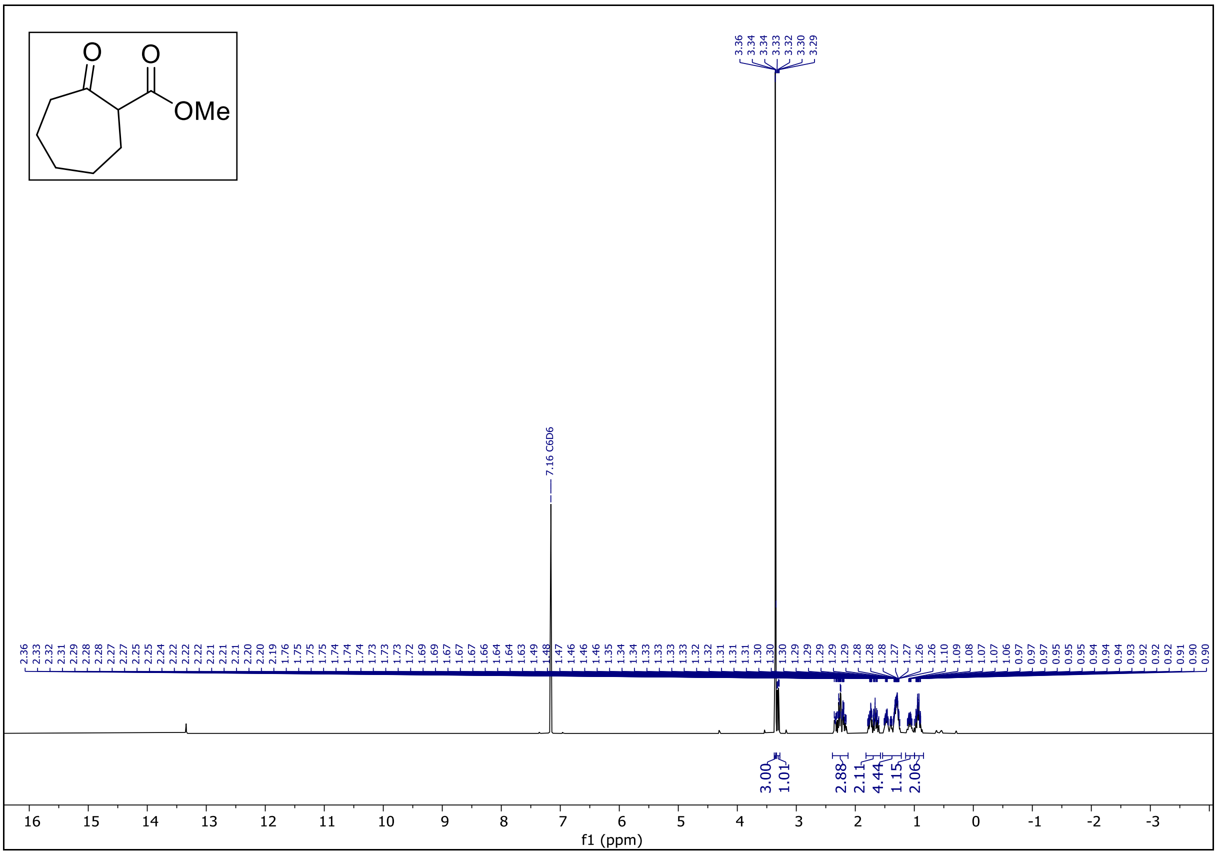

^{1}H NMR (400 MHz, $CDCl_3$): **S27**

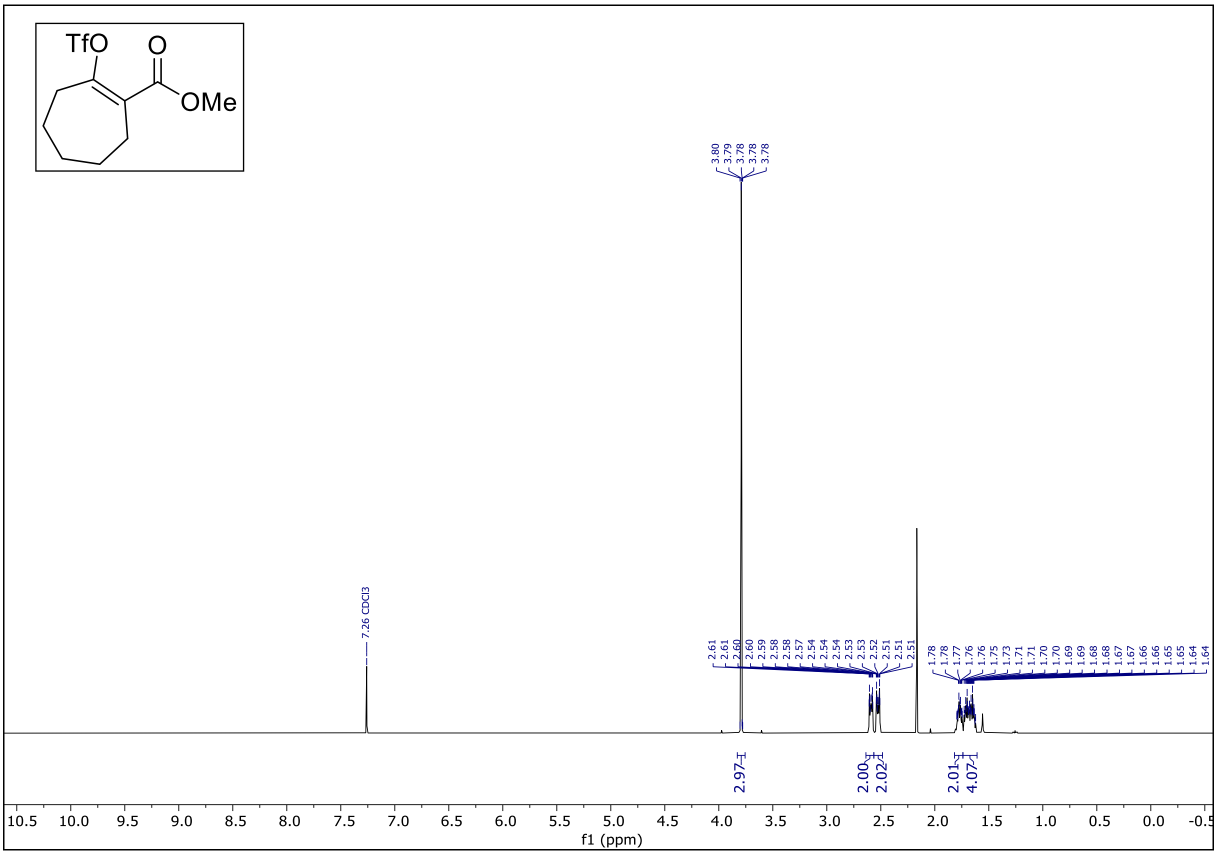

^{19}F NMR (377 MHz, $CDCl_3$): **S27**

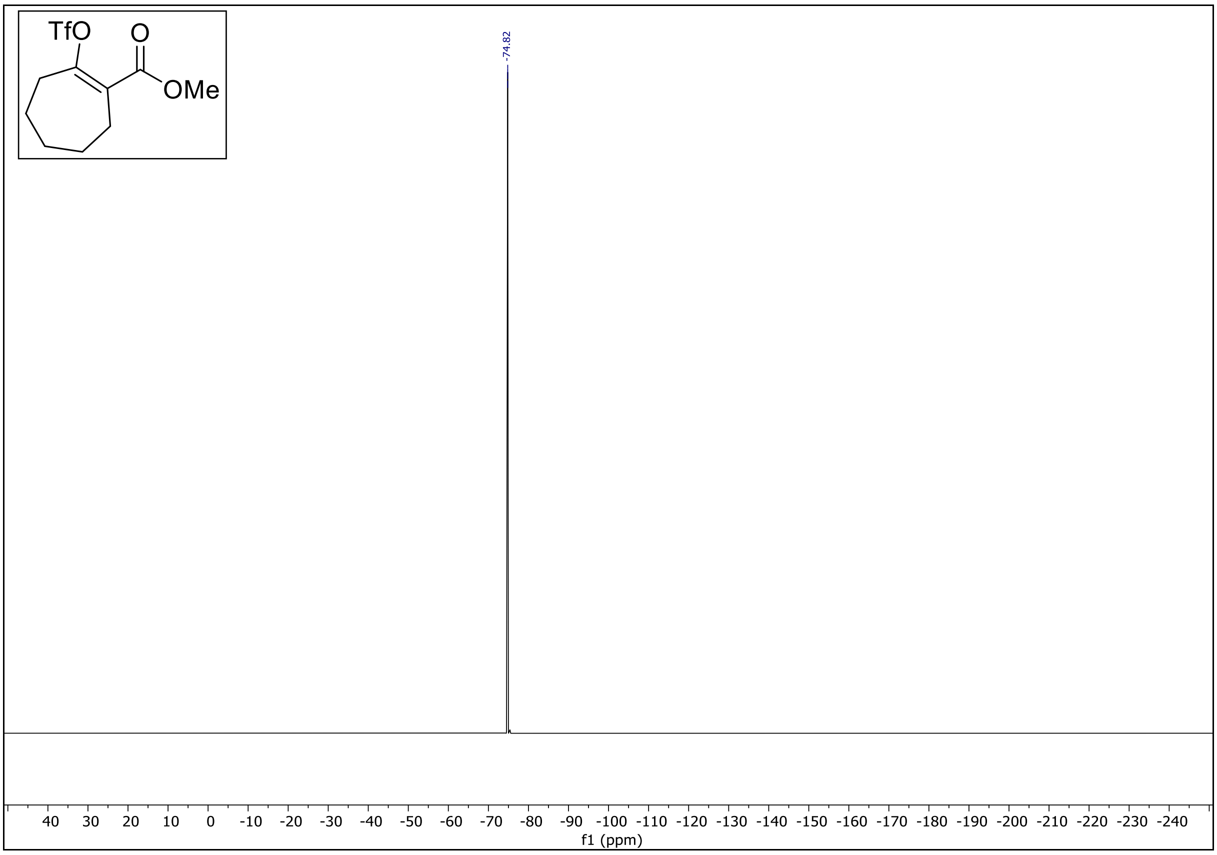

^{1}H NMR (400 MHz, $CDCl_3$): **S28**

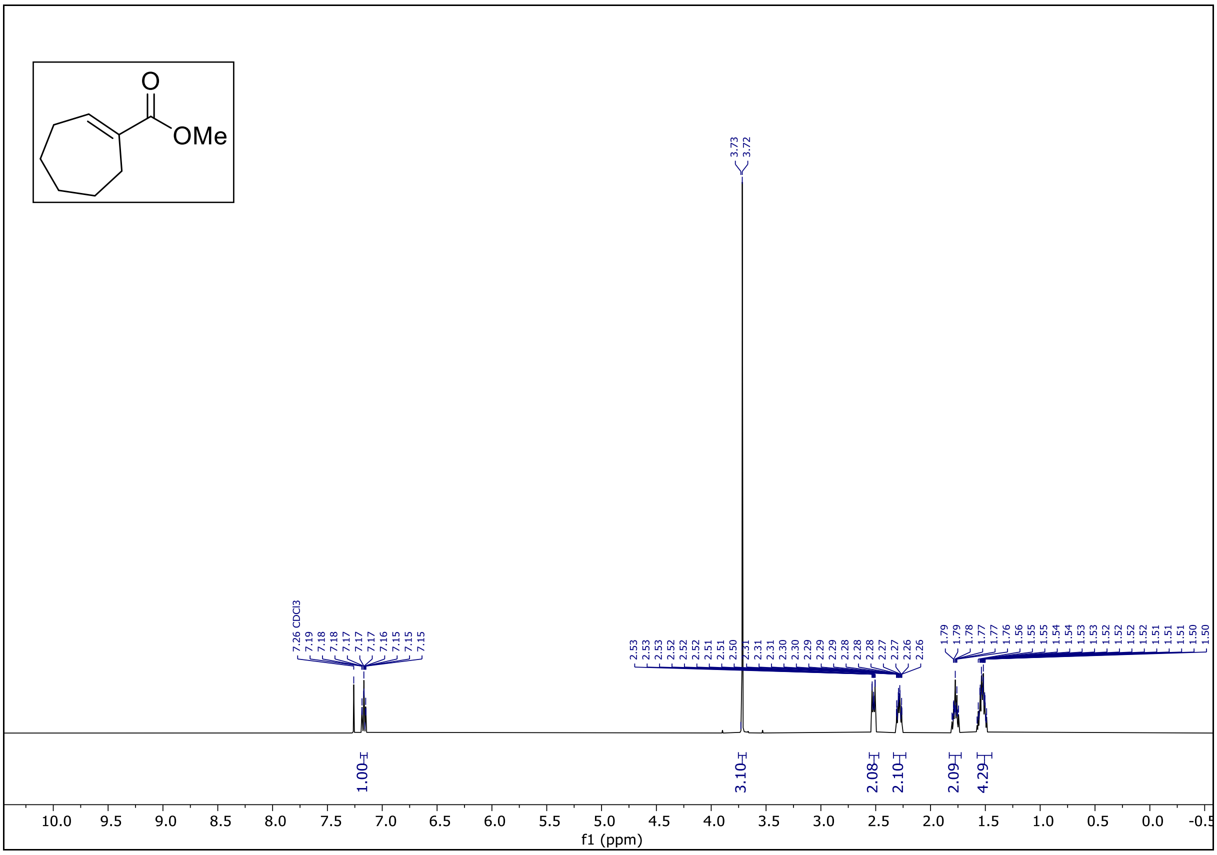

^{1}H NMR (400 MHz, $CDCl_3$): **1n**

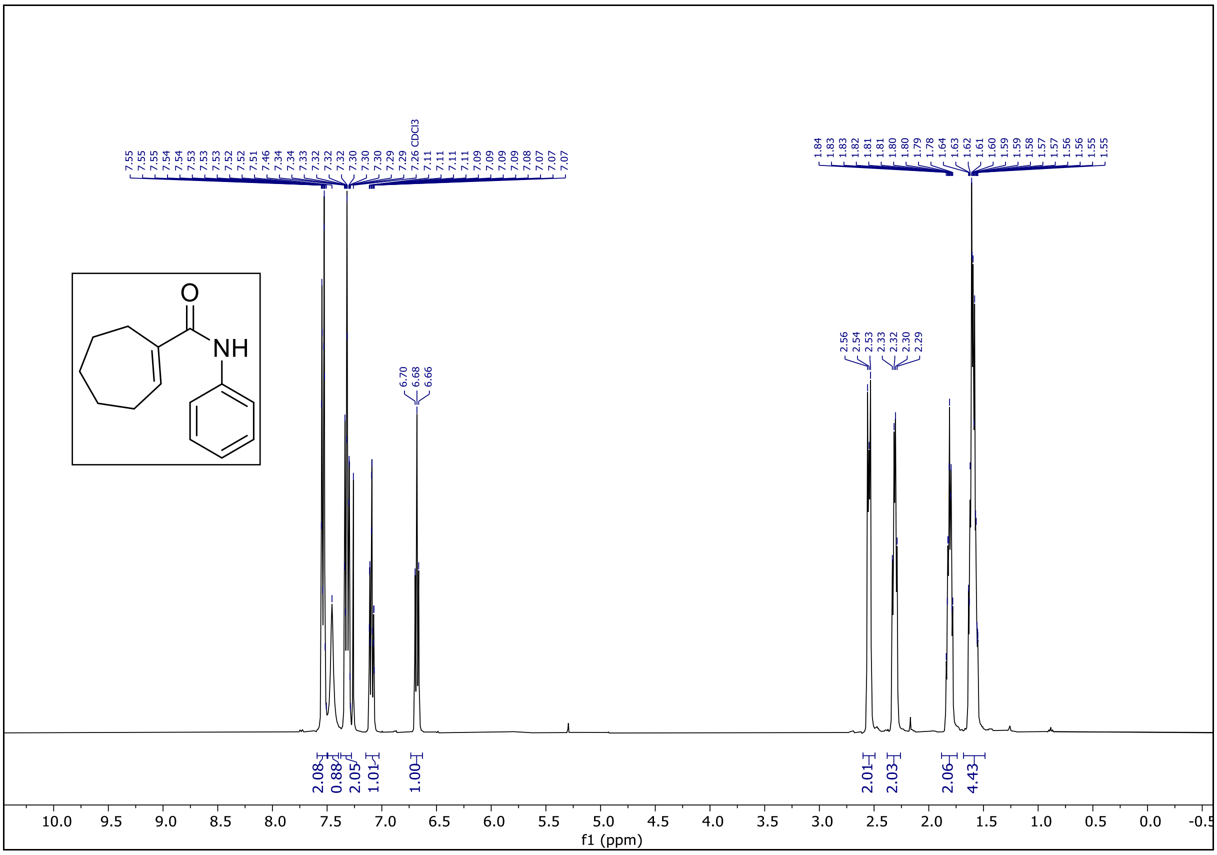

^{1}H NMR (600 MHz, $CDCl_3$): **1o**

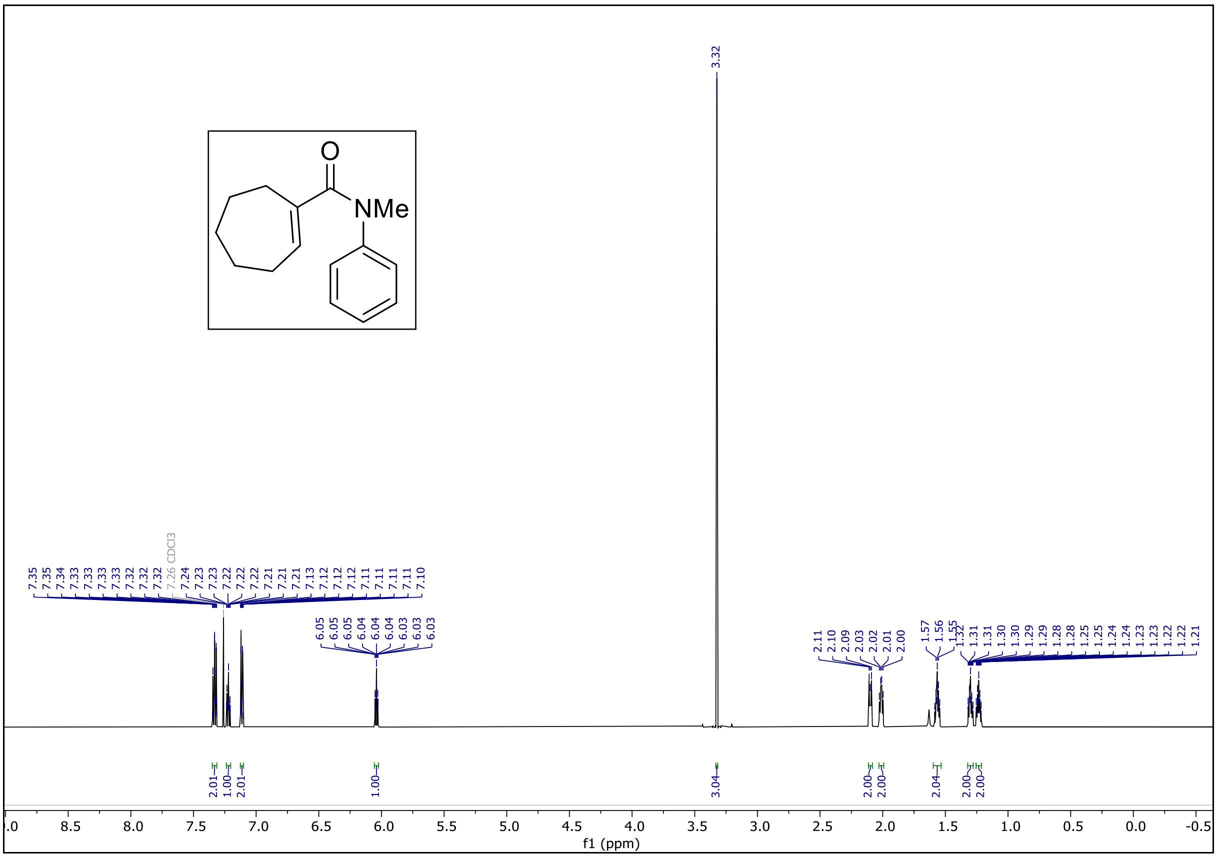

^{13}C NMR (151 MHz, $CDCl_3$): **1o**

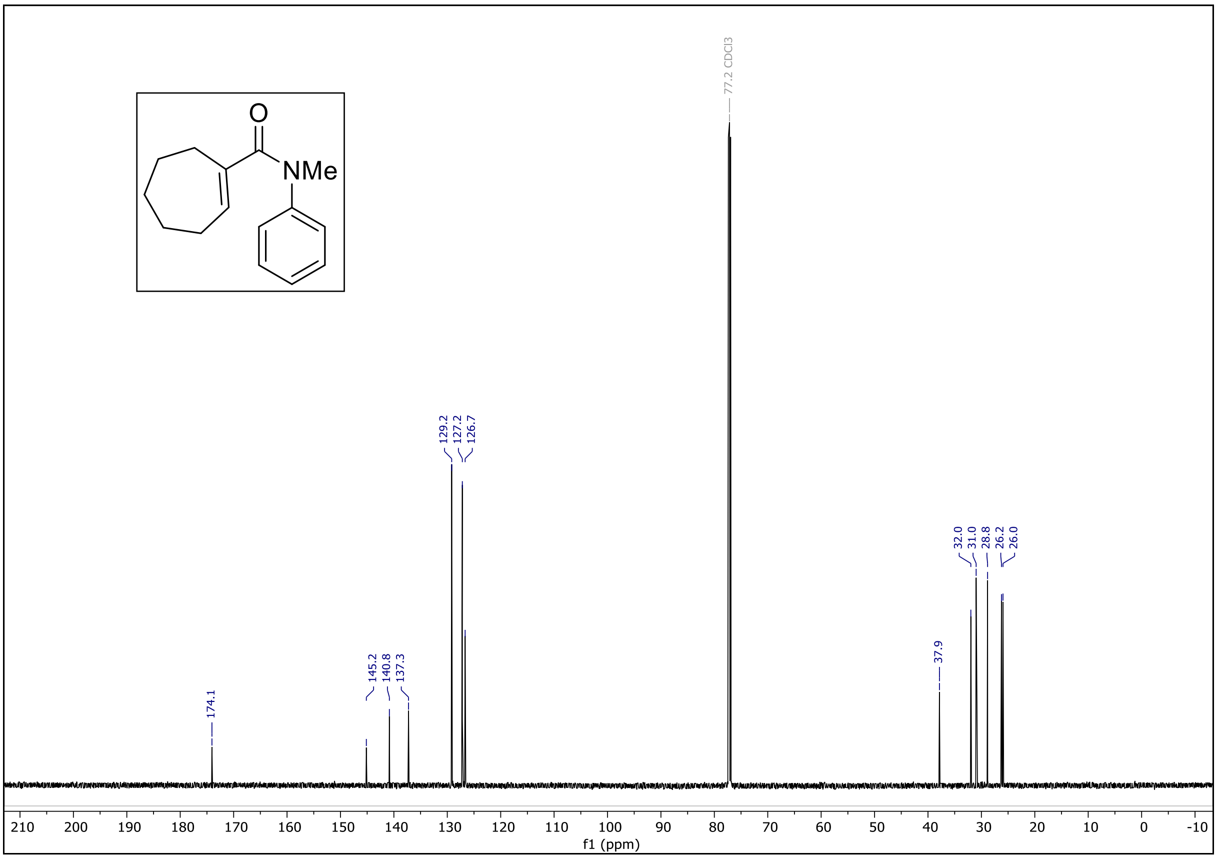

^{1}H NMR (400 MHz, $CDCl_3$): **S29**

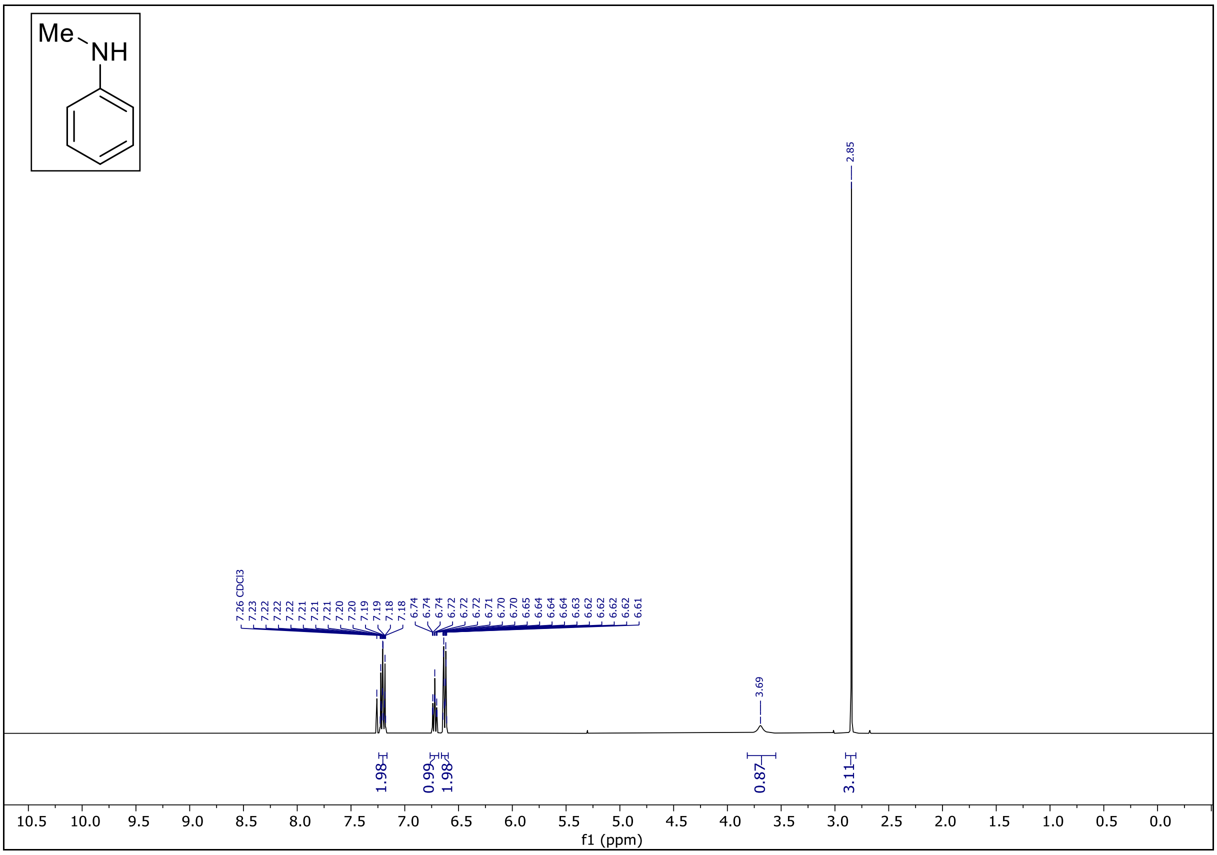

^{1}H NMR (400 MHz, $CDCl_3$): **1p**

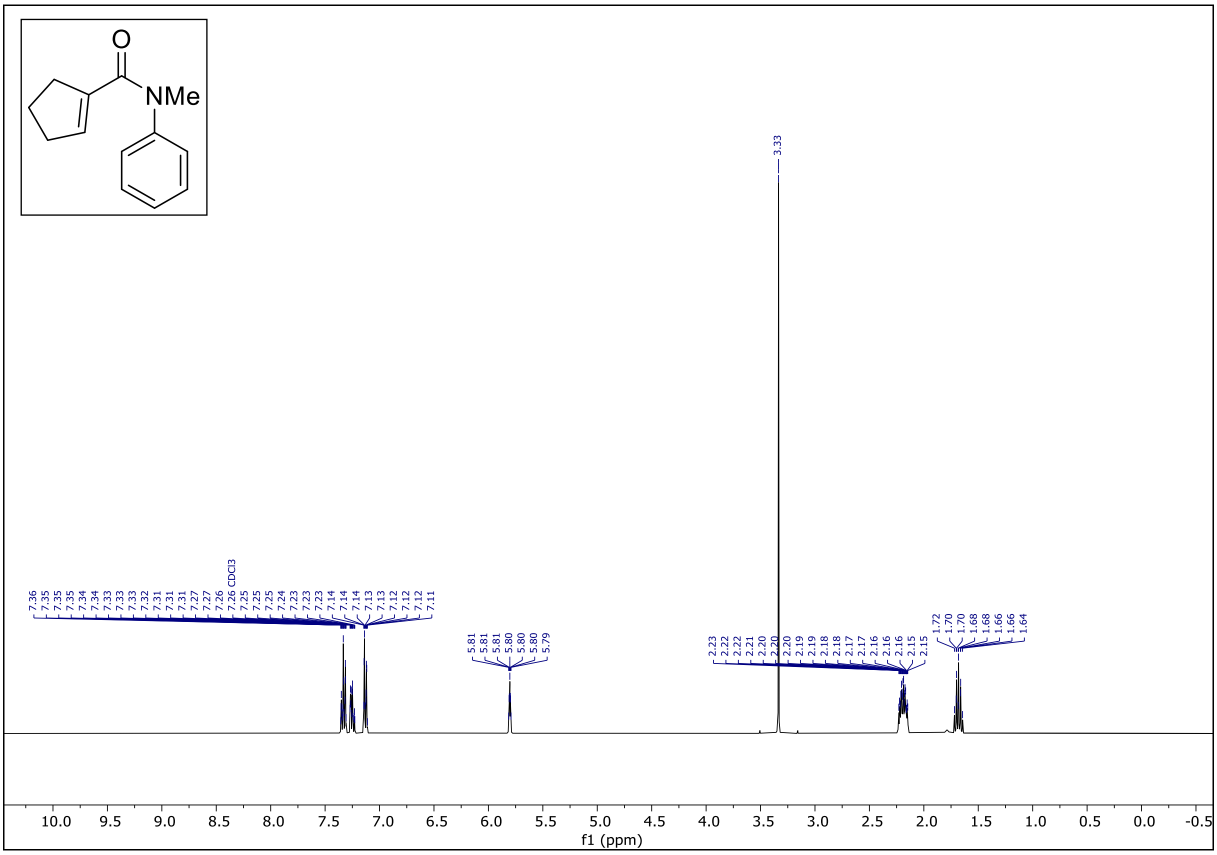

^{1}H NMR (400 MHz, $CDCl_3$): **1q**

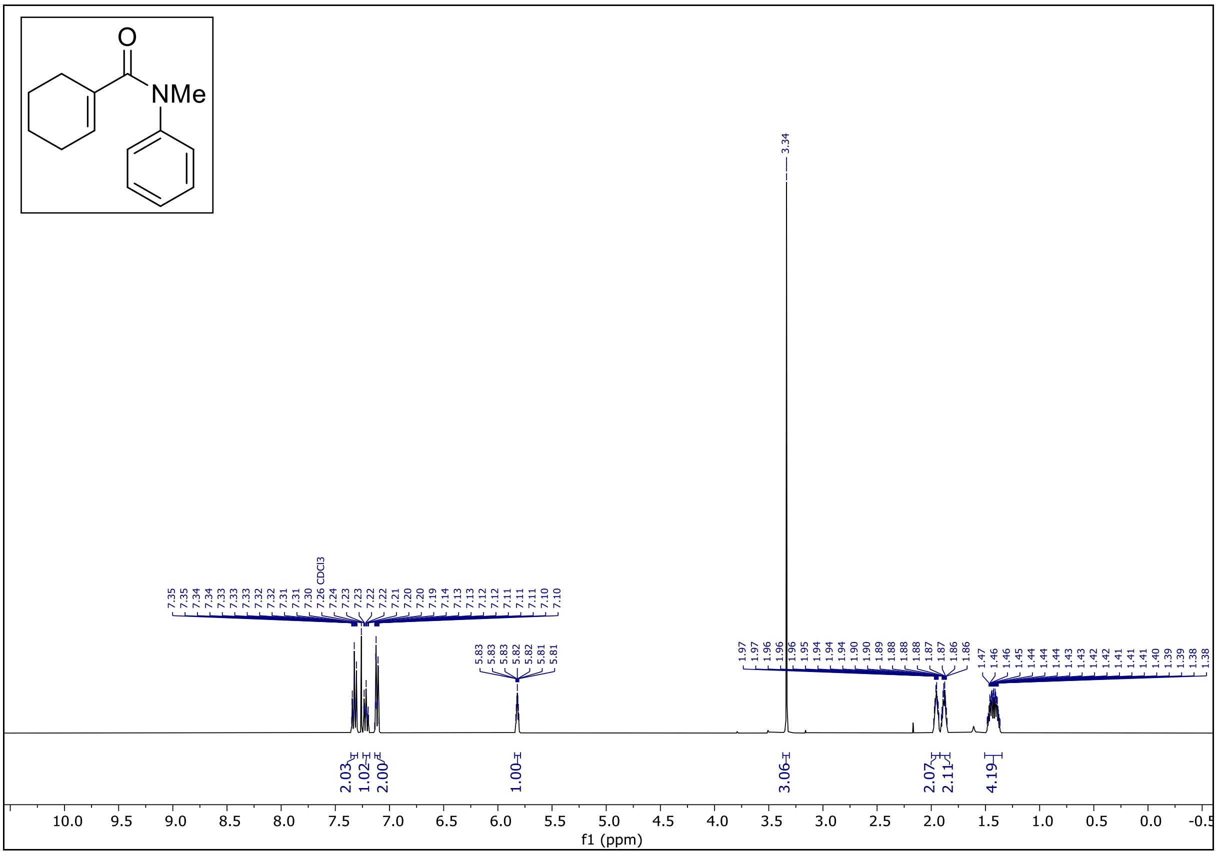

^{1}H NMR (600 MHz, $CDCl_3$): **1r**

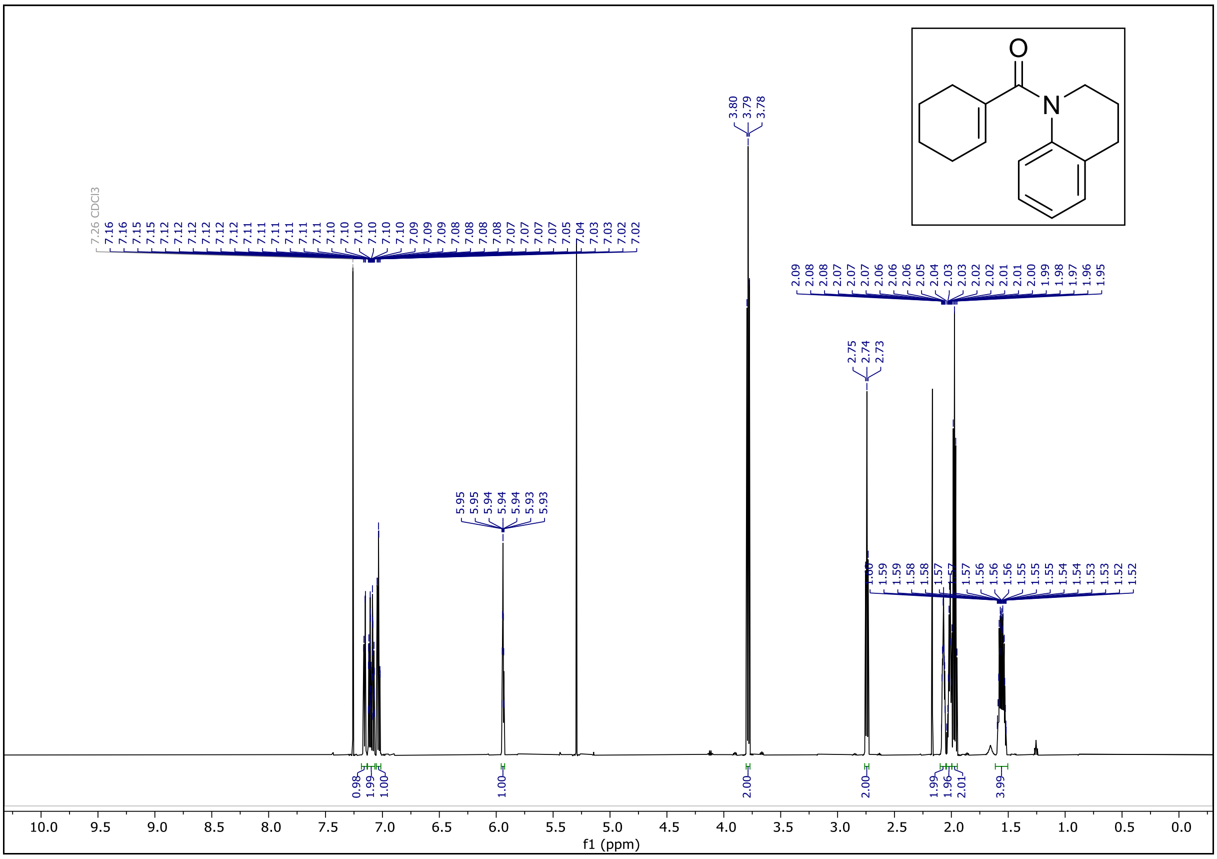

^{13}C NMR (151 MHz, $CDCl_3$): **1r**

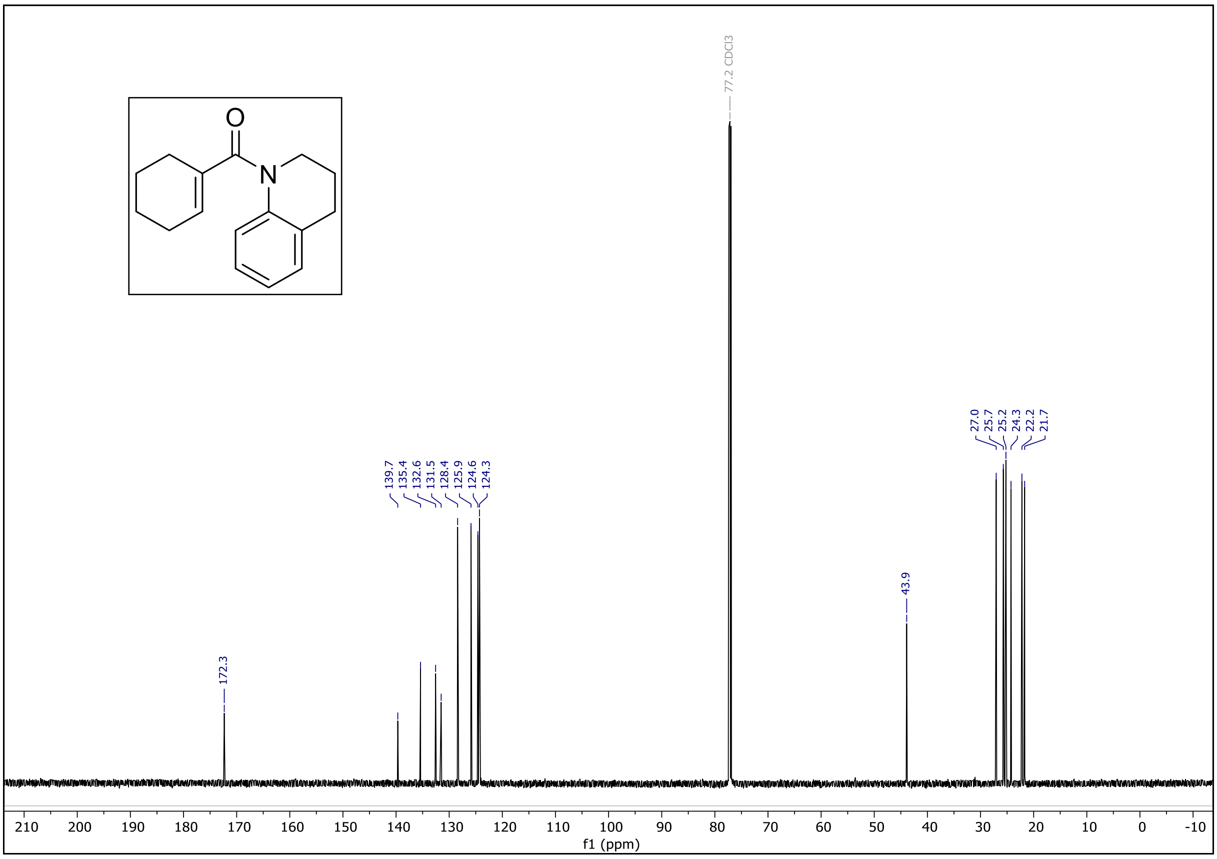

^1H NMR (500 MHz, $CDCl_3$): **1s**

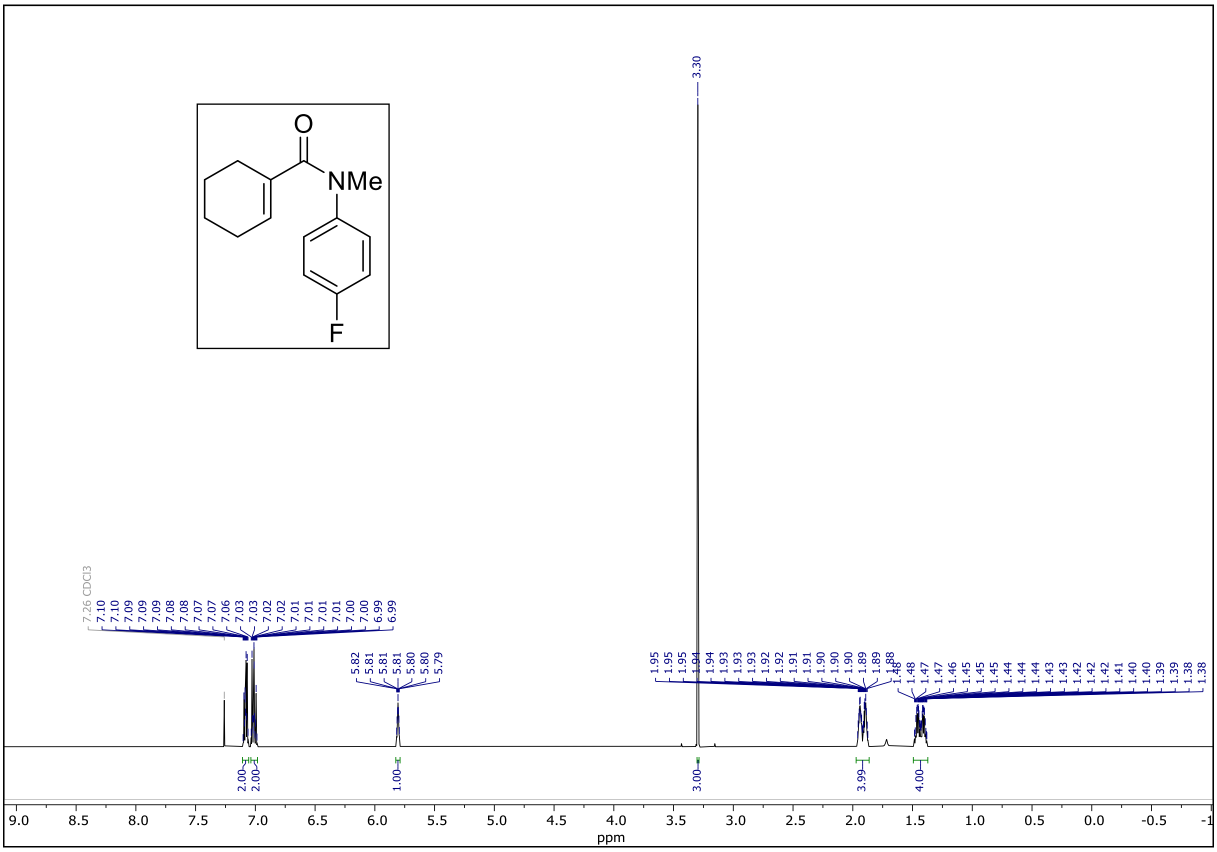

^{13}C NMR (126 MHz, $CDCl_3$): **1s**

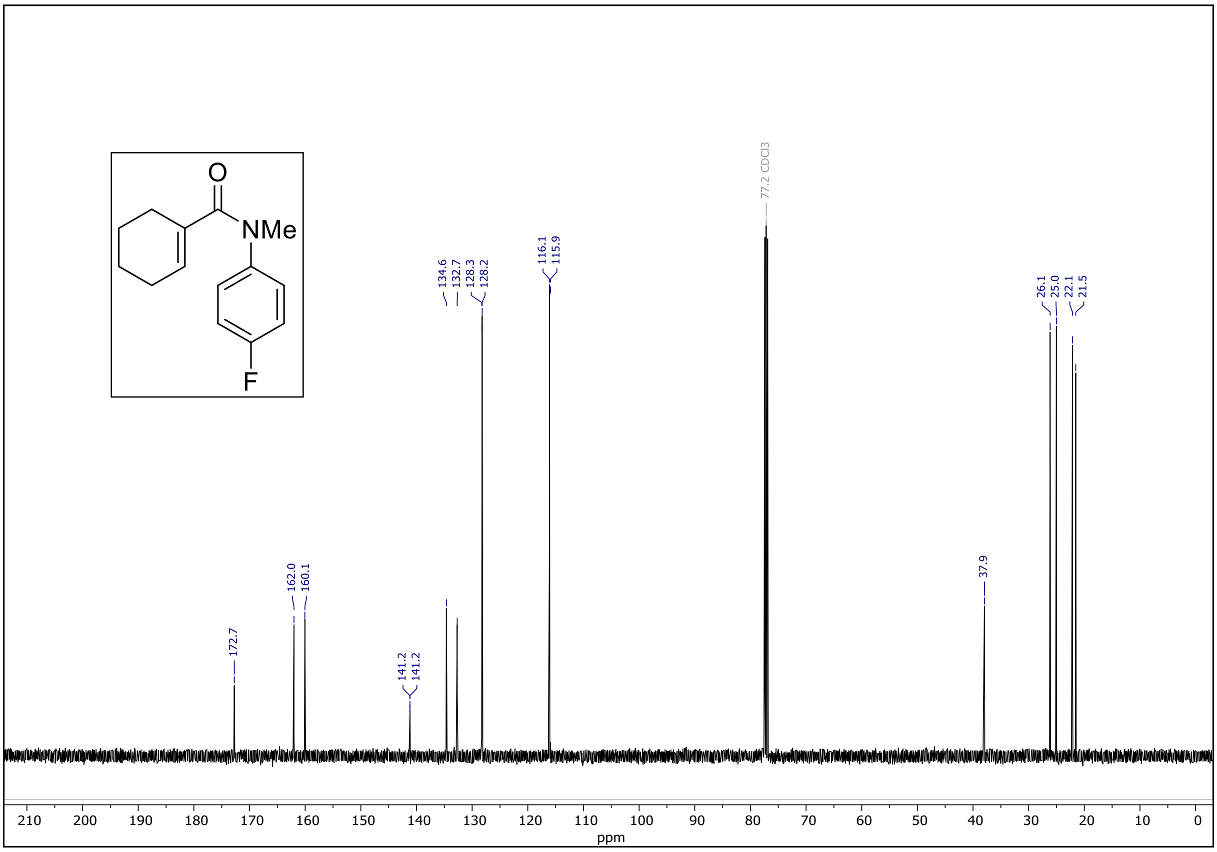

^{19}F NMR (470 MHz, $CDCl_3$): **1s**

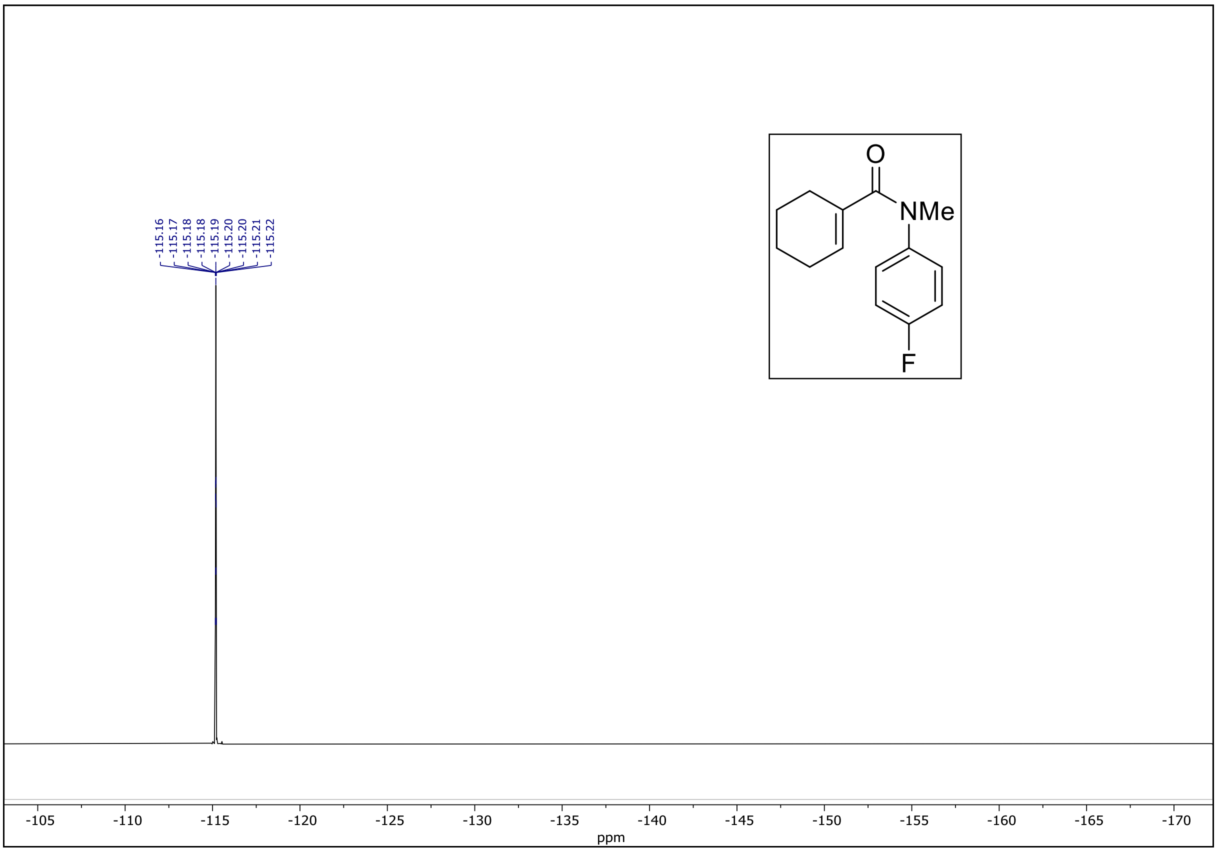

^{1}H NMR (400 MHz, $CDCl_3$): **S30**

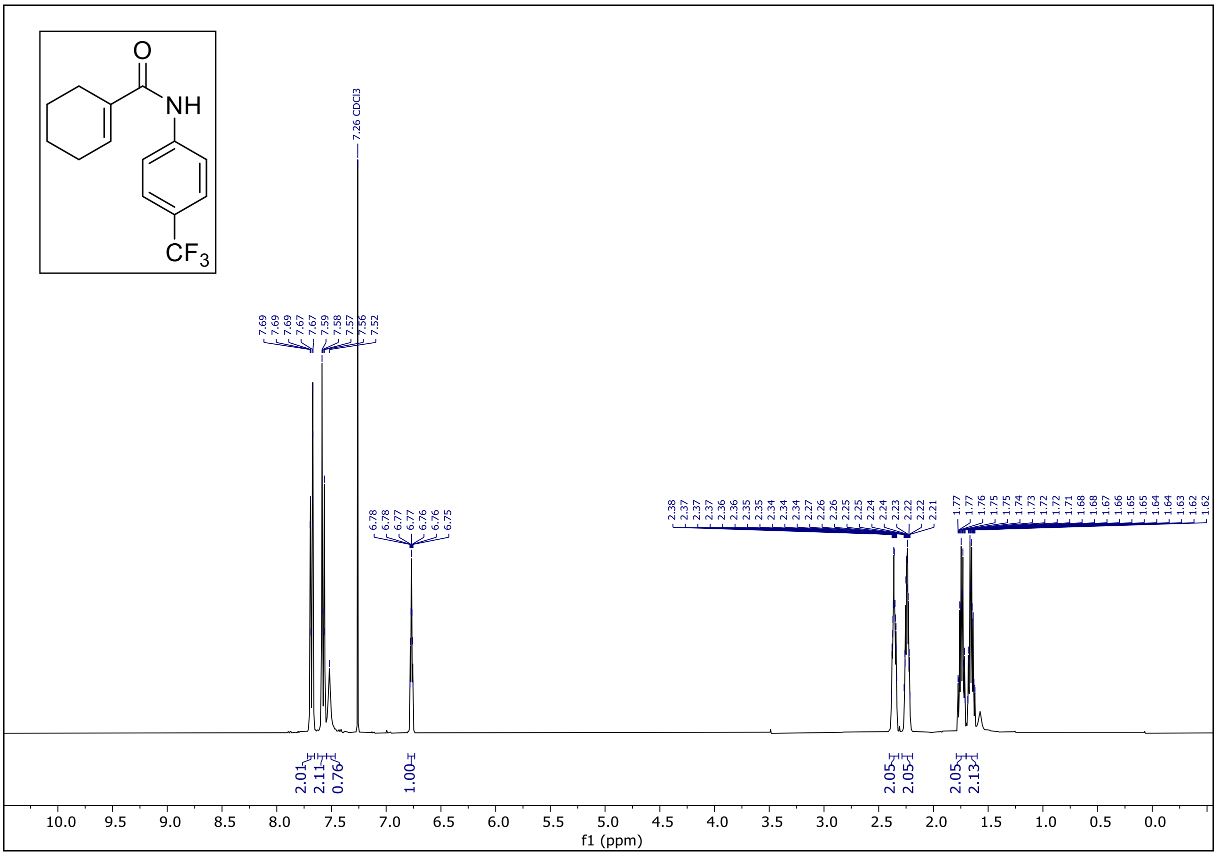

^{19}F NMR (377 MHz, $CDCl_3$): **S30**

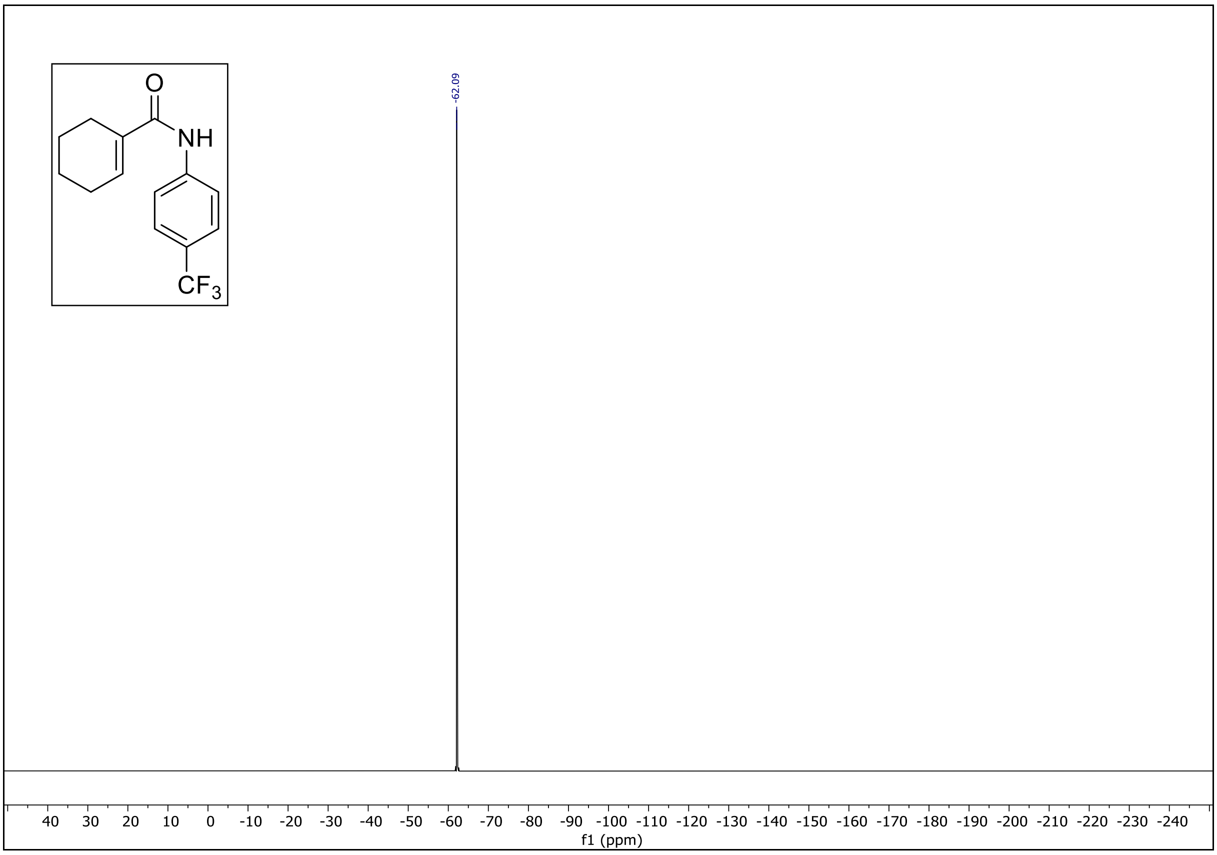

^{1}H NMR (500 MHz, $CDCl_3$): **1t**

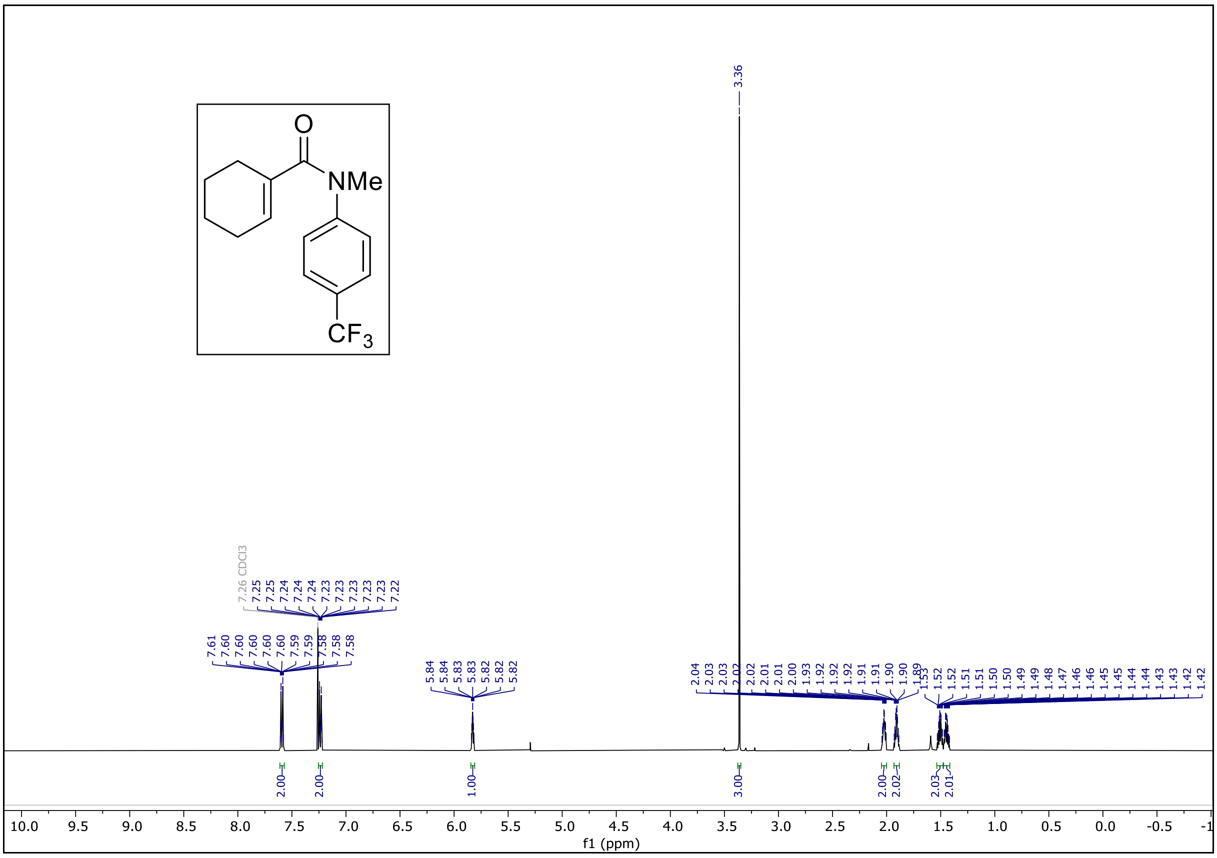

^{13}C NMR (126 MHz, $CDCl_3$): **1t**

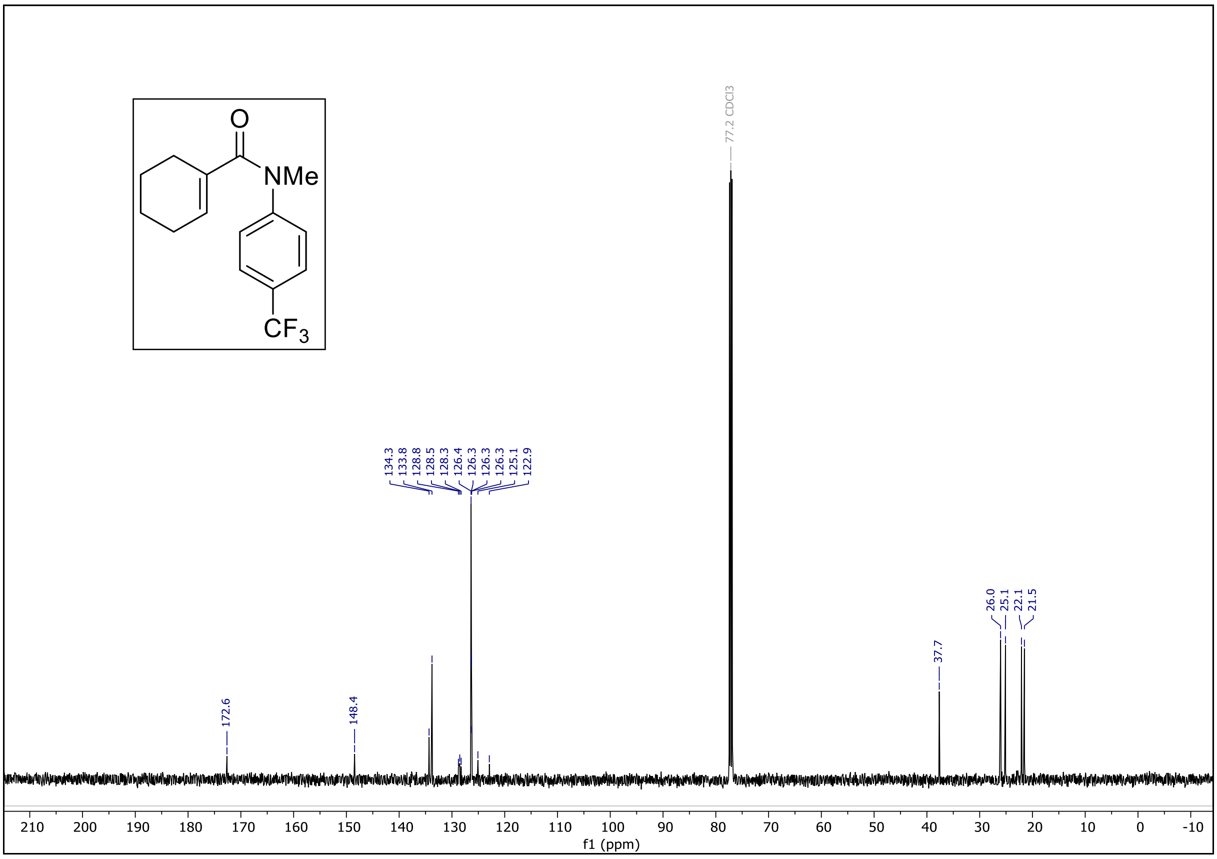

^{19}F NMR {^{1}H} (470 MHz, $CDCl_3$): **1t**

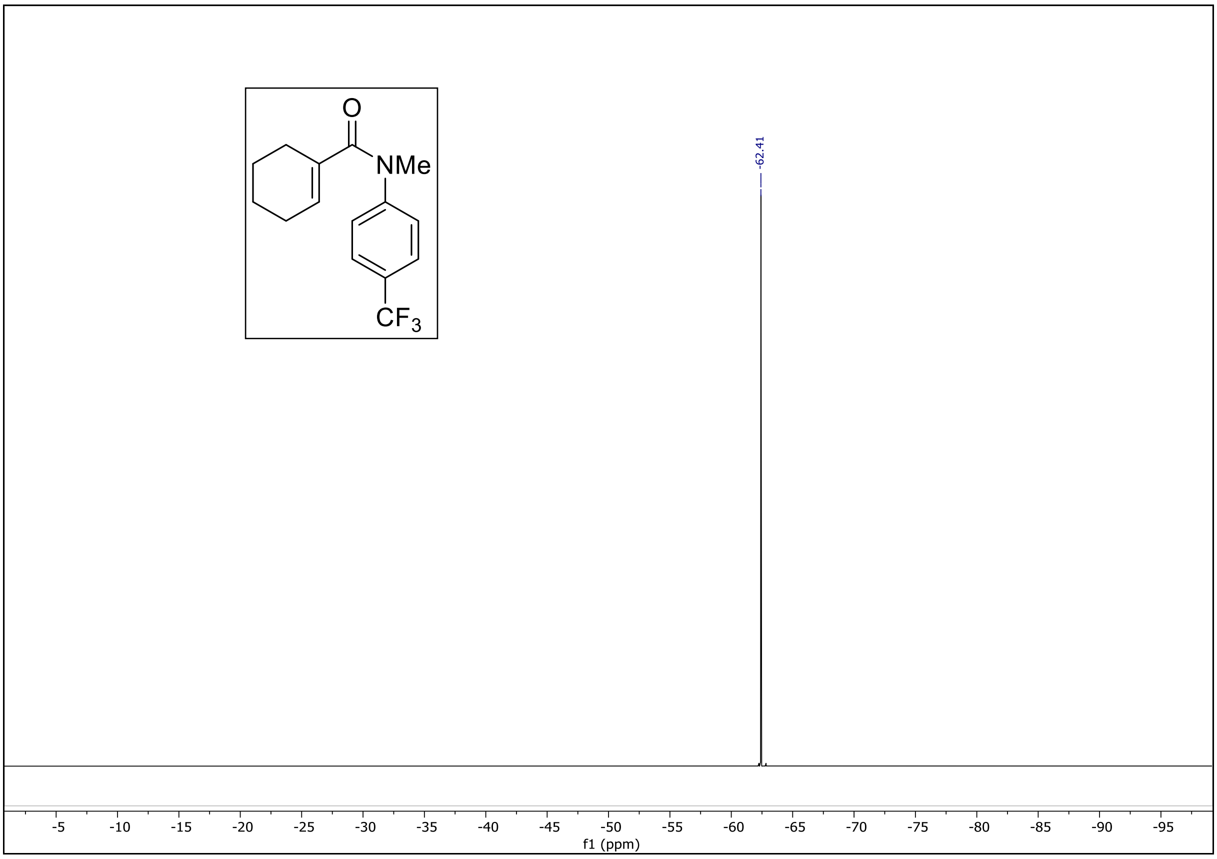

^{1}H NMR (400 MHz, $CDCl_3$): **S31**

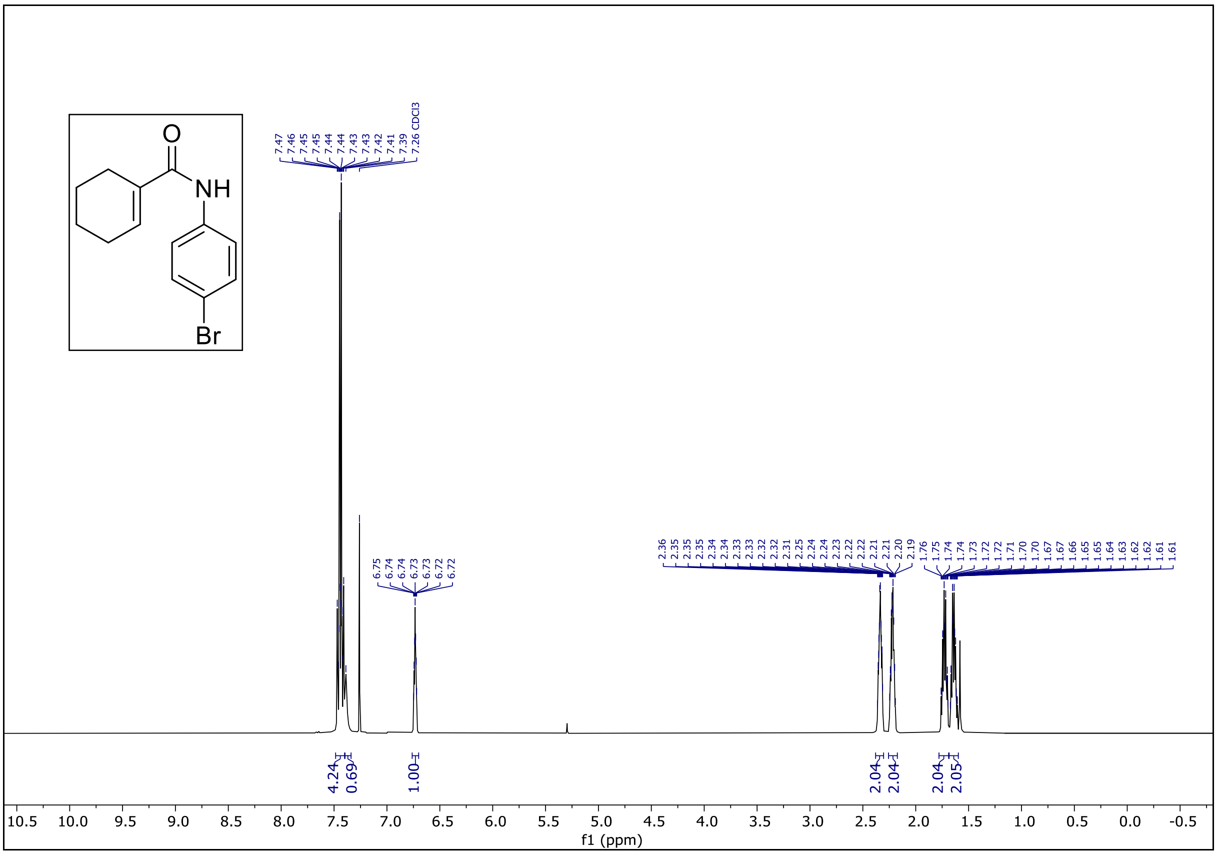

^{1}H NMR (500 MHz, $CDCl_3$): **1u**

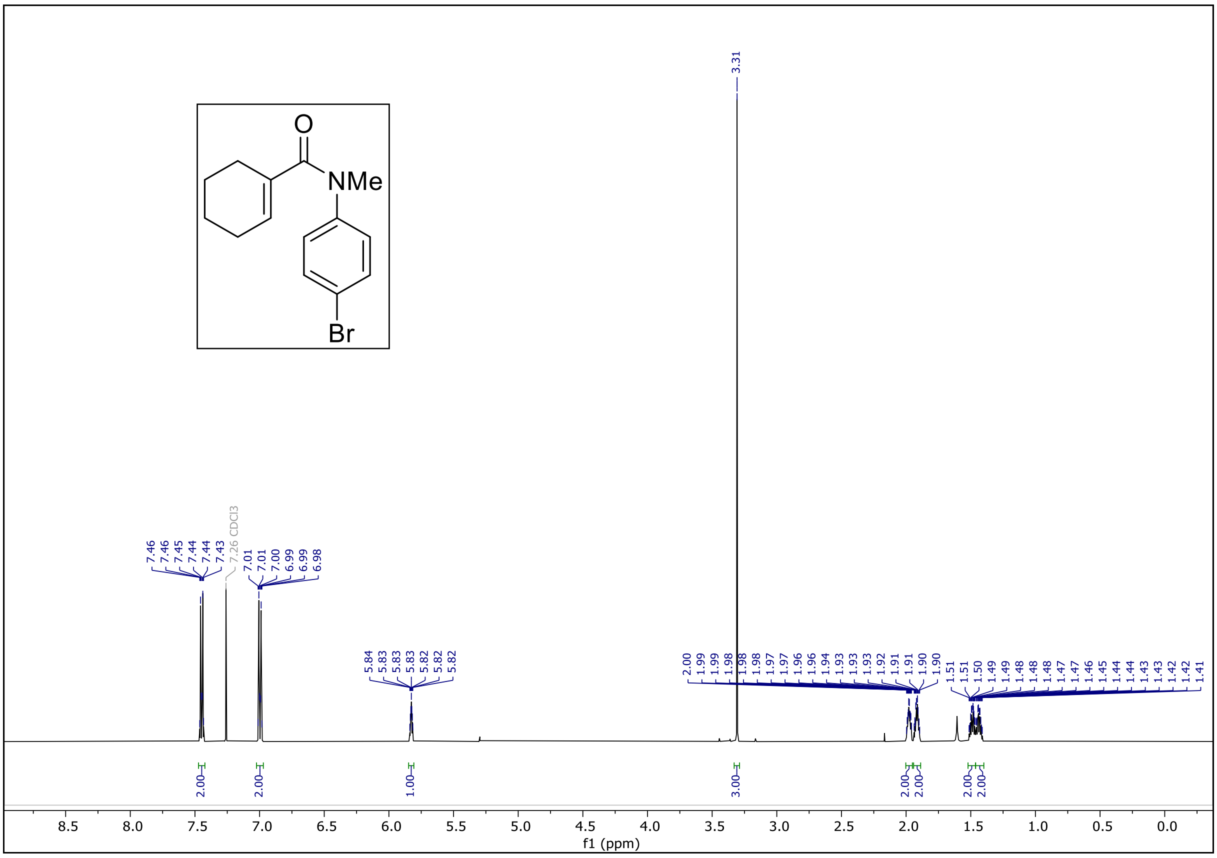

^{13}C NMR (126 MHz, $CDCl_3$): **1u**

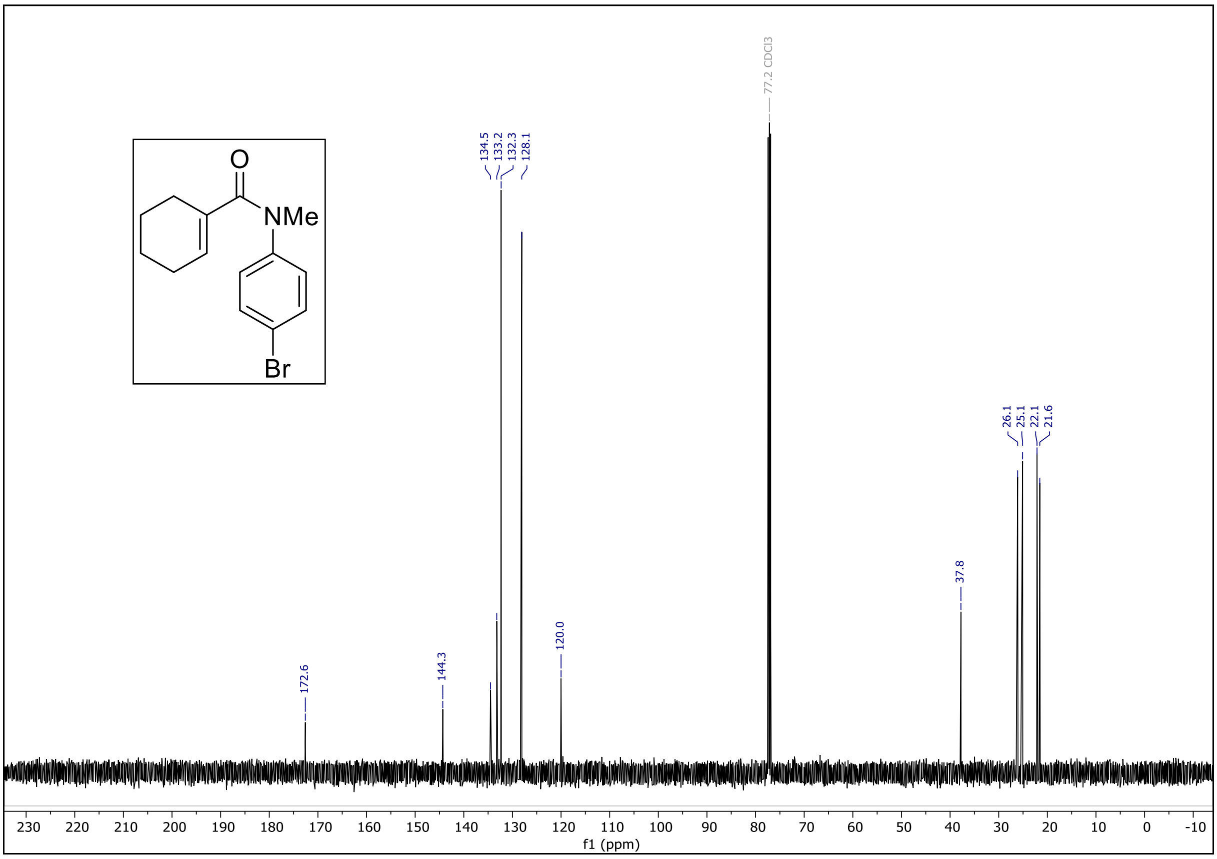

^{1}H NMR (600 MHz, $CDCl_3$): **1v**

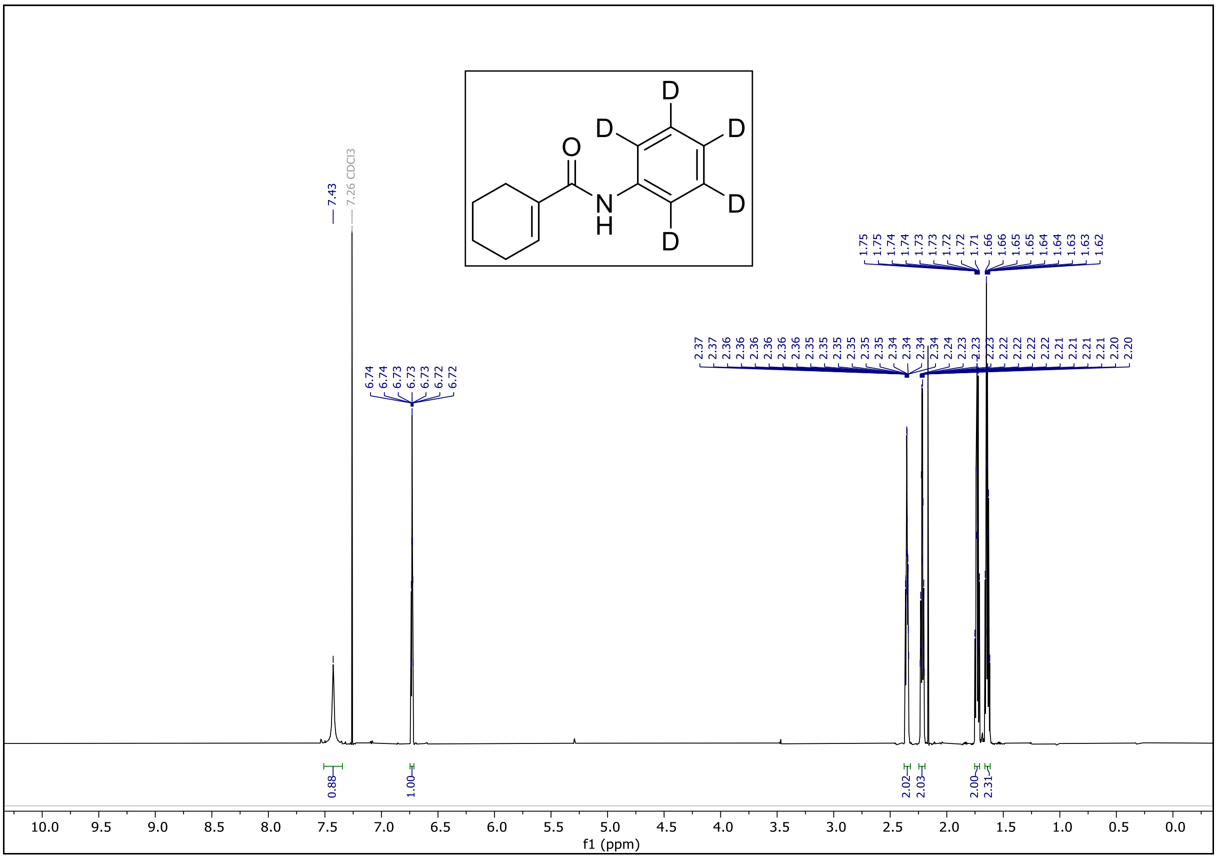

^{13}C NMR (151 MHz, $CDCl_3$): **1v**

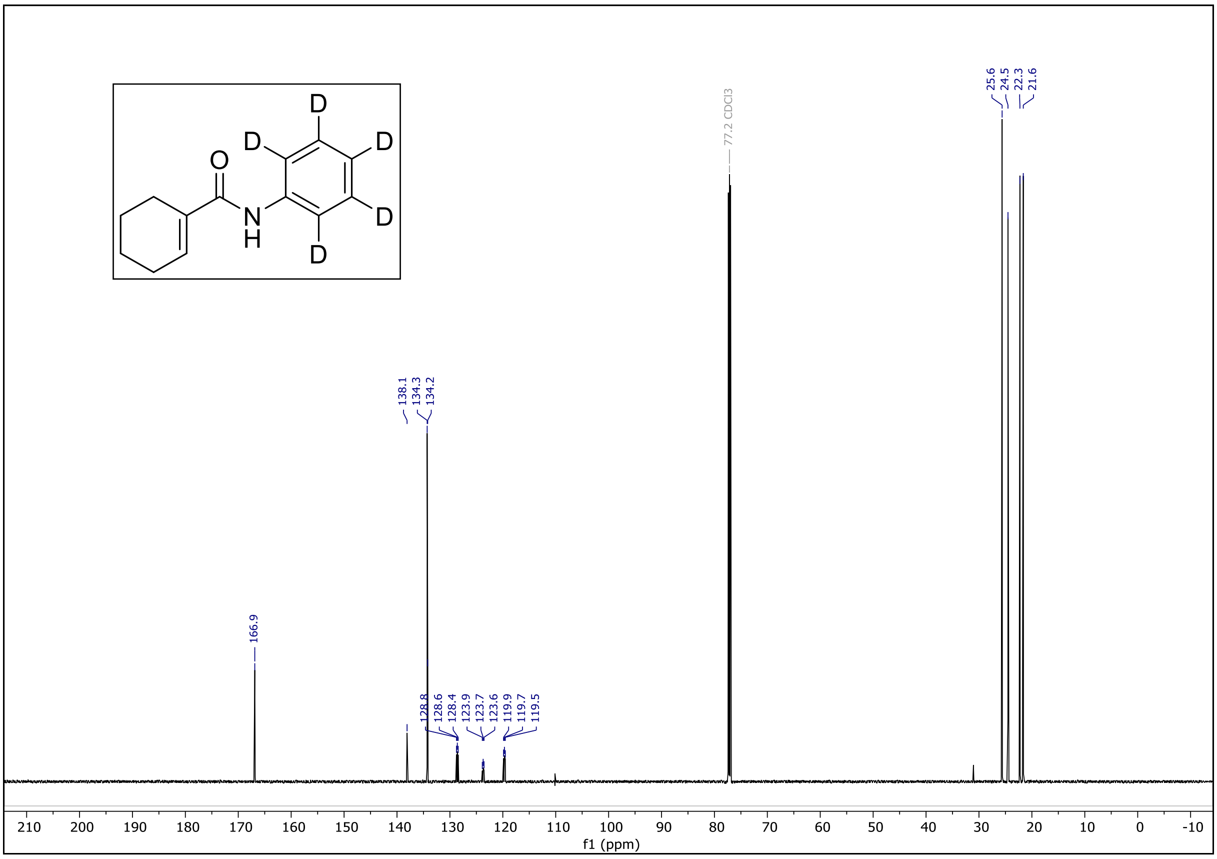

^{1}H NMR (600 MHz, $CDCl_3$): **1w**

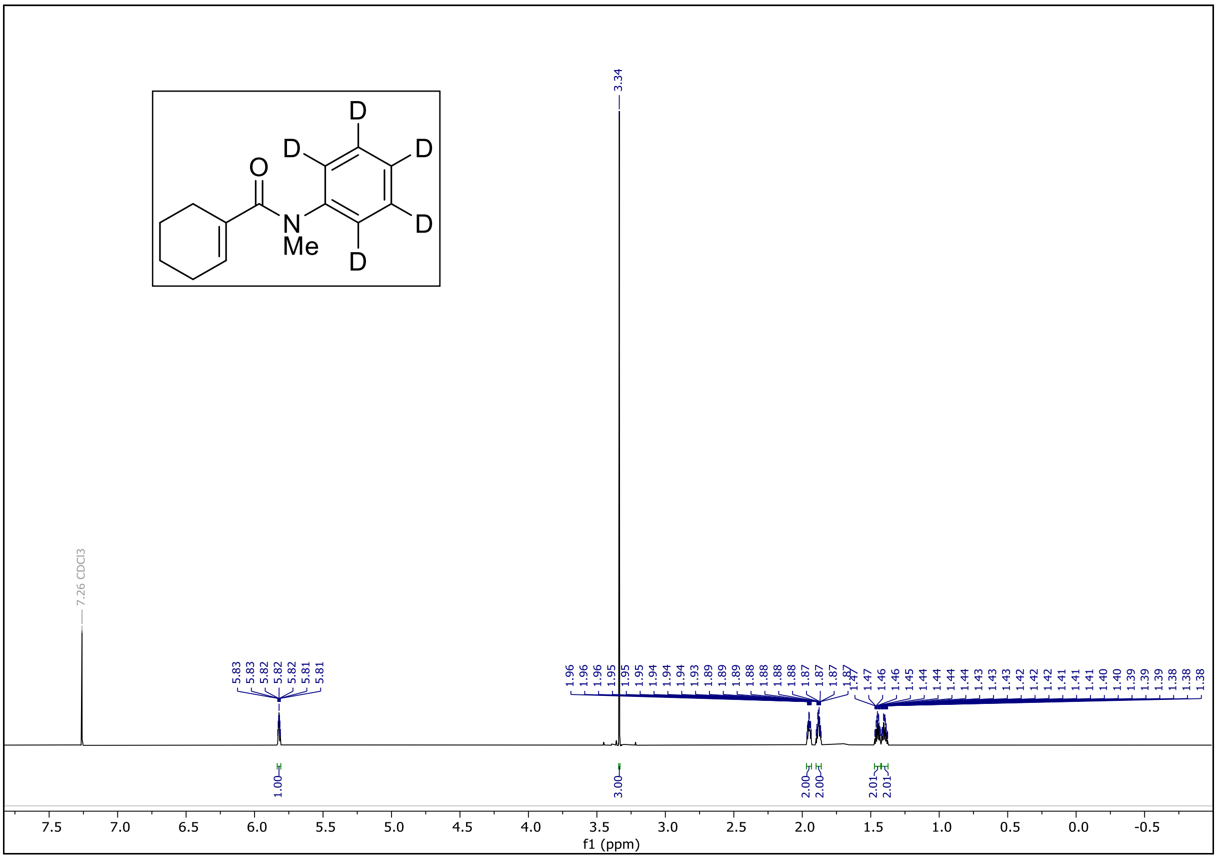

^{13}C NMR (151 MHz, $CDCl_3$): **1w**

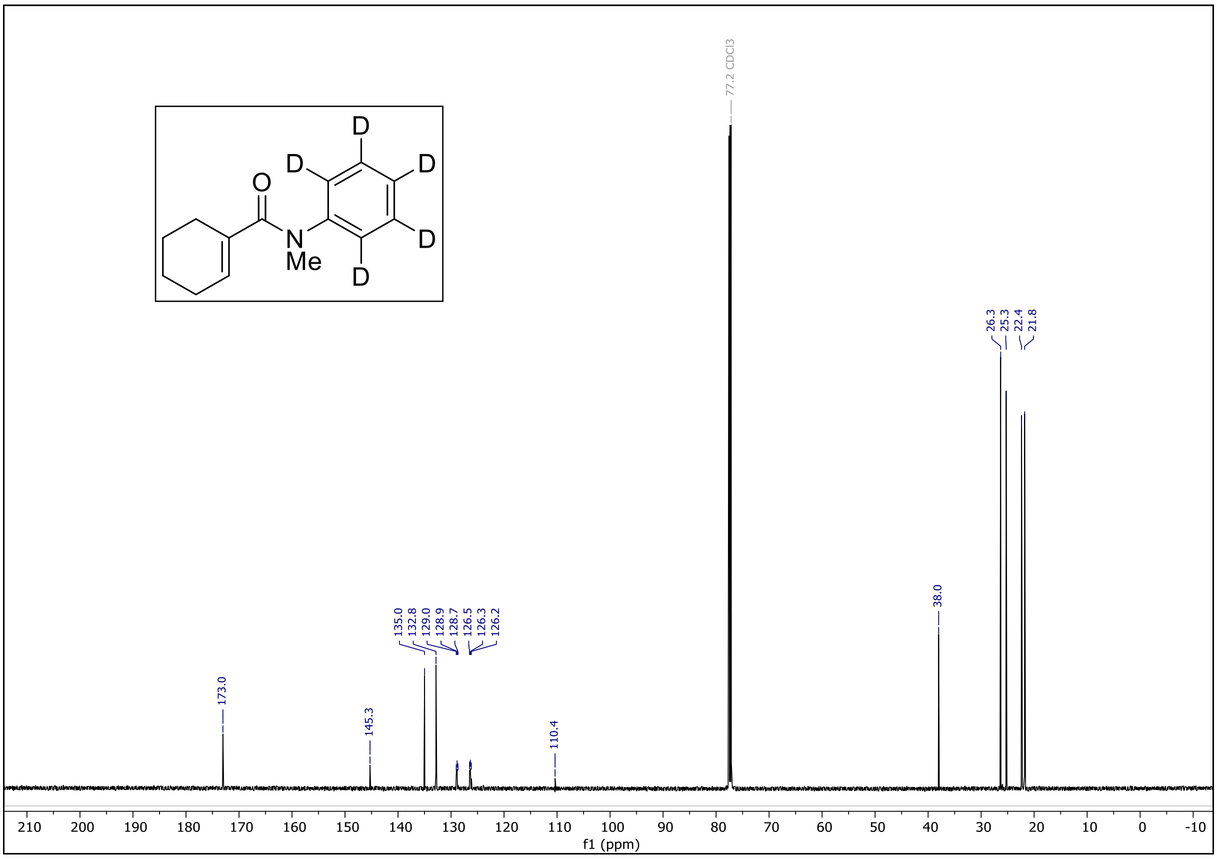

^{1}H NMR (400 MHz, $CDCl_3$): **S32**

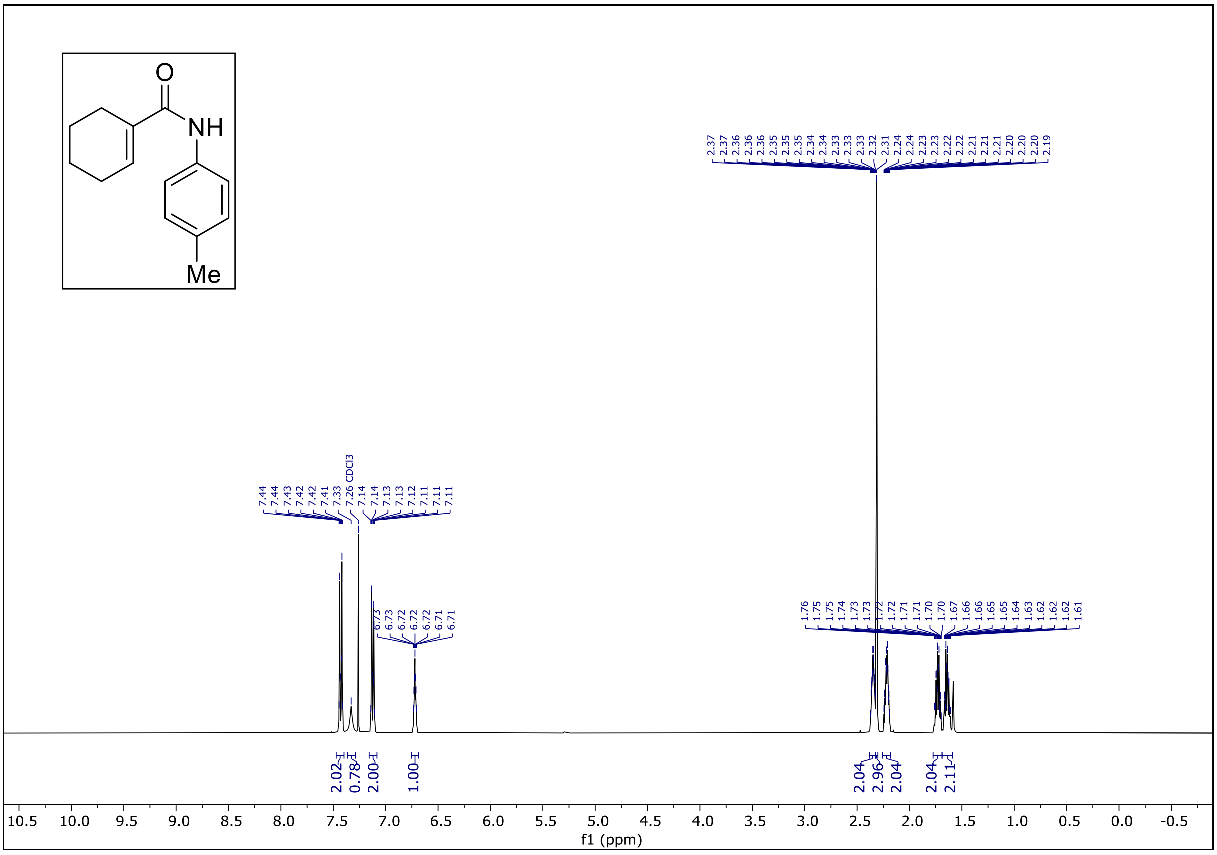

^{1}H NMR (500 MHz, $CDCl_3$): **S33**

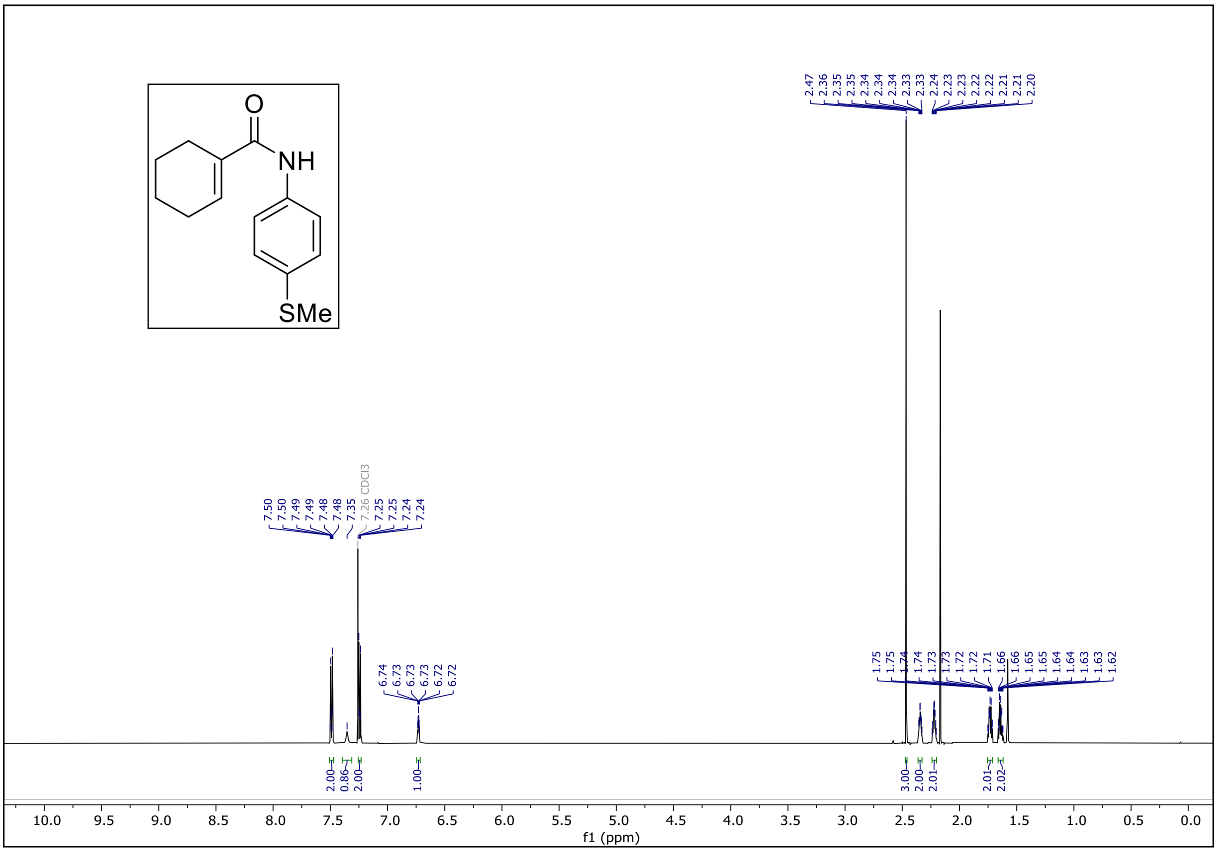

^{13}C NMR (126 MHz, $CDCl_3$): **S33**

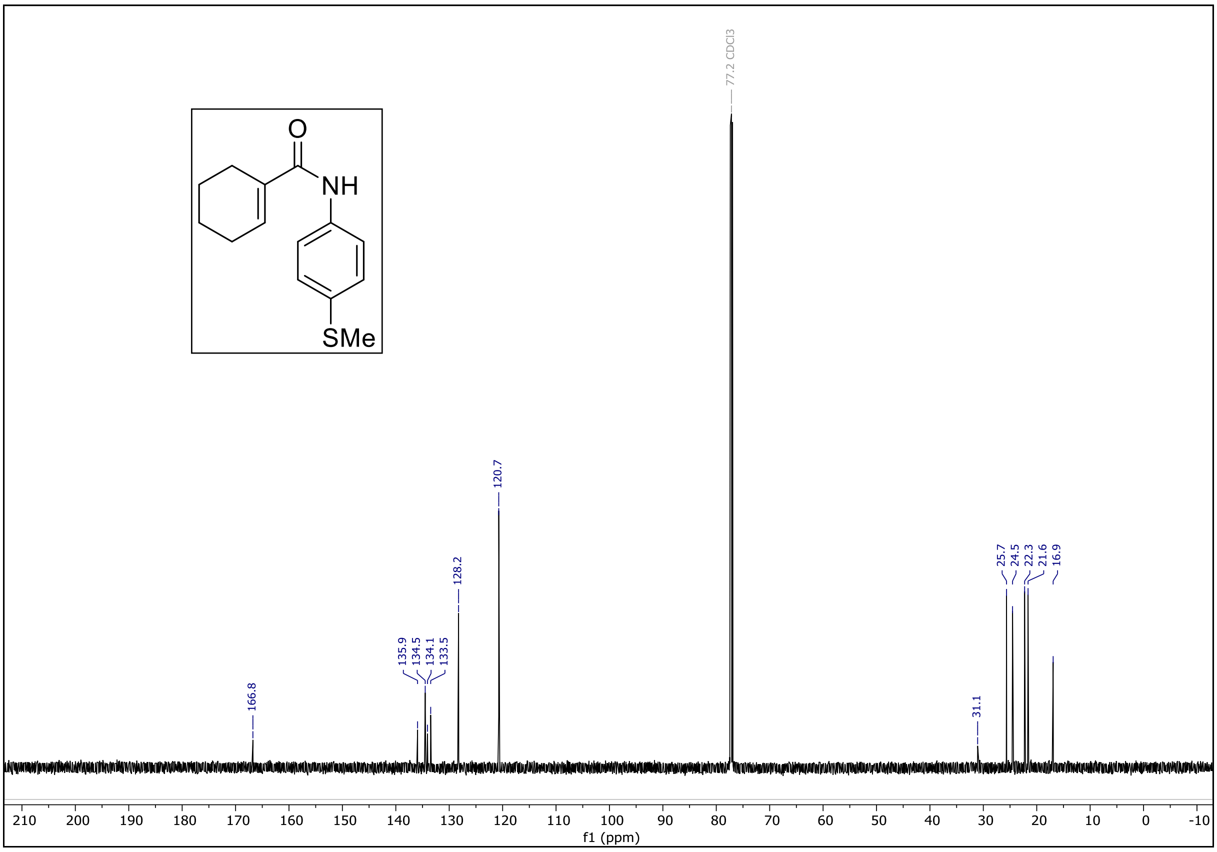

^{1}H NMR (600 MHz, $CDCl_3$): **S34**

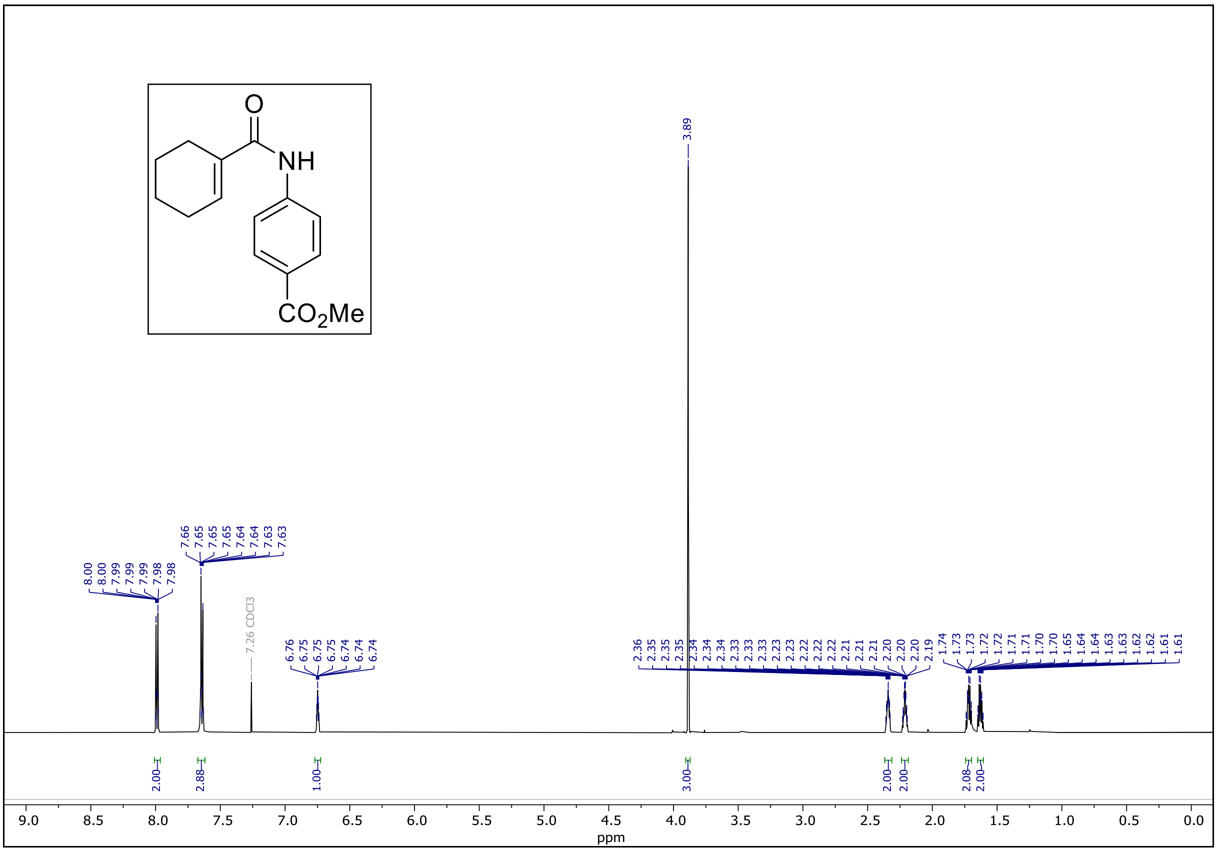

^{13}C NMR (151 MHz, $CDCl_3$): **S34**

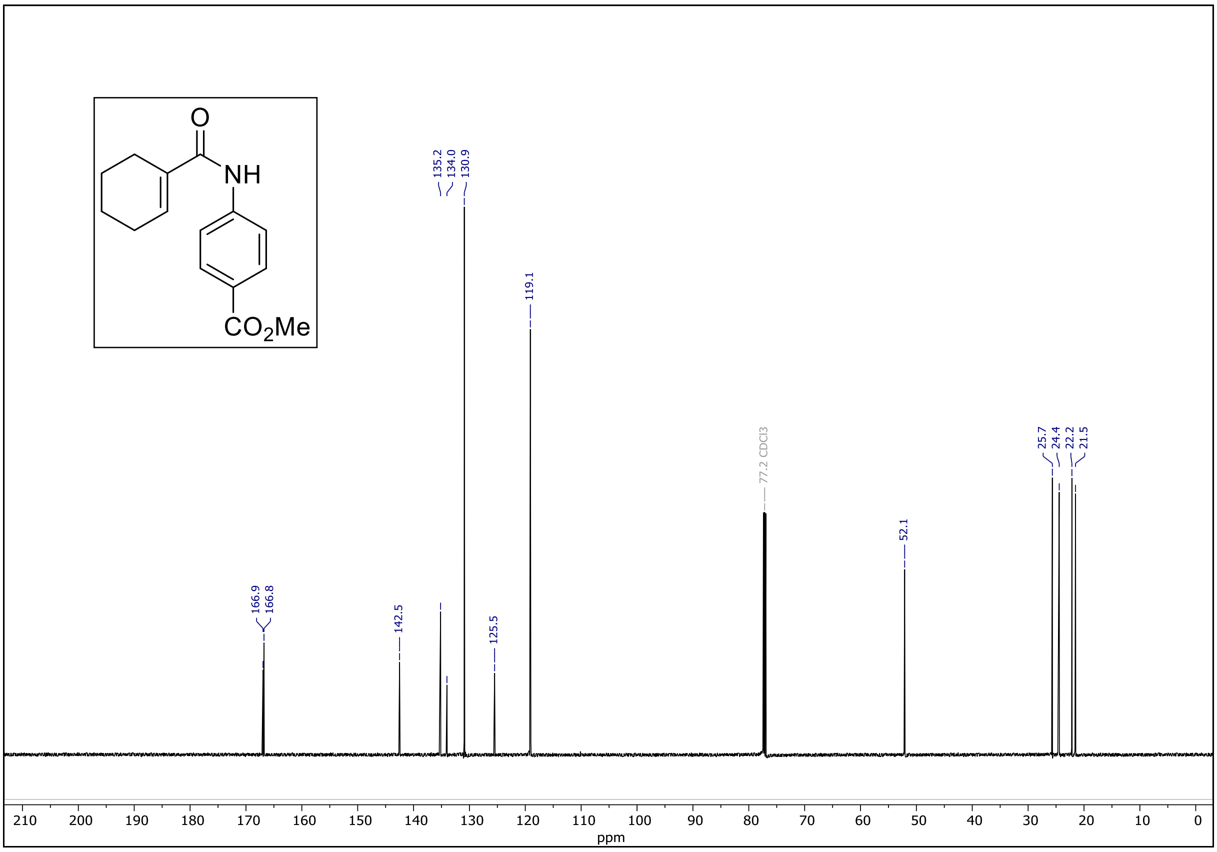

^1H NMR {^{19}F} (500 MHz, $CDCl_3$): **S35**

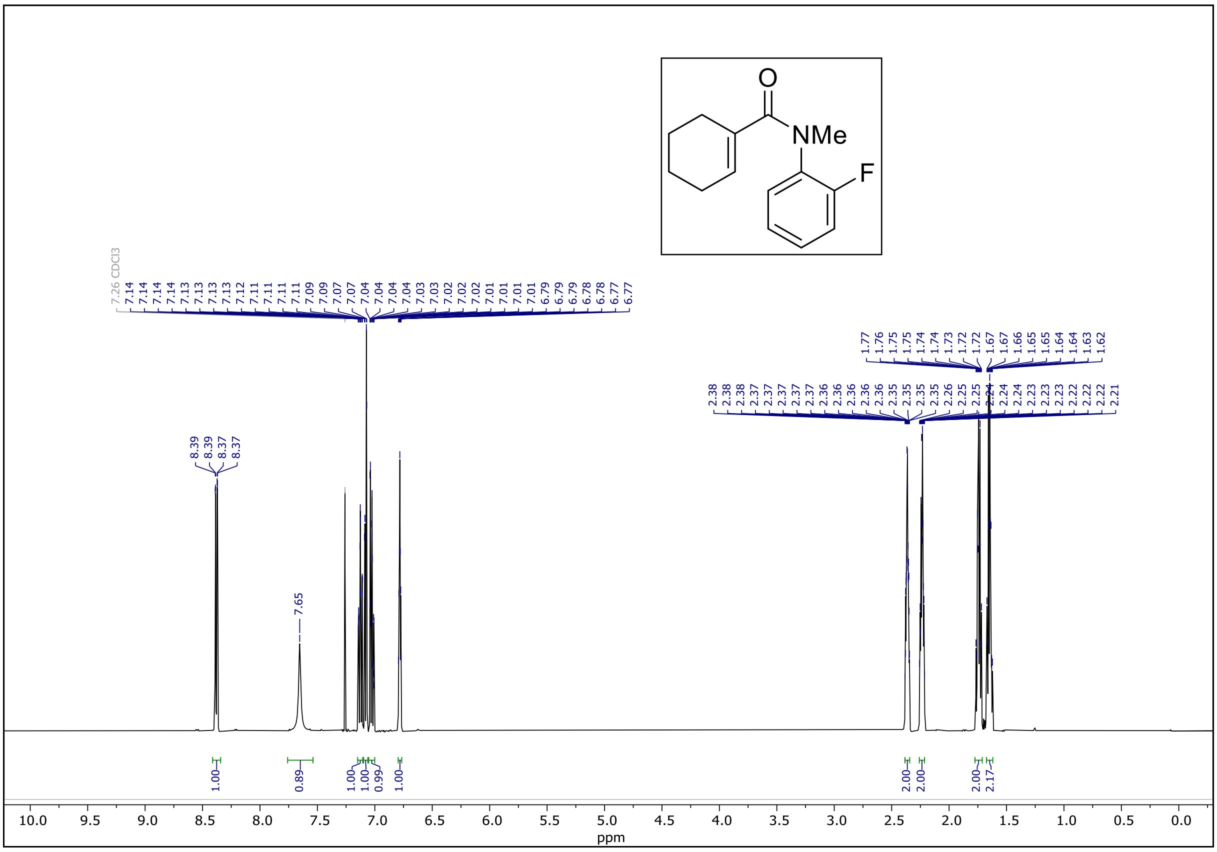

^{13}C NMR (126 MHz, $CDCl_3$): **S35**

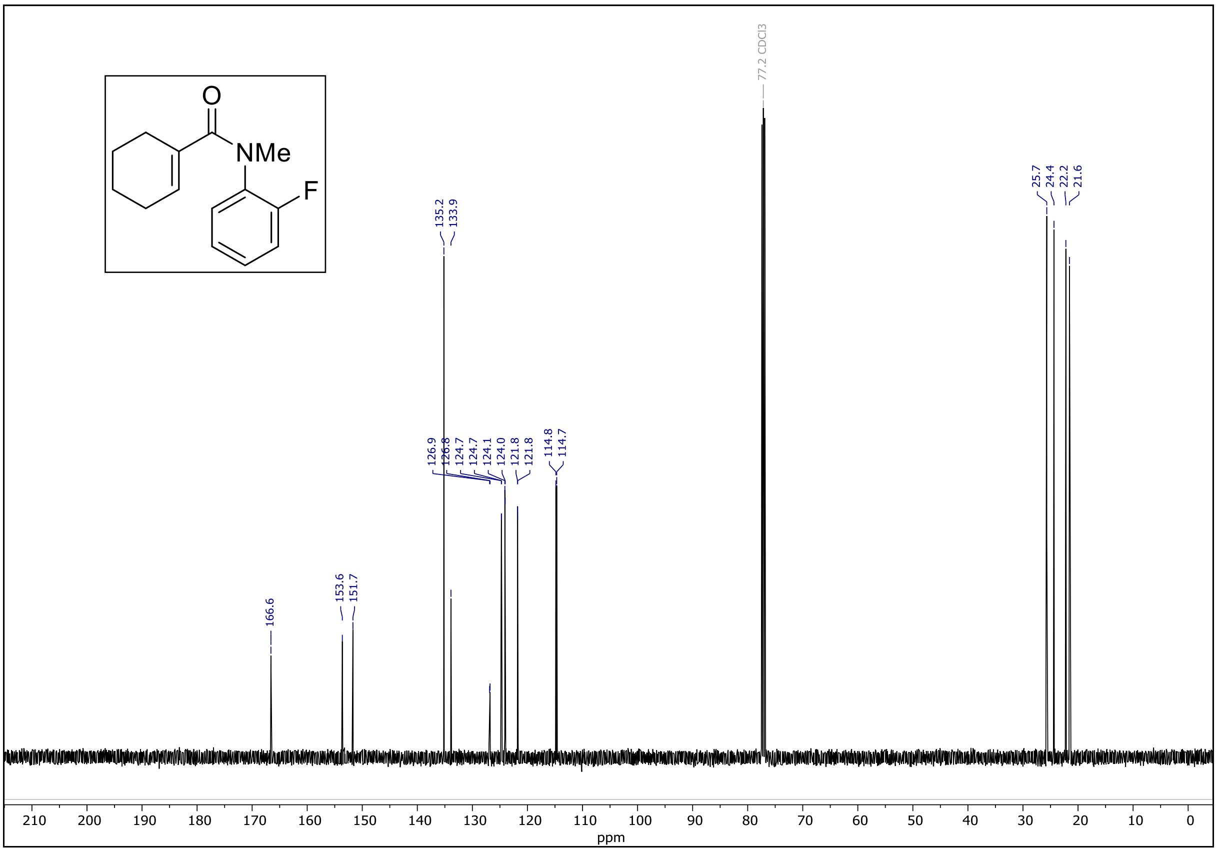

^{19}F NMR {^{1}H} (470 MHz, $CDCl_3$): **S35**

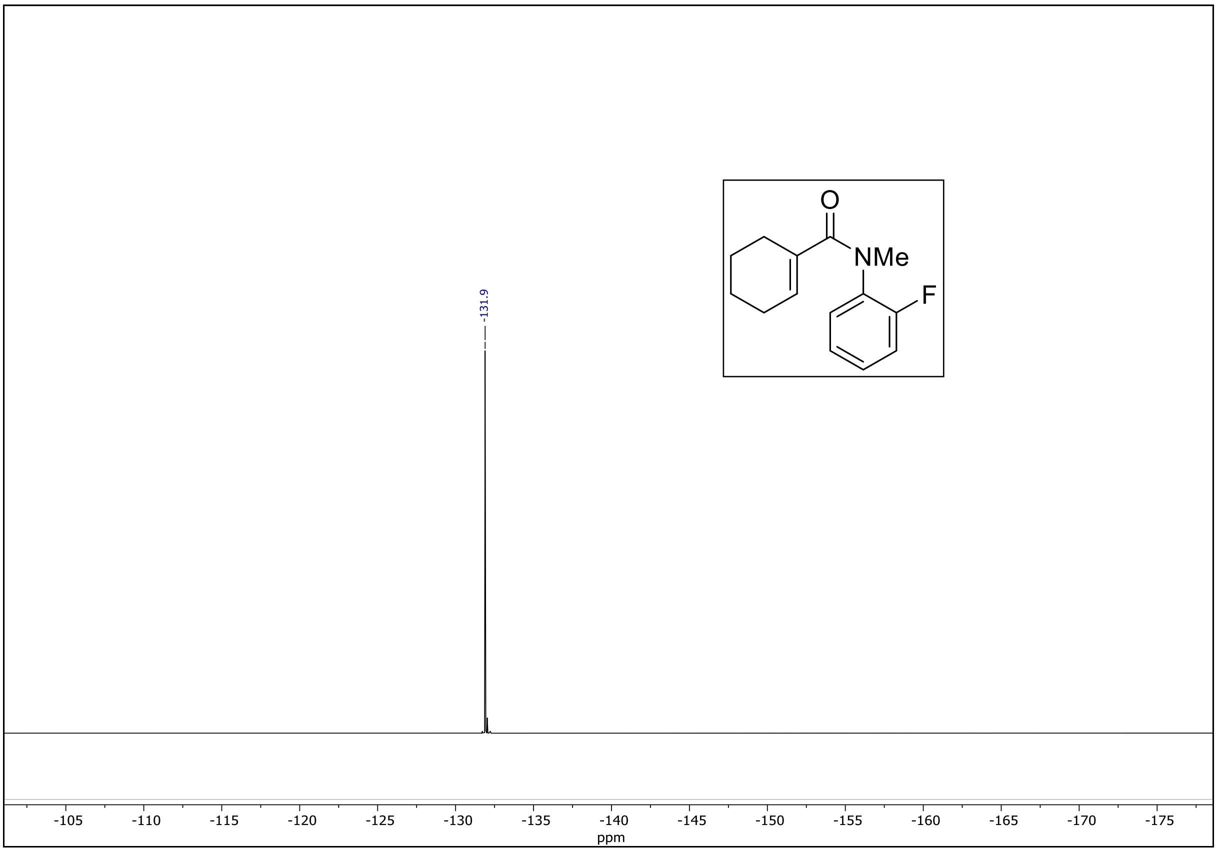

^{1}H NMR (500 MHz, $CDCl_3$): **S36**

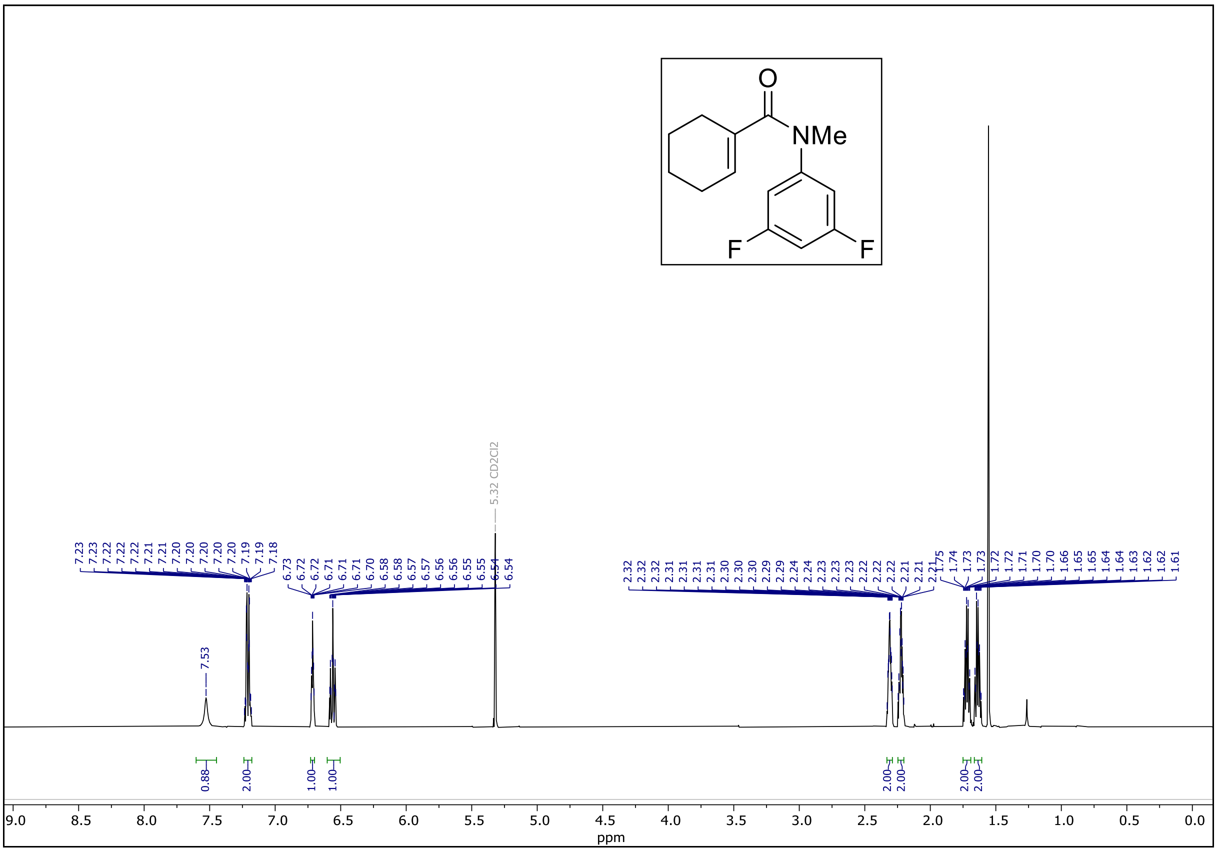

^{13}C NMR {^{19}F} (126 MHz, $CDCl_3$): **S36**

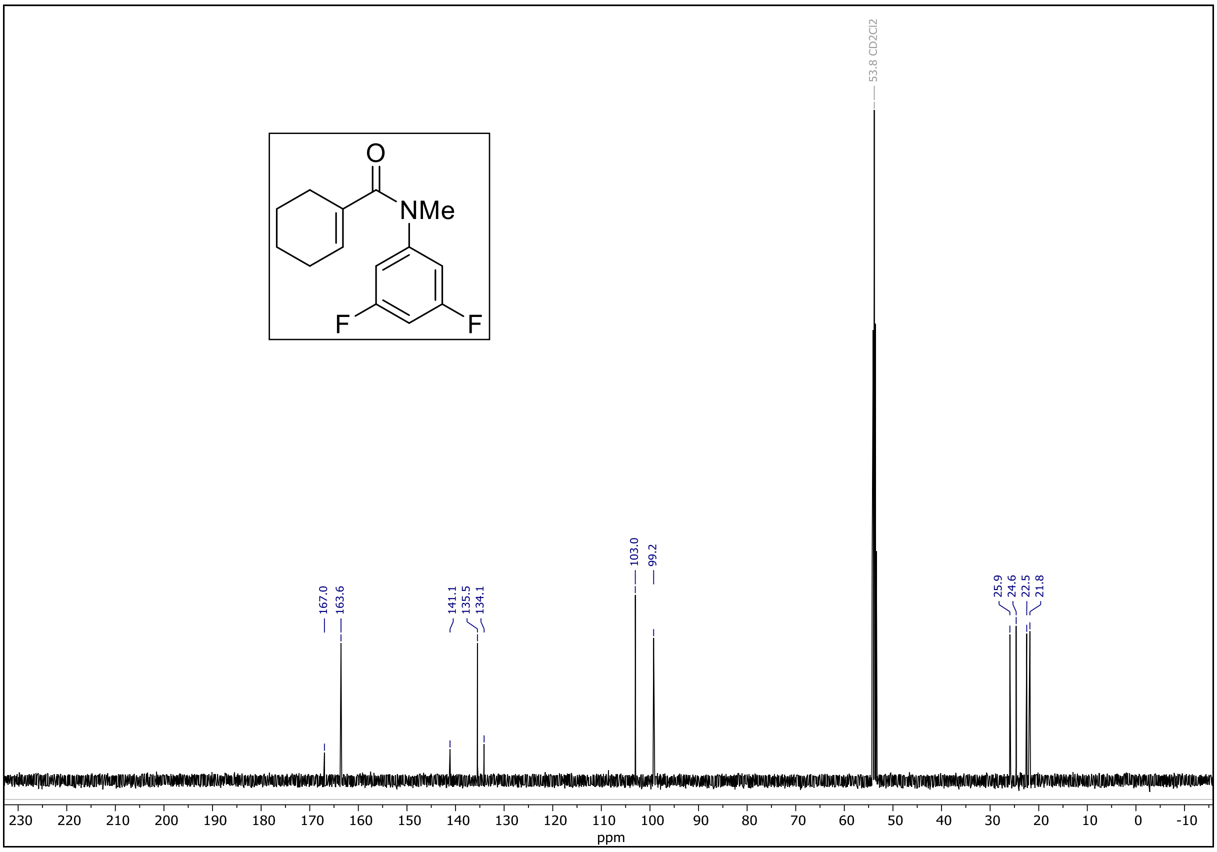

^{19}F NMR {^{1}H} (470 MHz, $CDCl_3$): **S36**

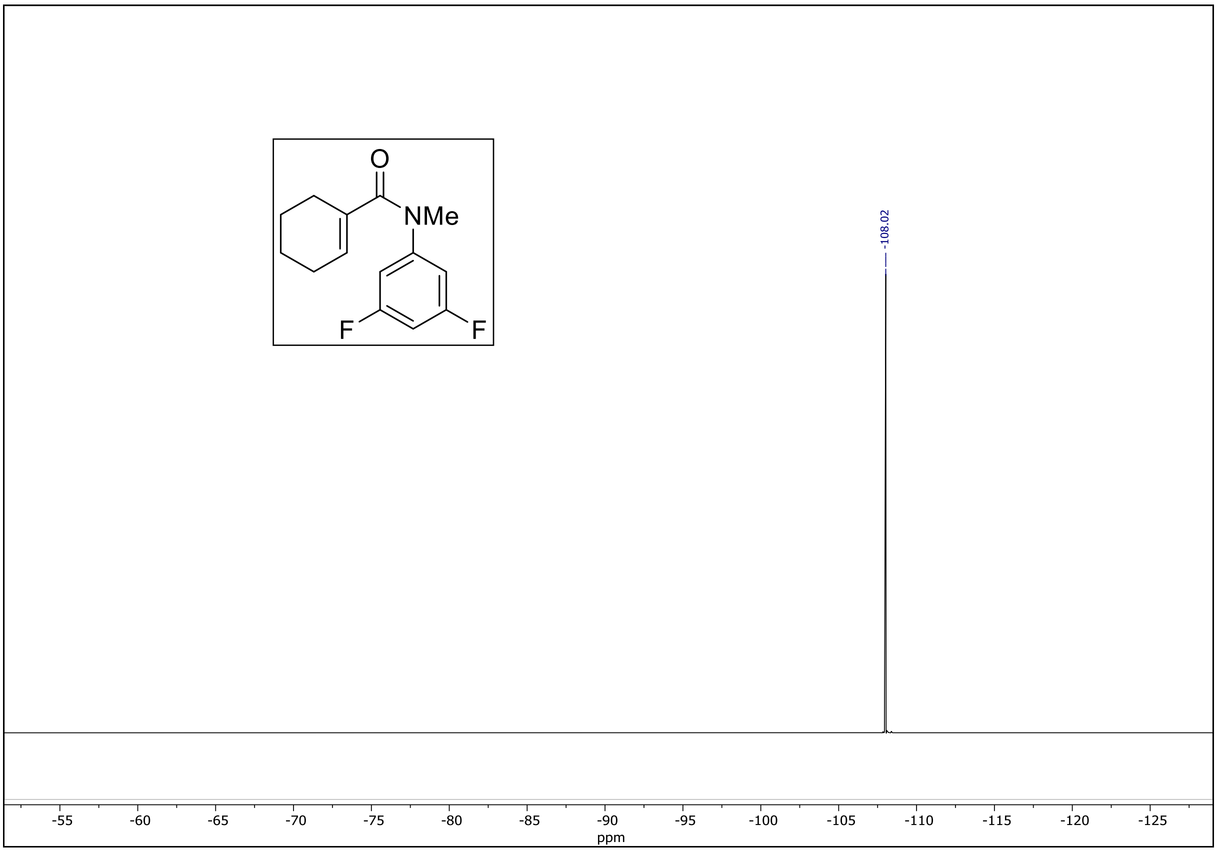

^{1}H NMR (600 MHz, $CDCl_3$): **S37**

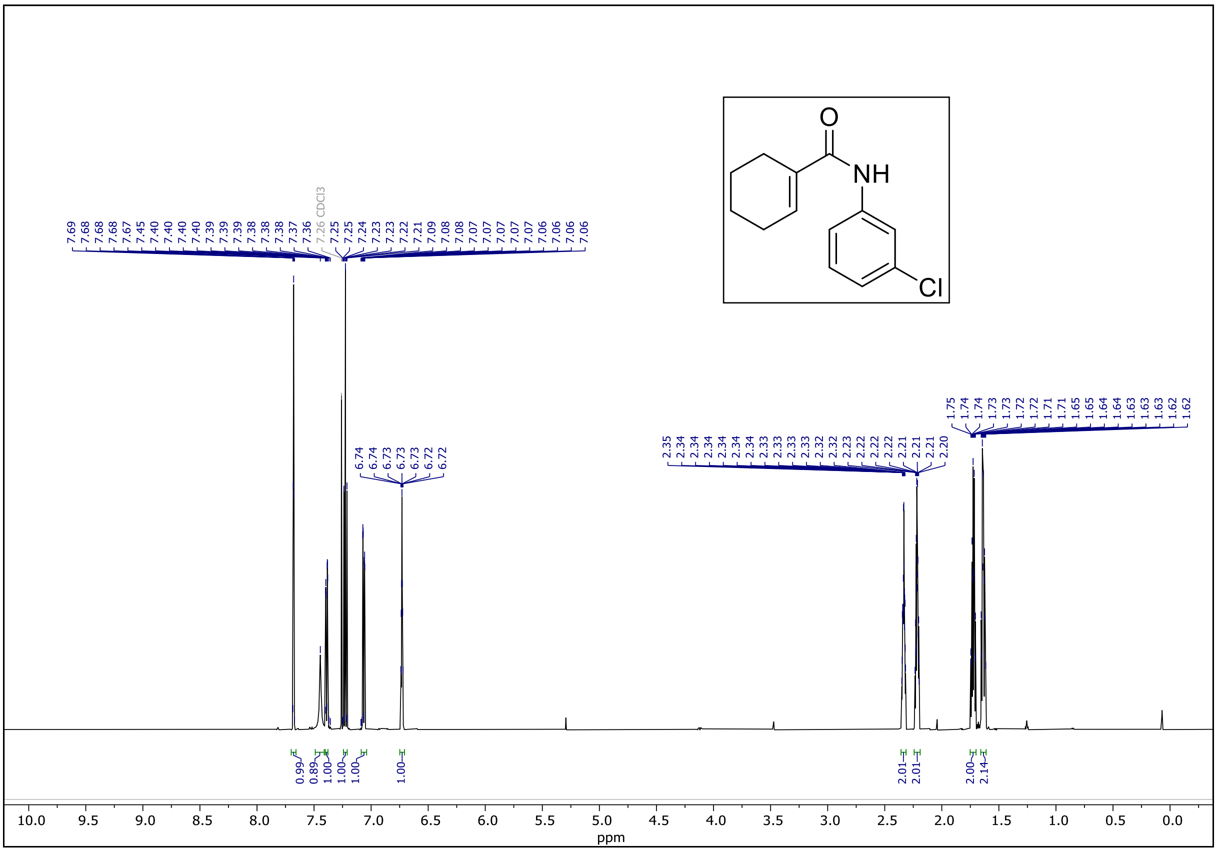

^{13}C NMR (151 MHz, $CDCl_3$): **S37**

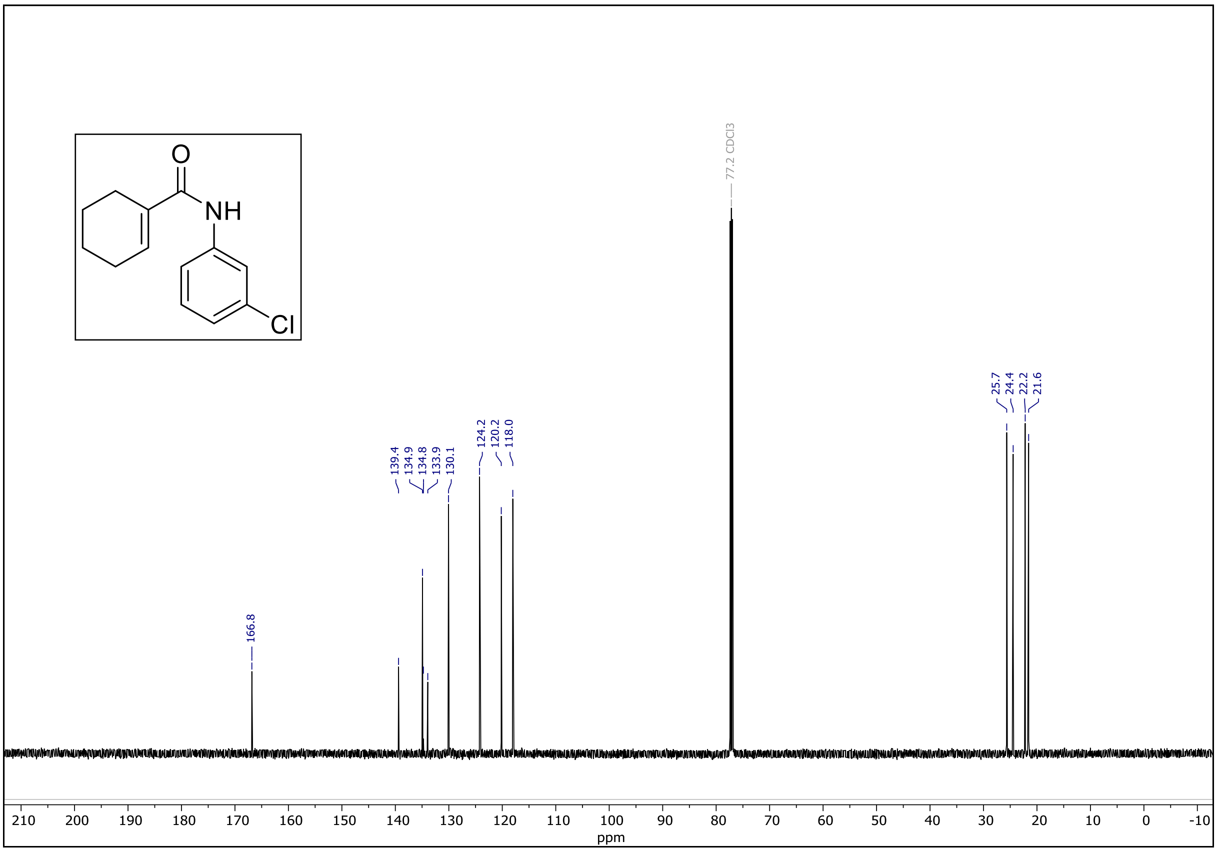

^{1}H NMR (600 MHz, $CDCl_3$): **S38**

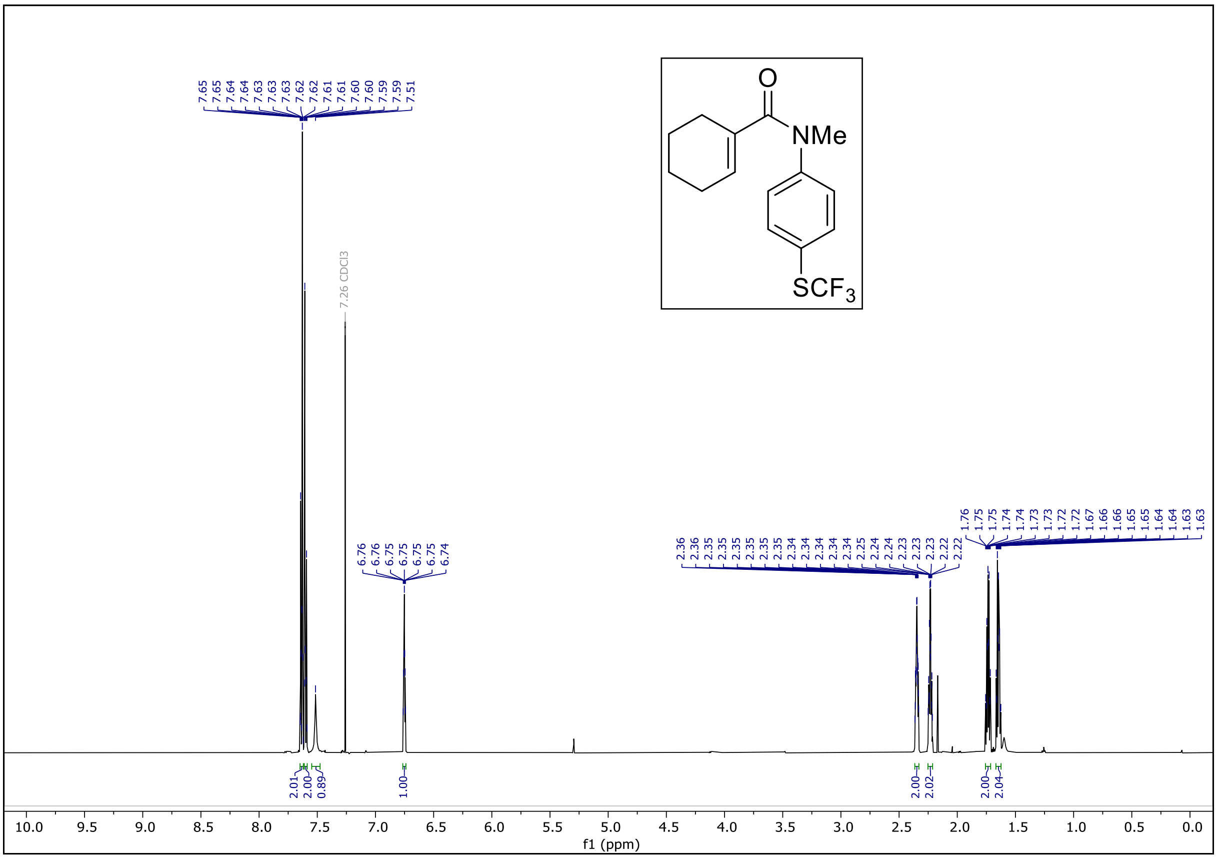

^{13}C NMR {^{19}F} (151 MHz, $CDCl_3$): **S38**

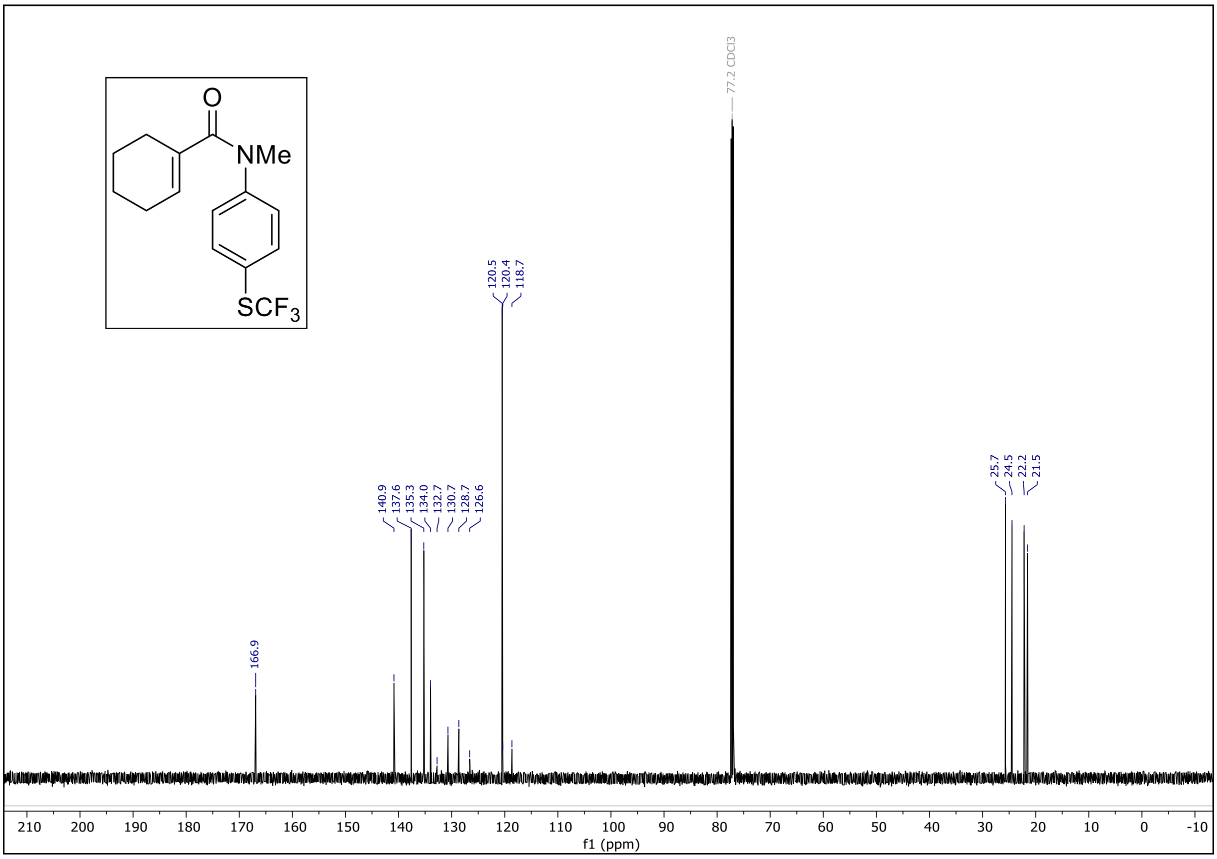

^{19}F NMR {^{1}H} (564 MHz, $CDCl_3$): **S38**

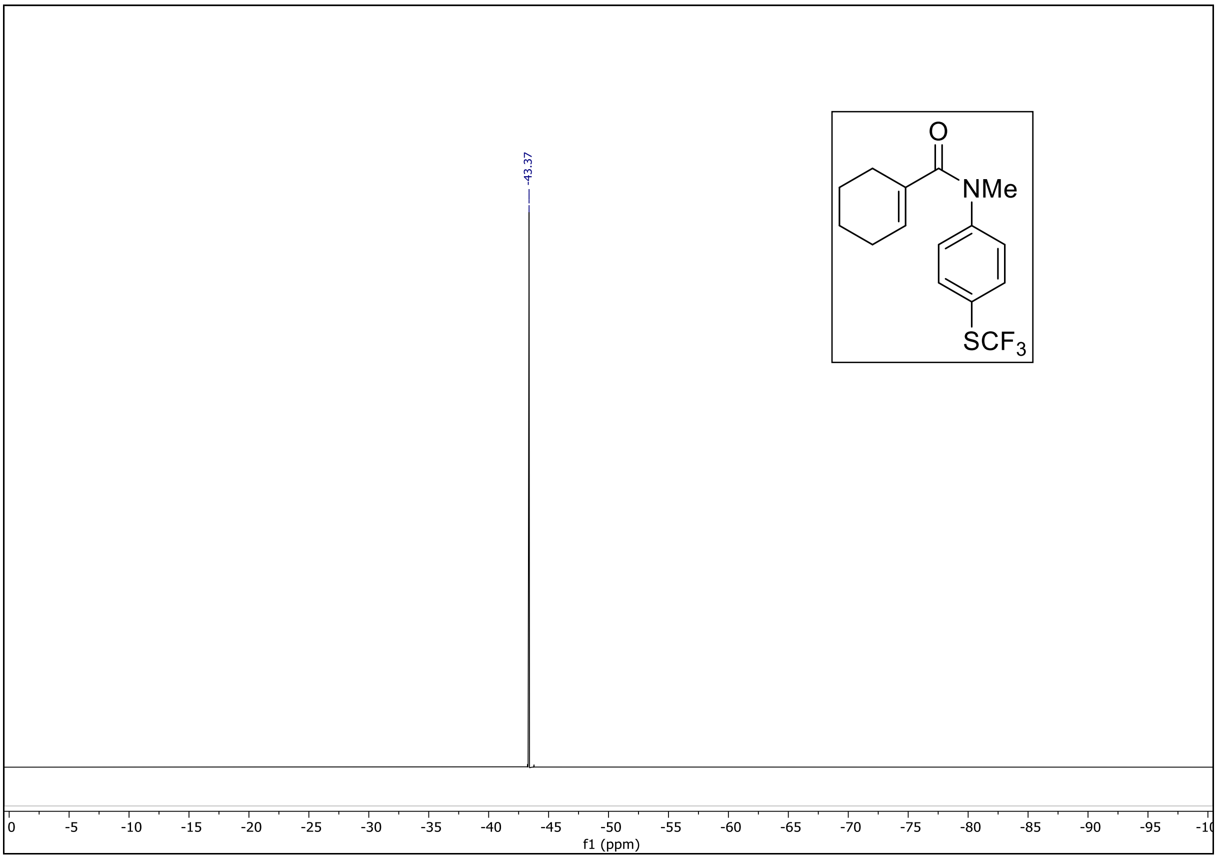

^{1}H NMR (500 MHz, $CDCl_3$): **S39**

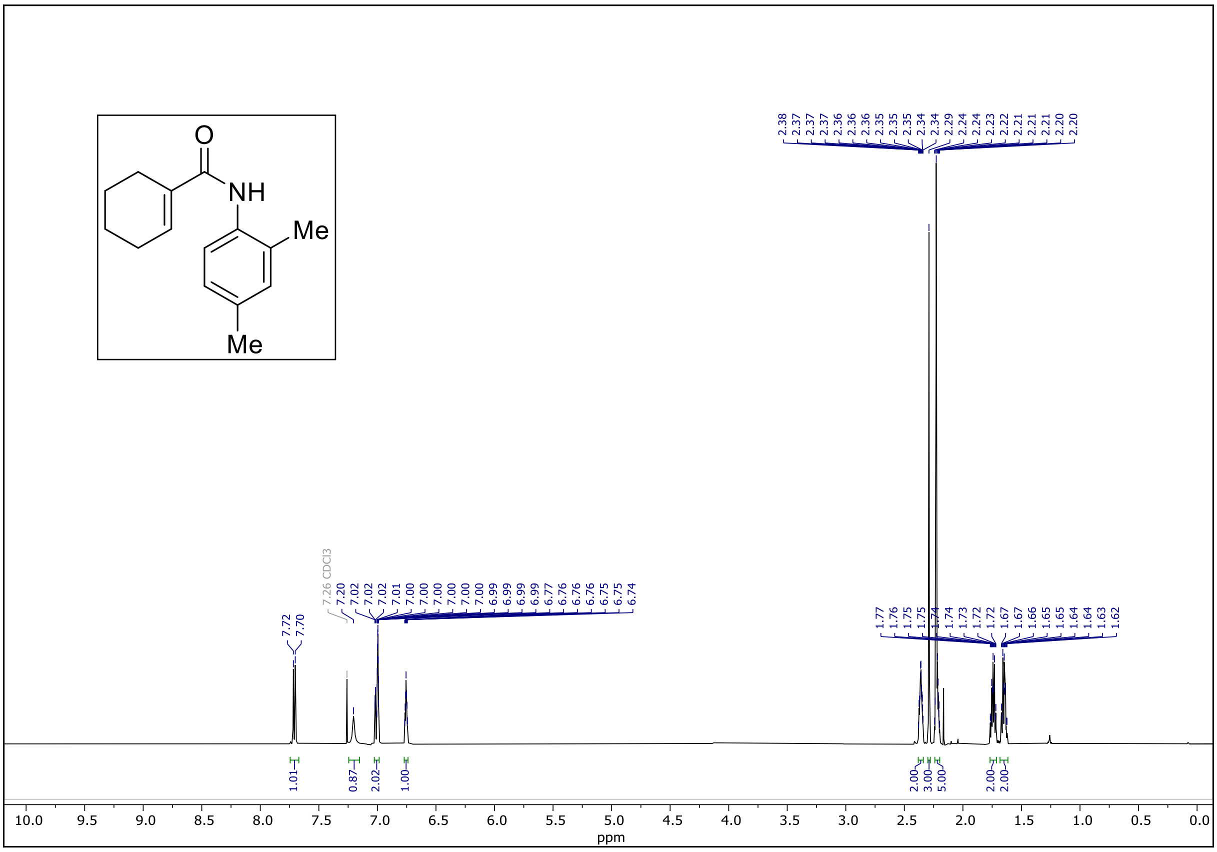

^{13}C NMR (126 MHz, $CDCl_3$): **S39**

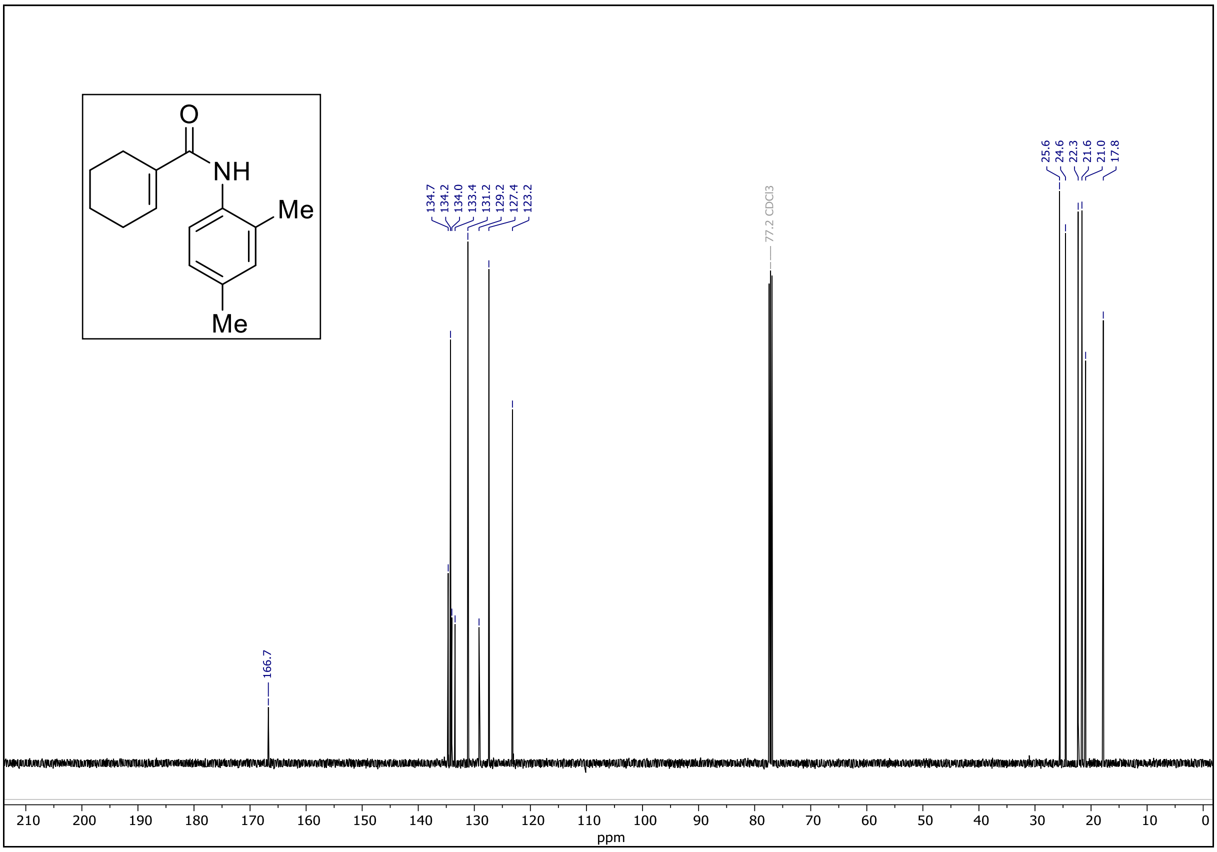

^{1}H NMR (400 MHz, $CDCl_3$): **S40**

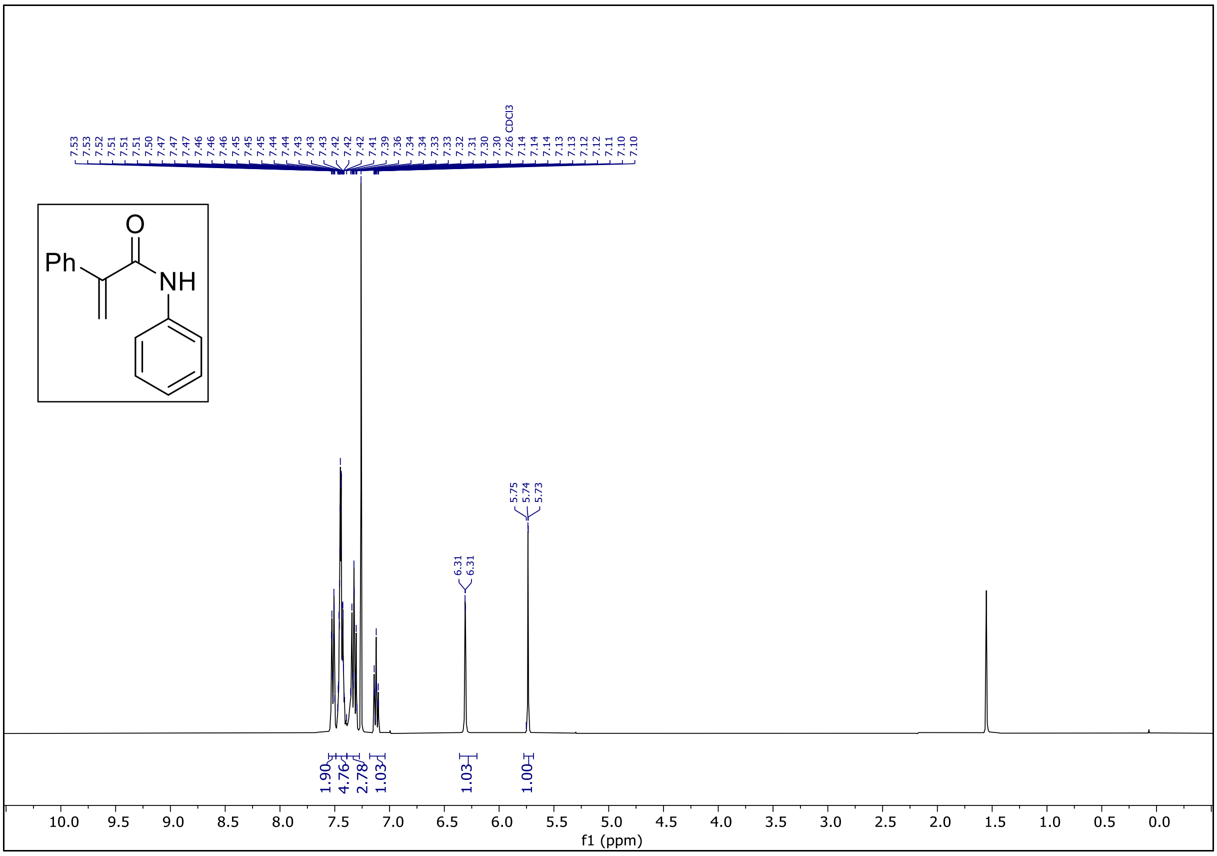

^{1}H NMR (400 MHz, $CDCl_3$): **S41**

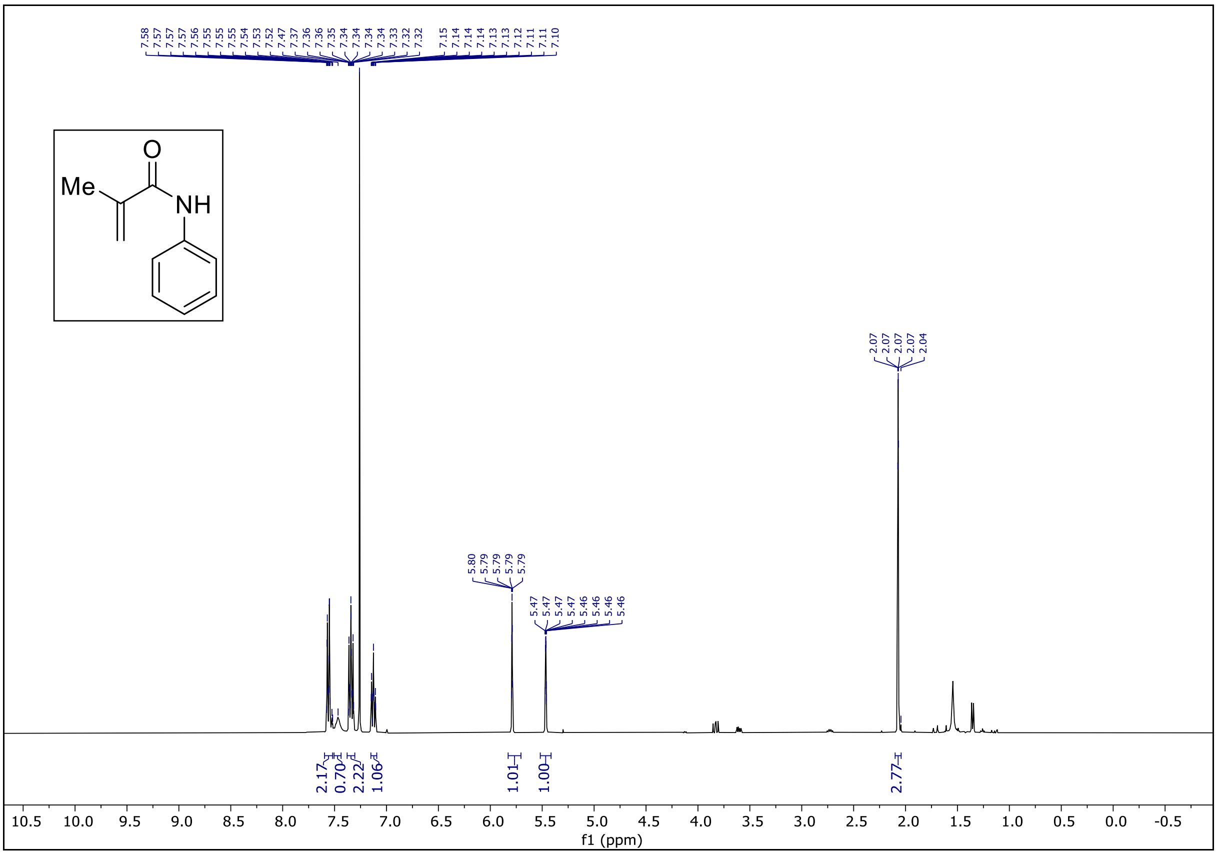

^{1}H NMR (500 MHz, $CDCl_3$): **S13**

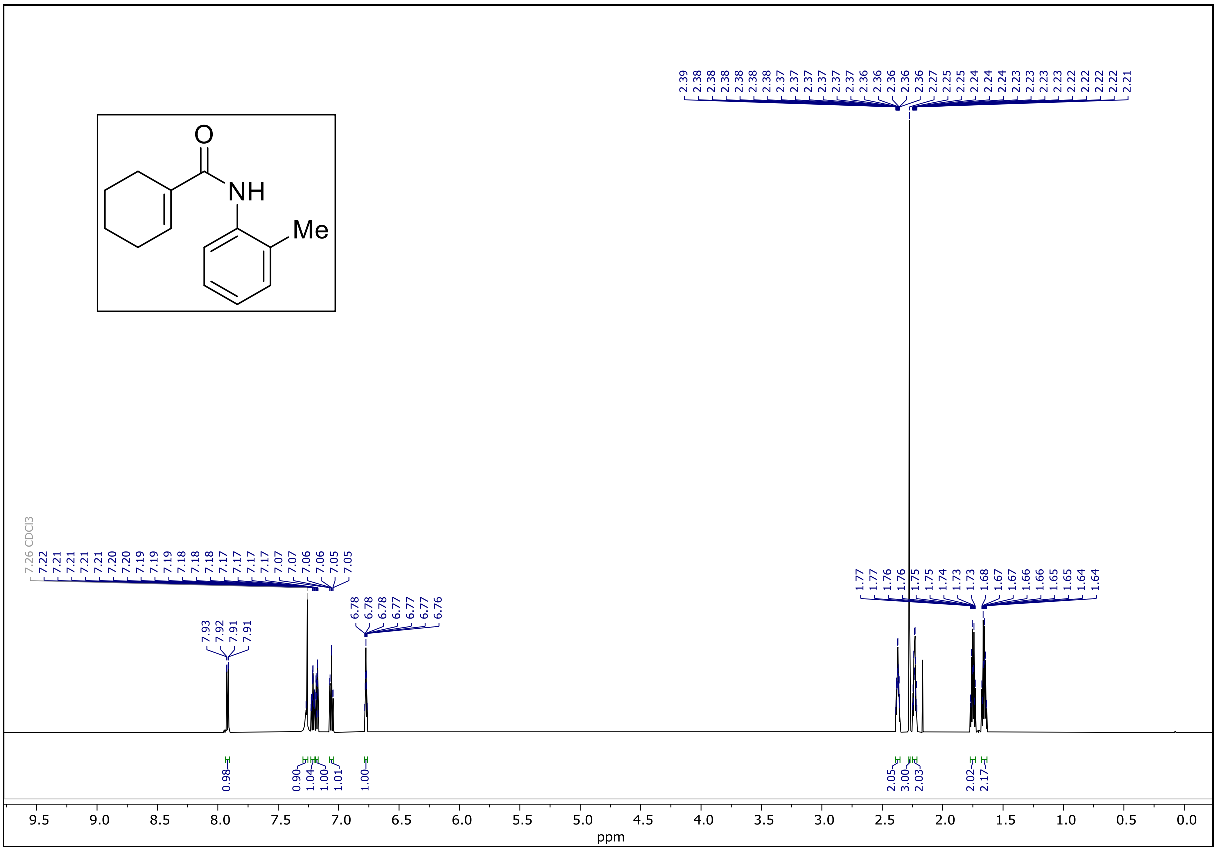

^{13}C NMR (126 MHz, $CDCl_3$): **S13**

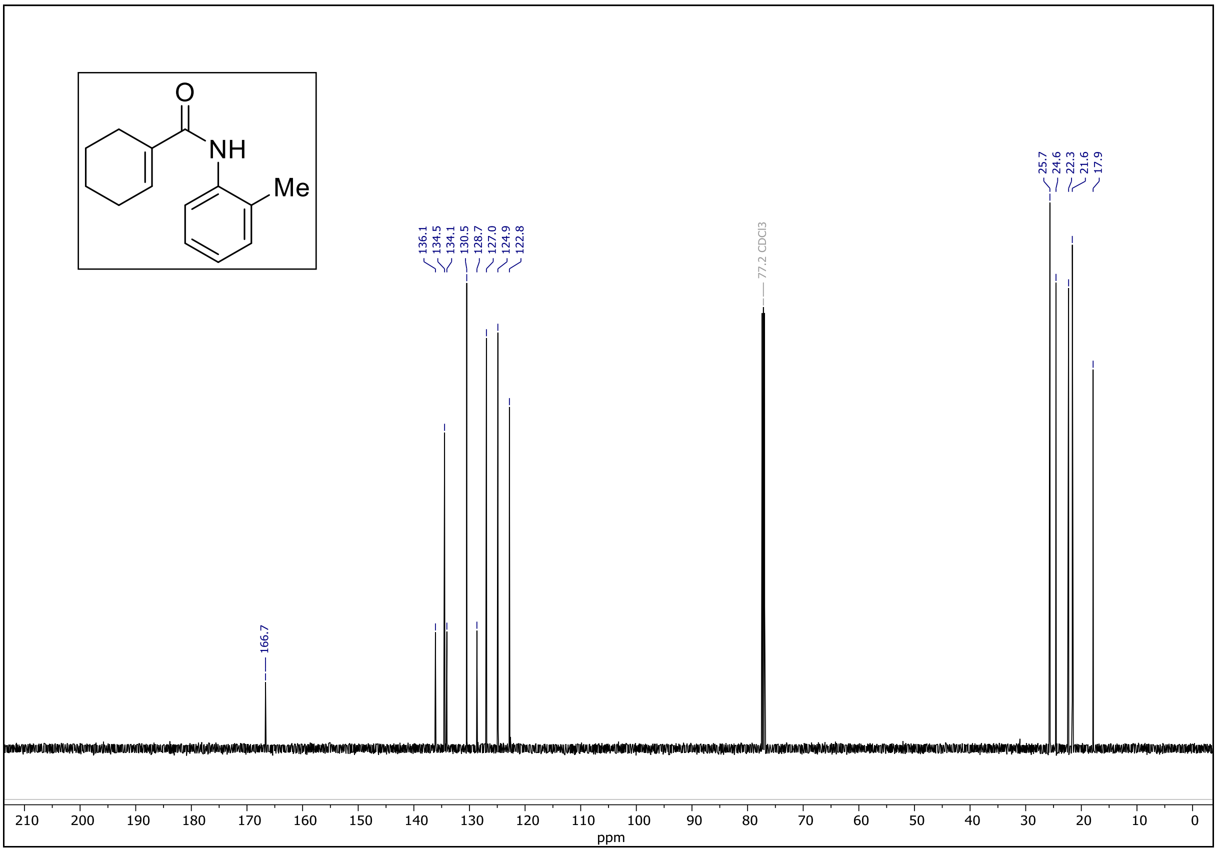

^{1}H NMR (400 MHz, $CDCl_3$): **S14**

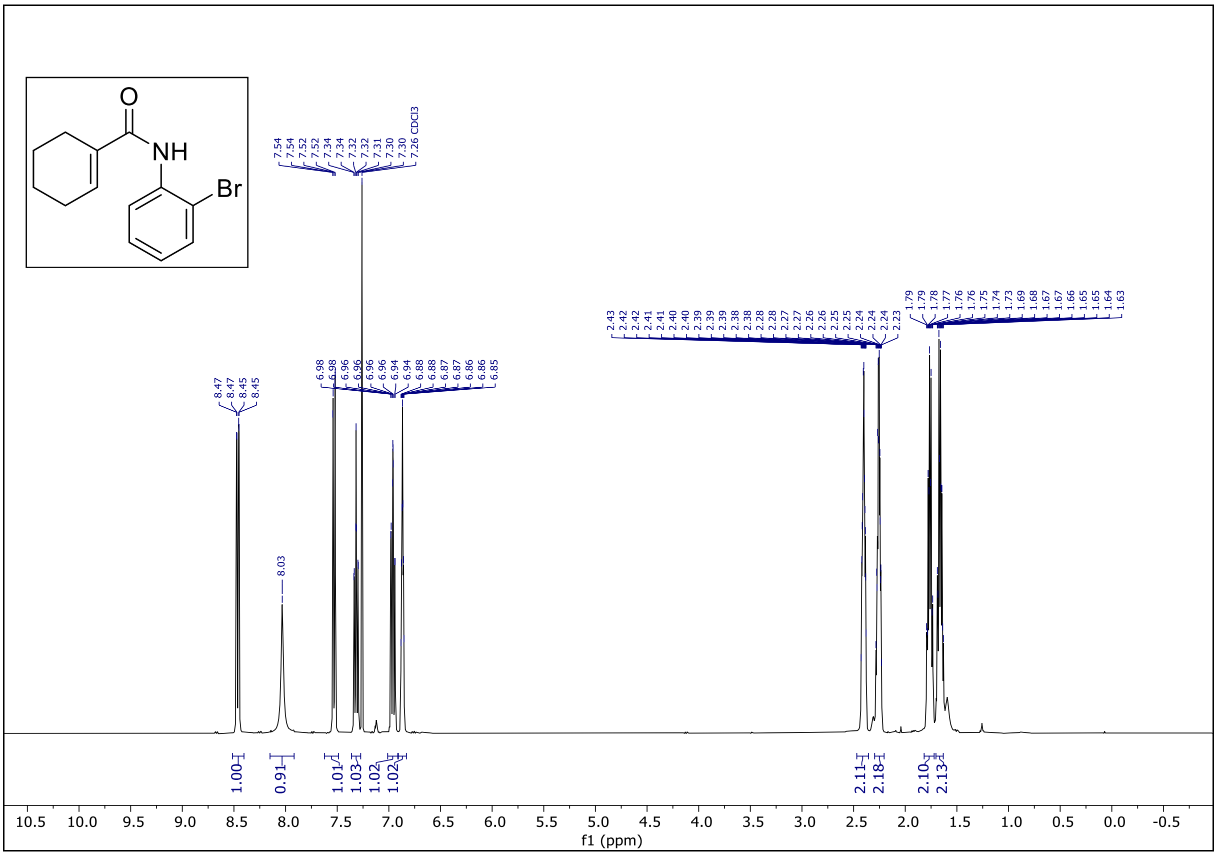

^1H NMR (400 MHz, $CDCl_3$): **S15**

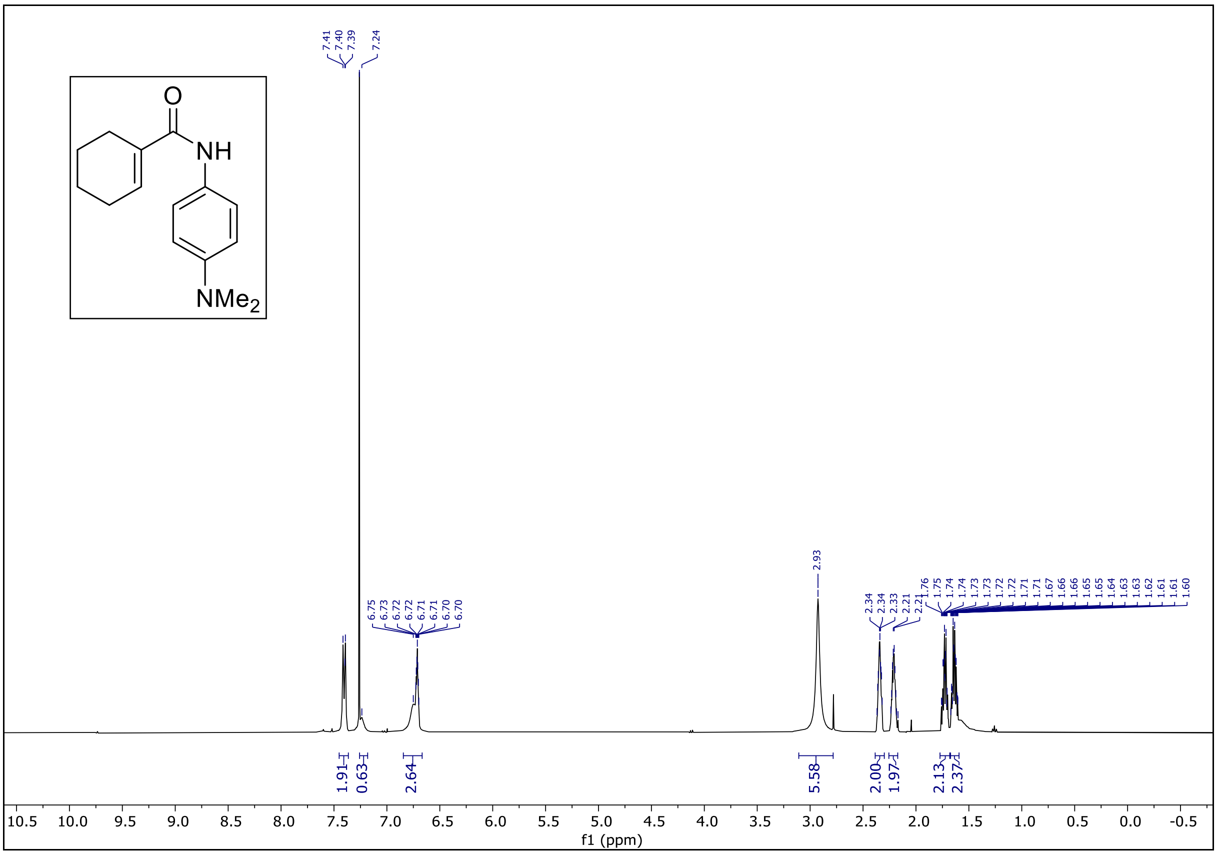

^1H NMR (400 MHz, $CDCl_3$): **S16**

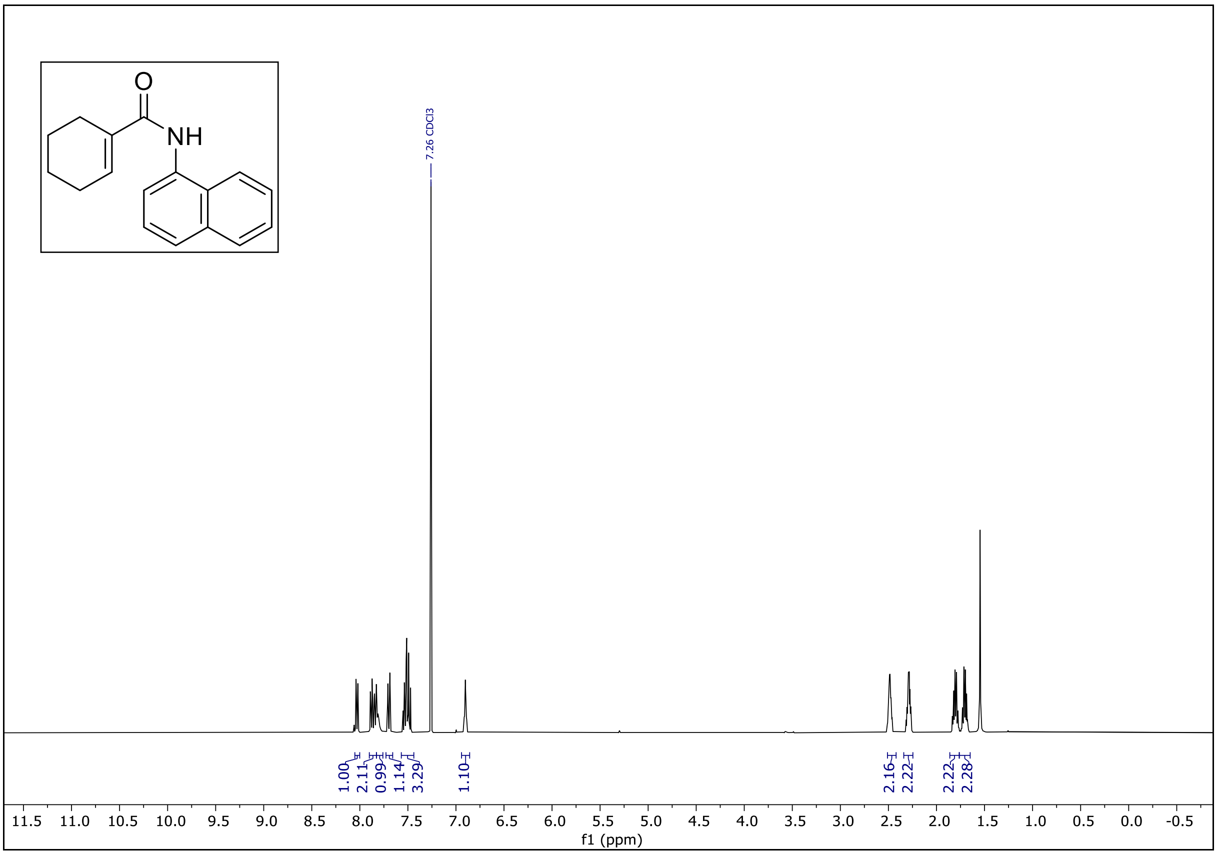

^{1}H NMR (600 MHz, $CDCl_3$): **S17**

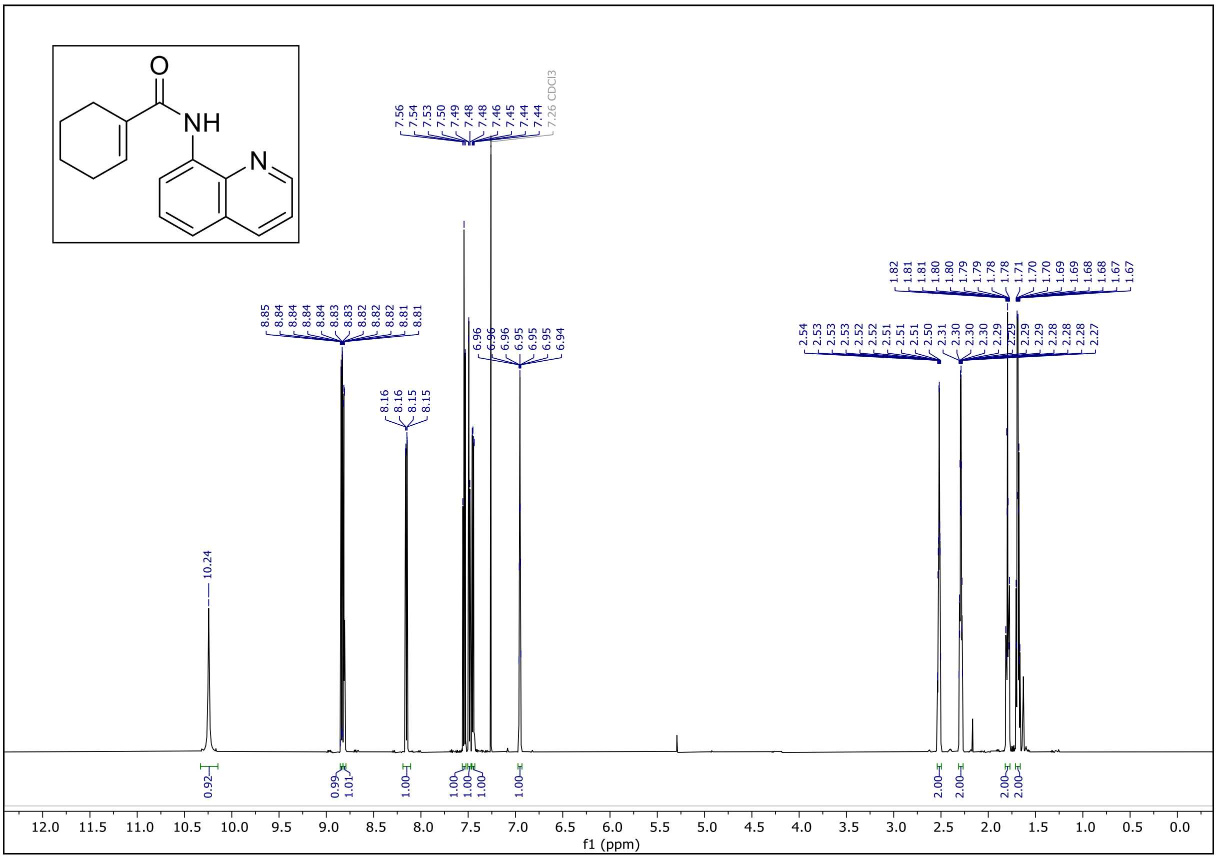

^{13}C NMR (151 MHz, $CDCl_3$): **S17**

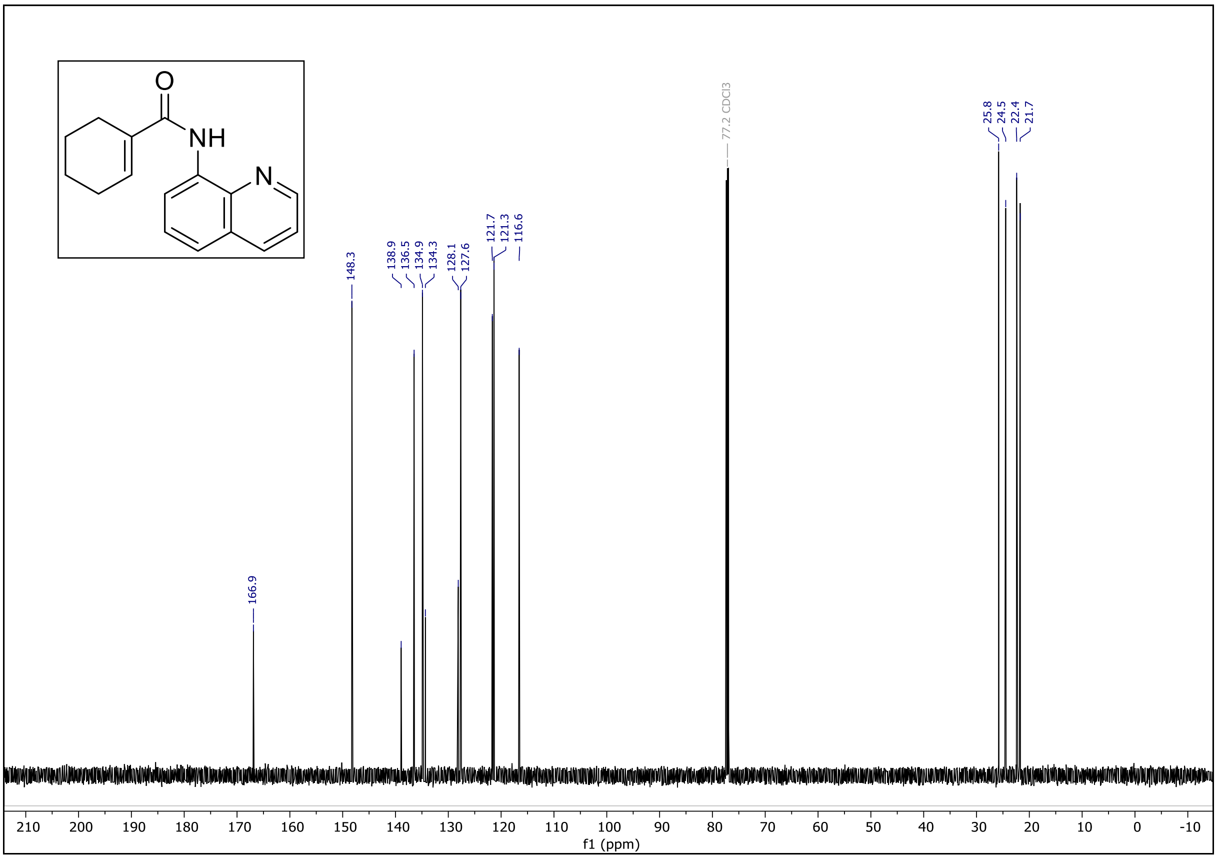

^1H NMR (400 MHz, $CDCl_3$): **S18**

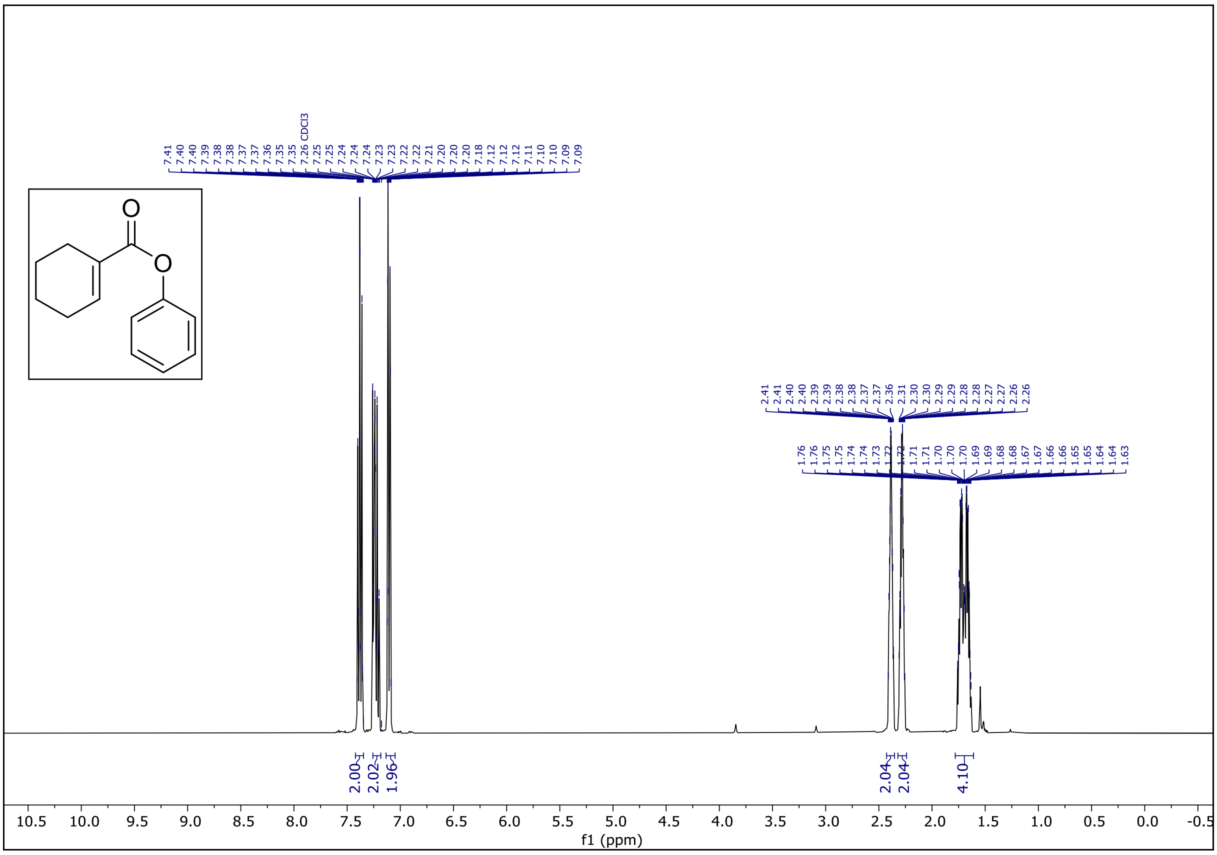

^1H NMR (400 MHz, $CDCl_3$): **S19**

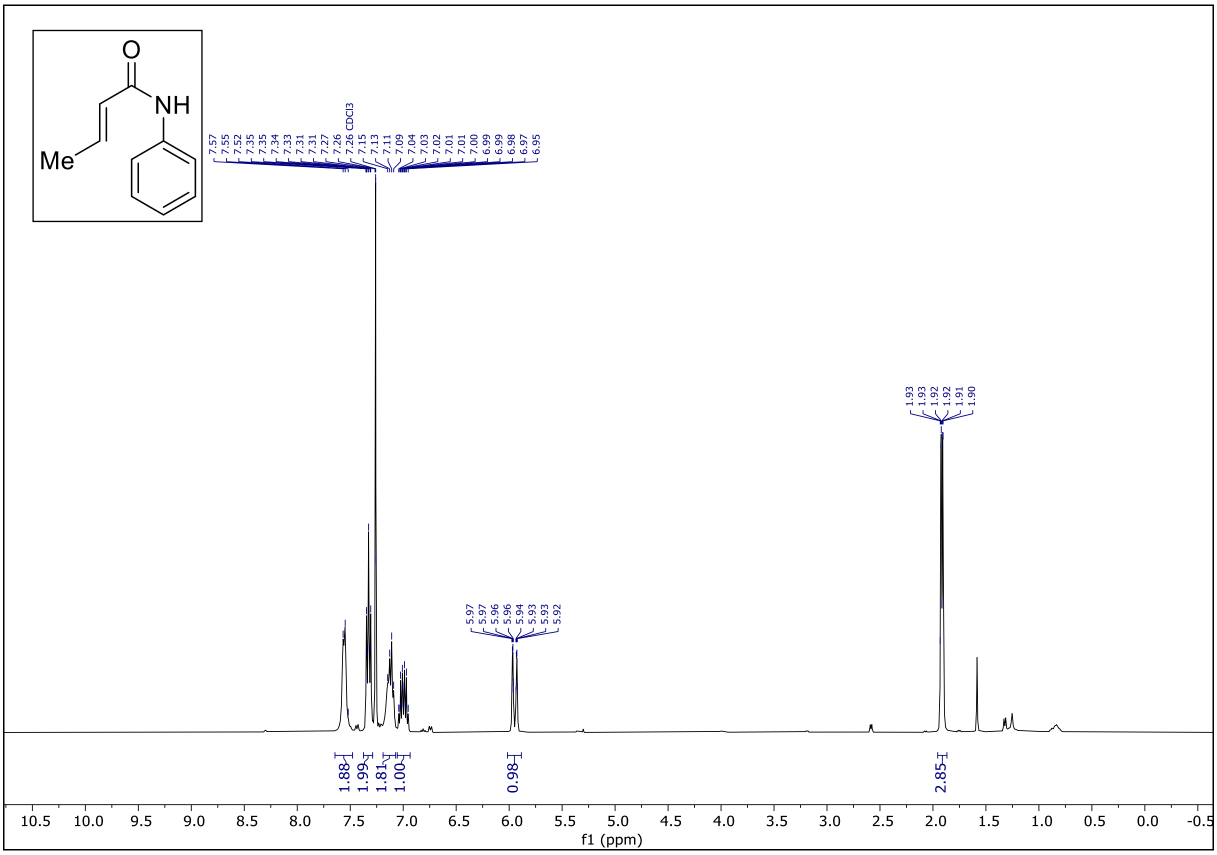

^{1}H NMR (400 MHz, $CDCl_3$): **S20**

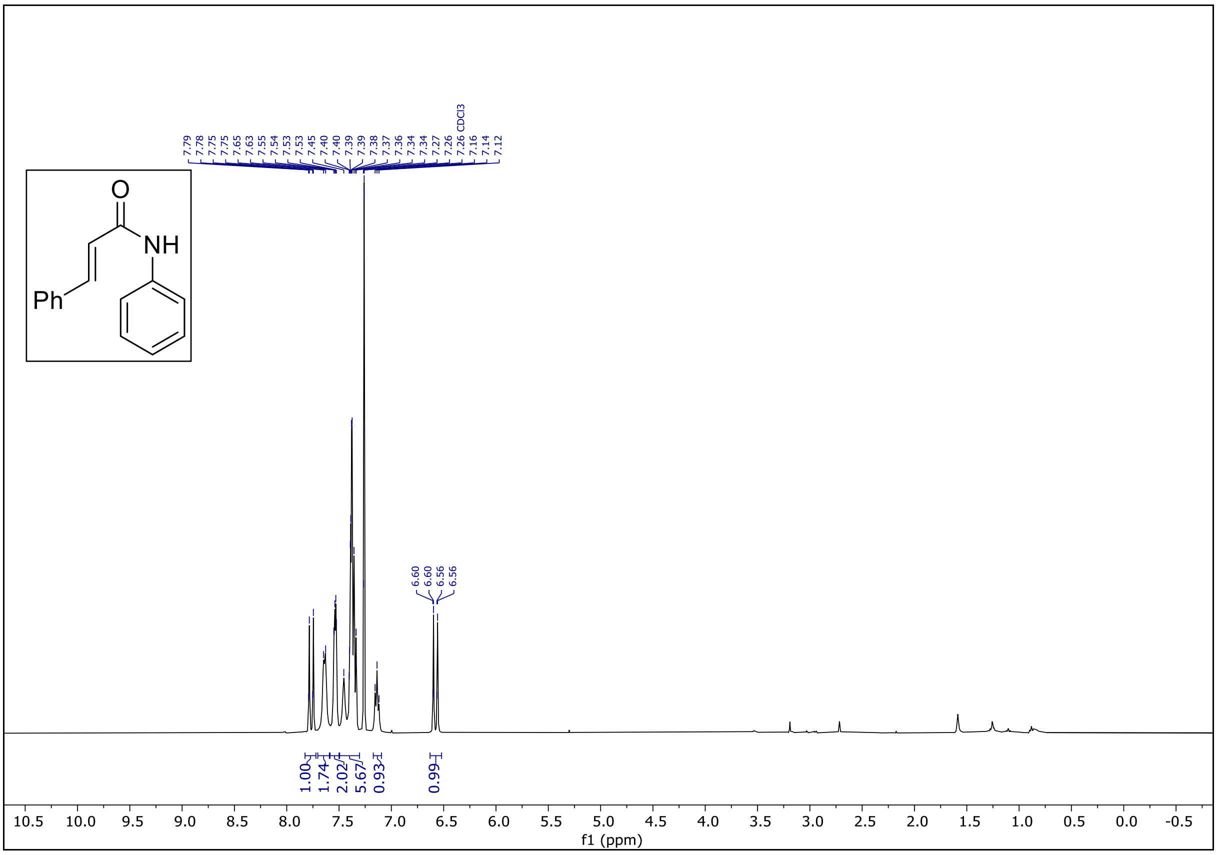

^{1}H NMR (300 MHz, CD_2Cl_2): **Al-2**

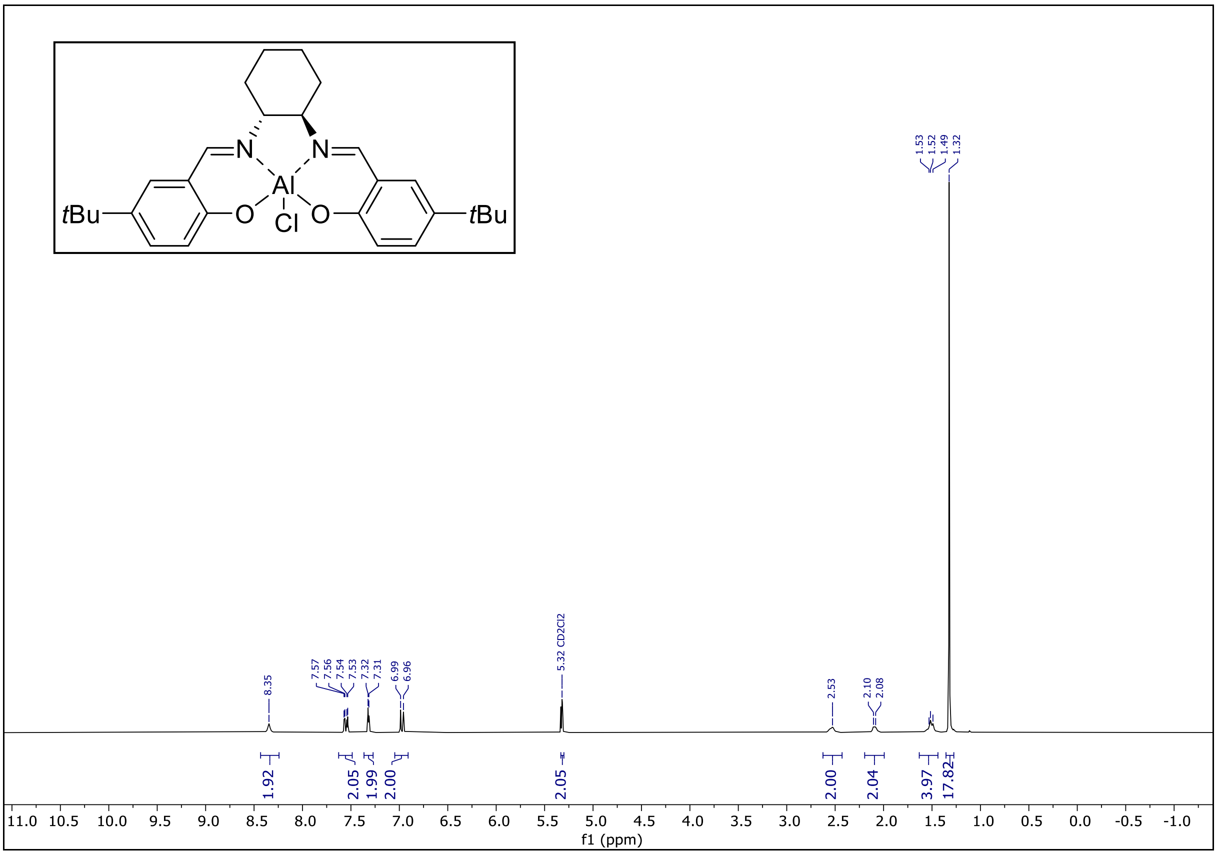

^{1}H NMR (400 MHz, CD_2Cl_2): **Al-3**

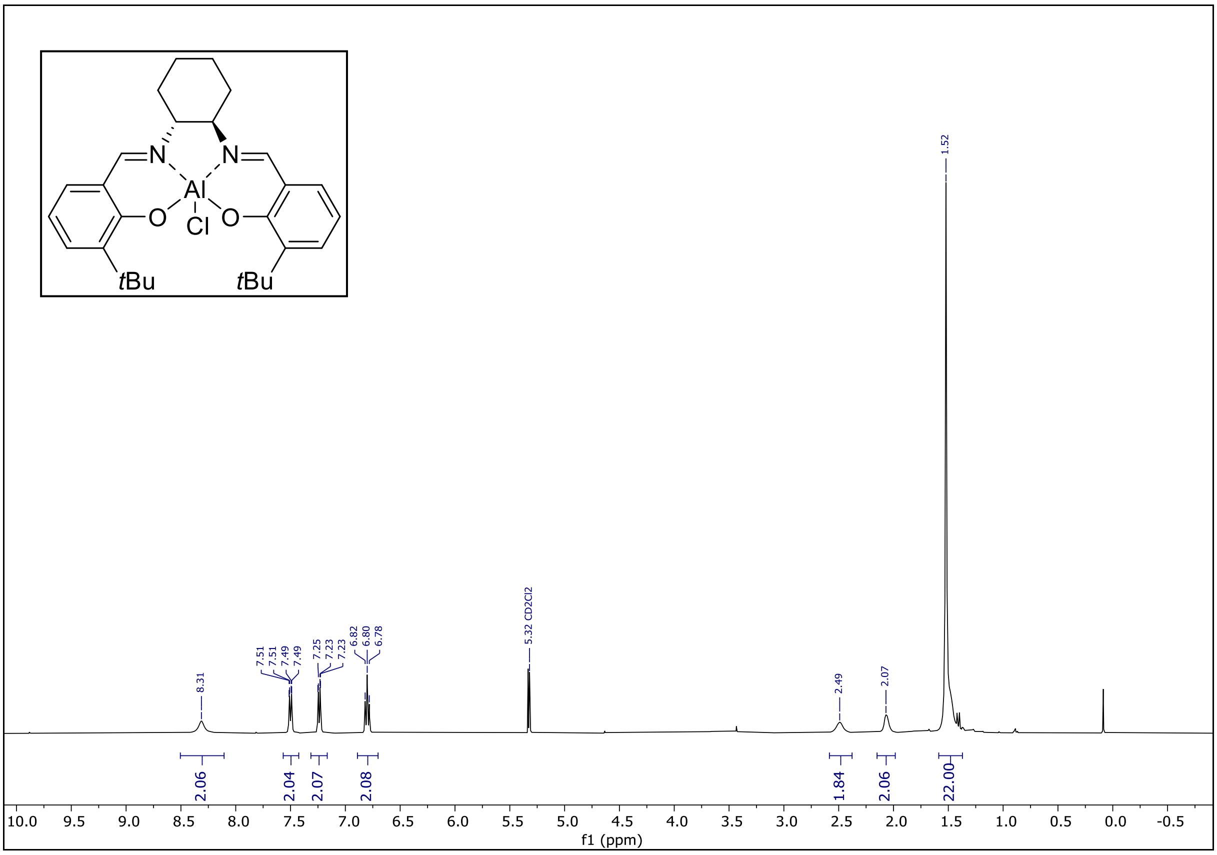

^{1}H NMR (400 MHz, CD_2Cl_2): **Al-4**

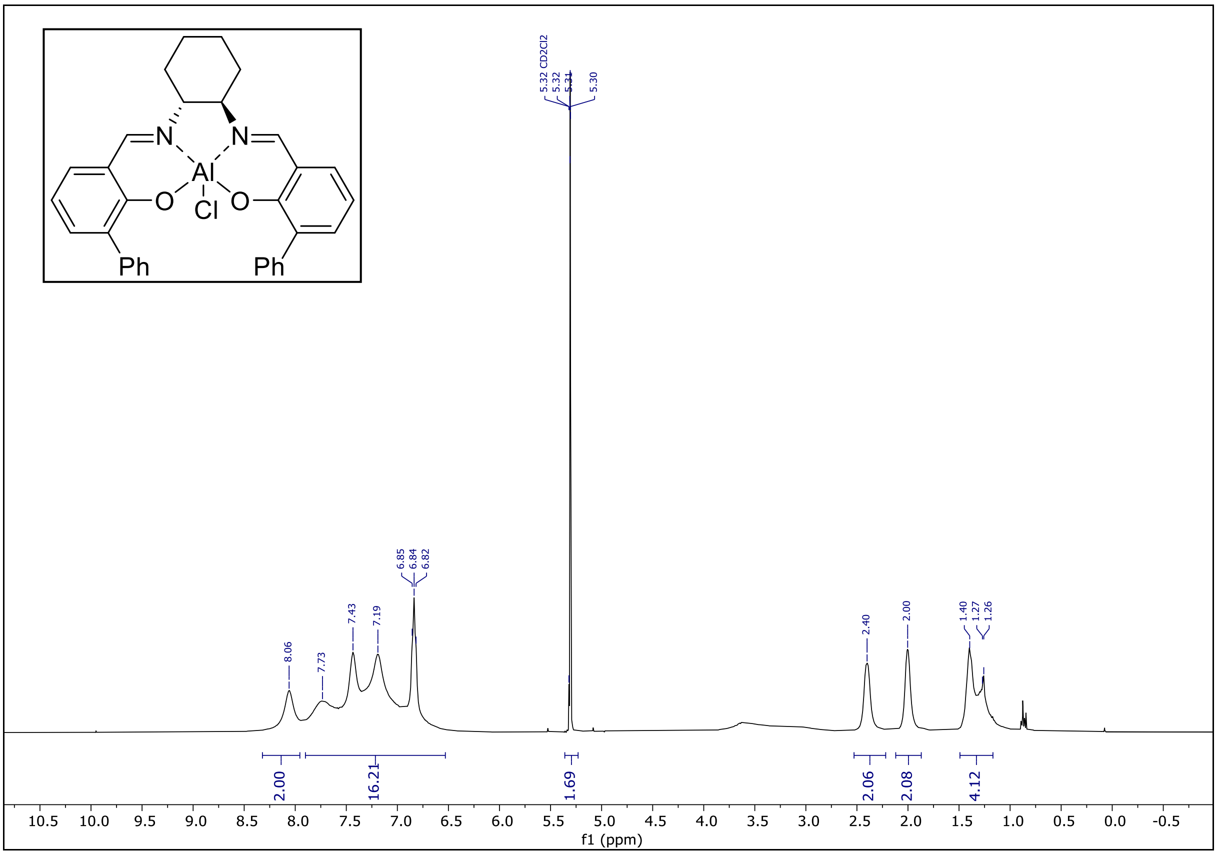

^{1}H NMR (400 MHz, CD_2Cl_2): **Al-8**

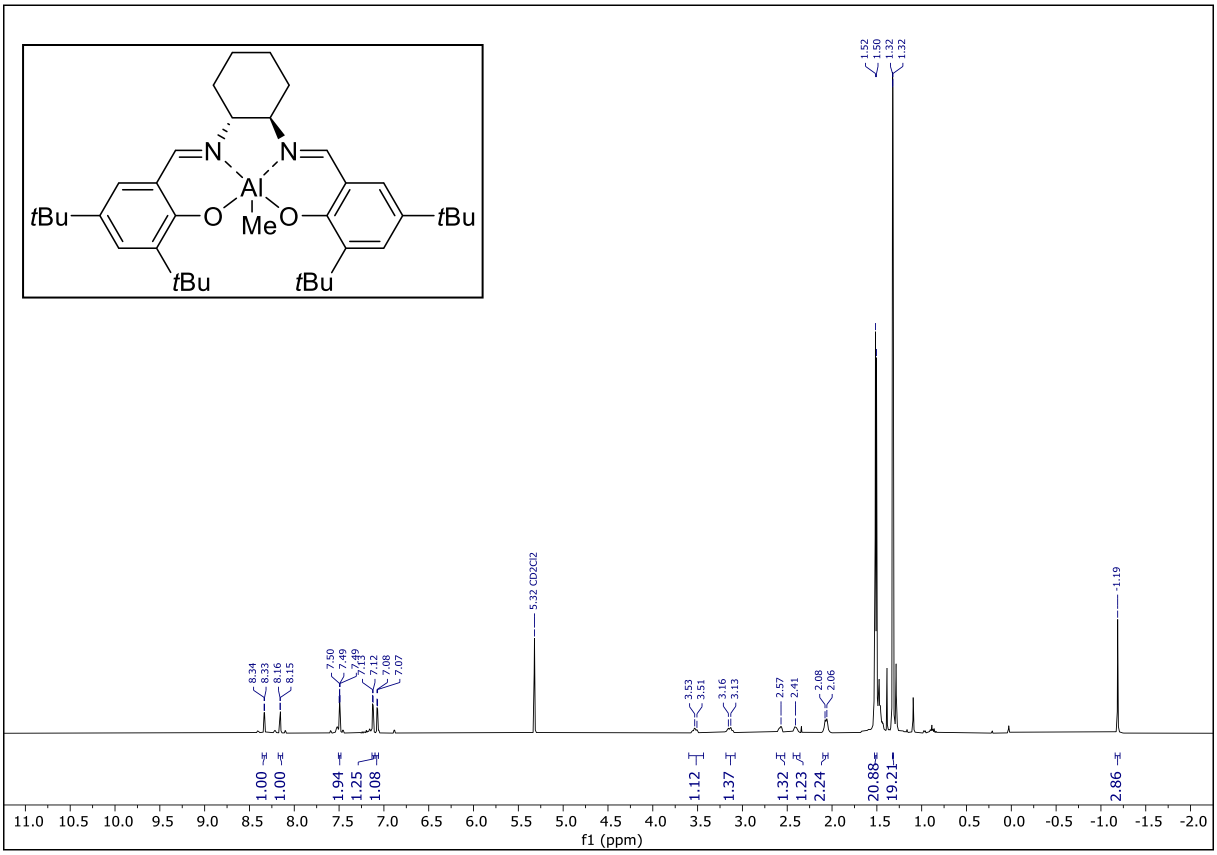

^{1}H NMR (400 MHz, $CDCl_3$): **2a**

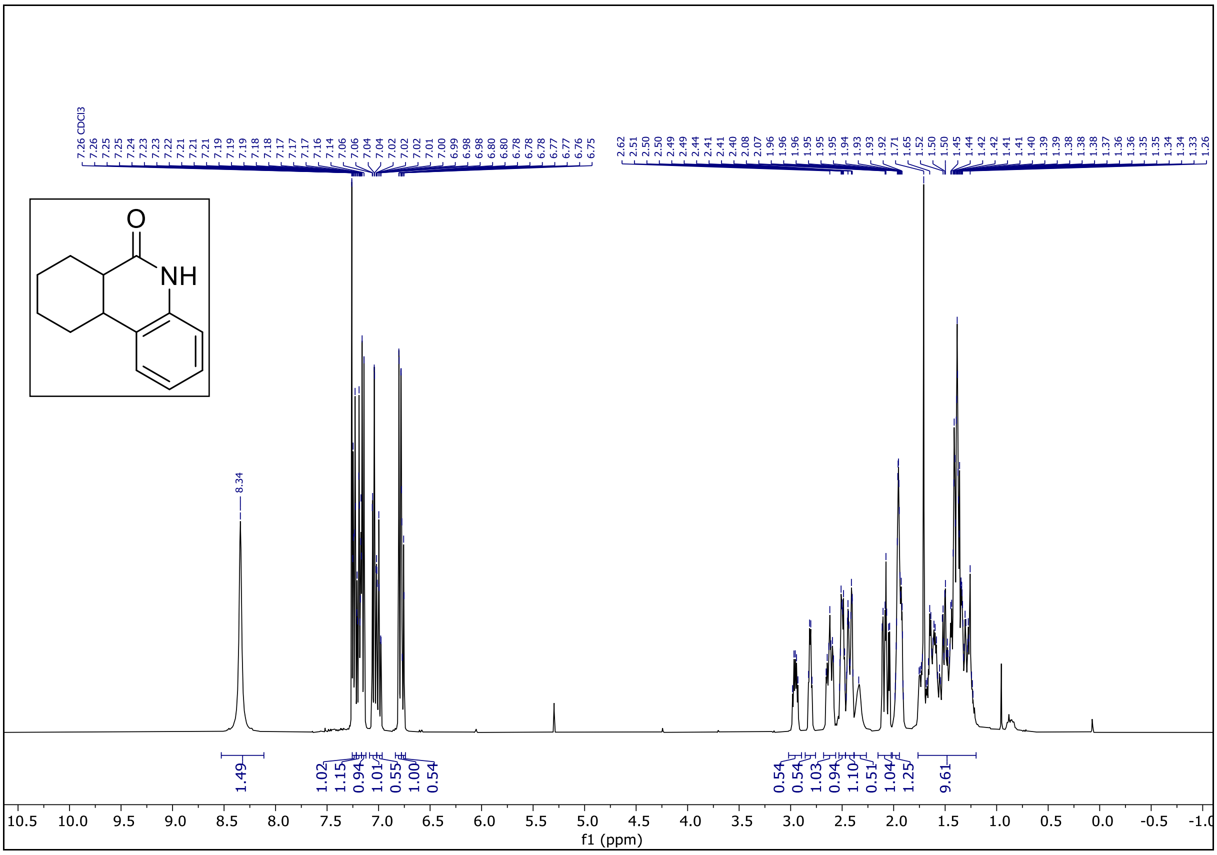

^1H NMR (500 MHz, $CDCl_3$): *trans*-**2b**

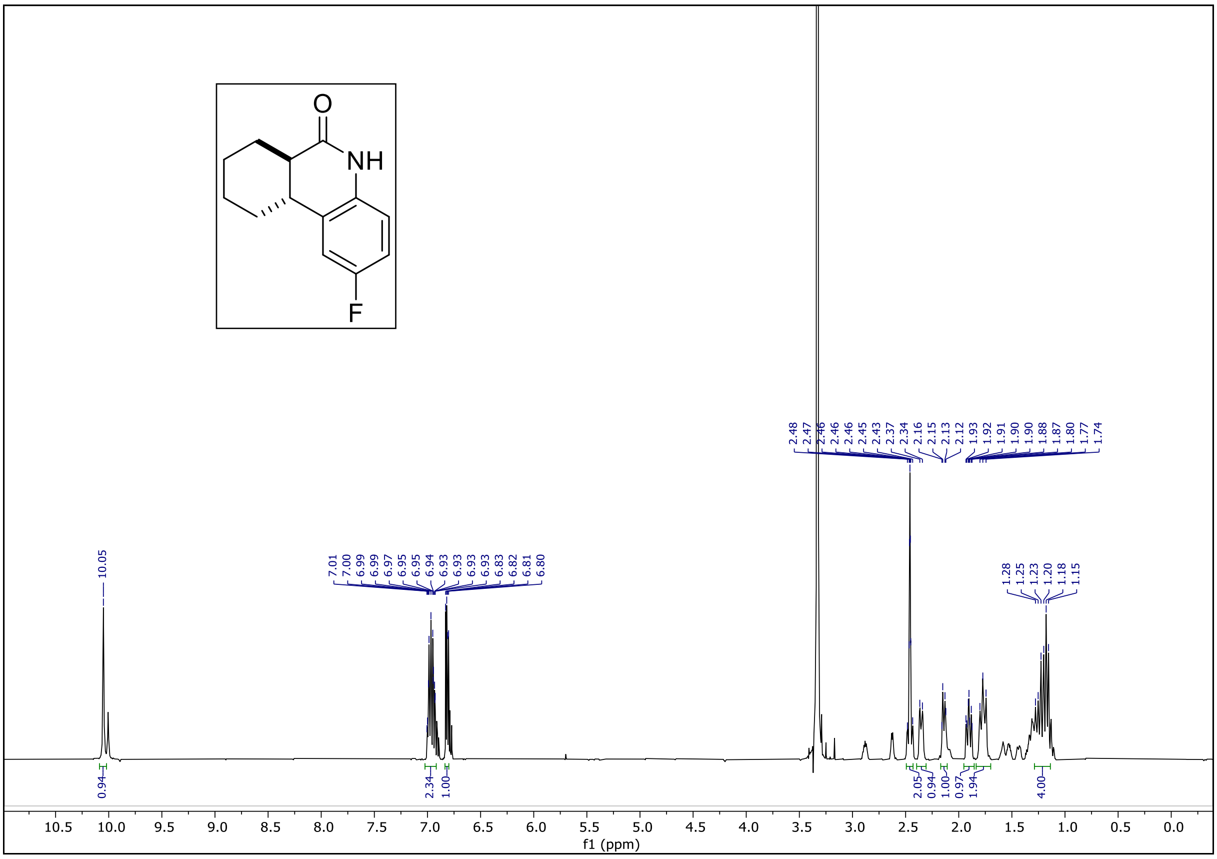

^{13}C NMR {^{19}F} (126 MHz, $CDCl_3$): *trans*-**2b**

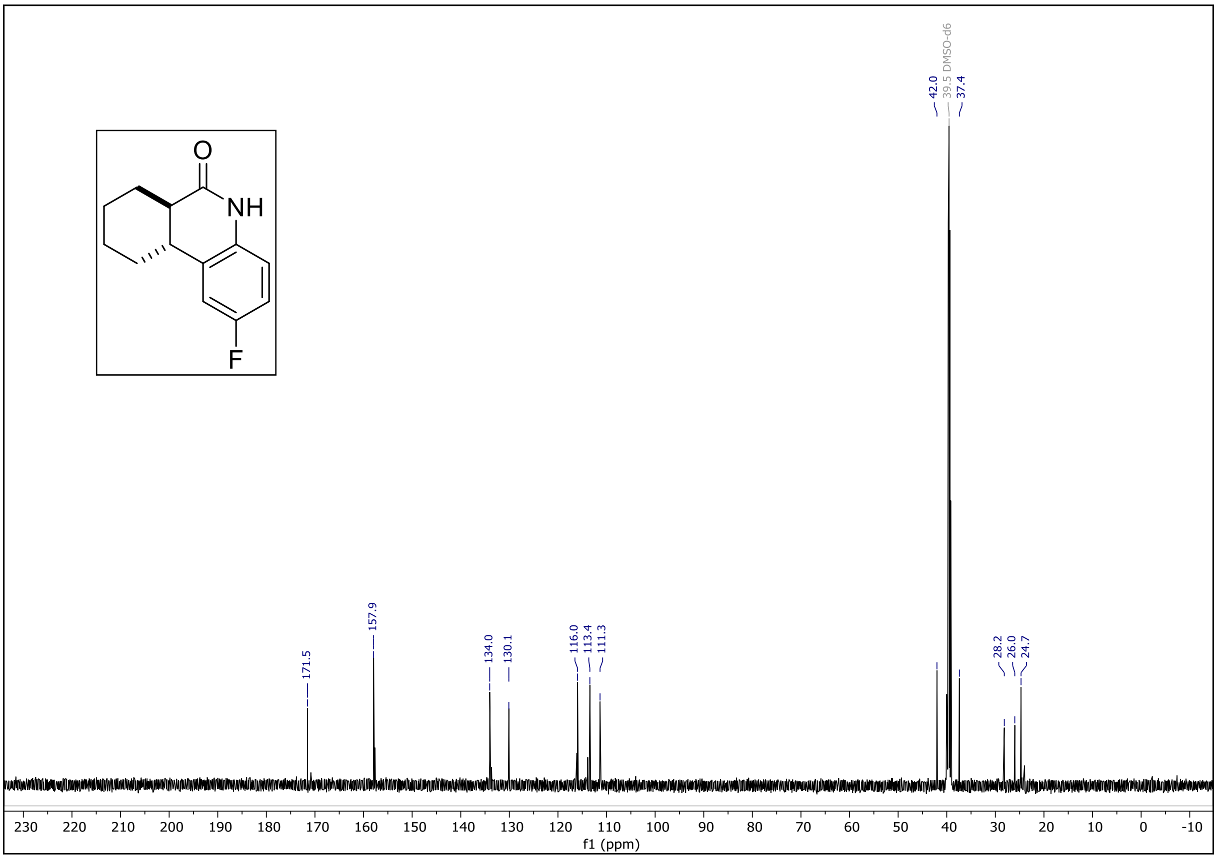

^{19}F NMR (470 MHz, $CDCl_3$): *trans*-**2b**

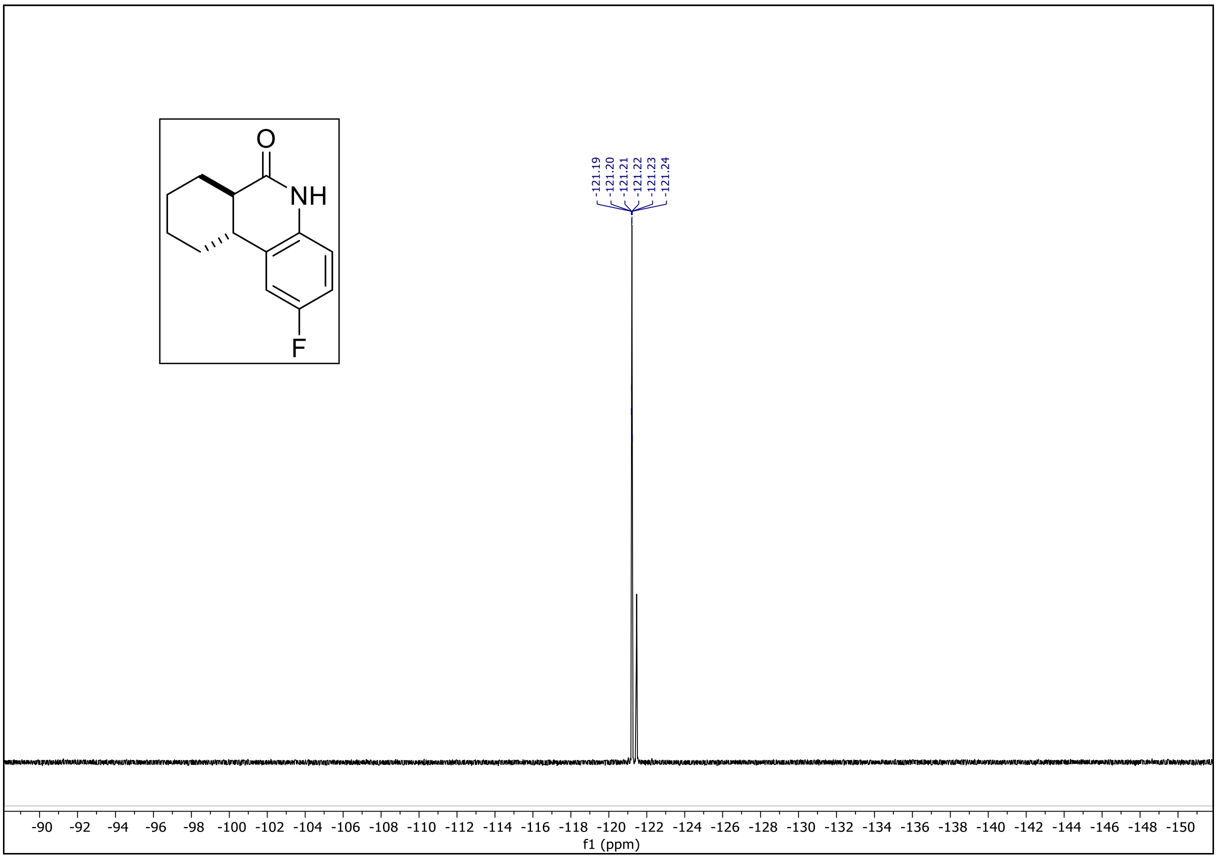

^{1}H NMR (500 MHz, $CDCl_3$): *cis*-**2b**

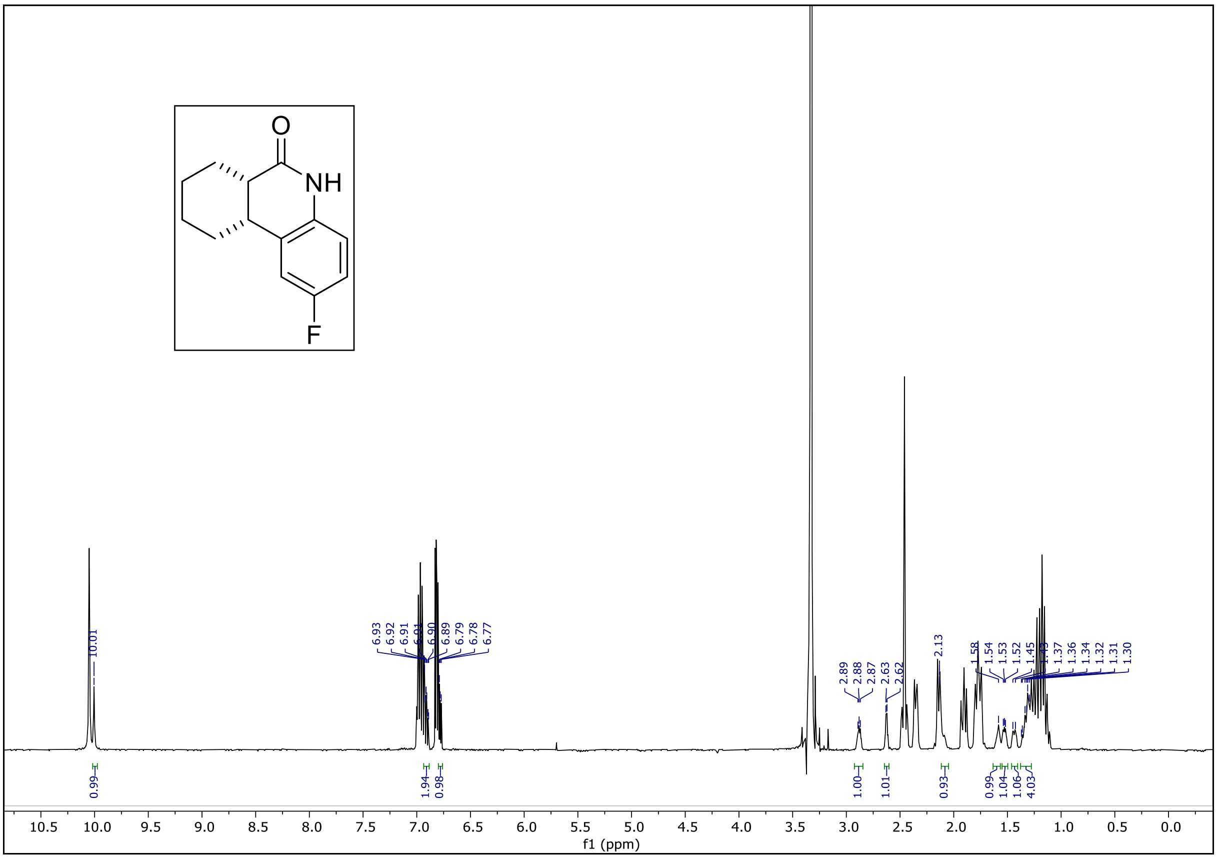

^{13}C NMR {^{19}F} (126 MHz, $CDCl_3$): *cis*-**2b**

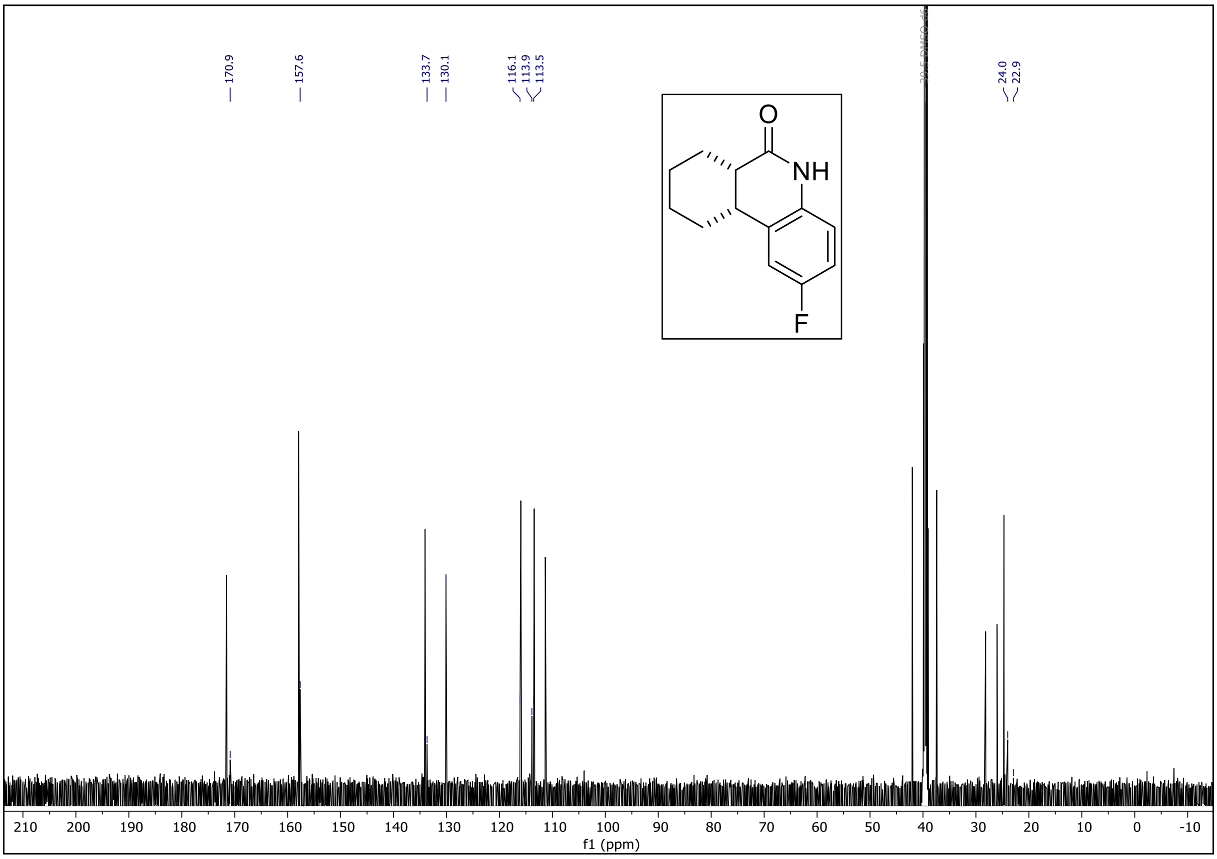

^{19}F NMR (470 MHz, $CDCl_3$): *cis*-**2b**

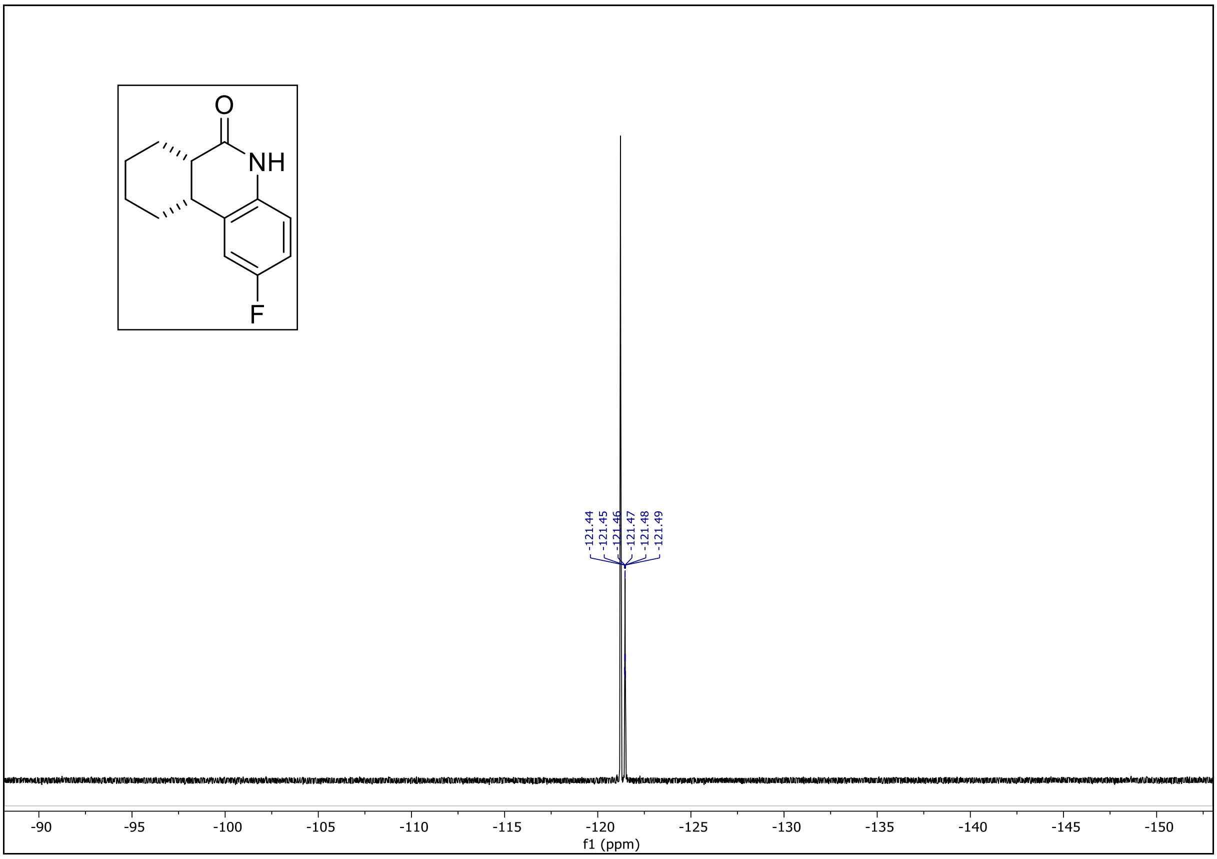

^{1}H NMR (500 MHz, $CDCl_3$): *trans*-**2c**

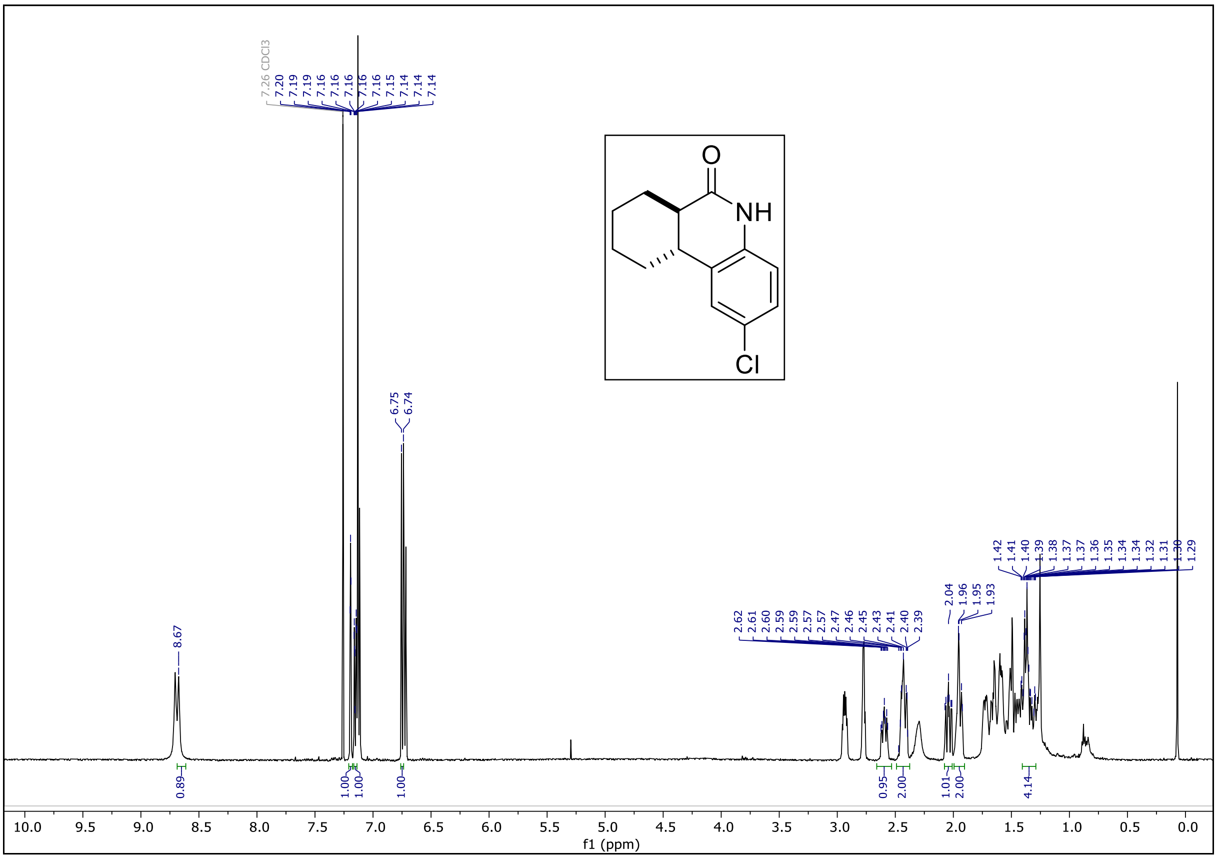

^{13}C NMR (126 MHz, $CDCl_3$): *trans*-**2c**

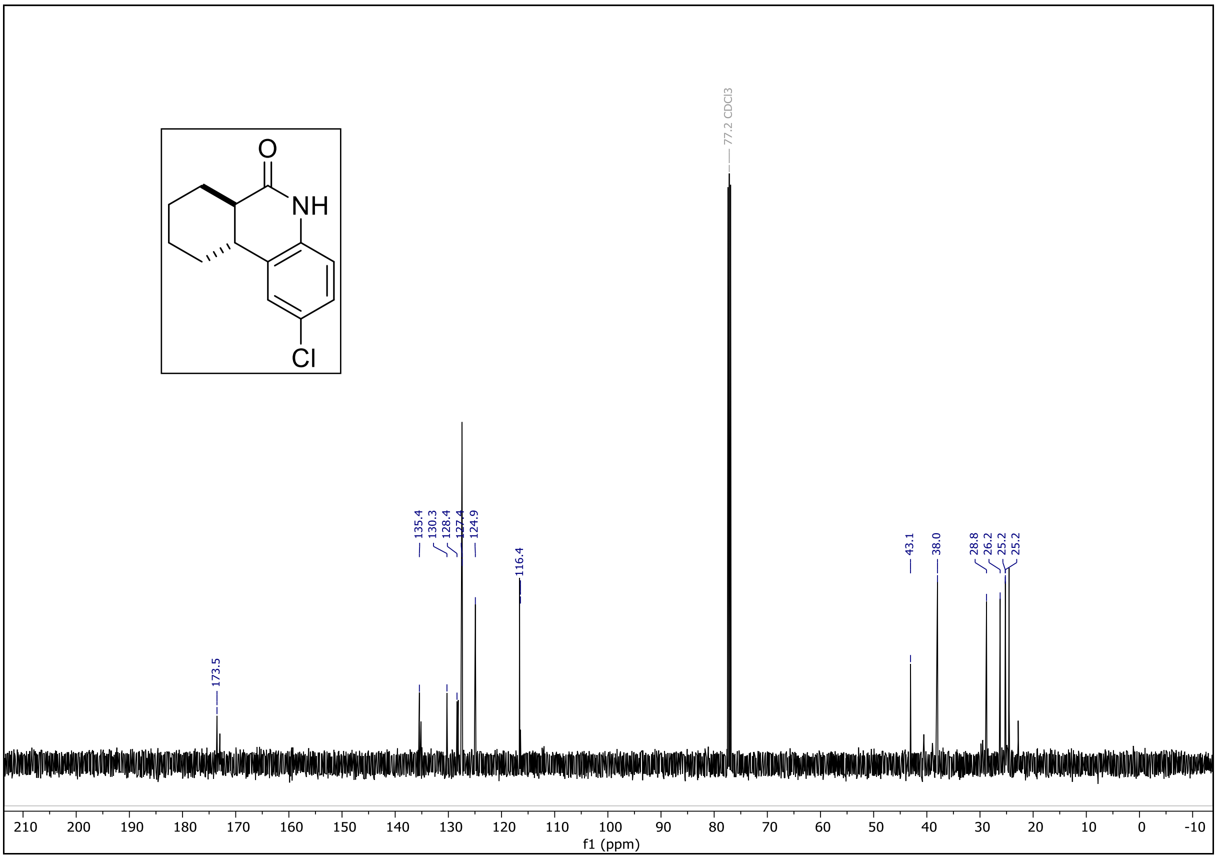

^{1}H NMR (500 MHz, $CDCl_3$): *cis*-**2c**

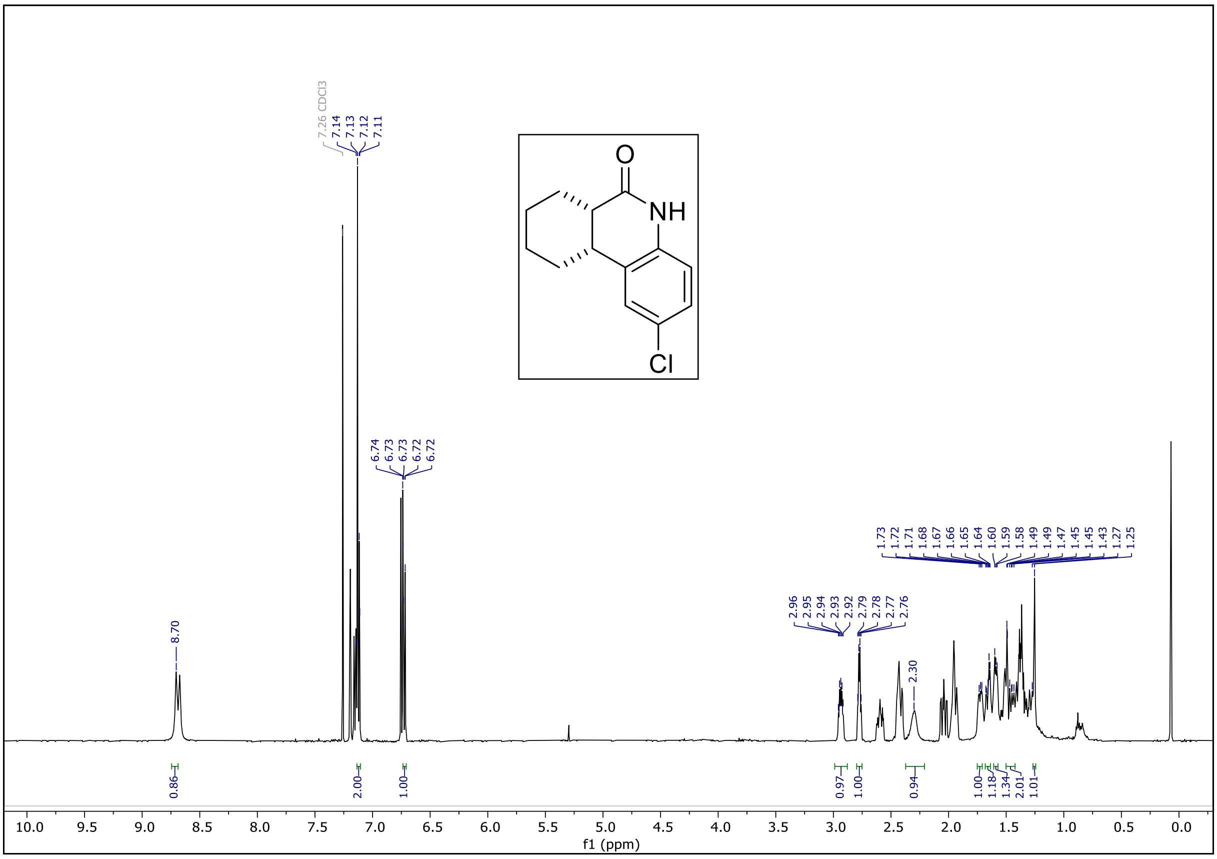

^{13}C NMR (126 MHz, $CDCl_3$): *cis*-**2c**

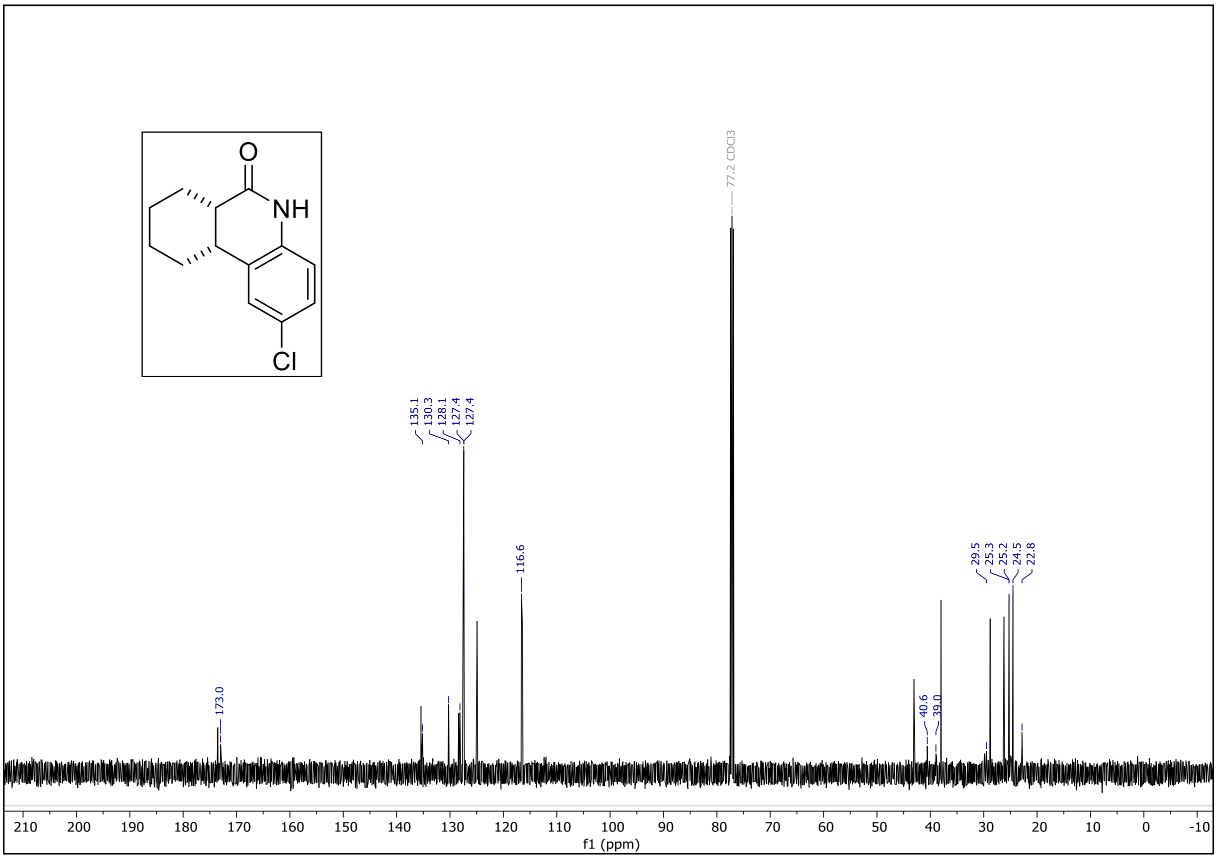

^{1}H NMR (600 MHz, $CDCl_3$): *trans*-**2d**

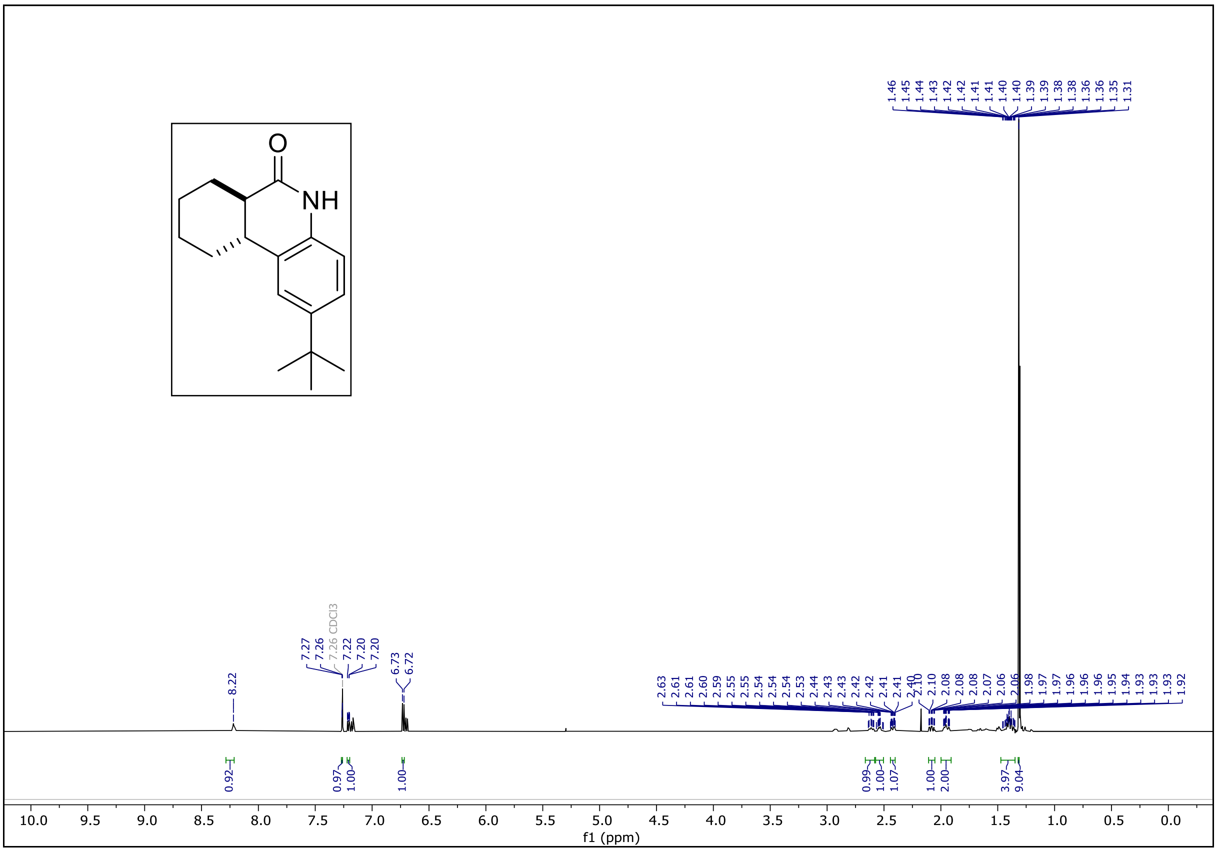

^{13}C NMR (151 MHz, $CDCl_3$): *trans*-**2d**

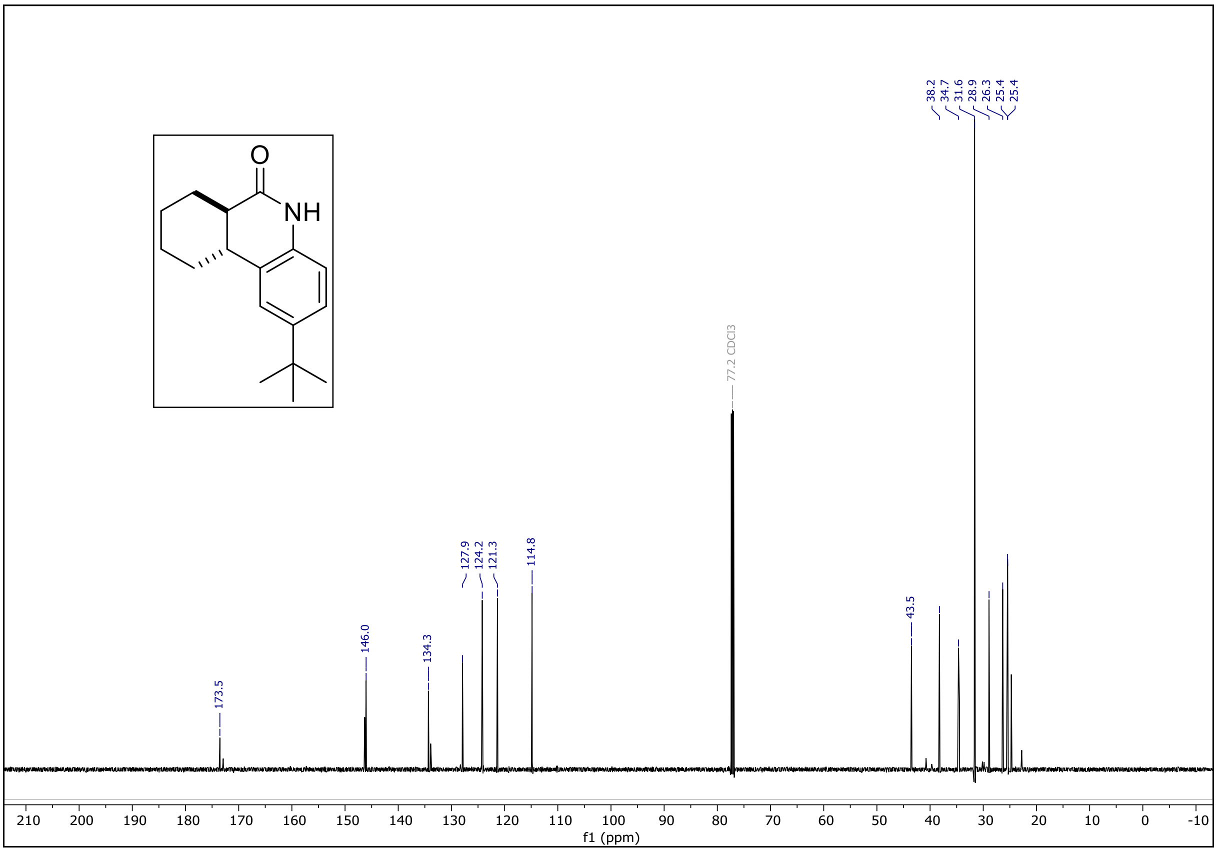

^{1}H NMR (600 MHz, $CDCl_3$): *cis*-**2d**

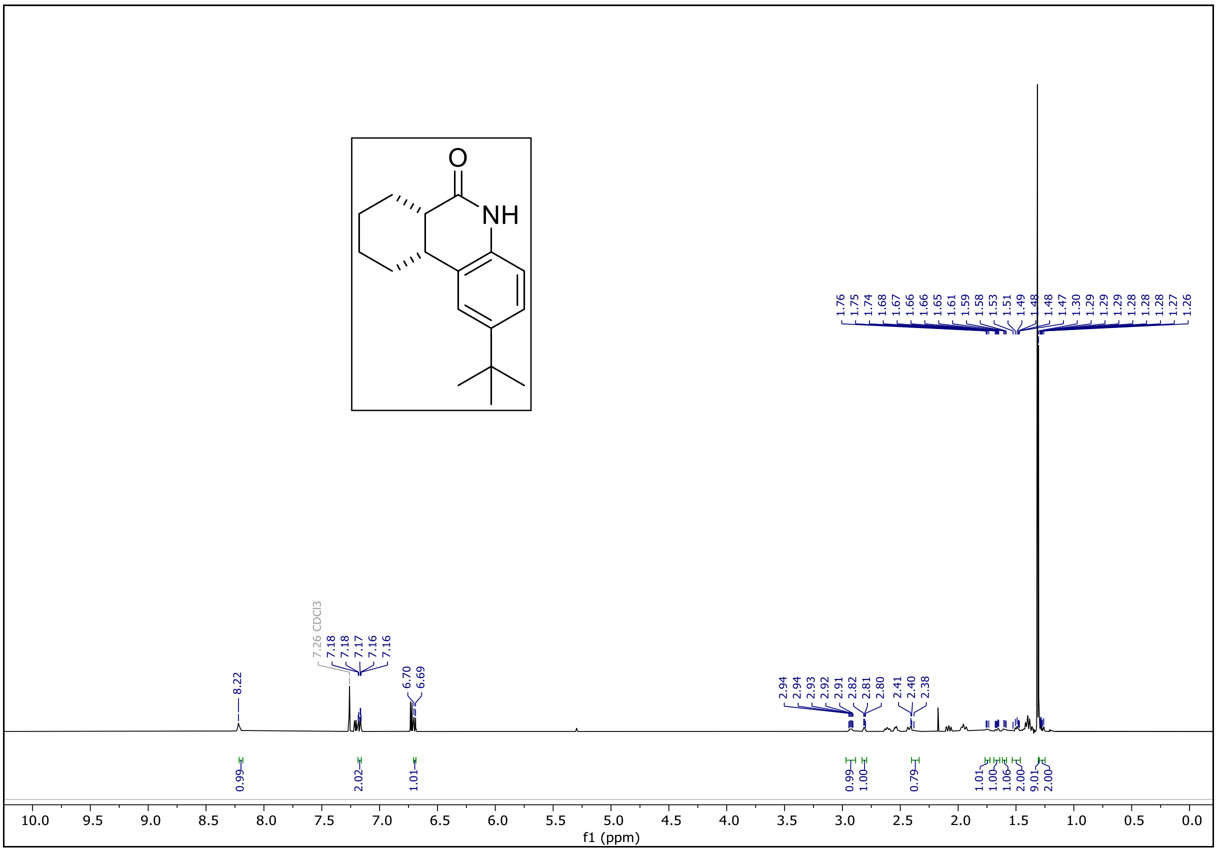

^{13}C NMR (151 MHz, $CDCl_3$): *cis*-**2d**

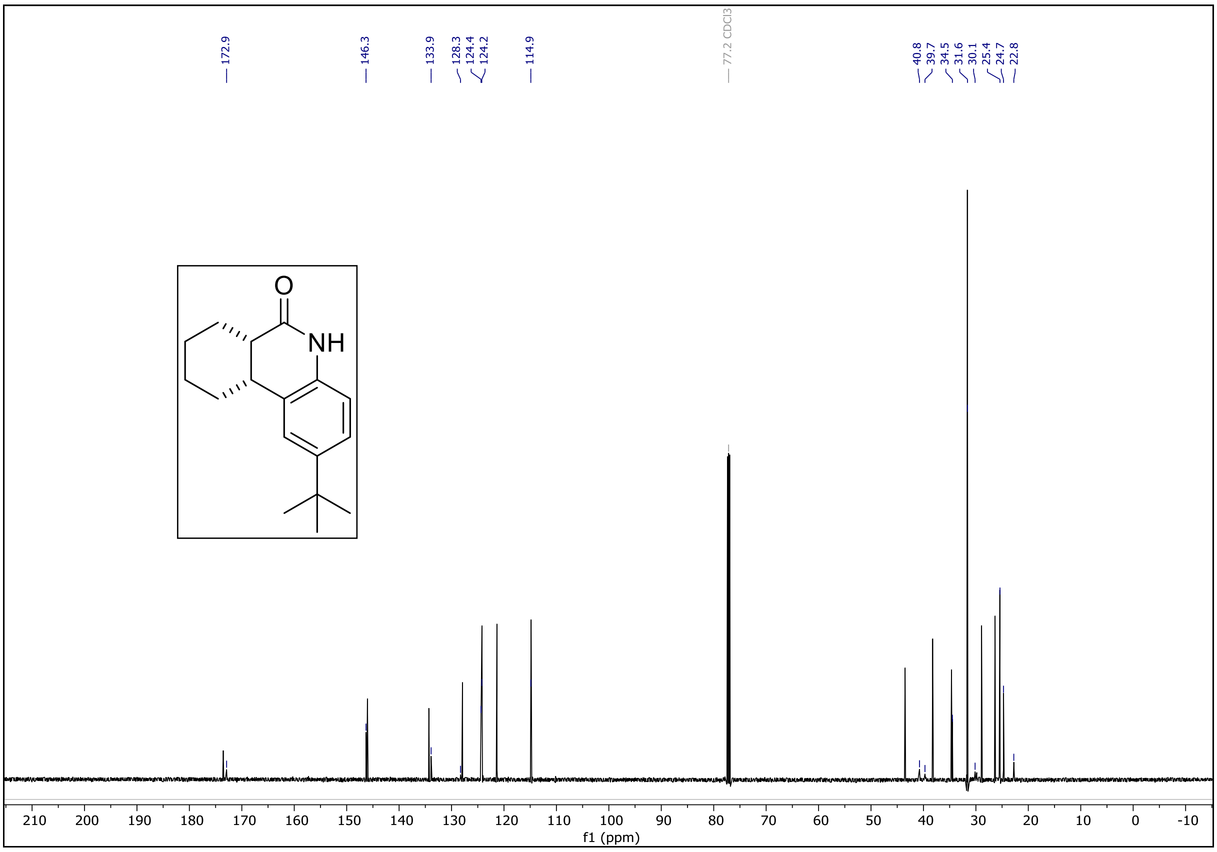

^{1}H NMR (500 MHz, $CDCl_3$): *trans*-**2e**

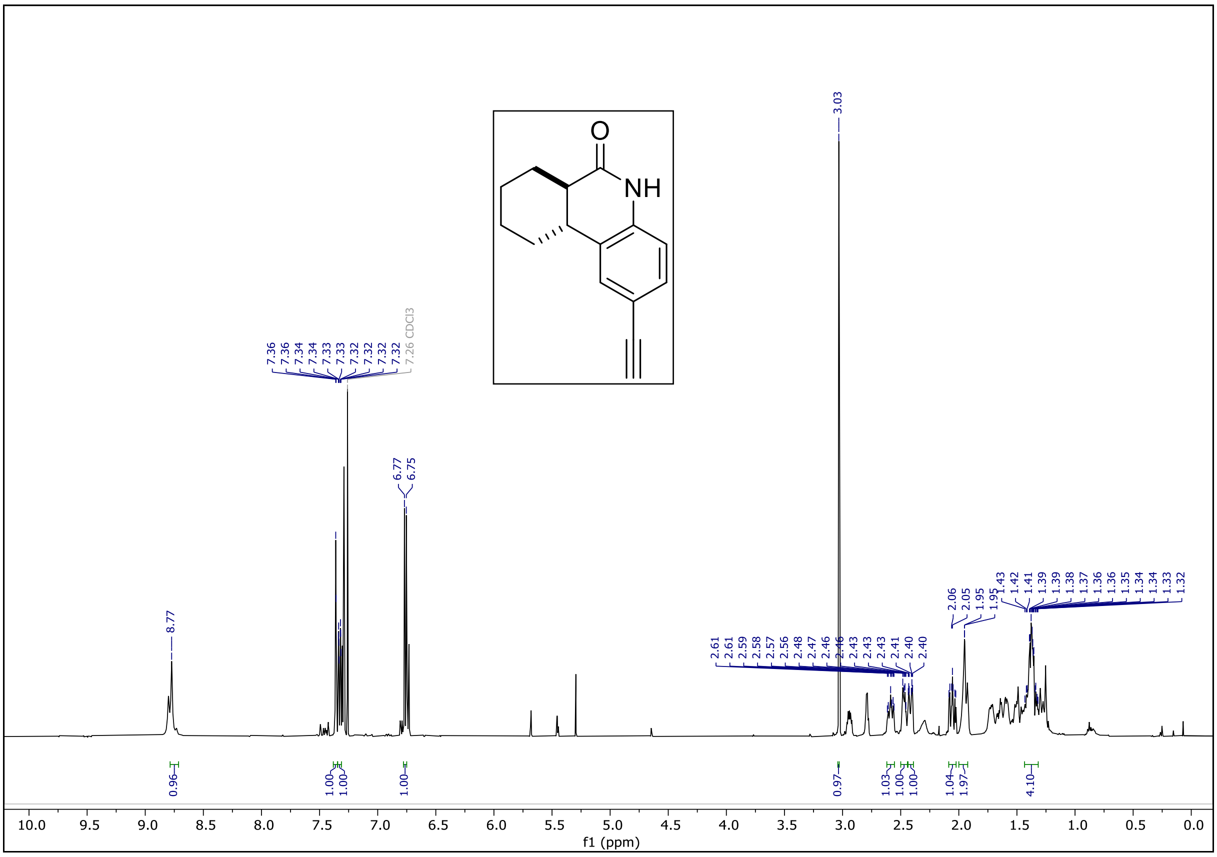

^{13}C NMR (126 MHz, $CDCl_3$): *trans*-**2e**

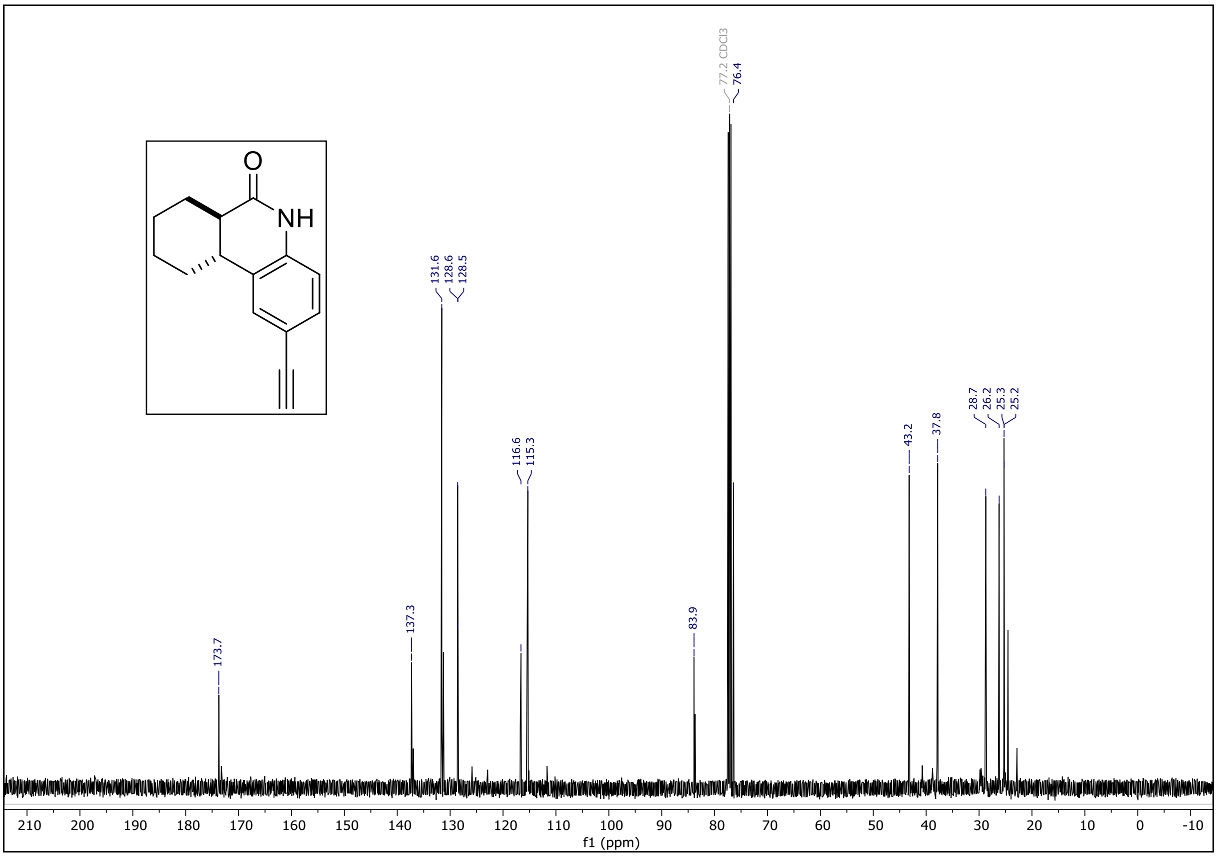

^{1}H NMR (500 MHz, $CDCl_3$): *cis*-**2e**

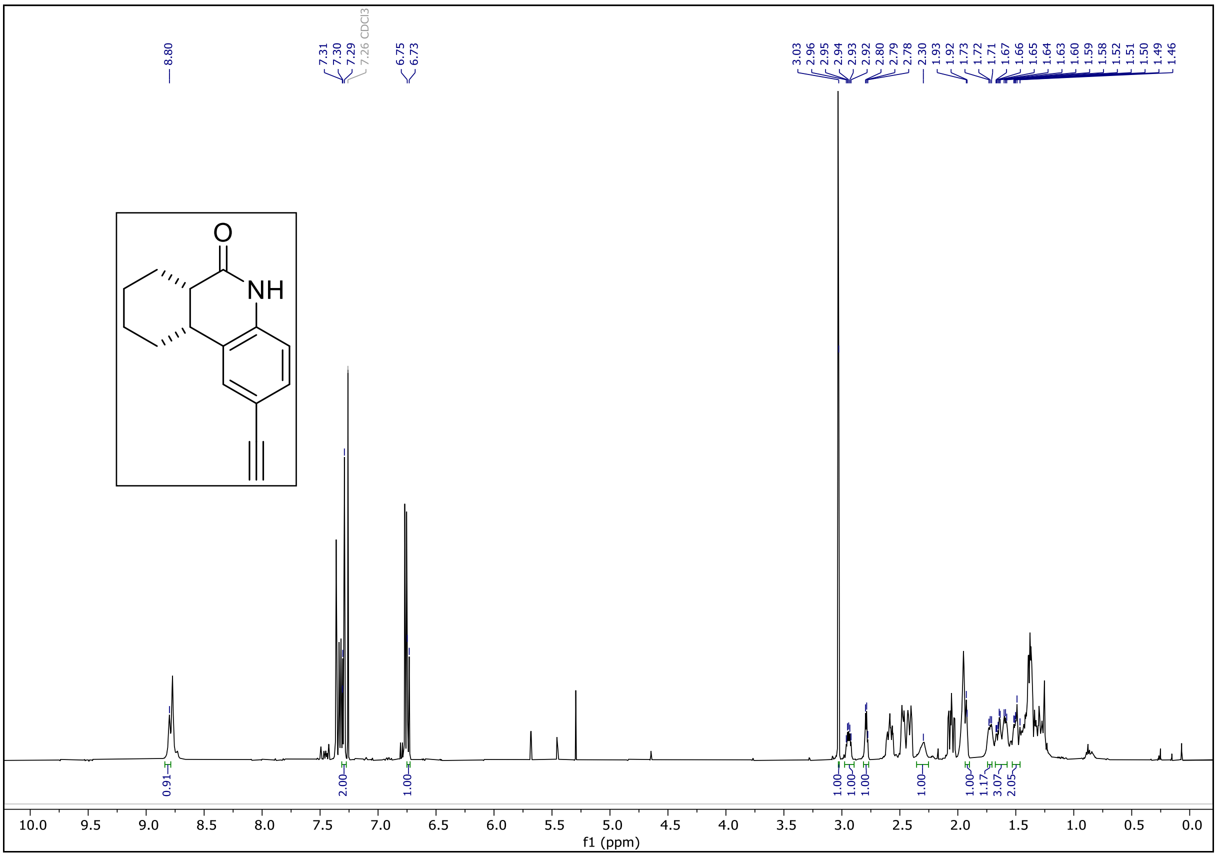

^{13}C NMR (126 MHz, $CDCl_3$): *cis*-**2e**

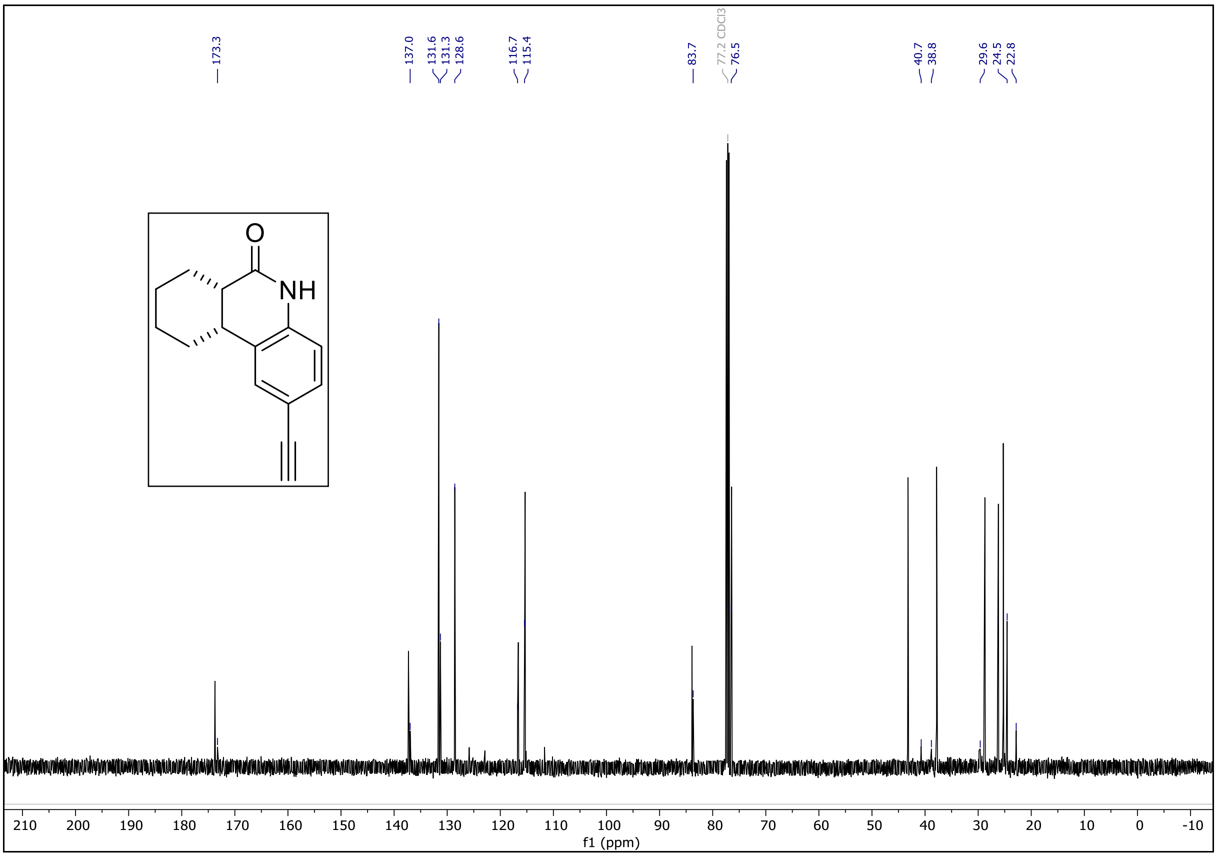

^{1}H NMR (600 MHz, $CDCl_3$): *trans*-**2f**

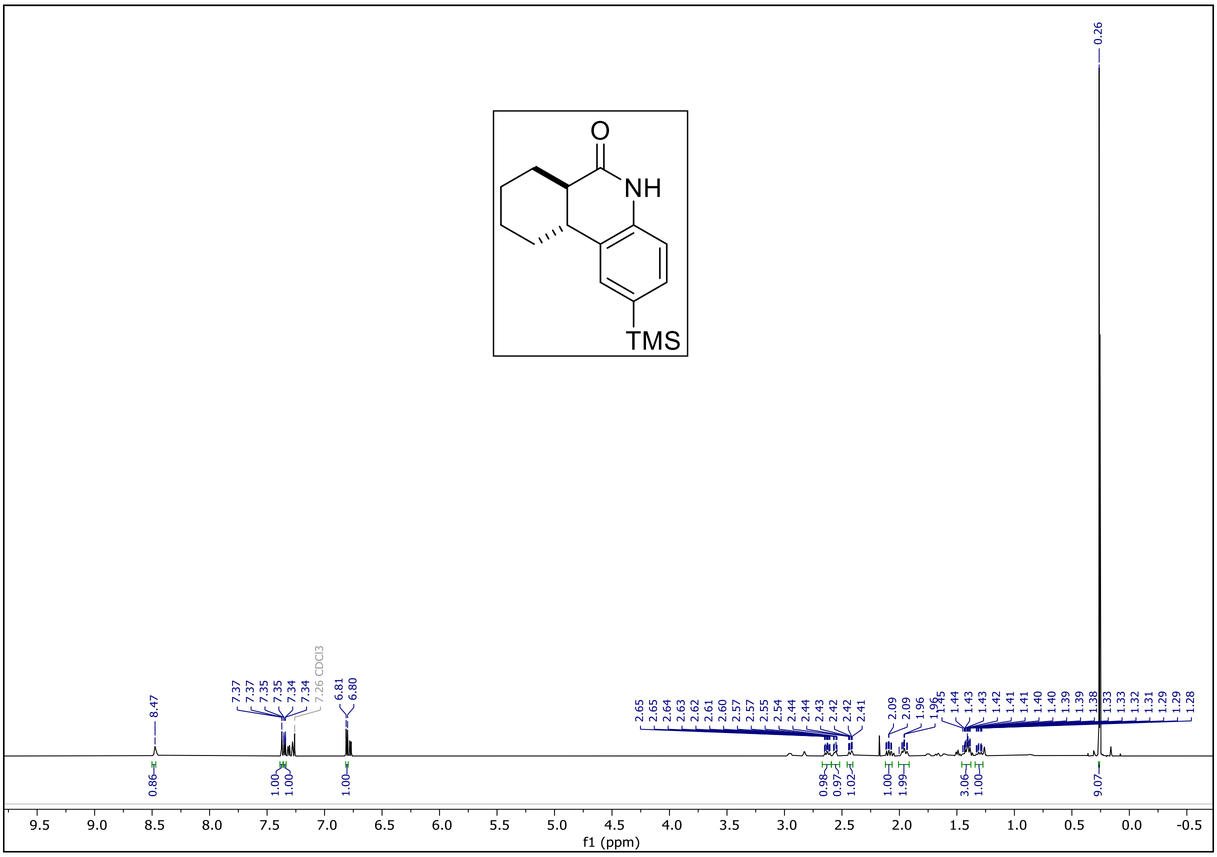

^{13}C NMR (151 MHz, $CDCl_3$): *trans*-**2f**

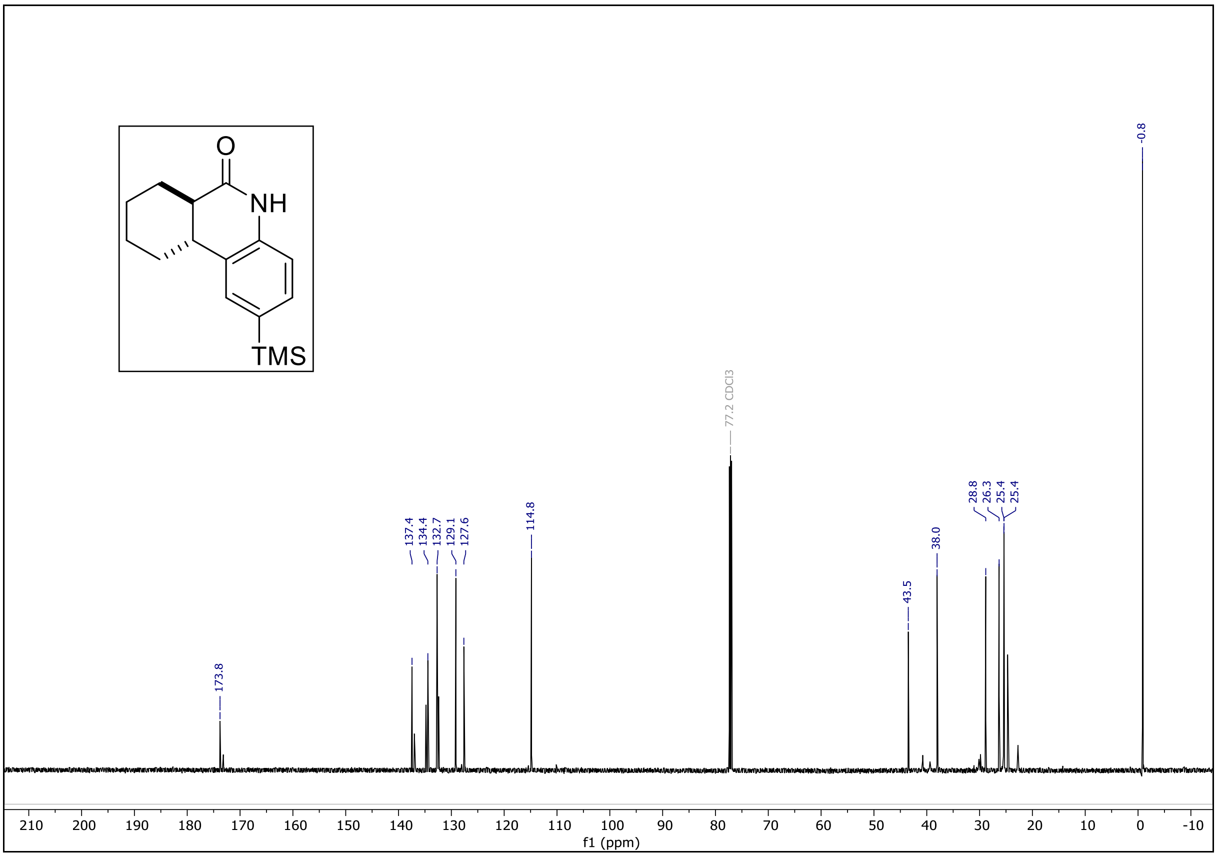

^{29}Si NMR (119 MHz, $CDCl_3$): *trans*-**2f**

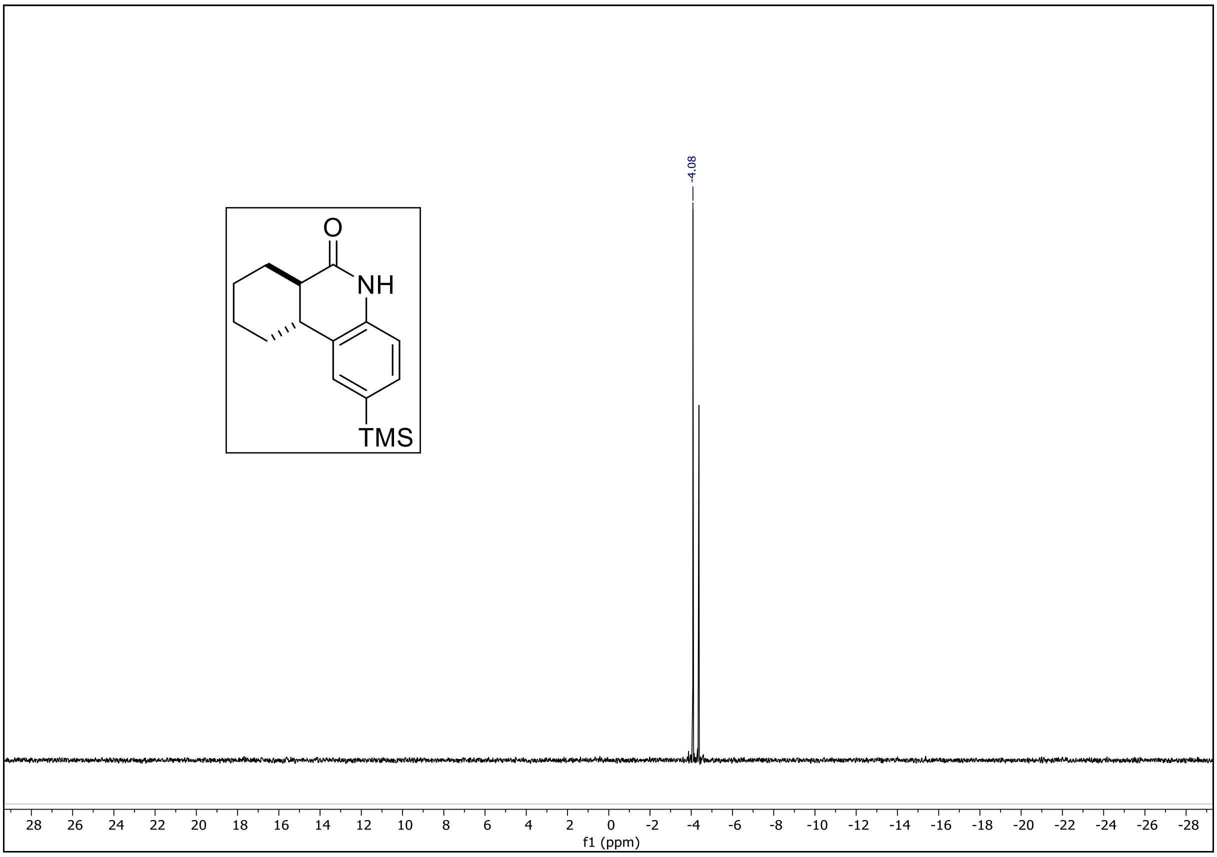

^{1}H NMR (600 MHz, $CDCl_3$): *cis*-**2f**

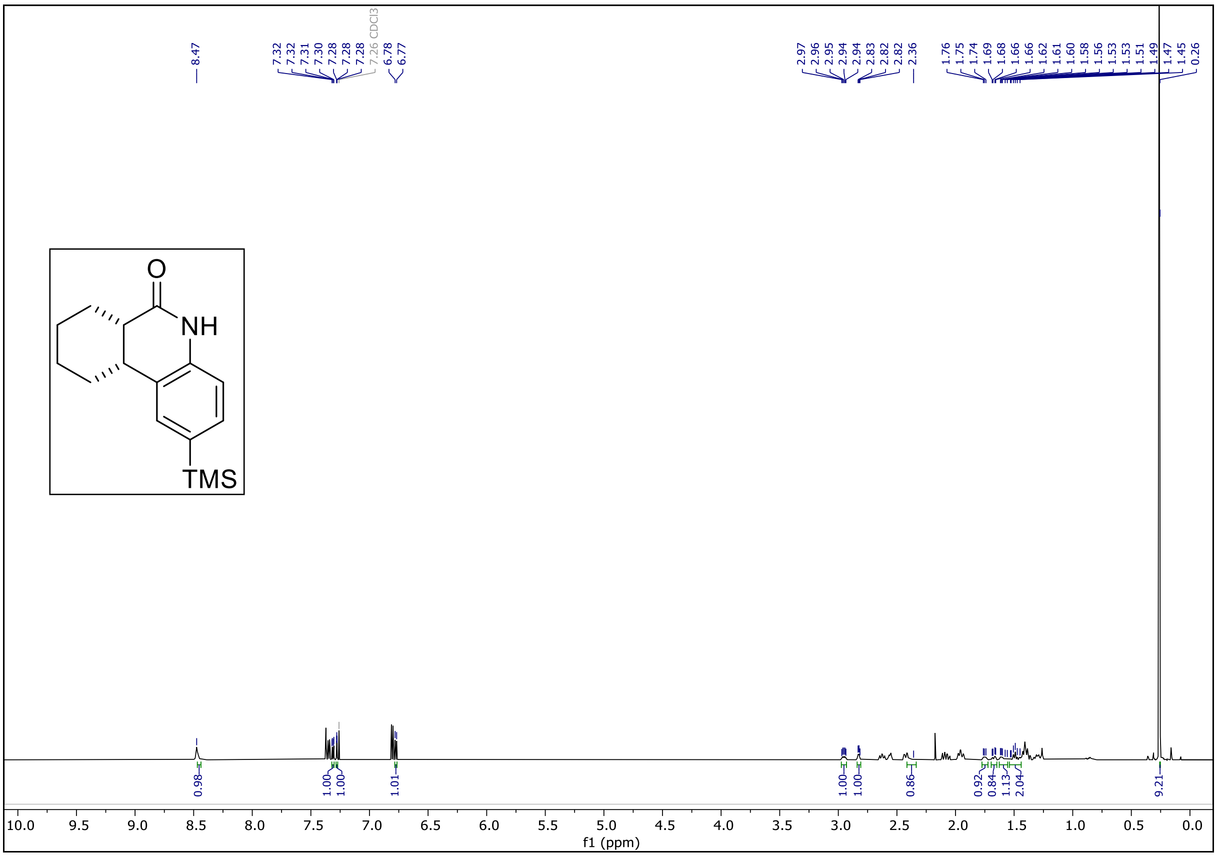

^{13}C NMR (151 MHz, $CDCl_3$): *cis*-**2f**

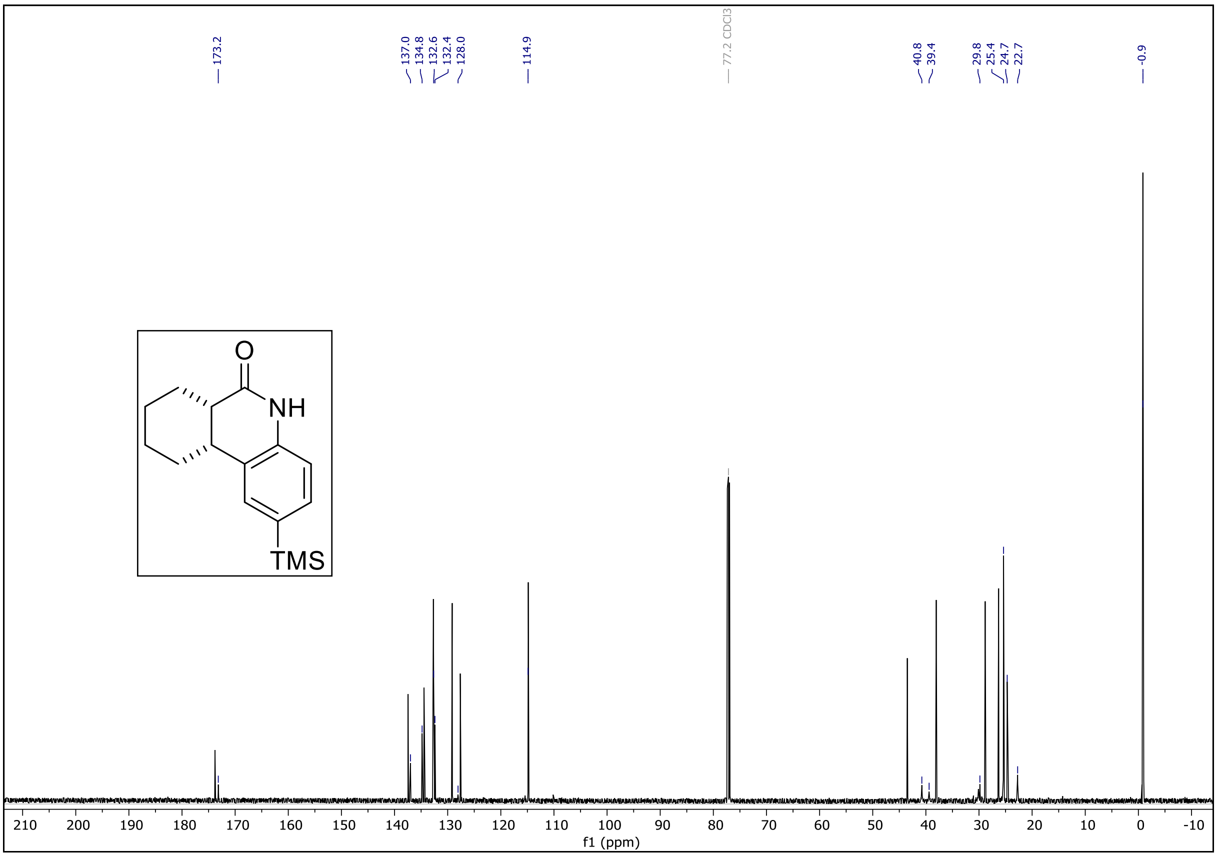

^{29}Si NMR (119 MHz, $CDCl_3$): *cis*-**2f**

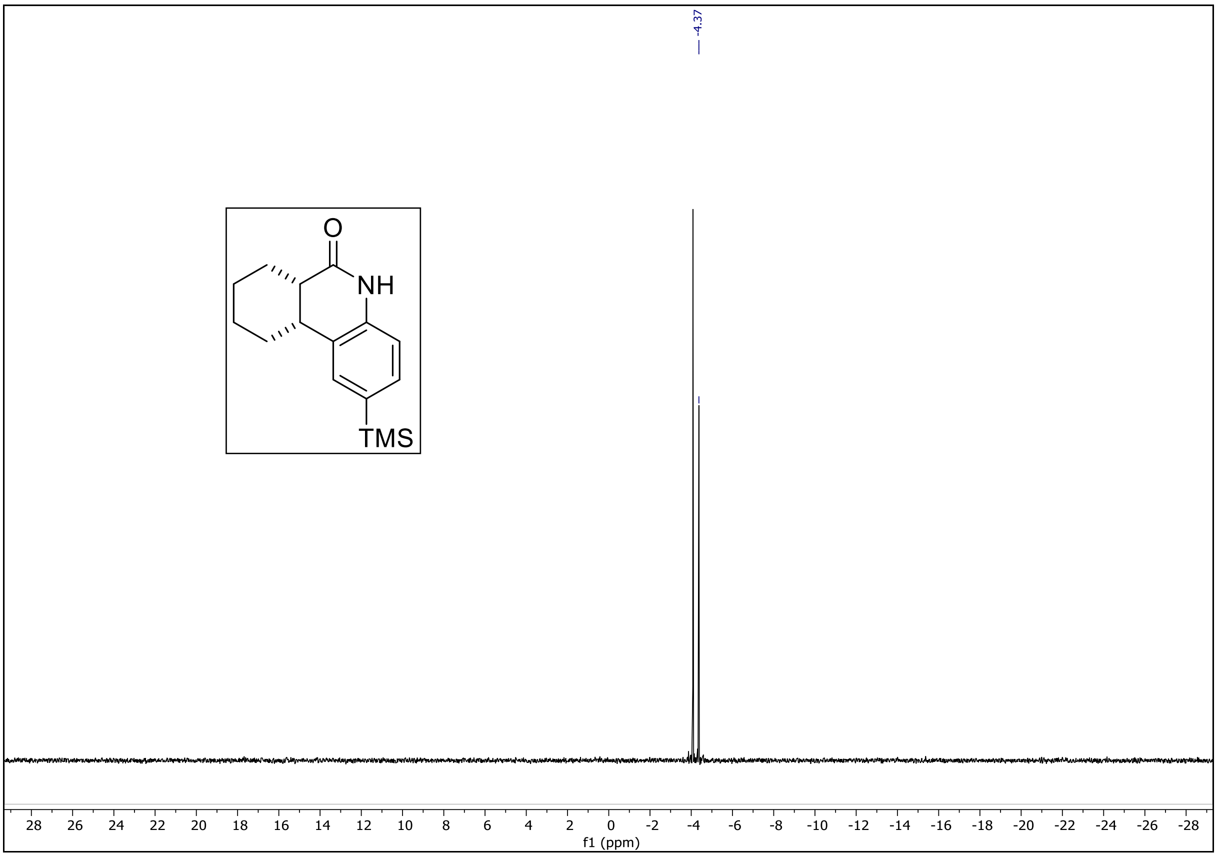

^{1}H NMR (500 MHz, $CDCl_3$): *trans*-**2g**

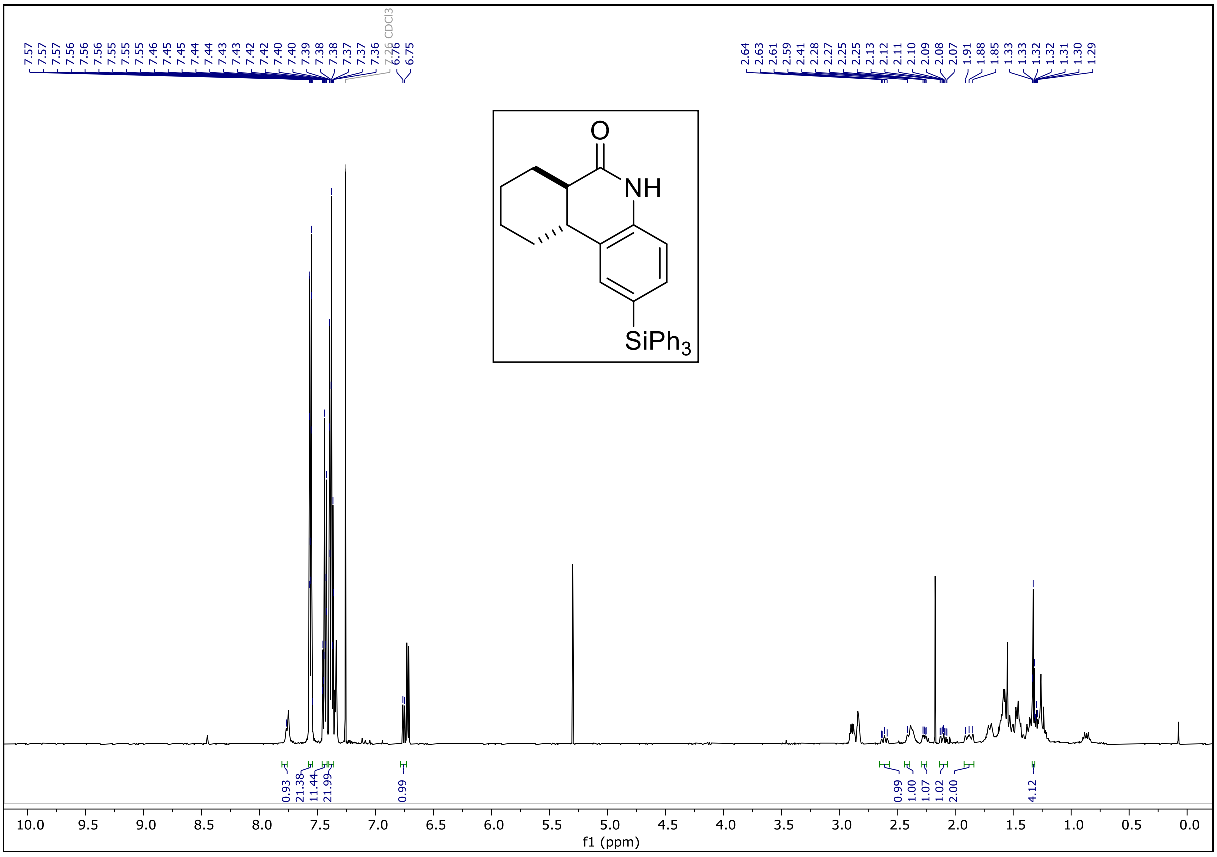

^{13}C NMR (1261 MHz, $CDCl_3$): *trans*-**2g**

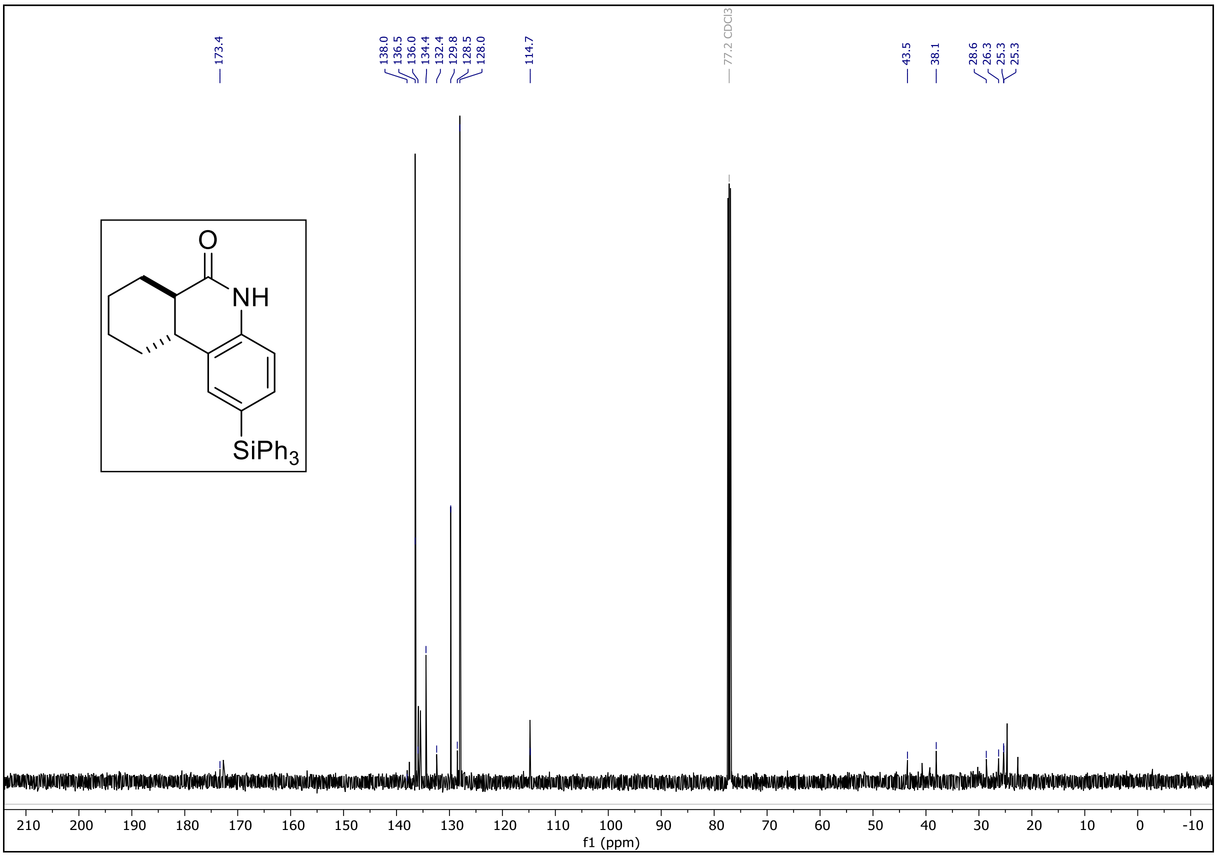

^{29}Si NMR (99 MHz, $CDCl_3$): *trans*-**2g**

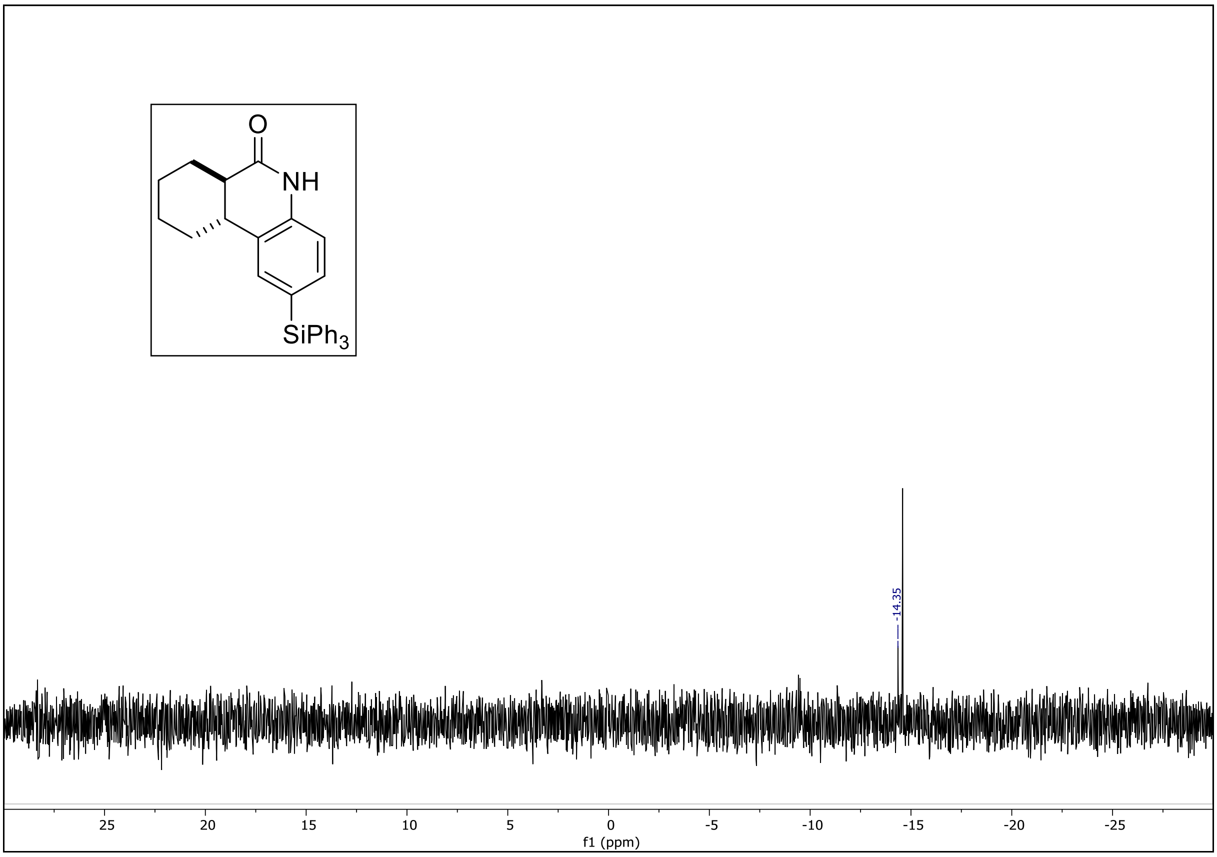

^{1}H NMR (500 MHz, $CDCl_3$): *cis*-**2g**

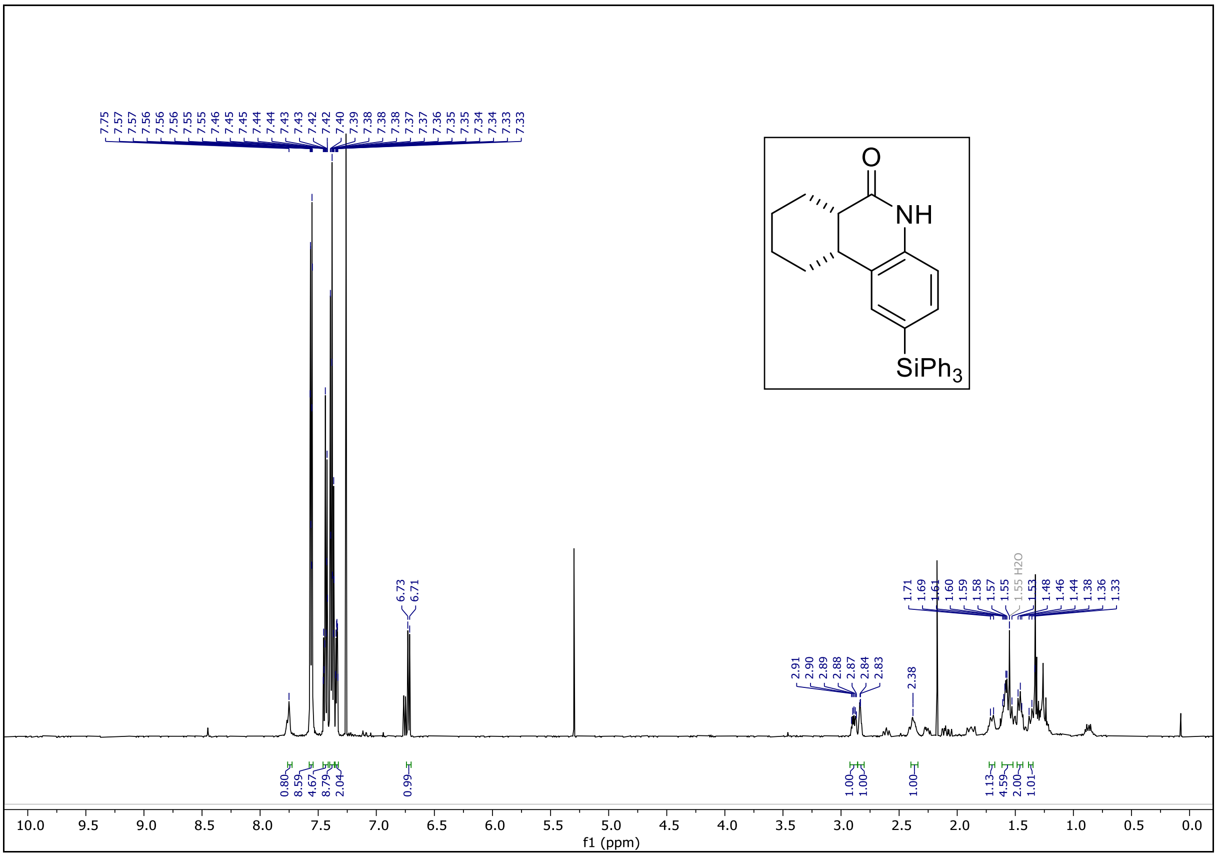

^{13}C NMR (126 MHz, $CDCl_3$): *cis*-**2g**

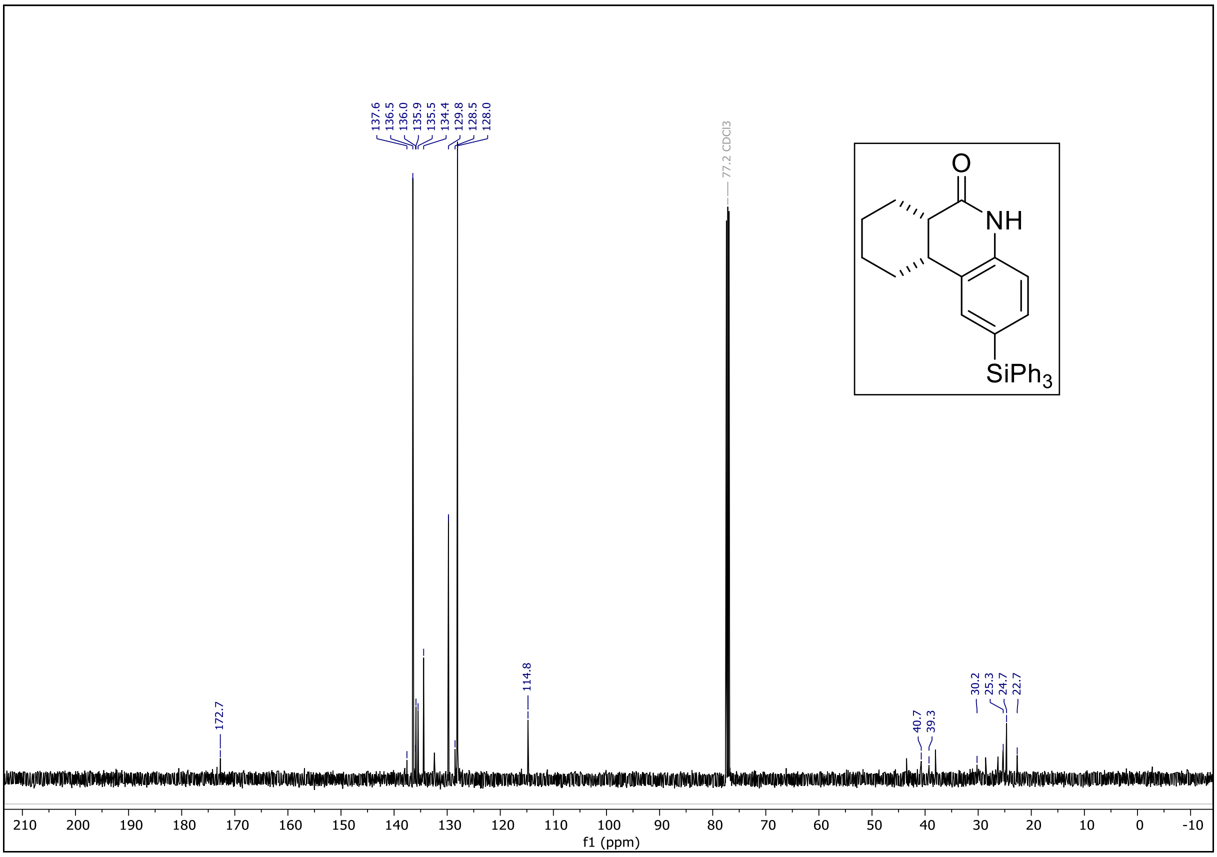

^{29}Si NMR (99 MHz, $CDCl_3$): *cis*-**2g**

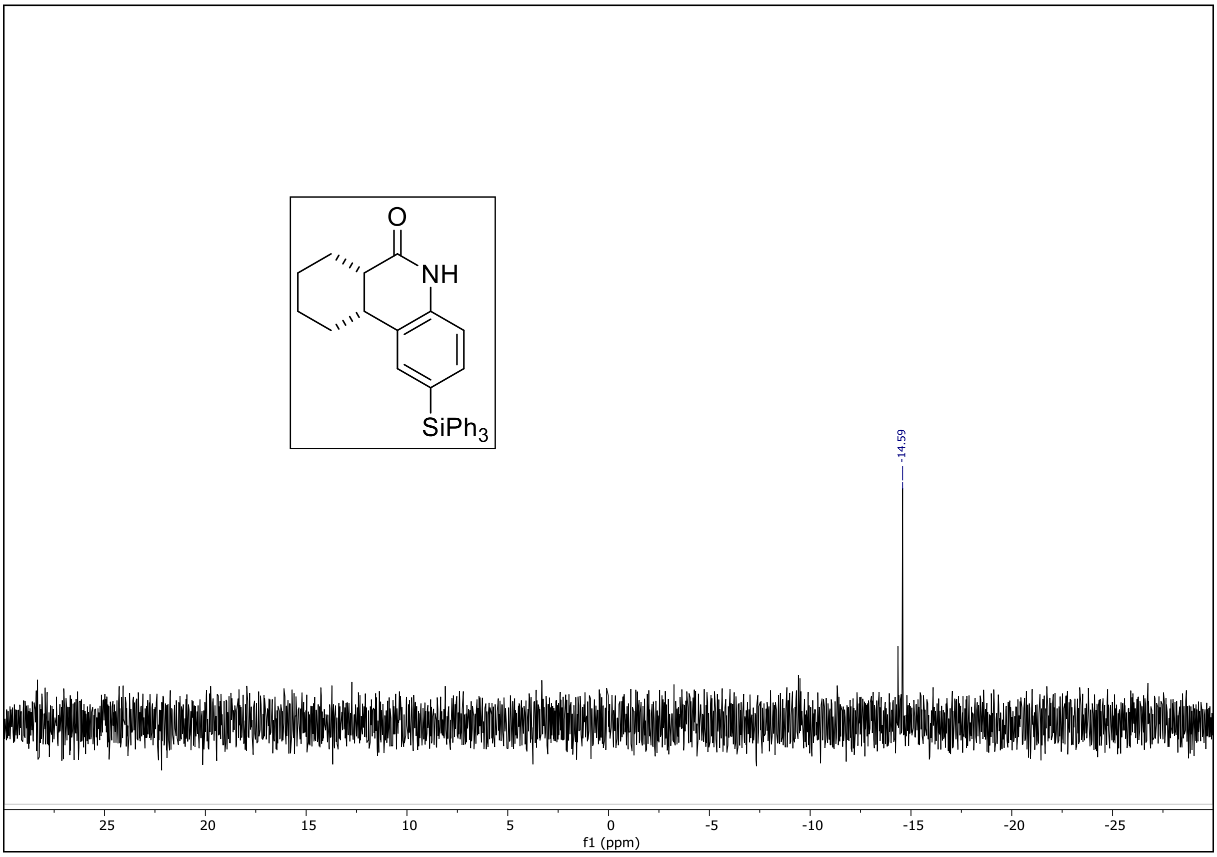

^{1}H NMR (500 MHz, $CDCl_3$): *trans*-**2h**

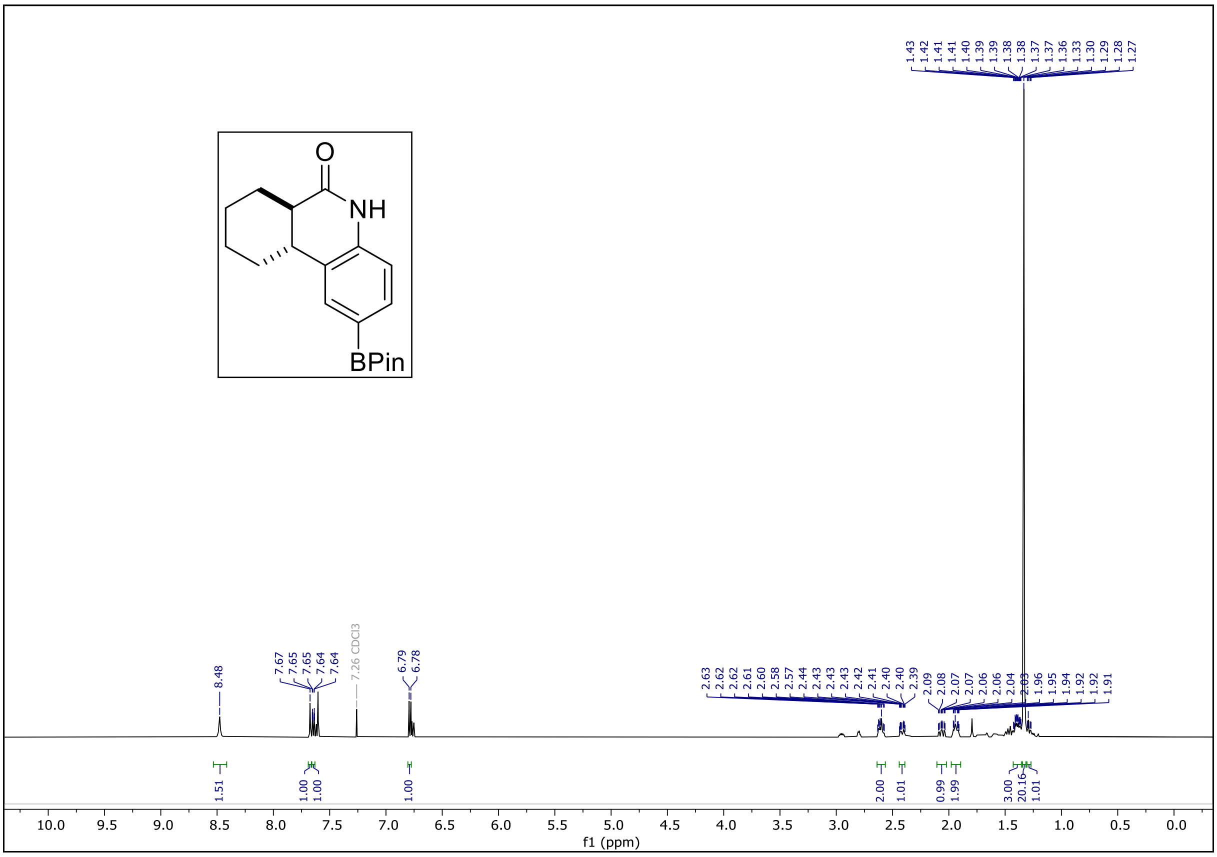

^{13}C NMR (1261 MHz, $CDCl_3$): *trans*-**2h**

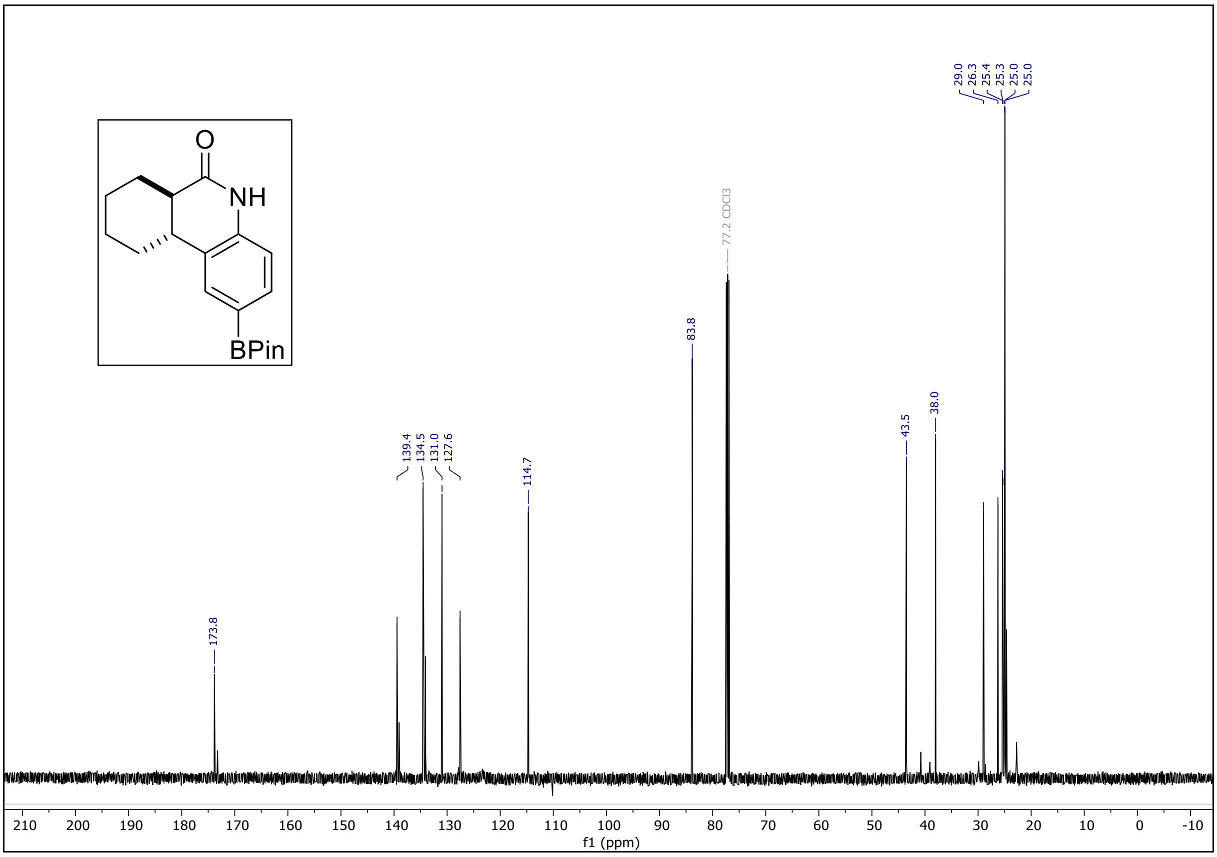

^{1}H NMR (500 MHz, $CDCl_3$): *cis*-**2h**

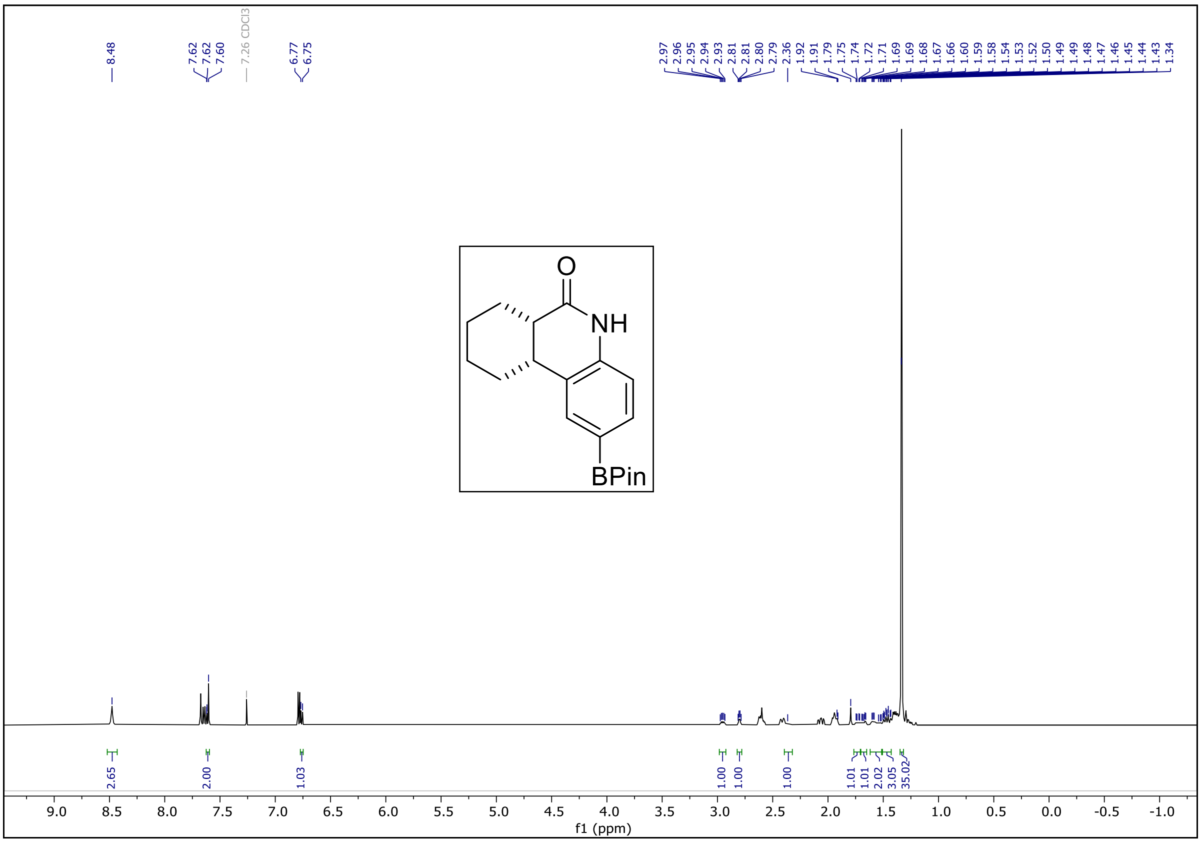

^{13}C NMR (126 MHz, $CDCl_3$): *cis*-**2h**

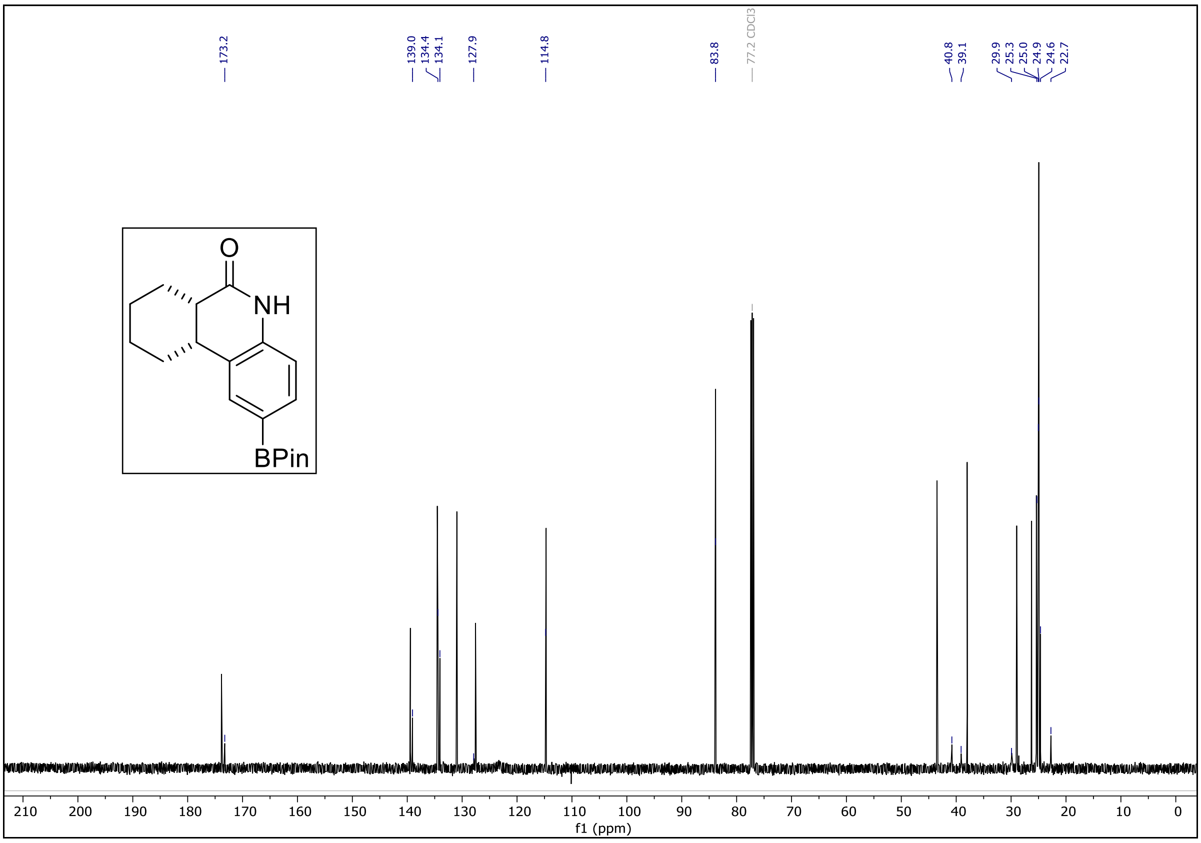

^{1}H NMR (400 MHz, $CDCl_3$): **2i**

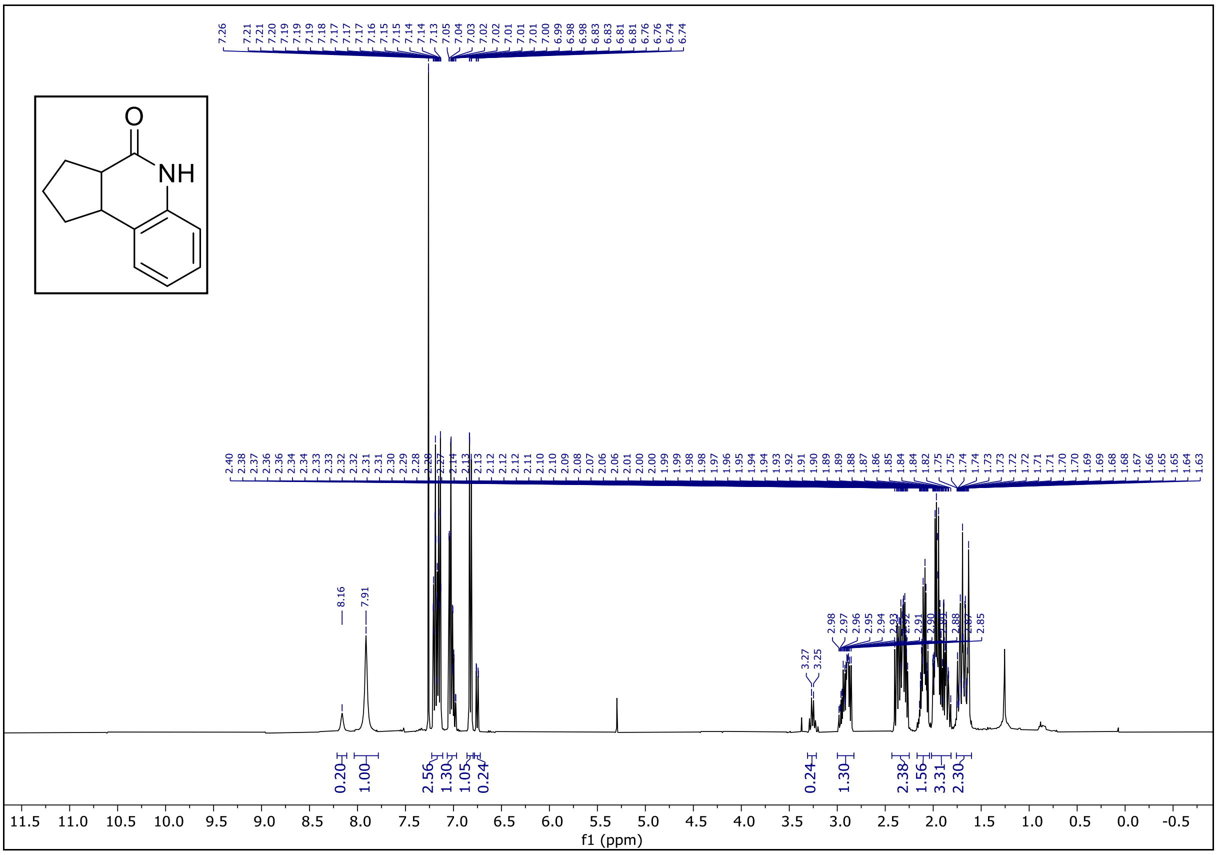

^{1}H NMR (500 MHz, $CDCl_3$): *trans*-**2j**

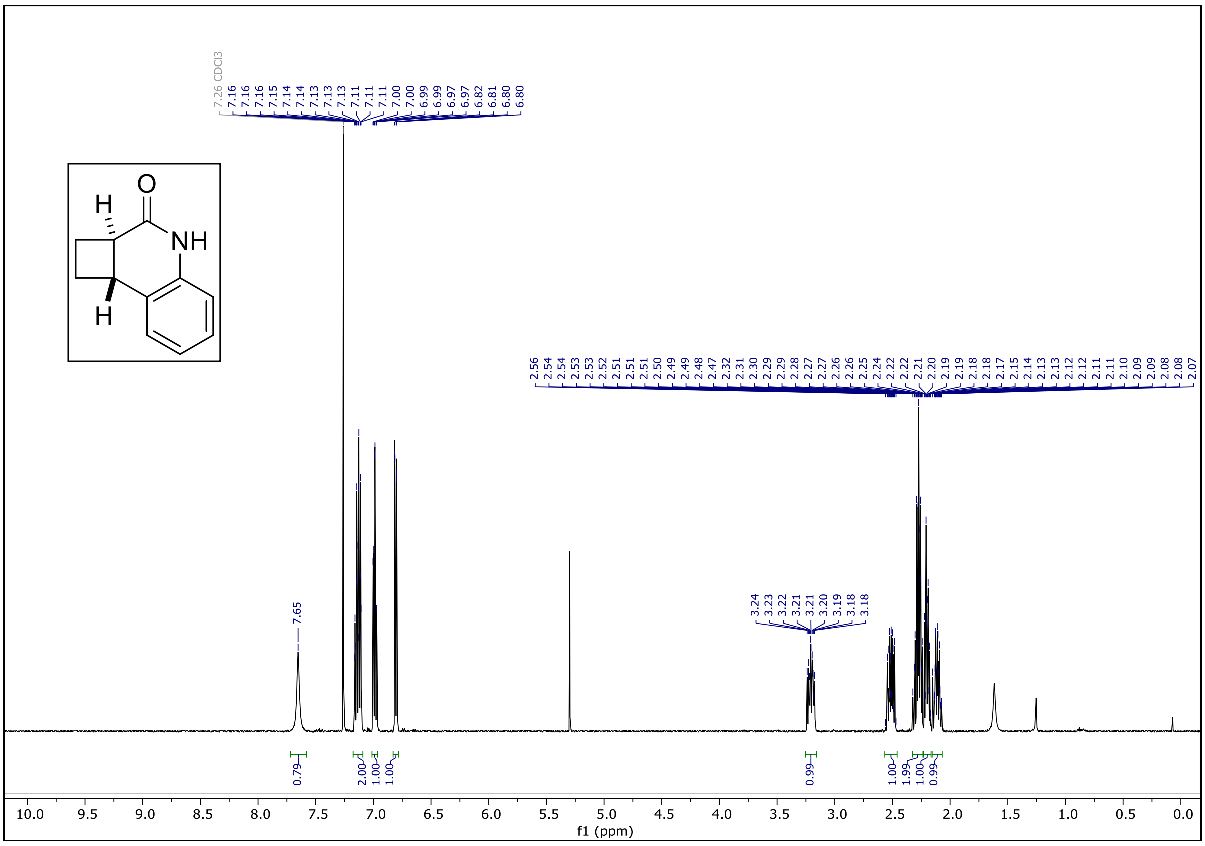

^{13}C NMR (1261 MHz, $CDCl_3$): *trans*-**2j**

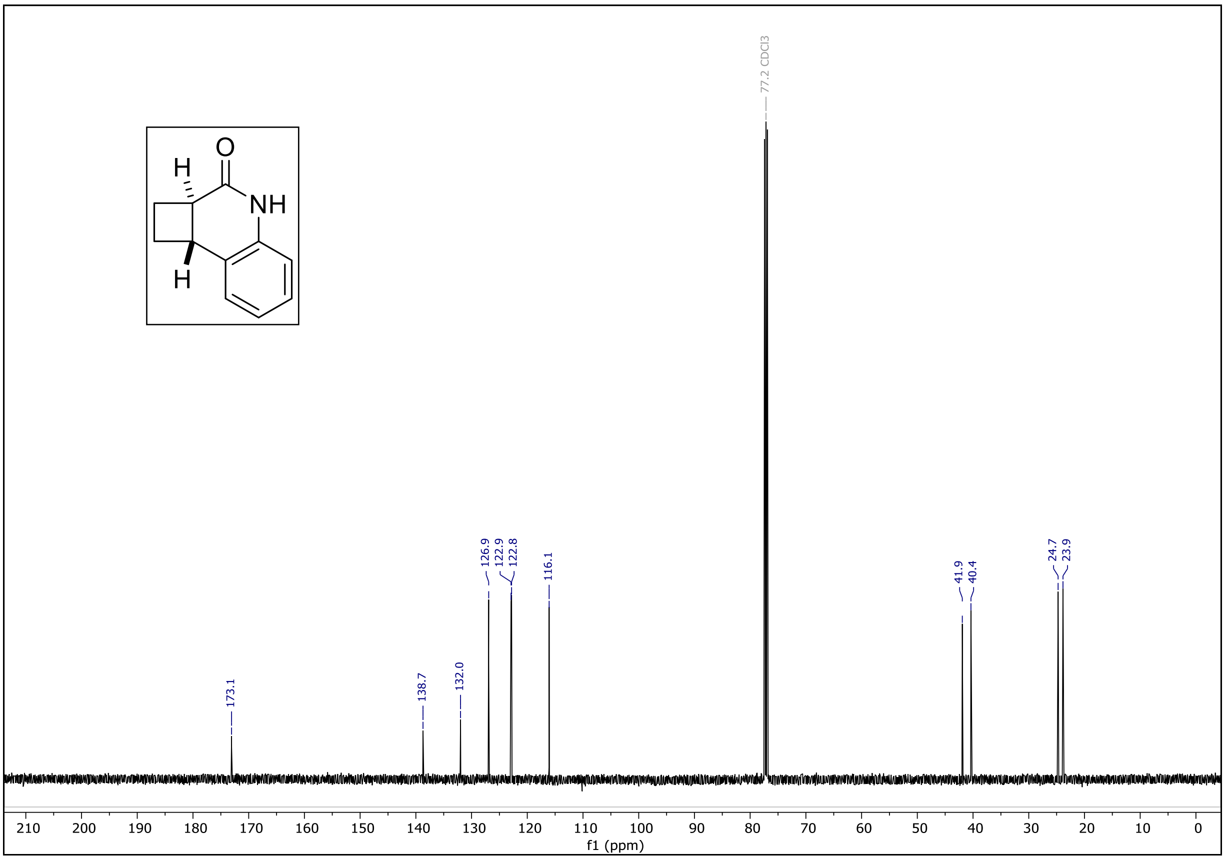

^{1}H NMR (500 MHz, $CDCl_3$): *cis*-**2j**

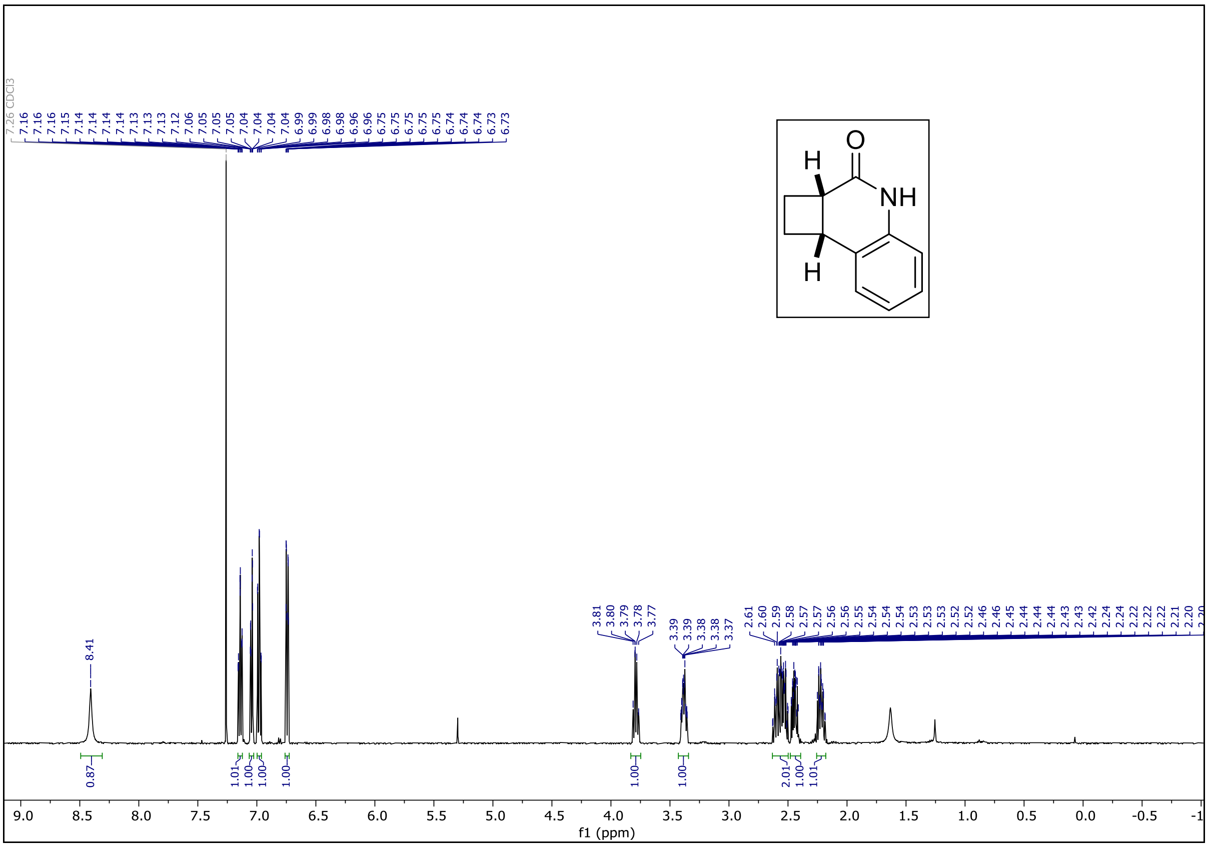

^{13}C NMR (126 MHz, $CDCl_3$): *cis*-**2j**

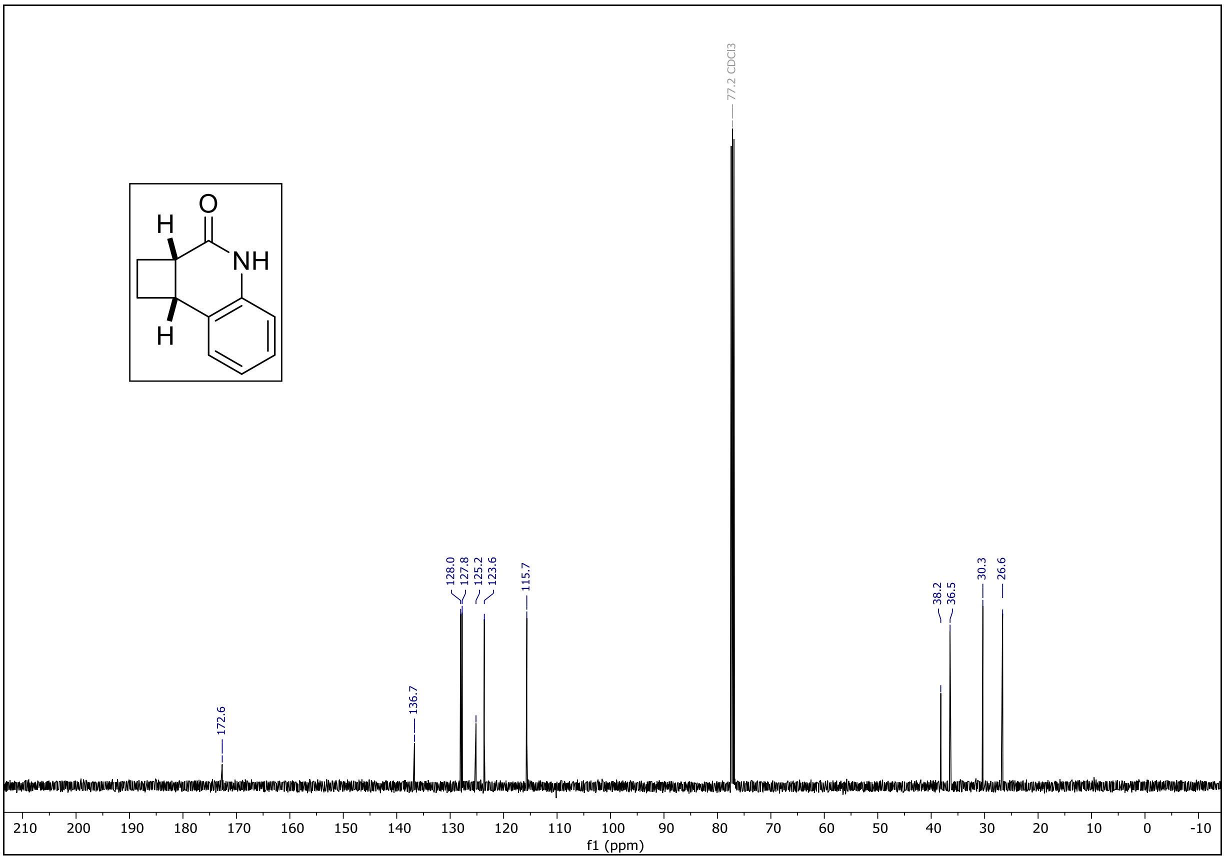

^{1}H NMR (500 MHz, $CDCl_3$): *trans*-**2k**

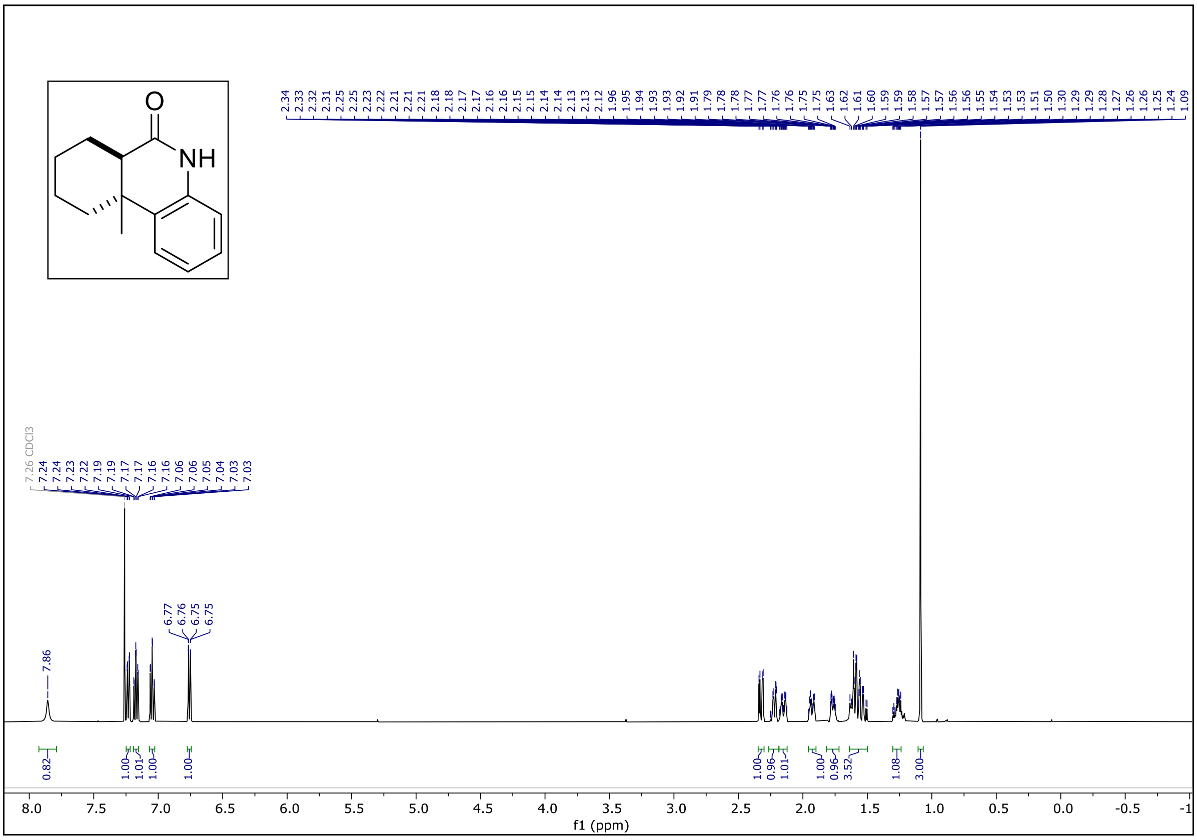

^{13}C NMR (1261 MHz, $CDCl_3$): *trans*-**2k**

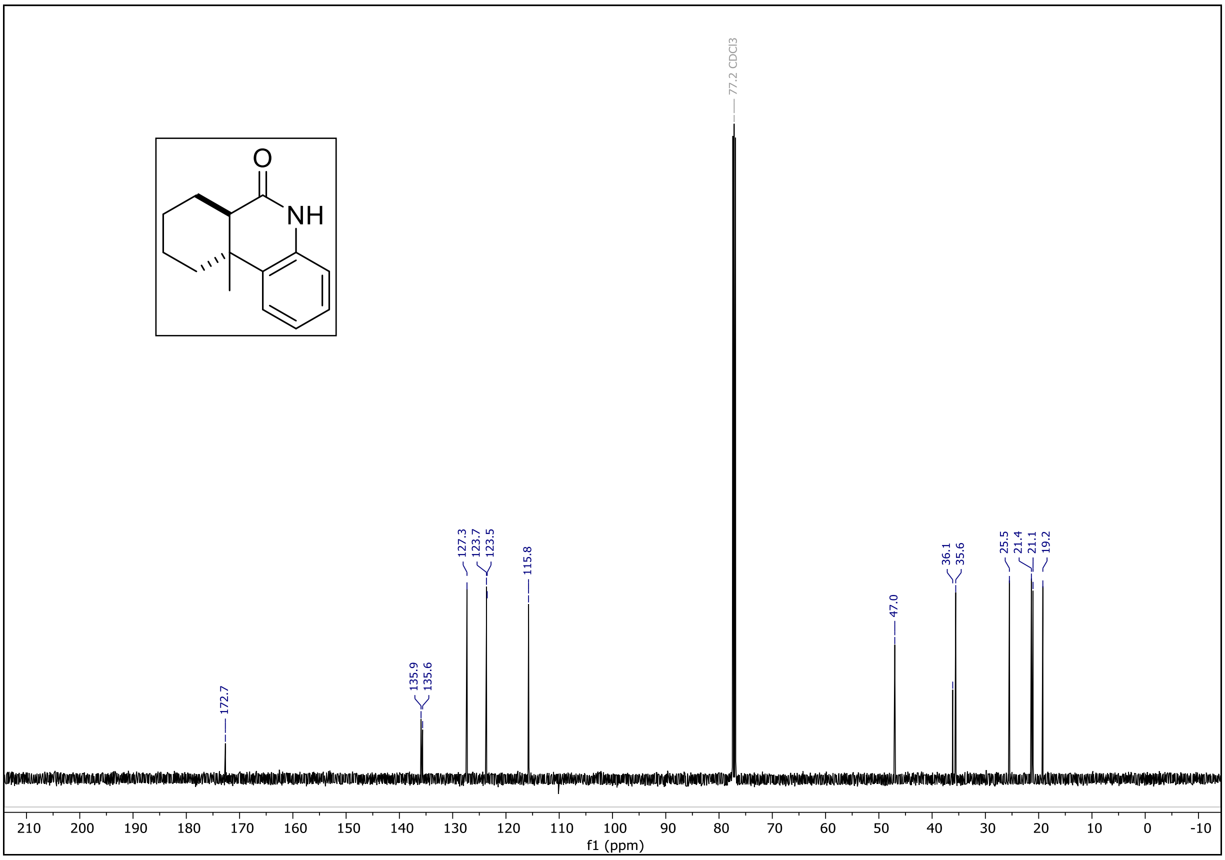

^{1}H NMR (500 MHz, $CDCl_3$): *trans*-**2l**

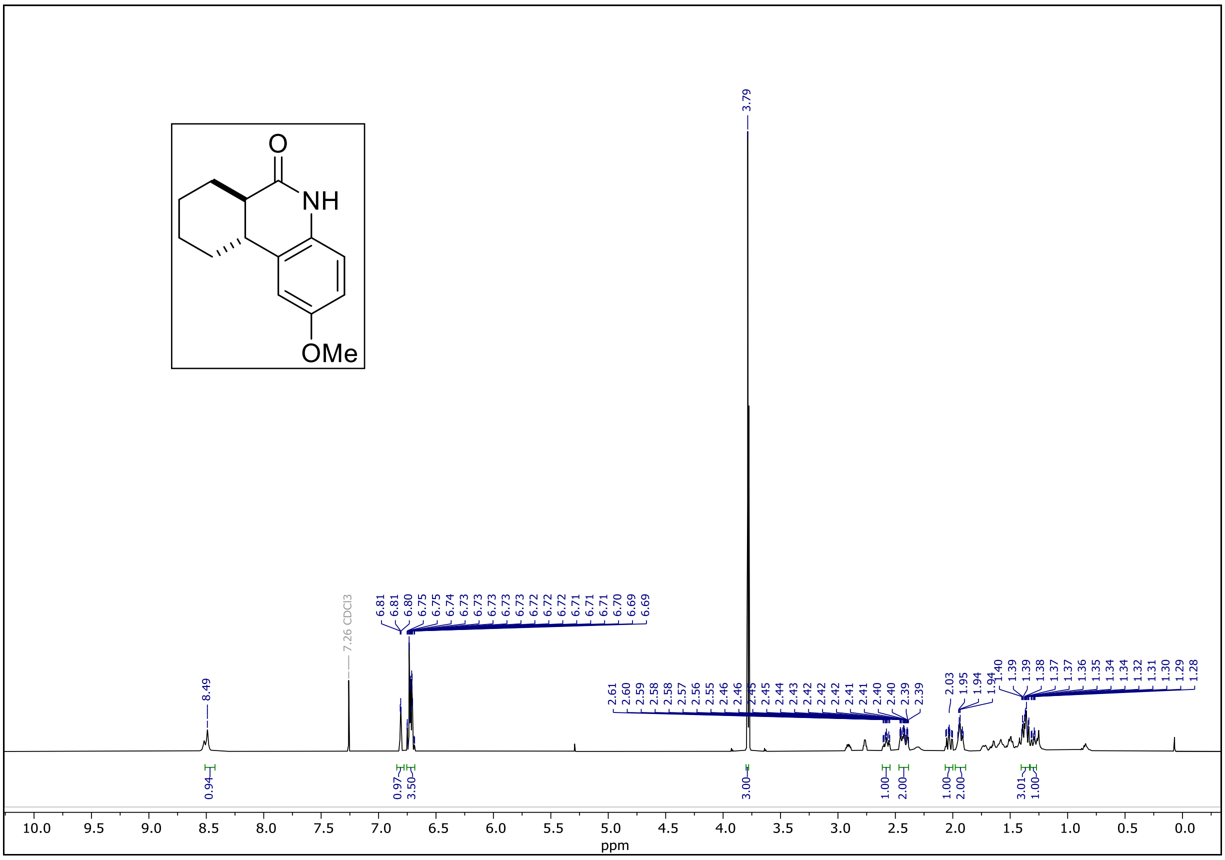

^{13}C NMR (126 MHz, $CDCl_3$): *trans*-**2l**

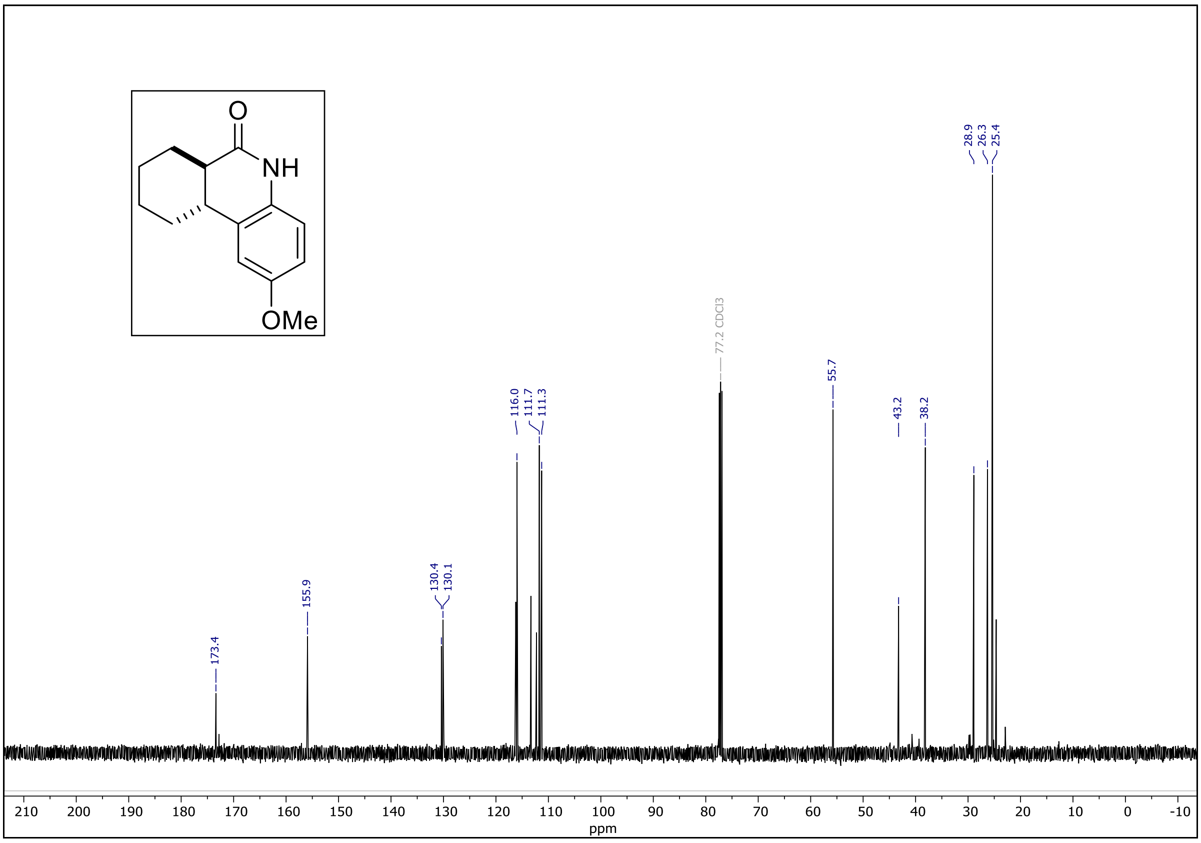

^{1}H NMR (500 MHz, $CDCl_3$): *cis*-**2l**

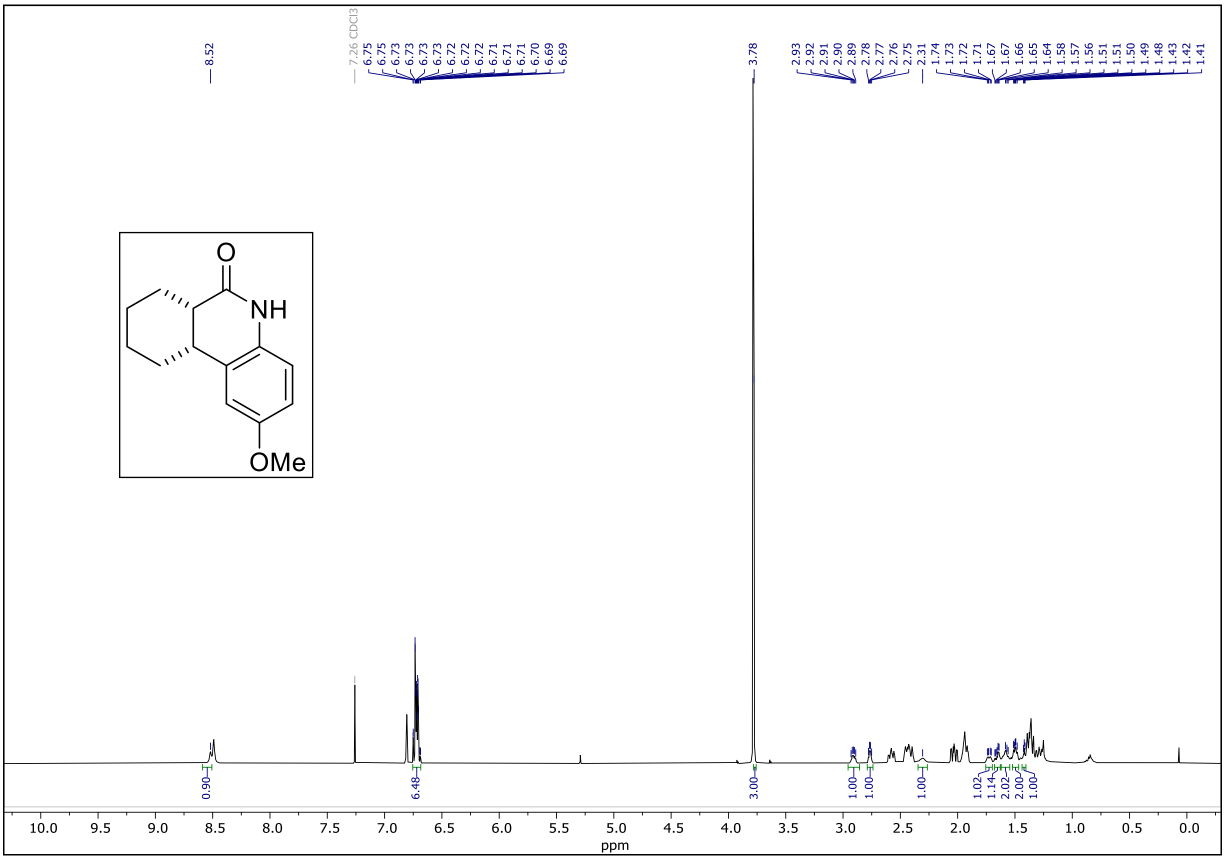

^{13}C NMR (126 MHz, $CDCl_3$): *cis*-**2l**

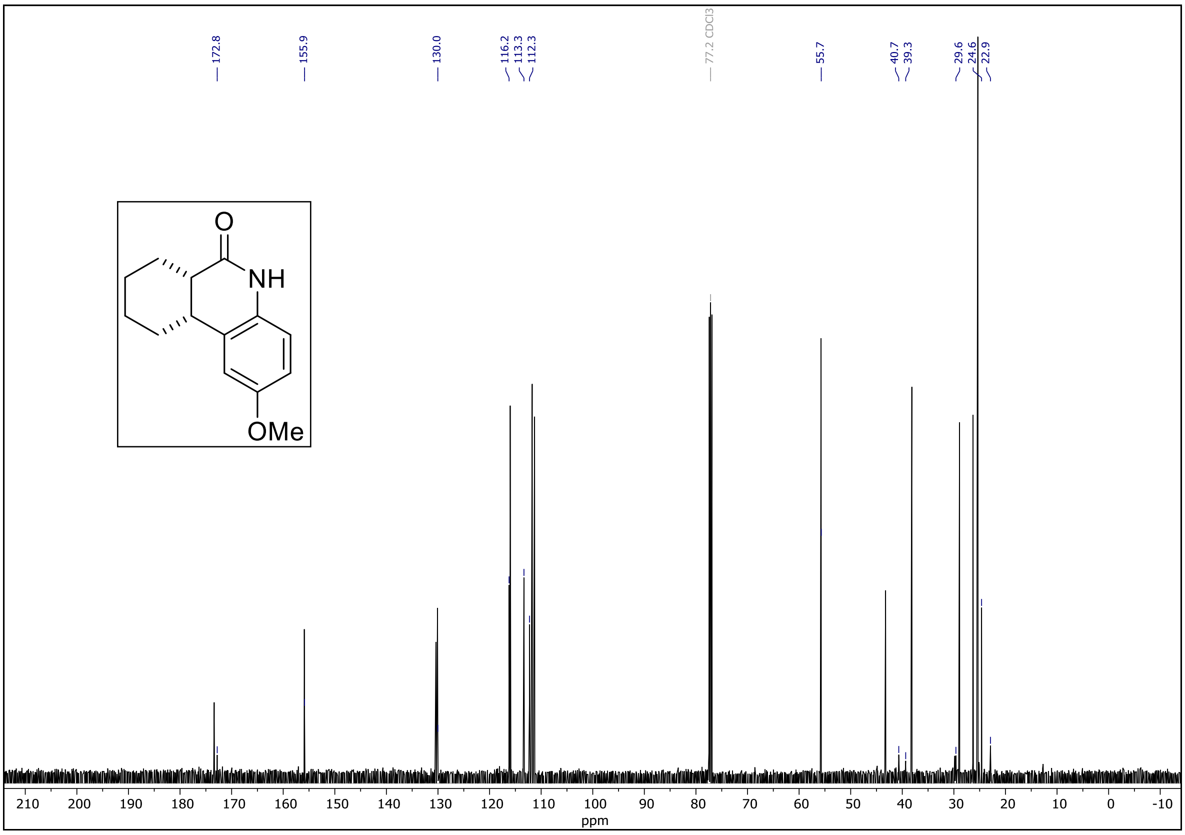

^{1}H NMR (600 MHz, $CDCl_3$): *trans*-**2m**

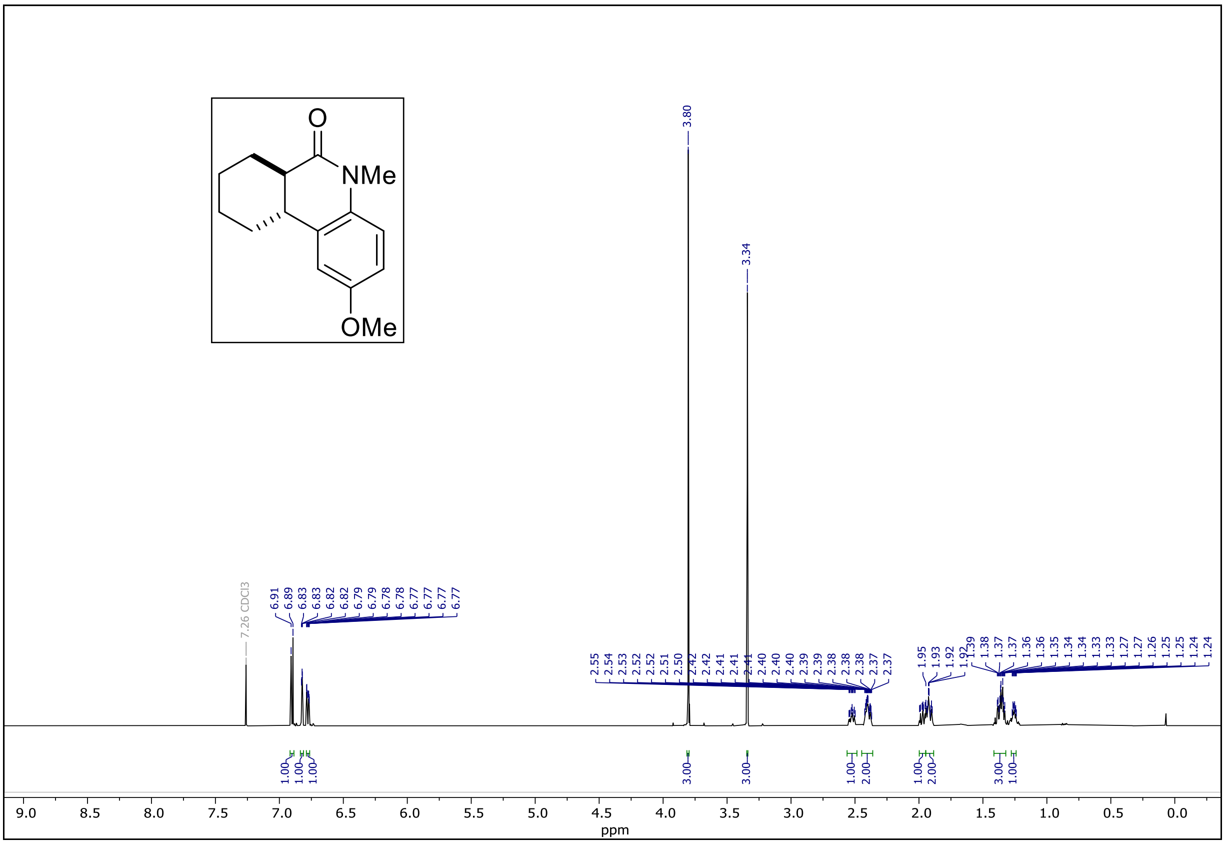

^{13}C NMR (151 MHz, $CDCl_3$): *trans*-**2m**

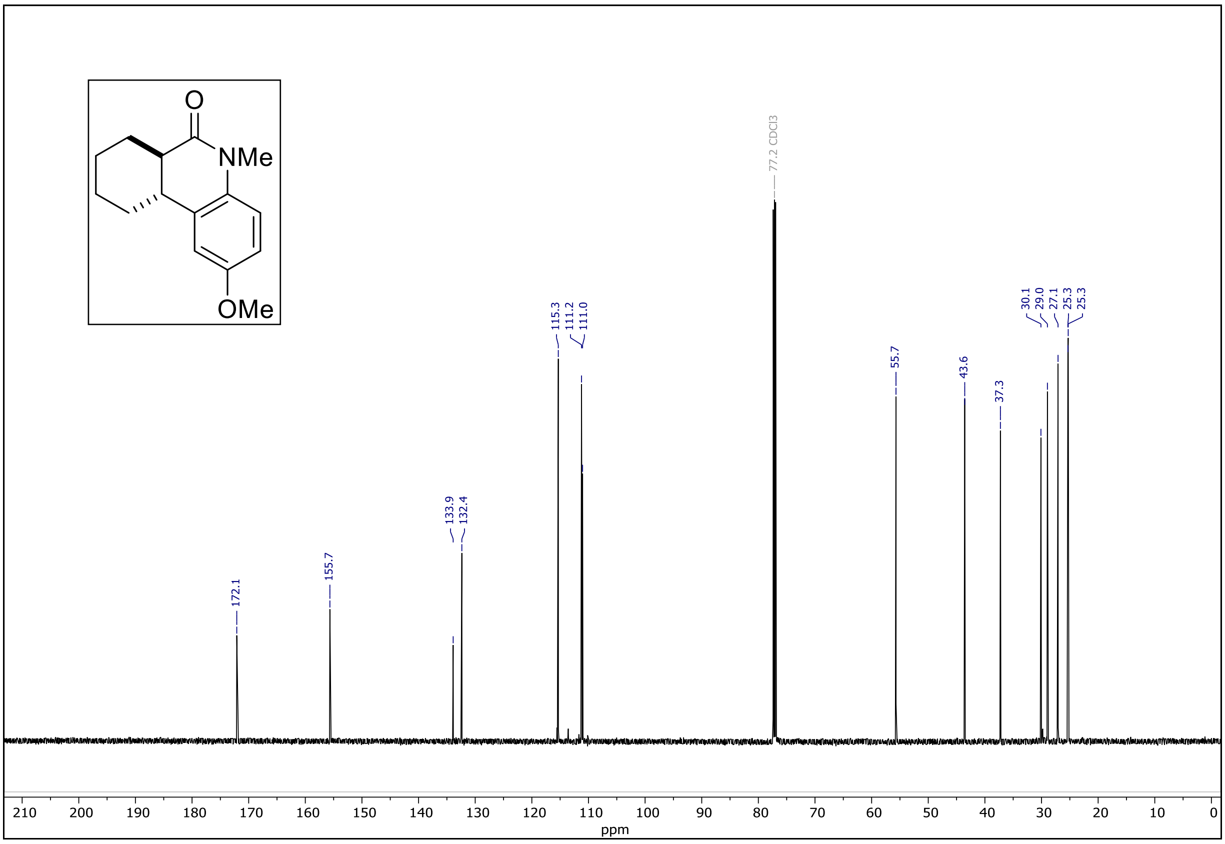

^{1}H NMR (600 MHz, $CDCl_3$): *cis*-**2m**

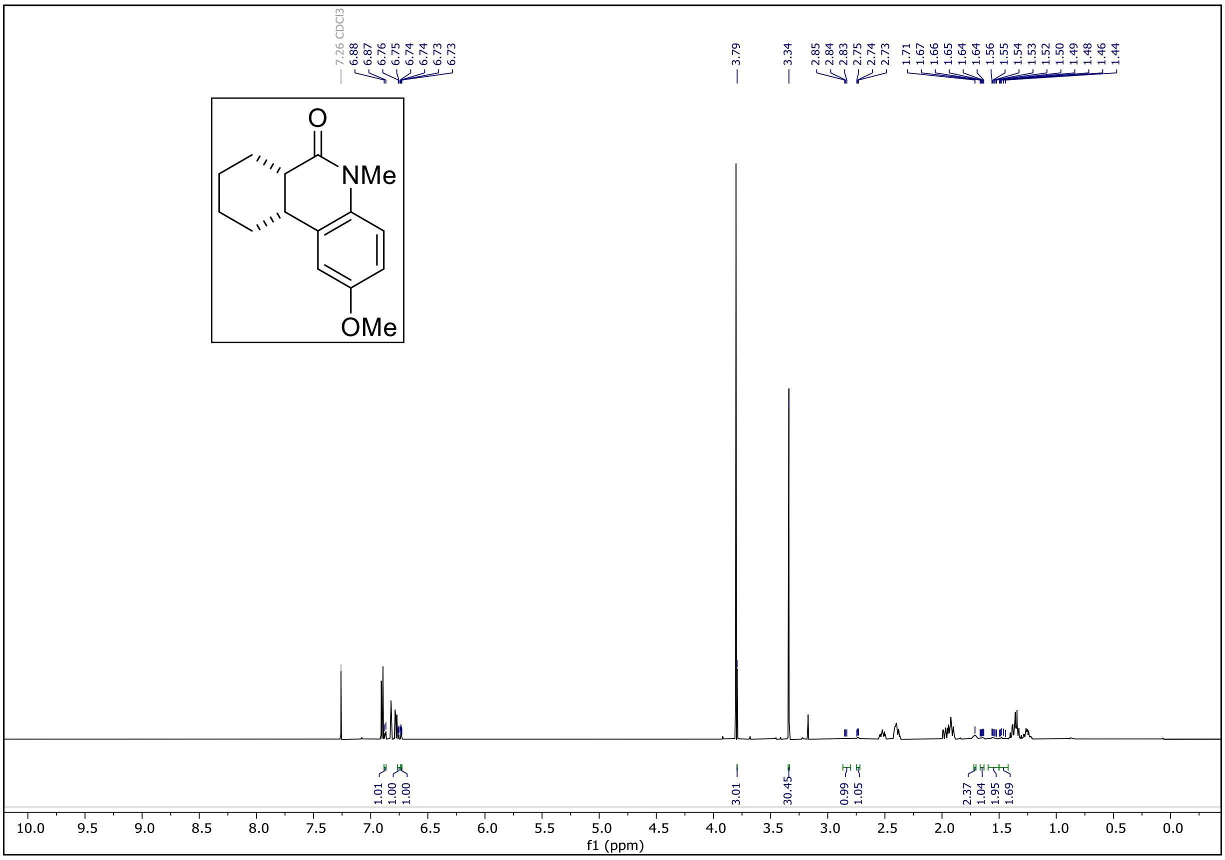

^{13}C NMR (151 MHz, $CDCl_3$): *cis*-**2m**

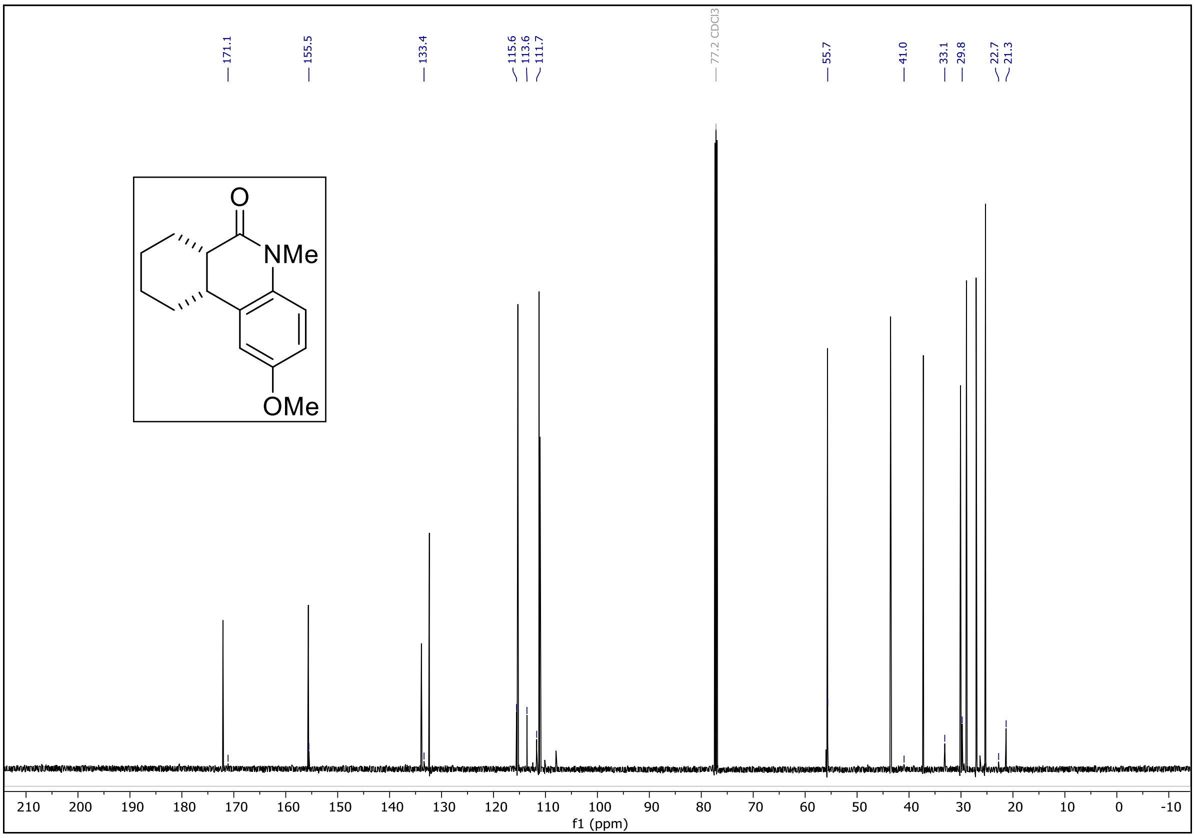

^{1}H NMR (500 MHz, $CDCl_3$): *trans*-**2n**

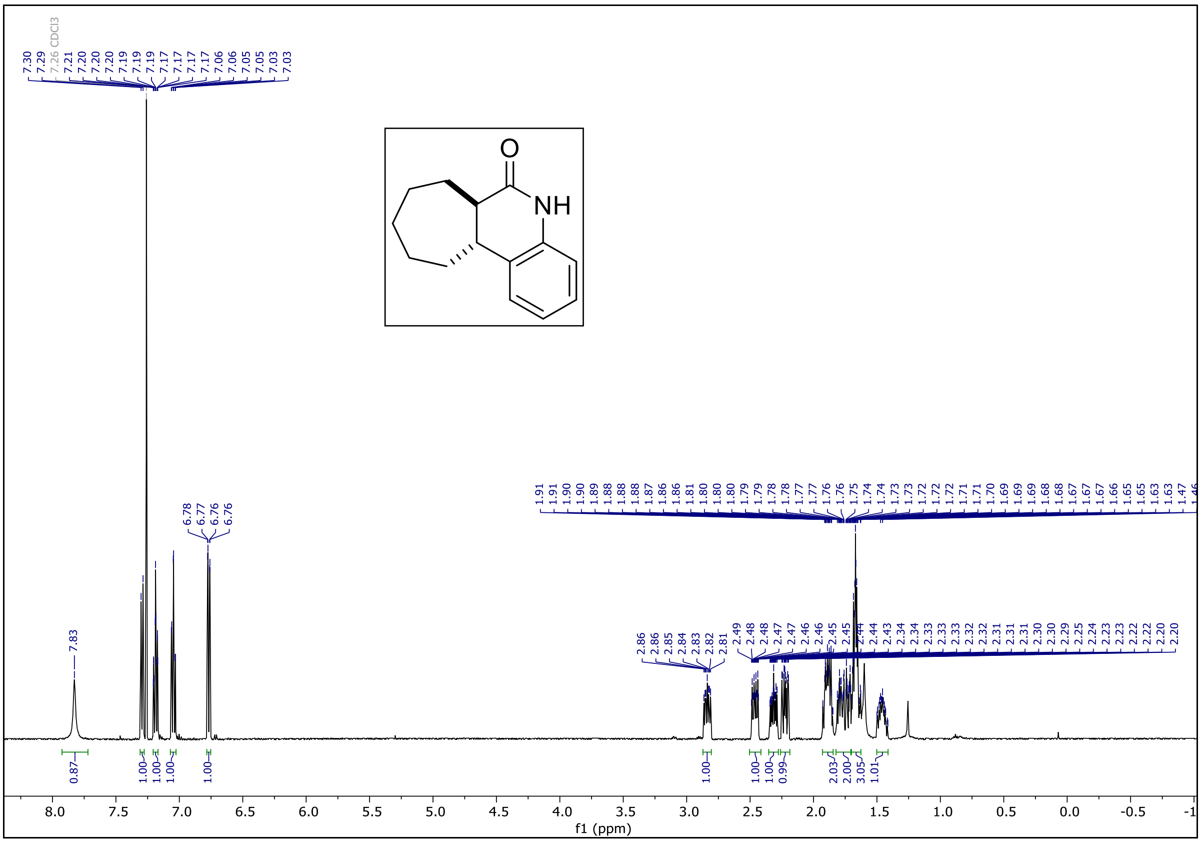

^{13}C NMR (1261 MHz, CDCl$_3$): *trans*-**2n**

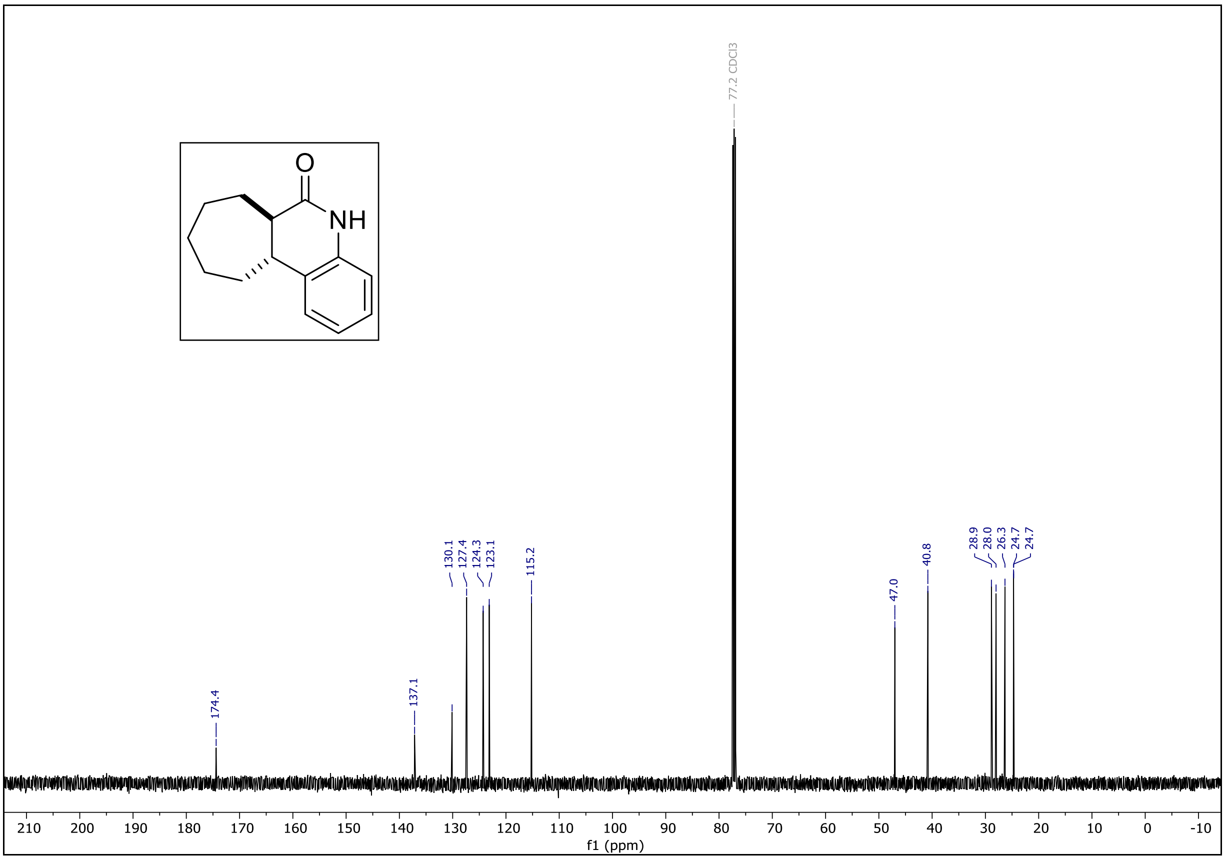

^{1}H NMR (500 MHz, CDCl$_3$): *cis*-**2n**

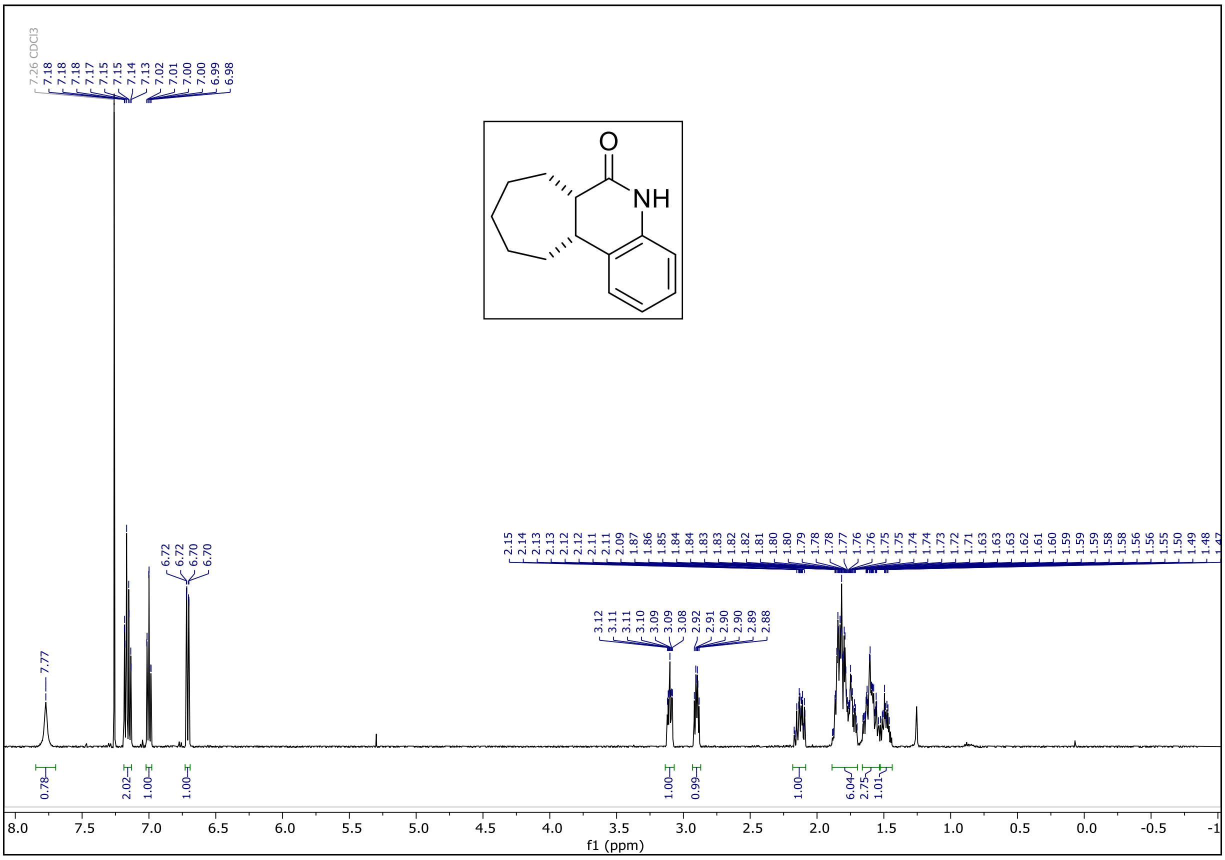

^{13}C NMR (126 MHz, $CDCl_3$): *cis*-**2n**

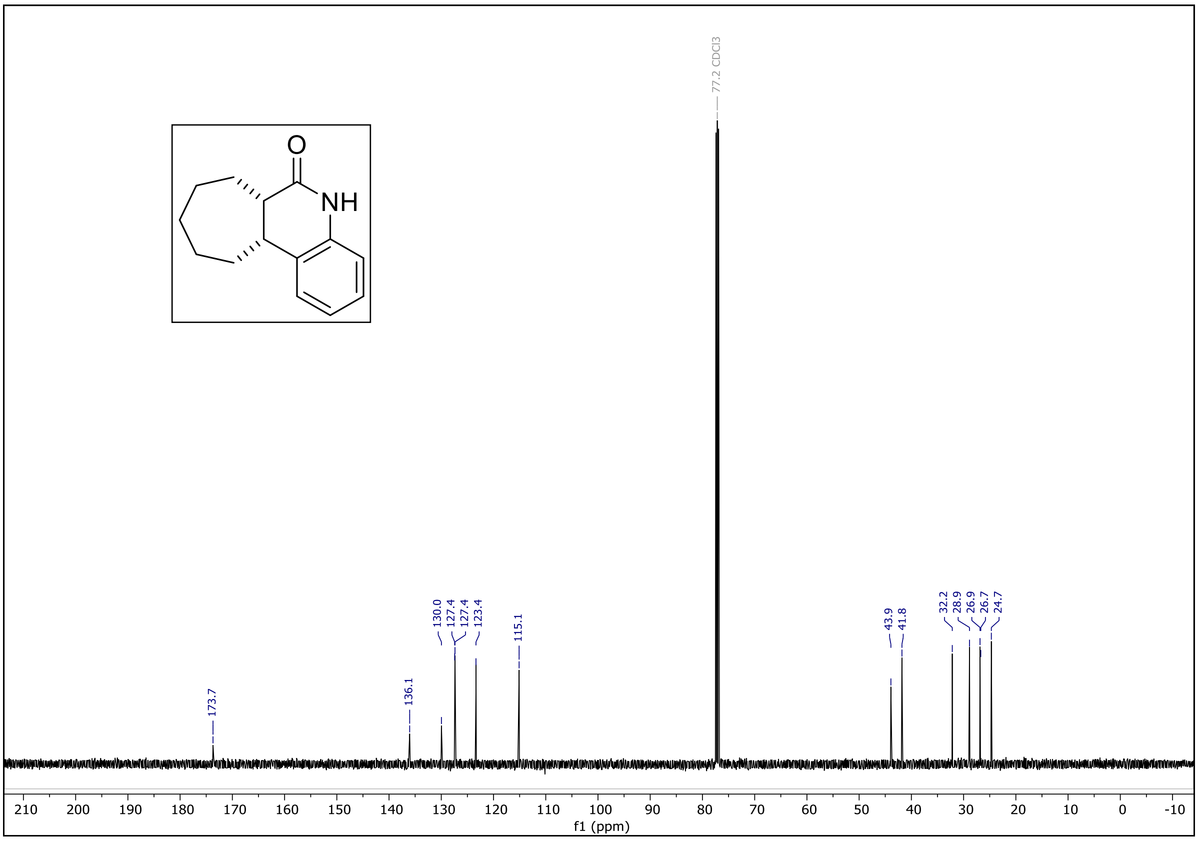

^{1}H NMR (500 MHz, $CDCl_3$): *trans*-**2o**

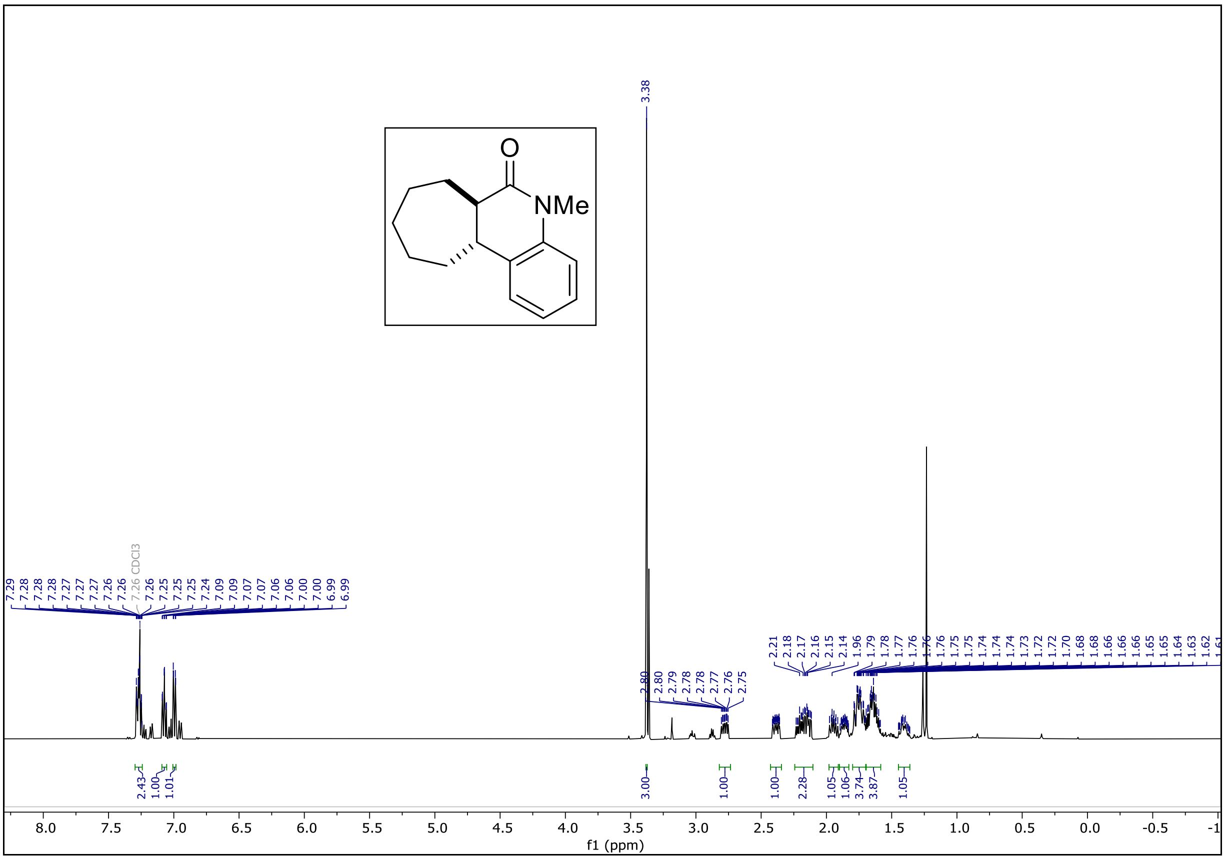

^{13}C NMR (1261 MHz, $CDCl_3$): *trans*-**2o**

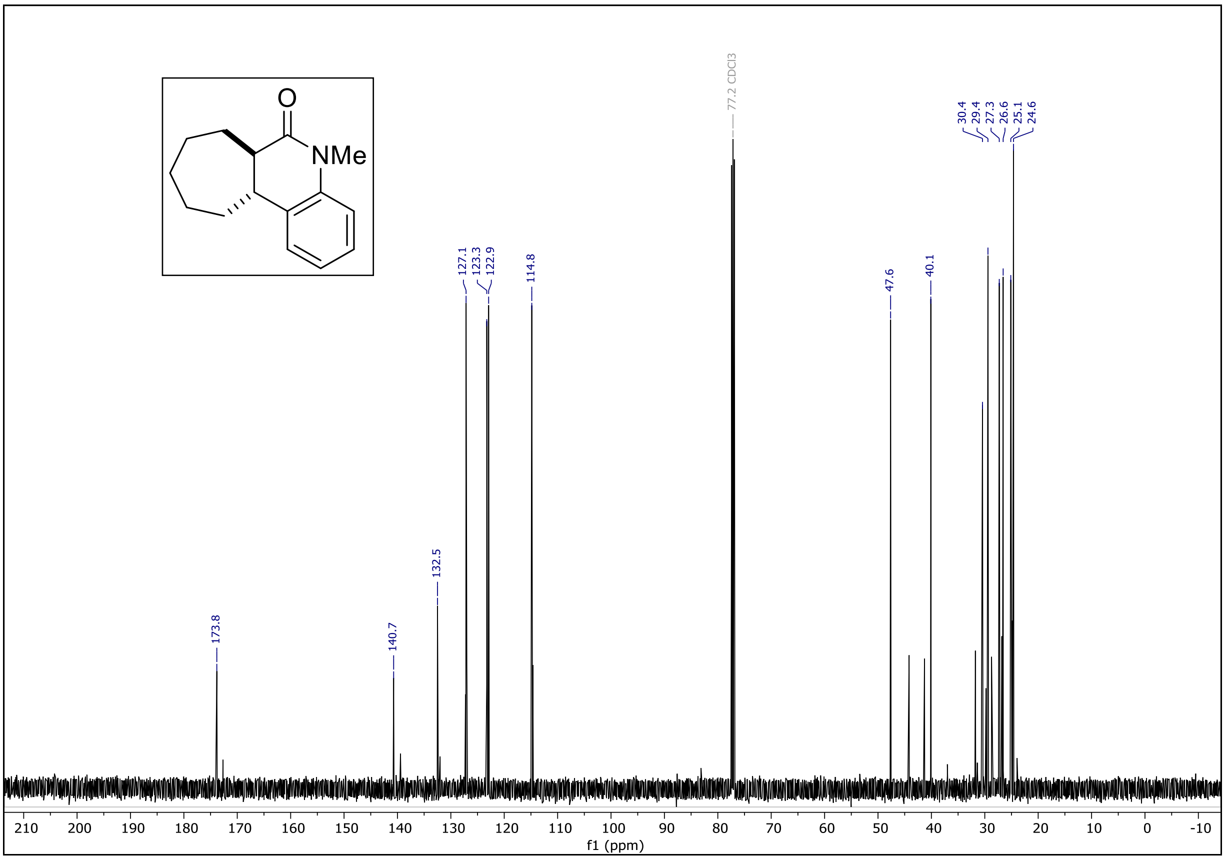

^{1}H NMR (500 MHz, $CDCl_3$): *cis*-**2o**

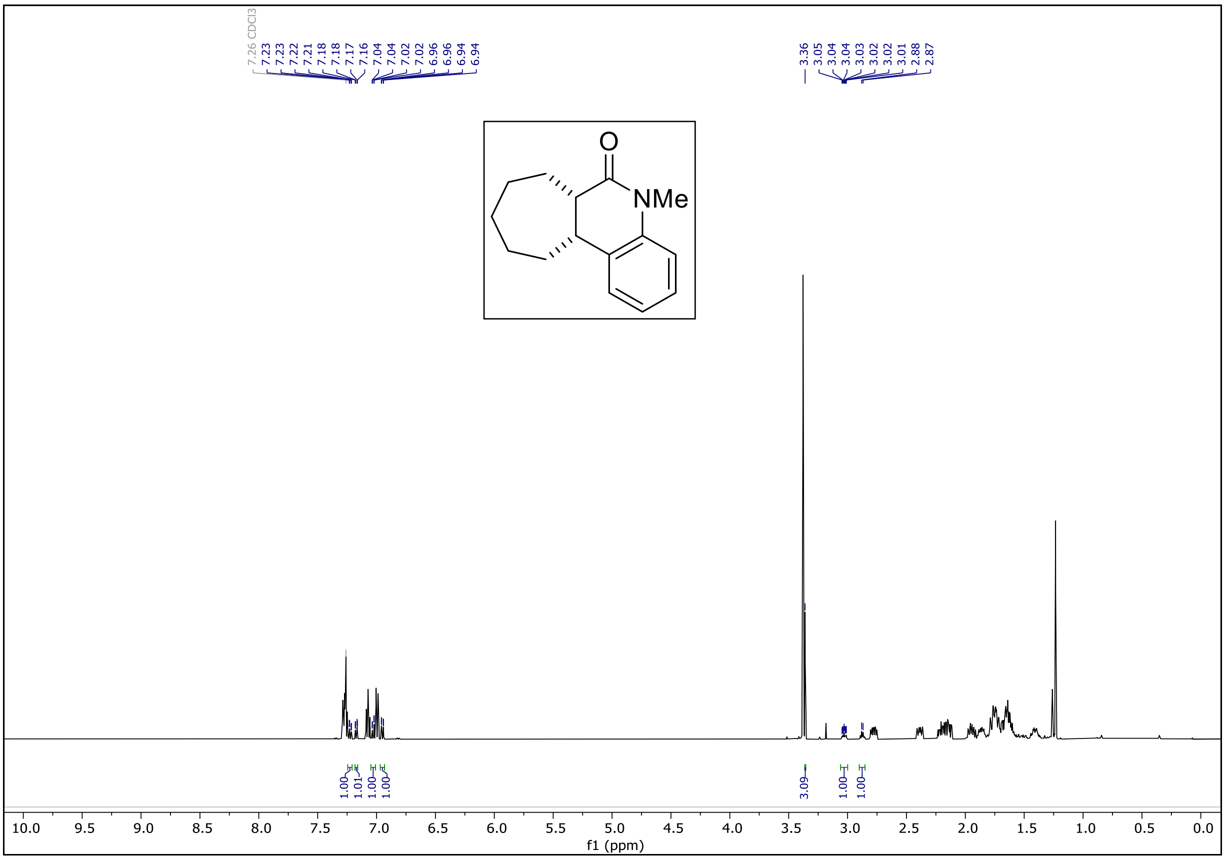

^{13}C NMR (126 MHz, $CDCl_3$): *cis*-**2o**

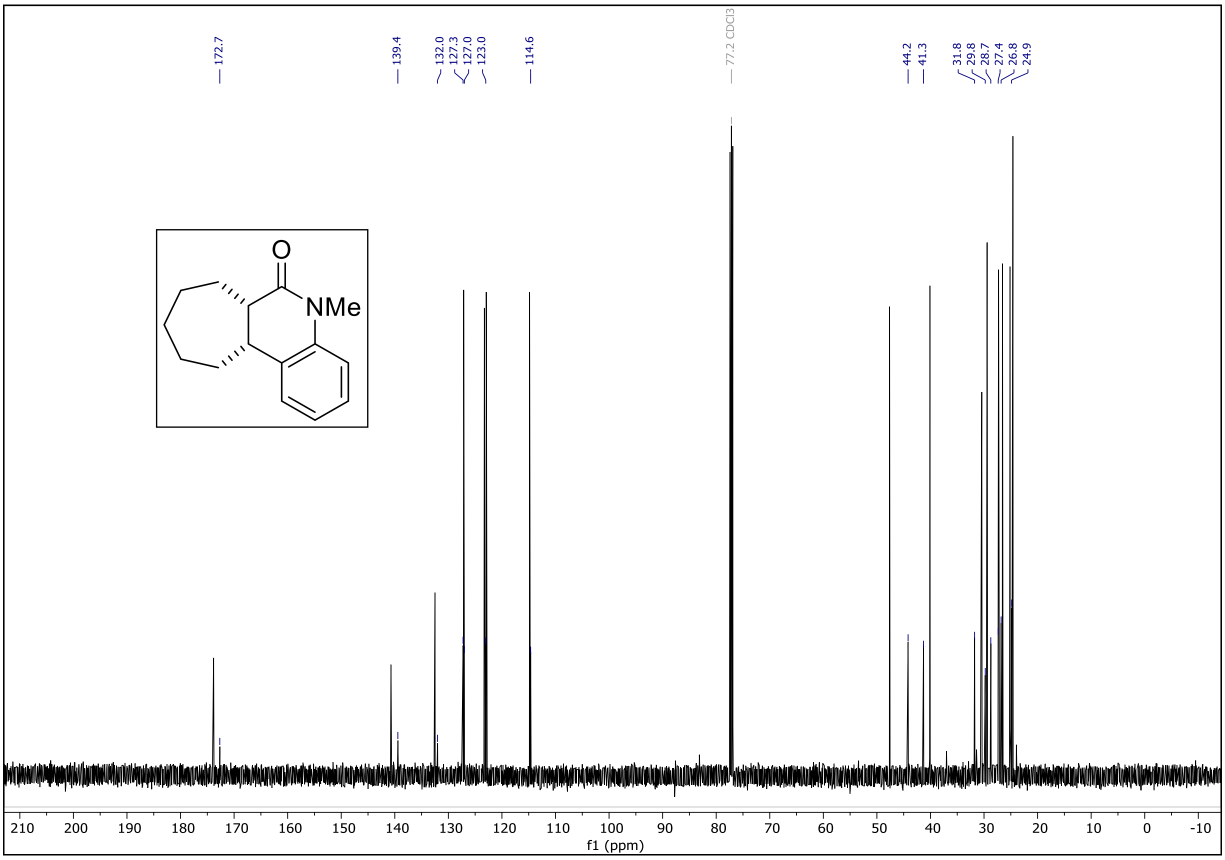

^{1}H NMR (400 MHz, $CDCl_3$): **2p**

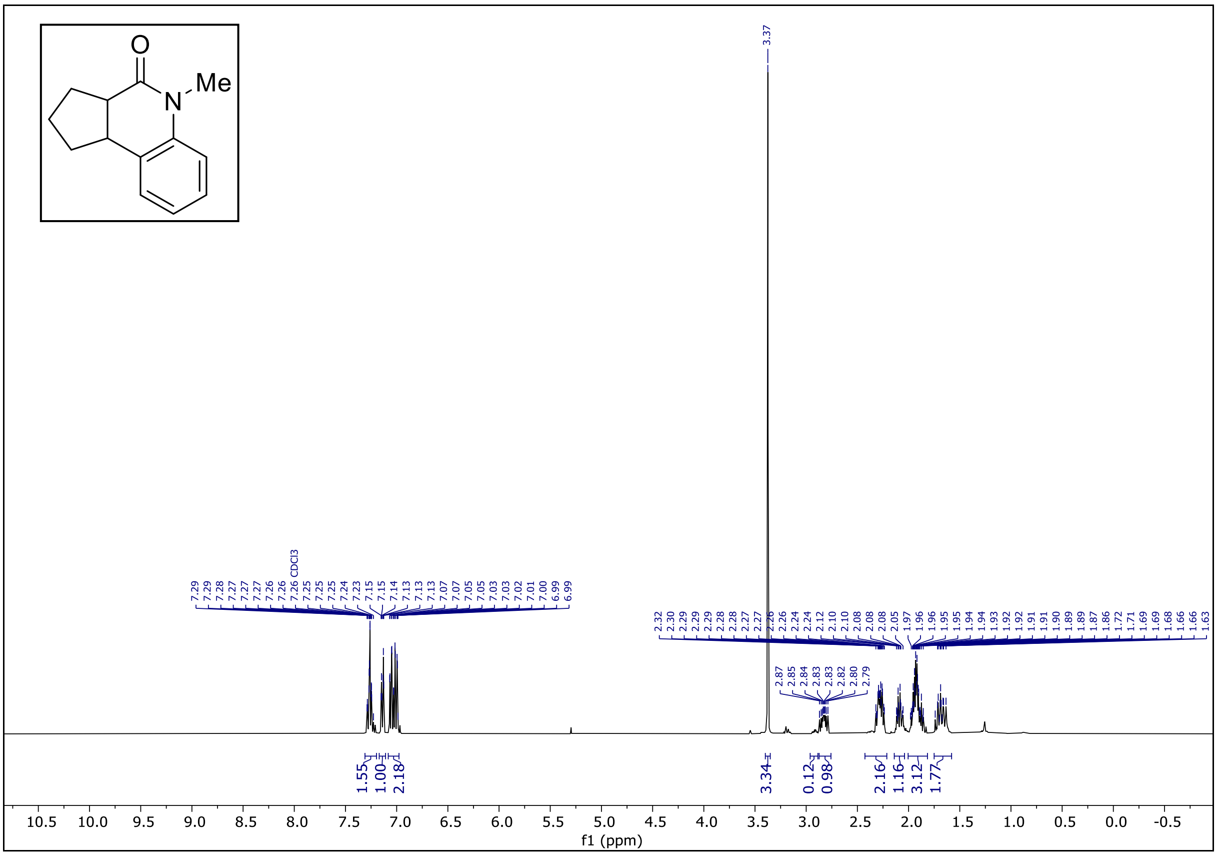

^{1}H NMR (400 MHz, $CDCl_3$): **2q**

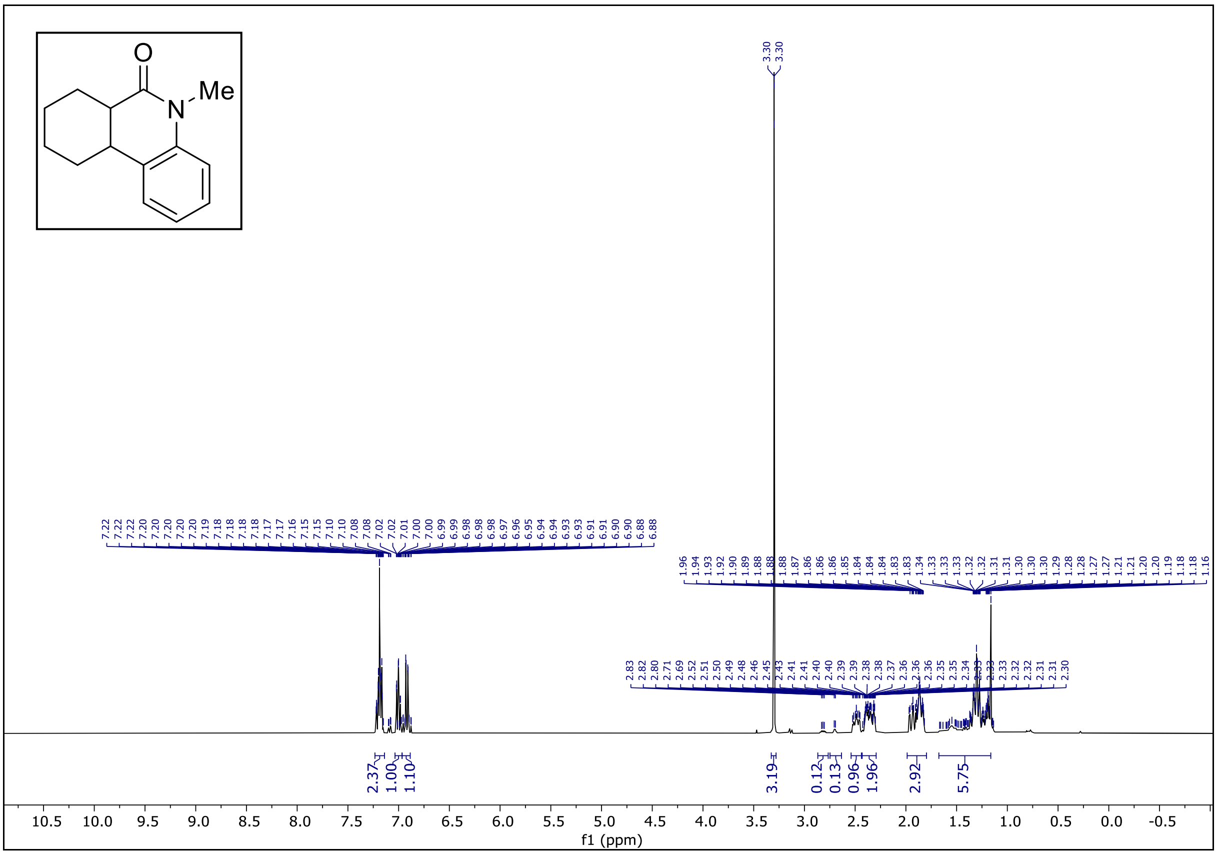

^{1}H NMR (600 MHz, $CDCl_3$): *trans*-**2r**

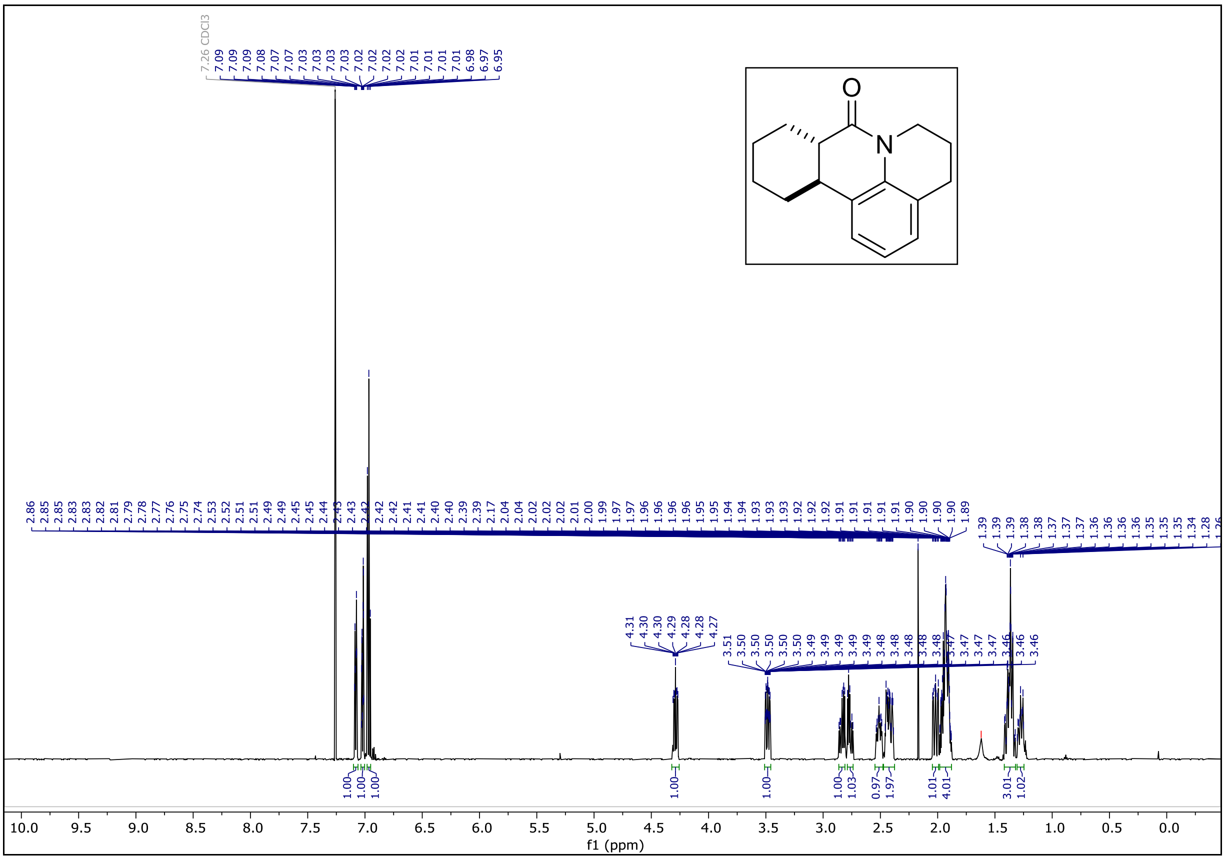

^{13}C NMR (151 MHz, $CDCl_3$): *trans*-**2r**

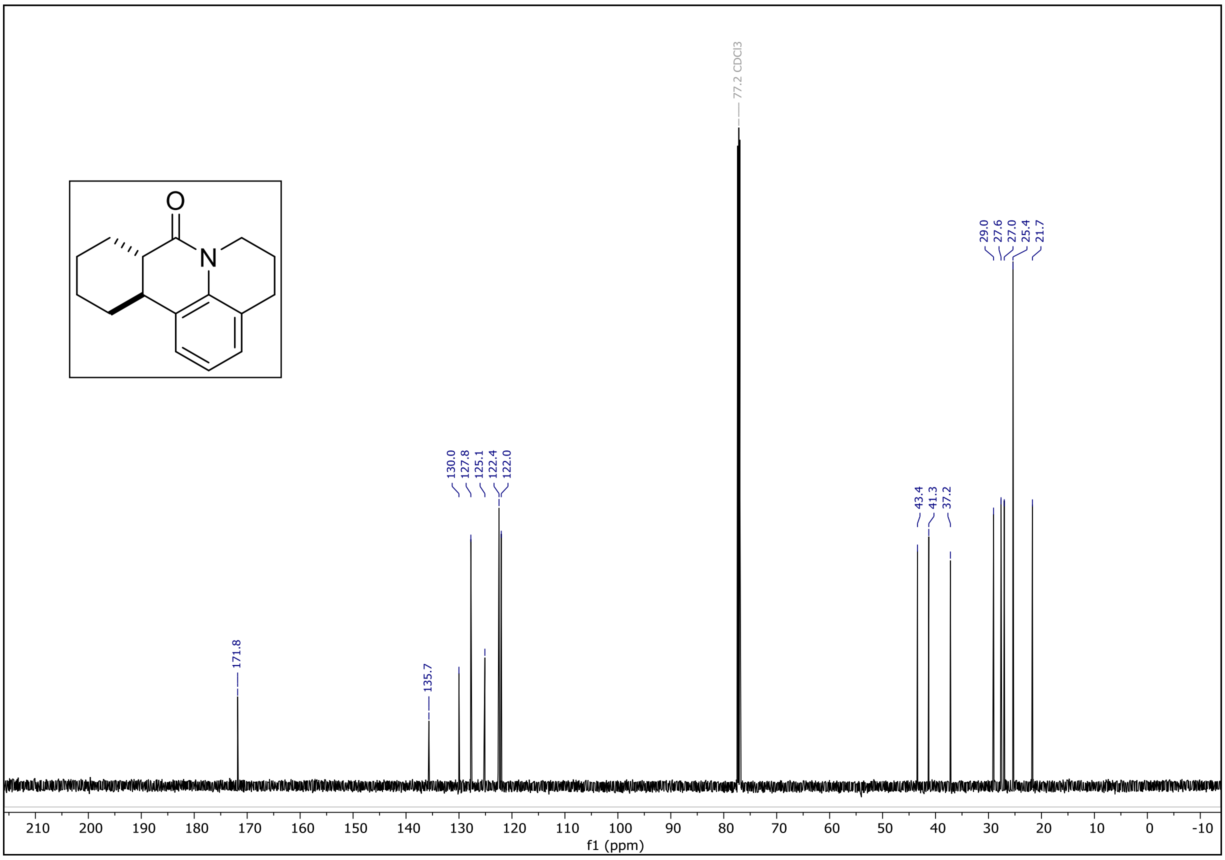

^{1}H NMR (500 MHz, $CDCl_3$): *trans*-**2s**

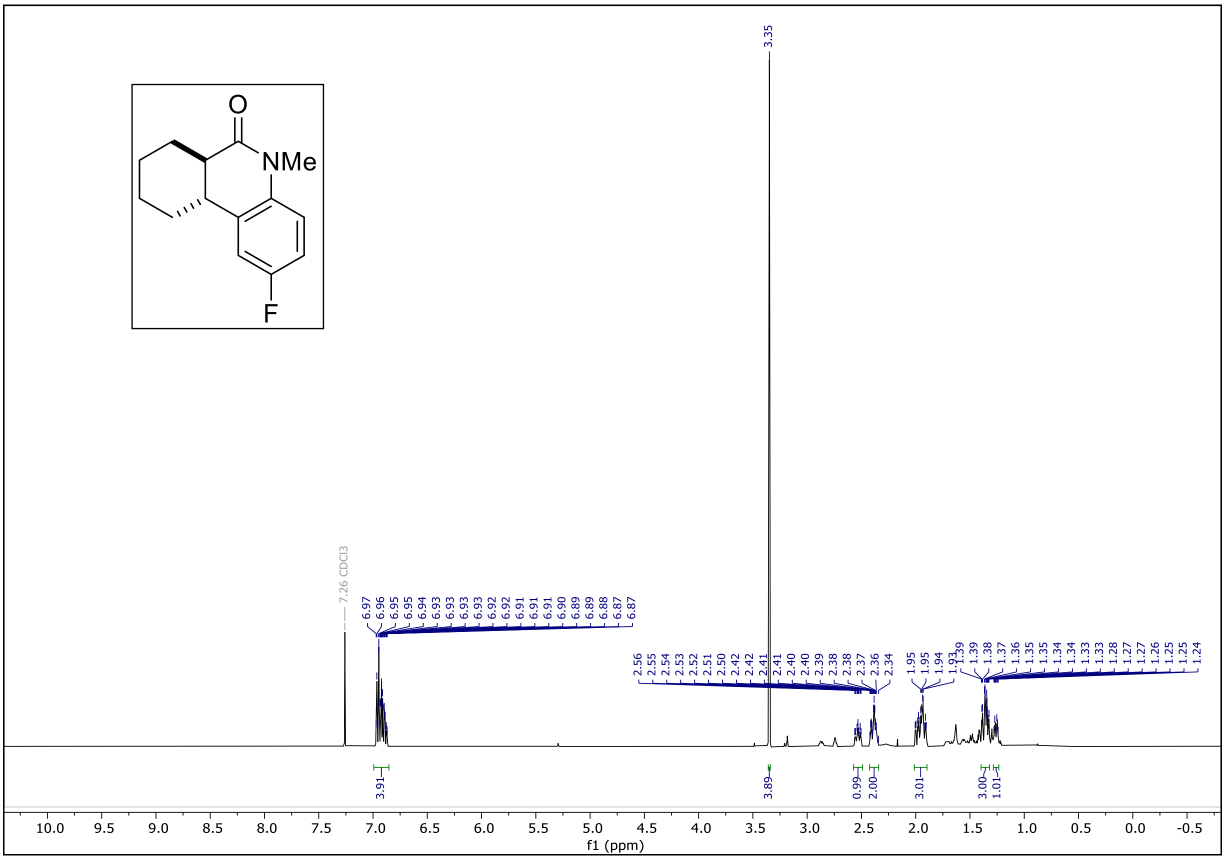

^{13}C NMR {^{19}F} (126 MHz, $CDCl_3$): *trans*-**2s**

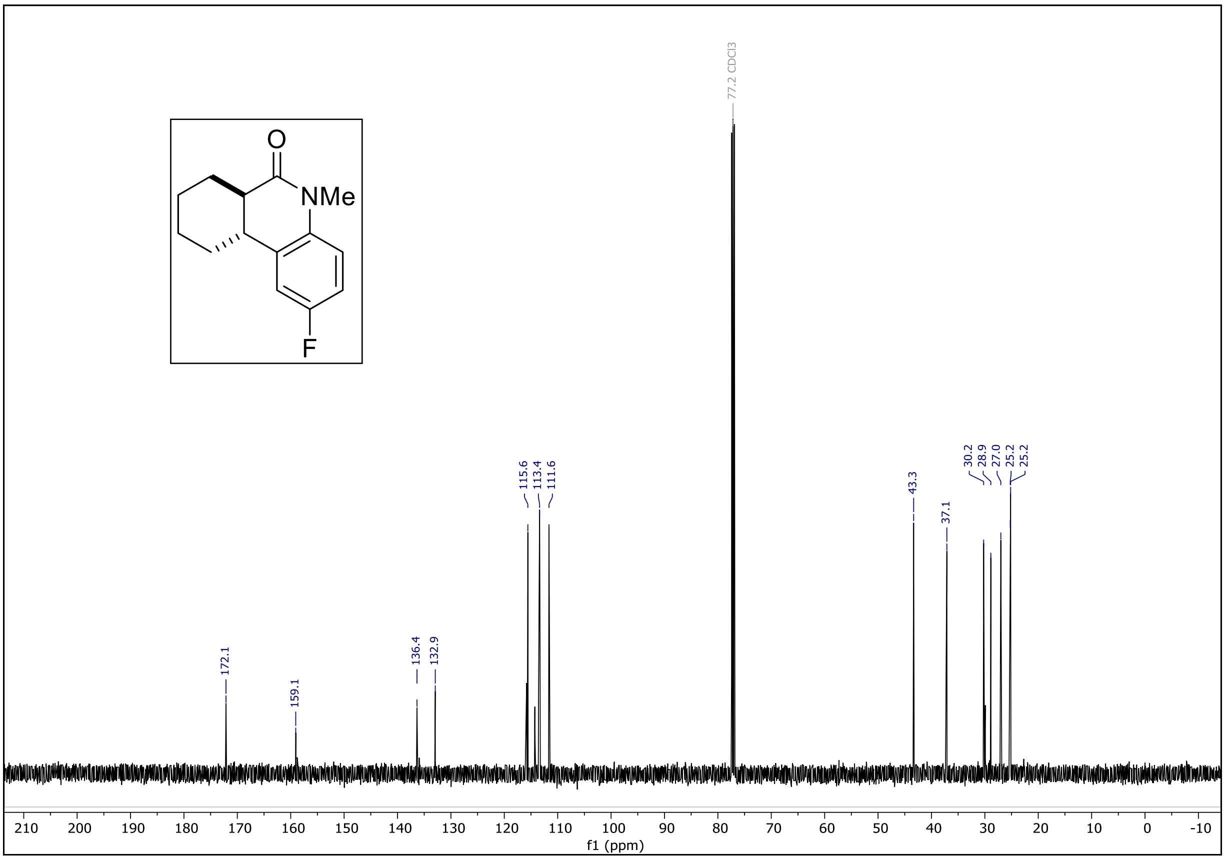

^{19}F NMR {^{1}H} (470 MHz, $CDCl_3$): *trans*-**2s**

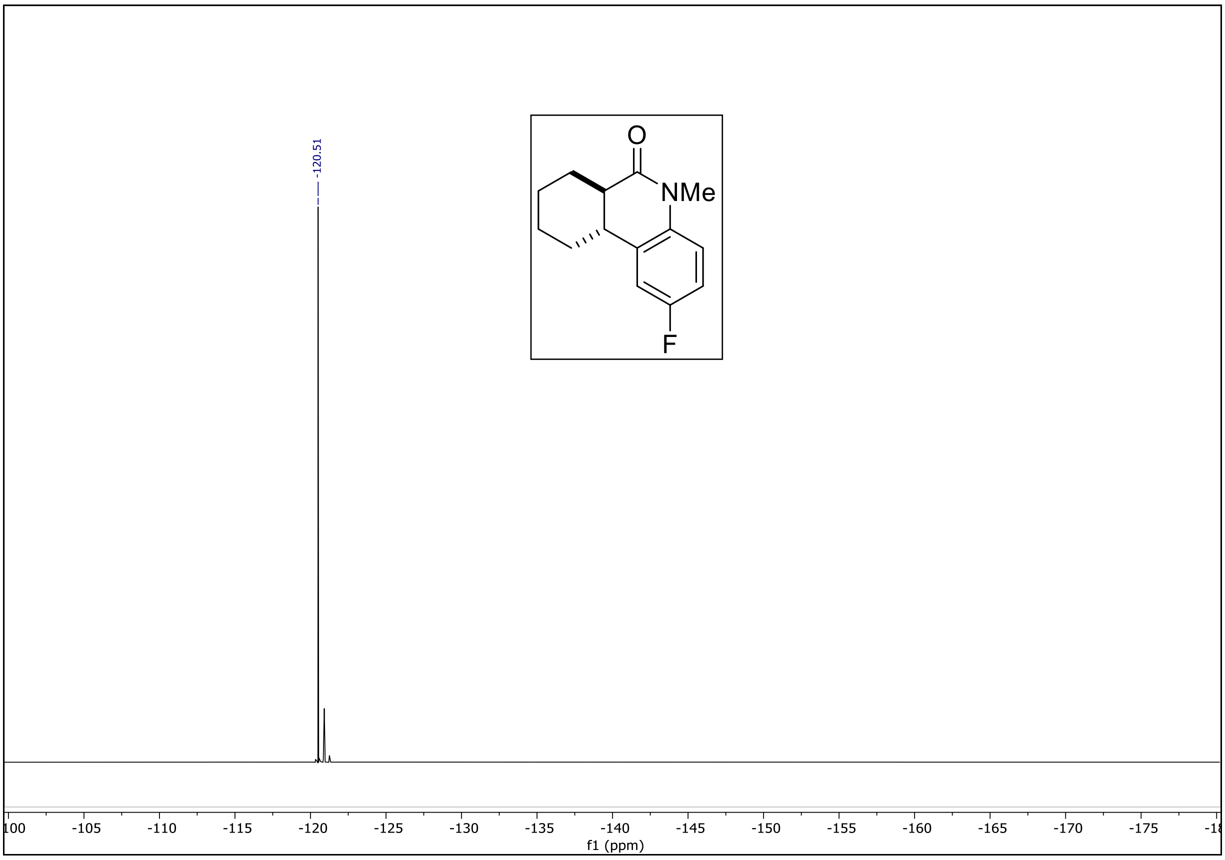

^{1}H NMR (600 MHz, $CDCl_3$): *trans*-**2t**

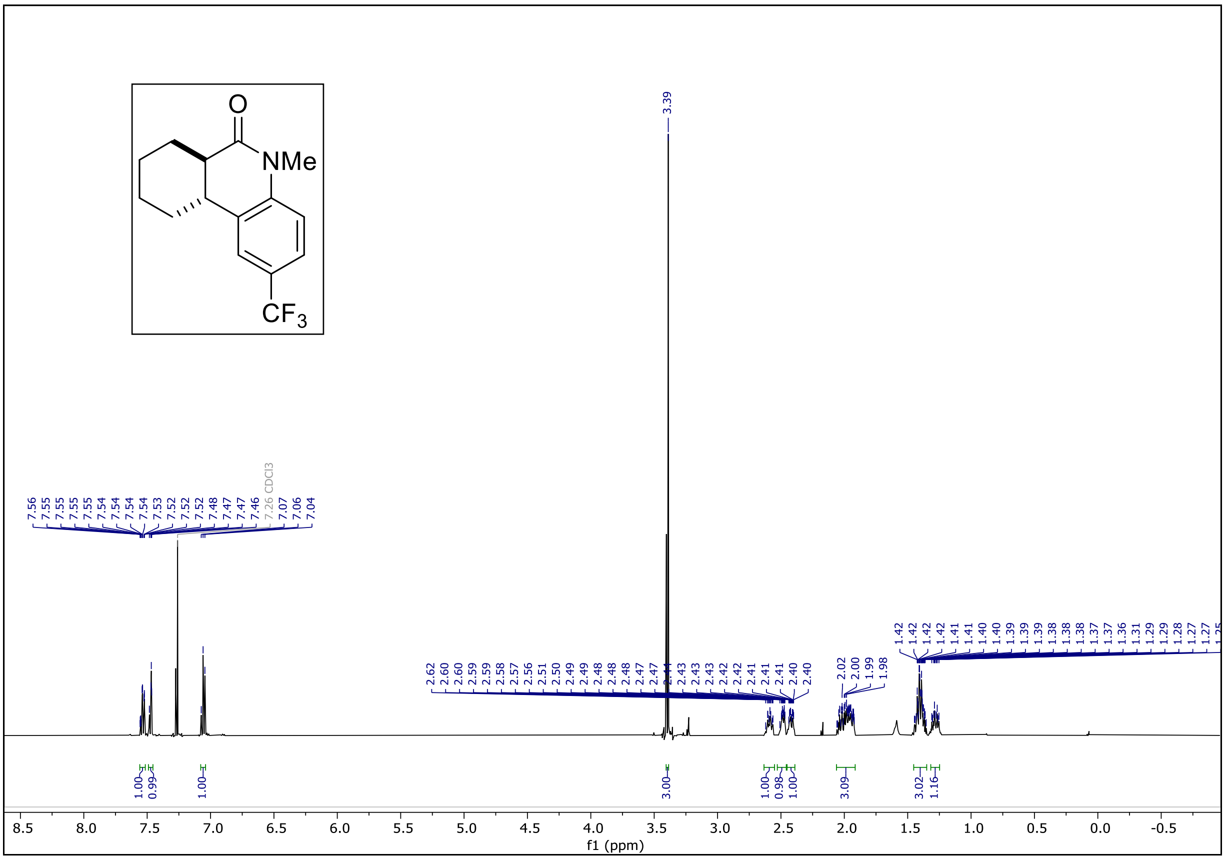

^{13}C NMR (151 MHz, $CDCl_3$): *trans*-**2t**

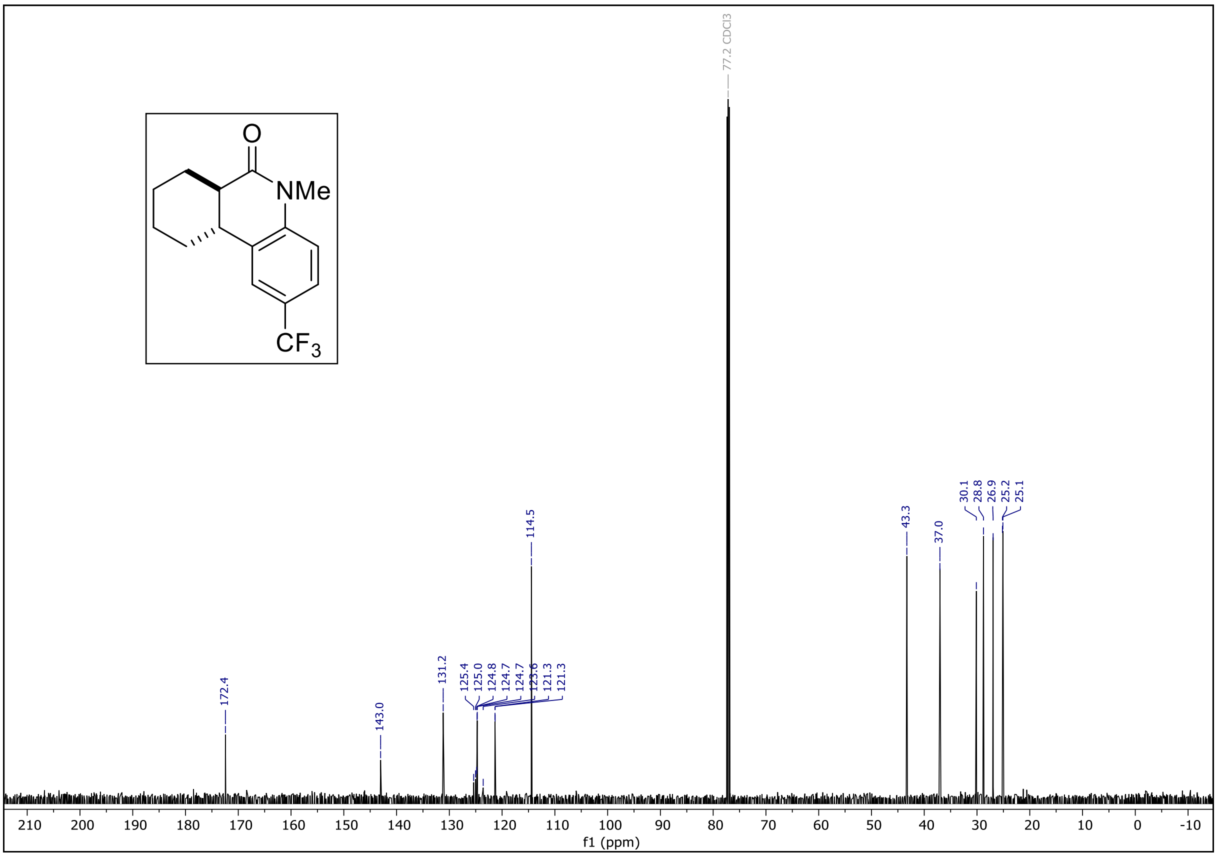

^{19}F NMR {^1H} (564 MHz, $CDCl_3$): *trans*-**2t**

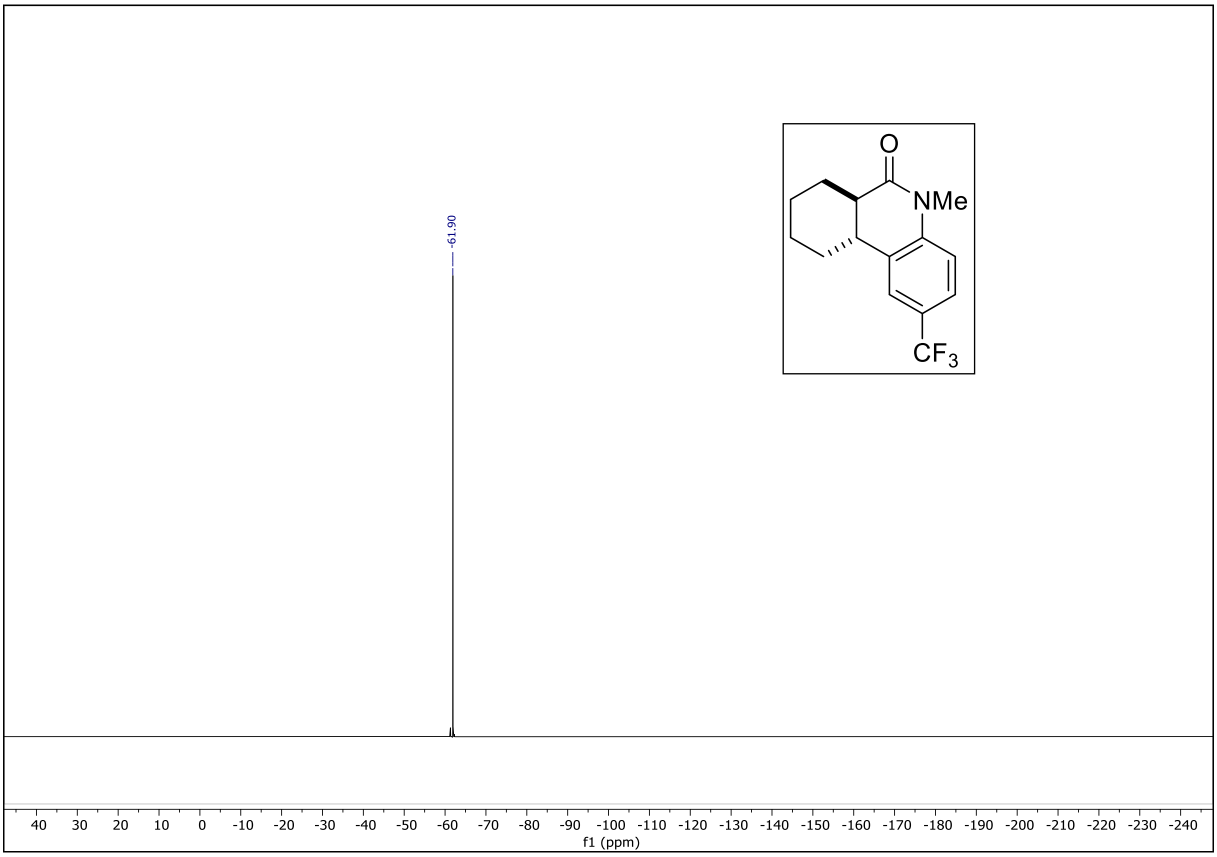

^1H NMR (600 MHz, $CDCl_3$): *trans*-**2u**

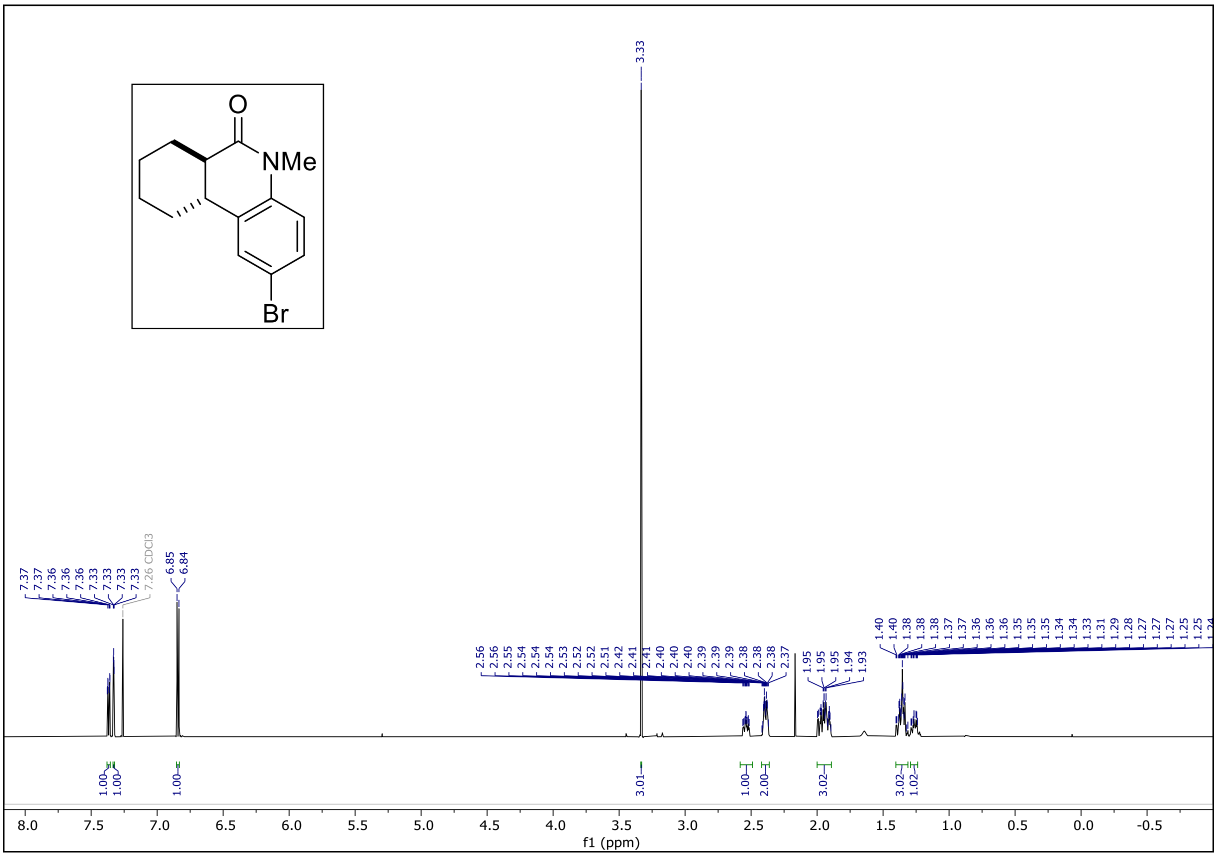

^{13}C NMR (151 MHz, $CDCl_3$): *trans*-**2u**

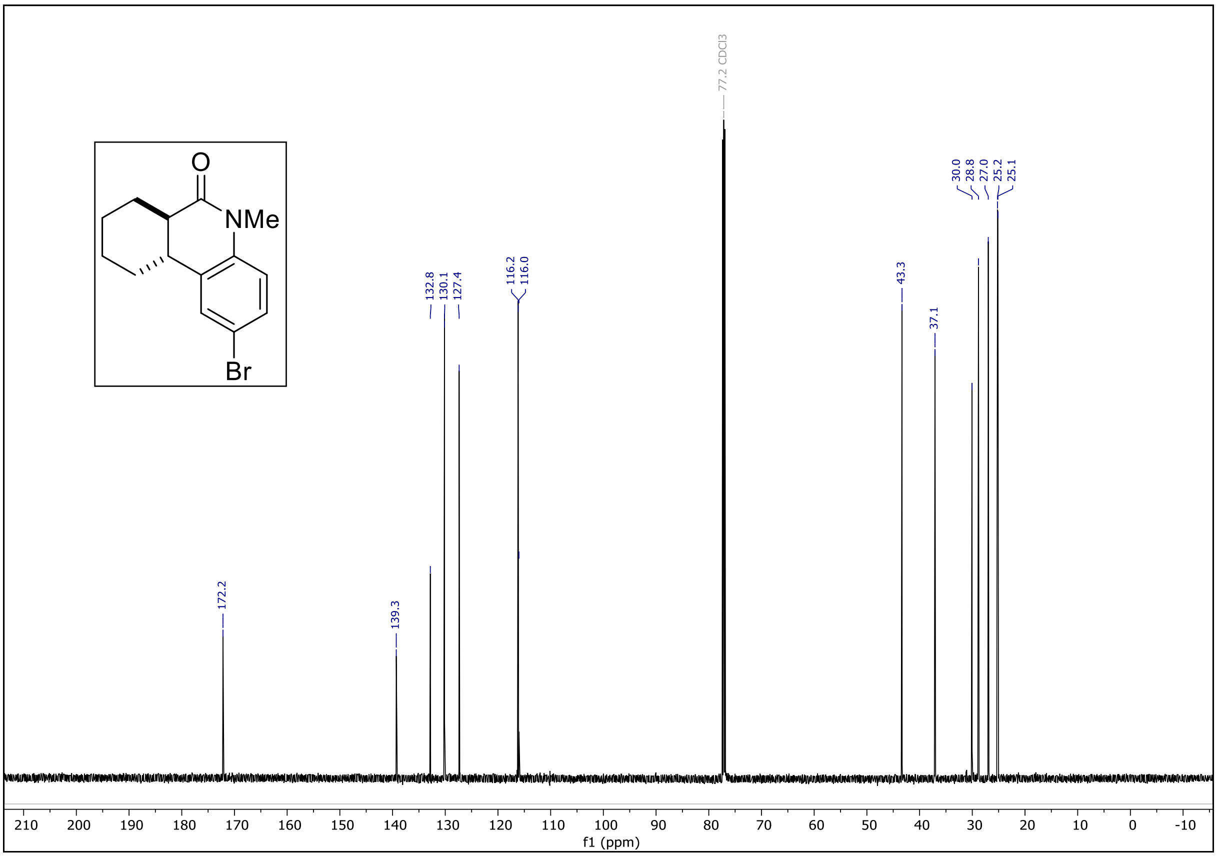

^{1}H NMR (500 MHz, $CDCl_3$): *trans*-**S1**

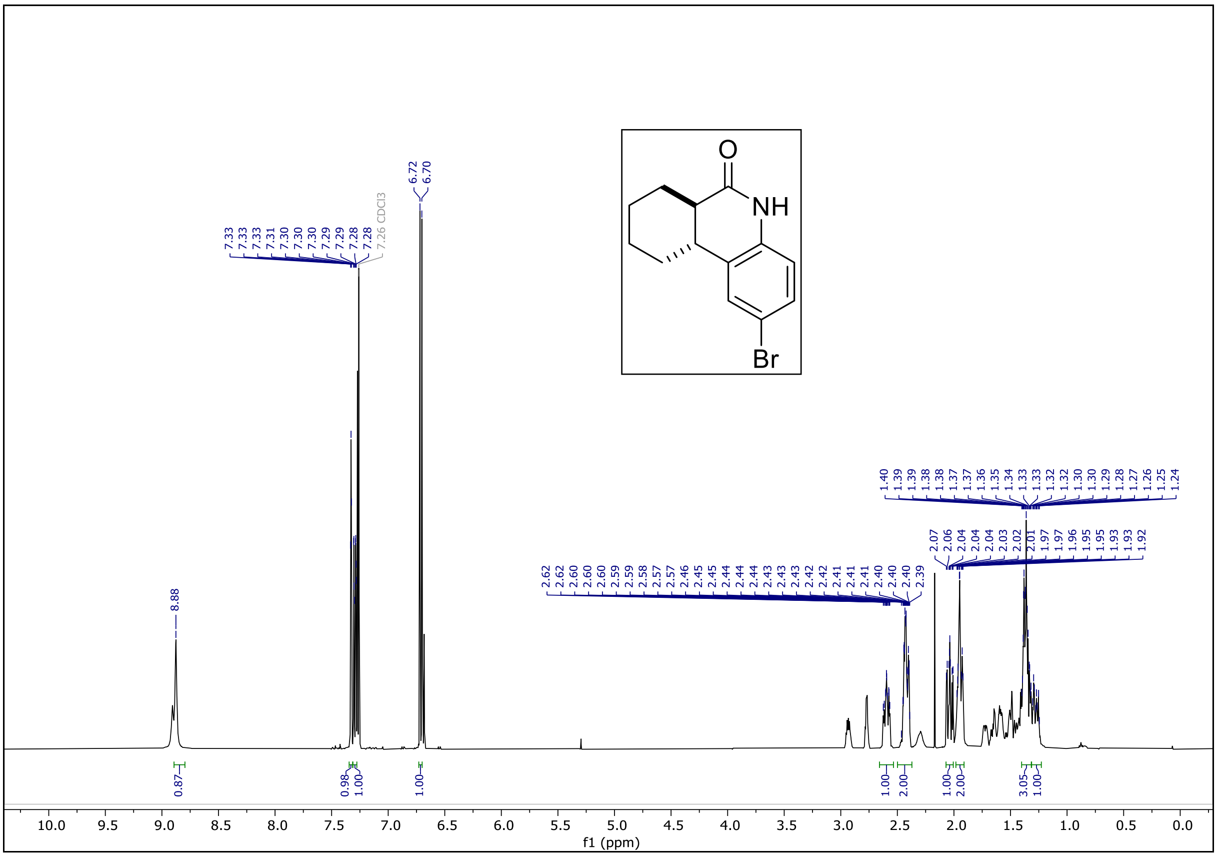

^{13}C NMR (126 MHz, $CDCl_3$): *trans*-**S1**

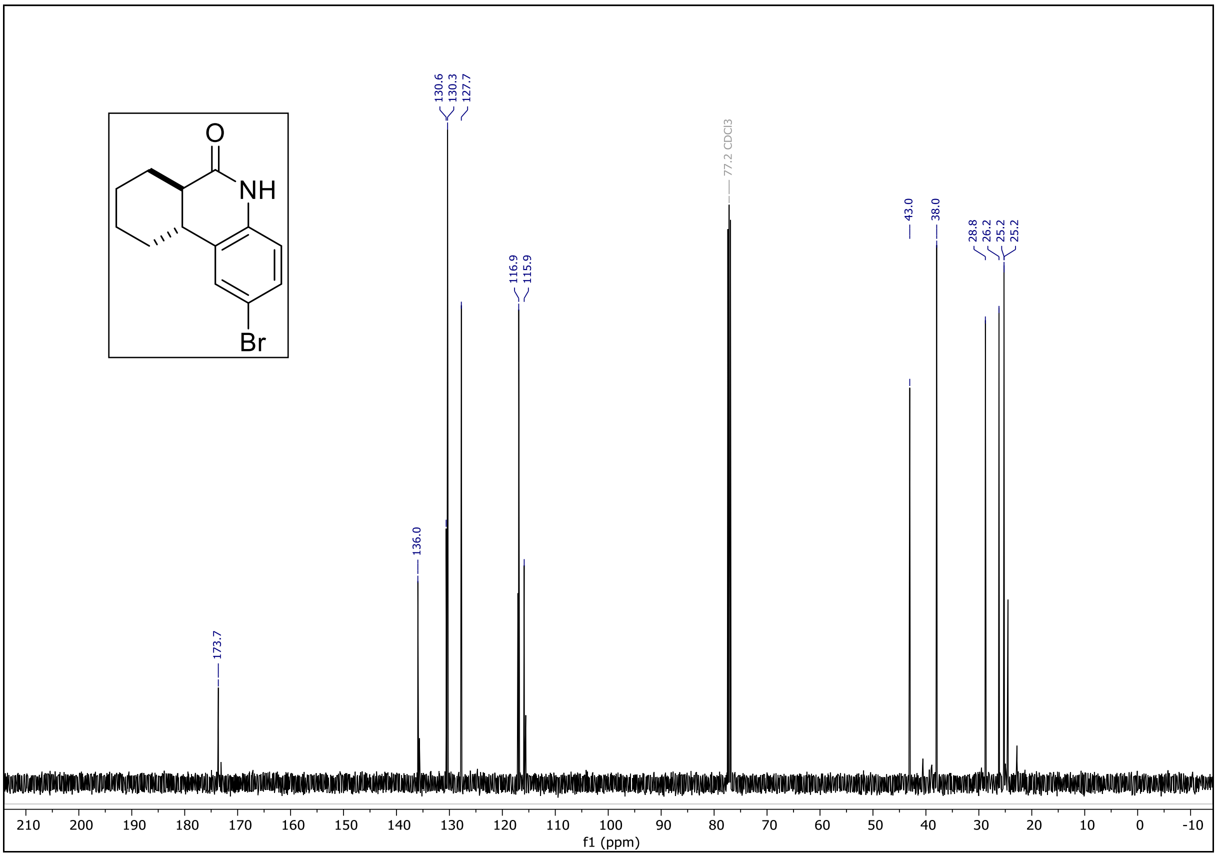

^{1}H NMR (500 MHz, $CDCl_3$): *cis*-**S1**

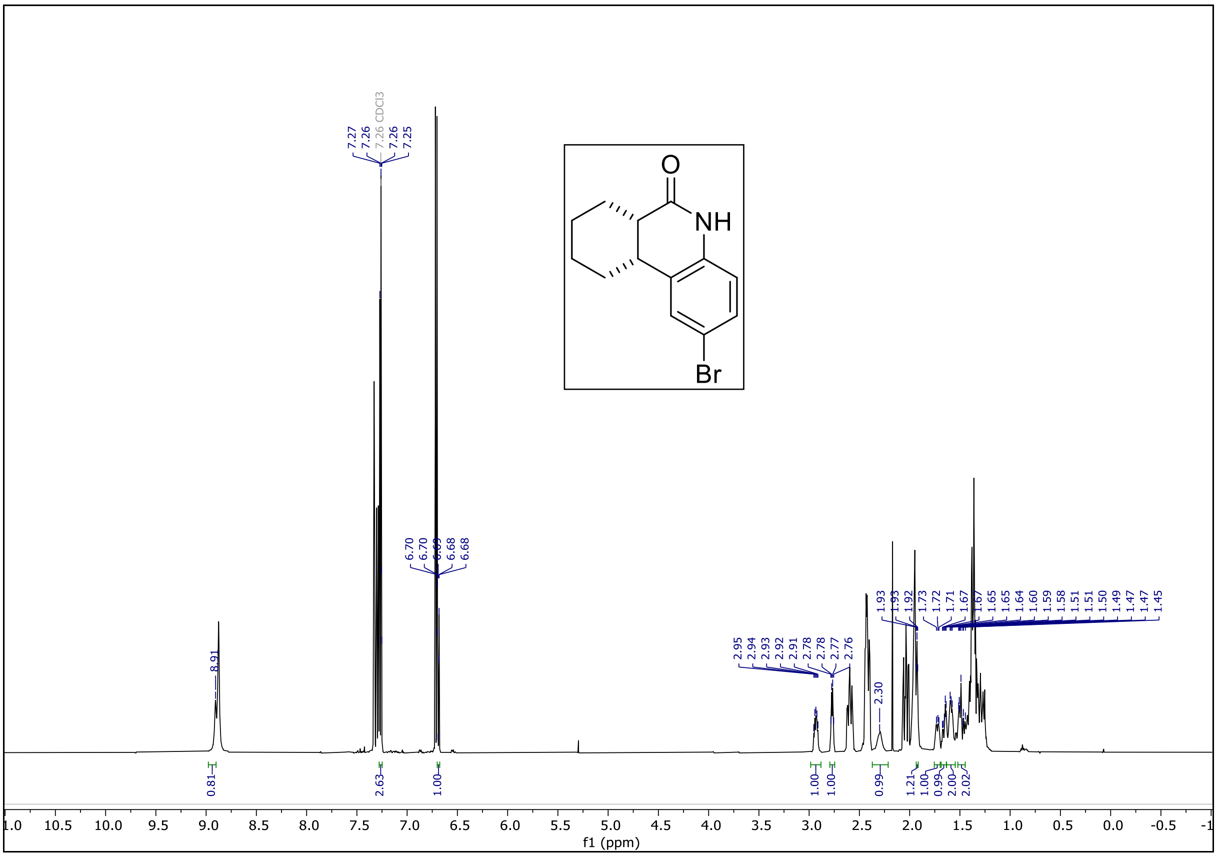

^{13}C NMR (126 MHz, $CDCl_3$): *cis*-**S1**

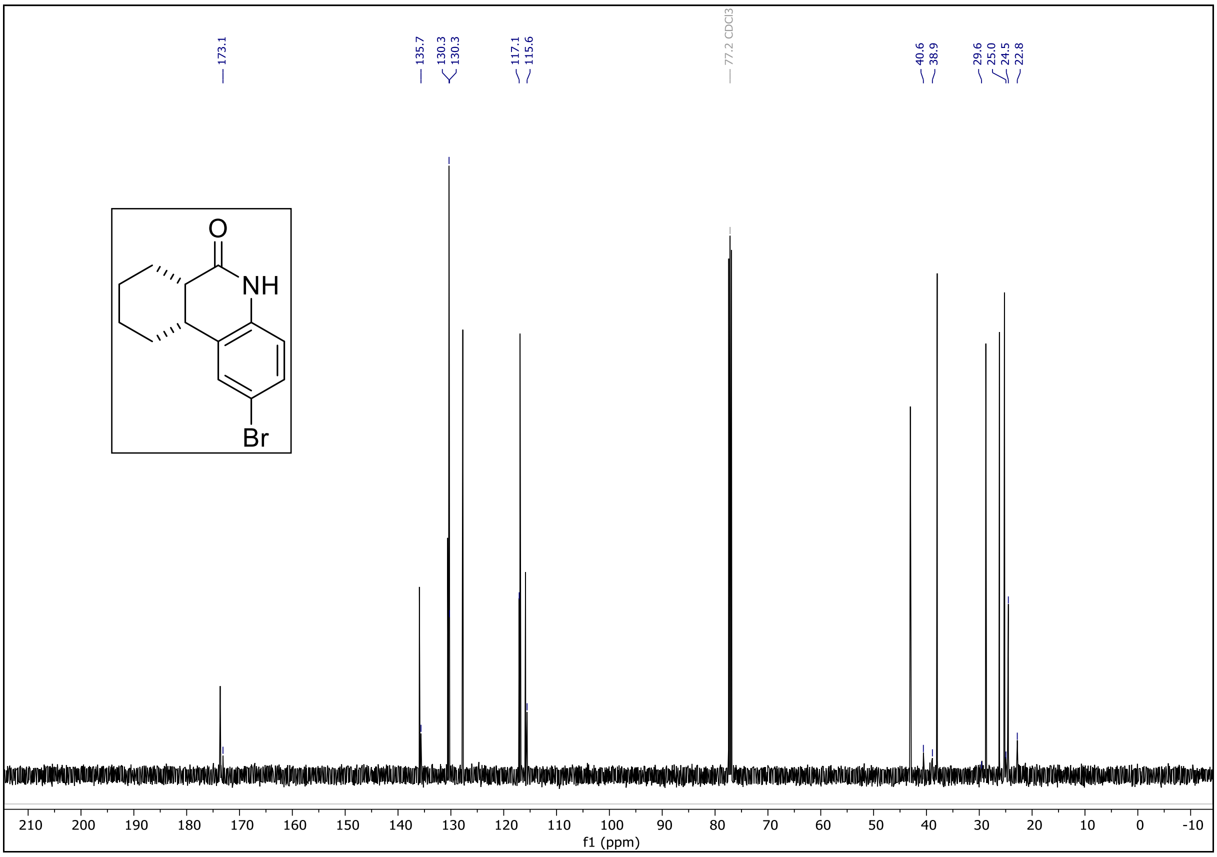

^{1}H NMR (500 MHz, $CDCl_3$): *trans*-**S2**

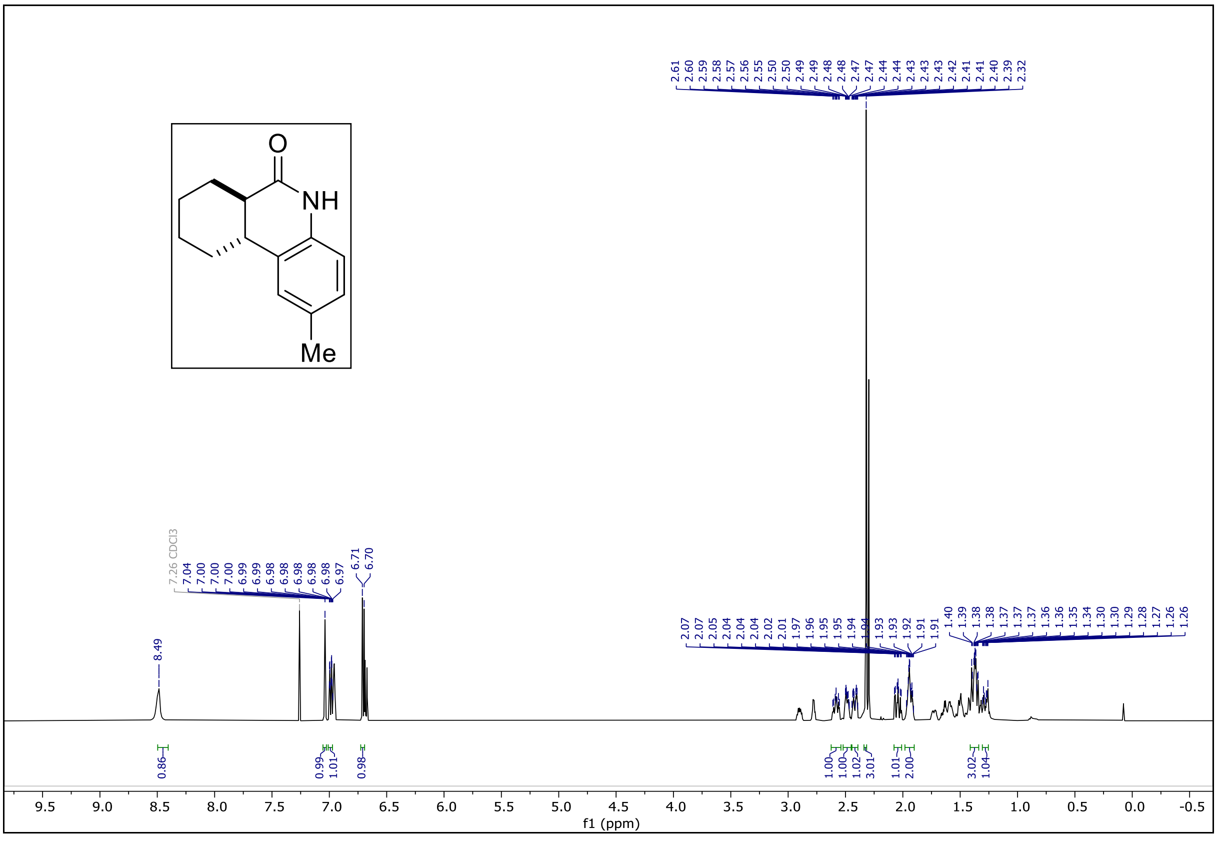

^{13}C NMR (126 MHz, $CDCl_3$): *trans*-**S2**

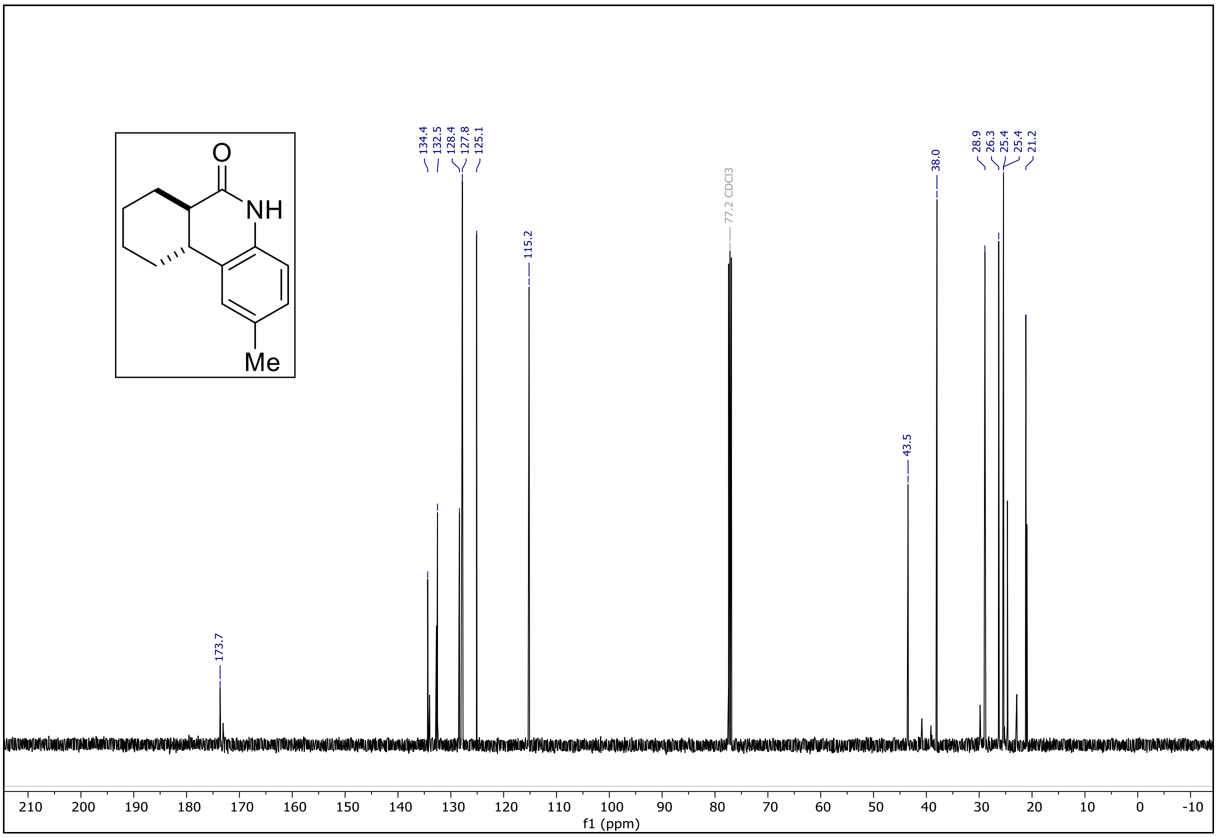

^{1}H NMR (500 MHz, $CDCl_3$): *cis*-**S2**

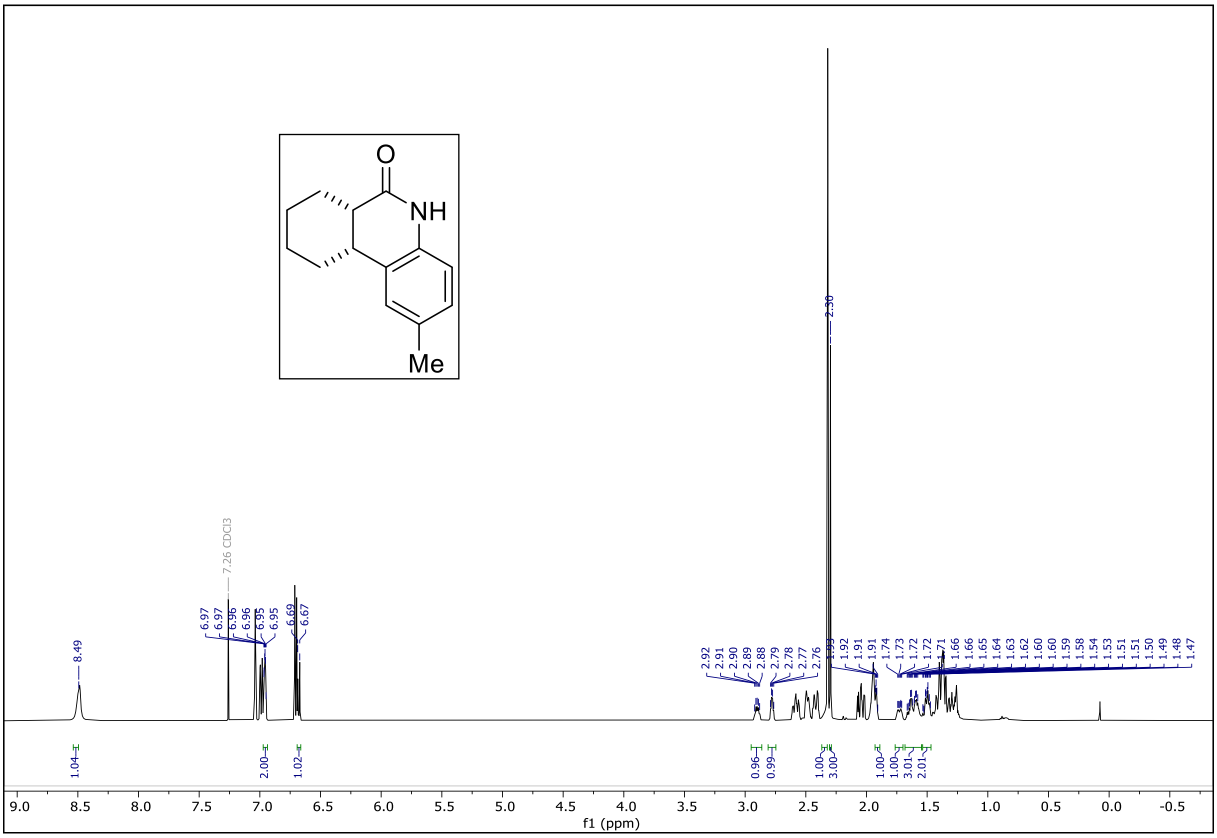

^{13}C NMR (126 MHz, $CDCl_3$): *cis*-**S2**

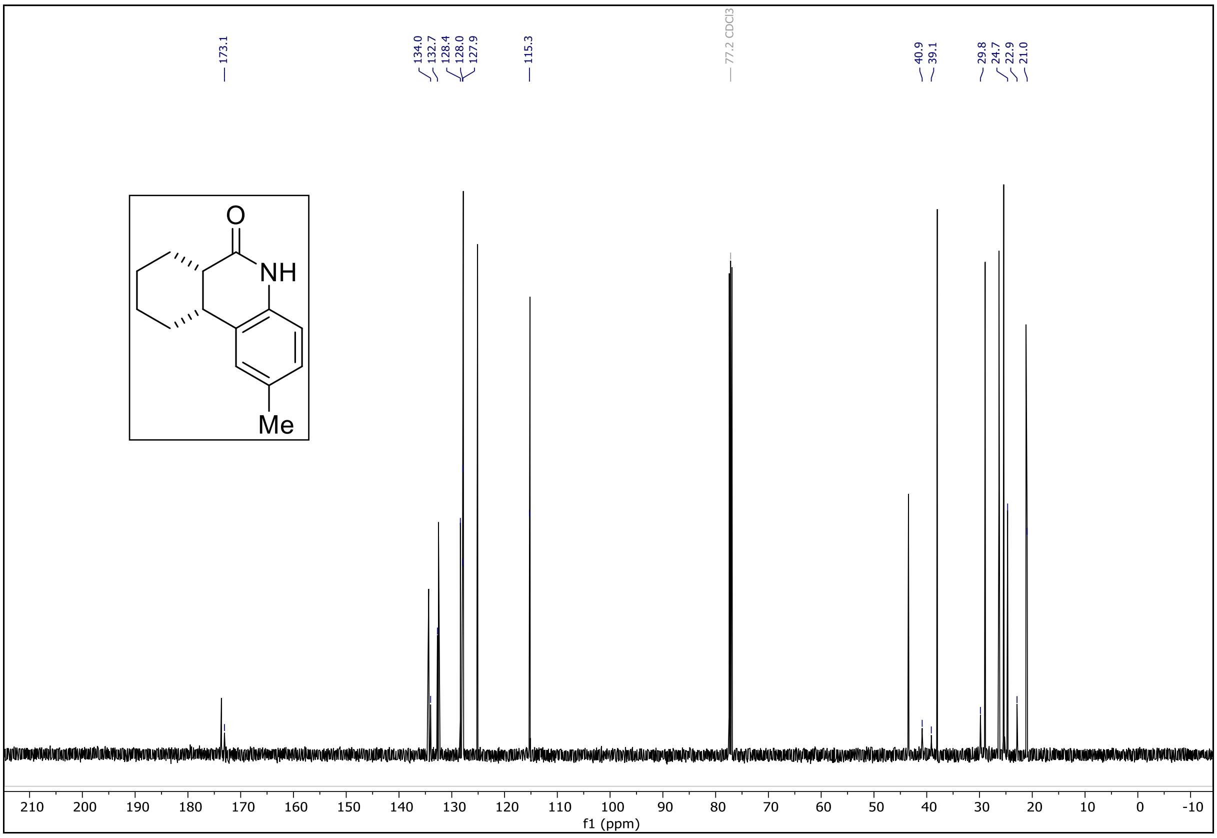

^{1}H NMR (600 MHz, $CDCl_3$): *trans*-**S3**

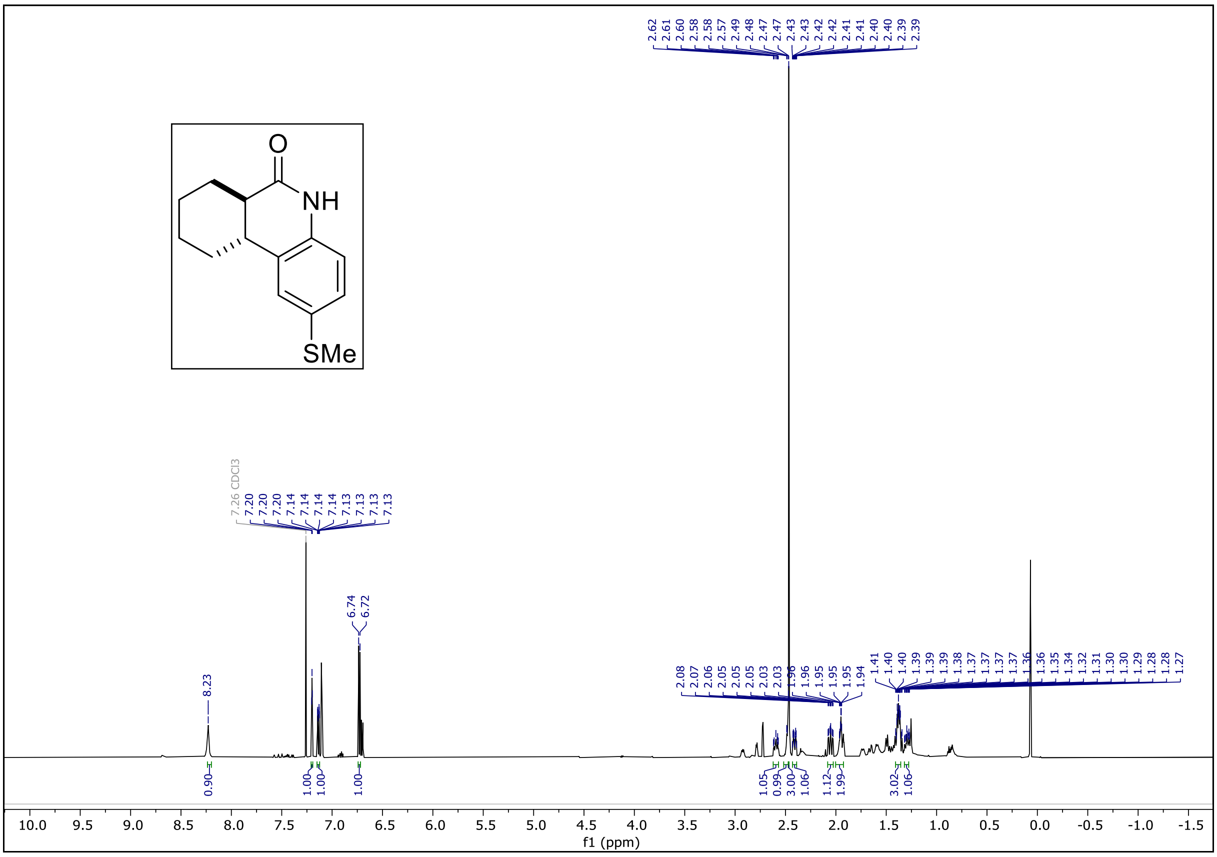

^{13}C NMR (151 MHz, CDCl$_3$): *trans*-**S3**

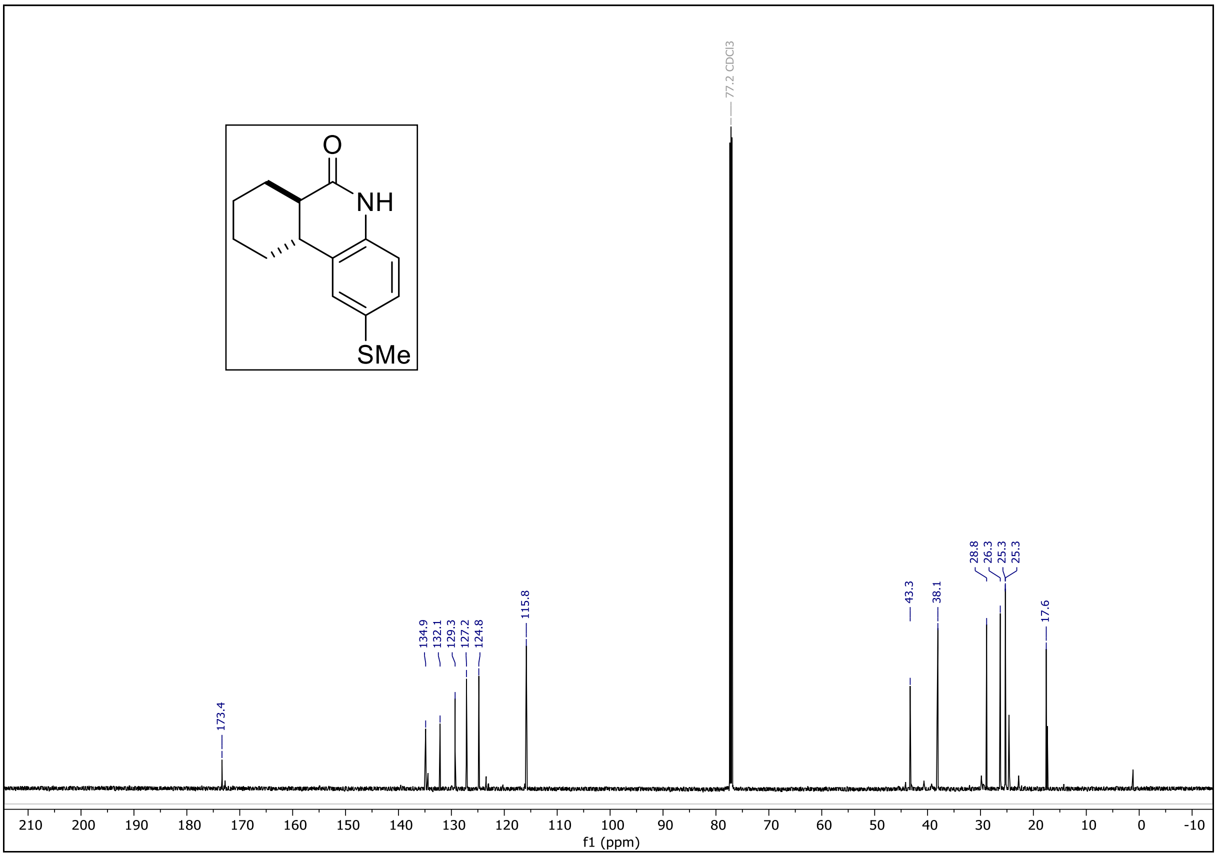

^{1}H NMR (600 MHz, CDCl$_3$): *cis*-**S3**

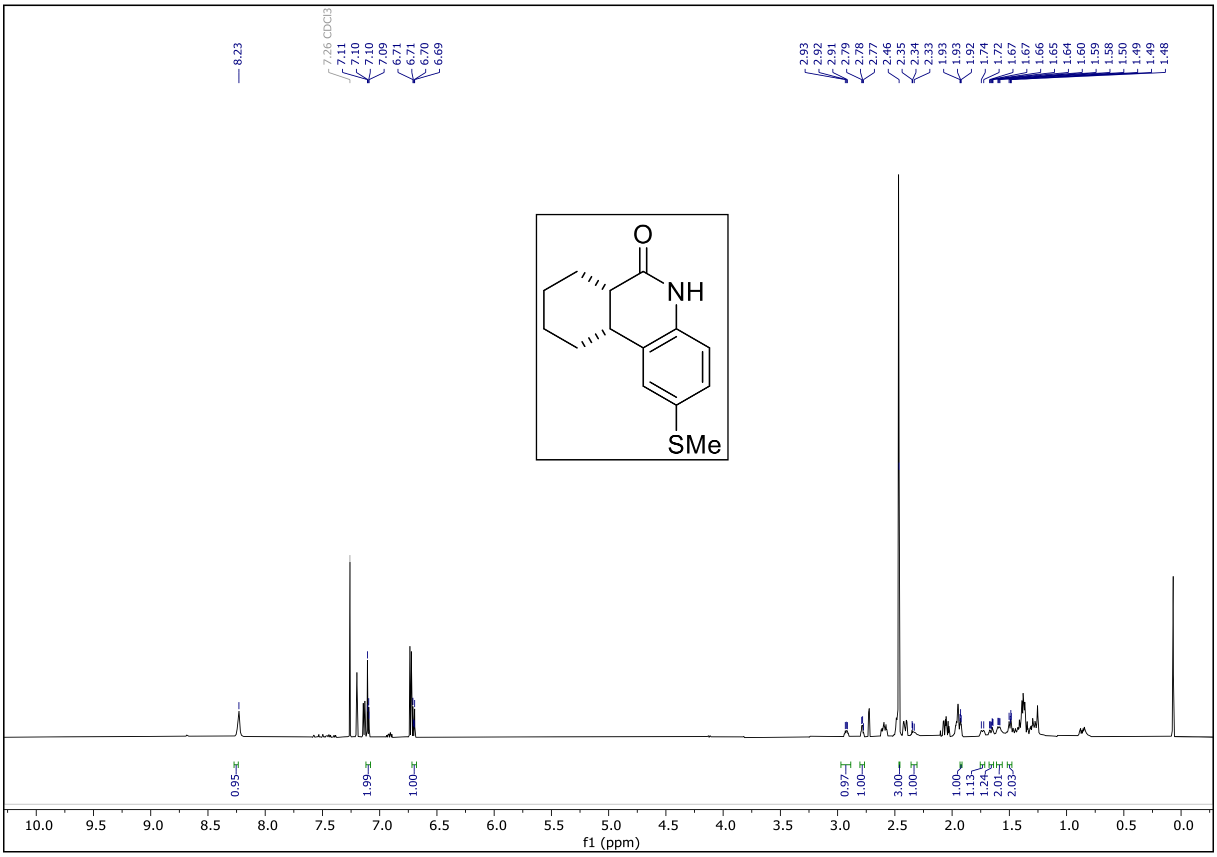

^{13}C NMR (151 MHz, $CDCl_3$): *cis*-**S3**

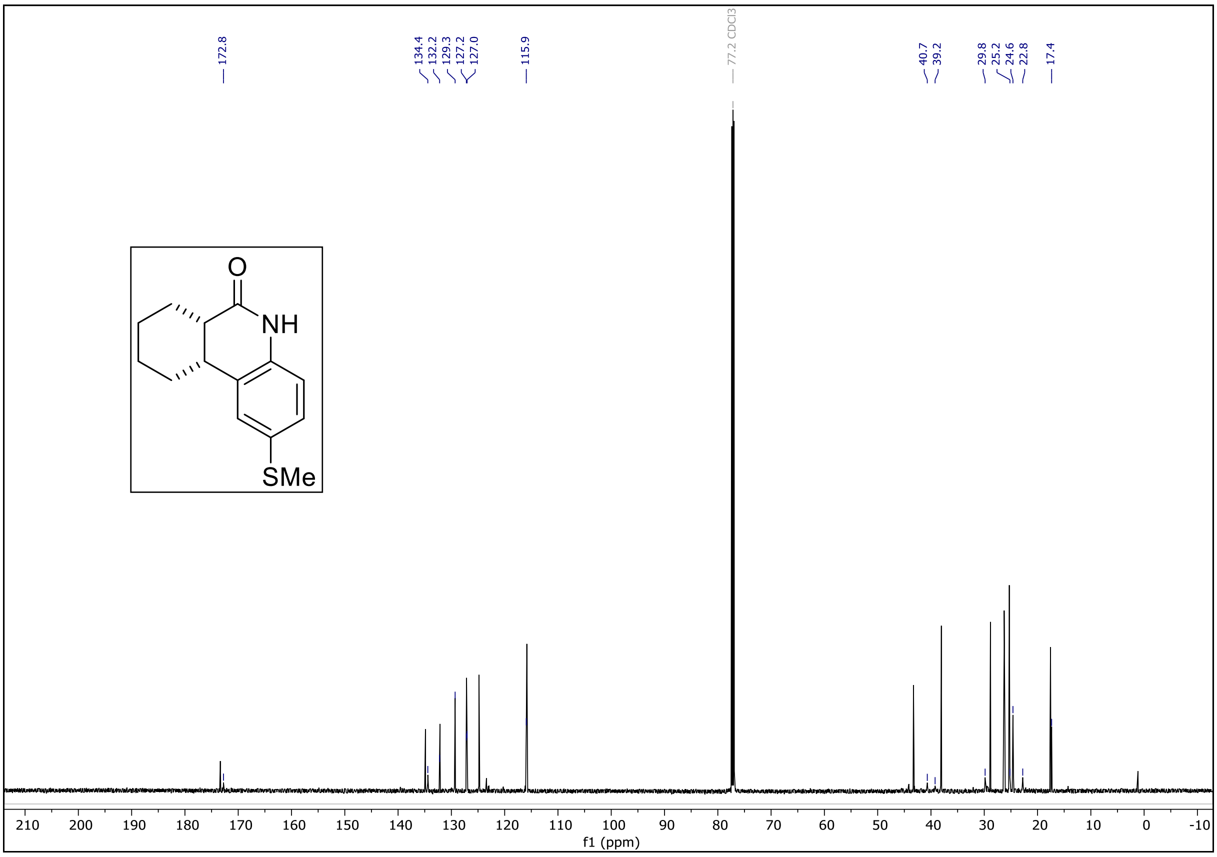

^{1}H NMR (600 MHz, $CDCl_3$): *trans*-**S4**

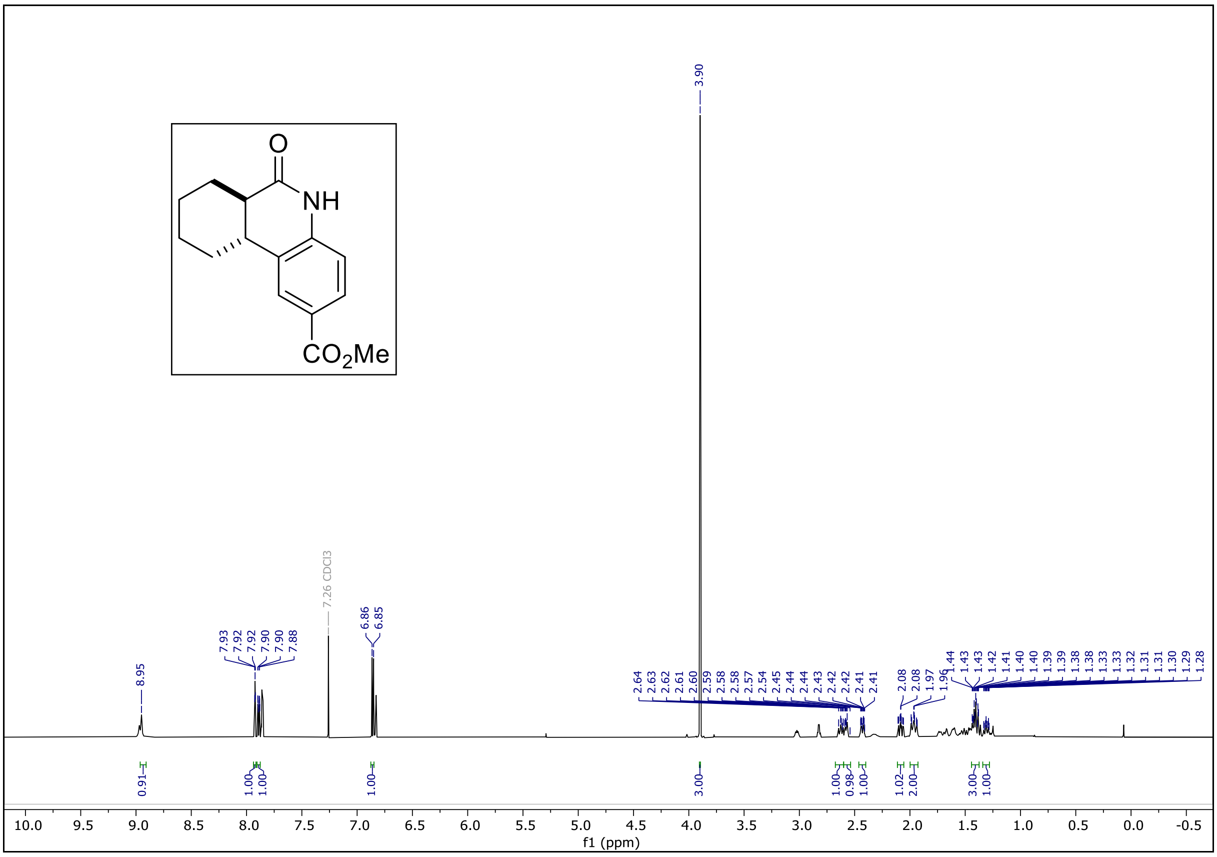

^{13}C NMR (151 MHz, $CDCl_3$): *trans*-**S4**

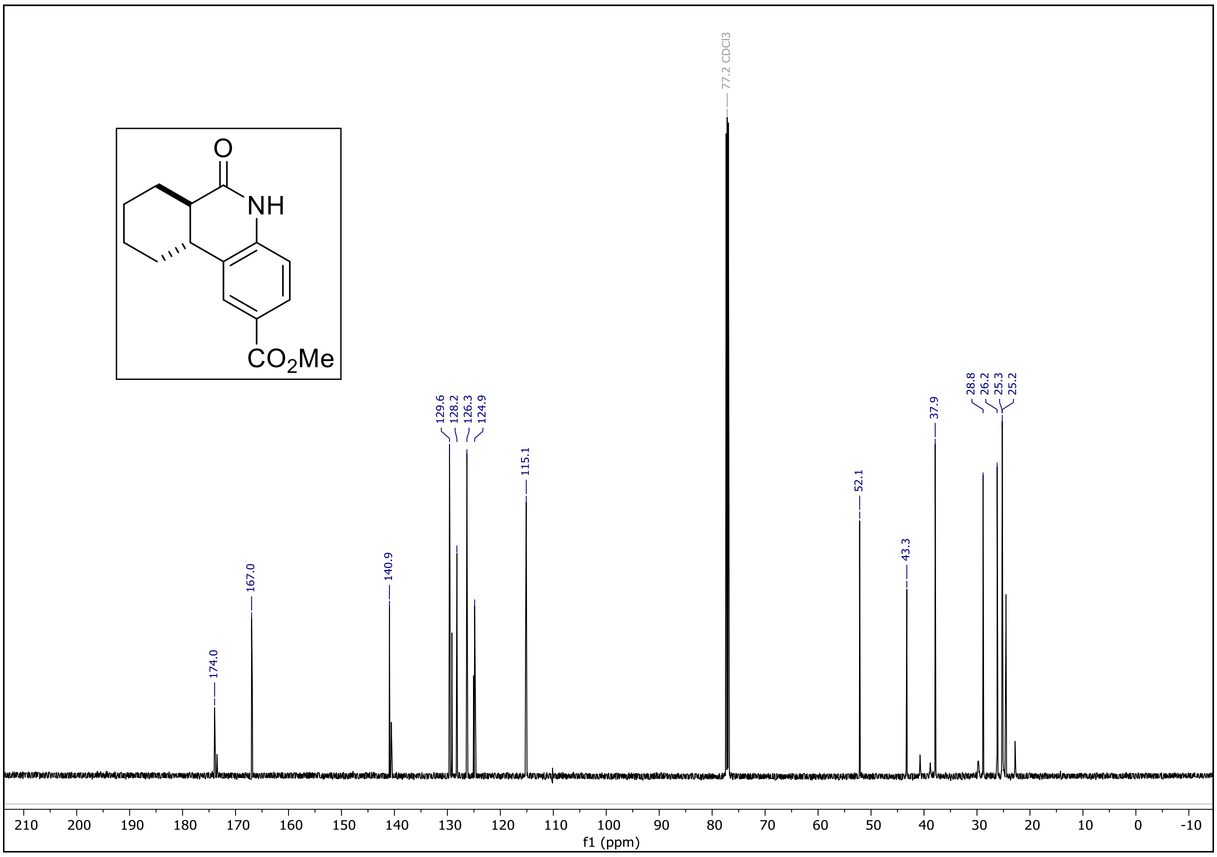

^{1}H NMR (600 MHz, $CDCl_3$): *cis*-**S4**

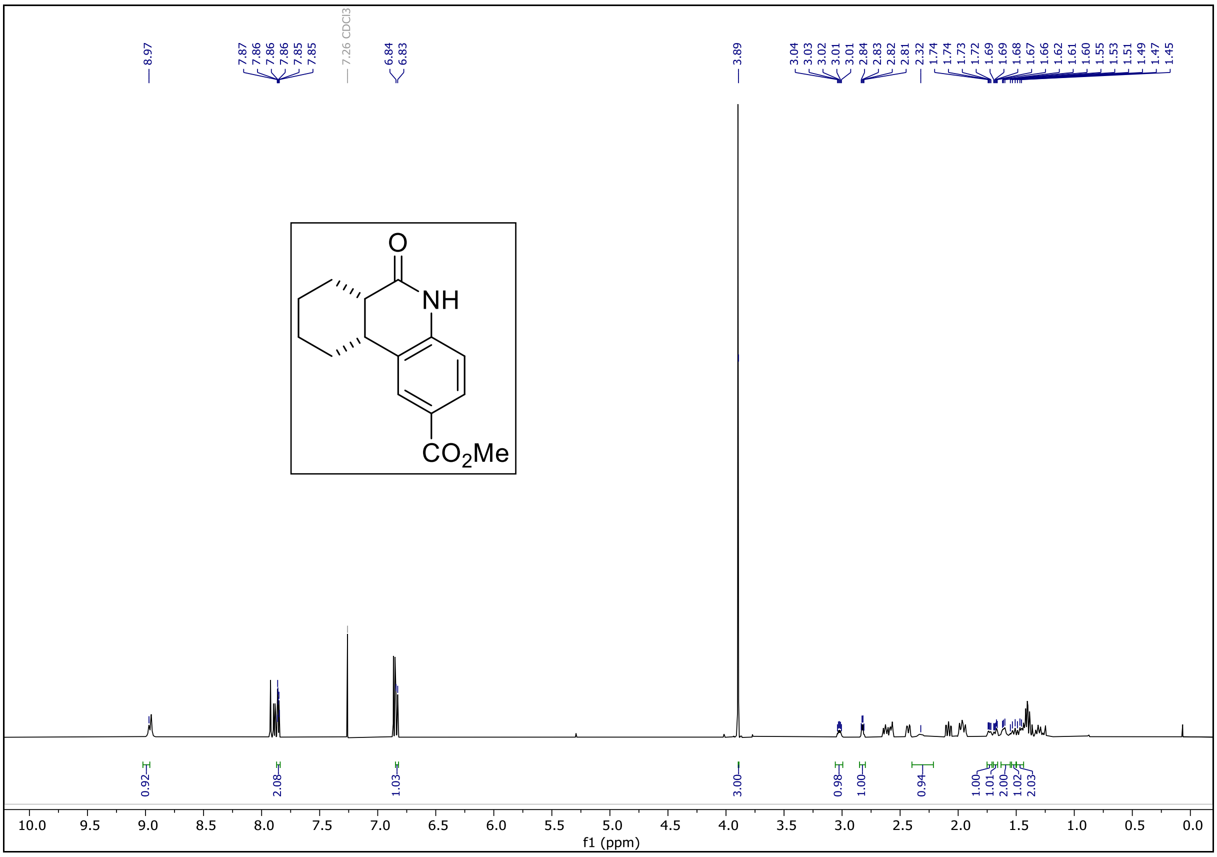

^{13}C NMR (151 MHz, $CDCl_3$): *cis*-**S4**

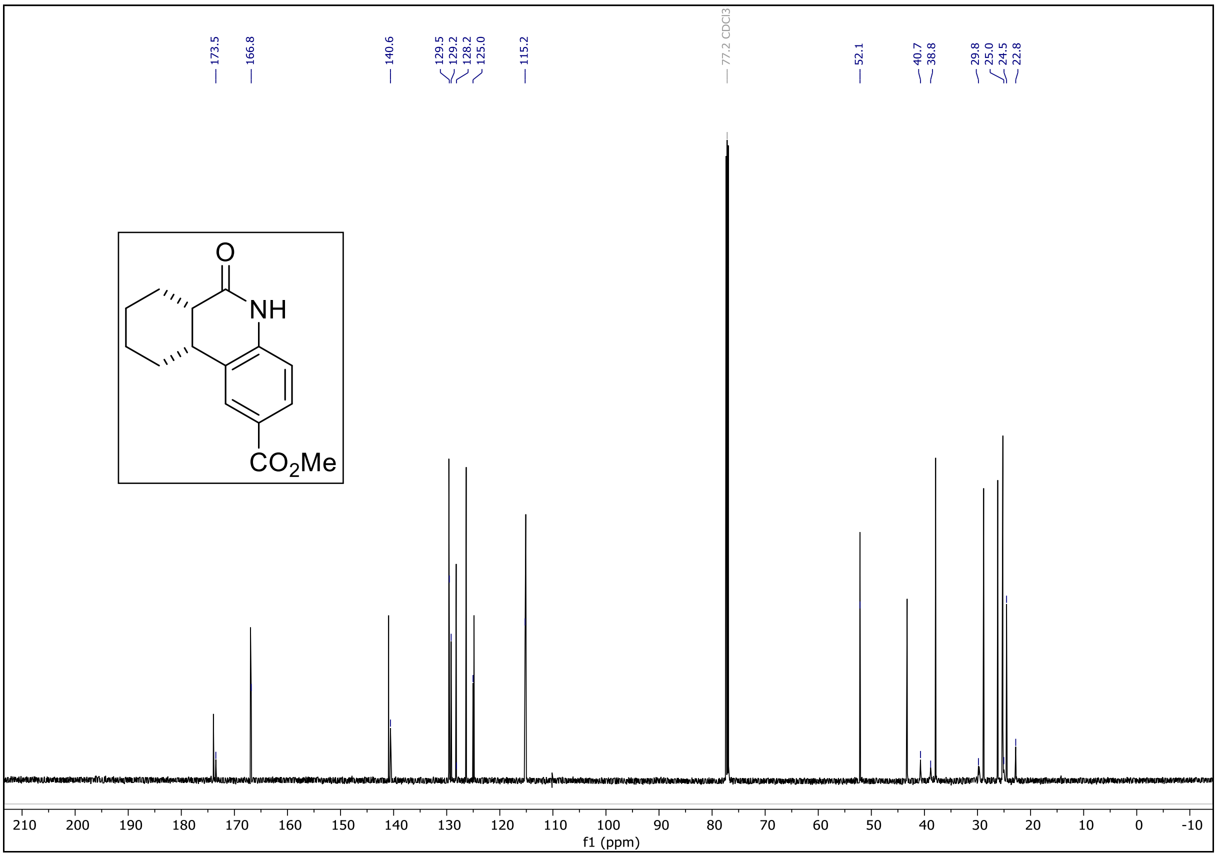

^{1}H NMR (500 MHz, $CDCl_3$): *trans*-**S5**

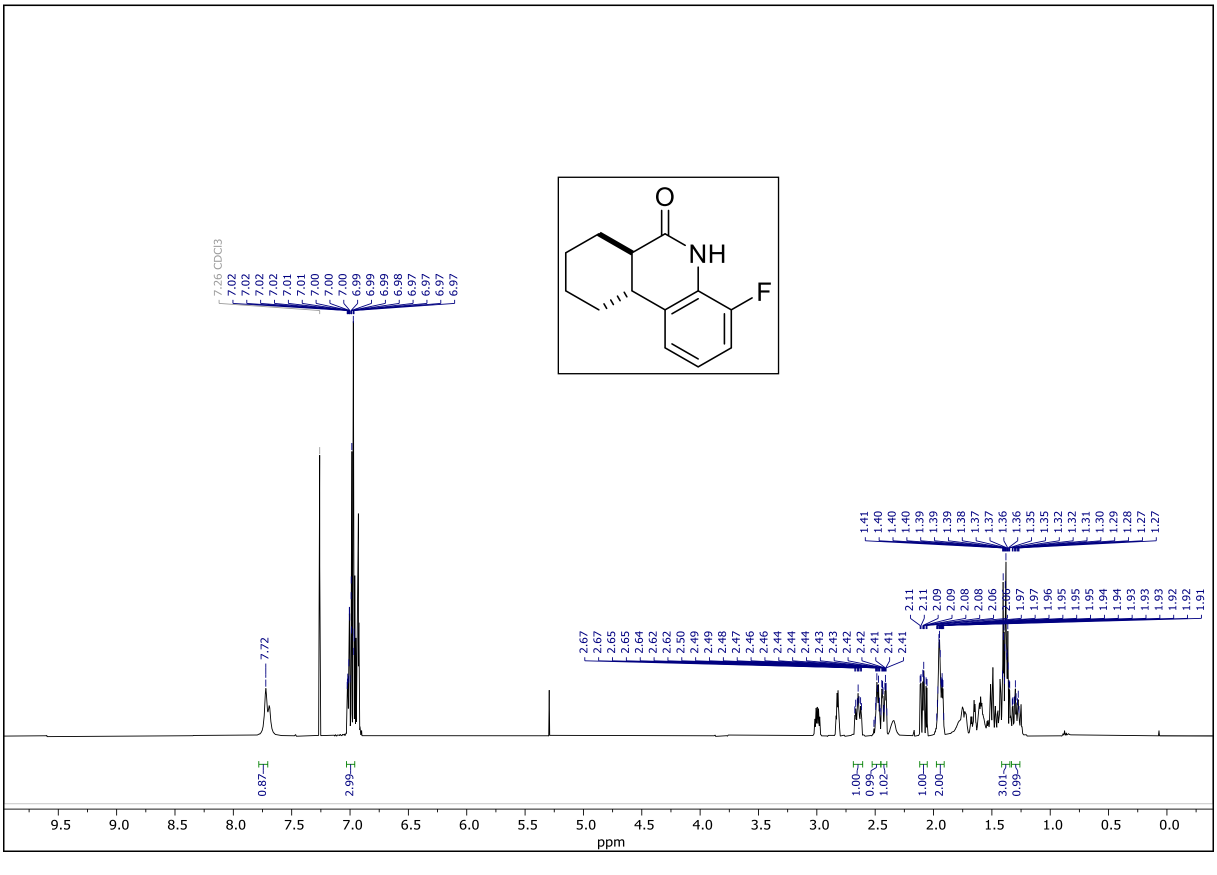

^{13}C NMR (126 MHz, $CDCl_3$): *trans*-**S5**

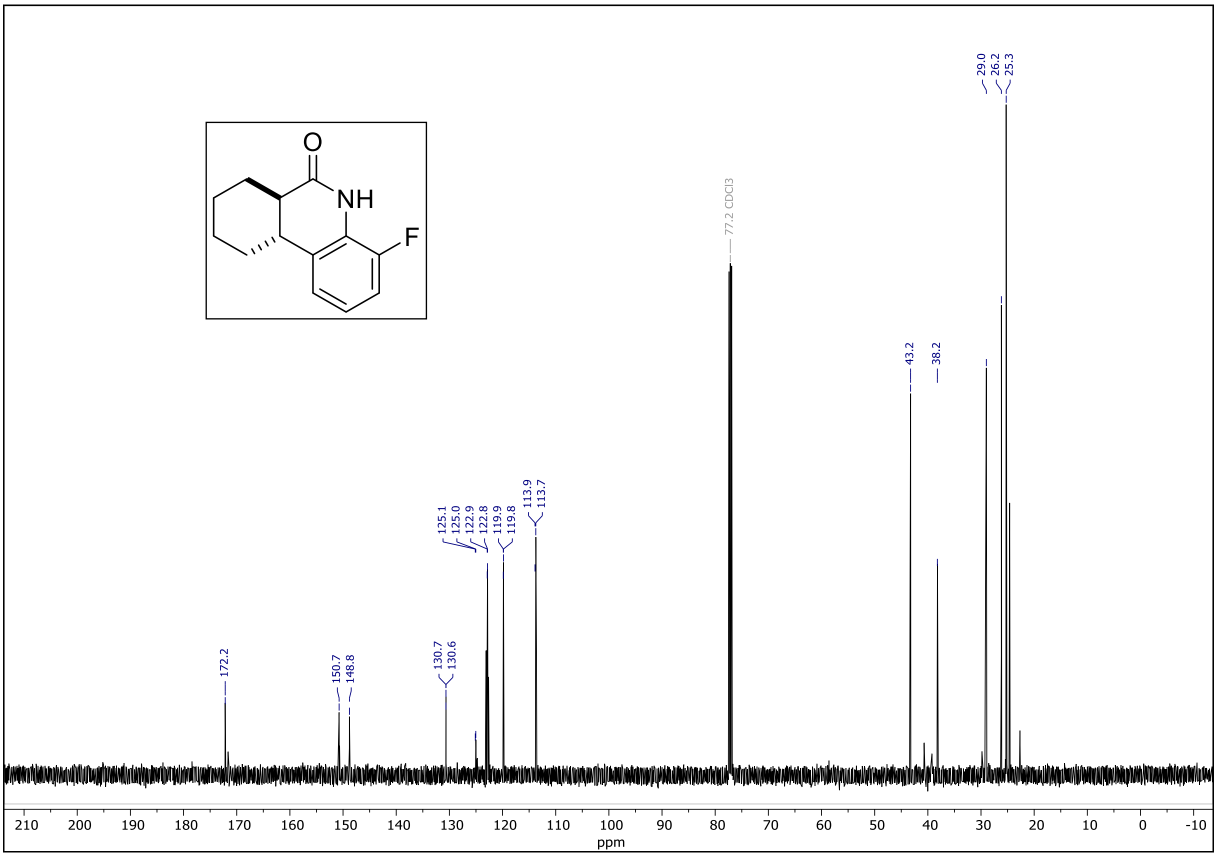

^{19}F NMR {^{1}H} (376 MHz, $CDCl_3$): *trans*-**S5**

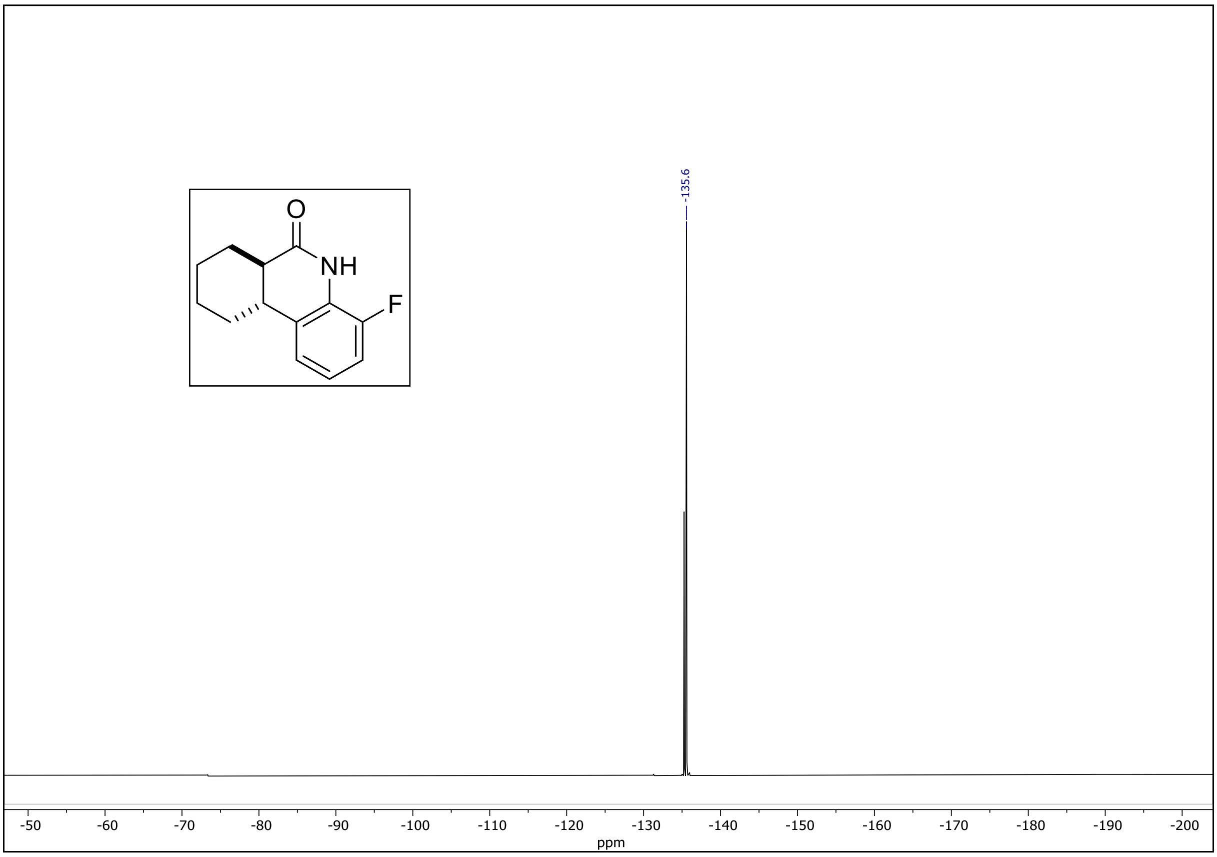

^{1}H NMR (500 MHz, $CDCl_3$): *cis*-**S5**

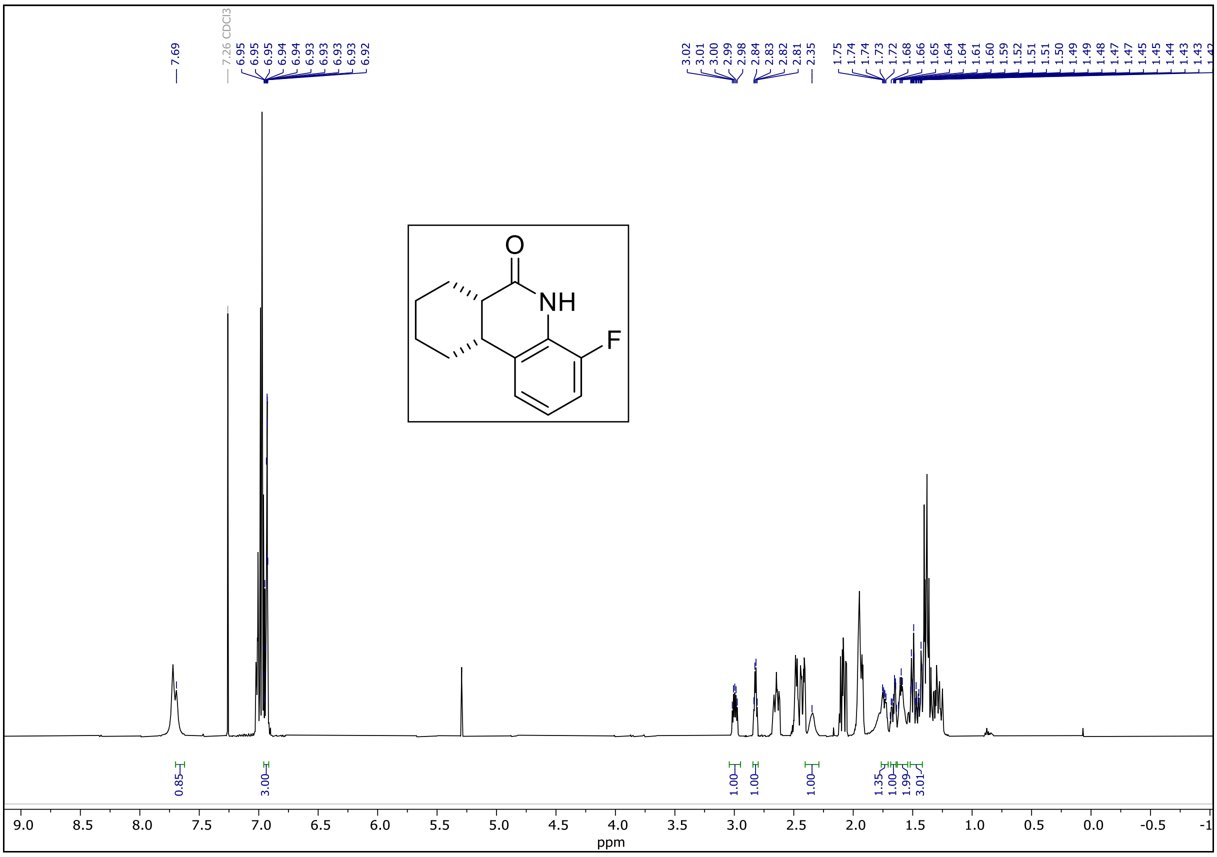

^{13}C NMR (126 MHz, $CDCl_3$): *cis*-**S5**

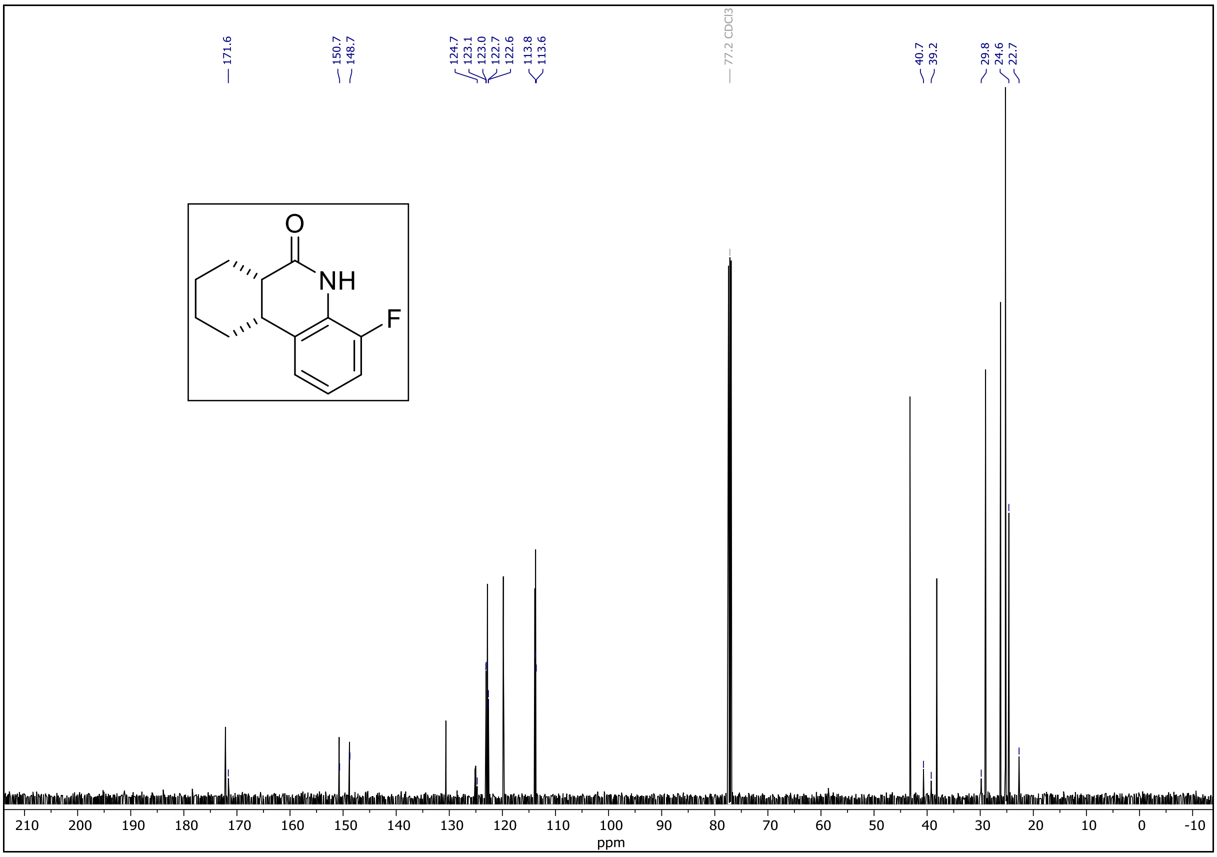

^{19}F NMR {^{1}H} (376 MHz, $CDCl_3$): *cis*-**S5**

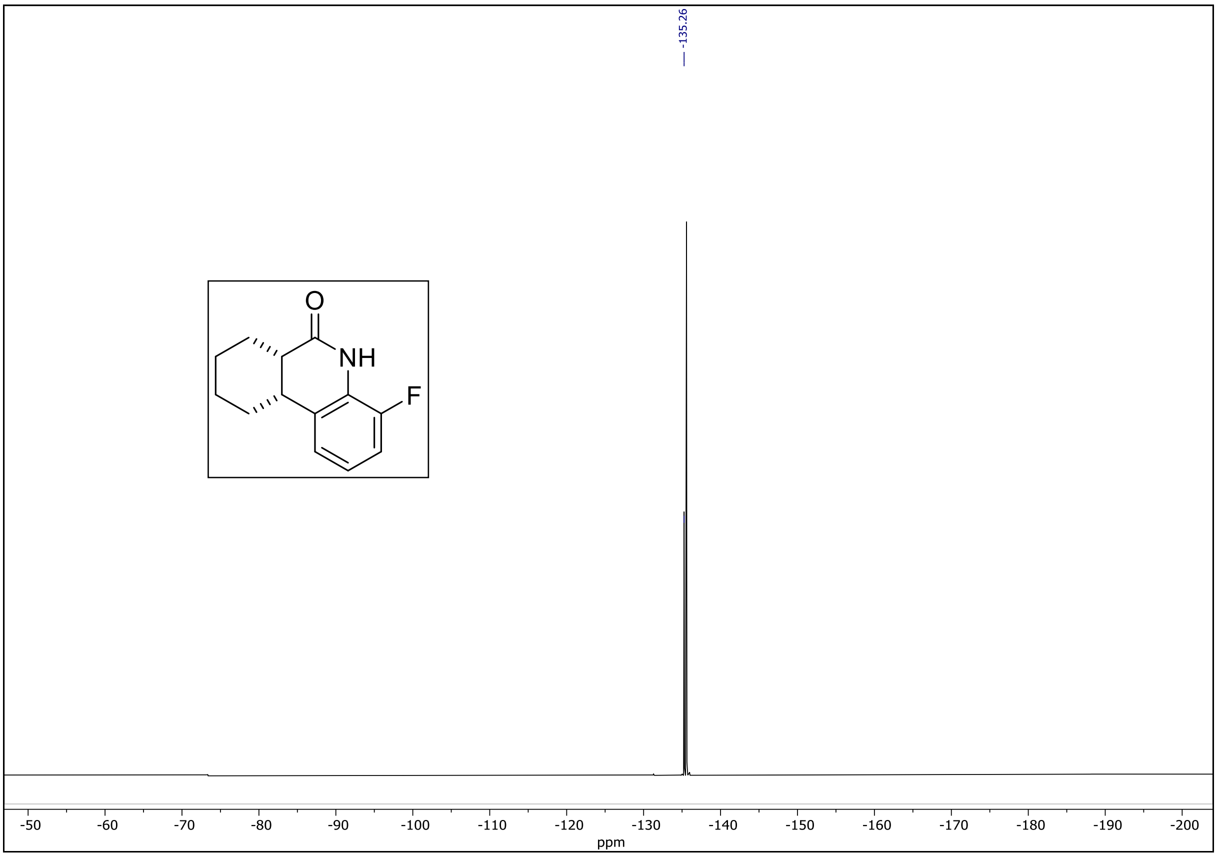

^{1}H NMR (500 MHz, $CDCl_3$): *trans*-**S6**

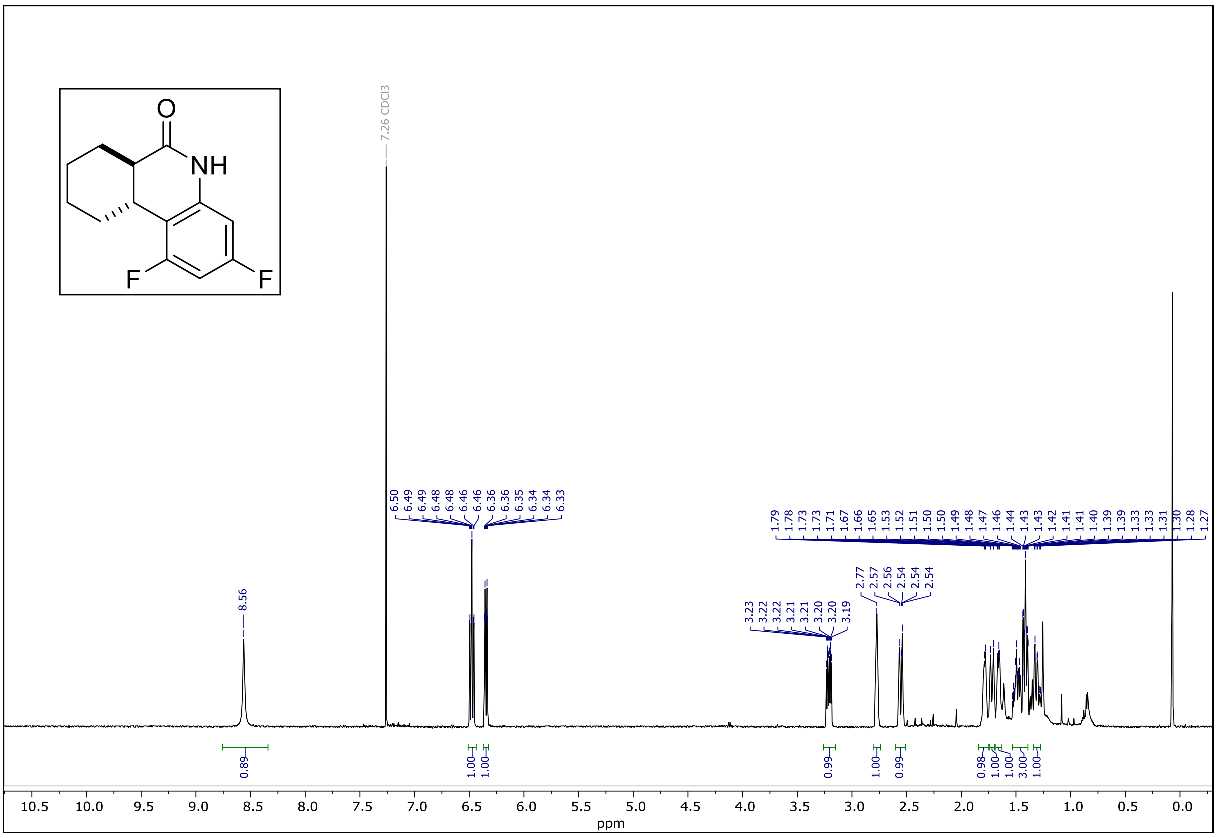

^{13}C NMR {^{19}F} (126 MHz, $CDCl_3$): *trans*-**S6**

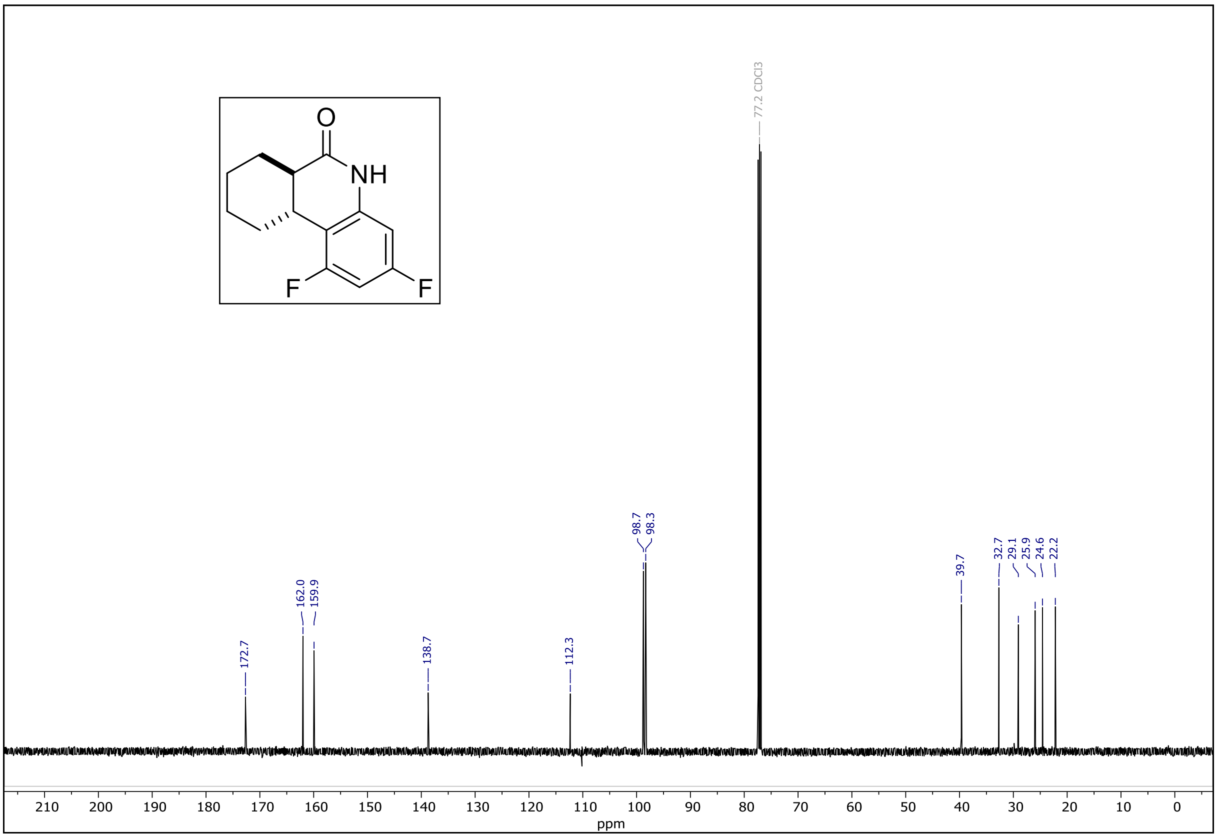

^{19}F NMR {^{1}H} (470 MHz, $CDCl_3$): *trans*-**S6**

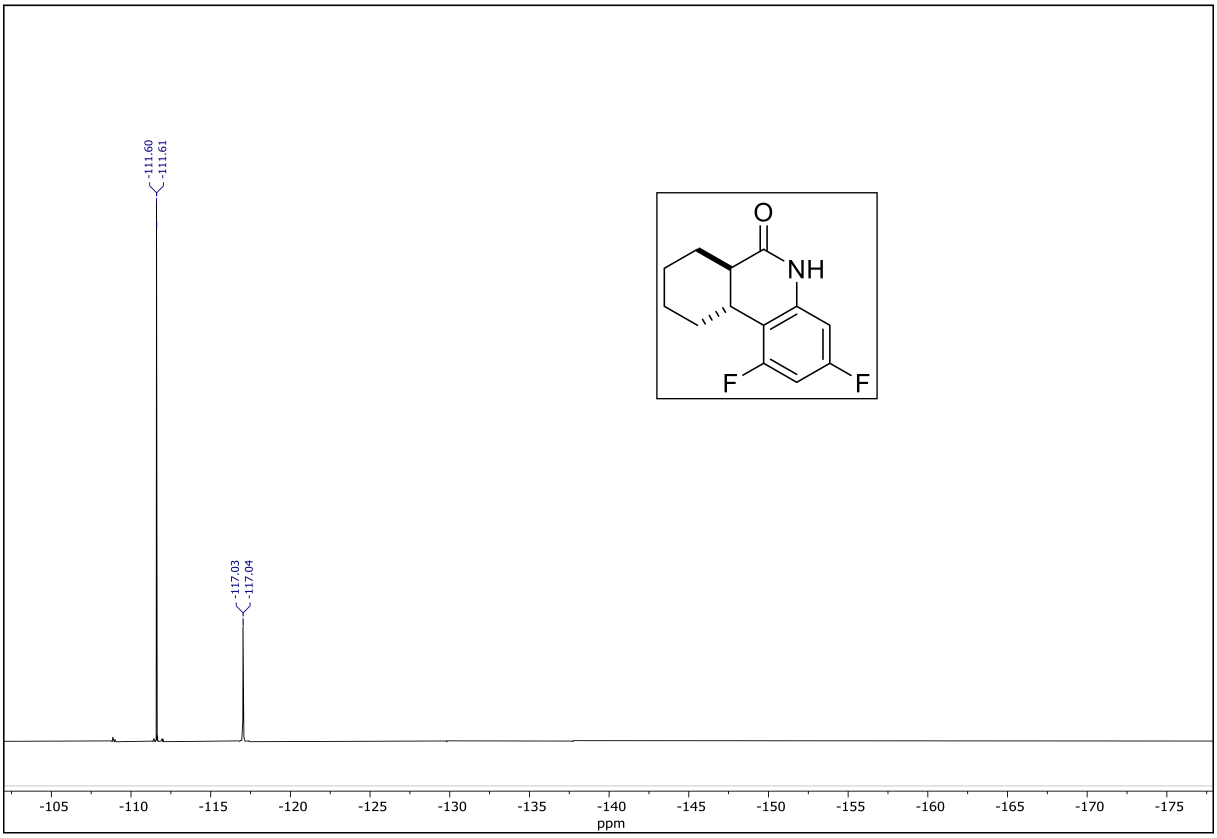

^{1}H NMR (500 MHz, $CDCl_3$): *cis*-**S6**

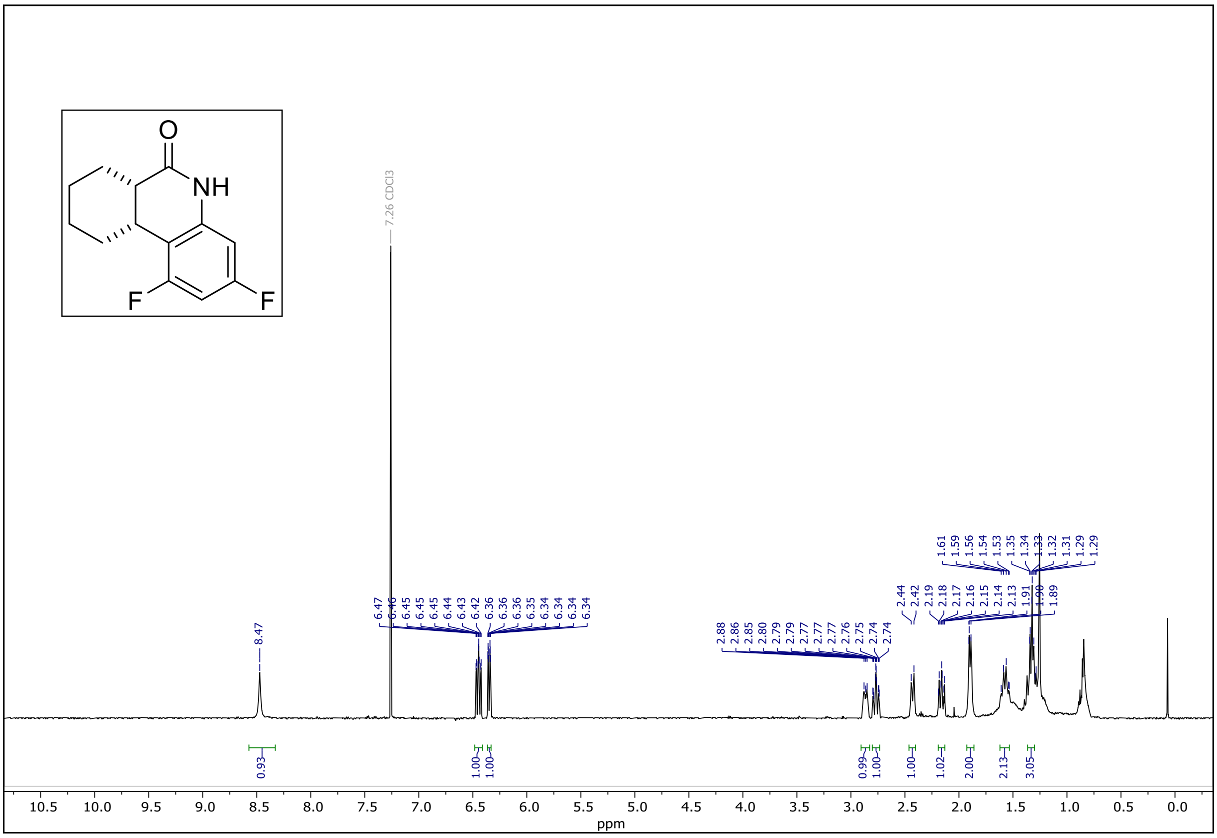

^{13}C NMR {^{19}F} (126 MHz, $CDCl_3$): *cis*-**S6**

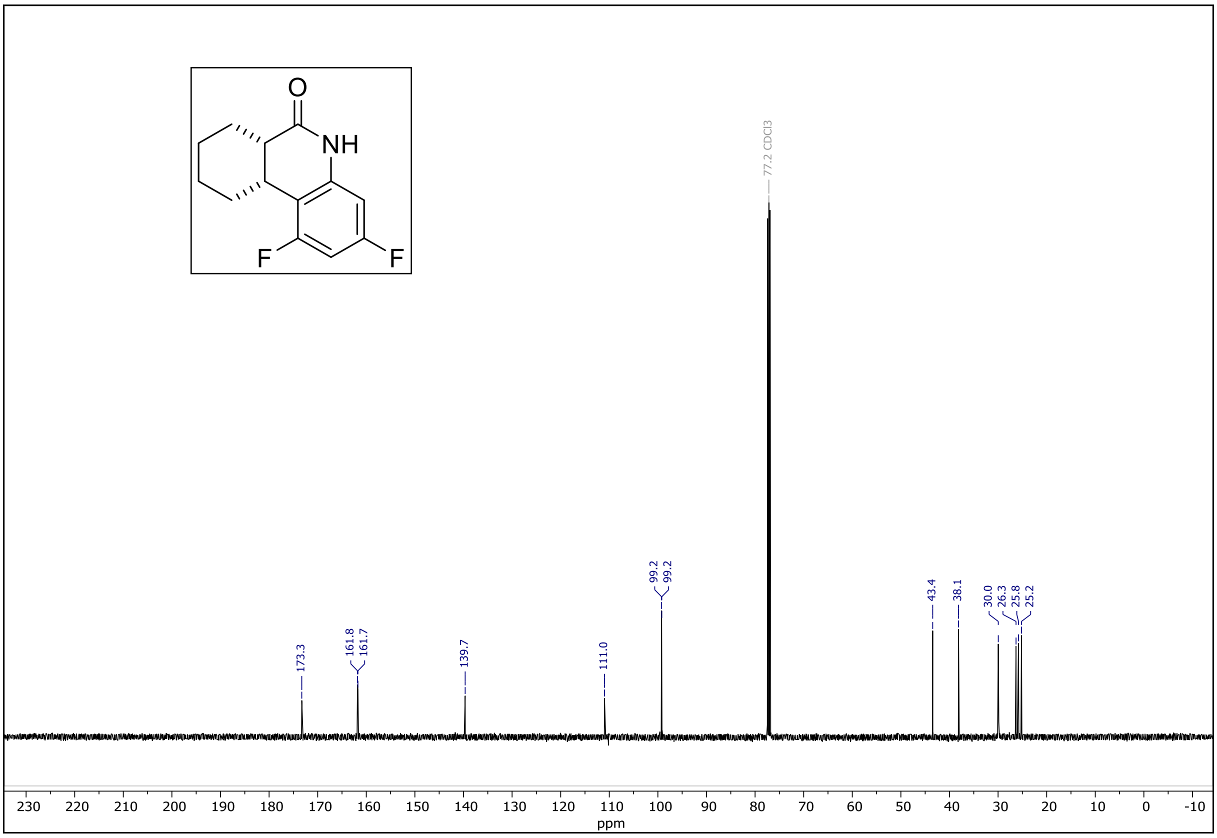

^{19}F NMR {^{1}H} (470 MHz, $CDCl_3$): *cis*-**S6**

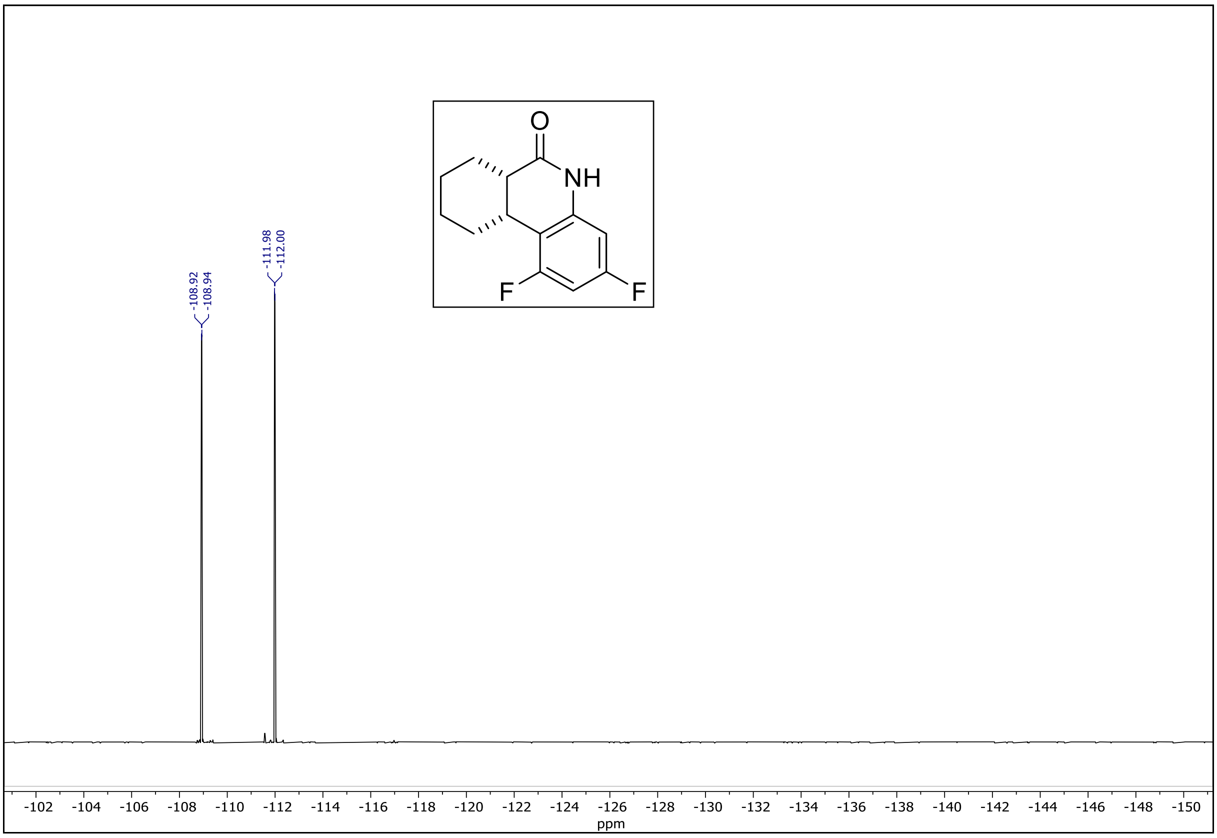

^{1}H NMR (500 MHz, $CDCl_3$): *trans*-3-Cl-**S7**

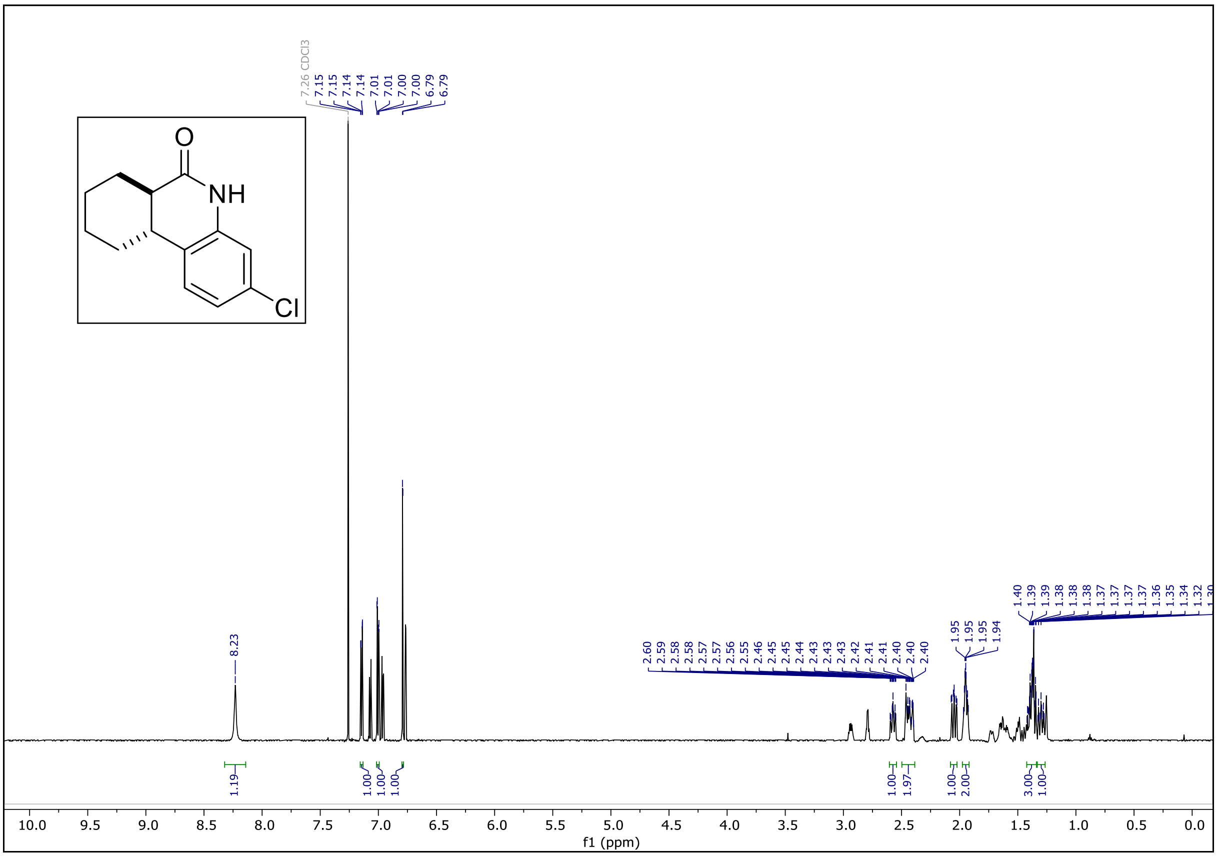

^{13}C NMR (126 MHz, $CDCl_3$): *trans*-3-Cl-**S7**

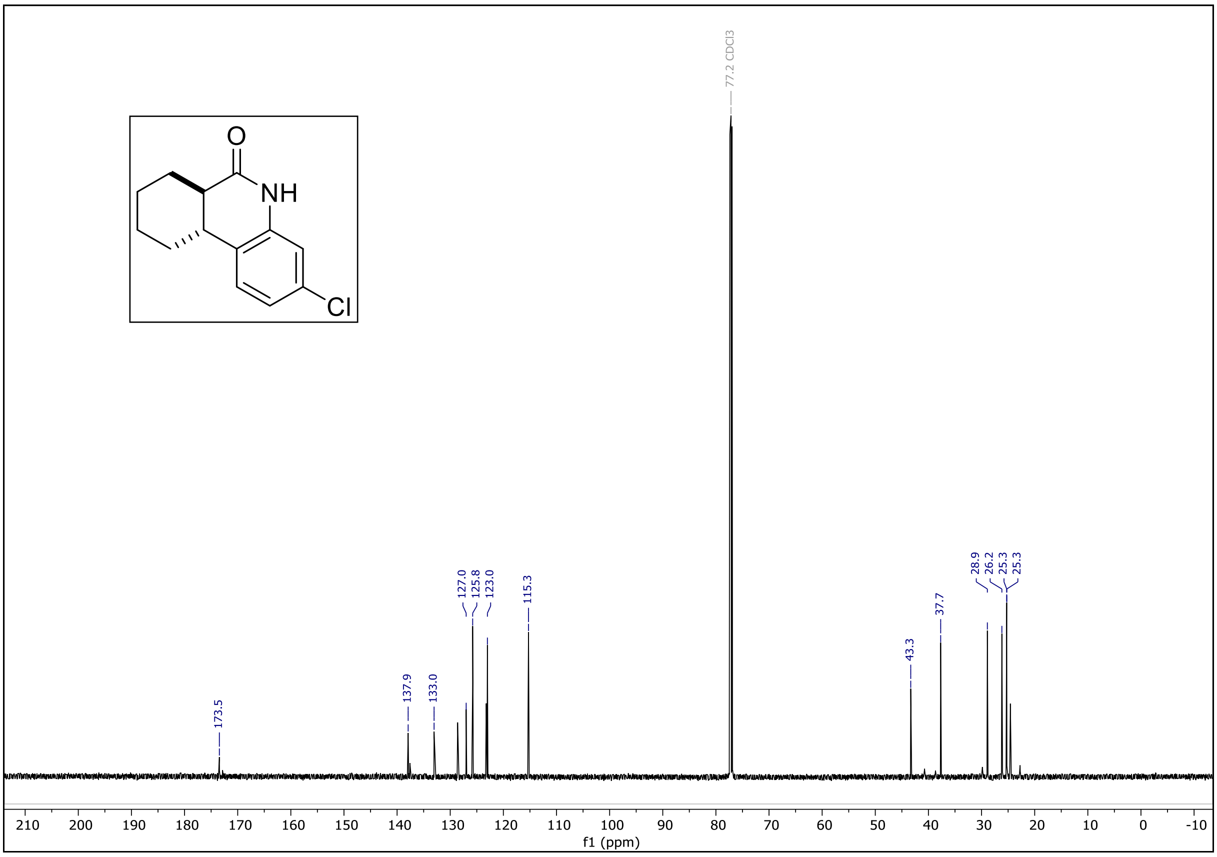

^{1}H NMR (500 MHz, $CDCl_3$): *cis*-3-Cl-**S7**

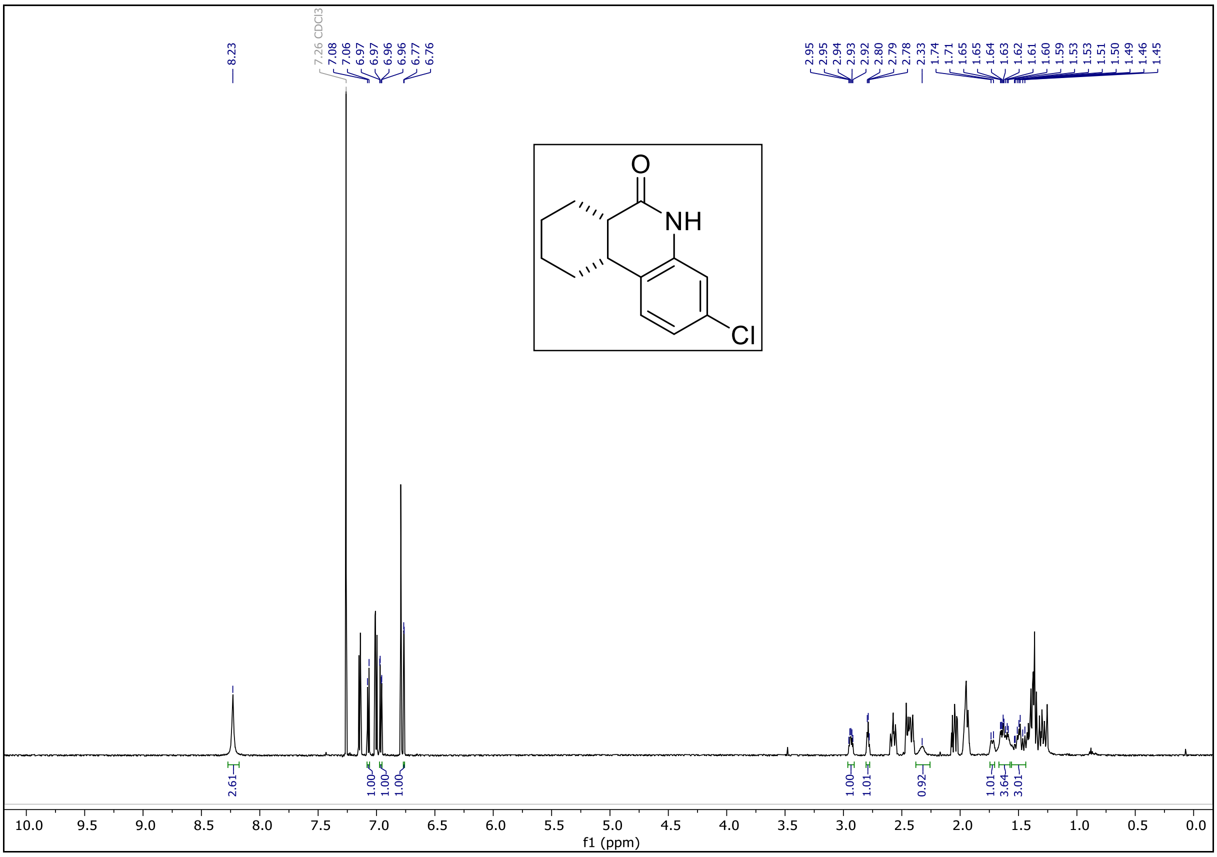

^{13}C NMR (126 MHz, $CDCl_3$): *cis*-3-Cl-**S7**

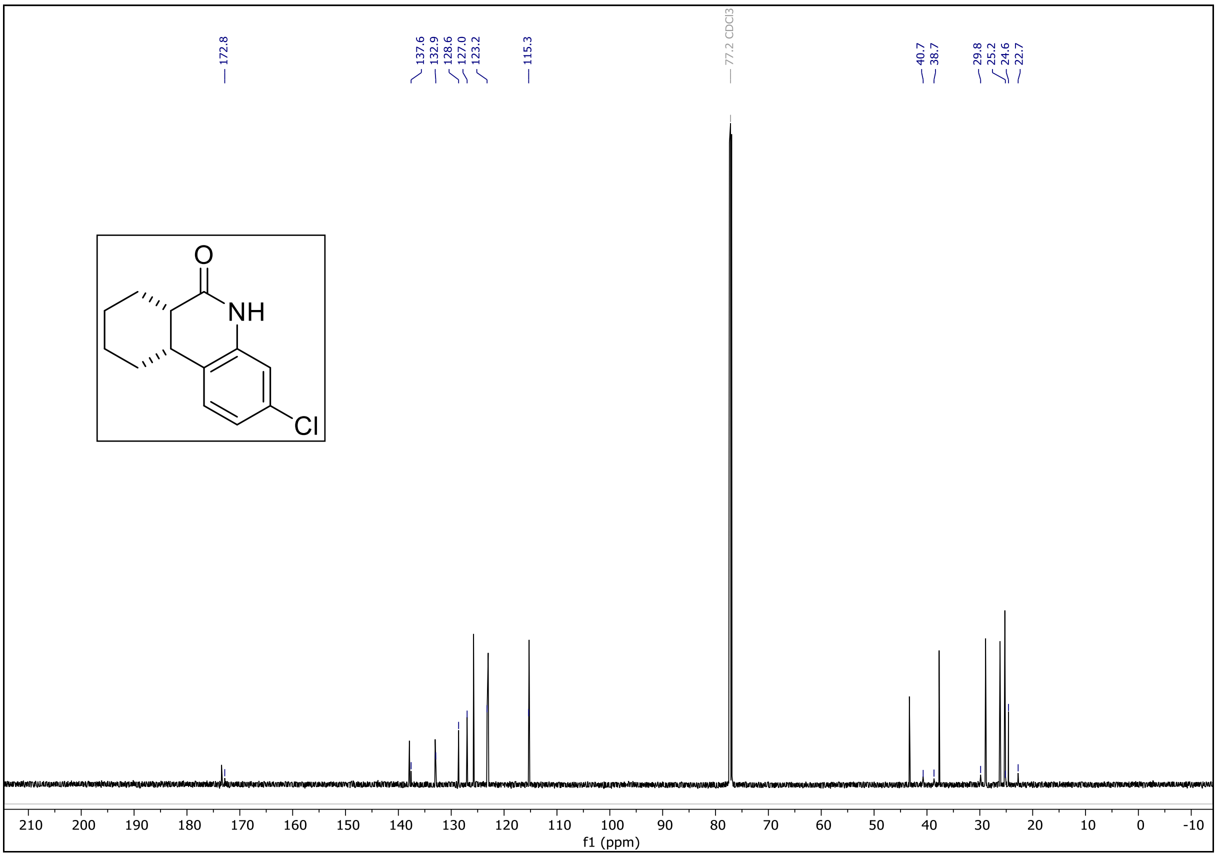

^{1}H NMR (500 MHz, $CDCl_3$): 1-Cl-**S7** (major)

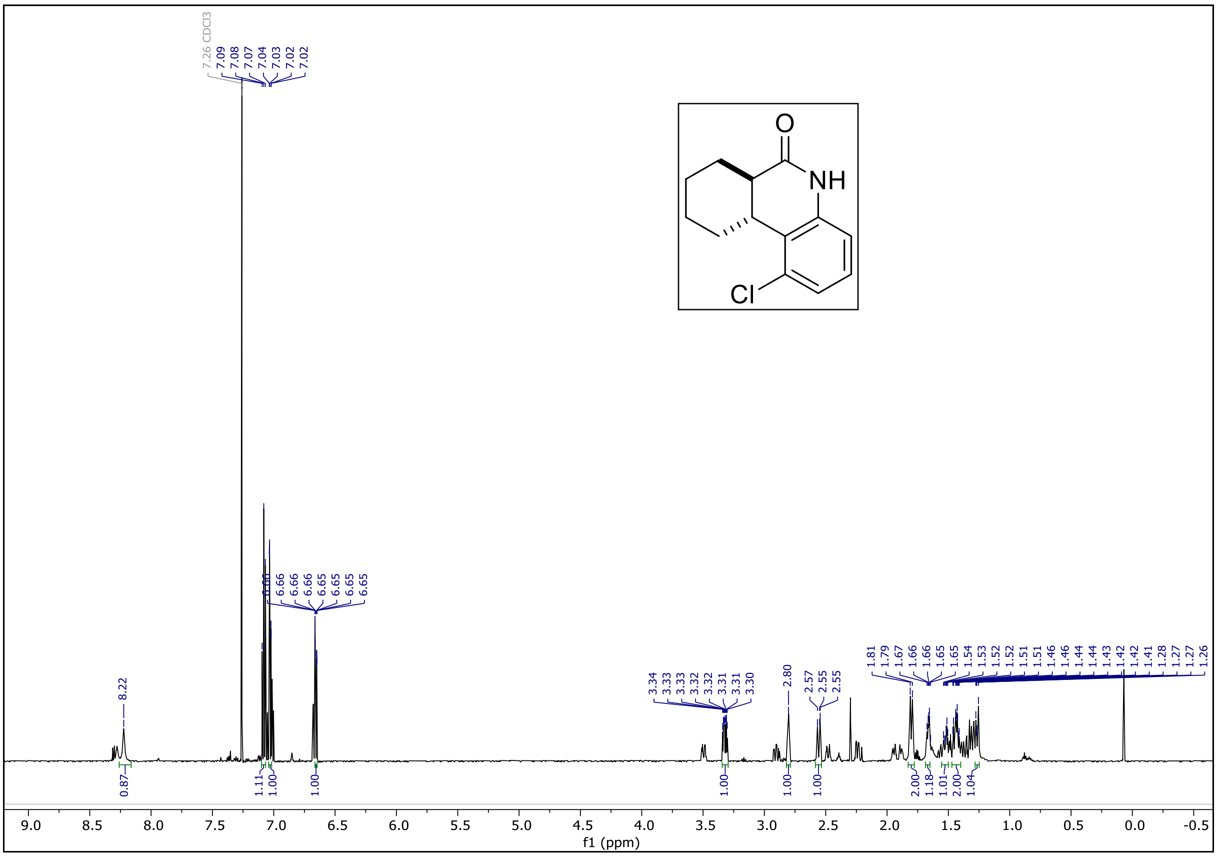

^{13}C NMR (126 MHz, $CDCl_3$): 1-Cl-**S7** (major)

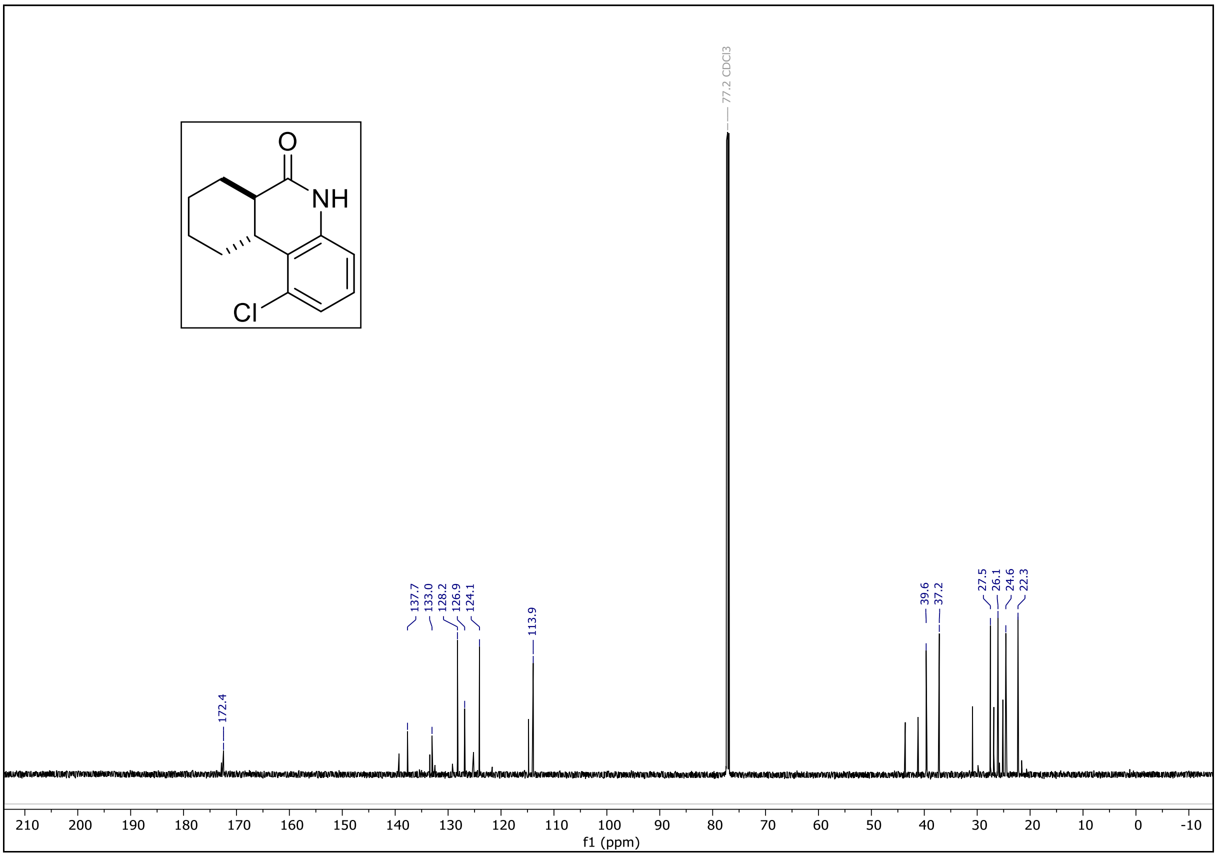

^{1}H NMR (500 MHz, $CDCl_3$): 1-Cl-**S7** (minor)

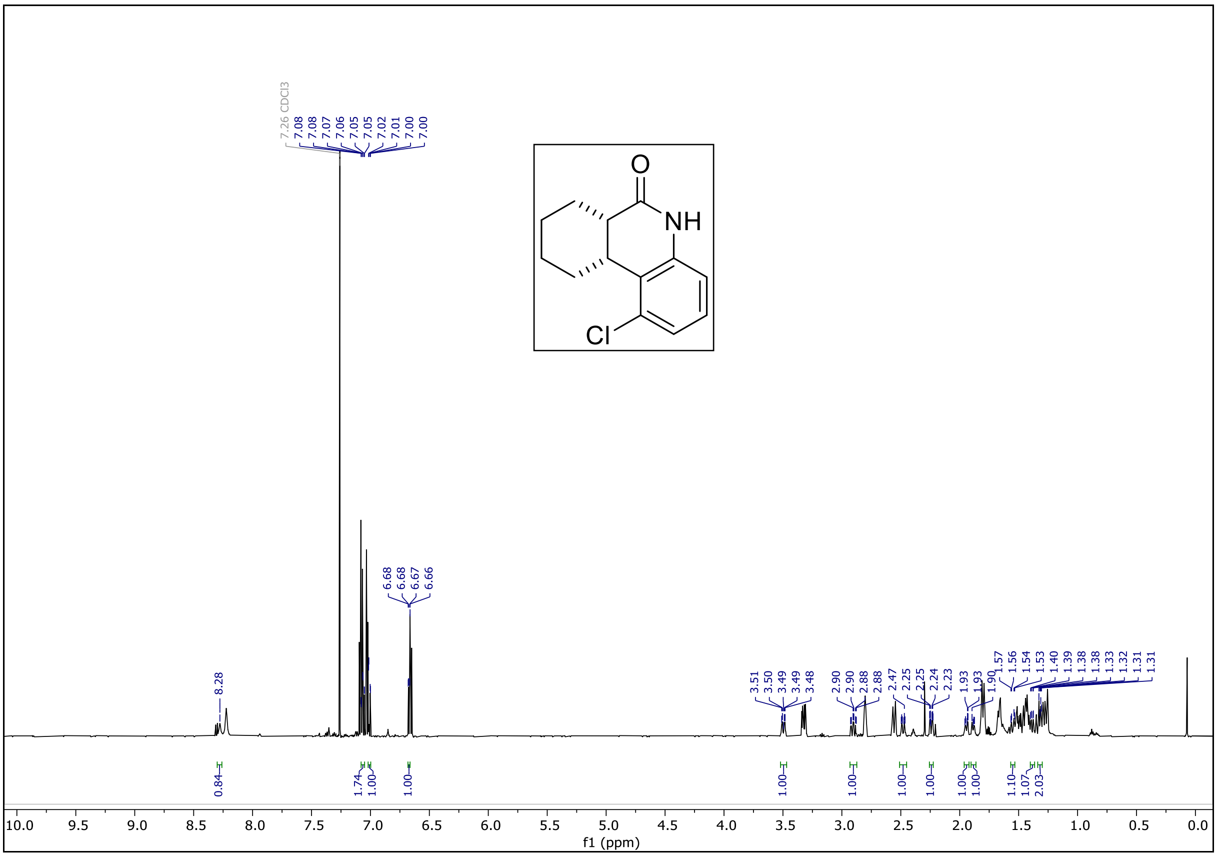

^{13}C NMR (126 MHz, $CDCl_3$): 1-Cl-**S7** (minor)

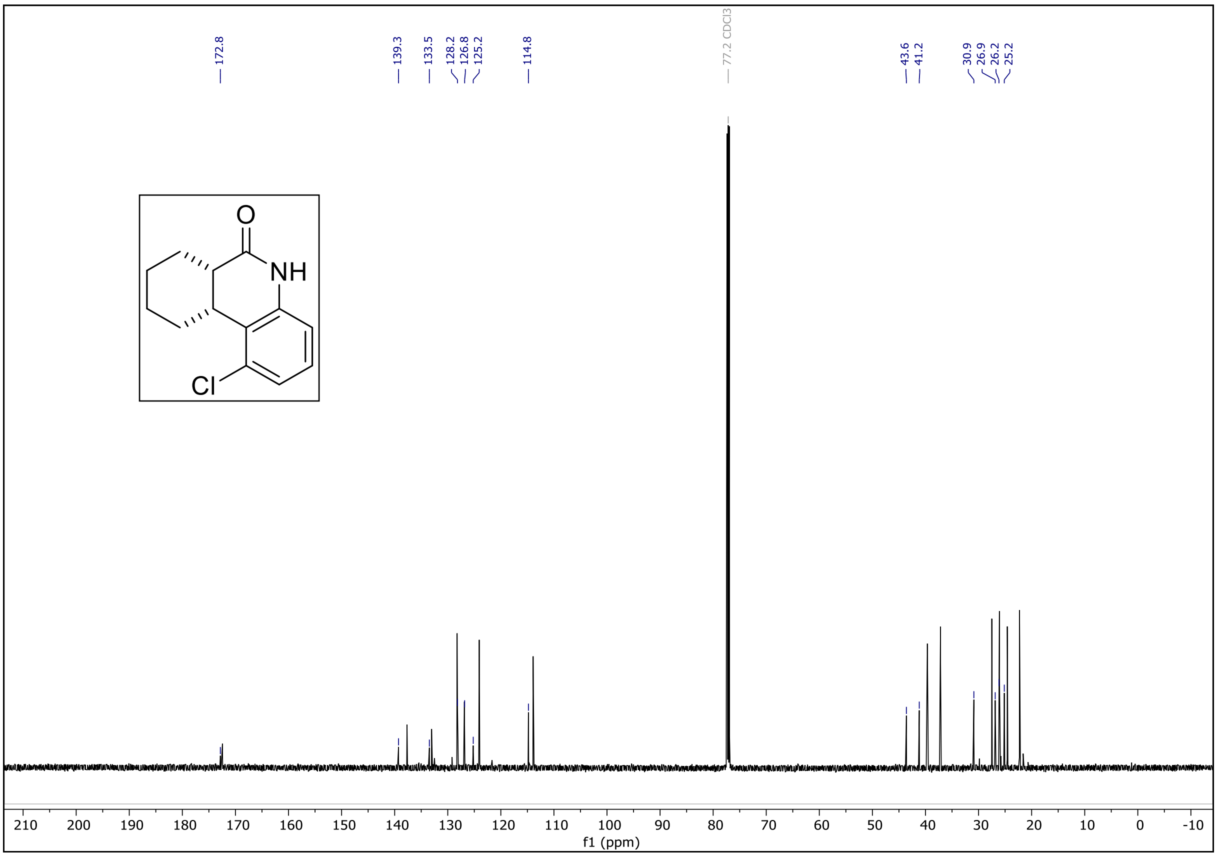

^{1}H NMR (600 MHz, $CDCl_3$): *trans*-**S8**

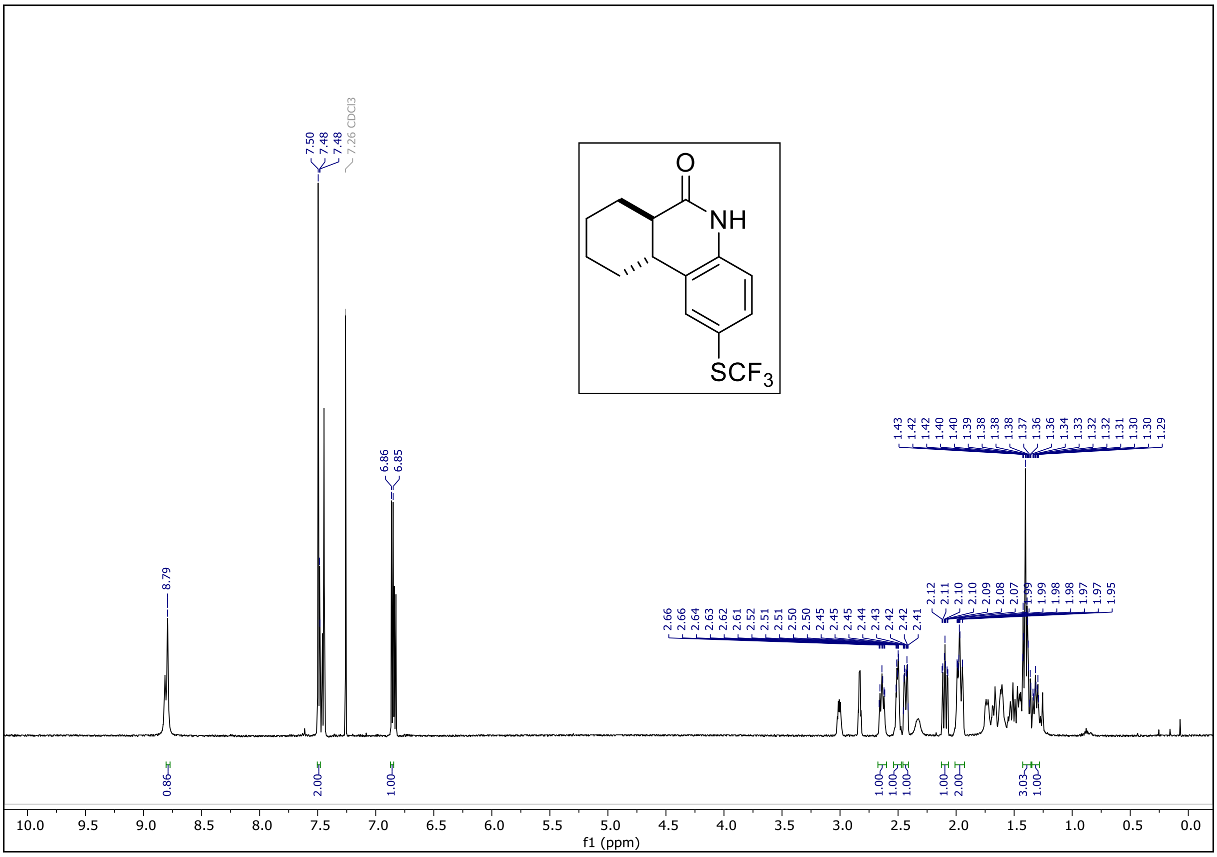

^{13}C NMR {^{19}F} (151 MHz, $CDCl_3$): *trans*-**S8**

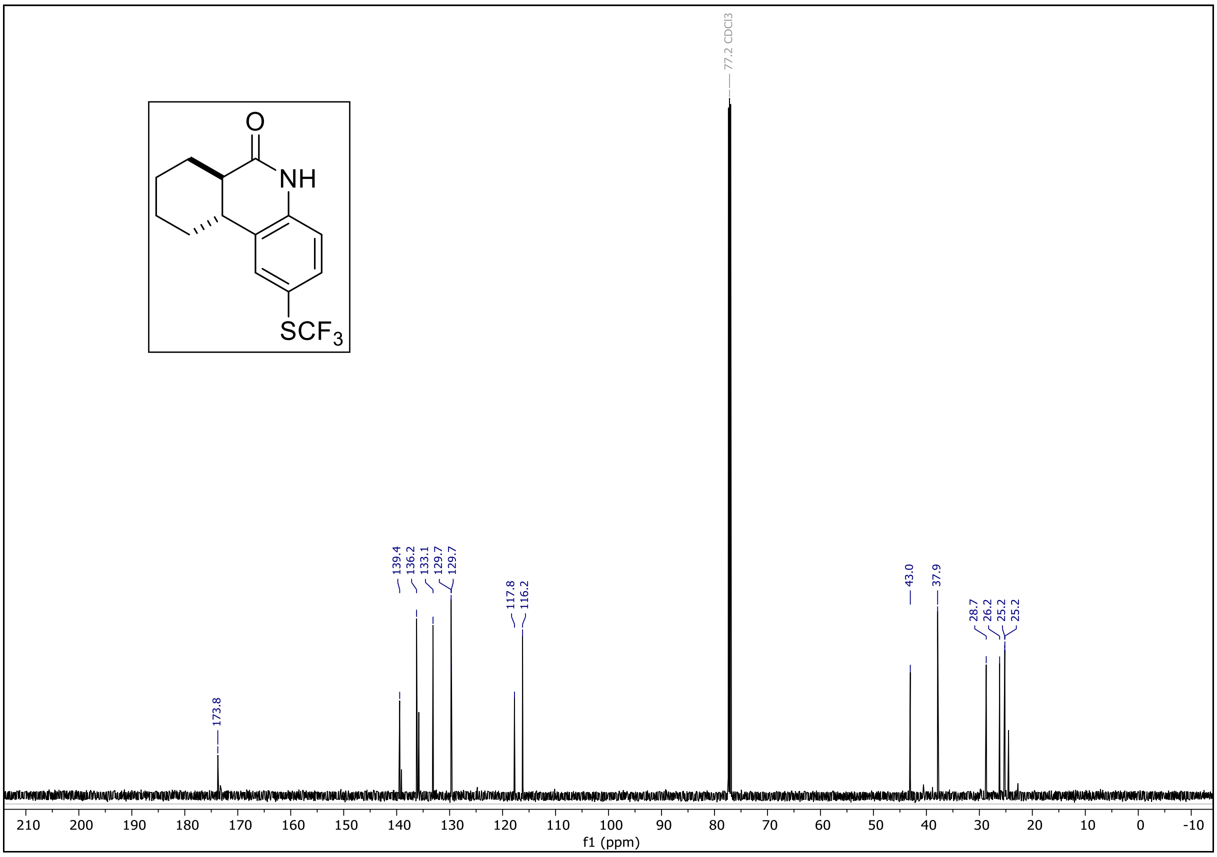

^{19}F NMR {^{1}H} (564 MHz, $CDCl_3$): *trans*-**S8**

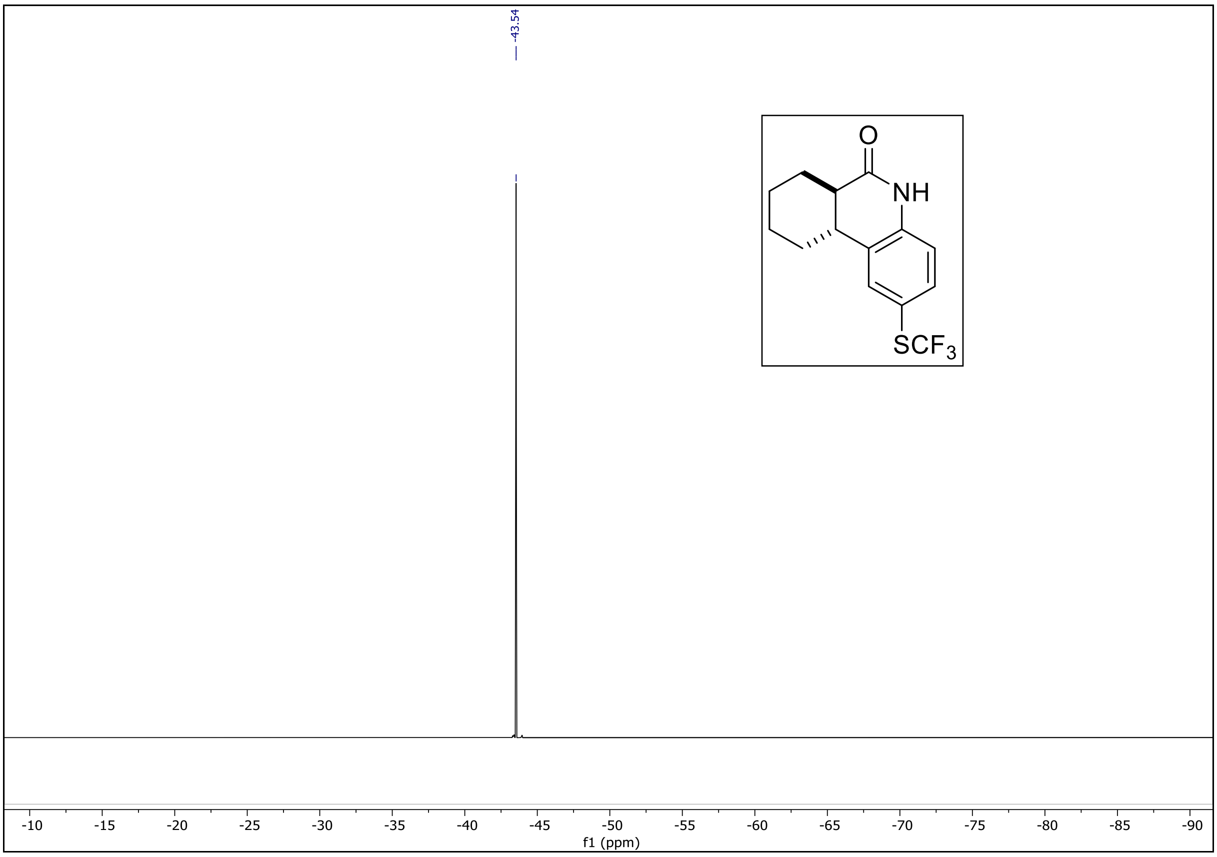

^{1}H NMR (600 MHz, $CDCl_3$): *cis*-**S8**

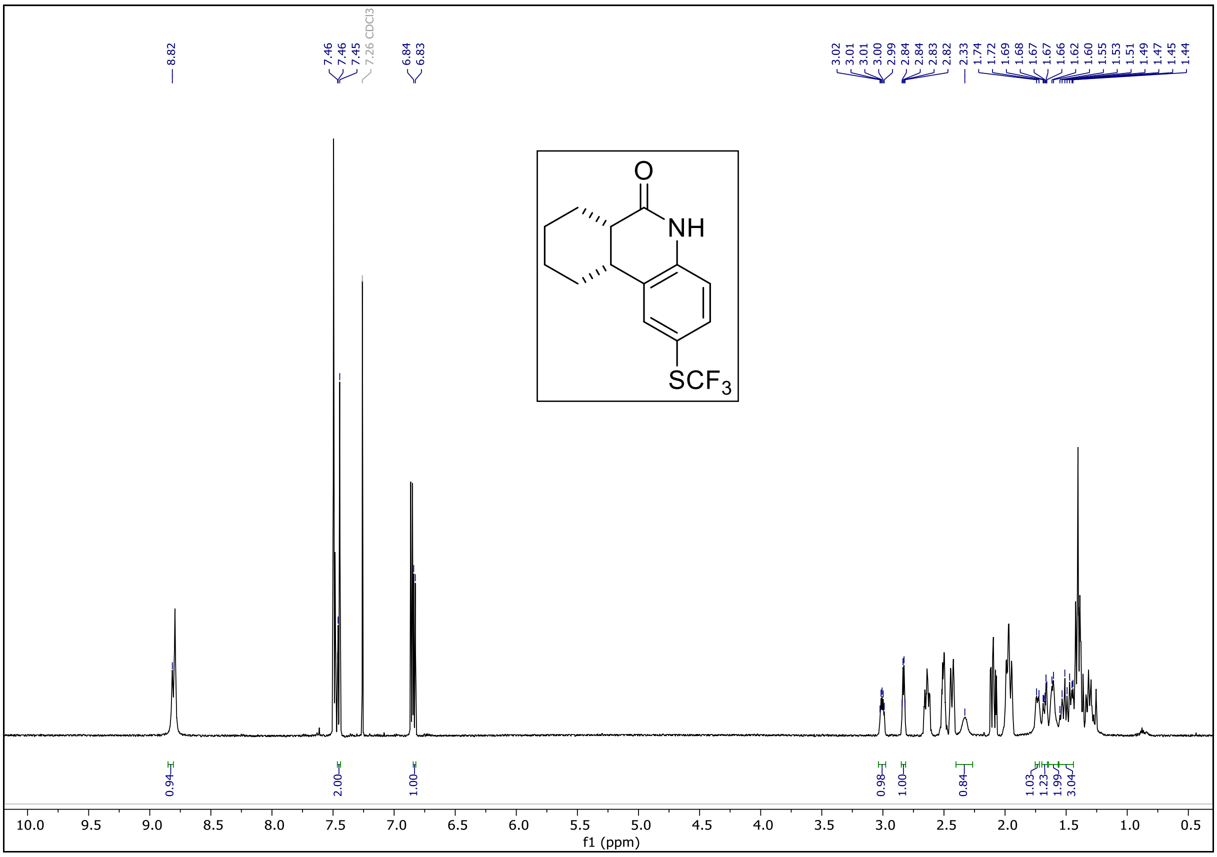

^{13}C NMR {^{19}F} (151 MHz, $CDCl_3$): *cis*-**S8**

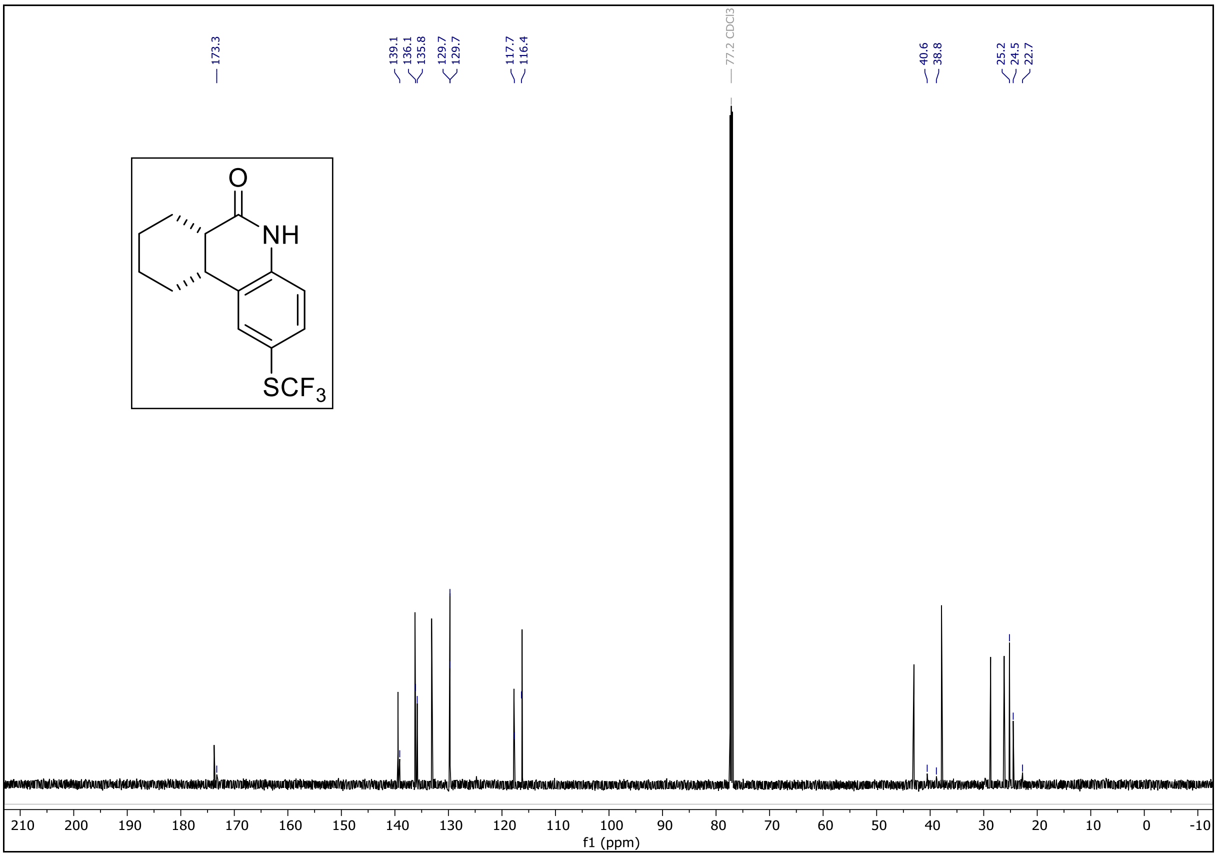

^{19}F NMR {^{1}H} (564 MHz, $CDCl_3$): *cis*-**S8**

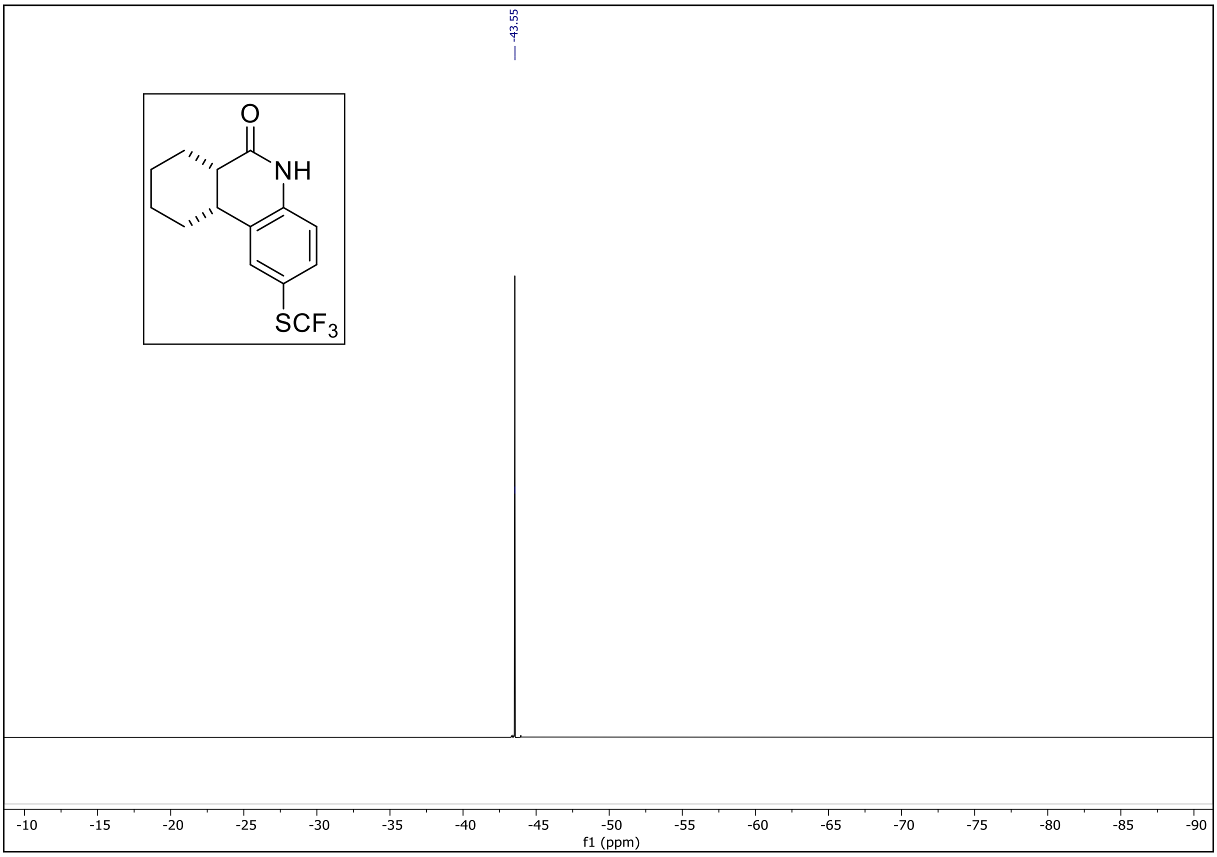

^{1}H NMR (500 MHz, $CDCl_3$): *trans*-**S9**

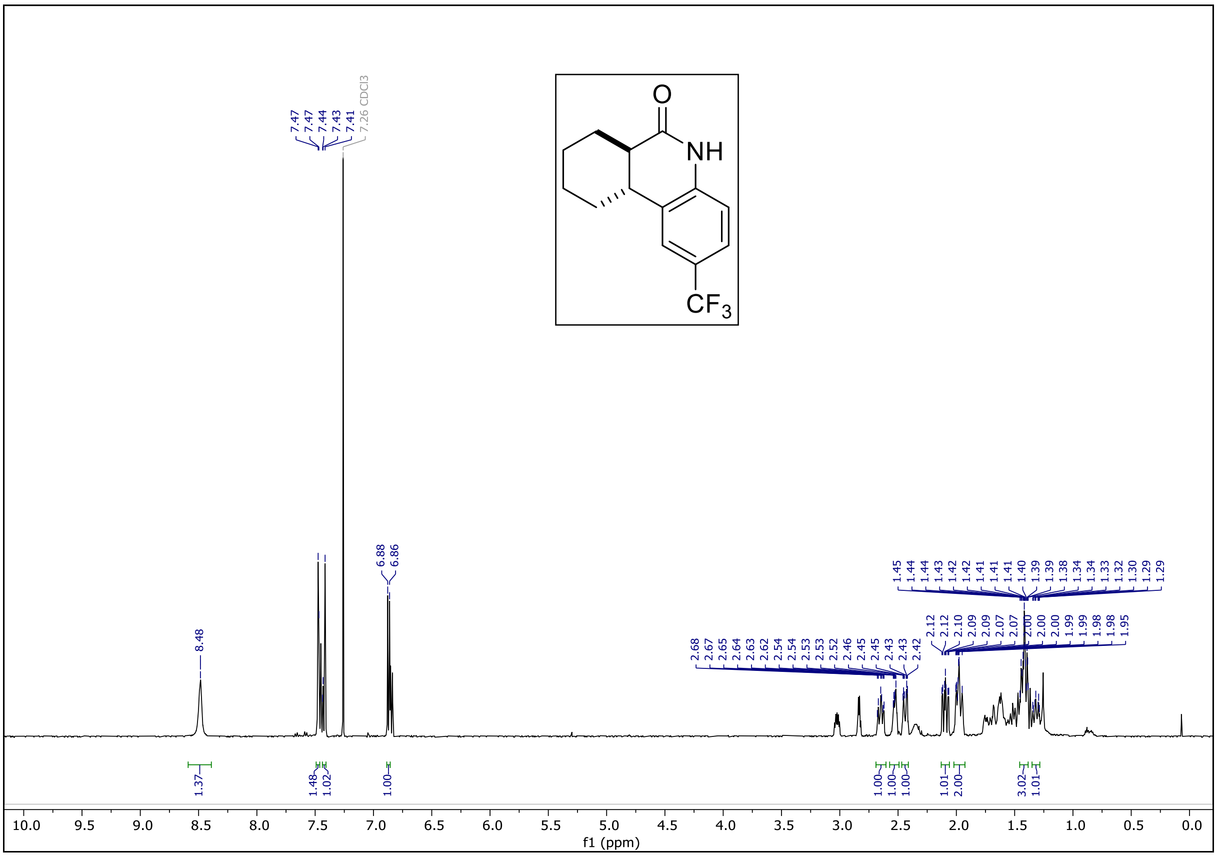

^{13}C NMR {^{19}F} (126 MHz, $CDCl_3$): *trans*-**S9**

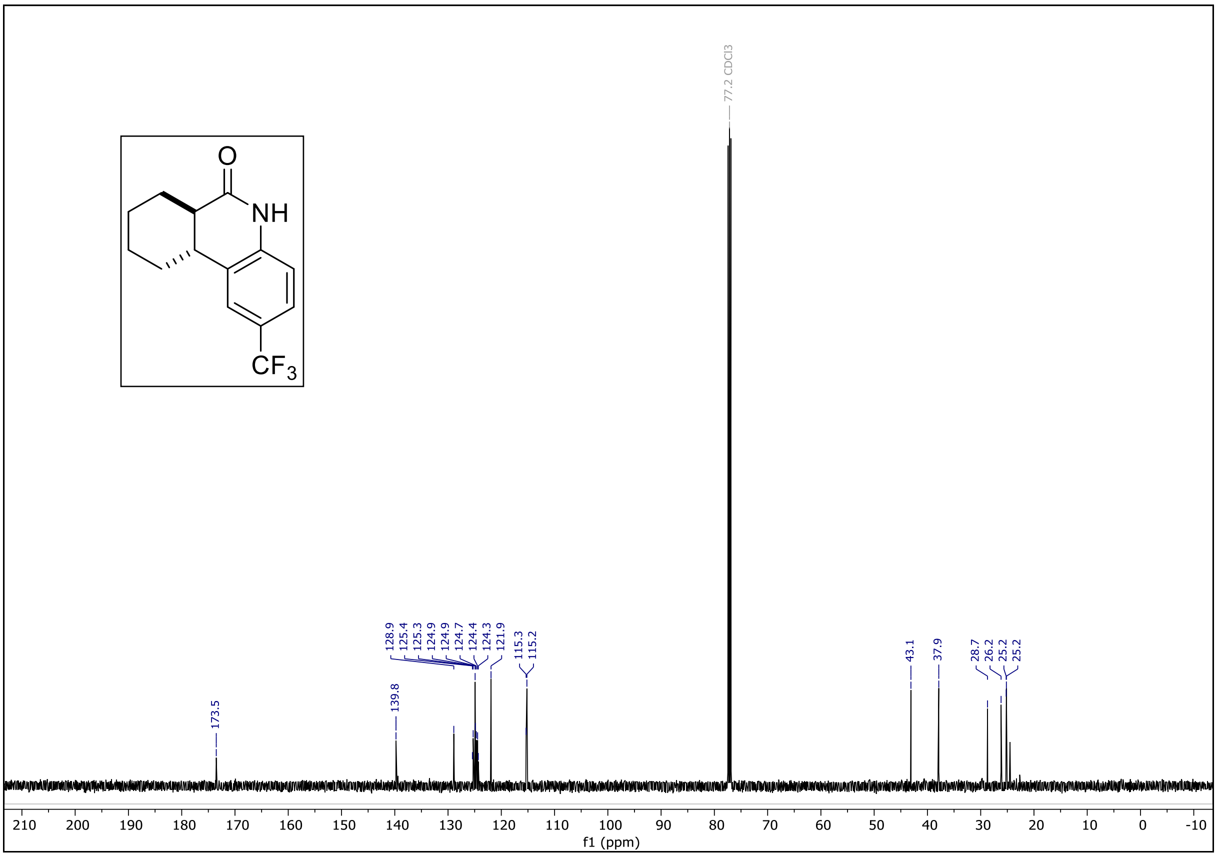

^{19}F NMR {^{1}H} (470 MHz, $CDCl_3$): *trans*-**S9**

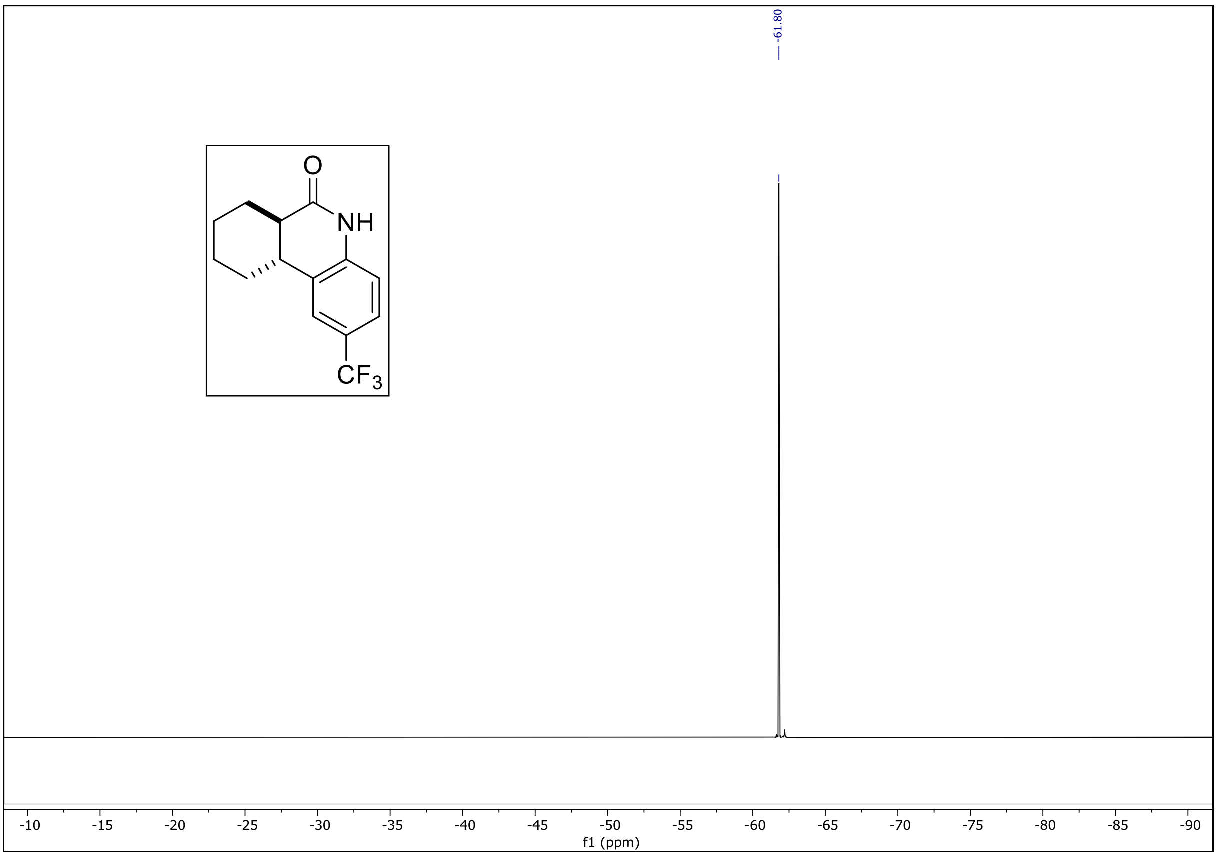

^{1}H NMR (500 MHz, $CDCl_3$): *cis*-**S9**

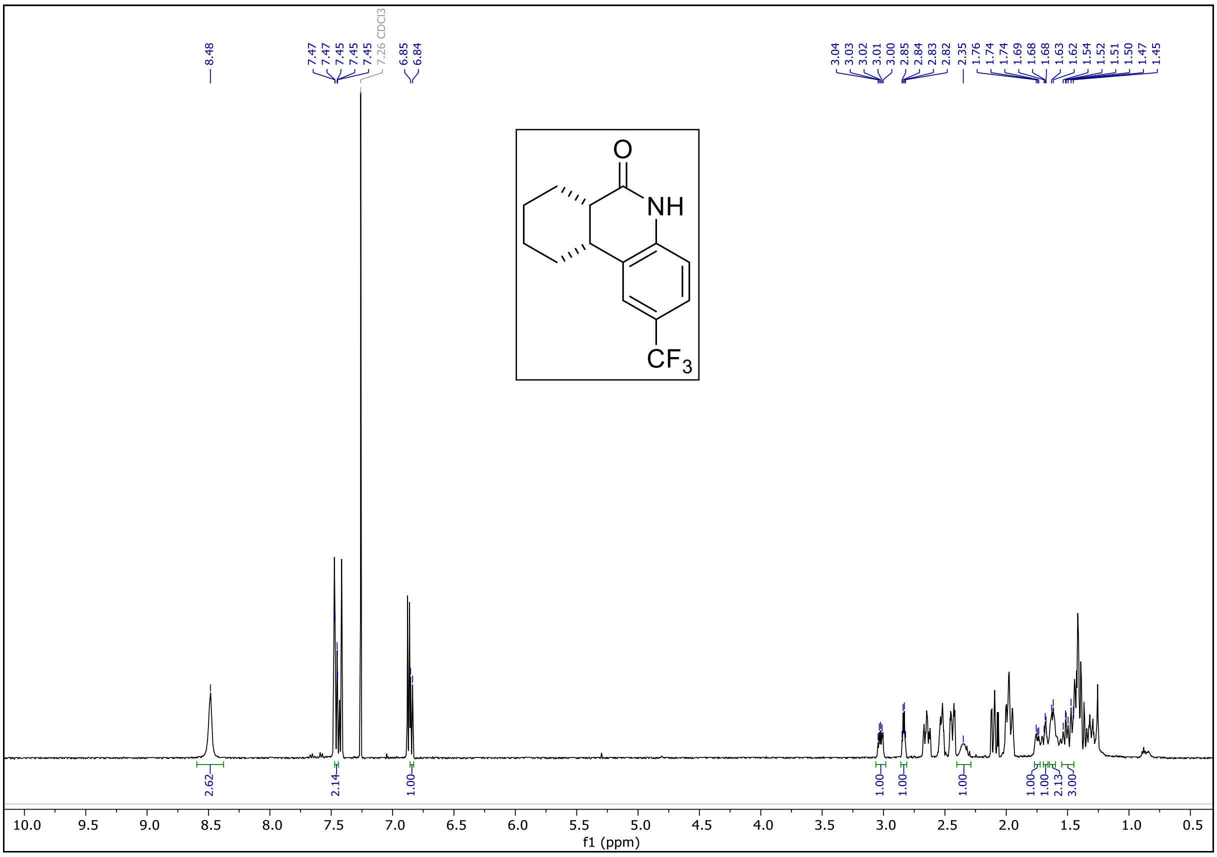

^{13}C NMR {^{19}F} (126 MHz, $CDCl_3$): *cis*-**S9**

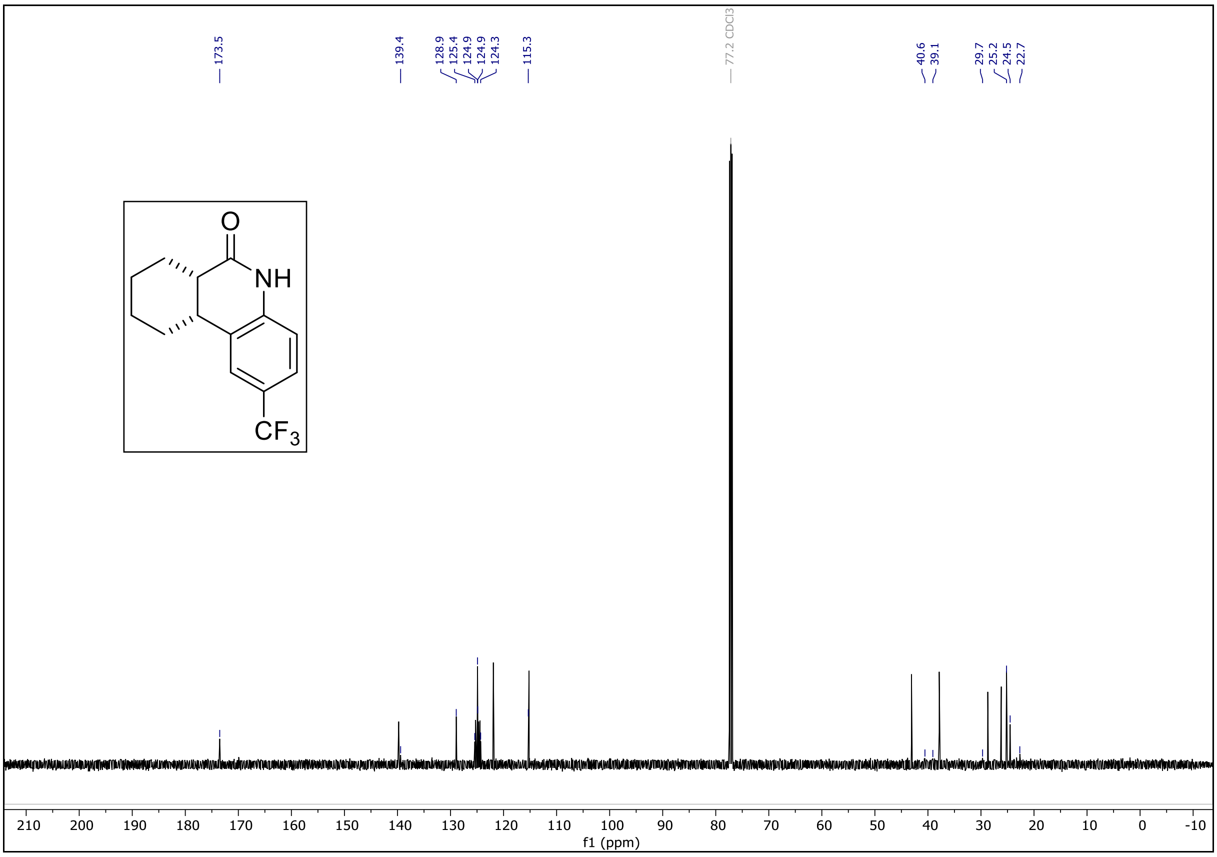

^{19}F NMR {^{1}H} (470 MHz, $CDCl_3$): *cis*-**S9**

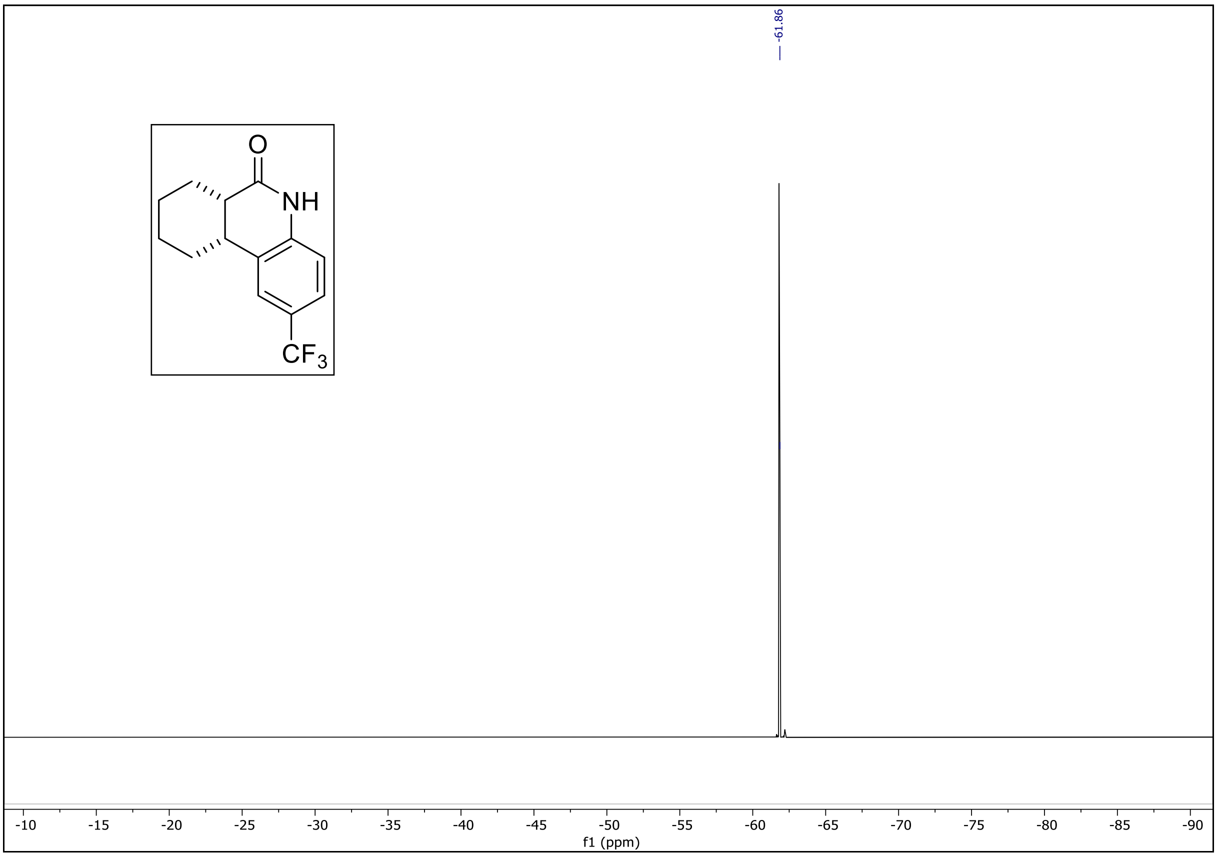

^{1}H NMR (500 MHz, $CDCl_3$): *trans*-**S10**

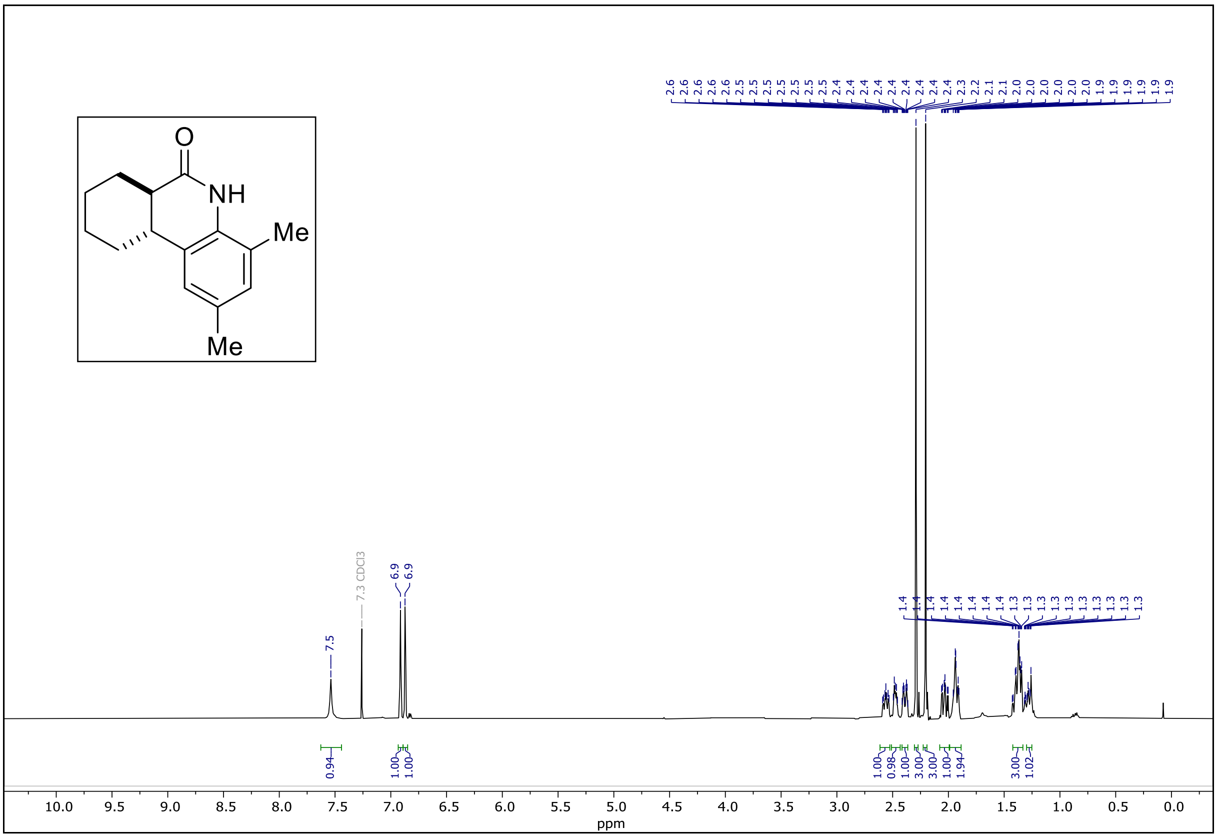

^{13}C NMR (126 MHz, $CDCl_3$): *trans*-**S10**

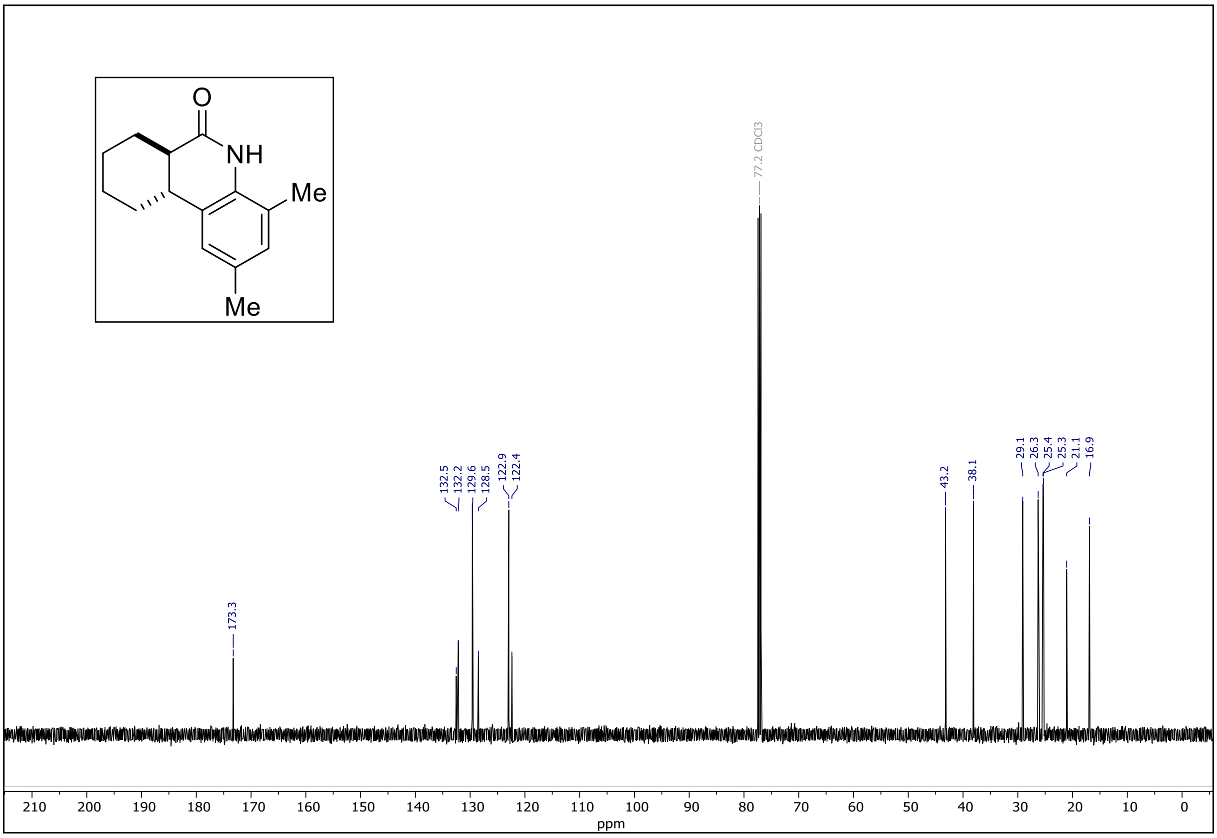

^{1}H NMR (500 MHz, $CDCl_3$): *cis*-**S10**

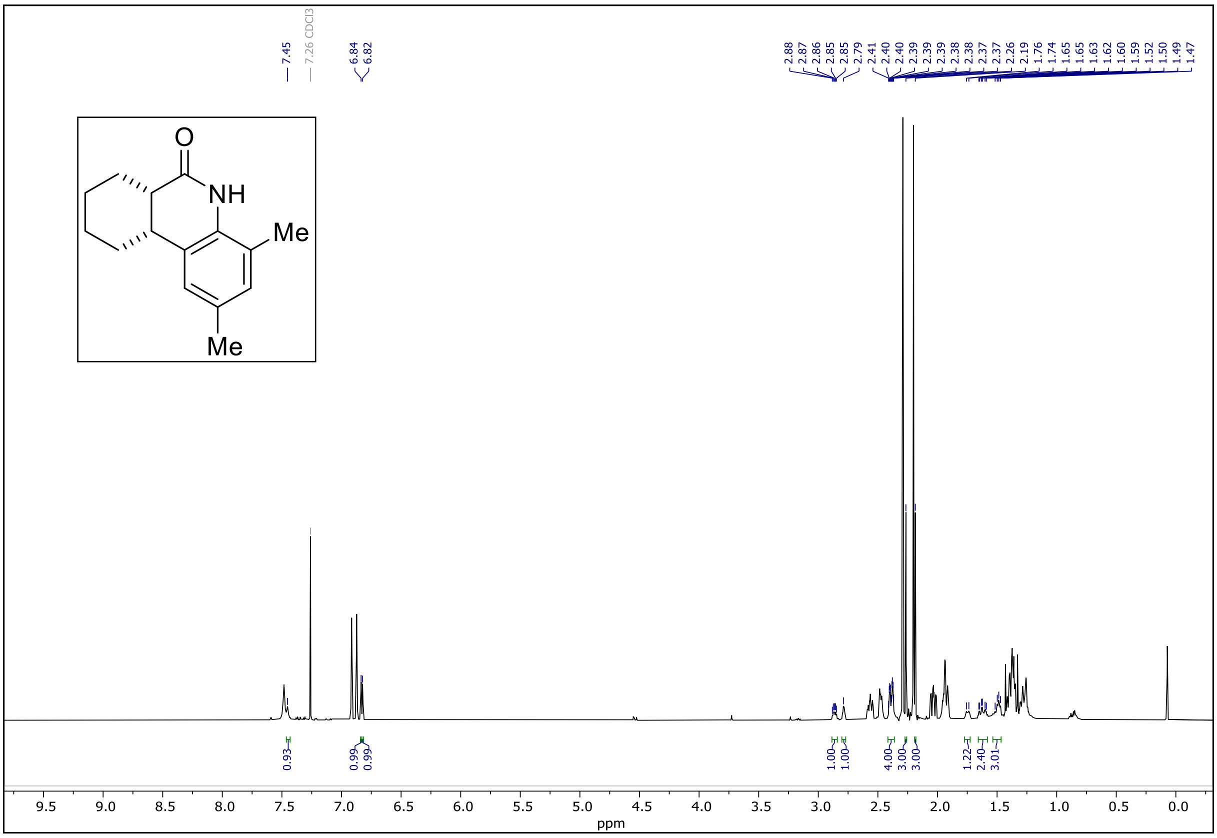

^{13}C NMR (126 MHz, $CDCl_3$): *cis*-**S10**

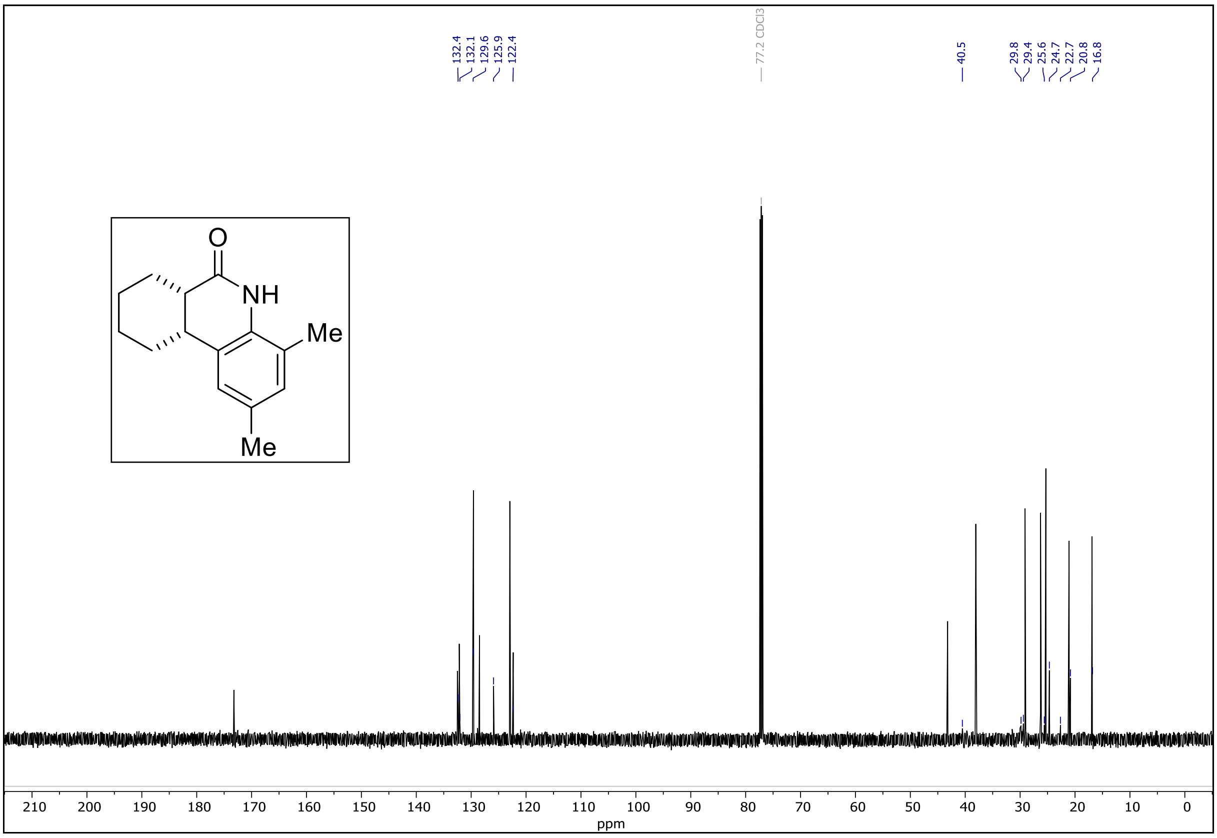

^{1}H NMR (400 MHz, $CDCl_3$): **S11**

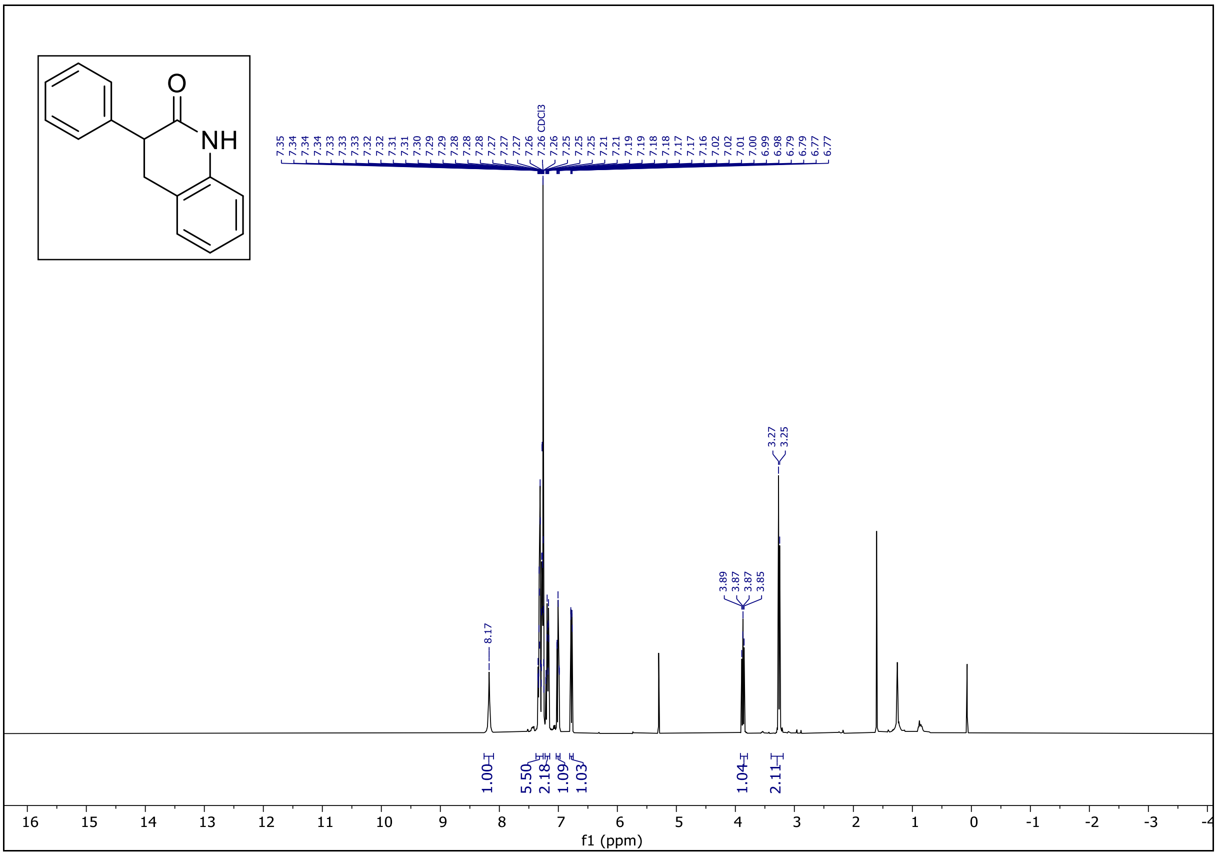

^{1}H NMR (400 MHz, $CDCl_3$): **S12**

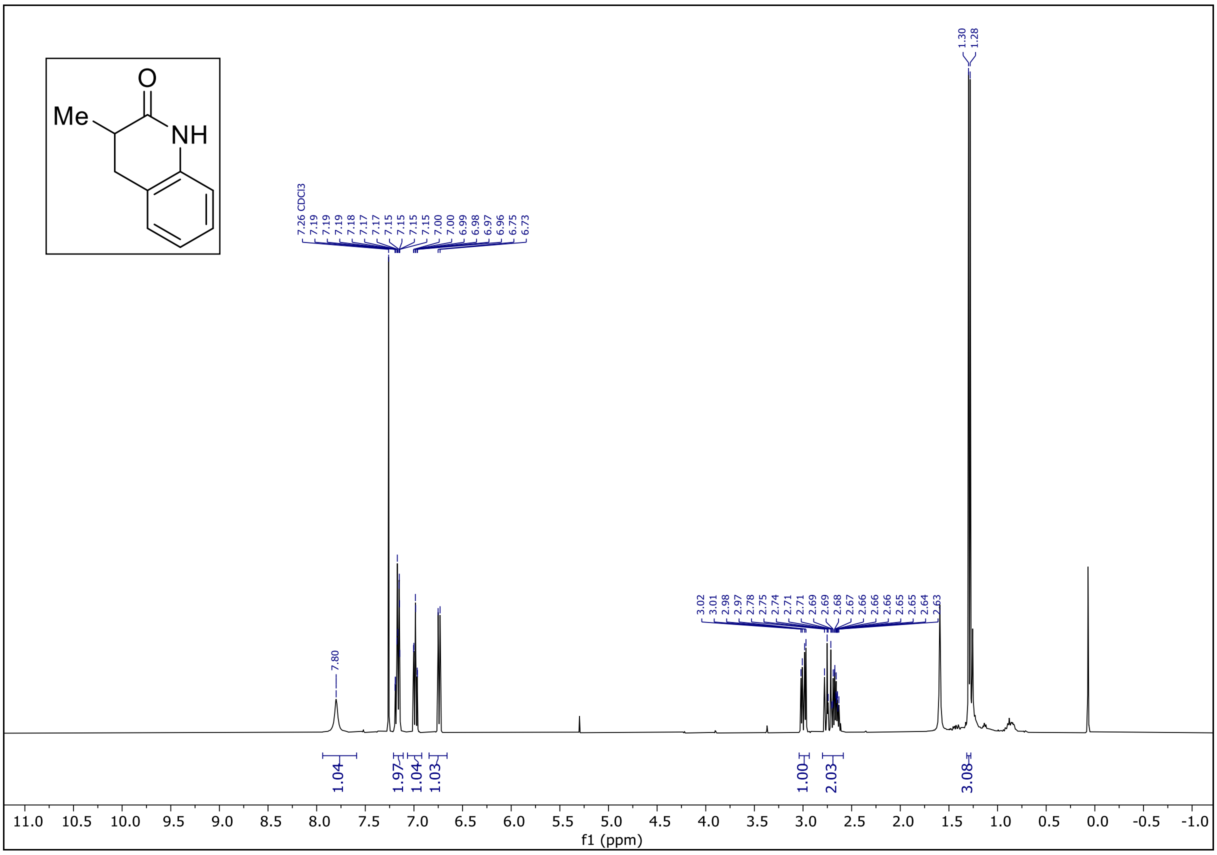

References

1. Fulmer, G. R. *et al.* NMR Chemical Shifts of Trace Impurities: Common Laboratory Solvents, Organics, and Gases in Deuterated Solvents Relevant to the Organometallic Chemist. *Organometallics* **29,** 2176–2179; 10.1021/om100106e (2010).

2. Onneken, C. *et al.* Light-enabled deracemization of cyclopropanes by Al-salen photocatalysis. *Nature* **621,** 753–759; 10.1038/s41586-023-06407-8 (2023).

3. Bach, T., Grosch, B., Strassner, T. & Herdtweck, E. Enantioselective 6pi-photocyclization reaction of an acrylanilide mediated by a chiral host. Interplay between enantioselective ring closure and enantioselective protonation. *J. Org. Chem.* **68,** 1107–1116; 10.1021/jo026602d (2003).

4. Huo, Y.-W., Yao, L., Qi, X. & Wu, X.-F. Nickel-catalyzed reductive aminocarbonylation of vinyl triflates with nitro compounds for the synthesis of α,β-unsaturated amides. *Org. Chem. Front.* **8,** 6974–6978; 10.1039/D1QO01508G (2021).

5. Zhang, S., Neumann, H. & Beller, M. Pd-Catalyzed Carbonylation of Vinyl Triflates To Afford α,β-Unsaturated Aldehydes, Esters, and Amides under Mild Conditions. *Org. Lett.* **21,** 3528–3532; 10.1021/acs.orglett.9b00765 (2019).

6. Cheung, C. W., Surry, D. S. & Buchwald, S. L. Mild and highly selective palladium-catalyzed monoarylation of ammonia enabled by the use of bulky biarylphosphine ligands and palladacycle precatalysts. *Org. Lett.* **15,** 3734–3737; 10.1021/ol401612c (2013).

7. Yamada, S., Okuda, M. & Yamamoto, N. Tetrabutylammonium-assisted diastereoselective [6π]-photocyclization of acrylanilides. *Tetrahedron Lett.* **56,** 2098–2101; 10.1016/j.tetlet.2015.03.029 (2015).

8. Jung, M. E. & Deng, G. Synthesis of the 1-monoester of 2-ketoalkanedioic acids, for example, octyl α-ketoglutarate. *J. Org. Chem.* **77,** 11002–11005; 10.1021/jo302308q (2012).

9. Hell, S. M. *et al.* Silyl Radical-Mediated Activation of Sulfamoyl Chlorides Enables Direct Access to Aliphatic Sulfonamides from Alkenes. *J. Am. Chem. Soc.* **142,** 720–725; 10.1021/jacs.9b13071 (2020).

10. Peil, S. & Fürstner, A. Mechanistic Divergence in the Hydrogenative Synthesis of Furans and Butenolides: Ruthenium Carbenes Formed by gem-Hydrogenation or through

Carbophilic Activation of Alkynes. *Angew. Chem. Int. Ed.* **58,** 18476–18481; 10.1002/anie.201912161 (2019).

11. Jolit, A., Vazquez-Rodriguez, S., Yap, G. P. A. & Tius, M. A. Diastereospecific nazarov cyclization of fully substituted dienones: generation of vicinal all-carbon-atom quaternary stereocenters. *Angew. Chem. Int. Ed.* **52,** 11102–11105; 10.1002/anie.201305218 (2013).
12. Wan, C. *et al.* Benzyne Polyfunctionalization via a Tandem C-C σ-Bond Insertion and Photo-Nazarov Cyclization. *Org. Lett.* **24,** 7276–7281; 10.1021/acs.orglett.2c02652 (2022).
13. Ralph, M. J., Harrowven, D. C., Gaulier, S., Ng, S. & Booker-Milburn, K. I. The profound effect of the ring size in the electrocyclic opening of cyclobutene-fused bicyclic systems. *Angew. Chem. Int. Ed.* **54,** 1527–1531; 10.1002/anie.201410115 (2015).
14. Duan, Y. *et al.* A Flavoprotein Dioxygenase Steers Bacterial Tropone Biosynthesis via Coenzyme A-Ester Oxygenolysis and Ring Epoxidation. *J. Am. Chem. Soc.* **143,** 10413–10421; 10.1021/jacs.1c04996 (2021).
15. Hu, Y., Bai, M., Yang, Y., Tian, J. & Zhou, Q. Rapid Access to Tetracyclic Core of Wortmannin via an Intramolecular Reductive Olefin Coupling Strategy. *Org. Lett.* **22,** 6308–6312; 10.1021/acs.orglett.0c02135 (2020).
16. Barba, F., Elinson, M. N., Escudero, J. & Feducovich, S. K. Indirect electrochemical oxidation of cyclic ketones: Strong influence of ring size on the result of the reaction. *Tetrahedron Lett.* **37,** 5759–5762; 10.1016/0040-4039(96)01177-x (1996).
17. Gallage, P. C., McKee, M. G. & Pitre, S. P. 1,4-Dihydropyridine Anions as Potent Single-Electron Photoreductants. *Org. Lett.* **26,** 1975–1979; 10.1021/acs.orglett.4c00513 (2024).
18. Fabry, D. C., Stodulski, M., Hoerner, S. & Gulder, T. Metal-free synthesis of 3,3-disubstituted oxoindoles by iodine(III)-catalyzed bromocarbocyclizations. *Chem. Eur. J.* **18,** 10834–10838; 10.1002/chem.201201232 (2012).
19. Sarkar, S. *et al.* Excited-State Copper-Catalyzed 4 + 1 Annulation Reaction Enables Modular Synthesis of α,β-Unsaturated-γ-Lactams. *J. Am. Chem. Soc.* **144,** 20884–20894; 10.1021/jacs.2c09006 (2022).
20. Takashi Harayama, Hiroko Toko, Hiromi Nishioka, Hitoshi Abe & Yasuo Takeuchi. Concise synthesis of tetrahydrophenanthridone by palladium reagent. *Heterocycles* **59,** 541–546; 10.3987/COM-02-S51 (2003).

21. Sasaki, I. *et al.* Iridium(I)-catalyzed vinylic C-H borylation of 1-cycloalkenecarboxylates with bis(pinacolato)diboron. *Chem. Commun.* **49,** 7546–7548; 10.1039/c3cc44149k (2013).

22. Wang, Y., Lin, C., Zhang, Z., Shen, L. & Zou, B. Directed Nickel-Catalyzed Selective Arylhydroxylation of Unactivated Alkenes under Air. *Org. Lett.* **25,** 2172–2177; 10.1021/acs.orglett.3c00085 (2023).

23. Brittain, W. D. G. & Cobb, S. L. Carboxylic Acid Deoxyfluorination and One-Pot Amide Bond Formation Using Pentafluoropyridine (PFP). *Org. Lett.* **23,** 5793–5798; 10.1021/acs.orglett.1c01953 (2021).

24. Myers, J. K. & Jacobsen, E. N. Asymmetric Synthesis of β-Amino Acid Derivatives via Catalytic Conjugate Addition of Hydrazoic Acid to Unsaturated Imides. *J. Am. Chem. Soc.* **121,** 8959–8960; 10.1021/ja991621z (1999).

25. Ayitou, A. J.-L. & Sivaguru, J. Light-induced transfer of molecular chirality in solution: enantiospecific photocyclization of molecularly chiral acrylanilides. *J. Am. Chem. Soc.* **131,** 5036–5037; 10.1021/ja9003552 (2009).

26. Cai, X.-P., Han, B.-H., Cen, F.-T., Qu, J.-P. & Kang, Y.-B. Endo/Exo-Controllable Photocyclization by EnT-SET-Switch. *Org. Lett.* **25,** 2863–2867; 10.1021/acs.orglett.3c00837 (2023).

27. Arcadi, A. *et al.* One-pot synthesis of dihydroquinolones by sequential reactions of o-aminobenzyl alcohol derivatives with Meldrum's acids. *Org. Biomol. Chem.* **20,** 3160–3173; 10.1039/d2ob00289b (2022).

28. Aranzaes, J. R., Daniel, M.-C. & Astruc, D. Metallocenes as references for the determination of redox potentials by cyclic voltammetry Permethylated iron and cobalt sandwich complexes, inhibition by polyamine dendrimers, and the role of hydroxy-containing ferrocenes. *Can. J. Chem.* **84,** 288–299; 10.1139/v05-262 (2006).

29. Pracht, P., Bohle, F. & Grimme, S. Automated exploration of the low-energy chemical space with fast quantum chemical methods. *Phys. Chem. Chem. Phys.* **22,** 7169–7192; 10.1039/c9cp06869d (2020).

30. Grimme, S. Exploration of Chemical Compound, Conformer, and Reaction Space with Meta-Dynamics Simulations Based on Tight-Binding Quantum Chemical Calculations. *J. Chem. Theory Comput.* **15,** 2847–2862; 10.1021/acs.jctc.9b00143 (2019).

31. Bannwarth, C. *et al.* Extended tight-binding quantum chemistry methods. *WIREs Comput Mol Sci* **11**; 10.1002/wcms.1493 (2021).

32. Bannwarth, C., Ehlert, S. & Grimme, S. GFN2-xTB-An Accurate and Broadly Parametrized Self-Consistent Tight-Binding Quantum Chemical Method with Multipole Electrostatics and Density-Dependent Dispersion Contributions. *J. Chem. Theory Comput.* **15,** 1652–1671; 10.1021/acs.jctc.8b01176 (2019).

33. Ehlert, S., Stahn, M., Spicher, S. & Grimme, S. Robust and Efficient Implicit Solvation Model for Fast Semiempirical Methods. *J. Chem. Theory Comput.* **17,** 4250–4261; 10.1021/acs.jctc.1c00471 (2021).

34. Grimme, S., Brandenburg, J. G., Bannwarth, C. & Hansen, A. Consistent structures and interactions by density functional theory with small atomic orbital basis sets. *J. Chem. Phys.* **143,** 54107; 10.1063/1.4927476 (2015).

35. Perdew, J. P., Burke, K. & Ernzerhof, M. Generalized Gradient Approximation Made Simple. *Phys. Rev. Lett.* **77,** 3865–3868; 10.1103/PhysRevLett.77.3865 (1996).

36. Grimme, S., Antony, J., Ehrlich, S. & Krieg, H. A consistent and accurate ab initio parametrization of density functional dispersion correction (DFT-D) for the 94 elements H-Pu. *J. Chem. Phys.* **132,** 154104; 10.1063/1.3382344 (2010).

37. Grimme, S., Ehrlich, S. & Goerigk, L. Effect of the damping function in dispersion corrected density functional theory. *J. Comput. Chem.* **32,** 1456–1465; 10.1002/jcc.21759 (2011).

38. Weigend, F. & Ahlrichs, R. Balanced basis sets of split valence, triple zeta valence and quadruple zeta valence quality for H to Rn: Design and assessment of accuracy. *Phys. Chem. Chem. Phys.* **7,** 3297–3305; 10.1039/b508541a (2005).

39. Grimme, S. *et al.* Efficient Quantum Chemical Calculation of Structure Ensembles and Free Energies for Nonrigid Molecules. *J. Phys. Chem. A* **125,** 4039–4054; 10.1021/acs.jpca.1c00971 (2021).

40. Balasubramani, S. G. *et al.* TURBOMOLE: Modular program suite for ab initio quantum-chemical and condensed-matter simulations. *J. Chem. Phys.* **152,** 184107; 10.1063/5.0004635 (2020).

41. TURBOMOLE V7.8 2023, a development of University of Karlsruhe and Forschungszentrum Karlsruhe GmbH, 1989-2007, TURBOMOLE GmbH, since 2007 & available from https://www.turbomole.org. ,

42. Sinnecker, S., Rajendran, A., Klamt, A., Diedenhofen, M. & Neese, F. Calculation of solvent shifts on electronic g-tensors with the conductor-like screening model (COSMO) and its self-consistent generalization to real solvents (direct COSMO-RS). *J. Phys. Chem. A* **110,** 2235–2245; 10.1021/jp056016z (2006).

43. Zhao, Y. & Truhlar, D. G. Design of density functionals that are broadly accurate for thermochemistry, thermochemical kinetics, and nonbonded interactions. *J. Phys. Chem. A* **109,** 5656–5667; 10.1021/jp050536c (2005).

44. Klamt, A. Conductor-like Screening Model for Real Solvents: A New Approach to the Quantitative Calculation of Solvation Phenomena. *J. Phys. Chem.* **99,** 2224–2235; 10.1021/j100007a062 (1995).

45. Eckert, F. & Klamt, A., COSMOtherm, Version C3.0, Release 13.01, COSMOlogic GmbH & Co. KG, Leverkusen, Germany, 2013 ,

46. Yanai, T., Tew, D. P. & Handy, N. C. A new hybrid exchange–correlation functional using the Coulomb-attenuating method (CAM-B3LYP). *Chem. Phys. Lett.* **393,** 51–57; 10.1016/j.cplett.2004.06.011 (2004).

47. Unsleber, J. P. *et al.* Serenity: A subsystem quantum chemistry program. *J. Comput. Chem.* **39,** 788–798; 10.1002/jcc.25162 (2018).

48. Niemeyer, N. *et al.* The subsystem quantum chemistry program Serenity. *WIREs Comput Mol Sci* **13**; 10.1002/wcms.1647 (2023).

49. Barton, D. et al. qcserenity/serenity: Release 1.6.1 (2024). Zenodo https://doi.org/10.5281/zenodo.10838411.

50. Neugebauer, H., Bohle, F., Bursch, M., Hansen, A. & Grimme, S. Benchmark Study of Electrochemical Redox Potentials Calculated with Semiempirical and DFT Methods. *J. Phys. Chem. A* **124,** 7166–7176; 10.1021/acs.jpca.0c05052 (2020).

51. Neese, F., Wennmohs, F., Becker, U. & Riplinger, C. The ORCA quantum chemistry program package. *J. Chem. Phys.* **152,** 224108; 10.1063/5.0004608 (2020).

52. Mena, L. D. *et al.* Quantitative prediction of excited-state decay rates for radical anion photocatalysts. *Chem. Commun.* **59,** 9726–9729; 10.1039/d3cc02534a (2023).

53. Lin, H. *et al.* Integrating Photoactive Ligands into Dimension-Reduced Metal–Organic Frameworks: Harnessing the Power of Organic Photocatalysts. *Acc. Mater. Res.* **5,** 236–248; 10.1021/accountsmr.3c00144 (2024).

54. Käfer, S., Niemeyer, N., Tölle, J. & Neugebauer, J. Triplet Excitation-Energy Transfer Couplings from Subsystem Time-Dependent Density-Functional Theory. *J. Chem. Theory Comput.* **20,** 2475–2490; 10.1021/acs.jctc.3c01365 (2024).

55. Becke, A. D. Density-functional thermochemistry. III. The role of exact exchange. *J. Chem. Phys.* **98,** 5648–5652; 10.1063/1.464913 (1993).

56. Lee, C., Yang, W. & Parr, R. G. Development of the Colle-Salvetti correlation-energy formula into a functional of the electron density. *Phys. Rev. B Condens Matter* **37,** 785–789; 10.1103/physrevb.37.785 (1988).

57. Lin, Y.-S., Li, G.-D., Mao, S.-P. & Chai, J.-D. Long-Range Corrected Hybrid Density Functionals with Improved Dispersion Corrections. *J. Chem. Theory Comput.* **9,** 263–272; 10.1021/ct300715s (2013).

58. Marenich, A. V., Cramer, C. J. & Truhlar, D. G. Universal solvation model based on solute electron density and on a continuum model of the solvent defined by the bulk dielectric constant and atomic surface tensions. *J. Phys. Chem. B* **113,** 6378–6396; 10.1021/jp810292n (2009).

59. Solé-Daura, A. & Maseras, F. Straightforward computational determination of energy-transfer kinetics through the application of the Marcus theory. *Chem. Sci.* **15,** 13650–13658; 10.1039/d4sc03352c (2024).

60. Pierloot, K., Dumez, B., Widmark, P.-O. & Roos, B. O. Density matrix averaged atomic natural orbital (ANO) basis sets for correlated molecular wave functions. *Theoret. Chim. Acta* **90,** 87–114; 10.1007/BF01113842 (1995).

61. Li Manni, G. *et al.* The OpenMolcas Web: A Community-Driven Approach to Advancing Computational Chemistry. *J. Chem. Theory Comput.* **19,** 6933–6991; 10.1021/acs.jctc.3c00182 (2023).

62. Pracht, P. & Bannwarth, C. Finding Excited-State Minimum Energy Crossing Points on a Budget: Non-Self-Consistent Tight-Binding Methods. *J. Phys. Chem. Lett.* **14,** 4440–4448; 10.1021/acs.jpclett.3c00494 (2023).

63. Isse, A. A. & Gennaro, A. Absolute potential of the standard hydrogen electrode and the problem of interconversion of potentials in different solvents. *J. Phys. Chem. B* **114,** 7894–7899; 10.1021/jp100402x (2010).

64. Gualandi, A. *et al.* Aluminum(III) Salen Complexes as Active Photoredox Catalysts. *Eur. J. Org. Chem.* **2020,** 1486–1490; 10.1002/ejoc.201901086 (2020).

65. Sheldrick, G. M. SHELXT – integrated space-group and crystal-structure determination. *Acta Crystallogr. A* **71,** 3–8; 10.1107/S2053273314026370 (2015).

66. Sheldrick, G. M. *SHELXL-2019/1* (Bruker AXS Inc., Madison, WI, USA, 2019).

67. Sheldrick, G. M. Crystal structure refinement with SHELXL. *Acta Crystallogr. C* **71,** 3–8; 10.1107/S2053229614024218 (2015).
